# Complete plastome sequence of *Iodes cirrhosa* Turcz., the first in the Icacinaceae, comparative genomic analyses and possible split of *Idoes* species in response to climate changes

**DOI:** 10.7717/peerj.6663

**Published:** 2019-04-01

**Authors:** Liqiang Wang, Hui Zhang, Mei Jiang, Haimei Chen, Linfang Huang, Chang Liu

**Affiliations:** Key Laboratory of Bioactive Substances and Resource Utilization of Chinese Herbal Medicine from Ministry of Education, Institute of Medicinal Plant Development, Chinese Academy of Medical Sciences and Peking Union Medical College, Beijing, China

**Keywords:** *Iodes cirrhosa* Turcz., Icacinaceae, Plastome, Selective pressure, Phylogenomic analysis

## Abstract

Plastome-based phylogenetic study has largely resolved the phylogeny of Icacinaceae. However, no single complete plastome sequence is available for Icacinaceae species, thereby limiting the further phylogenomics analysis of the members of this family. Here, we obtained the complete plastome sequence of *Iodes cirrhosa* Turcz., which is the first in Icacinaceae, by using the next-generation sequencing technology. The genome was annotated and compared with other closely related plastomes by using mVISTA. The divergence time of six *Iodes* species was analyzed using the BEAST software. The plastome of *I. cirrhosa* was 151,994 bp long, with a pair of inverted repeats (IRs, 24,973 bp) separated by a large single-copy (LSC, 84,527 bp) region and a small single-copy (SSC, 17,521 bp) region. The plastome encoded 112 unique genes, including 80 protein-coding, 28 tRNA, and four rRNA genes. Approximately 59 repeat sequences and 188 simple sequence repeats were identified. Four pairs of partially overlapped genes, namely, *psb*D/*psb*C, *ndh*F/Ψ*ycf*1, *atp*B/*atp*E, and *rpl*22/*rps*3, were observed. A comparison of the boundaries of the LSC, SSC, and IR regions with four other plastomes from Aquifoliales and Sapindales exhibited a high overall degree of sequence similarity. Four most highly variable regions, namely, *trn*H-GUG/*psb*A, *psb*M/*trn*D-GUC, *pet*A/*psb*J, and *rps*16/*trn*Q-UUG, were found. Using the plastome of *I. cirrhosa* as reference, we reassembled the plastomes of five *Iodes* species. *K*_a_/*K*_s_ ratio analyses revealed that 27 genes and 52 amino acid residue sites from 11 genes had undergone strong positive selection in the *Iodes* branch, with the most abundant proteins being the NDH and ribosomal proteins. Divergence-time analysis indicated that *Iodes* species were first formed 34.40 million years ago. Results revealed that the ancestor of the six species was likely to have split in the late Eocene epoch. In summary, the first complete plastome sequence of *I. cirrhosa* provided valuable information regarding the evolutionary processes of *Iodes* species.

## Introduction

*Iodes* is a genus belonging to the Icacinaceae family that contains many primary species of the tropics, such as trees, shrubs, and lianas. *Iodes* consists of 16 accepted species, as recorded in the Plant List (version 1.1, 2013, http://www.theplantlist.org); four of these species, namely, *Iodes cirrhosa* Turcz., *Iodes vitiginea* (Hance) Hemsl., *Iodes seguinii* (H.Lev.) Rehder, and *Iodes balansae* Gagnep, were found in China. *Iodes* species represent a rich source of potentially active compounds. For example, *I. cirrhosa* is a woody vine medicinal plant from the Icacinaceae family that is widely distributed in Guangxi and Yunnan Provinces in China and adjacent areas in Southeast Asia. *I. cirrhosa* has gained attention for its remarkable treatment effects on multiple diseases, such as rheumatism, nephritis, urine acerbity, and swelling pain (Gan et al., 2010). Modern pharmacology studies have shown that liposoluble constituents from *I. cirrhosa* have neuroprotective and K channel-blocking activity, and glycosides from the same species present activity against glutamate-induced PC12 cell damage (Gan et al., 2011; Gan et al., 2010). Hence, understanding the phylogenetic relationships and plastome evolution of *Iodes* species will likely lead to the discovery of novel active compounds produced from this group. Our original goal was to conduct phylogenetic analyses of the plastomes of *Iodes* species. Unfortunately, after extensive effort, we failed to obtain the materials for these samples, other than that of *I*. *cirrhosa*. Consequently, we focused on *I. cirrhosa* in this study.

Previous studies showed that Icacinaceae is highly polyphyletic (Lens et al., 2008). Kårehed (2001) conducted the first family-wide phylogenetic investigation of species from Icacinaceae on the basis primarily of *ndh*F sequence, with a sparse sampling of several other loci (*rbc*L, *atp*B, and 18S rDNA). Stull et al. (2015) sequenced 50 plastid genomes across the core asterid, focusing on the basal lamiid genera. Combining their data with other available asterid plastome data, this research group resolved the basal lamiid phylogeny. Several new orders, including Icacinales, have been recognized in the Angiosperm Phylogeny Group classification of the orders and families of angiosperms partly because of this study (The Angiosperm Phylogeny Group et al., 2016). With the resolution of the phylogenetic position of Icacinaceae, the next logical steps would be to resolve the relationships among species and genera within Icacinaceae.

While the nuclear genome may contain numerous potentially useful markers, the plastome sequences have been widely used in resolving phylogenetic relationship and discovering novel molecular markers. The highly conservative nature and slow evolutionary rate of the plastome make it uniform enough in conducting comparative studies across different species but divergent sufficiently to capture evolutionary events, including insertions, deletions, reversals, and translocations. These characteristics make plastome sequence a suitable and invaluable tool in studying molecular phylogeny, barcode identification, plant evolution, and phylogenetics (Dong et al., 2012; Dong et al., 2015; Dong et al., 2017). The recent development of next-generation sequencing (NGS) technologies have significantly reduced the time and cost associated with the sequencing of an entire plastome for nonmodel species. The availability of thousands of plastome sequences and the improvement in bioinformatics analysis methods allows the assembly of the complete plastome sequences without the need to isolate the plastids from the cells experimentally and then sequence the plastid DNA (Kane et al., 2012; Nock et al., 2011).

Although the plastome sequences have been used successfully to resolve the basal lamiid phylogeny (Stull et al., 2015), the sequencing reads have not been assembled to obtain complete plastome sequences. The corresponding protein sequences used for the phylogenetics analysis are largely fragmental. The availability of complete plastome sequences may be useful in several ways. First, complete plastome sequences can be used as the reference to obtain the proteome and genome sequences of plastids from other related species. Second, complete plastome sequences can be used to resolve phylogenetic relationships among low taxonomic levels. Third, molecular markers can be identified to distinguish related species closely.

In this study, we sequenced the complete plastome of *I. cirrhosa* by using the genome skimming strategy and then analyzed the genome in detail. First, we identified all protein-coding, tRNA, and rRNA genes and several sequence repeats. Second, by using the complete plastome of *I. cirrhosa* as a reference, we reassembled the NGS reads from the study of Stull et al. (2015). Consequently, much better plastome assemblies and more complete proteomes for five *Iodes* species were obtained than those of the previous reports. Third, we compared the *I. cirrhosa* plastome with its most closely related species from Aquifoliales and Sapindales. Fourth, several intergenic regions were selected for the identification of candidate molecular barcodes. Fifth, we identified positively selected genes and sites during the evolution of *Iodes* species. Finally, divergence time was estimated for the six *Iodes* species, and a possible split was identified. In summary, these results may help further understand the evolutionary history of the *Iodes* clade and facilitate the biospecting of the active compounds from this plant group.

## Materials and Methods

### Plant materials and total DNA purification

The fresh leaves of *I. cirrhosa* were collected from the Institute of Medicinal Plant Development in Guangxi Province, China and frozen at −80 °C. Total DNA was extracted using a plant genomic DNA extraction kit (Tiangen Biotech, Beijing, China). DNA quality was assessed by electrophoresis in 1% (w/v) agarose gel, and their quantity was examined using Qubit 3.0 (Life Technologies, Carlsbad, CA, USA).

### Plastome sequencing, assembly, and annotation

Approximately 500 ng DNA was used to construct a library with an insert size of 150 bp and sequenced according to the manufacturer’s instructions for HiSeq 2500 platform (Illumina Inc., San Diego, CA, USA). The raw sequence data were preprocessed according to the following steps: (1) removal of the adaptor sequences; (2) removal of reads that are <140 bp long; (3) removal of those bases before the first base acquired a quality score of <20 from the 5′ of each read; and (4) removal of those bases after the first base acquired a quality score of <20 at 3′ of each read. Consequently, 6,353,850 reads were obtained, with the total length of 1.91 Gb. These clean paired-end reads were filtered against all the plastomes of plants recorded in the GenBank by using BLASTN with an *e*-value cut-off of 1*e*−5. The extracted reads were assembled using AbySS (v. 1.5.2), and the resulting contigs were extended by Sanger sequencing by using sequence-specific primers (Table S1). The correctness of the complete draft plastome was validated by mapping all raw reads to the draft plastome by using Bowtie 2 (v. 2.0.1) (Langmead, 2010) and eyeballing the read coverage by using Tablet (v. 1.14.10.20; Milne et al., 2010). Genes were annotated by CpGAVAS web service (Liu et al., 2012) and edited manually by Apollo genome editor (Lewis et al., 2002). The circular map was generated using OrganellarGenomeDRAW (Lohse, Drechsel & Bock, 2007). Codon usage and GC content were also analyzed using the Cusp and Compseq programs from EMBOSS (Chojnacki et al., 2017). The raw sequencing results and the genome assembly and annotation results have been deposited in GenBank, with the accession numbers SRR8503885 and NC_036304, respectively.

### Comparative genome analyses

The complete plastomes of *I. cirrhosa* were compared with four other species, namely, *Helwingia himalaica* (Aquifoliales, NC_031370), *Ilex paraguariensis* (Sapindales, KP016928), *Sapindus mukorossi* (Sapindales, NC_025554.1), and *Euonymus japonicus* (Sapindales, NC_028067), by using the software mVISTA in Shuffle-LAGAN mode. The plastome of *I. cirrhosa* served as a reference genome in comparative analyses. To identify the most divergent regions among the congeneric species, we defined the sequences of the same regions from the congeneric species as mutually most similar sequences after the comparison with the BLASTN program. Then, the sequences were subjected to multiple sequence alignment by using ClustalW2 (Chenna et al., 2003). The Kimura two-parameter distance was calculated using the distmat program from the EMBOSS package (Chojnacki et al., 2017).

### Repeat sequence analysis

The REPuter (http://vmatch.de/) web service was used to identify the size and location of the four types of repeats, including forward, palindromic, reverse, and complement repeats in five species (Kurtz et al., 2001). The minimum repeat size was set to 30 bp, and the cut-off for the similarities among the repeat units was 90%. Simple sequence repeats (SSRs) were predicted using MISA Perl Script, with the following thresholds: eight units of mononucleotides; four units of di- and trinucleotides; and three units of tetra-, penta-, and hexanucleotides (Beier et al., 2017). Tandem repeats were identified using the Tandem Repeats Finder (Castelo, Martins & Gao, 2002), with the following parameter settings: matches = 2, mismatches and indels = 7, minimum alignment score = 50, maximum period size = 500, minimum repeat size = 9 bp, and the cut-off for similarities among the repeat units = 70%.

### Reconstruction of the plastomes and proteomes of the five *Iodes* species

To conduct the phylogenetic analyses of the *Iodes* species, we downloaded the reads of five other *Iodes* species from the NCBI SRA database, with the following run accession numbers: *Iodes klaineana* (SRR2401796), *Iodes liberica* (SRR2401797), *Iodes perrieri* (SRR2401798), *Iodes scandens* (SRR2401799), and *Iodes seretii* (SRR2401800). The plastomes of the five species were assembled following the procedure described above, with the plastome sequences of *I. cirrhosa* as the reference. To obtain shared proteins among the *Iodes* species, we utilized the protein sequences translated from the plastome of *I. cirrhosa* as the reference sequences in extracting the shared proteins from the contigs of the five *Iodes* species by using custom Python scripts. Briefly, the contig sequences of each *Iodes* species were searched against the full-length reference sequences of the *I*. *cirrhosa* proteins by using the BLASTX with an *e*-value cut-off of 1*e*−5. These hit sequences were assembled using the program Phrap. Then, the assembled contigs were compared against the reference protein sequences by using the BLASTX with the cut-off *e*-value of 1*e*−5. The fragments corresponding to those found in the high-scoring pairs were concatenated together and used as the corresponding protein sequences for the corresponding *Iodes* species.

### Phylogenetics analyses

Seventy-four protein sequences shared by the six *Iodes* species, including ACCD, ATPA, ATPB, ATPE, ATPF, ATPH, ATPI, CCSA, CEMA, CLPP, MATK, NDHA, NDHB, NDHC, NDHD, NDHE, NDHF, NDHG, NDHH, NDHI, NDHJ, NDHK, PETA, PETB, PETD, PETG, PETL, PETN, PSAA, PSAB, PSAC, PSAI, PSAJ, PSBA, PSBB, PSBC, PSBD, PSBE, PSBF, PSBH, PSBI, PSBK, PSBL, PSBM, PSBN, PSBT, PSBZ, RBCL, RPL14, , RPL2, RPL20, RPL22, RPL23, RPL32, RPL33, RPOA, RPOB, RPOC1, RPOC2, RPS11, RPS12, RPS14, RPS15, RPS16, RPS18, RPS19, RPS2, RPS3, RPS4, RPS7, YCF1, YCF2, YCF3, and YCF4, were extracted. The 74 protein sequences were concatenated and aligned using the ClustalW program (Corpet, 1988). The phylogenetic trees were constructed using the software Randomized Axelerated Maximum Likelihood (RAxML) and the maximum likelihood (ML) method, with *Arabidopsis thaliana* and *Nicotiana tabacum* as the outgroups, respectively. The detailed parameters were “raxmlHPC-PTHREADS-SSE3 -fa -N 1000 -m PROTGAMMACPREV/GTRGAMMA -x 551314260 -p 551314260 -o *Arabidopsis_thaliana*, *Nicotiana_tabacum* -T 20”. The significant level of the phylogenetic tree was assessed by bootstrap testing with 1,000 replications. The bootstrap values of each branch were shown beyond each node.

### Selective pressure analyses

On the basis of the ML tree, we conducted selective pressure analysis by using the adaptive branch-site random effects likelihood (aBSREL) model (Smith et al., 2015) that is implemented in HyPhy (https://veg.github.io/hyphy-site/getting-started/#characterizing-selective-pressures). Significance was assessed using the likelihood ratio test at a threshold of *p* ≤ 0.05 after correcting for multiple testing implemented in the program. The sites that were subject to positive selection in the *Iodes* genus branch were identified using the codeml program implemented in PAML (v. 4.9; Yang & Nielsen, 2002).

### Divergence-time estimation

Then, the ML tree and the alignment of the 74 shared proteins were loaded into the BEAST software (v. 1.8.4; Drummond et al., 2012) for the divergence-time estimation using the cpREV substitution model, which is a model of amino acid substitution for proteins encoded by chloroplast DNA (Adachi et al., 2000). The 1,000,000 generations underwent MCMC analysis, in which every 1,000 generations were sampled under a strict clock approach by using the Yule speciation tree prior with the substitution rate. The 93 and 107 million years ago (Mya) were used as the lower and upper boundaries of the lamiid species splitting time in calibrating the clock (He et al., 2016; Hedges, Dudley & Kumar, 2006). TRACER software (v. 1.6; Rambaut & Drummond, 2007) was used to check the effective population size of >200. The default settings were used otherwise when performing MCMC TREE analysis. TREEANNOTATOR software (v. 1.8.4; Bouckaert et al., 2014) was used to produce the maximum clade credibility trees from the trees after burning-in of 10%. The chronogram of the tree with the highest credibility was visualized in FigTree (v. 1.4.3; http://tree.bio.ed.ac.uk/software/figtree/, November 2018).

## Results

### General features of the *I. cirrhosa* plastome

**Figure 1:**
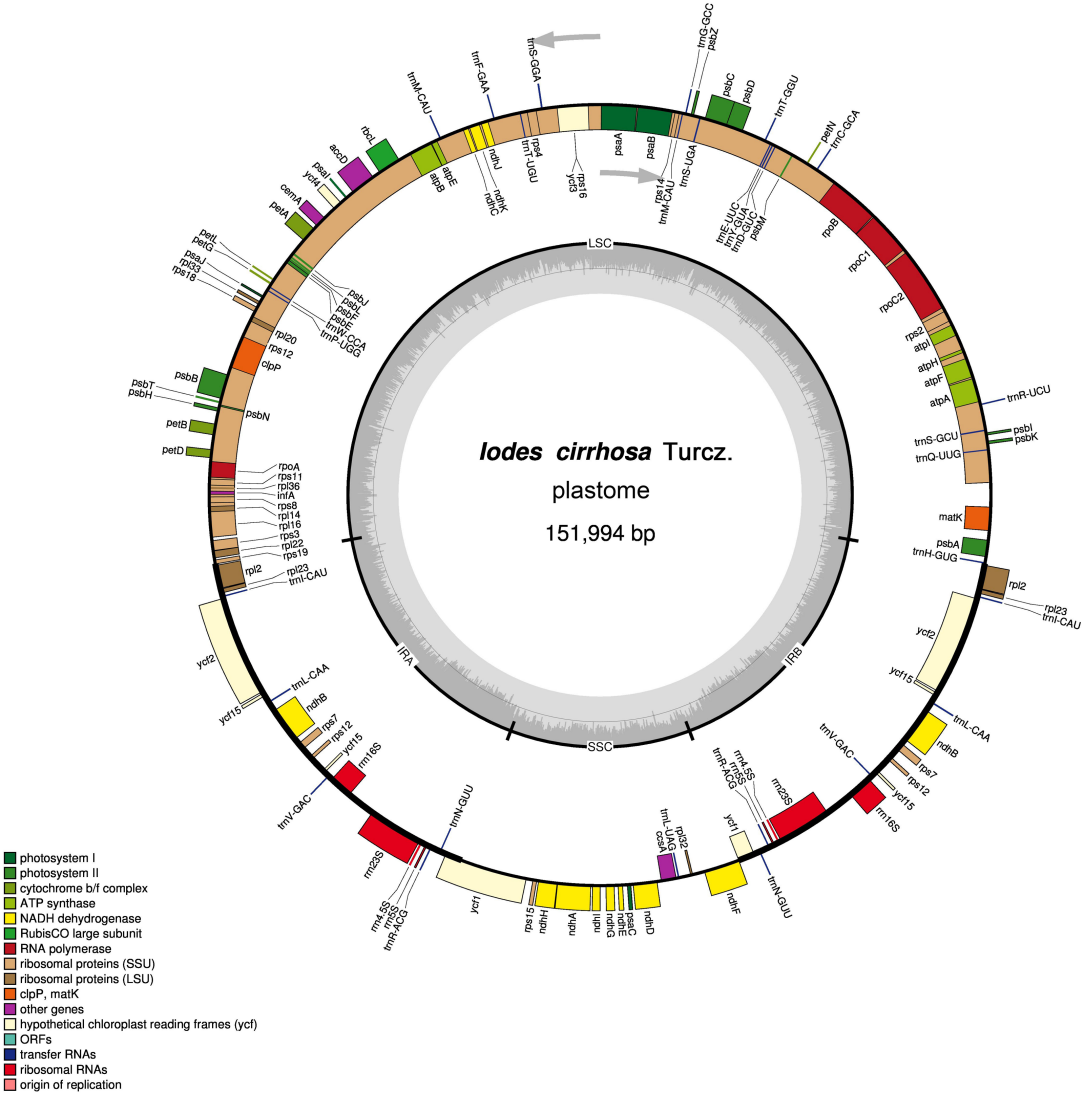
Schematic representation of the *I. cirrhosa* plastome. The outer circle shows the distribution of various genes on the genome, whose color indicate their functional classifications. The genes drawn outside the circle are transcribed clockwise, whereas those drawn inside are transcribed counterclockwise. The inner circle shows the distribution of the GC contents along the genome and is divided into the large single copy (LSC) region, small single copy (SSC) region and two inverted repeat (IR) regions, IRA and IRB.

The assembled plastome of *I. cirrhosa* was 151,994 bp long. A total of 582,754 (4.1%) out of the 14,176,506 paired-end sequencing reads were successfully mapped to the *I. cirrhosa* plastome, thereby yielding approximately 508-fold average coverage. The genome possessed a typical quadripartite structure with a pair of inverted repeat (IR) regions of 24,973 bp separated by a large single-copy (LSC) region of 84,527 bp and a small single-copy (SSC) region of 17,521 bp (Fig. 1). Overall, 47.33%, 1.86%, and 6.76% of the plastome sequence-encoded proteins, tRNAs, and rRNAs, respectively, whereas the remaining 44.05% corresponded to the noncoding region, including introns, intergenic spacers, and pseudogenes. The genome contained 134 genes in total, corresponding to 112 unique genes encoding 80 proteins, 28 tRNAs, and 4 rRNAs (Table 1 and Dataset S1). Seven genes (i.e., *rpl*2, *rpl*23, *ycf*2, *ycf*15, *ndh*B, *rps*7, and *rps*12) and all rRNA genes were identified in the IR regions (Fig. 1). The genome also had 21 intron-containing genes. Among these genes, 11 protein-coding genes and 8 tRNA genes had only one intron in each of them. Two protein-coding genes, namely, *clp*P and *ycf*3, had two introns (Table S2). Two genes (i.e., *trn*G-UCC and *rps*12) lost their introns compared with those in the Sapindales species (Wang, Chen & Zhang, 2017; Zhang et al., 2016). The *rps*12 gene is a special trans-splicing gene. The 5′ exon of the gene was located in the LSC region, whereas the 3′ exon of the gene was located in the IR region. The trans-spliced gene *rps*12 has also been found in other plant species, such as *Olea europaea* (Mariotti et al., 2010). Four pairs of overlapping genes (i.e., *psb*D/*psb*C, *atp*E/*atp*B, *rps*3/*rpl*22, and *ndh*F/Ψ*ycf*1) were identified, which is similar to those found in the Sapindales species.

**Table 1:** Genes predicted in the plastome of *I. cirrhosa*.

**Group of name**	**List of predicted genes**
Photosystem I	*psa*A, B, C, I, J
Photosystem II	*psb*A, B, C, D, E, F, H, I, J, K, L, M, N, T, Z
Cytochrome b/f complex	*pet*A,B,D*,*G,L,N
ATP synthase	*atp*A, B, E, F, H, I
NADH-dehydrogenase	*ndh*A, B (×2), C, D, E ,F, G, H, I, J, *K*
Rubisco large subunit	*rbc*L
RNA polymerase	*rpo*A, B, *C*1, *C*2
Small subunit of ribosome	*rps*2, 3, 4*,*7 (×2), 8*,* 11, 12, 14, 15, 16, 18*,* 19
Large subunit of ribosome	*rpl*16, 2 (×2), 14, 20, 22, 23 (×2), 32, 33, 36
Proteins of unknown function	*ycf*1 (×2), *ycf*2 (×2), *ycf*3, *ycf*4, *ycf*15 (×4)
Other proteins	*acc*D, *ccs*A, *cem*A, *clp*P, *inf*A, *mat*K
Ribosomal RNAs	*rrn*16S (×2), 23S (×2), 4.5S (×2), 5S (×2)
Transfer RNAs	*trn*A(UGC) (×2), C(GCA)*,* C(ACA)*,* D(GUC), E(UUC) (×3), F(GAA)*,* G(GCC), H(GUG)*,* I(CAU) (×2), K(UUU), L(CAA) (×2), L(UAA)*,* L(UAG), M(CAU) (×4), N(GUU) (×2)*,* P(UGG), Q(UUG)*,* R(ACG) (×2)*,* R(UCU), S(UGA)*,* S(GCU)*,* S(GGA), T(UGU)*,* T(GGU)*,* T(CGU), V(GAC) (×2), W(CCA)*,* Y(GUA)

**Notes.**

Numbers in the parentheses represent the number of copies.

LSC large single copy region SSC small single copy region IR Inverted repeat region CDS coding sequence

The GC content of the entire plastome sequence was 37.44%, which was lower than that of the IR regions (43.0%) and higher than those of the LSC (35.45%) and SSC regions (31.2%, Table S3). This result suggested that the LSC, SSC, and IR regions may have different origins. The plastome contained 89 protein-coding genes, with 73,620 nucleotides forming 24,540 nonstop codons and 267 nucleotides forming 89 stop codons (Table S4). Among the 24,629 stop and nonstop codons, those for leucine and isoleucine presented the highest frequencies of 2,641 (10.72%) and 2,084 (8.48%), respectively. The codons for cysteine showed the lowest frequencies of 284 (1.15%, Table S5). Given that only 28 tRNA gene types were found in the plastome, at least 36 types of tRNAs coded in the nuclear genomes were required to translate all the proteins encoded in the plastome. The most abundant types of codons (Table S4) had no apparent correlation with the most abundant types of tRNA genes (Table 1). Within the CDS of the plastome, the A/T contents for the first, second, and third codon positions were 55.30%, 60.75%, and 69.65%, respectively (Table S3). A bias of high A/T ratio existed at the third codon position, which is similar to other land plants (Nie et al., 2014).

### Repeat sequences and SSR analyses

**Figure 2:**
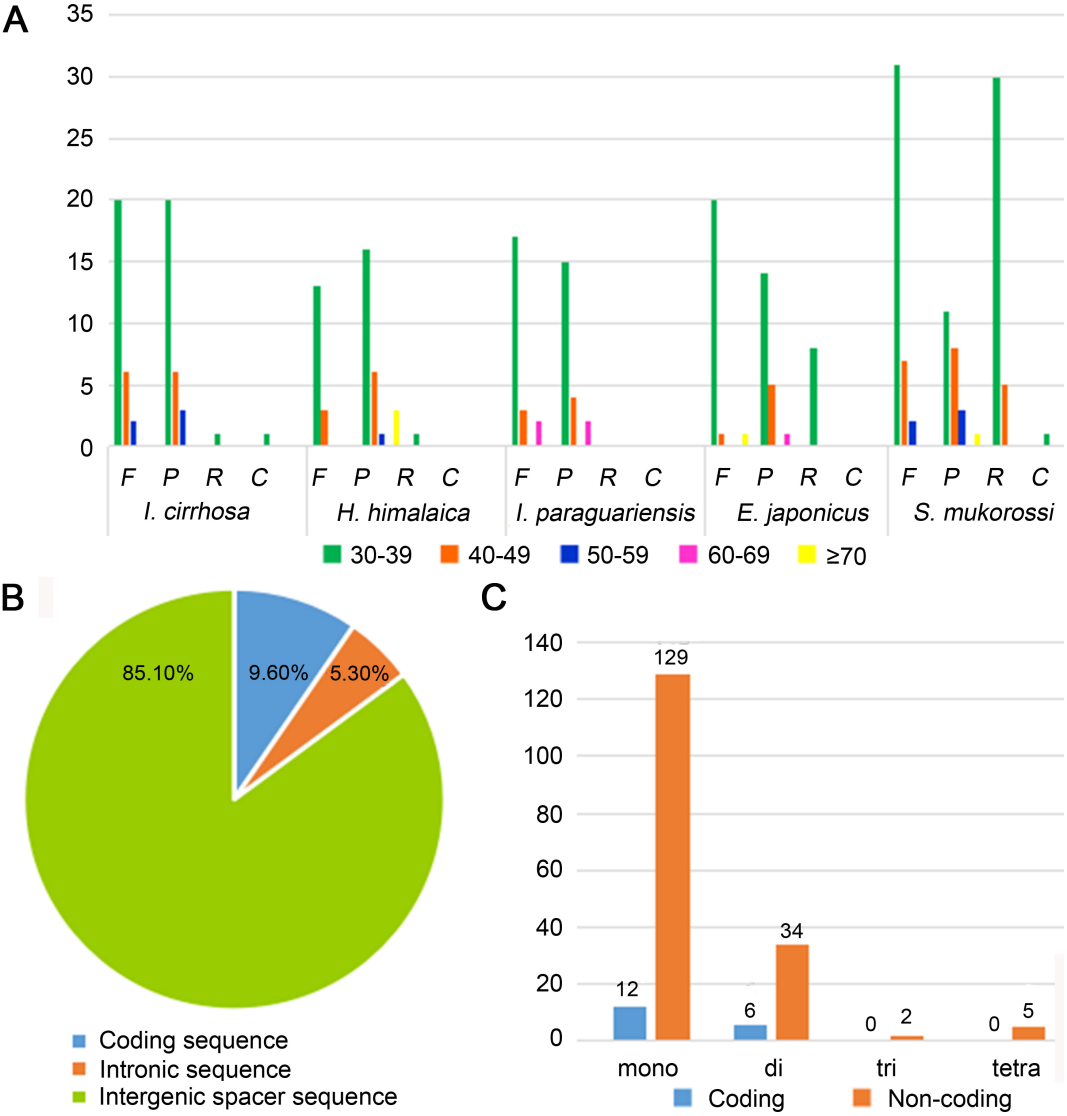
Statistics of repeat sequences detected in the plastome of *I. cirrhosa*. (A) Repeat sequences identified in the five plastomes. F, Forward; P, Palindrome; R, Reverse; C, Complement. (B) SSRs distribution among three different types of sequences: coding, intragenic and intergenic spacer sequences. (C) Different types of SSRs distribution among coding and non-coding regions.

Repeat sequences play an important role in genetic diversity, such as genome length variation and structural rearrangement (Jo et al., 2011). We analyzed the repeat structures in the plastomes of four species that are most closely related to *I. cirrhosa* and those of *I. cirrhosa* (Fig. 2A). Overall, the relative abundance of each type of repeats (i.e., forward, palindrome, reverse, and complement) was similar among the five species. The number of repeats for these five species may change once the complete plastome sequences for these species become available. In the plastome of *I. cirrhosa*, we detected 59 repeat sequences consisting of 28 forward, 29 palindromic, 1 reverse, and 1 complement repeats, with the length of >30 bp and similarities of >90% (Table S6). A total of 34 pairs of repeats were located in the *ycf*2 gene alone, which may be associated with the rapid evolution of this protein.

Tandemly repeated DNA sequences consisting of 1–6 nucleotide repeat units are called SSRs and also known as microsatellites that are distributed throughout the genomes (Powell et al., 1995). SSRs have been widely used as molecular markers in the studies of population genetics, species identification, and phylogenetic investigations on the basis of their high-degree variations (Provan et al., 1997). A total of 188 SSRs were identified in the plastomes of *I. cirrhosa* (Table S7). The most abundant types of SSRs are shown in Table 2. The SSRs were mostly distributed in the IGS and intron sequences (Fig. 2B). Among these SSRs, the majority is mononucleotide repeats (Fig. 2C). As shown in Table 2, most mononucleotide repeats consisted of A/T repeats (97.2%), whereas most dinucleotide repeats consisted of AT/AT (65%). These results agreed with those of the previous report, wherein SSRs from plastomes are generally composed of short polyA or polyT repeats and rarely contain tandem G or C repeats in many plants (Wang, Shi & Gao, 2013). In rare cases, SSRs have also been found in the coding regions. For example, 18 SSRs were identified in the CDS of 11 genes in *I. cirrhosa*, namely, *mat*K, *rpo*A, *rpo*B, *rpo*C1, *rpo*C2, *atp*B, *rps*7, *rps*19, *ndh*B, *ccs*A, and *ycf*2.

**Table 2:** Frequency of classified repeat types.

**SSR repeats Type**	**Units**	**Number of copies**																
		**3**	**4**	**5**	**6**	**7**	**8**	**9**	**10**	**11**	**12**	**13**	**14**	**15**	**16**	**17**	**18**	**Total**
Mono	A/T	–	–	–	–	–	58	33	26	11	3	2	1	1	1	–	1	137
	C/G	–	–	–	–	–	1	3	–	–	–	–	–	–	–	–	–	4
Di	AC/GT	–	1	–	–	–	–	–	–	–	–	–	–	–	–	–	–	1
	AG/CT	–	12	1	–	–	–	–	–	–	–	–	–	–	–	–	–	13
	AT/AT	–	19	5	1	–	1	–	–	–	–	–	–	–	–	–	–	26
Tri	AAG/CTT	–	1	–	–	–	–	–	–	–	–	–	–	–	–	–	–	1
	AAT/ATT	–	1	–	–	–	–	–	–	–	–	–	–	–	–	–	–	1
Tetra	AAAC/GTTT	1	–	–	–	–	–	–	–	–	–	–	–	–	–	–	–	1
	AAAG/CTTT	1	–	–	–	–	–	–	–	–	–	–	–	–	–	–	–	1
	AATC/ATTG	1	–	–	–	–	–	–	–	–	–	–	–	–	–	–	–	1
	AATG/ATTC	1	–	–	–	–	–	–	–	–	–	–	–	–	–	–	–	1
	AATT/AATT	1	–	–	–	–	–	–	–	–	–	–	–	–	–	–	–	1

The tandemly repeated DNA sequences with the size of repeat units longer than those of the SSR were identified using the Tandem Repeats Finder (Table S8). The following information, such as the start position, end position, size of repeat unit, numbers of repeat units, pattern, percent of matches between adjacent copies, sequences of the repeat unit, and the sequence of the entire repeat, are shown. In total, 30 repeats were identified. The repeat sizes ranged from 9 bp to 33 bp, with an average of 18.9 bp. The number of repeat units in the repeat sequences ranged from 1.9 to 12.6 with an average of 3.48. The overall percentages of matches among adjacent copies ranged from 70% to 100%, with an average of 91.1%. The corresponding alignment scores among adjacent repeat units ranged from 50 to 183, with an average of 91.1. The results identified using REPuter and Tandem Repeats Finder exhibited some overlaps. The differences among the results were due to the difference in the algorithm and parameter of each program used.

### Comparative genome analyses

**Figure 3:**
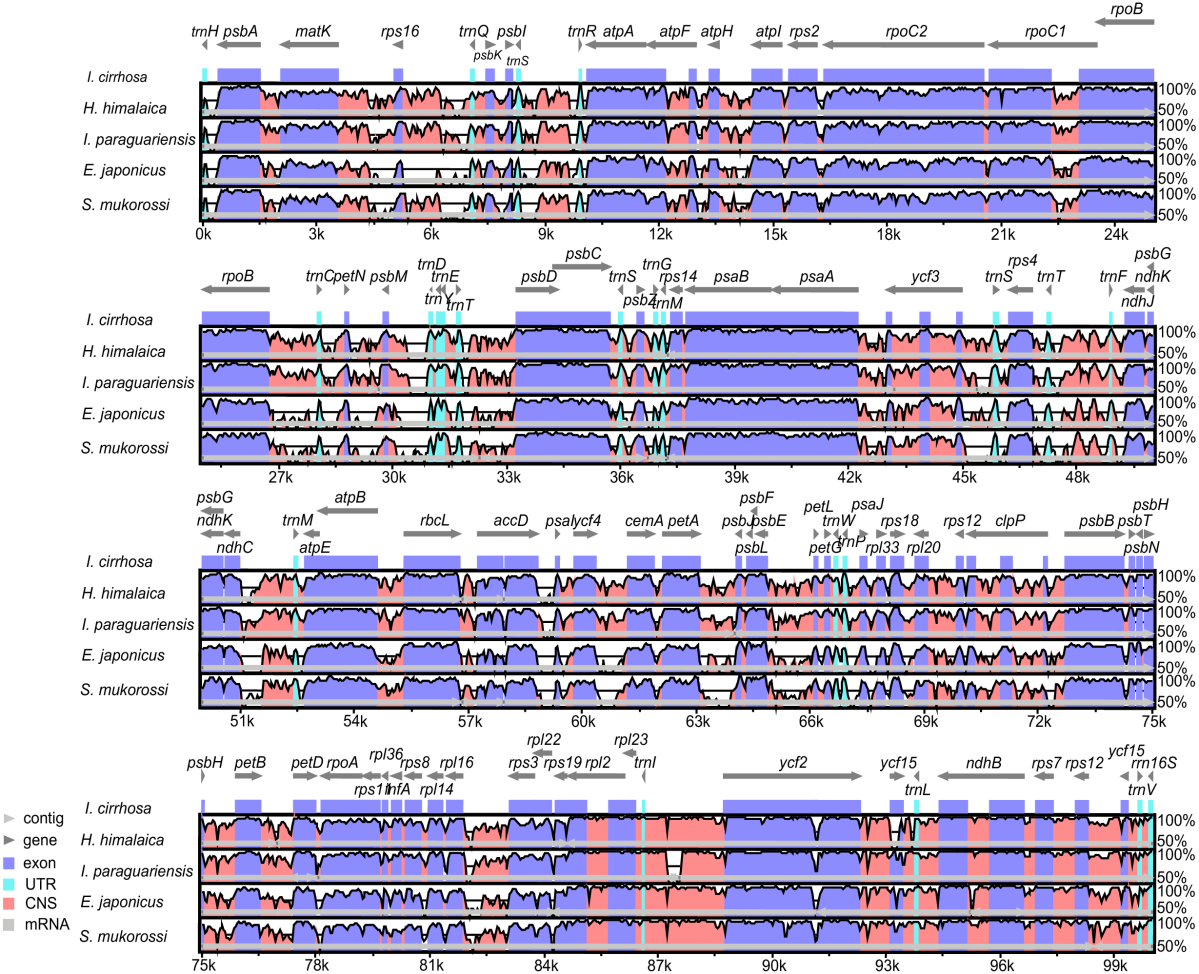
Identity plot comparing five species plastomes using *I. cirrhosa* as the reference. The vertical scale, ranging from 50% to 100%, indicates the percentage of identity calculated in sliding windows. The horizontal axis indicates the coordinates within the plastome. Different colors correspond to the types of the genome regions, Blue, regions coding for proteins; Pink, regions that are non-coding; Light blue, regions coding for tRNAs and rRNAs. Light horizontal grey arrows represent genes.

The plastome of *I. cirrhosa* was compared with four other species, namely, *I. paraguariensis, S. mukorossi*, *E. japonicas*, and *H. himalaica* (Fig. 3) because these species were the most closely related whose plastome sequences were available at the time of this study. Among these species, *I. paraguariensis* and *S. mukorossi* are from Sapindales, *E. japonicus* are from Celastrales, and *H. himalaica* are from Aquifoliales. The comparison revealed that the length of the plastome of *I. cirrhosa* was the shortest in the five plastomes. While various lengths of the plastomes of these plants are mainly attributed to the varied length of their LSC and IR regions, the lengths of all LSC, SSC, and IR regions of *I. cirrhosa* were the shortest among the five plastomes.

The genome organizations and sequences from the five plastomes are highly conserved, as expected in angiosperms (Dong et al., 2013). Overall, the IR regions were more conserved than the LSC and SSC regions. Similarly, the coding regions were more conserved than their noncoding counterparts. To identify the regions that were more likely to contain markers that can distinguish these congeneric species, we calculated the Kimura two-parameter distances for sequences from each pair of the species by using the distmat programs from the EMBOSS package. The distances for the most divergent regions are shown in Table S9. The most divergent coding regions among the five plastomes were *ycf*1 (2.11 ± 1.49), *ycf*2 (1.07 ± 1.32), *ndh*F (15.55 ± 10.16), *rbc*L (1.27 ± 0.35), *acc*D (2.73 ± 2.14), and *psa*A (39.74 ± 39.48) genes. By contrast, the noncoding regions, such as *trn*H-GUG/*psb*A (85.61 ± 71.57), *psb*M/*trn*D-GUC (5.58 ± 3.73), *pet*A/*psb*J (37.89 ± 34.05), and *rps*16/*trn*Q-UUG (32.75 ± 18.87), showed the highest degree of sequence divergence. The numbers in the parentheses were the mean ± standard deviation of the Kimura two-parameter distances for each region. Potential molecular markers for genetic diversity and evolutionary studies can be identified from these regions (Shaw et al., 2007). However, the molecular markers from these regions need to be further identified and verified experimentally.

### Boundary analysis of *I. cirrhosa* plastome

**Figure 4:**
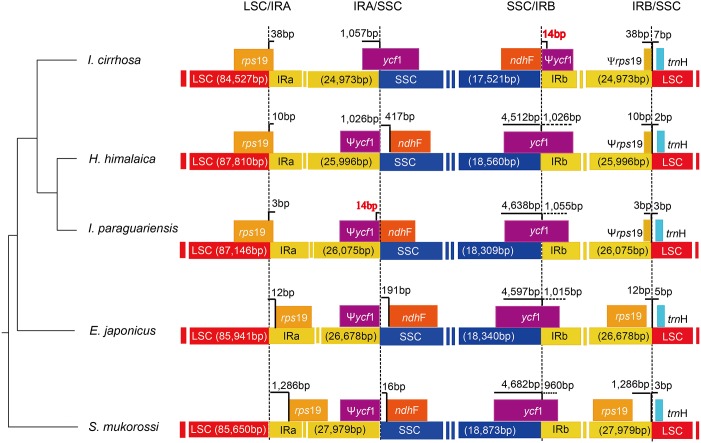
Analysis of the plastomic structure at the LSC/IR and SSC/IR boundary regions. Sequences from *I. cirrhosa, H. himalaica, I. paraguariensis, E. japonicus* and *S. mukorossi* are shown. The phylogenetic relationships of these five species are shown on the left. For each species, a long horizontal strip is used to represent the overall structure of the plastome. The dashed vertical lines mark either the 5′ end or 3′ end of the genes at the junctions. The solid vertical lines indicate the actual junctions. “Ψ” indicates that the corresponding gene is a pseudogene. The length of overlap gene is labeled by red in *I. cirrhosa* and *I. paraguarie*.

The causes of variation in the size of plastomes include the gains and losses of genes and introns (Delannoy et al., 2011), the expansion/contraction of the IR regions (Luo et al., 2014; Zhang et al., 2013), and major structural rearrangements, such as inversions (Walker, Zanis & Emery, 2014) and transpositions (Cosner et al., 1997). The boundary regions of *I. cirrhosa*, *I. paraguariensis*, *S. mukorossi*, *E. japonicus*, and *H. himalaica* (Fig. 4) were compared with to illustrate the evolutionary history of the IR boundaries. In the *I. cirrhosa* plastome, *rps*19 gene was located at the LSC/IRa junction, and only 38 bp of *rps*19 was duplicated in the IRb region. Similarly, *ycf*1 gene was located at the junction of SSC/IRa, and 1,057 bp of *ycf*1 was duplicated in the IRa region. The incomplete genes of *rps*19 and *ycf*1 in the IR regions were pseudogenes. These boundaries generally fluctuated among different plastomes. For example, *rps*19 was located inside the IR regions in the plastomes of *S. mukorossi* and *E. japonicus*. The *ndh*F gene spanned across the junction of SSC/IR in *I. cirrhosa*, which is the same as that observed in *I. paraguariensis.* The *ndh*F gene and *ycf*1 pseudogene had 14 bp long overlap (Fig. 4). In the plastomes of *E. japonicas*, *H. himalaica*, and *S. mukorossi*, the *ndh*F gene was located inside of the SSC regions completely. The SSC region of the *I. cirrhosa* plastome was in an inverted configuration, which is similar to Dioscoreaceae plastomes (Hansen et al., 2007) but different from those of *I. paraguariensis*, *S. mukorossi*, *E. japonicus*, and *H. himalaica*.

### Reconstruction of the plastomes and plastid proteomes of *Iodes* species

**Figure 5:**
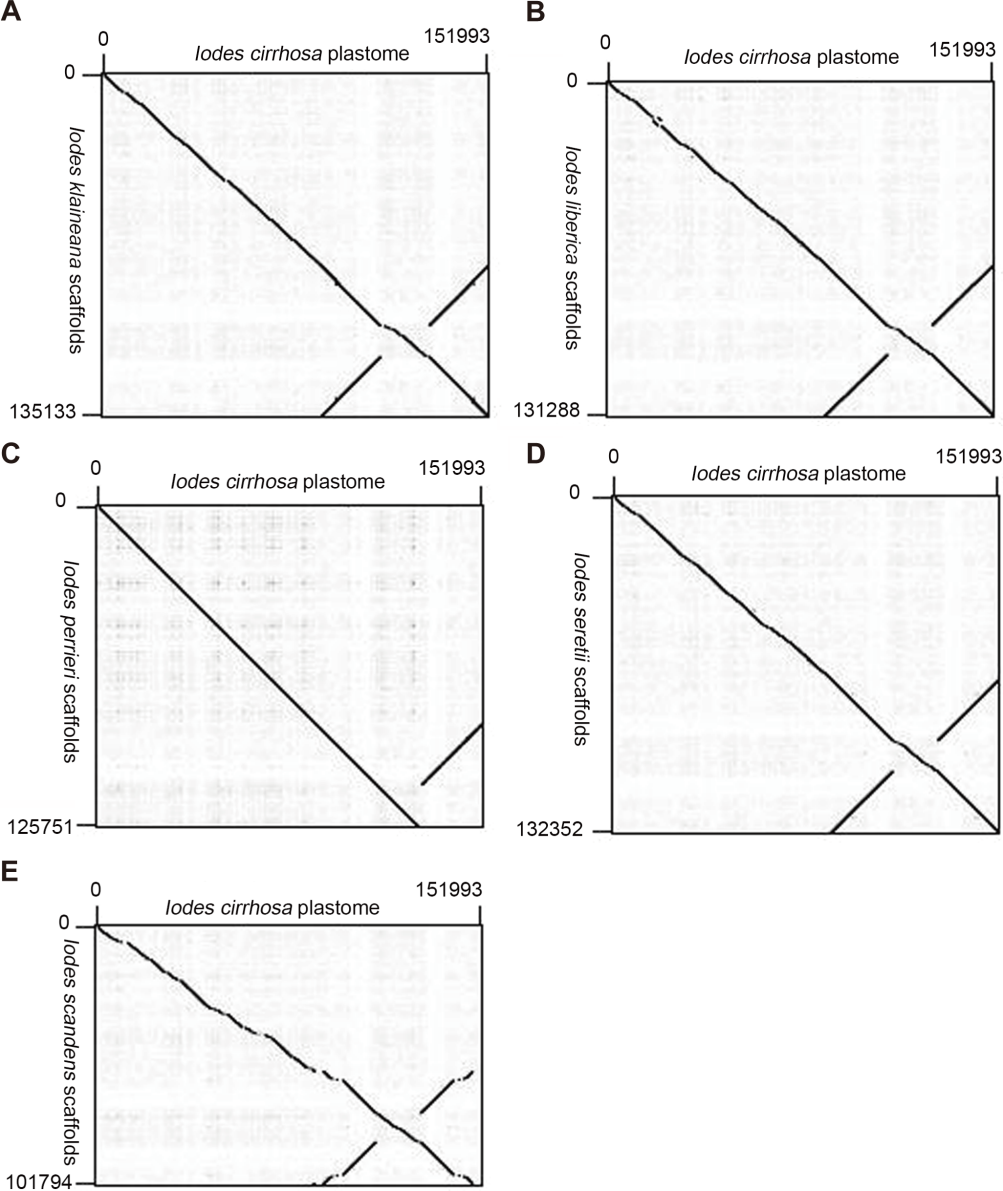
Dotplots of the plastome from *Idoes cirrhosa* and scaffolds from (A) *Iodes klaineana*, (B) *Iodes liberica*, (C) *Iodes perrieri*, (D) *Iodes seretii*, (E) *Iodes scandens*. The horizontal axis represents the *I. cirrhosa* plastome. The vertical axis represents the scaffolds of the other five *Iodes* species.

We reassembled the plastome sequences of *I. klaineana* (Dataset S2), *I. liberica* (Dataset S3), *I. perrieri* (Dataset S4), *I. scandens* (Dataset S5), and *I. seretii* (Dataset S6) by using the complete plastome sequences of *I. cirrhosa* as the reference. The reconstructed plastomes significantly improved as shown in the dotplots, showing the alignment between the plastome of *I. cirrhosa* and those of the five other five *Iodes* species (Fig. 5). However, we failed to fill the gaps in these plastomes experimentally because we cannot obtain the materials for these five species. Overall, our reference guided the reconstruction of the plastid proteomes and plastomes, thereby resulting in considerable improvement compared with those obtained before (see below). Thus, using closely related reference plastome in the assembly of new plastome sequences is important.

We identified the proteomes for each of the five *Iodes* species by using the plastid proteome of the *I. cirrhosa* reference. The multiple sequence alignments of proteins from the six *Iodes* species and *A. thaliana* are shown with highlights in Figs. S1–S74 and in FASTA format in Dataset S7. The quality of the assembly results of most homologous proteins have been improved significantly, although some parts of the protein sequences may still be missing. To demonstrate how much our reference guided reassembly has improved the proteome, we aligned the MATK protein from this study and that reported from the previous study for each of the five *Iodes* species in Figs. S75–S80. The regions that were significantly improved are shown in red square.

### Phylogenetics analysis

Then, we rebuilt the phylogenetic tree on the basis of the reconstructed protein sequence as described above, with *A*. *thaliana* and *N. tabacum* as the outgroups, respectively. The ML tree constructed with 74 concatenated proteins is shown in Fig. S81. The topological structure of the *Iodes* genus branch was the same as those described previously by Stull et al. (2015). The alignment of all the 74 proteins concatenated to construct the phylogenetic tree is shown in NEXUS format in Dataset S8. The corresponding alignments of the nucleotide sequences for each gene were shown with highlights in Figs. S82–S155 and in FASTA format in Dataset S9. The *pet*G, *psb*Z, *rps*7, *rps*12, *ycf*1, and *ycf*3 genes were used in the phylogenetics analysis in the present study. However, these genes were excluded from the phylogenetics analysis due to poor sequence quality in the study of Stull et al. (2015). The alignments of these six genes are shown in Figs. S107, S128, S144, S146, S152, and S154. In summary, using the reconstructed proteome, the phylogenetic relationships of the six *Iodes* species have been resolved completely.

### Identification of positively selected genes and sites

Synonymous substitutions were accumulated nearly neutrally, while nonsynonymous substitutions were subjected to selective pressure of varying degrees and directions (positive or negative). In general, the ratio of nonsynonymous to synonymous substitution (*ω*) measures the levels of selective pressure operating in a protein-coding gene (Jiang et al., 2018). To test which shared genes were subjected to positive selection, we conducted the selection analysis of the exons of each protein-coding gene by using the aBSREL model. A total of 11 branches among the six *Iodes* species were tested to diversify the selection.

A total of 27 shared genes evolved under positive selection in the *Iodes* genus branch. The significance and number of rate categories inferred at the *Iodes* genus branch are provided in Table 3. The 27 genes can be classified into 8 groups. The first group contained two ATP synthase genes, namely, *atp*B and *atp*F. The second group contained seven NADH-dehydrogenase genes, namely, *ndh*A, *ndh*B, *ndh*E, *ndh*F, *ndh*F, *ndh*G, and *ndh*K. The third group contained one gene, that is, *pet*G, which codes a subunit of the cytochrome b/f complex. The fourth group contained *psa*A, which is a gene involved in photosynthesis. The fifth group contained seven ribosomal protein-coding genes, namely, *rpl*2, *rpl*22, *rpl*23, *rps*3, *rps*7, *rps*18, and *rps*19. The sixth group contained three RNA polymerase genes, namely, *rpo*B, *rpo* C1, and *rpo*C2. The seventh group contained *acc* D, *ccs*A, and *mat*K, which were classified into other protein groups. The eighth group contained *ycf*1, *ycf*2, and *ycf*3, which have unknown functions.

To determine which sites were subjected to positive selection along the *Iodes* genus branch, we used the codeml from PAML (v. 4.9; Yang & Nielsen, 2002) to analyze the 27 genes that were under positive selection by using the branch-site model. The *Iodes* genus branch shown in the ML tree was set as the foreground branch, and the outgroup branches were set as the background branches. A total of 52 amino acid residue sites from 11 genes were positively selected (Table 4). The gene names and the numbers of selected sites were *ccs*A (4), *mat*K (5), *ndh*E (4), *ndh*F (3), *ndh*K (1), *psa*A (25), *rpl*23 (2), *rpo*C1 (2), *rps*18 (2), *rps*7 (2), and *ycf*2 (2). A total of 25 amino acid residue sites in the gene *psa*A were positively selected. *psa*A is a gene that is related to photosynthesis. The biological relevance of these selected sites will be an interesting topic for future study.

### Divergence-time analysis of the *Iodes* genus

**Figure 6:**
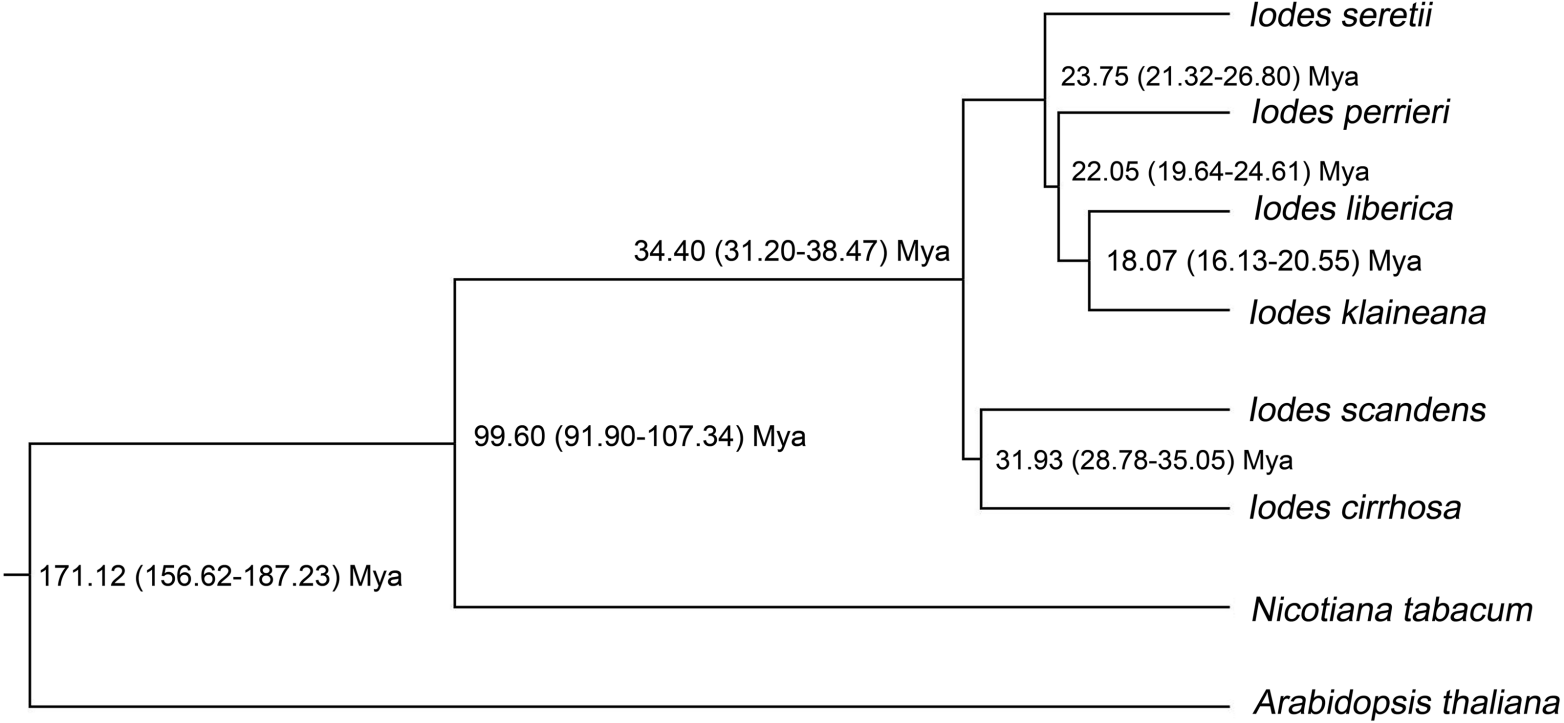
Divergence time of the six *Iodes* species estimated using the BEAST software. Numbers beyond the node represent *Iodes* species divergence time (million years ago, Mya) and 95% highest posterior density intervals. The topology of the *Iodes* branch based on 74 protein-coding genes is completely consistent with ML tree.

To identify the divergence time of the six *Iodes* species, we utilized the ML tree (Dataset S10) and alignment of the 74 shared proteins (Dataset S8) as the original files for the Bayesian evolutionary analysis by using sampling trees. The tree showed that the six *Iodes* species formed a monophyletic group. The topology of the branch was identical to that in the ML tree. The divergence time of each node in the monophyly of *Iodes* genus was annotated beyond each node (Fig. 6). The divergence time of the *Iodes* monophyly was estimated to be 34.40 Mya. This time period was later than the 54 Mya of Icacinaceae appearance according to the TreeTime report (Hedges, Dudley & Kumar, 2006). The divergence time between *I. cirrhosa* and *I. scandens* was approximately 31.93 Mya, which was approximately during the early Oligocene epoch. However, the four other *Iodes* species began to diverge until approximately 23.75 Mya, thereby suggesting that these species were the young population in *Iodes* (Fig. 6). *I. klaineana* and *I. liberica* were the youngest species, which split at approximately 18.07 Mya (Fig. 6). The split time of the six *Iodes* species was estimated to be from the late Oligocene epoch to the early Miocene epoch.

**Table 3:** The list of genes under positive selection in *Iodes* genus.

**Species under selection**	**B**	**LRT**	**Test *p*-value**	**Uncorrected *p*-value**	*ω* Distribution over Sites	**Gene name**
*I. scandens*	0.0395	69.1914	0	0	*ω*1 = 0.219 (97%) *ω*2 = 10,000 (2.8%)	*acc* D
*I. liberica*	0.0161	13.5704	0.0046	0.0004	*ω*1 = 0.00 (100%) *ω*2 = 10,000 (0.21%)	
*I. seretii*	0.0177	20.9163	0.0001	0	*ω*1 = 0.00 (100%) *ω*2 = 10,000 (0.43%)	*atp* B
*I. scandens*	0.0395	43.2439	0	0	*ω*1 = 0.0396 (79%) *ω*2 = 10,000 (21%)	*atp* F
*I. seretii* *I. perrieri* *I. liberica* *I. klaineana*	0.0033	41.7172	0	0	*ω*1 = 0.00 (98%) *ω*2 = 10,000 (2.3%)	
*I. liberica*	0.0161	41.5182	0	0	*ω*1 = 0.00 (99%) *ω*2 = 10,000 (0.78%)	*ccs* A
*I. perrieri*	0.0131	21.6819	0.0001	0	*ω*1 = 0.237 (99%) *ω*2 = 10,000 (0.81%)	
*I. liberica*	0.0161	31.5631	0	0	*ω*1 = 0.00 (91%) *ω*2 = 10,000 (9.2%)	*mat* K
*I. cirrhosa* *I. scandens*	0.0034	232.27	0	0	*ω*1 = 1.00 (72%) *ω*2 = 10,000 (28%)	
*I. cirrhosa*	0.023	15.8778	0.0013	0.0001	*ω*1 = 1.00 (95%) *ω*2 = 10,000 (5.1%)	
*I. cirrhosa*	0.023	151.9285	0	0	*ω*1 = 0.0229 (92%) *ω*2 = 2,240 (8.0%)	*ndh* A
*I. scandens*	0.0395	138.8669	0	0	*ω*1 = 0.465 (90%) *ω*2 = 2,240 (10%)	
*I. seretii*	0.0177	125.9252	0	0	*ω*1 = 0.555 (92%) *ω*2 = 10,000 (8.2%)	
*I. perrieri*	0.0131	118.0196	0	0	*ω*1 = 0.0245 (95%) *ω*2 = 3,330 (5.0%)	
*I. perrieri* *I. liberica* *I. klaineana*	0.0006	151.8593	0	0	*ω*1 = 0.00 (92%) *ω*2 = 3,330 (8.4%)	
*I. klaineana*	0.0074	18.3085	0.0003	0	*ω*1 = 0.271 (100%) *ω*2 = 10,000 (0.35%)	
*I. scandens*	0.0395	40.0987	0	0	*ω*1 = 0.434 (99%) *ω*2 = 10,000 (0.52%)	*ndh* B
*I. seretii*	0.0177	38.5771	0	0	*ω*1 = 0.00 (71%) *ω*2 = 1,850 (29%)	*ndh* E
*I. liberica*	0.0161	32.0969	0	0	*ω*1 = 0.176 (99%) *ω*2 = 10,000 (1.3%)	*ndh* F
*I. perrieri*	0.0131	63.2828	0	0	*ω*1 = 0.264 (97%) *ω*2 = 377 (2.6%)	*ndh* F
*I. scandens*	0.0395	90.4163	0	0	*ω*1 = 0.241 (96%) *ω*2 = 324 (4.1%)	
*I. seretii*	0.0177	13.5496	0.0039	0.0004	*ω*1 = 0.320 (99%) *ω*2 = 10,000 (0.82%)	
*I. liberica* *I. klaineana*	0.0022	13.3158	0.0039	0.0004	*ω*1 = 0.375 (100%) *ω*2 = 10,000 (0.24%)	
*I. scandens*	0.0395	25.8917	0	0	*ω*1 = 0.749 (80%) *ω*2 = 100 (20%)	*ndh* G
*I. cirrhosa* *I. scandens*	0.0034	29.2692	0	0	*ω*1 = 1.00 (99%) *ω*2 = 10,000 (1.2%)	*ndh* K
*I. perrieri*	0.0131	17.129	0.0008	0.0001	*ω*1 = 0.00 (93%) *ω*2 = 839 (6.7%)	*pet* G
*I. perrieri*	0.0131	362.9645	0	0	*ω*1 = 0.0306 (94%) *ω*2 = 10,000 (6.4%)	*psa* A
*I. seretii*	0.0177	332.4549	0	0	*ω*1 = 0.143 (95%) *ω*2 = 1,550 (4.9%)	
*I. seretii* *I. perrieri* *I. liberica* *I. klaineana*	0.0033	158.1644	0	0	*ω*1 = 0.188 (90%) *ω*2 = 3,640 (10%)	
*I. perrieri*	0.0131	44.4643	0.0000	0.0000	*ω*1 = 0.0577 (72%) *ω*_2_= 2000 (28%)	*rpl* 2
*I. liberica*	0.0161	10.1996	0.0253	0.0021	*ω*_1_= 1.00 (99%) *ω*_2_= 10,000 (1.5%)	*rpl* 22
*I. scandens*	0.0395	15.2617	0.0021	0.0002	*ω*_1_= 0.195 (97%) *ω*_2_= 110 (3.3%)	*rpl* 23
*I. scandens*	0.0395	19.7602	0.0002	0.0000	*ω*_1_= 0.173 (100%) *ω*_2_= 255 (0.32%)	*rpo* B
*I. perrieri*	0.0131	116.6650	0.0000	0.0000	*ω*_1_= 0.0980 (97%) *ω*_2_= 3,330 (2.5%)	*rpo* C1
*I. scandens*	0.0395	44.2547	0.0000	0.0000	*ω*_1_= 1.00 (99%) *ω*_2_= 10,000 (0.94%)	
*I. seretii*	0.0177	72.9439	0.0000	0.0000	*ω*_1_= 0.304 (99%) *ω*_2_= 10,000 (0.86%)	*rpo* C2
*I. scandens*	0.0395	98.4880	0.0000	0.0000	*ω*_1_= 0.336 (98%) *ω*_2_= 10,000 (2.3%)	
*I. liberica*	0.0161	28.3779	0.0000	0.0000	*ω*_1_= 0.540 (100%) *ω*_2_= 10,000 (0.47%)	
*I. liberica* *I. klaineana*	0.0022	26.4708	0.0000	0.0000	*ω*_1_= 0.777 (100%) *ω*_2_= 5,480 (0.38%)	
*I. perrieri*	0.0131	14.7652	0.0017	0.0002	*ω*_1_= 0.402 (100%) *ω*_2_= 443 (0.18%)	
*I. perrieri* *I. liberica* *I. klaineana*	0.0006	13.1292	0.0063	0.0005	*ω*_1_= 0.00 (99%) *ω*_2_= 10,000 (0.51%)	*rps* 3
*I. seretii* *I. perrieri* *I. liberica* *I. klaineana*	0.0033	9.9293	0.0290	0.0024	*ω*_1_= 0.00 (99%) *ω*_2_= 10,000 (1.1%)	
*I. perrieri*	0.0131	75.2232	0.0000	0.0000	*ω*_1_= 0.00 (79%) *ω*_2_= 320 (21%)	*rps* 7
*I. scandens*	0.0395	12.6120	0.0081	0.0006	*ω*_1_= 0.00 (97%) *ω*_2_= 10,000 (3.4%)	*rps* 18
*I. liberica*	0.0161	69.4590	0.0000	0.0000	*ω*_1_= 1.00 (83%) *ω*_2_= 3,330 (17%)	*rps* 19
*I. cirrhosa*	0.0230	25.6408	0.0000	0.0000	*ω*_1_= 1.00 (99%) *ω*_2_= 10,000 (0.66%)	*ycf* 1
*I. perrieri*	0.0131	22.7892	0.0000	0.0000	*ω*_1_= 0.00 (100%) *ω*_2_= 10,000 (0.30%)	
*I. scandens*	0.0395	194.6220	0.0000	0.0000	*ω*_1_= 0.286 (98%) *ω*_2_= 10,000 (2.0%)	*ycf* 2
*I. liberica*	0.0161	134.1704	0.0000	0.0000	*ω*_1_= 0.0920 (99%) *ω*_2_= 6,070 (1.1%)	
*I. liberica* *I. klaineana*	0.0022	51.3748	0.0000	0.0000	*ω*_1_= 0.399 (100%) *ω*_2_= 3,330 (0.36%)	
*I. liberica*	0.0161	40.5254	0.0000	0.0000	*ω*_1_= 0.367 (95%) *ω*_2_= 10,000 (4.9%)	*ycf* 3

**Notes.**

B Optimized branch length LRT Likelihood ratio test statistic for selection Test *p*-value *p*-value corrected for multiple testing Uncorrected *p*-value Raw *p*-value without correction for multiple testing *ω* The ratio of nonsynonymous to synonymous substitution

**Table 4:** The list of amino acid residues under positive selection in the *Iodes* branch.

**Gene**	**Positive selected position**	**Amino acid**	**Omega value**
*ccs*A	50	T	0.587
	52	F	0.505
	131	A	0.502
	242	R	0.647
*mat*K	186	R	0.815
	434	G	0.771
	495	S	0.587
	568	V	0.672
	639	R	0.868
*ndh*E	5	Y	0.660
	34	F	0.669
	68	G	0.559
	78	S	0.525
*ndh*F	503	M	0.685
	512	F	0.642
	723	I	0.639
*ndh*K	224	R	0.994
*psa*A	18	H	0.717
	28	R	0.719
	652	A	0.702
	680	P	0.896
	771	H	0.946
	856	F	0.985
	859	R	0.580
	860	D	0.984
	861	Y	0.979
	862	D	0.569
	871	L	0.934
	873	R	0.979
	875	L	0.756
	877	H	0.993
	879	D	0.608
	880	A	0.884
	881	I	0.902
	928	A	0.943
	929	Q	0.904
	931	I	0.971
	932	Q	0.972
	987	V	0.880
	988	H	0.992
	989	H	0.989
	993	F	0.568
*rpl*23	33	I	0.629
	37	R	0.623
*rpo*C1	102	T	0.657
	693	F	0.712
*rps*18	74	S	0.621
	95	I	0.698
*rps*7	12	A	0.714
	69	V	0.709
*ycf*2	1,478	S	0.894
	2,127	V	0.994

**Notes.**

Omega value = 1, >1, and <1, indicates neutral evolution, purifying selection, and positive selection, respectively.

## Discussion

In this study, we sequenced and analyzed the plastome of *I. cirrhosa*, which was the first complete plastome sequence in Icacinaceae. In particular, we (1) extracted total DNA from the leaves of *I. cirrhosa*; (2) filtered the sequence reads on the basis of sequence similarity to the plastome sequences of the other closely related species; (3) assembled the sequence reads followed by gap-filling to obtain the complete plastome; (4) annotated the genome to identify protein-coding, rRNA, and tRNA genes; (5) calculated the bias of codon usage; (6) analyzed the long repeat and SSR sequences; (7) characterized the boundary of the IR regions; (8) reconstructed proteomes and plastomes of the five *Iodes* species by using the plastome sequence of *I. cirrhosa* as reference; (9) further confirmed the phylogenetic relationships among the six *Iodes* species; and (10) identified the positively selected genes and sites in the plastomes on the basis of this phylogeny and resolved the evolutionary history of these six *Iodes* species.

In a previous study, plastome-scale dataset has been used to resolve the basal lamiid phylogeny (Stull et al., 2015). As described before, complete plastome sequences are needed for many applications. Hence, we downloaded the plastome-scale dataset for six *Iodes* species reported in the study of Stull et al. and assembled them following the standard procedures described above. To our surprise, all six assembly results were highly fragmental and contained numerous gaps. Failure to obtain high-quality assemblies may be due to the following reasons. First, the samples in Stull et al.’s (2015) study may be of relatively poor quality. The extracted DNAs had been degraded extensively and lost some regions completely. Second, the Icacinaceae genomes may be highly heterogeneous. Third, the plastomes may be highly fragmented. Unfortunately, we failed to collect samples of the species reported previously and cannot test the hypotheses above. Instead, we collected the samples of *I. cirrhosa* and successfully obtained the first complete plastome sequence in Icacinaceae.

From the complete plastome sequences, many SSR markers were identified. These SSRs are potentially useful in studying the evolutionary history of *I. cirrhosa* populations (Chae et al., 2014; Khadivi-Khub et al., 2014). SSRs play an important role in genomic rearrangement and sequence variation in plastomes (Huang et al., 2014). The dominance of A/T in mononucleotide SSRs in *I. cirrhosa* was similar to those described previously (Yao et al., 2016). Approximately 83% of the repeats were found in the intergenic regions, which were often also divergent hotspot regions (Huang et al., 2014; Yao et al., 2015). These regions would be useful for the development of new phylogenetic markers for species identification and discrimination.

Regarding the gene contents, four pairs of overlapping genes were identified in the *I. cirrhosa* plastome. The presence of overlapping genes in plastomes generally indicates the low evolutionary degree of species and is a common feature of bacterial genomes. Overlapping genes are often transcriptionally and translationally coregulated and have been found in other plastomes. In tobacco plastome, four pairs of overlapping genes, namely, *psb*D/*psb*C, *ndh*C/*ndh*K, *atp*B/*atp*E, and *rpl*22/*rps*3, have been found (Shinozaki et al., 1986). Compared with those in the tobacco genome, the overlapping gene pair of *ndh*C/*ndh*K in the tobacco plastome was replaced by the pair of *ndh*F/Ψ*ycf*1 in the *I. cirrhosa* plastome. The overlapping gene pair *ndh*F/Ψ*ycf*1 was also found before in the *I. paraguariensis* plastome and in some Asteraceae species (Curci et al., 2015).

For other gene pairs, the 50-nucleotide overlapping regions of the *psb*D and *psb*C genes were first identified in the plastome of spinach. The in vitro translation of the *psb*C cistron is dependent upon the upstream *psb* D cistron (Adachi et al., 2012). However, not all overlapping genes are coregulated, transcribed, and translated. For example, the overlapping genes *atp*B and *atp*E have a stop codon (UGA) for *atp*B and the start codon (AUG) for *atp*E. Therefore, the translation of *atp*B and *atp*E is considered independent (Suzuki et al., 2011). Whether or not these overlapping genes represent their ancestral states and are kept for particular reason will be interesting topics for future studies.

In the selection pressure analysis, eight groups of genes were under positive selection pressure. The largest groups of genes encoded NADH-dehydrogenases and ribosomal proteins, each of which had seven members. The chloroplast NADH dehydrogenase-like (NDH) complex is involved in the cyclic electron transport around photosystem I and chlororespiration. The NDH complex was discovered more than 20 years ago. However, analyzing the function of this complex has been difficult because of low abundance and fragile nature. The NDH subunit is involved in the stabilization of the NDH complex, especially under high light conditions (Fan et al., 2015). Considering that the *Iodes* species is largely distributed in the tropical and subtropical areas, which are generally under high light and temperature conditions, the selection of these NDH genes will likely increase the stability of the complex, thereby allowing the effective functioning of the chloroplast in these environments.

Similarly, the ribosomal proteins are part of the ribosome complex, which is the translational machinery that is critical for the correct production of proteins required for the normal functioning of a cell. The selection of the ribosomal proteins may increase the stability of ribosomal complex under the conditions frequently associated with high light, such as high temperature, which is similar to the selection of the NDH proteins under high light. However, whether or not these ribosomal proteins have better stability under high light or associated conditions than those ancestral proteins still require further validation experimentally.

The *ycf*1, *ycf*2, and *ycf*3 genes were also positively selected. The functions of these three genes are largely unknown at present. These genes are known to evolve rapidly. The question on whether the rapid evolution rates of these genes and these selected sites are related remain to be elucidated. Sequence similarity search against GenBank showed that this protein is similar to photosystem I assembly protein *ycf*3 from *Anethum graveolens* (EU016788) and *Lomandra lonqifolia* (HQ183650). Consistent with the proposal above, the positive selection of the YCF3 gene may reflect the adaptation of these plants to the high-light environmental condition.

As shown in the divergence-time analysis, *Iodes* species were first formed on the last Eocene epoch. The time was later than the early-middle Eocene epoch of Icacinaceae fossils (Stull, Moore & Manchester, 2011), which indicated the varied forming times of Icacinaceae species. The older group of *I. cirrhosa* and *I. scandens* split on the last Eocene epoch. From the middle Eocene epoch, the climate have become cold, and the continental interiors have begun to dry out. The formation of the *Iodes* genus may be affected by the cooling environment. The divergence times of the six *Iodes* species in this present study were distributed in the Oligocene and the early Miocene. The climate was slow global cooling during the Oligocene and Oligocene-Miocene transition, although it remained stable. The cooling climate may have driven the evolution of these *Iodes* species. However, the six *Iodes* species formed two branches in the *Iodes* monophyly, which may be attributed by considerably complex reasons.

The current study failed to identify genes that are related to the therapeutic effects of *I. cirrhosa*. Given that chloroplast is mostly involved in the photosynthesis process, it is not directly related to the biosynthesis of bioactive compounds. In the future, the analysis of nuclear genes will likely identify the genes that are related to the production of their activity. Lastly, while a plastome-scale dataset is generally sufficient to resolve a phylogenetic relationship, complete plastome sequences are required to understand the evolutionary processes at the genome scale and identify markers from noncoding regions. The results obtained from this study have set the stage to understand how plastomes from Icacinaceae evolve.

## Conclusions

The complete plastome of *I. cirrhosa*, which is the first sequenced member of the Icacinaceae family, was determined and characterized in detail. Using the plastome of *I. cirrhosa* as a reference, we reconstructed the proteomes and plastomes of the five *Iodes* species. We identified 27 genes and 52 amino acid sites that had undergone positive selection. The divergence time of the six *Iodes* species was 34.40 Mya.

##  Supplemental Information

10.7717/peerj.6663/supp-1 Supplemental Information 1 Supplemental Tables

10.7717/peerj.6663/supp-2 Supplemental Information 2 Supplemental Figures

10.7717/peerj.6663/supp-3 Dataset S1 *Iodes cirrhosa* chloroplast complete genome

10.7717/peerj.6663/supp-4 Dataset S2 *Iodes klaineana* plastome scaffolds

10.7717/peerj.6663/supp-5 Dataset S3 *Iodes liberica* plastome scaffolds

10.7717/peerj.6663/supp-6 Dataset S4 *Iodes perrieri* plastome scaffold

10.7717/peerj.6663/supp-7 Dataset S5 *Iodes scandens* plastome scaffolds

10.7717/peerj.6663/supp-8 Dataset S6 *Iodes seretii* plastome scaffolds

10.7717/peerj.6663/supp-9 Dataset S7 Multiple alignment of amino acid sequences in fasta format

10.7717/peerj.6663/supp-10 Dataset S8 Alignment of 74 shared proteins

10.7717/peerj.6663/supp-11 Dataset S9 Multiple alignment of nucleotide sequences in fasta format

10.7717/peerj.6663/supp-12 Dataset S10 ML tree as the user-specified starting tree

## Additional Information and Declarations

### DNA Deposition

The following information was supplied regarding the deposition of DNA sequences: The plastome sequences of *Iodes cirrhosa* is available via GenBank: NC_036304.

### Data Availability

The following information was supplied regarding data availability: The raw data is available in GenBank: SRR8503885.
