# Supplementary material for: Complete plastome sequence of Iodes cirrhosa Turcz., the first in the Icacinaceae, comparative genomic analyses and possible split of Idoes species in response to climate changes
Source: PeerJ. 2019 Apr 1;7:e6663. doi: 10.7717/peerj.6663 (PMC6448556; doi:10.7717/peerj.6663)
Supplement: Supplemental Information 1 [file peerj-07-6663-s001.docx]

**Supplemental Tables**

**Complete plastome sequence of *Iodes cirrhosa* Turcz., the first in the Icacinaceae, comparative genomic analyses and possible split of *Iodes* species in response to climate changes**

***Liqiang Wang*** ***^¶^, Hui Zhang*** ***^¶^, Mei Jiang, Haimei Chen, Linfang Huang*, Chang Liu****

Key Laboratory of Bioactive Substances and Resource Utilization of Chinese Herbal Medicine from Ministry of Education, Institute of Medicinal Plant Development, Chinese Academy of Medical Sciences and Peking Union Medical College, Beijing, P. R. China.

E-mails:

[lys832000@163.com](mailto:lys832000@163.com) (LQW);

[18238801021@163.com](mailto:18238801021@163.com) (HZ);

[mjiang0502@163.com](mailto:mjiang0502@163.com) (MJ);

[hmchen@implad.ac.cn](mailto:hmchen@implad.ac.cn) (HMC);

15801545922@139.com (LFH);

[cliu6688@yahoo.com](mailto:cliu6688@yahoo.com) (CL)

**^¶^**These authors contributed equally to this work.

*****Correspondence:

Professors Chang Liu, Linfang Huang

cliu6688@yahoo.com; Tel.: +86-010-5783-3111; Fax: +86-10-62899715

15801545922@139.com; Tel: +86-010-5783-3197; Fax: +86-10-62899715

**Table S1 PCR primers used in this study.**

| **Primer ID** | **Sequence (5’-3’)** |
| --- | --- |
| 1-F | GTCCAGATTAAGGCCCTGCT |
| 1-R | TTTGGTCCCGCTATTCGGAG |
| 2-F | CTCCACGCTTTCTTTCCTTTG |
| 2-R | ATTGGGTCCTCTCGGATCTA |
| 3-F | GCTCTAGCACTGCTTCCTAAG |
| 3-R | CCGTCGCTCAACGGATAAA |
| 4-F | AACCCTGTAGACCATCCCCA |
| 4-R | GTAAGGCCCCCGTCTTAGTG |
| 5-F | TCGTAAGAACGCCCACGAAT |
| 5-R | AACCCTGTAGACCATCCCCA |
| 6-F | CCGTCGCTCAACGGATAAA |
| 6-F | TACGCCTAGGACACCAGAATA |

**Note:** F and R represent “Forward” and “Reverse,” respectively.

**Table S2 The lengths of introns and exons for genes having introns.**

| **Gene** | **Location** | **Length (bp)** | | | | |
| --- | --- | --- | --- | --- | --- | --- |
|  |  | **Exon I** | **Intron I** | **Exon II** | **Intron II** | **Exon III** |
| *rps*16 | LSC | 45 | 870 | 222 |  |  |
| *atp*F | LSC | 411 | 704 | 144 |  |  |
| *rpo*C1 | LSC | 435 | 763 | 1617 |  |  |
| *ycf*3 | LSC | 127 | 727 | 230 | 748 | 153 |
| *clp*P | LSC | 69 | 840 | 291 | 668 | 228 |
| *pet*B | LSC | 6 | 48 | 639 |  |  |
| *pet*D | LSC | 6 | 47 | 474 |  |  |
| *rpl*16 | LSC | 399 | 1039 | 8 |  |  |
| *rpl*2 (×2) | IR | 390 | 660 | 441 |  |  |
| *ndh*B (×2) | IR | 777 | 687 | 756 |  |  |
| *ndh*A | SSC | 559 | 986 | 539 |  |  |
| *trn*A-UGC (×2) | IR | 38 | 791 | 37 |  |  |
| *trn*C-ACA | LSC | 40 | 553 | 57 |  |  |
| *trn*E-UUC (×2) | IR | 33 | 943 | 41 |  |  |
| *trn*K-UUU | LSC | 38 | 2539 | 36 |  |  |
| *trn*L-UAA | LSC | 36 | 480 | 51 |  |  |
| *trn*T-CGU | LSC | 35 | 676 | 44 |  |  |

**Note:** Numbers in the parentheses represent the number of copies. LSC: Large Single Copy Region; SSC: Small Single Copy Region; IR: Inverted Repeat Region; CDS: Coding Sequence.

**Table S3 Base compositions for different regions of *I. cirrhosa* plastome.**

| **Type** | **Codon Position** | **T(U) (%)** | **C (%)** | **A (%)** | **G (%)** | **Length (bp)** |
| --- | --- | --- | --- | --- | --- | --- |
| LSC |  | 32.96 | 18.26 | 31.59 | 17.19 | 84527 |
| SSC |  | 34.63 | 14.77 | 34.17 | 16.43 | 17522 |
| IRA |  | 28.46 | 20.73 | 28.54 | 22.27 | 24973 |
| IRB |  | 28.54 | 22.26 | 28.46 | 20.73 | 24972 |
| Total |  | 31.69 | 18.92 | 30.87 | 18.52 | 151994 |
| CDS |  | 31.73 | 17.79 | 30.18 | 20.30 | 73017 |
|  | 1^st^ | 25.17 | 18.39 | 30.13 | 26.30 | 24342 |
|  | 2^nd^ | 32.73 | 20.61 | 28.02 | 18.62 | 24342 |
|  | 3^rd^ | 37.28 | 14.35 | 32.37 | 15.98 | 24342 |

**Note:** LSC: large single-copy region, SSC: small single-copy region, IR: inverted repeat region, CDS: coding sequence.

**Table S4 Codon usage in the plastome of *I. cirrhosa* plastome.**

| **Codon** | **Amino Acid coded** | **Number of Codons** | **RSCU** |
| --- | --- | --- | --- |
| UUU | F | 879 | 1.28 |
| UUC | F | 498 | 0.72 |
| UUA | L | 808 | 1.84 |
| UUG | L | 542 | 1.23 |
| CUU | L | 573 | 1.3 |
| CUC | L | 181 | 0.41 |
| CUA | L | 370 | 0.84 |
| CUG | L | 167 | 0.38 |
| AUU | I | 1016 | 1.46 |
| AUC | I | 409 | 0.59 |
| AUA | I | 663 | 0.95 |
| AUG | M | 595 | 1 |
| GUU | V | 510 | 1.48 |
| GUC | V | 168 | 0.49 |
| GUA | V | 509 | 1.48 |
| GUG | V | 190 | 0.55 |
| UCU | S | 562 | 1.74 |
| UCC | S | 315 | 0.97 |
| UCA | S | 378 | 1.17 |
| UCG | S | 174 | 0.54 |
| CCU | P | 390 | 1.52 |
| CCC | P | 199 | 0.78 |
| CCA | P | 305 | 1.19 |
| CCG | P | 133 | 0.52 |
| ACU | T | 527 | 1.65 |
| ACC | T | 220 | 0.69 |
| ACA | T | 390 | 1.22 |
| ACG | T | 139 | 0.44 |
| GCU | A | 600 | 1.8 |
| GCC | A | 228 | 0.69 |
| GCA | A | 365 | 1.1 |
| GCG | A | 137 | 0.41 |
| UAU | Y | 746 | 1.62 |
| UAC | Y | 173 | 0.38 |
| UAA | * | 49 | 1.65 |
| UAG | * | 20 | 0.67 |
| CAU | H | 449 | 1.53 |
| CAC | H | 139 | 0.47 |
| CAA | Q | 645 | 1.51 |
| CAG | Q | 212 | 0.49 |
| AAU | N | 879 | 1.53 |
| AAC | N | 271 | 0.47 |
| AAA | K | 889 | 1.49 |
| AAG | K | 304 | 0.51 |
| GAU | D | 813 | 1.6 |
| GAC | D | 204 | 0.4 |
| GAA | E | 935 | 1.5 |
| GAG | E | 314 | 0.5 |
| UGU | C | 210 | 1.48 |
| UGC | C | 74 | 0.52 |
| UGA | * | 20 | 0.67 |
| UGG | W | 408 | 1 |
| CGU | R | 325 | 1.32 |
| CGC | R | 95 | 0.39 |
| CGA | R | 359 | 1.46 |
| CGG | R | 114 | 0.46 |
| AGU | S | 401 | 1.24 |
| AGC | S | 113 | 0.35 |
| AGA | R | 434 | 1.76 |
| AGG | R | 153 | 0.62 |
| GGU | G | 557 | 1.28 |
| GGC | G | 181 | 0.42 |
| GGA | G | 709 | 1.63 |
| GGG | G | 294 | 0.68 |

RSCU: Relative synonymous codon usage.

**Table S5 Codon frequencies in the plastome of *I. cirrhosa* plastome.**

| **Coded Amino Acids** | **Number of Codon Types** | **Total Numbers of Codons** | **Percentage** |
| --- | --- | --- | --- |
| * | 3 | 89 | 0.36% |
| C | 2 | 284 | 1.15% |
| W | 1 | 408 | 1.66% |
| H | 2 | 588 | 2.39% |
| M | 1 | 595 | 2.42% |
| Q | 2 | 857 | 3.48% |
| Y | 2 | 919 | 3.73% |
| D | 2 | 1017 | 4.13% |
| P | 4 | 1027 | 4.17% |
| N | 2 | 1150 | 4.67% |
| K | 2 | 1193 | 4.84% |
| E | 2 | 1249 | 5.07% |
| T | 4 | 1276 | 5.18% |
| A | 4 | 1330 | 5.40% |
| F | 2 | 1377 | 5.59% |
| V | 4 | 1377 | 5.59% |
| R | 6 | 1480 | 6.01% |
| G | 4 | 1741 | 7.07% |
| S | 6 | 1943 | 7.89% |
| I | 3 | 2088 | 8.48% |
| L | 6 | 2641 | 10.72% |

**Table S6 Repeats identified in the *I. cirrhosa* plastome using the program REPuter. P: Palindromic; F: Forward; R: Reverse; C: Complementary: IGS: Intergenic spacer regions.**

| **Size** | **Start Position** | **Region** | **Type** | **Size** | **Start Position** | **Region** | **P Value** |
| --- | --- | --- | --- | --- | --- | --- | --- |
| 30 | 8258 | IGS | F | 30 | 35955 | IGS | 6.18E-04 |
| 30 | 8258 | IGS | P | 30 | 45888 | IGS | 5.07E-07 |
| 31 | 9605 | IGS | F | 31 | 36929 | IGS | 1.71E-04 |
| 30 | 9648 | IGS | R | 30 | 9648 | IGS | 5.64E-09 |
| 30 | 15336 | IGS | C | 30 | 47040 | IGS | 6.18E-04 |
| 32 | 28449 | IGS | P | 32 | 28449 | IGS | 1.57E-06 |
| 38 | 31895 | IGS | F | 38 | 31913 | IGS | 9.80E-12 |
| 35 | 32613 | IGS | P | 35 | 32613 | IGS | 5.78E-10 |
| 30 | 39090 | psaB | F | 30 | 41314 | psaA | 2.21E-05 |
| 49 | 57908 | accD | F | 49 | 57932 | accD | 2.17E-16 |
| 31 | 57926 | accD | F | 31 | 57950 | accD | 1.31E-07 |
| 30 | 59686 | IGS | P | 30 | 59686 | IGS | 5.64E-09 |
| 33 | 61838 | cemA | P | 33 | 61838 | cemA | 1.30E-05 |
| 30 | 72250 | IGS | F | 30 | 72275 | IGS | 6.18E-04 |
| 42 | 74548 | IGS | P | 42 | 74548 | IGS | 2.60E-12 |
| 52 | 76939 | IGS | P | 52 | 76939 | IGS | 3.82E-18 |
| 48 | 91100 | ycf2 | F | 48 | 91133 | ycf2 | 3.83E-14 |
| 48 | 91100 | ycf2 | P | 48 | 145340 | ycf2 | 3.83E-14 |
| 36 | 91112 | ycf2 | F | 36 | 91163 | ycf2 | 1.49E-10 |
| 36 | 91112 | ycf2 | F | 36 | 91181 | ycf2 | 7.80E-09 |
| 36 | 91112 | ycf2 | P | 36 | 145304 | ycf2 | 7.80E-09 |
| 36 | 91112 | ycf2 | P | 36 | 145322 | ycf2 | 1.49E-10 |
| 35 | 91113 | ycf2 | F | 35 | 91146 | ycf2 | 5.50E-12 |
| 35 | 91113 | ycf2 | P | 35 | 145340 | ycf2 | 5.50E-12 |
| 48 | 91133 | ycf2 | P | 48 | 145373 | ycf2 | 3.83E-14 |
| 59 | 91146 | ycf2 | F | 59 | 91164 | ycf2 | 3.46E-24 |
| 45 | 91146 | ycf2 | F | 45 | 91182 | ycf2 | 2.01E-12 |
| 45 | 91146 | ycf2 | P | 45 | 145294 | ycf2 | 2.01E-12 |
| 59 | 91146 | ycf2 | P | 59 | 145298 | ycf2 | 3.46E-24 |
| 35 | 91146 | ycf2 | P | 35 | 145373 | ycf2 | 5.50E-12 |
| 34 | 91157 | ycf2 | F | 34 | 91193 | ycf2 | 1.11E-07 |
| 34 | 91157 | ycf2 | P | 34 | 145294 | ycf2 | 1.11E-07 |
| 36 | 91163 | ycf2 | P | 36 | 145373 | ycf2 | 1.49E-10 |
| 59 | 91164 | ycf2 | P | 59 | 145316 | ycf2 | 3.46E-24 |
| 30 | 91175 | ycf2 | F | 30 | 91193 | ycf2 | 5.64E-09 |
| 30 | 91175 | ycf2 | P | 30 | 145298 | ycf2 | 5.64E-09 |
| 36 | 91181 | ycf2 | P | 36 | 145373 | ycf2 | 7.80E-09 |
| 45 | 91182 | ycf2 | P | 45 | 145330 | ycf2 | 2.01E-12 |
| 30 | 91193 | ycf2 | P | 30 | 145316 | IGS | 5.64E-09 |
| 34 | 91193 | ycf2 | P | 34 | 145330 | ycf2 | 1.11E-07 |
| 31 | 92472 | ycf2 | F | 31 | 92502 | ycf2 | 1.41E-09 |
| 31 | 92472 | ycf2 | P | 31 | 143988 | ycf2 | 1.41E-09 |
| 31 | 92502 | ycf2 | P | 31 | 144018 | ycf2 | 1.41E-09 |
| 40 | 98282 | ycf2 | P | 40 | 117076 | Intron | 5.37E-15 |
| 36 | 109034 | IGS | F | 36 | 109052 | IGS | 7.80E-09 |
| 36 | 109034 | IGS | P | 36 | 127433 | ycf1 | 7.80E-09 |
| 36 | 109052 | IGS | P | 36 | 127451 | ycf1 | 7.80E-09 |
| 40 | 117076 | Intron | F | 40 | 138199 | IGS | 5.37E-15 |
| 30 | 118583 | IGS | P | 30 | 118583 | IGS | 2.21E-05 |
| 30 | 119665 | IGS | F | 30 | 119686 | IGS | 6.18E-04 |
| 36 | 127433 | ycf1 | F | 36 | 127451 | ycf1 | 7.80E-09 |
| 31 | 143988 | ycf2 | F | 31 | 144018 | ycf2 | 1.41E-09 |
| 45 | 145294 | ycf2 | F | 45 | 145330 | ycf2 | 2.01E-12 |
| 59 | 145298 | ycf2 | F | 59 | 145316 | ycf2 | 3.46E-24 |
| 36 | 145304 | ycf2 | F | 36 | 145373 | ycf2 | 7.80E-09 |
| 30 | 145311 | ycf2 | F | 30 | 145347 | ycf2 | 6.18E-04 |
| 36 | 145322 | ycf2 | F | 36 | 145373 | ycf2 | 1.49E-10 |
| 30 | 145329 | ycf2 | F | 30 | 145347 | ycf2 | 2.21E-05 |
| 48 | 145340 | ycf2 | F | 48 | 145373 | ycf2 | 3.83E-14 |

**Table S7 SSR sequences identified in the plastome of *I. cirrhosa.***

| **Count** | **SSR seqeunce** | **Size** | **Start** | **End** | **Region** |
| --- | --- | --- | --- | --- | --- |
| 1 | (T)8 | 8 | 140 | 147 | IGS |
| 2 | (A)13 | 13 | 314 | 326 | IGS |
| 3 | (A)8 | 8 | 1663 | 1670 | IGS |
| 4 | (T)9 | 9 | 3524 | 3532 | CDS |
| 5 | (AC)4 …… (GA)4 | 82 | 3828 | 3909 | IGS |
| 6 | (A)8 …… (A)8 …… (A)9 | 94 | 4331 | 4424 | IGS |
| 7 | (A)10 | 10 | 4596 | 4605 | IGS |
| 8 | (A)8 | 8 | 4956 | 4963 | IGS |
| 9 | (TA)4 | 8 | 6431 | 6438 | IGS |
| 10 | (A)10 | 10 | 7674 | 7683 | IGS |
| 11 | (T)11 | 11 | 8003 | 8013 | IGS |
| 12 | (T)8 …… (A)8t(A)12 | 50 | 8160 | 8209 | IGS |
| 13 | (TA)5 …… (T)10 | 87 | 9768 | 9854 | IGS |
| 14 | (A)12 | 12 | 10071 | 10082 | IGS |
| 15 | (A)10 | 10 | 11652 | 11661 | IGS |
| 16 | (A)8 …… (T)11 | 93 | 12263 | 12355 | IGS |
| 17 | (T)10 | 10 | 13024 | 13033 | IGS |
| 18 | (A)10 | 10 | 13571 | 13580 | IGS |
| 19 | (AT)5 …… (T)8 | 59 | 14068 | 14126 | IGS |
| 20 | (A)8 | 8 | 14443 | 14450 | IGS |
| 21 | (T)10 …… (T)12 | 25 | 15341 | 15365 | IGS |
| 22 | (T)9 …… (A)10 …… (A)10 | 31 | 16162 | 16192 | IGS |
| 23 | (T)11 | 11 | 18368 | 18378 | CDS |
| 24 | (A)8 | 8 | 18511 | 18518 | CDS |
| 25 | (AT)5 | 10 | 19747 | 19756 | CDS |
| 26 | (AT)5 | 10 | 20775 | 20784 | CDS |
| 27 | (T)9 | 9 | 20999 | 21007 | CDS |
| 28 | (T)8 | 8 | 26093 | 26100 | CDS |
| 29 | (A)8 | 8 | 27957 | 27964 | IGS |
| 30 | (T)8 | 8 | 28449 | 28456 | IGS |
| 31 | (A)11 | 11 | 28884 | 28894 | IGS |
| 32 | (T)8 | 8 | 29292 | 29299 | IGS |
| 33 | (T)10 | 10 | 29883 | 29892 | IGS |
| 34 | (A)9 …… (T)8 | 31 | 30105 | 30135 | IGS |
| 35 | (TC)5 | 10 | 31087 | 31096 | IGS |
| 36 | (T)10 | 10 | 31849 | 31858 | IGS |
| 37 | (AT)4 | 8 | 32495 | 32502 | IGS |
| 38 | (A)10 | 10 | 32812 | 32821 | IGS |
| 39 | (TTC)4 | 12 | 35573 | 35584 | IGS |
| 40 | (A)10 …… (T)9 | 28 | 35769 | 35796 | IGS |
| 41 | (A)8 …… (GA)4 | 38 | 35929 | 35966 | IGS |
| 42 | (A)8 | 8 | 36707 | 36714 | IGS |
| 43 | (CAAA)3 …… (TA)4 | 56 | 42893 | 42948 | IGS |
| 44 | (T)9 | 9 | 43804 | 43812 | Intron |
| 45 | (A)10 | 10 | 44812 | 44821 | Intron |
| 46 | (T)8 | 8 | 45093 | 45100 | IGS |
| 47 | (G)9 | 9 | 45581 | 45589 | IGS |
| 48 | (T)8 …… (T)11 | 36 | 45697 | 45732 | IGS |
| 49 | (A)10 …… (A)14 | 27 | 47045 | 47071 | IGS |
| 50 | (AT)4 | 8 | 47520 | 47527 | IGS |
| 51 | (A)8 | 8 | 47840 | 47847 | IGS |
| 52 | (T)8 | 8 | 48291 | 48298 | IGS |
| 53 | (T)8 | 8 | 49171 | 49178 | IGS |
| 54 | (T)9 | 9 | 51051 | 51059 | IGS |
| 55 | (T)8 | 8 | 51563 | 51570 | IGS |
| 56 | (T)8 | 8 | 51675 | 51682 | IGS |
| 57 | (T)9 | 9 | 52562 | 52570 | IGS |
| 58 | (T)8 | 8 | 54518 | 54525 | CDS |
| 59 | (T)9 | 9 | 54942 | 54950 | IGS |
| 60 | (A)8 …… (TA)4 | 42 | 55066 | 55107 | IGS |
| 61 | (GA)4 | 8 | 55791 | 55798 | IGS |
| 62 | (A)8 …… (T)16 | 62 | 57126 | 57187 | IGS |
| 63 | (A)8 | 8 | 58040 | 58047 | IGS |
| 64 | (G)8 | 8 | 58423 | 58430 | IGS |
| 65 | (TA)4 | 8 | 59016 | 59023 | IGS |
| 66 | (T)9 …… (AT)4 | 41 | 59424 | 59464 | IGS |
| 67 | (TA)4 | 8 | 59594 | 59601 | IGS |
| 68 | (A)8 …… (A)8 | 49 | 59731 | 59779 | IGS |
| 69 | (T)8 | 8 | 59967 | 59974 | IGS |
| 70 | (A)8 | 8 | 60846 | 60853 | IGS |
| 71 | (AATG)3 | 12 | 61852 | 61863 | IGS |
| 72 | (T)8 …… (AT)4 | 60 | 62132 | 62191 | IGS |
| 73 | (A)8 | 8 | 62544 | 62551 | IGS |
| 74 | (A)9 | 9 | 63134 | 63142 | IGS |
| 75 | (A)9 | 9 | 63882 | 63890 | IGS |
| 76 | (T)9 | 9 | 65519 | 65527 | IGS |
| 77 | (TA)5 …… (A)8 …… (A)9 | 76 | 65644 | 65719 | IGS |
| 78 | (T)8 | 8 | 66357 | 66364 | IGS |
| 79 | (T)10 | 10 | 66509 | 66518 | IGS |
| 80 | (AT)4(ATA)4 …… (T)11 | 119 | 67065 | 67183 | IGS |
| 81 | (A)8 | 8 | 67485 | 67492 | IGS |
| 82 | (A)15 | 15 | 68559 | 68573 | IGS |
| 83 | (T)8 | 8 | 68737 | 68744 | IGS |
| 84 | (T)11 | 11 | 69292 | 69302 | IGS |
| 85 | (T)9 | 9 | 69753 | 69761 | IGS |
| 86 | (A)8 | 8 | 70426 | 70433 | Intron |
| 87 | (T)8 | 8 | 70643 | 70650 | Intron |
| 88 | (T)8 | 8 | 70974 | 70981 | Intron |
| 89 | (T)8 …… (A)11 | 23 | 71488 | 71510 | Intron |
| 90 | (T)9 | 9 | 71727 | 71735 | Intron |
| 91 | (TA)4 | 8 | 75408 | 75415 | IGS |
| 92 | (T)8 | 8 | 77311 | 77318 | IGS |
| 93 | (T)9 | 9 | 78119 | 78127 | IGS |
| 94 | (TA)4 …… (T)10 | 91 | 78290 | 78380 | CDS |
| 95 | (T)13 | 13 | 80299 | 80311 | IGS |
| 96 | (T)10 …… (T)8 …… (TAAT)3 | 86 | 80812 | 80897 | IGS |
| 97 | (A)10 …… (T)10 | 99 | 81356 | 81454 | IGS |
| 98 | (T)8 | 8 | 82846 | 82853 | Intron |
| 99 | (AT)6 …… (TA)4 | 111 | 84139 | 84249 | IGS |
| 100 | (T)9 …… (T)10 | 45 | 84543 | 84587 | CDS |
| 101 | (TA)4 | 8 | 85168 | 85175 | Intron |
| 102 | (GA)4 …… (GA)4 | 20 | 86775 | 86794 | IGS |
| 103 | (A)9 | 9 | 89320 | 89328 | IGS |
| 104 | (TA)4 | 8 | 92769 | 92776 | IGS |
| 105 | (AG)4 | 8 | 94730 | 94737 | CDS |
| 106 | (A)9 | 9 | 96631 | 96639 | IGS |
| 107 | (T)9 | 9 | 97368 | 97376 | CDS |
| 108 | (T)10 | 10 | 98640 | 98649 | IGS |
| 109 | (T)11 | 11 | 102190 | 102200 | IGS |
| 110 | (G)9 | 9 | 103390 | 103398 | IGS |
| 111 | (CT)4 | 8 | 105509 | 105516 | IGS |
| 112 | (T)8 …… (A)11 | 38 | 109625 | 109662 | IGS |
| 113 | (A)9 | 9 | 109861 | 109869 | IGS |
| 114 | (A)10 | 10 | 111282 | 111291 | IGS |
| 115 | (A)9 …… (A)9 …… (A)8 | 118 | 111535 | 111652 | IGS |
| 116 | (A)8 | 8 | 111845 | 111852 | IGS |
| 117 | (A)8 | 8 | 112360 | 112367 | IGS |
| 118 | (A)9 …… (TA)4 | 32 | 112607 | 112638 | IGS |
| 119 | (T)8 | 8 | 112870 | 112877 | IGS |
| 120 | (GA)4 | 8 | 113054 | 113061 | IGS |
| 121 | (A)9 | 9 | 113514 | 113522 | IGS |
| 122 | (T)9 | 9 | 114042 | 114050 | IGS |
| 123 | (A)9 | 9 | 114267 | 114275 | IGS |
| 124 | (AT)4 …… (A)8 …… (GATT)3 | 187 | 116235 | 116421 | IGS |
| 125 | (AGAA)3 | 12 | 117735 | 117746 | IGS |
| 126 | (TA)8 | 16 | 118591 | 118606 | IGS |
| 127 | (T)8 | 8 | 120707 | 120714 | IGS |
| 128 | (T)8 | 8 | 122739 | 122746 | CDS |
| 129 | (T)8 | 8 | 123094 | 123101 | IGS |
| 130 | (A)10 | 10 | 123666 | 123675 | IGS |
| 131 | (T)18 | 18 | 123809 | 123826 | IGS |
| 132 | (A)8 …… (T)8 | 98 | 124168 | 124265 | IGS |
| 133 | (T)8 | 8 | 124478 | 124485 | IGS |
| 134 | (AG)4 | 8 | 131006 | 131013 | IGS |
| 135 | (C)9 | 9 | 133124 | 133132 | IGS |
| 136 | (A)11 | 11 | 134322 | 134332 | IGS |
| 137 | (A)10 | 10 | 137873 | 137882 | IGS |
| 138 | (A)9 | 9 | 139146 | 139154 | IGS |
| 139 | (T)9 | 9 | 139883 | 139891 | IGS |
| 140 | (CT)4 | 8 | 141785 | 141792 | IGS |
| 141 | (TA)4 | 8 | 143746 | 143753 | IGS |
| 142 | (T)9 | 9 | 147194 | 147202 | CDS |
| 143 | (TC)4 …… (TC)4 | 20 | 149728 | 149747 | CDS |
| 144 | (AT)4 | 8 | 151346 | 151353 | IGS |
| 145 | (A)10 …… (A)9 | 45 | 151935 | 151979 | IGS |

**Note:** IGS: intergenic spacers, CDS: coding sequence, Intron: intronic sequence.

**Table S8 Tandem repeat sequences identified in the *I. cirrhosa* plastome using the program Tandem Repeat Finder.**

| **Start** | **End** | **Size of Repeat Unit** | **Copy of Repeat Units** | **Matches between adjacent copies (%)** | **Alignment score** | **Sequence of the repeat unit** | **Sequence of the entire repeat** |
| --- | --- | --- | --- | --- | --- | --- | --- |
| 8431 | 8460 | 15 | 1.9 | 93 | 53 | TATATAATTAATATAT | TATATAATAATATATTATATAATTAATATA |
| 9651 | 9688 | 11 | 3.1 | 79 | 51 | TCTATTACATTA | TCTATTACATTATTATTACATTATCTCTATTACATTAT |
| 9649 | 9688 | 14 | 3.1 | 79 | 59 | TCTCTATTACATTA | TCTCTATTACATTATTATTACATTATCTCTATTACATTAT |
| 16171 | 16196 | 11 | 2.4 | 100 | 52 | GAAAAAAAAAA | GAAAAAAAAAAGAAAAAAAAAAGAAA |
| 28916 | 28945 | 13 | 2.3 | 100 | 60 | GAATGTATTATAT | GAATGTATTATATGAATGTATTATATGAAT |
| 31896 | 31951 | 18 | 3.1 | 97 | 103 | TATAATACATTATCATTA | TATAATACATTATCATTATATAATACATTATCATTATATAATACATTATCGTTATA |
| 32264 | 32298 | 17 | 2.1 | 100 | 70 | TTCTTTATTTTTTATTA | TTCTTTATTTTTTATTATTCTTTATTTTTTATTAT |
| 32257 | 32303 | 24 | 2 | 82 | 58 | TTCTTTATTCTTTATTATCTATTA | TTCTTTCTTCTTTATTTTTTATTATTCTTTATTTTTTATTATCTATT |
| 42259 | 42288 | 15 | 2 | 100 | 60 | GTCAAATAACTAATT | GTCAAATAACTAATTGTCAAATAACTAATT |
| 42914 | 42945 | 16 | 2 | 100 | 64 | TATTCTATATCTATAT | TATTCTATATCTATATTATTCTATATCTATAT |
| 47038 | 47075 | 15 | 2.4 | 86 | 60 | ATAGAATAAAAAAAAA | ATAGAATAAAAAAAAAATAGAAAAAAAAAAAAAAGAAT |
| 47506 | 47539 | 16 | 2.2 | 89 | 50 | TATATATTCATAGAA | TATATATTCATAGAATATATATTTCATATAATAT |
| 57909 | 57981 | 24 | 3 | 95 | 137 | GAGAAAGTTCGAATGATCTCGATG | GAGAAAGTTCGAATGATCTCGATGGAGAAAGTTCGAATGATTTCGATGGAGAAAGTTCGAATGATCTCGATGG |
| 72251 | 72300 | 25 | 2 | 100 | 100 | TTGATGTGTAAACCTAAAATAAAAA | TTGATGTGTAAACCTAAAATAAAAATTGATGTGTAAACCTAAAATAAAAA |
| 83009 | 83051 | 20 | 2.1 | 95 | 79 | ATTTTGATATTCTATCACCGG | ATTTTGATATTCTATCACCGGATTTTGTATTCTATCACCGGAT |
| 91101 | 91215 | 18 | 6.8 | 89 | 117 | TATTGATGATAGTCGA | TATTGATTATATCGATATTGATGATAGTGACGATATTGATGATAGTGATATTGATGATAGTGACGATATTGATGATAGTGACGATATTGATGCTAGTGACGATATTGATGCTAGT |
| 91113 | 91223 | 18 | 6.3 | 92 | 183 | CGATATTGATGATAGTGA | CGATATTGATGATAGTGACGATATTGATGATAGTGATATTGATGATAGTGACGATATTGATGATAGTGACGATATTGATGCTAGTGACGATATTGATGCTAGTGACGATAT |
| 91113 | 91222 | 9 | 12.6 | 70 | 73 | CGATAGTGA | CGATATTGATGATAGTGACGATATTGATGATAGTGATATTGATGATAGTGACGATATTGATGATAGTGACGATATTGATGCTAGTGACGATATTGATGCTAGTGACGATA |
| 91101 | 91215 | 33 | 3.4 | 90 | 171 | TATTGATGATAGTCGATATTGATGATAGTGACGA | TATTGATTATATCGATATTGATGATAGTGACGATATTGATGATAGTGATATTGATGATAGTGACGATATTGATGATAGTGACGATATTGATGCTAGTGACGATATTGATGCTAGT |
| 92473 | 92533 | 30 | 2 | 100 | 122 | ATTTATGATGAAGAGGATGAGCTTCAAGAG | ATTTATGATGAAGAGGATGAGCTTCAAGAGATTTATGATGAAGAGGATGAGCTTCAAGAGA |
| 98627 | 98666 | 18 | 2.2 | 90 | 64 | TTTTTATTTAATATTTTAT | TTTTTATTTACTATTTTTTTTTTATTTAATATTTTATTTT |
| 109039 | 109088 | 18 | 2.8 | 96 | 91 | GATCTAATAAGTACATTC | GATCGAATAAGTACATTCGATCTAATAAGTACATTCGATCTAATAAGTAC |
| 119658 | 119707 | 21 | 2.6 | 75 | 55 | ATAAAAATATTAATTAT | ATAAATAATATTAATTATATAAAAATATTTATTAATTATATAAAAATATT |
| 119666 | 119707 | 21 | 2 | 100 | 84 | TATTAATTATATAAAAATATT | TATTAATTATATAAAAATATTTATTAATTATATAAAAATATT |
| 127434 | 127483 | 18 | 2.8 | 96 | 91 | GTACTTATTAGATCGAAT | GTACTTATTAGATCGAATGTACTTATTAGATCGAATGTACTTATTCGATC |
| 137856 | 137896 | 18 | 2.3 | 86 | 64 | AAAATAAAATAGTAAATA | AAAATAAAATATTAAATAAAAAAAAAATAGTAAATAAAAAT |
| 143989 | 144049 | 30 | 2 | 100 | 122 | TCTCTTGAAGCTCATCCTCTTCATCATAAA | TCTCTTGAAGCTCATCCTCTTCATCATAAATCTCTTGAAGCTCATCCTCTTCATCATAAAT |
| 145312 | 145408 | 9 | 11.1 | 73 | 92 | CATCAATAT | CATCAATATCGTCACTAGCATCAATATCGTCACTATCATCAATATCGTCACTATCATCAATATCACTATCATCAATATCGTCACTATCATCAATATC |
| 145305 | 145421 | 33 | 3.5 | 90 | 164 | TCACTATCATCAATATCGTCACTATCATCAATA | TCACTAGCATCAATATCGTCACTAGCATCAATATCGTCACTATCATCAATATCGTCACTATCATCAATATCACTATCATCAATATCGTCACTATCATCAATATCGATATAATCAATA |
| 145299 | 145409 | 18 | 6.3 | 92 | 183 | ATATCGTCACTATCATCA | ATATCGTCACTAGCATCAATATCGTCACTAGCATCAATATCGTCACTATCATCAATATCGTCACTATCATCAATATCACTATCATCAATATCGTCACTATCATCAATATCG |

**Table S9 Kimura-2-parameter distances calculated for the most divergent coding and non-coding regions .**

| **Gene** | **Pair of species** | **K2P distance** | **Mean Distance** | **Standard Deviation of the distance** |
| --- | --- | --- | --- | --- |
| accD | *I. cirrhosa vs I. scandens* | 3.27 | 2.73 | 2.14 |
|  | *I. klaineana vs I. cirrhosa* | 1.26 |  |  |
|  | *I. klaineana vs I. liberica* | 6.11 |  |  |
|  | *I. klaineana vs I. perrieri* | 0.7 |  |  |
|  | *I. klaineana vs I. scandens* | 3.27 |  |  |
|  | *I. klaineana vs I. seretii* | 0.46 |  |  |
|  | *I. liberica vs I. cirrhosa* | 6.59 |  |  |
|  | *I. liberica vs I. scandens* | 2.94 |  |  |
|  | *I. liberica vs I. seretii* | 0.44 |  |  |
|  | *I. perrieri vs I. cirrhosa* | 1.35 |  |  |
|  | *I. perrieri vs I. liberica* | 6.07 |  |  |
|  | *I. perrieri vs I. scandens* | 3.28 |  |  |
|  | *I. perrieri vs I. seretii* | 0.7 |  |  |
|  | *I. seretii vs I. cirrhosa* | 1.05 |  |  |
|  | *I. seretii vs I. scandens* | 3.4 |  |  |
| ndhF | *I. cirrhosa vs I. scandens* | 3.82 | 15.55 | 10.16 |
|  | *I. klaineana vs I. cirrhosa* | 21.26 |  |  |
|  | *I. klaineana vs I. liberica* | 1.23 |  |  |
|  | *I. klaineana vs I. perrieri* | 21.13 |  |  |
|  | *I. klaineana vs I. scandens* | 23.51 |  |  |
|  | *I. klaineana vs I. seretii* | 2.91 |  |  |
|  | *I. liberica vs I. cirrhosa* | 21.82 |  |  |
|  | *I. liberica vs I. scandens* | 24.26 |  |  |
|  | *I. liberica vs I. seretii* | 2.31 |  |  |
|  | *I. seretii vs I. cirrhosa* | 23.47 |  |  |
|  | *I. perrieri vs I. liberica* | 23.78 |  |  |
|  | *I. perrieri vs I. scandens* | 6.31 |  |  |
|  | *I. perrieri vs I. seretii* | 25.01 |  |  |
|  | *I. perrieri vs I. cirrhosa* | 5.75 |  |  |
|  | *I. seretii vs I. scandens* | 26.63 |  |  |
| psaA | *I. cirrhosa vs I. scandens* | 0.62 | 39.74 | 39.48 |
|  | *I. klaineana vs I. cirrhosa* | 1.07 |  |  |
|  | *I. klaineana vs I. liberica* | 86.56 |  |  |
|  | *I. klaineana vs I. perrieri* | 0.53 |  |  |
|  | *I. klaineana vs I. scandens* | 0.91 |  |  |
|  | *I. klaineana vs I. seretii* | 62.93 |  |  |
|  | *I. liberica vs I. cirrhosa* | 100.54 |  |  |
|  | *I. liberica vs I. scandens* | 86.95 |  |  |
|  | *I. liberica vs I. seretii* | 1.88 |  |  |
|  | *I. perrieri vs I. cirrhosa* | 1.29 |  |  |
|  | *I. perrieri vs I. liberica* | 81.48 |  |  |
|  | *I. perrieri vs I. scandens* | 0.99 |  |  |
|  | *I. perrieri vs I. seretii* | 55.46 |  |  |
|  | *I. seretii vs I. cirrhosa* | 51.68 |  |  |
|  | *I. seretii vs I. scandens* | 63.17 |  |  |
| rbcL | *I. cirrhosa vs I. scandens* | 0.78 | 1.27 | 0.35 |
|  | *I. klaineana vs I. cirrhosa* | 1.56 |  |  |
|  | *I. klaineana vs I. liberica* | 0.49 |  |  |
|  | *I. klaineana vs I. perrieri* | 0.99 |  |  |
|  | *I. klaineana vs I. scandens* | 1.63 |  |  |
|  | *I. klaineana vs I. seretii* | 1.2 |  |  |
|  | *I. liberica vs I. cirrhosa* | 1.56 |  |  |
|  | *I. liberica vs I. scandens* | 1.63 |  |  |
|  | *I. liberica vs I. seretii* | 1.2 |  |  |
|  | *I. perrieri vs I. cirrhosa* | 1.57 |  |  |
|  | *I. perrieri vs I. liberica* | 0.92 |  |  |
|  | *I. perrieri vs I. scandens* | 1.56 |  |  |
|  | *I. perrieri vs I. seretii* | 1.28 |  |  |
|  | *I. seretii vs I. cirrhosa* | 1.2 |  |  |
|  | *I. seretii vs I. scandens* | 1.42 |  |  |
| ycf1 | *I. cirrhosa vs I. scandens* | 1.9 | 2.11 | 1.49 |
|  | *I. klaineana vs I. cirrhosa* | 2.28 |  |  |
|  | *I. klaineana vs I. liberica* | 0 |  |  |
|  | *I. klaineana vs I. perrieri* | 3.74 |  |  |
|  | *I. klaineana vs I. scandens* | 0.64 |  |  |
|  | *I. klaineana vs I. seretii* | 0.46 |  |  |
|  | *I. liberica vs I. cirrhosa* | 2.56 |  |  |
|  | *I. liberica vs I. scandens* | 0.72 |  |  |
|  | *I. liberica vs I. seretii* | 0.41 |  |  |
|  | *I. perrieri vs I. cirrhosa* | 2.74 |  |  |
|  | *I. perrieri vs I. liberica* | 4.22 |  |  |
|  | *I. perrieri vs I. scandens* | 4.15 |  |  |
|  | *I. perrieri vs I. seretii* | 4.05 |  |  |
|  | *I. seretii vs I. cirrhosa* | 2.67 |  |  |
|  | *I. seretii vs I. scandens* | 1.1 |  |  |
| ycf2 | *I. cirrhosa vs I. scandens* | 2.72 | 1.07 | 1.32 |
|  | *I. klaineana vs I. cirrhosa* | 0.14 |  |  |
|  | *I. klaineana vs I. liberica* | 0.11 |  |  |
|  | *I. klaineana vs I. perrieri* | 0.13 |  |  |
|  | *I. klaineana vs I. scandens* | 2.86 |  |  |
|  | *I. klaineana vs I. seretii* | 0.16 |  |  |
|  | *I. liberica vs I. cirrhosa* | 0.22 |  |  |
|  | *I. liberica vs I. scandens* | 2.97 |  |  |
|  | *I. liberica vs I. seretii* | 0.24 |  |  |
|  | *I. perrieri vs I. cirrhosa* | 0.14 |  |  |
|  | *I. perrieri vs I. liberica* | 0.21 |  |  |
|  | *I. perrieri vs I. scandens* | 2.89 |  |  |
|  | *I. perrieri vs I. seretii* | 0.16 |  |  |
|  | *I. seretii vs I. cirrhosa* | 0.18 |  |  |
|  | *I. seretii vs I. scandens* | 2.91 |  |  |
| trnH-GUG/psbA | *I. cirrhosa vs I. scandens* | 103.01 | 85.61 | 71.57 |
|  | *I. klaineana vs I. cirrhosa* | 0 |  |  |
|  | *I. klaineana vs I. liberica* | 12.27 |  |  |
|  | *I. klaineana vs I. perrieri* | 95.08 |  |  |
|  | *I. klaineana vs I. scandens* | 212.31 |  |  |
|  | *I. klaineana vs I. seretii* | 47.22 |  |  |
|  | *I. liberica vs I. cirrhosa* | 162.37 |  |  |
|  | *I. liberica vs I. scandens* | 212.31 |  |  |
|  | *I. liberica vs I. seretii* | 30.46 |  |  |
|  | *I. perrieri vs I. cirrhosa* | 8.18 |  |  |
|  | *I. perrieri vs I. liberica* | 137.44 |  |  |
|  | *I. perrieri vs I. scandens* | 40.52 |  |  |
|  | *I. perrieri vs I. seretii* | 96.78 |  |  |
|  | *I. seretii vs I. cirrhosa* | 109.24 |  |  |
|  | *I. seretii vs I. scandens* | 17.02 |  |  |
| rps16_trnQ/UUG | *I. cirrhosa vs I. scandens* | 3.37 | 32.75 | 18.87 |
|  | *I. klaineana vs I. cirrhosa* | 62.82 |  |  |
|  | *I. klaineana vs I. liberica* | 11.59 |  |  |
|  | *I. klaineana vs I. perrieri* | 27.85 |  |  |
|  | *I. klaineana vs I. scandens* | 50.47 |  |  |
|  | *I. klaineana vs I. seretii* | 41.02 |  |  |
|  | *I. liberica vs I. cirrhosa* | 52.18 |  |  |
|  | *I. liberica vs I. scandens* | 47.17 |  |  |
|  | *I. liberica vs I. seretii* | 35.54 |  |  |
|  | *I. perrieri vs I. cirrhosa* | 56.78 |  |  |
|  | *I. perrier vs I. liberica* | 11.94 |  |  |
|  | *I. perrier vs I. scandens* | 36.17 |  |  |
|  | *I.perrier vs I. seretii* | 10.98 |  |  |
|  | *I. seretii vs I. cirrhosa* | 25.6 |  |  |
|  | *I. scandens vs I. seretii* | 17.81 |  |  |
| psbM/trnD-GUC | *I. cirrhosa vs I. scandens* | 10.57 | 5.58 | 3.73 |
|  | *I. klaineana vs I. cirrhosa* | 2.51 |  |  |
|  | *I. klaineana vs I. liberica* | 0.52 |  |  |
|  | *I. klaineana vs I. perrier* | 2.24 |  |  |
|  | *I. klaineana vs I. scandens* | 9.17 |  |  |
|  | *I. klaineana vs I. seretii* | 4.36 |  |  |
|  | *I. liberica vs I. cirrhosa* | 1.95 |  |  |
|  | *I. liberica vs I. scandens* | 11.31 |  |  |
|  | *I. liberica vs I. seretii* | 3.85 |  |  |
|  | *I. perrieri vs I. cirrhosa* | 4.73 |  |  |
|  | *I. perrier vs I. liberica* | 2.4 |  |  |
|  | *I. perrier vs I. scandens* | 12.61 |  |  |
|  | *I. perrier vs I. seretii* | 5.84 |  |  |
|  | *I. seretii vs I. cirrhosa* | 5.6 |  |  |
|  | *I. seretii vs I. scandens* | 6.1 |  |  |
| petA/psbJ | *I. cirrhosa vs I. scandens* | 88.67 | 37.89 | 34.05 |
|  | *I.klaineana vs I. cirrhosa* | 7.21 |  |  |
|  | *I.klaineana vs I. liberica* | 2.37 |  |  |
|  | *I.klaineana vs I. perrieri* | 10.66 |  |  |
|  | *I.klaineana vs I. scandens* | 90.67 |  |  |
|  | *I.klaineana vs I. seretii* | 31.36 |  |  |
|  | *I. liberica vs I. cirrhosa* | 4.96 |  |  |
|  | *I. liberica vs I. scandens* | 83.06 |  |  |
|  | *I. liberica vs I. seretii* | 30.27 |  |  |
|  | *I. perrieri vs I. cirrhosa* | 8.54 |  |  |
|  | *I. perrieri vs I. liberica* | 5.81 |  |  |
|  | *I. perrieri vs I. scandens* | 88.55 |  |  |
|  | *I. perrieri vs I. seretii* | 34.23 |  |  |
|  | *I. seretii vs I. cirrhosa* | 30.74 |  |  |
|  | *I. seretii vs I. scandens* | 51.28 |  |  |
