# Supplementary material for: Complete plastome sequence of Iodes cirrhosa Turcz., the first in the Icacinaceae, comparative genomic analyses and possible split of Idoes species in response to climate changes
Source: PeerJ. 2019 Apr 1;7:e6663. doi: 10.7717/peerj.6663 (PMC6448556; doi:10.7717/peerj.6663)
Supplement: Supplemental Information 2 [file peerj-07-6663-s002.docx]

**Supplemental Figures**

**Complete plastome sequence of *Iodes cirrhosa* Turcz., the first in the Icacinaceae, comparative genomic analyses and possible split of *Iodes* species in response to climate changes**

***Liqiang Wang******^¶^, Hui Zhang******^¶^, Mei Jiang, Haimei Chen, Linfang Huang*, Chang Liu****

Key Laboratory of Bioactive Substances and Resource Utilization of Chinese Herbal Medicine from Ministry of Education, Institute of Medicinal Plant Development, Chinese Academy of Medical Sciences and Peking Union Medical College, Beijing, P. R. China.

E-mails:

[lys832000@163.com](mailto:lys832000@163.com) (LQW);

[18238801021@163.com](mailto:18238801021@163.com) (HZ);

[mjiang0502@163.com](mailto:mjiang0502@163.com) (MJ);

[hmchen@implad.ac.cn](mailto:hmchen@implad.ac.cn) (HMC);

15801545922@139.com (LFH);

[cliu6688@yahoo.com](mailto:cliu6688@yahoo.com) (CL)

**^¶^**These authors contributed equally to this work.

*****Correspondence:

Professors Chang Liu, Linfang Huang

cliu6688@yahoo.com; Tel.: +86-010-5783-3111; Fax: +86-10-62899715

15801545922@139.com; Tel: +86-010-5783-3197; Fax: +86-10-62899715

* 20 * 40 * 60 * 80 * 100 * 120 *
Ikl : -----GEENSMERWWFNSVLFKKELKHRCGPSKSMDSLGPTENTSQNEDPNINDTAKNIHSWSDRG-----NSN----LVDLKDIRHLISDDTFLVRDSNENSYSIYFDIENQIFEIDNDHSFLSE---- : 112
Ici : MQINRGEENSMERWWFNSVLFKKELKHRCGPSKSMDSLGPTENTSQNEDPNINDTAKNIHSWSDRG-----NSN----LVDLKDIRNLISDDTFLVRDSNENSYSIYFDIENQIFEIDNDHSFLSEVESS : 121
Ise : -----GEENSMERWWFNSVLFKKELKHRCGPSKSMDSLGPSENTSQNEDPNINDTAKNIHSWSDRG-----NSN----LVDLQDIRNLISDDTFLVRDSNENSYSIYFDIENQIFEIDNDHSFLSEVESS : 116
Ili : -----GEENSMERWWFNSVLFKKELKHRCGPSKSMDSLGPTENTSQNEDPNINDTAKNIHSWSDRG-----NSN----LVDLKDIRNLISDDTFLVRDSNENSYSIYFDIEN--FEIDNDHSFLSEVESS : 114
Ipe : -----GEENSMERWWFNSVLFKKELKHRCGPSKSMDSLGPTENTSQNEDPNINDTAKNIHSWSDRG-----NSN----LVDLKDIRNLISDDTFLVRDSNENSYSIYFDIENQIFEIDNDHSFLSEVESS : 116
Isc : ----RSSDLIVERWWFNSVFFKKELKHRCGPSKSMDSLGPTENTSQNEDPNINDTAKNIHSWSHRG-----NSN----LVDLKDIRNLISDDTFLVRDSNENSYSIYFDIENQIFEIDNDHSFLSEVESS : 117
Ath : ----------MEKSWFNFMFSKGELEYRGELSKAMDSFAPGEKTTISQDRFIYDMDKNFYGWDERSSYSSSYSNNVDLLVSSKDIRNFISDDTFFVRDSNKNSYSIFFDKKKKIFEIDND---FSDLEKF : 117

140 * 160 * 180 * 200 * 220 * 240 * 260
Ikl : -----------------DDLHYDRYMYDTQSSWNNHINSCIDSYLQSQISIDTYIESGSDNSSDSYIYSYICGESGKSSESGSSSIRTSTSGSDLTLRESSNDLDGESSN--------DLDGTQKYRHLW : 217
Ici : FYNYRNSSYLNNRSK-SDDLHYDRYMYNTQSSWNNHINSCIDSYLQSQISIDTYIESGSDNSSDSYIYSSICGESRKSSESGSSSIRTSTSGSDLTLRESSNDLDGESSNDFDGESSNDLDGTQKYRHLW : 250
Ise : FSNYRNSSYLNNRSK-SDDLHYDRYMYDTQSSWNNHINSCIDSYL------------------------------------------------DLTLRESSNDLDGESSN--------DLDGTQKYRHLW : 189
Ili : FYNYRNSSYLNNRSK-SDDLHYDRYMYDTQSSWNNHINSCIDSYLQSQISIDTYIESGSDNSSDSYIYSYICGESGKSSESGSSSIRTSTSGSDLTLRESSNDLDGESSN--------DLDGTQKYRHLW : 235
Ipe : FYNYRNSSYLNNRSK-SDDLHYDRYMYDTQSSWNNHINSCIDSYLQSQISIDTYIESGSDNSSDSYIYSYICGESGKSSESGSSSIRTSTSGSDLTLRESSNDLDGESSN--------DLDGTQKYRHLW : 237
Isc : FYNYRNSSYLNNRSK-SDDLHYDRYMYDTQSSWNNHINSCIDSYLQSQISIDT----------------------------------------------------------------------------- : 169
Ath : FYSYCSSSYLNNRSKGDNDLHYDPYIKDTKYNCTNHINSCIDSYFRSYICIDNNFLIDSNNFNESYIYNFICSESGKIRES-----------KNYKIRTNRNRSNLISSK--------DFDITQNYNQLW : 228

 * 280 * 300 * 320 * 340 * 360 * 380 *
Ikl : IQCENCYALNYKKIFKSKMNICEQCGYHLKISSSDRIELSIDPGTWDPMDEDMVSLDPIEFHSEEESYKDRIDSYQRKTGLTEAVQTGIGQLNGIPIAIGVMDFQFMGGSMGSVVGEKITRLIEYATNKC : 347
Ici : IQCENCYALNYKKNFKSKMNICEQCGYHLKISSSDRIELSIDPGTWDPMDEDMVSLDPIEFHSEEESYKDRIDSYQRKTGLTEAVQTGIGQLNGIPIAIGVMDFQFMGGSMGSVVGEKITRLIEYATNKC : 380
Ise : I---------------------------------------------------------IEFHSEEESYKDRIDSYQRKTGLTEAVQTGIGQLNGIPIAIGVMDFQFMGGSMGSVVGEKITRLIEYATNKC : 262
Ili : IQCENCYALNYKI--------CEQCGYHLKISSSDRIELSIDPGTWDPMDEDMVSLDPIEFHSEEESYKDRIDSYQRKTGLTEAVQTGIGQLNGIPIAIGVMDFQFMGGSMGSVVGEKITRLIEYATNKC : 357
Ipe : IQCENCYALNYKKNLKSKMNICEQCGYHLKISSSDRIELSIDPGTWDPMDEDMVSLDPIEFHSEEESYKDRIDSYQRKTGLTEAVQTGIGQLNGIPIAIGVMDFQFMGGSMGSVVGEKITRLIEYATNKC : 367
Isc : ------------------------------------------------------------------SYKDRIDSYQRKTGLTEAVQTGIGQLNGIPIAIGVMDFQFMGGSMGSVVGEKITRLIEYATNKC : 233
Ath : IQCDNCYGLMYKK---VKMNVCEQCGHYLKMSSSERIELSIDPGTWNPMDEDMVSADPIKFHSKEEPYKNRIDSAQKTTGLTDAVQTGTGQLNGIPVALGVMDFRFMGGSMGSVVGEKITRLIEYATNQC : 355

400 * 420 * 440 * 460 * 480 * 500 * 520
Ikl : LPLVIVCASGGARMQEGSLSLMQMAKISSALYDYQSNKKLFYVSILTSPTTGGVTASFGMLGDIIIAEPNAYIAFAGKRVIEQTLNKTVPEGSQEAEYLFQKGLFDLIVPRNPLKNVLKELFQLHAFFPL : 477
Ici : LPLVIVCASGGARMQEGSLSLMQMAKISSALYDYQSNKKLFYVSILTSPTTGGVTASFGMLGDIIIAEPNAYIAFAGKRVIEQTLNKTVPEGSQEAEYLFQKGLFDLIVPRNPLKNVLRELFQLHAFFPL : 510
Ise : LPLVIVCASGGARMQEGSLSLMQMAKISSALYDYQSNKKLFYVSILTSPTTGGVTASFGMLGDIIIAEPNAYIAFAGKRVIEQTLNKTVPEGSQEAEYLFQKGLFDLIVPRNPLKNVLKE---------- : 382
Ili : LPLVIVCASGGARMQEGSLSLMQMAKISSALYDYQSNKKLFYVSILTSPTTGGVTASFGMLGDIIIAEPNAYIAFAGKRVIEQTLNKTVPEGSQEAEYLFQKGLFDLIVPRNPLKNVLKELFQLHAFFP- : 486
Ipe : LPLVIVCASGGARMQEGSLSLMQMAKISSALYDYQSNKKLFYVSILTSPTTGGVTASFGMLGDIIIAEPNAYIAFAGKRVIEQTLNKTVPEGSQEAEYLFQKGLFDLIVPRNPLKNVLKELFQLHAFFPL : 497
Isc : LPLVIVCASGGARMQEGSLSLMQMAKISSALYDYQSNKKLFYVSILTSPTTGGVTASFGMLGDIIIAEPNT-LLFAGKRVIEQTLNKTVPEGSQEAEYLFQKGLFDLIVPRNPLKNVLKELFQLHAFFPL : 362
Ath : LPLILVCSSGGARMQEGSLSLMQMAKISSVLCDYQSSKKLFYISILTSPTTGGVTASFGMLGDIIIAEPYAYIAFAGKRVIEQTLKKAVPEGSQAAESLLRKGLLDAIVPRNLLKGVLSELFQLHAFFPL : 485

**Figure S1 Multiple sequence alignment of the deduced amino acid sequences of ACCD protein.** The origins of the protein sequences used in this alignment are from: *Arabidopsis thaliana* (Ath), *Iodes klaineana* (Ikl), *Iodes cirrhosa* (Ici), *Iodes seretii* (Ise), *Iodes scandens* (Isc), *Iodes perrieri* (Ipe) and *Iodes liberica* (Ili). The amino acid numbers for each sequence are indicated on the right. In the sequence alignment, identical residues are shown with a black background, and similar residues are shown with a gray background.

* 20 * 40 * 60 * 80 * 100 * 120 *
Isc : -----------------------VKIVNTGTVLQVGDGIARIHGLDEVMAGELVEFEEGTIGIALNLESNNVGVVLMGDGLMIQEGSSVKATGRIAQIPVSEACLGRVLNALAKPIDGRGEISASESRLI : 107
Ici : MVTIRADEISNIIRERIEQYNREVKIVNTGTVLQVGDGIARIHGLDEVMAGELVEFEEGTIGIALNLESNNVGVVLMGDGLMIQEGSSVKATGRIAQIPVSEAYLGRVLNALAKPIDGRGEISASESRLI : 130
Ise : MVTIRADEISNIIRERIEQYNREVKIVNTGTVLQVGDGIARIHGLDEVMAGELVEFEEGTIGIALNLESNNVGVVLMGDGLMIQEGSSVKATGRIAQIPVSEAYLGRVLNALAKPIDGRGEISASESRLI : 130
Ipe : MVTIRADEISNIIRERIEQYNREVKIVNTGTVLQVGDGIARIHGLDEVMAGELVEFEEGTIGIALNLESNNVGVVLMGDGLMIQEGSSVKATGRIAQIPVSEAYLGRVLNALAKPIDGRGEISASESRLI : 130
Ili : MVTIRADEISNIIRERIEQYNREVKIVNTGTVLQVGDGIARIHGLDEVMAGELVEFEEGTIGIALNLESNNVGVVLMGDGLMIQEGSSVKATGRIAQIPVSEAYLGRVLNALAKPIDGRG-ISASESRLI : 129
Ikl : MVTIRADEISNIIRERIEQYN-EVKIVNTGTVLQVGDGIARIHGLDEVMAGELVEFEEGTIGIALNLESNNVGVVLMGDGLMIQEGSSVKATGRIAQIPVSEAYLGRVLNALAKPIDGRGEISASESRLI : 129
Ath : MVTIRADEISNIIRERIEQYNREVTIVNTGTVLQVGDGIARIYGLDEVMAGELVEFEEGTIGIALNLESNNVGVVLMGDGLMIQEGSSVKATGKIAQIPVSEAYLGRVINALANPIDGRGKISASESRLI : 130

 140 * 160 * 180 * 200 * 220 * 240 * 260
Isc : ESPAPGIISRRSVYEPLQTGLIAIDSMIPIGRGQRELIIGDRQTGKTAVATDTILNQQGQNVICVYVAIGQKASSVAQVVTTLQERGAMEYTIVVAETADSPATLQYLAPYTGAALAEYFMYRERHTSII : 237
Ici : ESPAPGIISRRSVYEPLQTGLIAIDSMIPIGRGQRELIIGDRQTGKTAVATDTILNQQGQNVICVYVAIGQKASSVAQVVTTLQERGAMEYTIVVAETADSPATLQYLAPYTGAALAEYFMYRERHTSII : 260
Ise : ESPAPGIISRRSVYEPLQTGLIAIDSMIPIGRGQRELIIGDRQTGKTAVATDTILNQQGQNVICVYVAIGQKASSVAQVVTTLQERGAMEYTIVVAETADSPATLQYLAPYTGAALAESFMYRERHTSII : 260
Ipe : ESPAPGIISRRSVYEPLQTGLIAIDSMIPIGRGQRELIIGDRQTGKTAVATDTILNQQGQNVICVYVAIGQKASSVAQVVTTLQERGAMEYTIVVAETADSPATLQYLAPYTGAALAEYFMYRERHTSII : 260
Ili : ESPAPGIILRRSVYEPLQTGLIAIDSMIPIGRGQRELIIGDRQTGKTAVATDTILNQQGQNVICVYVAIGQKASSVAQVVTTLQERGAMEYTIVVAETADSPATLQYLAPYTGAALAEYFMYRERHTSII : 259
Ikl : ESPAPGIISRRSVYEPLQTGLIAIDSMIPIGRGQRELIIGDRQTGKTAVATDTILNQQGQNVICVYVAIGQKASSVAQVVTTLQERGAMEYTIVVAETADSPATLQYLAPYTGAALAEYFMYRERHTSII : 259
Ath : ESPAPGIISRRSVYEPLQTGLIAIDSMIPIGRGQRELIIGDRQTGKTAVATDTILNQQGQNVICVYVAIGQKASSVAQVVTSLQERGAMEYTIVVAETADSPATLQYLAPYTGAALAEYFMYREQHTLII : 260

 * 280 * 300 * 320 * 340 * 360 * 380 *
Isc : YDDPSKQAQAYRQMSLLLRRPPGREAYPGDVFYLHSRLLERAAKSSSSLGEGSMTALPIVETQSGDVSAYIPTNVISITDGQIFLSADLFNAGIRPAINVGISVSRVGSAAQIKAMKQVAGKLKLELAQF : 367
Ici : YDDPSKQAQAYRQMSLLLRRPPGREAYPGDVFYLHSRLLERAAKSSSSLGEGSMTALPIVETQSGDVSAYIPTNVISITDGQIFLSADLFNAGIRPAINVGISVSRVGSAAQIKAMKQVAGKLKLELAQF : 390
Ise : YDDLSKQAQAYRQMSLLLRRPPGHEAYPGDVFYLHSRLLERAAKSSSSLGEGSMTALPIVETQSGDVSAYIPTNVISITDGQIFLSADLFNAGIRPAIN------RVGSAAQIKAMKQVAGKLKLELAQF : 384
Ipe : YDDLSKQAQAYRQMSLLLRRPPGREAYPGDVFYLHSRLLERAAKSSSSLGEGSMTALPIVETQSGDVSAYIPTNVISITDGQIFLSADLFNAGIRPAINVGISVSRVGSAAQIKAMKQVAGKLKLELAQF : 390
Ili : YDDLSKQAQAYRQMSLLLRRPPGREAYPGDVFYLHSRLLERAAKSSSSLGEGSMTALPIVETQSGDVSAYIPTNVISITDGQIFLSADLFNAGIRPAINVGISVSRVGSAAQIKAMKQVAGKFKLELA-F : 388
Ikl : YDDLSKQAQAYRQMSLLLRRPPGREAYPGDVFYLHSRLLERAAKSSSSLGEGSMTALPIVETQSGDVSAYIPTNVISITDGQIFLSADLFNAGSRPAINVGISVSRVGSAAQIKAMKQVAGKLKLELAQF : 389
Ath : YDDLSKQAQAYRQMSLLLRRPPGREAYPGDVFYLHSRLLERAAKLSSQLGEGSMTALPIVETQSGDVSAYIPTNVISITDGQIFLSADLFNAGIRPAINVGISVSRVGSAAQIKAMKQVAGKLKLELAQF : 390

400 * 420 * 440 * 460 * 480 * 500 *
Isc : AELE---AF-AQFASDLDKATQNQLARGQRLRELLKQSQSAPLAVEEQIMTIYTGTNGYLDSLEIGQVRKFLVELRTYLKSNKPEFKKIISSTKTFTEEAETILKEAIQEQMERFRLQEQL : 484
Ici : AELE---AF-AQFASDLDKTTQNQLARGQRLRELLKQSQSAPLAVEEQIMTIYTGTNGYLDSLEIGQVRKFLVELRTYLKSNKPEFKKIISSTKTFTEEAEAILKEAIQEQMERFRLQEQL : 507
Ise : AELE---AF-AQFASDLDKATQNQLARGQRLRELLKQSQSAPLAVEEQIMTIYTGTNGYLDSLEIGQVRKFLVELRTYLKSNKPEFKKIISSTKTFTEEAEAILKEAIQEQMERFRLQEQ- : 500
Ipe : AELE---AF-AQFASDLDKATQNQLARGQRLRELLKQSQSAPLAVEEQIMTIYTGTNGYLDSLDIGQVRKFLVELRTYLKSNKPEFKKIISSTKTFTEEAEAILKEAIQEQMERFRLQEQL : 507
Ili : IELEGTICFGSQFASDLDKATQNQLARGQRLRELLKQSQSAPLAVEEQIIPIYTGTNGYLDSLEIGQVRKFLVELRTYLKSNKPEFKKIISSTKTFT------------------------ : 485
Ikl : AELE---AF-AQFASDLDKATQNQLARGQRLRELLKQSQSAPLAVEEQIMTIYTGTNGYLDSLEIGQVRKFLVELRTYL------------------------------------------ : 464
Ath : AELE---AF-SQFSSDLDKATQNQLARGQRLRELLKQSQSAPLTVEEQIMTIYTGTNGYLDGLEIGQVRKFLVQLRTYLKTNKPQFQEIIASTKTLTAEAESFLKEGIQEQLERFLLQEKV : 507

**Figure S2 Multiple sequence alignment of the deduced amino acid sequences of ATPA protein.** The origins of the protein sequences used in this alignment are from: *Arabidopsis thaliana* (Ath), *Iodes klaineana* (Ikl), *Iodes cirrhosa* (Ici), *Iodes seretii* (Ise), *Iodes scandens* (Isc), *Iodes perrieri* (Ipe) and *Iodes liberica* (Ili). The amino acid numbers for each sequence are indicated on the right. In the sequence alignment, identical residues are shown with a black background, and similar residues are shown with a gray background.

* 20 * 40 * 60 * 80 * 100 * 120 *
Ikl : MGTNPTTSGSGIS---KKNLGRIAQIIGPVLDVAFAPGKMPNIYNALVVKGRDTAGQPINVTCEVQQLLGNNRVRAVAMSATDGLTRGMEVIDTGAPLSVPVGGVTLGRIFNVLGEPVDNLGPVDTRTTSPI : 129
Ili : MGTNPTTSGSGISTLEKKNLGRIAQIIGPVLDVTFAPVKMPNIYNALVVKGRDTAGQPINVTCEVQQLLGNNRVRAVAMSATDGLTRGMEVIDTGAPLSVPVGGVTLGRIFNVLGEPVDNLGPVDTRTTSPI : 132
Ise : MGTNPTTSGSGISTLDKKNLGRIAQIIGPVLDVAFAPGKMPNIYNALVVKGRDTAGQPINVTCEVQQLLGNNRVRAVAMSATDGLTRGMEVIDTGAPLSVPVGGVTLGRIFNVLGEPVDNLGPVDTRTTSPI : 132
Isc : MGTNPTTSGSGISTLDKKNLGRIAQIIGPVLDVAFAPGKMPNIYNALVVKGRDTAGQPINVTCEVQQLLGNNRVRAVAMSATDGLTRGMEVIDTGAPLSVPVGGATLGRIFNVLGEPVDNLGPVDTRTTSPI : 132
Ici : MGTNPTTSGSGISTLDKKNLGRIAQIIGPVLDVAFARGKMPNIYNALVVKGRDTAGQPINVTCEVQQLLGNNQVRAVAMSATDGLTRGMEVIDTGAPLSVPVGGATLGRIFNVLGEPVDNLGPVDTRTTSPI : 132
Ipe : --------------LDKKNLGRIAQIIGPVLDVAFAPGKMPNIYNALVVKGRDTAGQPINVTCEVQQLLGNNRVRAVAMSATEGLTRGMEVIDTGAPLSVPVGGVTLGRIFNVLGEPVDNLGPVDTRTTSPI : 118
Ath : MRTNPTTSNPEVSIREKKNLGRIAQIIGPVLDVAFPPGKMPNIYNALVVKGRDTLGQEINVTCEVQQLLGNNRVRAVAMSATEGLKRGMDVVDMGNPLSVPVGGATLGRIFNVLGEPVDNLGPVDTRTTSPI : 132

 140 * 160 * 180 * 200 * 220 * 240 * 260
Ikl : HRSAPAFIQLDTKLSIFETGIKVVDLLAPYRRGGKIGLFGGAGVGKTVLIMELINNIAKAHGGVSVFGGVGERTREGNDLYMEMKESGVINEQNIAESKVALVYGQMNEPPGARMRVGLTALTMAEYFRDVN : 261
Ili : HRSAPAFIQLDTKLSIFETGIKVVDLLAPYRRGGKIGLFGGAGVGKTVLIMELINNIAKAHGGVSVFGGVGERTREGNDLYMEMKESGVINEQNIAESKVALVYGQMNEPPGARMRVGLTALTMAEYFRDVN : 264
Ise : HRSAPAFIQLDTKLSIFETGIKVVDLLAPYRRGGKIGLFGGAGVGKTVLIMELINNIAKAHGGVSVFGGVGERTREGNDLYMEMKESGVINEQNIAESKVALVYGQMNEPPGARMRVGLTALTMAEYFRDVN : 264
Isc : HRSAPAFIQLDTKLSIFETGIKVVDLLAPYRRGGKIGLFGGAGVGKTVLIMELINNIAKAHGGVSVFGGVGERTREGNDLYMEMKESGVINEQNIAESKVALVYGQMNEPPGARMRVGLTALTMAEYFRDVN : 264
Ici : HRSAPAFIQLDTKLSIFETGIKVVDLLAPYRRGGKIGLFGGAGVGKTVLIMELINNIAKAHGGVSVFGGVGERTREGNDLYMEMKESGVINEQNIAESKVALVYGQMNEPPGARMRVGLTALTMAEYFRDVN : 264
Ipe : HRSAPTFIQLDTKLSIFETGIKVVDLLAPYRRGGKIGLFGGAGVGKTVLIMELINNIAKAHGGVSVFGGVGERTREGNDLYMEMKESGVINEQNIAESKVALVYGQMNEPPGARMRVGLTALTMAEYFRDVN : 250
Ath : HKSAPAFIELDTKLSIFETGIKVVDLLAPYRRGGKIGLFGGAGVGKTVLIMELINNIAKAHGGVSVFGGVGERTREGNDLYMEMKESGVINEQNLAESKVALVYGQMNEPPGARMRVGLTALTMAEYFRDVN : 264

 * 280 * 300 * 320 * 340 * 360 * 380 *
Ikl : EQDVLLFIDNIFRFVQAGSEVSALLGRMPSAVGYQPTLSTEMGSLQERITSTKDGSITSIQAVYVPADDLTDPAPATTFAHLDATTVLSRGLAAKGIYPAVDPLDSTSTMLQPRIVGEEHYETAQRVKQTLQ : 393
Ili : EQDVLLFIDNIFRFVQAGSEVSALLGRMPSAVGYQPTLSTEMGSLQERITSTKEGSITSIQAVYVPADDLTDPAPATTFAHLDATTVLSRGLAAKGIYPAVDPLDSTSTMLQPRIVGEEHYETAQRVKQTLQ : 396
Ise : EQDVLFFIDSIFRFVQAGSEVSALLGRMPSAVGYQPTLSTEMGSLQERITSTKEGSITSIQAVYVPADDLTDPAPATTFAHLDATTVLSRGLAAKGIYPAVDPLDSTSTMLQPRIVGEEHYETAQRVKQTLQ : 396
Isc : EQDVLLFIDNIFRFVQAGSEVSALLGRMPSAVGYQPTLSTEMGSLQERITSTKEGSITSIQAVYVPADDLTDPAPATTFAHLDATTVLSRGLAAKGIYPAVDPLDSTSTMLQPRIVGEEHYETAQRVKQTLQ : 396
Ici : EQDVLLFIDNIFRFVQAGSEVSALLGRMPSAVGYQPTLSTEMGSLQERITSTKEGSITSIQAVYVPADDLTDPAPATTFAHLDATTVLSRGLAAKGIYPAVDPLDSTSTMLQPRIVGEEHYETAQRVKQTLQ : 396
Ipe : EQDVLLFIDNIFRFVQAGSEVSALLGRMPSAVGYQPTLSTEMGSLQERITSTKEGSITSIQAVYVPADDLTDPAPATTFAHLDATTVLSRGLAAKGIYPAVDPLDSTSTMLQPRIVGEEHYEMAQRVKQTLQ : 382
Ath : EQDVLLFIDNIFRFVQAGSEVSALLGRMPSAVGYQPTLSTEMGTLQERITSTKKGSITSIQAVYVPADDLTDPAPATTFAHLDATTVLSRGLAAKGIYPAVDPLDSTSTMLQPRIVGEEHYETAQQVKQTLQ : 396

400 * 420 * 440 * 460 * 480 *
Ikl : RYKELQDIIAILGLDELSEEDRLTVARARKIERFLSQPFFVAEVFTGSPGKYVGLTETIRGFQLILSGELDGLPEQAFYLVGNIDEATAKAMNLEMESNLKK : 495
Ili : RYKELQEIIAILGLDELSEEDRLTVARARKIERFLSQPFFVAEVFTGSPGKYVGLTETIRGFQLILSGELDGLPEQAFYLVGNIDEATAKAMNLEMESNLKK : 498
Ise : RYKELQDIIAILGLDELSEEDRLTVARARKIERFLSQPFFVAEVFTGSPGKYVGLTETIRGFQLILSGELDGLPEQAFYLVGNIDEATAKAMNLEMESNLKK : 498
Isc : RYKELQDIIAILGLDELSEEDRLTVARARKIERFLSQPFFVAEVFTGSPGKYVGLTETIRGFQLILSGELDGLPEQAFYLVGNIDEATAKAMNLEMESNLKK : 498
Ici : RYKELQDIIAILGLDELSEEDRLTVARARKIERFLSQPFFVAEVFTGSPGKYVGLTETIRGFQLILSGELDGLPEQAFYLVGNIDEATAKAMNLEMESNLKK : 498
Ipe : RYKELQDIIAILGLDELSEEDRLTVARARKIERFLSQPFFVAEVFTGSPGKYVGLTETIRGFQLILSGELDGLPEQAFYLVGNIDEATAKAINLEMESNLKK : 484
Ath : RYKELQDIIAILGLDELSEEDRLTVARARKIERFLSQPFFVAEVFTGSPGKYVGLAETIRGFNLILSGEFDSLPEQAFYLVGNIDEATAKATNLEMESKLKK : 498

**Figure S3 Multiple sequence alignment of the deduced amino acid sequences of ATPB protein.** The origins of the protein sequences used in this alignment are from: *Arabidopsis thaliana* (Ath), *Iodes klaineana* (Ikl), *Iodes cirrhosa* (Ici), *Iodes seretii* (Ise), *Iodes scandens* (Isc), *Iodes perrieri* (Ipe) and *Iodes liberica* (Ili). The amino acid numbers for each sequence are indicated on the right. In the sequence alignment, identical residues are shown with a black background, and similar residues are shown with a gray background.

* 20 * 40 * 60 * 80 * 100 * 120 *
Ikl : MTLNLCVLTPNRIVWDSEVNEIILSTNNGQIGVLPNHAPIATAVDIGILKKRLNGQWLAMALMGGFARIGKNEITILVNDAEKSSDIDPQEAQKTLEIAEANLRKAEGKRQTIEANLALRRARTRVEAINMI : 132
Ili : MTLNLCVLTPNRIVWDSEVNEIILSTNNGQIGVLPNHAPIATAVDIGILKKRLNGQWLAMALMGGFARIGKNEITILVNDAEKSSDIDPQEAQKTLEIAEANLRKAEGKRQTIEANLALRRARTRVEAINMI : 132
Ise : MTLNLCVLTPNRIVWDSEVNEIILSTNNGQIGVLPNHAPIATAVDIGILKIRLKGQWLAMALMGGFARIGKNEITILVNDAEKSSDIDPQEAQKTLEIAEANLRKAEGKRQTIEANLALRRARTRVEAINMI : 132
Ipe : MTLNLCVLTPNRIVWDSEVNEIILSTNNGQIGVLPNHAPIATAVDIGIFKIRLNDQWLAMALMGGFARIGKNEITILVNDAEKSSNIDPQEAQKTLEIAEANLRKAEGKRQTIEANLALRRARTRVEAINMI : 132
Ici : MTLNLCVLTPNRIVWDSEVKEIILSTNNGQIGVLPNHAPIATAVDIGILKIRLNDQWLAMALMGGFARIGKNEITILVNDAEKSSDIDPQEAQKTLEIAEANLRKAEGKRQTIEANLSLRRARTRVEAINMI : 132
Isc : MTLNLCVLTPNRIVWDSEVKEIILSTNNGQIGVLPNHAPIATAVDIGILKIRLNDQWLAMALMGGFARIGKNEITILVNDAEKSSDIDPQEAQKTLEIAEANLRKAEGKRQTIEANLALRRARTRVEAI--- : 129
Ath : MTLNLCVLTPNRIVWDSEVKEIILSTNSGQIGVLANHAPIATAVDIGILKIRLANQWLTMALMGGFARIGNNEITILVNDAEKNSDIDPQEAQQTLEIAEANLRKAEGKRQTIEANLALRRARTRVEALNTI : 132

Ikl : S : 133
Ili : S : 133
Ise : S : 133
Ipe : S : 133
Ici : S : 133
Isc : - : -
Ath : - : -

**Figure S4 Multiple sequence alignment of the deduced amino acid sequences of ATPE protein.** The origins of the protein sequences used in this alignment are from: *Arabidopsis thaliana* (Ath), *Iodes klaineana* (Ikl), *Iodes cirrhosa* (Ici), *Iodes seretii* (Ise), *Iodes scandens* (Isc), *Iodes perrieri* (Ipe) and *Iodes liberica* (Ili). The amino acid numbers for each sequence are indicated on the right. In the sequence alignment, identical residues are shown with a black background, and similar residues are shown with a gray background.

* 20 * 40 * 60 * 80 * 100 * 120 *
Ikl : MKTVTDSFVSLGYWPSAGSFGFNTDILATNPINLSVVLGVLIFFGKGVCASCL--RKQRILNTIQNSAELRGRAIEQLEKARARLRKVEMEADQFRVNGYSEIEREKRNLINSTYKALEQFENYKNETIRFE : 130
Ili : MKTVTDSFVSLGYWPSAGSFGFNTDILATNPINLSVVLGVLIFFGKGVCASCL--RKQRILNTIQNSAELRGRAIEQLEKARTRLRKVEMEADQFRVNGYSEIEREKRNLINSTYKALEQFENYKNETIRFE : 130
Ath : MKNLTDSFVYLGHWPSAGSFGFNTDILATNPINLSVVFGVLIFFGKGVLNDLLDNRKQRILNTIRNSEELREGAIQQLENARARLRNVETEADKFRVNGYSEIEREKLNLINSTYKTLKQLENYKNETILFE : 132
Ici : MKTVTDSFVSLGYWPSAGSFGFNTDILATNPINLSVVLGVLIFFGKGVLSDLLDNRKQRILNTIQNSAELRGRAIEQLEKARARLRKVEMEADQFRVNGYSEIEREKRNLINSTYKALEQFENYKNETIRFE : 132
Ipe : MKTVTDSFVSLGYWPSAGSFGFNTDILATNPINLSVVLGVLIFFGKGVCASCL--RKQRILNTIQNSAELRGRAIEQLEKARARLRKVEMEADQFRVNGYSEIEREKQNLINSTYKALEQFENYKNETIRFE : 130
Ise : MKTVTDSFVSLGSWPSAGSFGFNTDILATNPINLSVVLGVLIFFGKGVCASCL--RKQRILNTIQNSAELRGRAIEQLEKARARLRKVEMEADQFRVNGYSEIEREKGNLINSTYKALEQFENYKNETIRFE : 130
Isc : MKTVTDSFLSLGYWPSAGSFGFNTDILATNPINLSVVLGVLIFFGKGVCASCL-----------QNSAELRGRAIEQLEKARARL---------------AELRG----------RAIEQLE---------- : 86

 140 * 160 * 180
Ikl : QQRAINQVRQRVFQQALQGALGTLNSCLNNELHLRTISANIGMLGSMKEITD : 182
Ili : QQRAINQVRQRVFQQALQGALGTLNSCLNNELHLRTISANIGMLGSMKEITD : 182
Ath : QQRTINQVRERVFQQALQGAIGTLNSCLSNELHLRTINANIGMFGTMKEITD : 184
Ici : QQRAINQVRQRVFQQALQAALGTLNSCLNNELHLRTISANIGMLRSMKEITD : 184
Ipe : QQRAINQVRQRVFQQALQGALGTLNSCLNNELHLRTISANIGMLGSMKEITD : 182
Ise : QQRAINQVRQRVFQQALQGALGTLNSCLNNELHLRTISANIGMLGSMKEITD : 182
Isc : ------KARARLLQLLM----------------------------------- : 97

**Figure S5 Multiple sequence alignment of the deduced amino acid sequences of ATPF protein.** The origins of the protein sequences used in this alignment are from: *Arabidopsis thaliana* (Ath), *Iodes klaineana* (Ikl), *Iodes cirrhosa* (Ici), *Iodes seretii* (Ise), *Iodes scandens* (Isc), *Iodes perrieri* (Ipe) and *Iodes liberica* (Ili). The amino acid numbers for each sequence are indicated on the right. In the sequence alignment, identical residues are shown with a black background, and similar residues are shown with a gray background.

* 20 * 40 * 60 * 80
Ikl : MNPLISAASVIAAGLAVGLASIGPGVGQGTAAGQAVEGIARQPEAEGKIRGTLLLSLAFMEALTIYGLVV----------- : 70
Ipe : MNPLISAASVIAAGLAVGLASIGPGVGQGTAAGQAVEGIARQPEAEGKIRGTLLLSLAFMEALTIYGLVVALALLFANPFV : 81
Isc : MNPLISAASVIAAGLAVGLASIGPGVGQGTAAGQAVEGIARQPEAEGKIRGTLLLSLAFMEALTIYGLVVALALLFANPFV : 81
Ise : MNPLISAASVIAAGLAVGLASIGPGVGQGTAAGQAVEGIARQPEAEGKIRGTLLLSLAFMEALTIYGLVVALALLFANPFV : 81
Ili : MNPLISAASVIAAGLAVGLASIGPGVGQGTAAGQAVEGIARQPEAEGKIRGTLLLSLAFMEALTIYGLVVALALLFANPFV : 81
Ici : MNPLISAASVIAAGLAVGLASIGPGVGQGTAAGQAVEGIARQPEAEGKIRGTLLLSLAFMEALTIYGLVVALALLFANPFV : 81
Ath : MNPLVSAASVIAAGLAVGLASIGPGVGQGTAAGQAVEGIARQPEAEGKIRGTLLLSLAFMEALTIYGLVVALALLFANPFV : 81

**Figure S6 Multiple sequence alignment of the deduced amino acid sequences of ATPH protein.** The origins of the protein sequences used in this alignment are from: *Arabidopsis thaliana* (Ath), *Iodes klaineana* (Ikl), *Iodes cirrhosa* (Ici), *Iodes seretii* (Ise), *Iodes scandens* (Isc), *Iodes perrieri* (Ipe) and *Iodes liberica* (Ili). The amino acid numbers for each sequence are indicated on the right. In the sequence alignment, identical residues are shown with a black background, and similar residues are shown with a gray background.

* 20 * 40 * 60 * 80 * 100 * 120 *
Isc : MNVLSCSINTLK--GLYDISGVEVGQHLDWQIGGFKIHGQVLITSWVVIAILLGSATIAARNPQTIPTRAQNFFEYVLEFIRDVSKTQIGEEYGPWVSFIGTMFLFIFVSNWSGALLPWKIIQLPHGELAAP : 130
Ici : MNVLSCSINTLK--GLYDISGVEVGQHLYWQIGGFKIHGQVLITSWVVIAILLGSATIAVRNPQTIPTRAQNFFEYVLEFIRDVSKTQIGEEYGPWVSFIGTMFLFIFVSNWSGALLPWKIIQLPHGELAAP : 130
Ipe : MNVLSCSINTLK--GLYDISGVEVGQHLYWEIGGFKIHGQVLITSWVVIAILLGSATIAVRNPQTIPTRGQNFFEYVLEFIRDVSKTQIGEEYGPWVSFIGTMFLFIFVSNWSGALLPWKIIQLPHGELAAP : 130
Ili : MNVLSCSINTLK--GLYDISGVEVGQHLYWEIGGFKIHGQVLITSWVVIAILLGSATIAVRNPQTIPTRGQNFFEYVLEFIRDVSKTQIGEEYGPWVSFIGTMFLFIFVSNWSGALLPWKIIQLPHGELAAP : 130
Ikl : MNVLSCSINTLK--GLYDISGVEVGQHLYWEIGGFKIHGQVLITSWVVIAILLGSATIAVRNPQTIPTRGQNFFEYVLEFIRDVSKTQIGEEYGPWVSFIGTMFLFIFVSNWSGALLPWKIIQLPHGELAAP : 130
Ise : MNVLSCSINTLK--GLYDISVVEVGQHLYWEIGGFKIHGQVLITSWVVIAILLGSATIAVRNPQTIPTRGQNFFEYVLEFIRDVSKTQIGEEYGPWVSFIGTMFLFIFVSNWSGALLPWKIIQLPHGELAAP : 130
Ath : MNVLSCSINTLIKEGLYEISGVEVGQHFYWQIGGFQVHAQVLITSWVVIAILLGSAVLAIRNPQTIPTDGQNFFEFVLEFIRDVSKTQIGEEYGPWVPFIGTLFLFIFVSNWSGALLPWKIIQLPQGELAAP : 132

 140 * 160 * 180 * 200 * 220 * 240
Isc : TNDINTTVALSLLTSVAYFYAGLSKKGLGYFGKYIQPTPILLPINILEDFTKPLSLSFRLFGNILADELVVVVLVSLVPLVVPIPVMFLGLFTSGIQALIFATLAAAYIGESMEGHH : 247
Ici : TNDINTTVALALLTSVAYFYAGLSKKGLGYFGKYIQPTPILLPINILEDFTKPLSLSFRLFGNILADELVVVVLVSLVPLVVPIPVMFLGLFTSGIQALIFATLAAAYIGESMEGHH : 247
Ipe : TNDINTTVALALLTSVAYFYAGLSKKGLGYFGKYIQPTPILLPINILEDFTKPLSLSFRLFGNILADELVVVVLVSLVPLVVPIPVMFLGLFTSGIQALIFATLAAAYIGESMEGHH : 247
Ili : TNDINTTVALALLTSVAYFYAGLSKKGLGYFGKYIQPTPILLPINILD-FTKPLSLSFRLFGNILADELVVVVLVSLVPLVVPIPVMFLGLFTSGIQALIFATLAAAYIGESMEGHH : 246
Ikl : TNDINTTVALALLTSVAYFYAGLSKKGLGYFGKYIQPTPILLPINILEDFTKPLSLSFRLFGNILADELVVVVLVSLVPLVVPIPVMFLGLFTSGIQALIFATLAAAYIGESMEGHH : 247
Ise : TNDINTTVALALLTSVAYFYAGLSKKGLGYFGKYIQPTPILLPINILEDFTKPLSLSFRLFGNILADELVVLVLVSLVPLVVPIPVMFLGLFTSGIQALIFATLAAAYIGESMEGHH : 247
Ath : TNDINTTVALALLTSVAYFYAGLSKKGLGYFSKYIQPTPILLPINILEDFTKPLSLSFRLFGNILADELVVVVLVSLVPLVVPIPVMFLGLFTSGIQALIFATLAAAYIGESMEGHH : 249

**Figure S7 Multiple sequence alignment of the deduced amino acid sequences of ATPI protein.** The origins of the protein sequences used in this alignment are from: *Arabidopsis thaliana* (Ath), *Iodes klaineana* (Ikl), *Iodes cirrhosa* (Ici), *Iodes seretii* (Ise), *Iodes scandens* (Isc), *Iodes perrieri* (Ipe) and *Iodes liberica* (Ili). The amino acid numbers for each sequence are indicated on the right. In the sequence alignment, identical residues are shown with a black background, and similar residues are shown with a gray background.

* 20 * 40 * 60 * 80 * 100 * 120 *
Ipe : MIFLTLEHILTHISFSIVSIVITIHFITLFV--DEIVRLYDSSEKGMIAVFSCITGLLVTRWIYSGHFPLSDLYESLIFLSWSFSIIHLIPY---------------LKKNKTN-LRTITAPSAIFSQGFTT : 114
Ili : MIFLTLEHILTHISFSIVSIVITIHFITLFV--DEIVRLYDSSEKGMIAVFSCITGLLVTRWIYSGHFPLSDLYESLIFLSWSFSIIHLIPY---------------FKKNKKN-LRTITAPSAIFSQGFTT : 114
Isc : MIFFTLEHILT---FSIVSVVITIHFITLFV--DEIVRLYDSSEKGMIAVFSCITGLLVTRCIYSGHFPLSDLYESLIFLSWSFSIIHLIPY---------------FKKNKKD-LRTITAPSAIFSQGFTT : 111
Ise : ----------------------------LFV--DEIVRLYDSSEKGMIAVFSCITGLLVTRWIYSGHFPLSDLYESLIFLSWSFSIIHLIPYRSEEHTSSIIHLIPYFKKNKKN-LRTITAPSAIFSQGFTT : 101
Ici : MIFLTLEHILTHISFSIVSIVITIHFITLFV--DEIVRLYDSSEKGMIAVFSCITGLLVTRWIYSGHFPLSDLYESLIFLSWSFSIIHLIPY---------------FKKNKTN-LRPITVPSAIFSQGFTT : 114
Ikl : MIFLTLEHILTHISFSIVSIVITIHFITLFV--DEIVRLYDSSEKGMIAVFSCITGLLVTRWIYSGHFPLSDLYESLIFLSWSFSIIHLIPY---------------FKKNKTN-LRTITAPSAIFSQGFTT : 114
Ath : MIFSILEHILTHISFSVVSIVLTIYFLTLLVNLDEIIGFFDSSDKGIIITFFGITGLLLTRWIYSGHFPLSNLYESLIFLSWAFSIIHMVSY---------------FNKKQQNKLNTITAPSVIFIQGFAT : 117

 140 * 160 * 180 * 200 * 220 * 240 * 260
Ipe : SGLLTEIHQSTILVPALQSEWLIMHVSMMILGYAALLCGSLLSVALLVITFRKNISIFSKS----HNLLNKPFFFGEIQY--MNKKRNVLR-----NTSLFS--SKNYYQYQLIQQLDYWSYRVISLGFFFL : 233
Ili : SGLLTEIHQSTILVPALQSEWLIMHVSMMILGYAALLCGSLLSVALLVITFRKNLSIFSKS----HNLLNKPFFFDEIQY--MNKKRNVLRNTSLFNTSLFS--SKNYYQYQLIQQLDYWSYRVISLGFIFL : 238
Isc : SGLLTEINQSTILVPALQSEWLIMHVSMMILGYAALLCGSLLSVALLVITFRKNRKNYSKS----YNLLNKPFFFGEIQY--MNKKRNVLR-----NTSLFS--AKNYYQSQLIQQLDYWSYRVISLGFIFL : 230
Ise : SGLLTEIHQSTILVPALQSEWLIMHVSMMILGYAALLCGSLLSVALLVITFRKNRKNFSKS----HNLLNKPFFFGEIQYQYMNKKRNVLQ-----NTSLFS--SKNYYQ---------WSYRVISLGFIFL : 213
Ici : SGLLTEIHQSTILVPALQSEWLIMHVSMMILGYASLLCGSLLSVALLVITFRKNRKNYSKS----HNLLNKPFFFGEIQY--MNKKRNVLR-----NTSLFSFSAKNYYQSQLIQQLDYWSYRVISLGFIFL : 235
Ikl : SGLLTEIHQSTILVPALQSEWLIMHVSMMILGYAALLCGSLLSVALLVITFRKNRKIFSKS----HNLLNKPFFFDEIQY--MNKKRNVLR-----NTSLFS--SKNYYQYQLIQQLDYWSYRVISLGFIFL : 233
Ath : SGLLNKMPQSAILVPALQSQWLMMHVSMMILGYGALLCGSLLSIALLVITFRKVGPTFWKKNIKKNFLLNELFSFDVLYY--INERNSILLQ----QNINFS-FSRNYYRYQLIQQLDFWSFRIISLGFIFL : 242

 * 280 * 300 * 320 * 340 *
Ipe : TIGILSGAVWANEAWGSYWNWDPKETWAFITWIIFAIYLHTRINKNLQGVNSAIVPSIGFLIIWICYFGVNLLGIGLHSYGSFTLTSN : 321
Ili : TIGILSGAVWANEAWGSYWNWDPKETWAFITWIIFAIYLHTRINKNLQGVNSAIVPSIGFLII------------------------- : 301
Isc : TIGILSGAVWANEAWGSYWNWDPKETWAFITWIIFAIYLHTRINKNLQGVNSAIVPSIGFLIIWICYFGVNLLGIGLHSYGSFTLMSN : 318
Ise : TIGILSGAVWANEAWGSYWNWDPKETWAFITWIIFAIYLHTRINKNLQGVNSAIVPSIGFLIIWICYFGVNLLGIGLHSYGSFTLTSN : 301
Ici : TIGILSGAVWANEAWGSYWNWDPKETWAFITWIIFAIYLHTRINKNLQGVNSAIVPSIGFLIIWICYFGVNLVGIGLHSYGSFTLMSN : 323
Ikl : TIGILSGAVWANEAWGSYWNWDPKETWAFITWIIFAIYLHTRINKNLQGENSAIVPSIGFLIIWICYFGVNLLGIGLHSYGSFTLTSN : 321
Ath : TVGILSGAVWANETWGSYWNWDPKETWAFITWTIFAIYLHIKTNRNVRGINSAIVALIGFILIWICYFGVNLLGIGLHSYGSFTSN-- : 328

**Figure S8 Multiple sequence alignment of the deduced amino acid sequences of CCSA protein.** The origins of the protein sequences used in this alignment are from: *Arabidopsis thaliana* (Ath), *Iodes klaineana* (Ikl), *Iodes cirrhosa* (Ici), *Iodes seretii* (Ise), *Iodes scandens* (Isc), *Iodes perrieri* (Ipe) and *Iodes liberica* (Ili). The amino acid numbers for each sequence are indicated on the right. In the sequence alignment, identical residues are shown with a black background, and similar residues are shown with a gray background.

* 20 * 40 * 60 * 80 * 100 * 120 *
Isc : ---------------------------------------------------------------------------------------IHKEMIQLIKIYNEDHINTILHFSTNIICFVILSGYSILGNDELV : 45
Ici : MAKKKAFTPLLYLASIVFLPWCISLPFNKSFESWVTNWWDTRQSEILLNDIQEKSILEKFIELEELLLLEEIIKEYSETDRQKFRIGIHKEMIQLIKIYNEDHINTILHFSTNIICFVILSGYSILGNDELV : 132
Ikl : MAKKKAFTPLLYLASIVFLPWCISLPFNKSFESWVTNYTR--QSEILLNDIQEKSILEKFIELEELLLLDEIIKEYSETDRQKFRIGIHKEMIQLIKIYNEDHINTILHFSTNIICFIILSAYSILGNDELV : 130
Ipe : MEKKKAFIPLLYLASIVFLPWCISLPFNKSFESWVPNYTR--QSEILLNDIQEKSILEKFIELEELLLLDEIIKEYSETDRQKFRIGIHKEMIQLIKIYNEDHINTILHFSTNIICFIILSAYSILGNDELV : 130
Ili : --------------------------------SWVTNYTR--QSEILLNDIQEKSILEKFIELEELLLLDEIIKEYSETDRQKFRIGIHKEMIQLIKIYNEDHINTILHFSTNIICFIILSAYSILGNDELV : 98
Ise : ------------------------------------------------------------------------------------------------------------------------------GNEELV : 6
Ath : MAKKKAFIPFFYFLSIVFLPWLISLCCNKSLKTWITNWWNTRQCETFLNDIQEKSFLEKFIQLEELFQLDEMIKEYPETNLQQFRLGIHKETIQFIKIHNEYNIHTILHFSTNLISFVILSGYSFWGKEKLF : 132

 140 * 160 * 180 * 200 * 220
Isc : LLNSWVQEFLYNLSDTIKAFSILLLTDLCIGFHSP-------------------------------------------------------------- : 80
Ici : LLNSWVQEFLYNLSDTIKAFSILLLTDLCIGFHSPHGWELMIGSVYKDFGFVHNDQIISGLVSTFPVILDTILKYWIFRYLNRVSPSLVVIYHSMNE : 229
Ikl : LLNSWVQEFLYNLSDTIKAFSILLLTDLCIGFHSPHGWELMIGSVYKDFGFVHNDQIISGLVSTFPVILDTILKYWIFRYLNRVSPS---------- : 217
Ipe : LLNSWVQEFLYNLSDTIKAFSILLLTDLCIGFHSPHGWELMIGSVYKDFGFVHNDQIISGLVSTFPVILDTILKYWIFRYLNRVSPSLVVIYHSM-- : 225
Ili : LLNSWVQEFLYNLSDTIKAFSILLLTDFFIGFHSPHGWELMIGSVYKDFGFVHNDQIISGLVSTFPVILDTILKYWIFRYLNRVSPSLVVIYHSMNE : 195
Ise : LLNSWVQEFLYNLSDTIKAFSILLLTDLCIGFHSPHGWELMIGSVYKDFGFVHNDQIISGLVSTFPVILDTILKYWIFRYLNRVSPSLVVI------ : 97
Ath : ILNSWVQEFLYNLSDTIKAFSILLLTDLCIGFHSPHGWELMIGYIYKDFGFAHYEQILSGLVSTFPVILDTIFKYWIFRYLNRVSPSLVVIYHAIND : 229

**Figure S9 Multiple sequence alignment of the deduced amino acid sequences of CEMA protein.** The origins of the protein sequences used in this alignment are from: *Arabidopsis thaliana* (Ath), *Iodes klaineana* (Ikl), *Iodes cirrhosa* (Ici), *Iodes seretii* (Ise), *Iodes scandens* (Isc), *Iodes perrieri* (Ipe) and *Iodes liberica* (Ili). The amino acid numbers for each sequence are indicated on the right. In the sequence alignment, identical residues are shown with a black background, and similar residues are shown with a gray background.

* 20 * 40 * 60 * 80 * 100 * 120 *
Ikl : MPVGVPKVPFRSPGEEDASWVDIN-RLYRERLLFLGQAVD-----SEISNQLIGLMVYLSIEDDTKDLHLFINSPGGWVIPGVGIYDTMQFVRPDVHTICMGLAASMGSFILVGGEITKRLAFPHARVMIHQ : 126
Ili : MPVGVPKVPFRSPGEEDASWVDI--RLYRERLLFLGQAVD-----SEISNQLIGLMVYLSIEDDTKDLHLFINSPGGWVIPGVGIYDTMQFVRPDVHTICMGLAASMGSFILVGGEITKRLAFPHARVMIHQ : 125
Ipe : MPVGVPKVPFRSPGEEDASWVDIN-RLYRERLLFLGQAVD-----SEISNQLIGLMVYLSIEDDTKDLHLFINSPGGWVIPGVGIYDTMQFVRPDVHTICMGLAASMGSFILVGGEITKRLAFPHARVMIHQ : 126
Ise : MPVGVPKVPFRSPGEEDASWVDIN-RLYRERLLFLGQAVDSEAVDSEISNQLIGLMVYLSIEDDTKDLHLFINSPGGWVIPGVGIYDTMQFVRPDVHTICMGLAASMGSFILVGGEITKRLAFPHARVMIHQ : 131
Ici : MPVGVPKVPFRSPGEEDASWVDIN-RLYRERLLFLGQAVD-----SEISNQLIGLMVYLSIEDDTKDLHLFINSPGGWVIPGVGIYDTMQFVRPDVHTICMGLAASMGSFILVGGEITKRLAFPHARVMIHQ : 126
Isc : MPVGVPKVPFRSPGEEDASWVDIN-RLYRERLLFLGQAVD-----SEISNQLIGLMVYLSIEDDTKDLHLFINSPGGWVIPGVGIYDTMQFVRPDVHTICMGLAASMGSFILVGGEITKRLAFPHARVMIHQ : 126
Ath : MPIGVPKVPFRSPGEGDTSWVDIYNRLYRERLFFLGQEVD-----TEISNQLISLMIYLSIEKDTKDLYLFINSPGGWVISGMAIYDTMQFVRPDVQTICMGLAASIASFILVGGAITKRIAFPHARVMIHQ : 127

 140 * 160 * 180 * 200
Ikl : PASSFYESQTGEFILEAEELLKLRETLTRVYVQRTGKPLWVVSEDMERDVFMSATEAQAYGIVDLVAVE : 195
Ili : PASSFYESQTGEFILEAEELLKLRETLTRVYVQRTGKPLWVVSEDMERDVFMSATEAQAYGIVDLVAVE : 194
Ipe : PASSFYESQTGEFILEAEELLKLRETLTRVYVQRTGKPLWLVSEDMERDVFMSSTEAQAYGIVDLVAVE : 195
Ise : PASSF-ESQTGEFILEAEELLKLRETLTRVYVQRTGKPLWLVSEDMERDVFMSATEAQAYGIVDLVAVE : 199
Ici : PASSFYESQTGEFILEAEELLKLRETLTRVYVQRTGKPLWLVSEDMERDVFMSATEAQAYGIVDLVAVE : 195
Isc : PASSFYESQTGEFILEAEELLKLRETLTRVYVQRTGKPLWLVSEDMERDVFMSATEAQAYGIVDLVA-- : 193
Ath : PASSFYEAQTGEFILEAEELLKLRETITRVYVQRTGKPIWVISEDMERDVFMSATEAQAHGIVDLVAVQ : 196

**Figure S10 Multiple sequence alignment of the deduced amino acid sequences of CLPP protein.** The origins of the protein sequences used in this alignment are from: *Arabidopsis thaliana* (Ath), *Iodes klaineana* (Ikl), *Iodes cirrhosa* (Ici), *Iodes seretii* (Ise), *Iodes scandens* (Isc), *Iodes perrieri* (Ipe) and *Iodes liberica* (Ili). The amino acid numbers for each sequence are indicated on the right. In the sequence alignment, identical residues are shown with a black background, and similar residues are shown with a gray background.

* 20 * 40 * 60 *
Ili : MKEQKWIHEGLITESLPNGMFRVRLNNEDPILGYVSGKIRRSFIRILPGDRVKIEVSRYDSTRGRIIYRLRNKDSKD : 77
Ici : MKEQKWIHEGLITESLPNGMFRVRLNNEDPILGYVSGKIRRSFIRILPGDRVKIEVSRYDSTRGRIIYRLRNKDSKD : 77
Isc : MKEQKWIHEGLITESLPNGMFRVRLNNEDPILGYVSGKIRRSFIRILPGDRVKIEVSRYDSTRGRIIYRLRNKDSKD : 77
Ikl : MKEQKWIHEGLITESLPNGMFRVRLNNEDPILGYVSGKIRRSFIRILPGDRVKIEVSRYDSTRGRIIYRLRNKDSKD : 77
Ise : MKEQKWIHEGLITESLPNGMFRVRLNNEDPILGYVSGKIRRSFIRILPGD--------------------------- : 50
Ipe : MKEQKWIHEGLITESLPNGMFRVRLNNEDPILGYVSGKIRRSFIRILPGDKVKIEVSRYDSTRGRIIYRLRNKDSKD : 77

**Figure S11 Multiple sequence alignment of the deduced amino acid sequences of INFA protein.** The origins of the protein sequences used in this alignment are from: *Arabidopsis thaliana* (Ath), *Iodes klaineana* (Ikl), *Iodes cirrhosa* (Ici), *Iodes seretii* (Ise), *Iodes scandens* (Isc), *Iodes perrieri* (Ipe) and *Iodes liberica* (Ili). The amino acid numbers for each sequence are indicated on the right. In the sequence alignment, identical residues are shown with a black background, and similar residues are shown with a gray background.

* 20 * 40 * 60 * 80 * 100 * 120 *
Ikl : MEEFKKYLRIDKSQQHDFLYPLIFQESTYILAHDHGL-----NRSILLENGGYGKKFSLLIVKRLITRMYQQNHLIPFANDSNQNLFFGRNKNLYSQIISEGFAVIVEIPFSLRLTSFLEGKDIIQSHTL : 125
Ili : MEEFKKYLRIDKSQQHDFLYPLIFQESTYILAHDHGL-----NRSILLENGGYGKKSSLLIVKRLITRMYQQNHLIPFANDSNQNLFFGRNKNLYSQIISEGFAVIVEIPFSL----------------- : 108
Isc : MEEFKKYLRIDRSQQHDFLYPLIFQESTY----------------------------------------------------------------------------------------------------- : 29
Ici : MEEFKKKLRIDRFQQHDFLYPLIFQESTYILAHNHGL-----NRSILLENRGYDNKSSLLIVKRLITRMYQQNHLIPFANDSNQNQFFGRNKNLYSKIISEGFAVIVEIPFSLRLTSFLEGKDIIQSHTL : 125
Ipe : MEEFKKYLRIDKSQQHDFLYPLIFQESTYILAHDHGL-----NRSILLENRGYEKKSSLLIVKRLITRMYQQNHLIPFANDSNQNLFFGRNKNLYLQIISEGFAVIVEIPFSLRLTSFLEGKDIIQSHTL : 125
Ise : MEEFKKYLRIDKSQQHDFLYPLIFQESTYILAHDHGL-----NRSILLENRGYDKKSS-------------QNHLIPFANDSNQNQFFGRNKNLYSQIISEGFAVIVEIPFSLRLTSFLEGKDIIQSHTL : 112
Ath : MDKFQGYLEFDGARQQSFLYPLFFREYIYVLAYDHGLNRLNRNRYIFLENADYDKKYSSLITKRLILRMYEQNRLIIPTKDVNQNSFLGHTSLFYYQMISVLFAVIVEIPFSLRLGSSFQGKQLKKSYNL : 130

 140 * 160 * 180 * 200 * 220 * 240 * 260
Ikl : RSIHSIFPFLEDNFLHLNYALDILIPHPIHLEILVQTIRYWVKDASSLHLLRFFLHEYGNLTSTITSKKVSYSFSKGNHRFFFFLYNSYICEYESVFVFLCNQSSHLRSTSSGAFIERIYFYLKMECVVE : 255
Ili : ---------------------------------------------------------------------------------------------------------------------------------- : -
Isc : --------------------------------------------------------EYGNLNSPITSKKVSYSFSKRNHRFFFFLYNSYVCEYESVFVFLCNQSS------------------------- : 78
Ici : RSIHSIFPFLEDNFLHLNYALDIRIPHPIHLEILVQTIRYWVKDASSLHLLRFFLHEYGDLNSPITSKKVSYSFSKRNHRFFFFLYNSYVCEYESVFVFLCNQSSHLRSTSSGTFIERIYFYVKIECVVE : 255
Ipe : RSIHSIFPFLEDNFLHLNYALDILIPYPIHLEILVQTIRYWVKDASSLHLLRFFLHEYGNLASTITSKKVSYSFSKG--RFFFFLYNSYVCEYESVFVFLCNQSSHLRSTSSGAFIERIYFYLKMECVVE : 253
Ise : RSIHSIFPFLEDNFLHLNYALDILIPHPIHLEILVQTIRYWVKDASSLHLLRFFLHEYGNLTSTIS---------KGNQRFFFFLYNSYVCQYESVFVFLCNQS-------------------------- : 207
Ath : QSIHSIFPFLEDKLGHFNYVLDVLIPYPIHLEILVQTLRYRVKDASSLHFFRFCLYEYCNWKNFYIKKK-----SILNPRFFLFLYNSHVCEYESIFFFLRKRSSHLRSTSYEVLFERIVFYGKIHHFFK : 255

 * 280 * 300 * 320 * 340 * 360 * 380 *
Ikl : VFSKVSQANLWLFKDPFMYYVRYQGKSILASKGTFLLMKKWKYYLVNFCQSYFDLWSNPGRIYVNQLCKHSLDFMGYLSSVQLNSSIIRSQMLENSFLVDNAIKKFDTILPIITLIGSLAKAKFCNTLGH : 385
Ili : -----------------MYYVRYQGKSILASKGTFLLMKKWKYYLVNFCQSYFDLWSNPGRIYVNRLCKHSLDFMGYLSSVQLNSSIIRSQMLENSFLVDNAIKKFDTILPIITLIGSLAKAKFCNTLGH : 221
Isc : -----------LLKDPFMYYVRYQGKSILASKGTFLLMNKWKYYLVNFCQSYFHLWSNPGRIYINRLCKHSLDFMGYLSSVQLNSSIIRSQMLENSFLVDNAIKKFDTILPIITLIGSLAKAKFCNTLGH : 197
Ici : VFSKVSQANLWLFKDPFMYYVRYQGKSILASKGTFLLMNKWKYYLVNFCQSYFDLWSNPGRIHINRLSKHSLDFMGYLSSVQLNSSIIRSQMLENSFLVDNAIKKFDTILPIITLIGSLAKAKFCNTLGH : 385
Ipe : VFSKVSQANLWLFKDPFMYYVRYQGKSILASKGTFLLMKKWKYYLVNFCQSYFDLWSNPGRIYINRLCKHSLDFMGYLSSVQLNSSIIRSQMLENSFLVDNAIKKFDTILPIITLIGSLAKAKFCNTLGH : 383
Ise : --------------------------SHLRSTSYGAFIERISFYLK--MECVVEVFS--------RLCKHSLDFMGYLSSVQLNSSIIRSQMLENSFLVDNAITKFDTILPIITLIGSLAKAKFCNTLGH : 301
Ath : VFVNNFPAILGLLKDPFIHYVRYHGRCILATKDTPLLMNKWKYYFVNLWQCYFSVWFQSQKVNINQLSKDNLEFLGYLSSLRLNPLVVRSQMLENSFLIDNVRIKLDSKIPISSIIGSLAKDKFCNVLGH : 385

400 * 420 * 440 * 460 * 480 * 500
Ikl : PISKPVRAHLSDSDIIDRFGRICRNLFHYYSGSSKKKRLYRIKYILRLSCARTLARKHKSTVRAFLK---PEFFEKFLTAEGEVLSLTFPIVSSILRGLYRRRIWYLDIICINDLANYE : 501
Ili : PISKPVRAHLSDSDIIDRFGRICRNLFHYYSGSSKKKRLYRIKYILRLSCARTLARKHKSTVRAFLK---PEFFEKFLTAEGEVLSLTFPIVSSILRGLYRRRIWYLDIICINDLANYE : 337
Isc : PISKPVRAHLSDSDIIDRFGRICRNLFHYYSGSSTKKRLYRIKYILRLSCARTLARKHKSTVRAFLK---PEFLEKFLTAEGEVLSLTFPIVSSILRGLYKRRIWYLDIIFINDPANYE : 313
Ici : PISKPVRAHLSDSDIIDRFGRICRNLFHYYSGSSTKKRLYRIKYILRLSCARTLARKHKSTVRAFLK---PEFLEKFLTAEGEVLSLTFPIVSSILRGLYKRRIWYLDIICINDLANYE : 501
Ipe : PISKPVRAHLSDSDIIDRFGRLCRNLFHYYSGSSKKKRLYRIKYILRLSCARTLARKHKSTVRDFLK---PEFLEKFLTAEGEVLSLNFPIVSSILRGLYIRRIWYLDIICINDLANYE : 499
Ise : PISKPVRAHLSDSDIIDRFGRICRNLFHYYSGSSKKKRLYRIKYILRLSCARTLARKHKSTLRAFLK---PEFLEKFLTAEGEVLSLTFPIVSSILRGLYRRRIWYLDIICINDLANYE : 417
Ath : PISKATWTDSSDSDILNRFVRICRNISHYYSGSSKKKNLYRIKYILRLCCVKTLARKHKSTVRTFLKRLGSGLLEEFLTGEDQVLSLIFPRSYYASKRLYRVRIWYLDILYLNDLVNHE : 504

**Figure S12 Multiple sequence alignment of the deduced amino acid sequences of MATK protein.** The origins of the protein sequences used in this alignment are from: *Arabidopsis thaliana* (Ath), *Iodes klaineana* (Ikl), *Iodes cirrhosa* (Ici), *Iodes seretii* (Ise), *Iodes scandens* (Isc), *Iodes perrieri* (Ipe) and *Iodes liberica* (Ili). The amino acid numbers for each sequence are indicated on the right. In the sequence alignment, identical residues are shown with a black background, and similar residues are shown with a gray background.

* 20 * 40 * 60 * 80 * 100 * 120 *
Ipe : MIIDTTEVQAIKSFSRLELESLKEVYGIIGGLVPILTLVLGITIGVLVIVWLERQISAGIQQRIGPEYAGPLGILQVLADGTKLLLKENLLPSRGNIPLFSIGPSIAVISILLSYLVIPFGYHLVLADLSIG : 132
Ili : MRIDTTEVQAIKSFSRLELESLKEVYGIIGGLVPILTLVLGITIGVLVIVWLERQISAGIQQRIGPEYAGPLGILQVLADGTKLLLKENLLPSRGNIPLFSIGPSIAVISILLSYLVIPFGYHLVLADLSIG : 132
Ici : MIIDTTEVQAIKSFSRLELESLKEVYGIIGGLVPILTLVLGITIGVLVIVWLEREISAGIQQRIGPEYAGPLGILQVLADGTKLLFKENLLPSRGNIPLFSIGPSIAVISILLSYLVIPFGYHLVLANLSIG : 132
Ikl : MRIDTTEVQAIKSFSRLELESLKEVYGIIGGLVPILTLVLGITIGVLVIVWLEREISAGIQQRIGPEYAGPLGILQVLADGTKLLLKENLLPSRGNIPLFSIGPSIAVISILLSYLVIPFGYHLVLADLSIG : 132
Ise : MIIDTTEVQAIKSFSRLEFESLKEVYGIIGGLVPILTLVLGITIGVLVIVWLEREISAGIQQRIGPEYAGPLGILQVLADGTKLLLKENLLPSRGNIPLFSIGPSIAVISILLSYLVIPFGYHLVLADLSIG : 132
Isc : MIIDTTEVQAIKSFSRLELESLKEVYGIIGGLVPILTLVLGITIGVLVIVWLERET--------------------------KLLFKENLLPSRGNIPLFSIGPSIAVISILLSYLVIPFGYHLVLADLSIG : 106
Ath : MIIYATAVQTINSF--VKLESLKEVYGLIWIFVPIFSLVLGIITGVLVIVWLEREISAGIQQRIGPEYAGPLGILQALADGTKLLFKENLRPSRGNTPLFSIGPSIAVISILLSYSVIPFSNHLVLADLNIG : 130

 140 * 160 * 180 * 200 * 220 * 240 * 260
Ipe : LFLWIAISSIAPVGLLMSGYGSNNKYSFLGGLRAAAQSISYELPLTLCVLSISLT------VDIVEAQSKFGCWGWNLWRQPIGFIVFLISSLAECERLPFDLPEAEEELVAGYQTEYSGIKFGLFYIASYL : 258
Ili : LFLWIAISSIAPVGLLMSGYGSNNKYSFLGGLRAAAQSISYELPLTLCVLSISL-------------------------------------------------------------TEYSGIKFGLFYIASYL : 203
Ici : LFLWIAISSIAPVGLLMSGYGSNNKYSFLGGLRAAAQSISYELPLTLCVLSISLLSNSSSTVDIVEAQSKFGFWGWNLWRQPIGFIVFLISSLAECERLPFDLPEAEEELVAGYQTEYSGIKFGLFYIASYL : 264
Ikl : LFLWIAISSIAPVGLLMSGYGSNNKYSFLGGLRAAAQSISYELPLTLCVLSISLLSNSSSTVDIVEAQSKFGFWGWNLWRQPIGFIVFLISSLAECERLPFDLPEAEEELVAGYQTEYSGIKFGLFYIASYL : 264
Ise : LFLWISISSIAPVGLLMSGYGSNNKYSFLGGLRAAAQSISYELPLTLCVLSISLLSNSSSTVDIVEAQSKFGFWGWNLWRQPIGFIVFLISSLAECERLPFDLPEAEEELVAGYQTEYSGIKFGLFYIASYL : 264
Isc : LFLWIAISSIAPVGLLMSGYGSNNKYSFLGGLRAAAQSISYELPLTLCVLSISLLS-------------------------------------------------------------NSSSTVDIVEAQSKF : 177
Ath : IFLWIAISSIAPIGLLMSGYGSNNKYSFLGGLRAAAQSISYEIPLTLCVLSISLLSNSLSTVDIVEAQSKYGFWGWNLWRQPIGFIIFLISSLAECERLPFDLPEAEEELIAGYQTEYSGIKFGLFYVASYL : 262

 * 280 * 300 * 320 * 340 * 360
Ipe : NLLVSSLFVTVLYLGGWNLSIPYIFVPGLLDINKAGRVFGIIIGIFITLA-KTYLFLCIS-ITTRWTLPRLRMDQLLNLGWKFLLPISLGN------------ : 347
Ili : NLLVSSLFVTVLYLGGWNLSIPYIFVPGLLDINKAGRVFGIIIGIFITLA-KTYLFLCIS-ITTRWTLPRLRMDQLLNLGWKFLLPISLGN------------ : 292
Ici : NLLVSSLFVTVLYLGGWNLSIPYIFVPGLLDINKAGRVFGIIIGIFITLA-KTYLFLCIS-ITTRWTLPRLRMDQLLNLGWKFLLPISLGNLLLTTSSQLFSL : 365
Ikl : NLLVSSLFVTVLYLGGWNLSIPYIFVP--------GLVFGIILGIFITLA-KTYLFLCIS-ITTRWTLPRLRMDQLLNLGWKFLLPISLGN------------ : 345
Ise : NLLVSS----------------------------AGRVFGIIIDIFITLA-KTYLFLCIS-ITTRWTLPRLRMDQLLNLGWKF-------------------- : 317
Isc : GFWGWNLWRQP--IGFIVFLISSLAECERLPFDLPEAEEELVAGYQTEYSGIKFGFSCLRQVITRWTLPRLRMDQLLNLGWKFLLPISLGN------------ : 266
Ath : NLLISSLFVTVLYLGGWNISIPYISILELFQ---RDQIFGTTIGIFITLA-KTYLFLFVS-IATRWTLPRLRMDQLLNLGWKFLLPISLGNLLLTTSFQLFSL : 360

**Figure S13 Multiple sequence alignment of the deduced amino acid sequences of NDHA protein.** The origins of the protein sequences used in this alignment are from: *Arabidopsis thaliana* (Ath), *Iodes klaineana* (Ikl), *Iodes cirrhosa* (Ici), *Iodes seretii* (Ise), *Iodes scandens* (Isc), *Iodes perrieri* (Ipe) and *Iodes liberica* (Ili). The amino acid numbers for each sequence are indicated on the right. In the sequence alignment, identical residues are shown with a black background, and similar residues are shown with a gray background.

* 20 * 40 * 60 * 80 * 100 * 120 *
Ise : MIWHVQNENVILDSTRIFMKAFHLLLFDGSFIL----------PECILIFGLILLLMIDSTSDQK-DIPWFYFISSTSLVMSITALLFRWREEPMISFSGNFQTNNFNEIFQFLILLCSTLCIPLSVEYI : 119
Ici : MIWHVQNENVILDSTRIFMKAFHLLLFDGSFIL----------PECILIFGLILLLMIDSTSDQK-DIPWFYFISSTSLVMSITALLFRWREEPMISFSGNFQTNNFNEIFQFLILLCSTLCIPLSVEYI : 119
Ili : MIWHVQNENVILDSTRIFMKAFHLLLFDGSFIL----------PECILIFGLILLLMIDSTSDQK-DIPWFYFISSTSLVMSITALLFRWREEPMISFSGNFQTNNFNEIFQFLILLCSTLCIPLSVEYI : 119
Ipe : MIWHVQNENVILDSTRIFMKAFHLLLFDGSFIL----------PECILIFGLILLLMIDSTSDQK-DIPWFYFISSTSLVMSITALLFRWREEPMISFSGNFQTNNFNEIFQFLILLCSTLCIPLSVEYI : 119
Ikl : MIWHVQNENVILDSTRIFMKAFHLLLFDGSFIL----------PECILIFGLILLLMIDSTSDQK-DIPWFYFISSTSLVMSITALLFRWREEPMISFSGNFQTNNFNEIFQFLILLCSTLCIPLSVEYI : 119
Ath : MIWHVQNENFILDSTRIFMKAFHLLLFDGSFIF----------PECILIFGLILLLMIDSTSDQK-DIPWLYFISSTSFVMSITALLFRWREEPMISFSGNFQTNNFNEIFQFLILLCSTLCIPLSVEYI : 119
Isc : MIWHVQNENVILDSTRIFMKAFHLLLLDGSFILLFLVEVPIILPECILIFGLILLLMIDSTSDKKKDIPWFYFISSTSLVMSITALLFRWREEPMISFSGNFQTNNFNEIFQFLILLCSTLCIPLSVEYI : 130

 140 * 160 * 180 * 200 * 220 * 240 * 260
Ise : ECTEMAITEFLLFVLTATLGGMFLCGANDLITIFVAPECFSLCSYLLSGYTKKDVRSNEATMKYLLMGGASSSILVHGFSWLYGSSGGEIELQEIVNGLINTQMYNSPGISIALIFITVGIGFKLSLAPS : 249
Ici : ECTEMAITEFLLFVLTATLGGMFLCGANDLITIFVAPECFSLCSYLLSGYTKKDVRSNEATMKYLLMGGASSSILVHGFSWLYGSSGGEIELQEIVNGLINTQMYNSPGISIALIFITVGIGFKLSLAPS : 249
Ili : ECTEMAITEFLLFVLTATLGGMFLCGANDLITIFVAPECFSLCSYLLSGYTKKDVRSNEATMKYLLMGGASSSILVHGFSWLYGSSGGEIELQEIVNGLINTQMYNSPGISIALIFITVGIGFKLSLAPS : 249
Ipe : ECTEMAITEFLLFVLTATLGGMFLCGANDLITIFVAPECFSLCSYLLSGYTKKDVRSNEATMKYLLMGGASSSILVHGFSWLYGSSGGEIELQEIVNGLINTQMYNSPGISIALIFITVGIGFKLSLAPS : 249
Ikl : ECTEMAITEFLLFVLTATLGGMFLCGANDLITIFVAPECFSLCSYLLSGYTKKDVRSNEATMKYLLMGGASSSILVHGFSWLYGSSGGEIELQEIVNGLINTQMYNSPGISIALIFITVGIGFKLSLAPS : 249
Ath : ECTEMAITEFLLFILTATLGGMFLCGANDLITIFVAPECFSLCSYLLSGYTKKDIRSNEATMKYLLMGGASSSILVHGFSWLYGSSGGEIELQEIVNGLINTQMYNSPGISIALIFITVGIGFKLSLAPS : 249
Isc : ECTEMAITEFLLFVLTATLGGMFLCGANDLITIFVAPECFSLCSYLLSGYTKKDVRSNEATMKYLLMGGASSSILVHGFSWLYGSSGGEIELQEIVNGLINTQMYNSPGISIALIFITVGIGFKLSLAPS : 260

 * 280 * 300 * 320 * 340 * 360 * 380 *
Ise : HQWTPDVYEG----------------------------------SPTPVVAFLSVTSKVAASASATRIFDIPFYFSSNEWHLLLEILGILSMILGNLIAITQTSMKRMLAYSSIGQIGYVIIGIIVGDSN : 345
Ici : HQWTPDVYEG----------------------------------SPTPVVAFLSVTSKVAASASATRIFDIPFYFSSNEWHLLLEILGILSMILGNLIAITQTSMKRMLAYSSIGQIGYVIIGIIVGDSN : 345
Ili : HQWTPDVYEG----------------------------------SPTPVVAFLSVTSKVAASASATRIFDIPFYFSSNEWHLLLEILGILSMILGNLIAITQTSMKRMLAYSSIGQIGYVIIGIIVGDSN : 345
Ipe : HQWTPDVYEG----------------------------------SPTPVVAFLSVTSKVAASASATRIFDIPFYFSSNEWHLLLEILGILSMILGNLIAITQTSMKRMLAYSSIGQIGYVIIGIIVGDSN : 345
Ikl : HQWTPDVYEG----------------------------------SPTPVVAFLSVTSKVAASASATRIFDIPFYFSSNEWHLLLEILGILSMILGNLIAITQTSMKRMLAYSSIGQIGYVIIGIIVGDSN : 345
Ath : HQWTPDVYEG----------------------------------SPTPVVAFLSVTSKVAASASATRIFDIPFYFSSNEWHLLLEILAILSMIFGNLIAITQTSMKRMLAYSSIGQIGYVIIGIIVGDSN : 345
Isc : HQWTPDVYEGVRFVRKIPTSLSISEMFGFFKTPWTCRREMLSPLSPTPVVAFLSVTSKVAASASATRIFDIPFYFSSNEWHLLLEILGILSMILGNLIAITQTSMKRMLAYSSIGQIGYVIIGIIVGDSN : 390

400 * 420 * 440 * 460 * 480 * 500 * 520
Ise : DGYASMITYMLFYIAMNLGTFACIVLFGLRTGTDNIRDYAGLYTKDPFLALSLTLCLLSLGGLPPLAGFFGKLYLFWCGWQAGLYFLVLIGLLTSVVSIYYYLKIIKLMTGRNQEITPHVRNYRRSPLR : 475
Ici : DGYASMITYMLFYIAMNLGTFACIVLFGLRTGTDNIRDYAGLYTKDPFLALSLTLCLLSLGGLPPLAGFFGKLYLFWCGWQAGLYFLVLIGLLTSVVSIYYYLKIIKLMTGRNQEITPHVRNYRRSPLR : 475
Ili : DGYASMITYMLFYIAMNLGTFACIVLFGLRTGTDNIRDYAGLYTKDPFLALSLTLCLLSLGGLPPLAGFFGKLYLFWCGWQAGLYFLVLIGLLTSVVSIYYYLKIIKLMTGRNQEITPHVRNYRRSPLR : 475
Ipe : DGYASMITYMLFYIAMNLGTFACIVLFGLRTGTDNIRDYAGLYTKDPFLALSLTLCLLSLGGLPPLAGFFGKLYLFWCGWQAGLYFLVLIGLLTSVVSIYYYLKIIKLMTGRNQEITPHVRNYRRSPLR : 475
Ikl : DGYASMITYMLFYIAMNLGTFACIVLFGLRTGTDNIRDYAGLYTKDPFLALSLTLCLLSLGGLPPLAGFFGKLYLFWCGWQAGLYFLVLIGLLTSVVSIYYYLKIIKLMTGRNQEITPHVRNYRRSPLR : 475
Ath : GGYASMITYMLFYIAMNLGTFACIILFGLRTGTDNIRDYAGLYTKDPFLALSLALCLLSLGGLPPLAGFFGKLHLFWCGWQAGLYFLVSIGLLTSVLSIYYYLKIIKLMTGRNQEITPHMRNYRISPLR : 475
Isc : DGYASMITYMLFYIAMNLGTFACIVLFGLRTGTDNIRDYAGLYTKDPFLALSLTLCLLSLGGLPPLAGFFGKLYLFWCGWQAGLYFLVNF---------------------------------------- : 480

 * 540 * 560 *
Ise : SNNSIELSMIVCVIASTIPGISMNPIIAIAQDTLFVLFDLFYIAMNLGTFACIVLFG : 532
Ici : SNNSIELSMIVCVIASTIPGISMNPIIAIAQDTLF---------------------- : 510
Ili : SNNSIELSMIVCVIASTIPGISMNPIIAIAQDTLF---------------------- : 510
Ipe : SNNSIELSMIVCVIASTIPGISMNPIIAIAQDTLF---------------------- : 510
Ikl : SNNSIELSMIVCVIASTIPGISMNPIIAIAQDTLF---------------------- : 510
Ath : SNNSIELSMIVCVIASTIPGISMNPIIAIAQDTLFSF-------------------- : 512
Isc : -----ELSMIVCVIASTIPGISMNPIIAIAQDTLF---------------------- : 510

**Figure S14 Multiple sequence alignment of the deduced amino acid sequences of NDHB protein.** The origins of the protein sequences used in this alignment are from: *Arabidopsis thaliana* (Ath), *Iodes klaineana* (Ikl), *Iodes cirrhosa* (Ici), *Iodes seretii* (Ise), *Iodes scandens* (Isc), *Iodes perrieri* (Ipe) and *Iodes liberica* (Ili). The amino acid numbers for each sequence are indicated on the right. In the sequence alignment, identical residues are shown with a black background, and similar residues are shown with a gray background.

* 20 * 40 * 60 * 80 * 100 * 120 *
Ikl : TNYFPWLTIIVVLPISAGSLIFFLPHRGNRTIQWYTICICILELLLTTYTFCYHFQPDDPLIQLVEDYKWINFFDFRWRLGIDGLSIGPILLTGFITTLATLVAPPVTRDSRLFHFLMLAMYSGQIGSFS : 130
Ili : ---------------------------------------------------------------------------------------------------------------------------------- : -
Ipe : TNYFPWLTIIVVLPISAGSLIFFLPHKGNRTIRWYTICICILELLLTTYTFCYHFQPDDPLIQLVEDYKWINFFDFHWRLGIDGLSIGPILLTGFITTLATLVARPVTRDSRLFHFLMLAMYSGQIGSFS : 130
Ise : -------------PISAGSLIFFLPHRGNRTIKWYTICICILELLLTTYTFCYHFQPDDPLIQLVEDYKWINFFDFHWRLGIDGLSIGPILLTGFITTLATLVARPVTRDSRLFHFLMLAMYSGQIGSFS : 117
Ici : TNYFPWLTIIVVLPISAGSLIFFLPHRGNRTIQWYTICICILELLLTTYTFCYHFQPGDPLIQLVEDYKWINFFDFHWRLGIDGLSIGPILLTGFITTLATLVARPVTRDSRLFHFLMLAMYSGQIGSFS : 130
Isc : TNYFPWLTIIVVLPISAGSLIFFLPHRGNRTILWYTICICILELLLTTYTFCYHFQPGDPLIQLVEDYKWINFFYFHWRLGIDGLSIGPILLTGFITTLATLVARPVTRDSRLFHFLMLAMYSGQIGSFS : 130
Ath : -NDFPWLTIIVVFPISAGSLMLFLPHRGNKVNKWYTICICILELLLTTYAFCYNFKMDDPLIQLSEDYKWIDFFDFYWRMGIDGLSIGTILLTGFITTLATLAAFPVTRDSRFFHFLMLAMYSGQIGSFS : 129

 140 * 160 * 180 * 200 * 220 * 240 * 260
Ikl : SQNLLLFFIMWELELIPVYILLSMWGGKKRLYSATKFILYTAGGSVFLLMSVLGIHLYGSNEQLTFHFETLATRSYPVALEILFYIGFFIAFAVKLPIIPLHTWLPDTHGEAHYSTCMLLA--GILLKMG : 258
Ili : ----------------------------------------------------------GSNEQLTFHFETLATRSYPVALEILFYIGFFIAFAVKLPIIPLHTWLPDTHGEAHYSTCMLLA--GILLKMG : 70
Ipe : SQNLLFFFIMWELELIPVYILLSMWGGKKRLYSATKFILYTAGGSIFLLMSVLGIHLYGSNE-PTFHFETLATRSYPVALEILFYIGFFIAFAVKLPIIPLHTWLPDTHGEAHYSTCMLLA--GILLKMG : 257
Ise : SQNLLLFFIMWELELIPVYILLSMWGGKKRLYSATKFILYTAGGSVFLLMSVLGIHLYGSNE-PTFNFETLATRSYPVALEILFYIGFFIAFAVKLPIIPLHTWLPDTHGEAHYSTCMLLA--GILLKMG : 244
Ici : SQNLLLFFIMWELELIPVYILLSTWGGKKRLYSATKFILYTAGGSVFLLMSVLGIHLYGSNE-PTFHFEILATRSYPVALEILFYIGFFIAFAVKLPIIPLHTWLPDTHGEAHYSTCMLLA--GILLKMG : 257
Isc : SQNLLLFFIMWELELIPVYILLSMWGGKKRLYSATKFILYTAGGSVFLLMSVLGIHLYGSNE-PTFHFETLTTRSYPVALEILFYIGFFIAFAVKLPIIPLHTWLPDTHGEAHYSTCMLLAGIGILLKMG : 259
Ath : SRDLLLFFIMWELELIPVYLLLSMWGGKKRLYSATKFILYTAGSSIFLLIGVLGISLYGSNE-PTLNLELLANKSYPVTLEILFYIGFLIAFAVKSPIIPLHTWLPDTHGEAHYSTCMLLA--GILLKMG : 256

 * 280 * 300 * 320 * 340 * 360 * 380 *
Ikl : AYGLIRINMELLPHAHSIFSPWLMTVGAIQIIYAASTSPGQRNFKKRLAYSSVSHMGFIIIGIGSITDMGLNGALLQIISHGFIGAALFFLAGTSYDRIRLVYLDEMGGIGIPMPKIFTMFSSFSMASLA : 388
Ili : AYGLIRINMELLPHAHSIFSPWLMTVGAIQIIYAASTSPGQRNLKKRLAYSSVSHMGFIIIGIGSITDMGLNGALLQIISHGFIGAALFFLAGTSYDRIRLVYLDEMGGIGIPMPKIFTMFSSFSMASLA : 200
Ipe : AYGLIRINMELLPHAHSIFSPWLMTVGAIQIIYAASTSPGQRNLKKRLAYSSVSHMGFIIIGIGSITDMGLNGALLQIISHGFIGAALFFLAGTSYDRIRLVYLDEMGGIGIPMPKIFTMFSSFSMASLA : 387
Ise : AYGLIRINMELLPHAHSIFSPWLMTVGAIQIIYAASTSPGQRNLKKRLAYSSVSHMGFIIIGIGSITDMGLNGALLQIISHGFIGAALFFLAGTSYDRIRLVYLDEMGGIGIPMPKIFTMFSSFAMASLA : 374
Ici : AYGLIRINMELLPHAHSIFSPWLMTVGAIQIIYAASTSPGQRNFKKRLAYSSVSHMGFIIIGIGSITDMGLNGALLQIISHGFIGAALFFLAGTSYDRIRLVYLDEMGGIGIPMPKIFTMFSSFSMASLA : 387
Isc : AYGLIRINMGLMNFNE--------RSGAIQIIYAASTSPGQRNLKKRLAYSSVSHMGFIIIGIASITDMGLNGALLQIISHGFIGAALFFLAGTNYDRIRLVYLDEMGGIG------------------- : 362
Ath : AYGLVRINMELLPHAHSMFSPWLLVVGTIQIIYAASTSPGQRNLKKRIAYSSVSHMGFIIIGISSITDPGLNGAILQIISHGFIGAALFFLAGTSYDRIRLVYLDEMGGMAISIPKIFTMFTILSMASLA : 386

400 * 420 * 440 * 460 * 480 * 500 * 520
Ikl : LPGMSGFVAELIVFFGIITSQKCLLMPKILITLVMAIGMILTPIYSLSMLRQIFYGYKLFNTPNSYFFDSGPRELFVSISIFLPVIGIGIYPDFVLSLSVDKVEVILSNYFYR----------------- : 501
Ili : LPGMSGFVAELIVFFGIITSQKCLLMPKILITLVMAIGMILTPIYSLSMLRQIFYGYKLFNTPNSYFFDSGPRELFVSISIFLPVIGIGIYPDFVLSLSVDKVEVILSNYFYR----------------- : 313
Ipe : LPGMSGFVAELIVFFGIITSQKCLLMPKILITLVMAIGMILTPIYSLSMLRQIFYGYKLFNTPNSYFFDSGPRELFVSISIFLPVIGIGIYPDFVLSLSVDKVEVILSNFFYRTSSNLIQVYIFWELVGM : 517
Ise : LPGMSGFVAELIVFFGIITCQKCLLMPKIIITLVMAIGMILTPIYSLSMLRQIFYGYKLFNTPNSYFFDSGPRELFVSISIFLPVIGIGIYPDFVLSLSVDKVEVILSNY-------------------- : 484
Ici : LPGMSGFVAELIVFFGIITSQKCLLMTKILITLVMAIGMILTPIYSLSMLRQIFYGYKLFNTPNSYFFDSGPRELFVSISIFLPVIGIGIYPDFVLSLSVDKVELILSNYFYR----------------- : 500
Isc : ---------------------------------------------------------------------------------------------------------------------------------- : -
Ath : LPGMSGFIAEFIVFFGIITSQKYFLISKIFIIFVMAIGMILTPIYLLSMLRQMFYGYKLINIKNFSFFDSGPRELFLSISILLPIIGIGIYPDFVLSLASDKVESILSNYFYG----------------- : 499

 * 540 * 560 * 580 * 600 * 620 * 640 *
Ikl : ---------------------------------------------------------------------------------------------------------------------------------- : -
Ili : ---------------------------------------------------------------------------------------------------------------------------------- : -
Ipe : CSYLLIGFWFTRPAAANACQKAFVTNRVGDFGLLLGILSFYWITGTFEFRDLFEILTNLISNSEVNFLFVTLSAVLLFAGAVAKSAQFPLHVWLPDAMEGPTPISALIHAATMVAAGIFLVARLLPLFIV : 647
Ise : ---------------------------------------------------------------------------------------------------------------------------------- : -
Ici : ---------------------------------------------------------------------------------------------------------------------------------- : -
Isc : ---------------------------------------------------------------------------------------------------------------------------------- : -
Ath : ---------------------------------------------------------------------------------------------------------------------------------- : -

**Figure S15 Multiple sequence alignment of the deduced amino acid sequences of NDHD protein.** The origins of the protein sequences used in this alignment are from: *Arabidopsis thaliana* (Ath), *Iodes klaineana* (Ikl), *Iodes cirrhosa* (Ici), *Iodes seretii* (Ise), *Iodes scandens* (Isc), *Iodes perrieri* (Ipe) and *Iodes liberica* (Ili). The amino acid numbers for each sequence are indicated on the right. In the sequence alignment, identical residues are shown with a black background, and similar residues are shown with a gray background.

* 20 * 40 * 60 * 80 * 100
Isc : MILEYVLVLSAYLFSVGIYGLITSRNMVRALMCFELILNAVNINFVTFSDFFDSRQLKGSIFSI------------------------------------- : 64
Ici : MILEYVLVLSAYLFSVGIYGLITSRNMVRALMCFELILNAVNINFVTFSDFFDSRQLKGSIFSIFVIGIAAAEAAIGSAIVSSIYRNRKSTRINQSNLLNK : 101
Ili : MILEYVLVLSAYLFSIGIYGLITSRNMVRALMCFELILNAVNINFVTFSDFFDSRQLKGSIFSIFVIGIAAAEAAIGSAIVSSIYRNRKSTR--------- : 92
Ipe : MILEYVLVLSAYLFSIGIYGLITSRNMVRALMCFELILNAVNINFVTFSDFFDSRQLKGSIFSIFVIGIAAAEAAIGSAIVSSIYRNRKSTRINQSNLLNK : 101
Ikl : MILEYVLVLSAYLFSIGIYGLITSRNMVRALMCFELILNAVNINFVTFSDFFDSRQLKGSIFSIFVIGIAAAEAAIGSAIVSSIYRNRKSTRINQSNLLNK : 101
Ise : MILEYVLVLSAYLFSIGIYGLITSRNMVRALMCFELILNAVNYRNRKSTRINQSNLLNK------------------------------------------ : 59
Ath : MILEHVLVLSAYLFLIGLYGLITSRNMVRALMCLELILNAVNMNFVTFSDFFDNSQLKGEIFCIFVIAIAAAEAAIGLAIVSSIYRNRKSIRINQSTLLNK : 101

**Figure S16 Multiple sequence alignment of the deduced amino acid sequences of NDHE protein.** The origins of the protein sequences used in this alignment are from: *Arabidopsis thaliana* (Ath), *Iodes klaineana* (Ikl), *Iodes cirrhosa* (Ici), *Iodes seretii* (Ise), *Iodes scandens* (Isc), *Iodes perrieri* (Ipe) and *Iodes liberica* (Ili). The amino acid numbers for each sequence are indicated on the right. In the sequence alignment, identical residues are shown with a black background, and similar residues are shown with a gray background.

* 20 * 40 * 60 * 80 * 100 * 120
Ise : MEQTYQYAWIIPLVPLPVPMLIGVGLLLFPAATKNFRRMWAFFSVLLLSIAMAFSINLSIQQINNSSIYQYVWSWIINNDFSLEFGYLIDPLTSIMSILITTVGIMVLIY--SDNYMYHDRGYLRFFAY : 127
Ili : -----QYAWIIPLVPLPVPMLIGVGLLLFPTATKNFRRMSSFFSVLLLSIAMAFSINLSIQQINNSSIYQYVWSWIINNDFSLEFGYLIDPLTSIMSILITTVGIMVLIY--SDNYMYHDRGYLRFFAY : 122
Ath : MEHTYQYSWIIPFIPLPVPILLGVGLLLFPTATKNLRRMWTFLSIFLLSIVMIFSIYLSIQQIFLSCIHQNVWSWTINNEFSFEFGYFIDPLTSIMSILITTVGILVLIY--SDNYMSHDQGYLRFFAY : 127
Ikl : MEQTYQYAWIIPLVPLPVPMLIGVGLLLFPTATKNFRRMWSFFSVLLLSIAMAFSINLSIQQINNSSIYQYVWSWIINNDFSLEFGYLIDPLTSIMSILITTVGIMVLIYIYSDNYMYHDRGYLRFFAY : 129
Ipe : MEQTYQYAWIIPLVPLPVPMLIGVGLLLFPTATKNFRRMWSFFSVLLLSIAMAFSINLSIQQINNSSIYQYVWSWIINNDFSLEFGYLIDPLTSIMSILITTVGIMVLIY--SDNYMYHDRGYLRFFAY : 127
Isc : --------------------------IIFPTATKKFRRMWAFFSVLLLSIAMAFSINLSIEQINNSSIYQYVWSWIINNDFSLEFGYFIDPLTSIMSILITTVGIMVLIY--SDNYMYHDRGYLRFFAY : 101
Ici : MEQTYQYAWIIPLVPLPVPMLIGVGLLLFPTATKNFRRMWAFYSVLLLSIAMAFSINLSIEQINNSSIYQYVWSWIINNDFSLEFGYFIDPLTSIMSILITTVGIMVLIY--SDNYMYHDRGYLRFFAY : 127

 * 140 * 160 * 180 * 200 * 220 * 240 * 2
Ise : MSFFSSSMLGLVTSSNLIQVYIFWELVGMCSYLLIGFWFTRPAAANACQKAFVTNRVGDFGLLLGIFSFYWITGTFEFRDLFEILTNLIS--NSEVNFLFVTLSDVLLFAGAVAKSAQFPLHVWLPDAM : 254
Ili : MSFFSSSMLGLVTSSNLIQVYIFWELVGMCSYLLIGFWFTRPAAANACQKAFVTNRVGDFGLLLGILCFYWITGTFEFRDLFEIFTNLIS--NSEVNFLFVTLSAVLLFAGPVAKSAQFPLHVWLPDAM : 249
Ath : MGFFNTSMLGLVTSSNLIQVYFFWELVGMCSYLLIGFWFTRPIAANACQKAFVTNRVGDFGLLLGILGLYWITGSFEFQDLFEIFNNLIL--NNRVNLLFLTLCAFLLFVGPIAKSAQFPLHVWLPDAM : 254
Ikl : MSFFSSSMLGLVTSSNLIQVYIFWELVGMCSYLLIGFWFTRPAAANACQKAFVTNRVGDFGLLLGILCFYWITGTFEFRDLFEIFTNFISISNSEVNFLFVTLSAVLLFAGPVAKSAQFPLHVWLPDAM : 258
Ipe : MSFFSSSMLELVTSSNLIQVYIFWELVGMCSYLLIGFWFTRPAAANACQKAFVTNRVGDFGLLLGILSFYWITGTFEFRDLFEILTNLIS--NSEVNFLFVTLSAVLLFAGAVAKSAQFPLHVWLPDAM : 254
Isc : MS-----------------VFFFWELVGMCSYLLIGFWFTRPAAANACQKAFVTNRVGDFGLLLGILCFYWITGTFEFRDLFEIFTNLIS--NREVNFLFVTLSAVLLFAGAVAKSAQFPLHVWLPDAM : 211
Ici : MSFFSSFMLGLVTSSNLIQVYIFWELVGMCSYLLIGFWFTRPAAANACQKAFVTNRVGDFGLLLGIFCFYWITGTFEFRDLFEIFTNLIS--NSEVNFLFVTLSAVLLFAGAVAKSAQFPLHVWLPDAM : 254

 60 * 280 * 300 * 320 * 340 * 360 * 380
Ise : EGPTPISALIHAATMVAAGIFLVARLLPLFIVVPYIMNFISLIGLITLLLGATLALAQKDIKRGLAYSTMSQLGYMMLALGMGSYRSALFHLITHAYSKALLFLGSGSVIHSMETIVGYSPDKSQNMVL : 383
Ili : EGPTPISALIHAATMVAAGIFLVARLLPLFIFVPYLMNFISLIGLITLLLGATLALAQKDIKRGLAYSTMSQLGYMMLALGMGSYRSALFHLITHAYSKALLFLGSGSVIHSMETIVGYSPDKSQNMVL : 378
Ath : EGPTPISALIHAATMVAAGIFLVARLLPLFIVIPSIMYIISLIGIITVLLGATLALAQKDIKRGLAYSTMSQLGYMMLALGMGSYRSALFHLITHAYSKALLFLGSGSIIHSMEAIVGYSPDKSQNMIL : 383
Ikl : EGPTPISALIHAATMVAAGIFLVARLLPLFIFVPYIMNFISLIGLITLLLGATLALAQKDIKRGLAYSTMSQLGYMMLALGMGSYRSALFHLITHAYSKALLFLGSGSVIHSMETIVGYSPDKSQNMVL : 387
Ipe : EGPTPISALIHAATMVAAGIFLVARLLPLFIVVPYIMNFISLIGLITLLLGATLALAQKDIKRGLAYSTMSQLGYMMLALGMGSYRSALFHLITHAYSKALLFLGSGSVIHSMETIVGYSPDKSQNMVL : 383
Isc : EGPTPISALIHAATMVAAGIFLVARLLPLFIV------------------------------------------------------------------------------------------------- : 243
Ici : EGPTPISALIHAATMVAAGIFLVARLLPLFIVVPYIMNFISLIGLITLLLGATLALAQKDIKRGLAYSTMSQLGYMMLALGMGSYRSALFHLITHAYSKALLFLGSGSVIHSMETIVGYSPDKSQNMVL : 383

* 400 * 420 * 440 * 460 * 480 * 500 *
Ise : MGGLTKQIPISKTAFLLGTLSLCGIPPLACFWSKDEIINDSWLYFPIFGIIASATAVLTAFYMFRIYLLTFEGHFNFNFQNYSGTKNTPFFSISLWGQKGSKRINKKLTLLTFI--------------- : 497
Ili : MGGLTKQIPISKTAFLLGTLSLCGIPPLACFWSKDEIISDSWLYFPIFGIIASATAGLTAFYMFRIYLLTFEGHFNFNFQNYSGTKNT---LKTIFG-------------------------------- : 472
Ath : MGGLTKHVPITKTAFLIGTLSLCGIPPLACFWSKDEILNDSLLFSPIFAIIACSTAGLTAFYMFRIYLLTFEGHLNTYFLNYSGKKSGSFYSLSLWGKEEEKKLNKNFGLVPLLTMNNTKRASFFCNKT : 512
Ikl : MGGLTKQIPISKTAFLLGTLSLCGIPPLACFWSKDEIINDSWLYFPIFGIIASATAGLTAFYMFRIYLLTFEGHFNFHFKNYSGTKNTPFYSISLWGQKGSNSNNS----------------------- : 493
Ipe : MGGLTKQIPITKTSFLLGTLSLCGIPPLACFWSKDEIINDSWLYFPIFGIIASATAGLTAFYMFRIYLLTFEGHFNFHFKNYSGTKNSPFYSISLWGQKGSKRITKKFPLLTFFNLNKKSSSLRFKKTD : 512
Isc : --------------FLLGTLSLCGIPPLACFWSKDEILNDSWLYSPIFGIIAYATAGLTAFYMFRIYLLTFEGHFHFENENIYLLTFEGHFHFHFQ--NGSKRINKNFPLLTFFNLNK-KSSSLRFKKT : 355
Ici : MGGLTKQIPISKTAFLLGTLSLCGIPPLACFWSKDEILNDSWLYSPIFGIIAYATAGLTAFYMFRIYLLTFEGHFHFHFQNYSGRKNTSFYSISLWGQKGSKRINKNFPLLTFLNLNK-KSSSLRFKKT : 511

 520 * 540 * 560 * 580 * 600 * 620 * 640
Ise : --------------------------------------------------------------------------------------------------------------------------------- : -
Ili : --------------------------------------------------------------------------------------------------------------------------------- : -
Ath : YKISNNVRN----QIFITVENFGLNTRTFYYPHESDNTILFPMLILVLFTLFIGAIGIPFNQEGIDFDILSKFFTPSINLLHKNSQNFVDWYEFLRNATFSVSIAFFGIFIAYCLYKPFYSSLLNLTLL : 637
Ikl : ----------------------------------------------------------------------------------------MDCYEFVKDAVFSVSIASLGIFIASFLYKPGYSSLQNLDLI : 534
Ipe : ETVRNIIRP----FFTIPHFD---NKNTYEYPYESANTMLCPLLVLVLFTLFSGALGIPFTQEGMYLDILSKWLTPSINLLHQNYNNSMDCYEFVKDAVFSVSIASLGIFIASFLYKPVYSSLQNLDLI : 634
Isc : SRIDEAVRN--IIQPFITISHFD-NKNIYSYPYESANTMLFPLLVLGLFTLFSGALGIPFTQEGMDLDILSKWLTPSINLLHQNSNNSIDWYEFVKDAVFSVSIASLGIFIASFLYKPVYSSLQNLDLI : 481
Ici : SRIDETVRNIILIRPFITISHFD-NKNTYSYPYESSNTMLFPLLVLGLFTLFSGALGIPFTQEGMDLDILSKWLTPSINLLHQNSNNSIDWYEFVKDAVFSVSIASLGIFIASFLYKPVYLSLQNLDLI : 639

**Figure S17 Multiple sequence alignment of the deduced amino acid sequences of NDHF protein.** The origins of the protein sequences used in this alignment are from: *Arabidopsis thaliana* (Ath), *Iodes klaineana* (Ikl), *Iodes cirrhosa* (Ici), *Iodes seretii* (Ise), *Iodes scandens* (Isc), *Iodes perrieri* (Ipe) and *Iodes liberica* (Ili). The amino acid numbers for each sequence are indicated on the right. In the sequence alignment, identical residues are shown with a black background, and similar residues are shown with a gray background.

* 20 * 40 * 60 * 80 * 100 * 120 *
Ikl : MDLPGPIHDFLLVFLGLGLILGCLGVVFLPNPIYSAFSLGLVLVCISLFYILSNSYFVAAAQLLIYVG--AINVLIIFAVMFMNGSEYSKDENLWKVGDGITLMVCTSLFVSLITTISNTSWYGIIWSTRSN : 130
Ili : MDLPGPIHDFLLVFLGLGLILGCLGVVFLPNPIYSAFSLGLVLVCISLFYILSNSYFVAAAQLLIYVG--AINVLIIFAVMFMNGSEYSKDENLWKVGDGITLMVCTSLFVSLITTISNTSWYGIIWSTRSN : 130
Ipe : MDLPGAIHDFLLVFLGLGLILGCLGVVLLPNPIYSAFSLGLVLVCISLFYILSNSYFVAAAQLLIYVG--AINVLIIFAVMFMNGSEYSKDENLWKVGDGITLMVCTSLFVSLITTISNTSWYGIIWATRSN : 130
Ise : MDLPGPIHDFLLVFLGLGLILGCLGVVLLPNPIYSAFSLGLVLVCISLFYILSNSYFVAAAQLLIYVGAIAINVLIIFALMFMNGSEYSKDENLWKVGDGITLMVCTSLFVSLITTISNTSWYGIIWATRSN : 132
Ici : MDLPGPIHDFLLVFLGLGLILGCLGVVLLPNPIYSAFSLGLVLVCISLFYILSNSYFVAAAQLLIYVG--AINVLIIFAVMFMNGSEYSKDENLWKVGDGITLMVCTSLFVSLITTISNTSWYGIIWATRSN : 130
Ath : MDLPGPIHDFLLVFLGSGLLVGGLGVVLLPNPIFSAFSLGFVLVCISLLYILSNSHFVAAAQLLIYVG--AINVLIIFAVMFMNDSEYSTDFNLWTIGNGITSLVCTTILFLLMSTILDTSWYGVIWTTKLN : 130
Isc : MDLPGPIHDFLLVFLGLGLILGCLGVVLLPNPIYSAFSLGLVLVCISLFYILS-----------------------------------------FSLG---LVLVCISLFYILSKYFITL------------ : 76

 140 * 160 *
Ikl : QILEQDLISNSQQIGIRLSTDFFLPFE------------------- : 157
Ili : QILEQDLISNSQQIGIRLSTDFFLPFE------------------- : 157
Ipe : QILEQDLISNSQQIGIRLSTDFFLPFE------------------- : 157
Ise : QILEQDLISNSQQIGIRLSTDFFLPFE------------------- : 159
Ici : LILEQDLISNSQQIGIRLSTDFFLPFELISIILLVALIGAIAVARQ : 176
Ath : QILEQDLISNSQQIGIHLSTDFFLPFELISIILLVALIGAISVARQ : 176
Isc : ------LIPGTKN--------------------------------- : 83

**Figure S18 Multiple sequence alignment of the deduced amino acid sequences of NDHG protein.** The origins of the protein sequences used in this alignment are from: *Arabidopsis thaliana* (Ath), *Iodes klaineana* (Ikl), *Iodes cirrhosa* (Ici), *Iodes seretii* (Ise), *Iodes scandens* (Isc), *Iodes perrieri* (Ipe) and *Iodes liberica* (Ili). The amino acid numbers for each sequence are indicated on the right. In the sequence alignment, identical residues are shown with a black background, and similar residues are shown with a gray background.

* 20 * 40 * 60 * 80 * 100 * 120 *
Ikl : MTAQVTRKDLMIVNMGPHHPSMHGVLRLILTLDGEDVIDCEPILGYLHRGMEKIAENRTILQYLPYVTRWDYLATMFTEAITVNAPEQLGNIQVPKRASYIRVIMLELSRIASHLLWLGPFMADIGAQTPFF : 132
Ili : MTAQVTRKDLMIVNMGPHHPSMHGVLRLILTLDGEDVIDCEPILGYLHRGMEKIAENRTILQYLPYVTRWDYLATMFTEAITVNAPEQLGNIQVPKRASYIRVIMLELSRIASHLLWLGPFMADIGAQTPFF : 132
Ise : MTAQVTRKDLMIVNMGPHHPSMHGVLRLILTLDGEDVIDCEPILGYLHRGMEKIAENRTILQYLPYVTRWDYLATMFTEAITVNAPEQLGNIQVPKRASYIRVIMLELSRIASHLLWLGPFMADIGAQTPFF : 132
Isc : ----------------------------------------EPILGYLHRGMEKIAENRTILQYLPYVTRWDYLATMFTEAITVNAPEQLGNIQVPKRASYIRVIMLELSRIASHLLWLGPFMADIGAQTPFF : 92
Ici : MTAQVTRKDFMIVNMGPHHPSMHGVLRLILTLDGEDVIDCEPILGYLHRGMEKIAENRTILQYLPYVTRWDYLATMFTEAITVNAPEQLGNIQVPKRASYIRVIMLELSRIASHLLWLGPFMADIGAQTPFF : 132
Ipe : MTAQVTRKDLMIVNMGPHHPSMHGVLRLILTLDGEDVIDCEPILGYLHRGMEKIAENRTILQYLPYVTRWDYLATMFTEAITVNAPEQLGNIQVPKRASYIRVIMLELSRIASHLLWLGPFMADIGAQTPFF : 132
Ath : MKRPVTGKDLMIVNMGPHHPSMHGVLRLIVTLDGEDVVDCEPILGYLHRGMEKIAENRAIIQYLPYVTRWDYLATMFTEAITVNGPEQLGNIQVPKRASYIRVIMLELSRIASHLLWLGPFMADIGAQTPFF : 132

 140 * 160 * 180 * 200 * 220 * 240 * 260
Ikl : YIFRERELIYDLFEAATGMRMMHNYFRIGGVAADLPHGWIDKCLDFCDYFLTGVAEYQKLITQNPIFLERVEGVGIIGREEAINWGLSGPMLRASGIQWDLRKIDHYECYDEFDWGVQWQKEGDSLARYLVR : 264
Ili : YIFRERELIYDLFEAATGMRMMHNYFRIGGVAADLPHGWIDKCLDFCDYFLTGVAEYQKLITRNPIFLERVEGVGIIGREEAINWGLSGPMLRASGIQWDLRKIDHYECYDEFDWGVQWQKEGDSLARYLVR : 264
Ise : YIFRERELIYDLFEAATGMRMMHNYFRIGGVAADLPHGWIDKCLDFCDYFLTGVAEYQKLITRNPIFLERVEGVGIIGSEEAINWGLAGPMLRASGIQWDLRKIDHYECYDEFDWGVQWQKEGDSLARYLVR : 264
Isc : YIFRERELIYDLFEAATGMRMMHNYFRIGGVAADLPHGWIDKCLDFCDYFLTGVAEYQKLITRNPIFLERVEGVGIIGREEAINWGLSGPMLRASGIQWDLRKIDHYECYDEFDWGIQWQKEGDSLARYLVR : 224
Ici : YIFRERELIYDLFEAATGMRMMHNYFRIGGVAADLPHGWIDKCLDFCDYFLTGVAEYQKLITRNPIFLERVEGVGIIGREEAINWGLSGPMLRASGIQWDLRKIDHYECYDEFDWGIQWQKEGDSLARYLVR : 264
Ipe : YIFRERELIYDLFEAATGMRMMHNFFRIGGVAADLPHGWIDKCLDFCDYFLTGVAEYQKLITRNPIFLERVEGVGIIGREEAINWGLSGPMLRASGIQWDLRKIDHYESYDEFDWGVQWQKEGDSLARYLVR : 264
Ath : YIFREREFVYDLFEAATGMRMMHNFFRIGGIAADLPYGWIDKCLDFCDYFLTEVVEYQKLITRNPIFLERVEGVGIIGGEEAINWGLSGPMLRASGIPWDLRKIDRYESYDEFEWEIQWQKQGDSLARYLVR : 264

 * 280 * 300 * 320 * 340 * 360 * 380 *
Ikl : ISEMKESVKIIQQALEGIPGGPYENLEIRRFDKVRDP---EFEYRFISKKPSPTFELSKQELYVRVEAPKGELGIFLIGDQNVFPWRWKIRPPGFINLQILPQLVKRMKLADIMTILGSIDIIMGEIDR : 390
Ili : ISEMKESVKIIQQALEGIPGGPYENLEIRRFDKVRDPEWNDFEYRFISKKPSPTFELSKQELYVRVEAPKGELGIFLIGDQNVFPWRWKIRPPGFINLQILPQLVKRMKLADIMTILGSIDIIMGEIDR : 393
Ise : ISEMKESVKIIQQALEGIPGGPYENLEIRRFDKVRDPEWNDFEYRFISKKPSPTFELSKQELYVRIEAPKGELGIFLIGDQNVFPWRWKIRPPGFINLQILPQLVKRMKLADIMTILGSIDIIMGEIDR : 393
Isc : ISEMKESVKIIQQALEGIPGGPYDNLEIRRFDKVRDPEWNDFEYRFFSKKPSPTFELSKQELYVRVEAPKGELGIFLIGDQNVFPWRWKIHPPGFINLQILPQLVKRMKLADIMTILGSIDIIMGEIDR : 353
Ici : ISEMKESVKIIQQALEGIPGGPYENLEIRRFDKVRDPEWNDFEYRFISKKPSPTFELSKQELYVRVEAPKGELGIFLIGDQNVFPWRWKIHPPGFINLQILPQLVKRMKLADIMTILGSIDIIMGEIDR : 393
Ipe : ISEMKESVKIIQQALEGIPGGPYENFEIRRFDKVRDLEWNDFEYRFISKKPSPTFELSKQELYVRVEAPKGELGIFLIGDQNVFPWRWKIRPPGFINLQILPQLVKRMKLADIMTILGSIDIIMGEIDR : 393
Ath : LSEMTESIKIIQQALEGLPGGPYENLESRGFDRKRNPEWNDFEYRFISKKPSPTFELSKQELYVRVEAPKGELGIFLIGDQSGFPWRWKIRPPGFINLQILPELVKRMKLADIMTILGSIDIIMGEVDR : 393

**Figure S19 Multiple sequence alignment of the deduced amino acid sequences of NDHH protein.** The origins of the protein sequences used in this alignment are from: *Arabidopsis thaliana* (Ath), *Iodes klaineana* (Ikl), *Iodes cirrhosa* (Ici), *Iodes seretii* (Ise), *Iodes scandens* (Isc), *Iodes perrieri* (Ipe) and *Iodes liberica* (Ili). The amino acid numbers for each sequence are indicated on the right. In the sequence alignment, identical residues are shown with a black background, and similar residues are shown with a gray background.

* 20 * 40 * 60 * 80 * 100 * 120 *
Isc : MLPMVTEFMNYGQQTVRAARHIGQSFMITLSHAN-------------------------------------------------------------------------------------------------- : 34
Ici : MLPMVTEFMNYGQQTVRAARHIGQSFMITLSHANRLPVTIQYPYEKLITSERFRGRIHFEFDKCIACEVCVRVCPIDLPVVDWKLEMDIRKKRLLNYSIDFGICIFCGNCVEYCPTNCLSMTEEYELSTYDR : 132
Ipe : MLPMVTEFMNYGQQTVRAARYIGQSFMITLSHANRLPVTIQYPYEKLITSERFRGRIHFEFDKCIACEVCVRVCPIDLPVVDWKFEIDIRKKRLLNYSIDFGICIFCGNCVEYCPTNCLSMTEEYELSTYDR : 132
Ikl : MLPMVTEFMNYGQQTVRAARYIGQSFMITLSHANRLPVTIQYPYEKLITSERFRGRIHFEFDKCIACEVCVRVCPIDLPVVDWKLEMDIRKKRLLNYSIDFGICIFCGNCVEYCPTNCLSMTEEYELSTYDR : 132
Ili : MLPMVTEFMNYGQQTVRAARYIGQSFMITLSHANRLPVTIQYPYEKLITSERFRGRIHFEFDKCIACEVCVRVCPIDLPVVDWKLEMDIRKKRLLNYSIDFGICIFCGNCVEYCPTNCLSMTEEYELSTYDR : 132
Ise : MLPMVTEFMNYGQQTVRAARYIGQSFMITLSHANRLPVTIQYPYEKLITSERFRGRIHFEFDKCIACEVCVRVCPIDLPVVDWKLEMDIRKKRLLNYSIDFGICIFCGNCVEYCPTNCLSMTE--------- : 123
Ath : MLPMITGFMNYGQQTLRAARYIGQGFMITLSHTNRLPVTIQYPYEKLITSERFRGRIHFEFDKCIACEVCVRVCPIDLPVVDWKLETNIRKKRLLNYSIDFGICIFCGNCVEYCPTNCLSMTEEYEFSTYDR : 132

 140 * 160 * 180 * 200 * 220
Isc : ------------------------------------------------------------------------------------------- : -
Ici : HKLNYNQIALGRLPMSVIDDYTIRTILNSPKRK---------------------------------------------------------- : 165
Ipe : HKLNYNQIALGRLPMSVIDDYTIRTIWNSPKKKYDTCIGCTQCVRACPTDVLEMIPWDGCKAKQIASAPRTEDCVGCKRCESACPTDFLSV : 223
Ikl : HKLNYNQISLGRLPMSVIDDYTIRTIWNSPKTK---------------------------------------------------------- : 165
Ili : HKLNYNQISLGRLPMSVIDDYTIRTIWNSPKTK---------------------------------------------------------- : 165
Ise : ------------------------------------------------------------------------------------------- : -
Ath : HELNYNQIALGRLPMSVIDDYTIRTIWNSPQTKN----GVNPLI----------------------------------------------- : 172

**Figure S20 Multiple sequence alignment of the deduced amino acid sequences of NDHI protein.** The origins of the protein sequences used in this alignment are from: *Arabidopsis thaliana* (Ath), *Iodes klaineana* (Ikl), *Iodes cirrhosa* (Ici), *Iodes seretii* (Ise), *Iodes scandens* (Isc), *Iodes perrieri* (Ipe) and *Iodes liberica* (Ili). The amino acid numbers for each sequence are indicated on the right. In the sequence alignment, identical residues are shown with a black background, and similar residues are shown with a gray background.

* 20 * 40 * 60 * 80 * 100 * 120 *
Ise : MQGRLSAWLVKHGLIHRSLGFDYQGIETLQIKPEDWHSIAVILYVYGYNYLRSQCAYDVAPGGLLASVYHLTRIEYGVDQPEEVCIKVFAPRRNPRIPSVFWVWKSVDFQEREAYDMLGISYTNHPRLKRIL : 132
Ili : MQGRLSAWLVKHGLIHRSLGFDYQGIETLQIKPEDWHSIAVILYVYGYNYLRSQCAYDVAPGGLLASVYHLTRIEYGVDQPEEVCIKVFAPRRNPRIPSVFWVWKSVDFQEREAYDMLGISYTNHPRLKRIL : 132
Ikl : MQGRLSAWLVKHGLIHRSLGFDYQGIETLQIKPEDWHSIAVILYVYGYNYLRSQCAYDVAPGGLLASVYHLTRIEYGVDQPEEVCIKVFAPRRNPRIPSVFWIWKSVDFQEREAYDMLGISYTNHPRLKRIL : 132
Ipe : MQGRLSAWLVKHGLIHRSLGFDYQGIETLQIKPEDWHSIAVILYVYGYNYLRSQCAYDVAPGGLLASVYHLTRIEYGVDQPEEVCIKVFAPRSNPRIPSVFWVWKSVDFQEREAYDMLGISYTNHPRLKRIL : 132
Ici : MQGRLSAWLVKHGLIHRSLGFDYQGIETLQIKPEDWHSIAVILYVYGYNYLRSQCAYDVAPGGLLASVYHLTRIEYGVDQPEEVCIKVFATRRNPKILSVFWVWKSVDFQEREAYDMLGISYTNHPRLKRIL : 132
Ath : MQGTLSVWLAKRGLVHRSLGFDYQGIETLQIKPEDWHSIAVILYVYGYNYLRSQCAYDVAPGGLLASVYHLTRIEYGVNQAEEVCIKVFTHRSNPRIPSVFWVWKSTDFQERESYDMLGITYDSHPRLKRIL : 132
Isc : --------------------------------------------------------------------------------------------------------KSVDFRNRE-----------HPNQKRIQ : 17

 140 *
Ise : MPESWIGWPLRKDYIAPNFYEIQDAH : 158
Ili : MPESWIGWPLRKDYIAPNFYEIQDAH : 158
Ikl : MPESWIGWPLRKDYIAPNFYEIQDAH : 158
Ipe : MPESWIGWPLRKDYIAPNFYEIQDAH : 158
Ici : MPESWIGWPLRKDYIAPNFYEIQDAH : 158
Ath : MPESWIGWPLRKDYIAPNFYEIQDAY : 158
Isc : YSTSYLNW----------FYEIQDAH : 33

**Figure S21 Multiple sequence alignment of the deduced amino acid sequences of NDHJ protein.** The origins of the protein sequences used in this alignment are from: *Arabidopsis thaliana* (Ath), *Iodes klaineana* (Ikl), *Iodes cirrhosa* (Ici), *Iodes seretii* (Ise), *Iodes scandens* (Isc), *Iodes perrieri* (Ipe) and *Iodes liberica* (Ili). The amino acid numbers for each sequence are indicated on the right. In the sequence alignment, identical residues are shown with a black background, and similar residues are shown with a gray background.

* 20 * 40 * 60 * 80 * 100 * 120 *
Isc : ---------------------------------------------------------------------------------------------------------------------------TVRGVDKLI : 9
Ici : --MNSIEFPLLNRTAQTSVISTTLTDLSNWSRLSSLWPLLYGTSCCFIEFASLIGSRFDFDRYGLVPRSSPRQADLILTAGTVTMKMAPSLVRLYEQMPEPKYVIAMGACTITGGMFSTDSYSTVRGVDKLI : 130
Ili : MNSIEFQFPLLDRTAQTSVISTTLTDLSNWSRLSSLWPLLYGTSCCFIEFASLIGSRFDFDRYGLVPRSSPRQADLILTAGTVTMKMAPSLVRLYEQMPEPKYVIAMGACTITGGMFSTDSYSTVRGVDKLI : 132
Ipe : MNS--IEFPLLDRTAQTSVISTTLTDLSNWSRLSSLWPLLYGTSCCFIEFASLIGSRFDFDRYGLVPRSSPRQADLILTAGTVTMKMAPSLVRLYEQMPEPKYVIAMGACTITGGMFSTDSYSTVRGVDKLI : 130
Ise : MNS--IEFPLLDRTAQTSVISTTLTDLSNWSRLSSLWPLLYGTSCCFIEFASLIGSRFDFDRYGLVPRSSPRQADLILTAGTVTMKMAPSLVRLYEQMPEPKYVIAMGACTITGGMFSTDSYSTVRGVDKLI : 130
Ikl : MNS--IEFPLLDRTAQTSVISTTLTDLSNWSRLSSLWPLLYGTSCCFIEFASLIGSRFDFDRYGLVPRSSPRQADLILTAGTVTMKMAPSLVRLYEQMPEPKYVIAMGACTITGGMFSTDSYSTVRGVDKLI : 130
Ath : MNS--IKFPILDRTTKNSVISTTLNDLSNWSRLSSLWPLLYGTSCCFIEFASLIGSRFDFDRYGLVPRSSPRQADLILTAGTVTMKMAPSLVRLYEQMPEPKYVIAMGACTITGGMFSTDSYSTVRGVDKLI : 130

 140 * 160 * 180 * 200 * 220
Isc : PVDVYLPGCPPKPEAVIDALTKLRKKVSREIYEDRIRSQRENRCFTTKHKFNVGRSMNTGNYDQGFLYQRP--S----------------------- : 81
Ici : PVDVYLPGCPPKPEAVIDALTKLRKKVSREIYEDRIRSQRENRCFTTKHKFNVGRSMNTGNYDQGFLYQRP--STSEILPETFFKYKSPVSSHELVN : 225
Ili : PVDVYLPGCPPKPEAVIDALTKLRKKVSRELYEDRIRSQRENRCFTTKHKFNVGRSIHTGNFNQGFLYQRP--STSEILPETFFKYKSPVSSHELVN : 227
Ipe : PVDVYLPGCPPKPEAVIDALTKLRKKVSRELYEDRIRSQRENRCFITKHKFNVGRSIHTGNYDQGFLYQRP--STSEILPETFFKYKSPVSSHELVN : 225
Ise : PVDVYLPGCPPKPEAVIDALTKLRKKVSRELYEDRIRFQRENRCFTTKHKFNVGRSIHTGNYDQGFLYQRP--STSEILPETFFKYKSPVSSHELVN : 225
Ikl : PVDVYLPGCPPKPEAVIDALTKLRKKVSRELYEDRIRSQRENRCFTTKHKFNVGRSIHTGNYDQGFLYQRPSTSTSEILPETFFKYKSPVSSHELVN : 227
Ath : PVDVYLPGCPPKPEAVIDAITKLRKKIAREIYKDRIRPQQGNRCFTTNHKFFVVRSPHIGNYDQELLYPPS--STSEISTETFFKYKSPVSSHELVN : 225

**Figure S22 Multiple sequence alignment of the deduced amino acid sequences of NDHK protein.** The origins of the protein sequences used in this alignment are from: *Arabidopsis thaliana* (Ath), *Iodes klaineana* (Ikl), *Iodes cirrhosa* (Ici), *Iodes seretii* (Ise), *Iodes scandens* (Isc), *Iodes perrieri* (Ipe) and *Iodes liberica* (Ili). The amino acid numbers for each sequence are indicated on the right. In the sequence alignment, identical residues are shown with a black background, and similar residues are shown with a gray background.

* 20 * 40 * 60 * 80 * 100 * 120 *
Ikl : MQTINTFFWIKEQIT-----------RFISASLMIYIITRTSISSAYPIFAQQGYENPREATGRIVCANCHLANKPVDIEVPQAVLPDTVFEAVVRIPYDMQLKQVLANGKKGALNVGAVLILPEGFELAPS : 121
Ili : MQTINTFFWIKEQIT-----------RFISASLMIYIITRTSISSAYPIFAQQGYENPREATGRIVCANCHLANKPVDIEVPQAVLPDTVFEAVVRIPYDMQLKQVLANGKKGALNVGAVLILPEGFELAPS : 121
Ipe : MQTRNTFFWIKEQIT-----------RFIYASLMIYIITRTSISSAYPIFAQQGYENPREATGRIVCANCHLANKPVDIEVPQAVLPDTVFEAVVRIPYDMQLKQVLANGKKGALNVGAVLILPEGFELAPS : 121
Ise : MQTRNTFFWIKEQIT-----------RFISASLMIYIITRTSISSAYPIFAQQGYENPREATGRIVCANCHLANKPVDIEVPQAVLPDTVFEAVVRIPYDMQLKQVLANGKKGALNVGAVLILPEGFELAPS : 121
Ath : MQTRNTFSWIREEIT-----------RSISVSLIIYIITWASISSAYPIFAQQNYENPREATGRIVCANCHLANKPVDIEVPQTVLPDTVFEAVVKIPYDMQLKQVLANGKKGALNVGAVLILPEGFELAPP : 121
Ici : MQTRNTFFWIKEQIT-----------RFISASLMIYIITRTSISSAYPIFAQQGYENPREATGRIVCANCHLANKPVDIEVPQAVLPDTVFEAVVRIPYDMQLKQVLANGKKGALNVGAVLILPEGFELAPS : 121
Isc : MQTRNTLFWIKEQITRFIRKTFFWIKRFISASLMIYIITRTSISSAYPIFAQQGYENPREATGRIVCANCHLANKPVDIDFFQA------------------------------------------------ : 84

 140 * 160 * 180 * 200 * 220 * 240 * 260
Ikl : DRISPEIKEKIGNLSFQSYRPNKKNILVVGPVPGQKYSEITFPILSPSPATKKDVNFLKYPIYVGGNRGRGQIYPDGSKSNNTVYTATAAGVVSKILRKEKGGYEITITDASDGRQVVDIIPPGPELLVSEG : 253
Ili : DRISPEIKEKIGNLSFQSYRPNKKNILVVGPVPGQKYSEITFPILSPSPATKKDVNFLKYPIYVGGNRGRGQIYPDGSKSNNTVYTATAAGVVSKIVRKEKGGYEITITDASDGRQVVDIIPPGPELLVSEG : 253
Ipe : DRISPEIKEKIGNLSFQSYRPNKKNILVVGPVPGQKYSEITFPILSPSPATKKDVNFLKYPIYVGGNRGRGQIYPDGSKSNNTVYTATAAGVVSKIVRKEKGGYEITITDASDGRQVVDIIPPGPELLVSEG : 253
Ise : DRISPEMKEKIGNLSFQSYRPNKKNILVVGPVPGQKYSEITFPILSPSPATKKDVNFLKYPIYVGGNRGRGQIYPDGSKSNNTVYTATAAGVVSKIVRKEKGGYEITITDASDGRQVVDIIPPGPELLVSEG : 253
Ath : DRISPEMKEKIGNLSFQNYRPNKKNILVIGPVPGQKYSEITFPILAPDPATNKDVHFLKYPIYVGGNRGRGQIYPDGSKSNNTVYNATAGGIISKILRKEKGGYEITIVDASNGREVIDIIPRGLELLVSEG : 253
Ici : DRISPEMKEKIGNLSFQSYRPNKKNILVVGPVPGQKYSEITFPILSPSPATKKDVNFLKYPIYVGGNRGRGQIYPDGSKSNNTVYTATAAGVVSKIVRKEKGGYEISITDASDGRQVVDIIPPGPELLVSEG : 253
Isc : ------------------------------------------------------------------------------------------------------------------------------------ : -

 * 280 * 300 * 320 *
Ikl : EFLKLDQPLTSNPNVGGFGQGDAEIVLQDPLRVQGLLFFLASVILAQIFLVLKKKQFEKVQLSEMNF : 320
Ili : EFLKLDQPLTSNPNVGGFGQGDAEIVLQDPLRVQGLLFFLASVILAQIFLVLKKKQFEKVQLSEMNF : 320
Ipe : EFLKLDQPLTSNPNVGGFGQGDAEIVLQDPLRVQGLLFFLASVILAQIFLVLKKKQFEKVQLSEMNF : 320
Ise : EFIKLDQPLTSNPNVGGFGQGDAEIVLQDPLRVQGLLFFLASVILAQIFLVLKKKQFEKVQLSEMNF : 320
Ath : ESIKLDQPLTSNPNVGGFGQGDAEIVLQDPLRVQGLLFFLGSVVLAQIFLVLKKKQFEKVQLSEMNF : 320
Ici : EFLKLDQPLTSNPNVGGFGQGDAEIVLQDPLRVQGLLFFLASVILAQIFLVLKKKQFEKVQLSEMNF : 320
Isc : ------------------------------------------------------------------- : -

**Figure S23 Multiple sequence alignment of the deduced amino acid sequences of PETA protein.** The origins of the protein sequences used in this alignment are from: *Arabidopsis thaliana* (Ath), *Iodes klaineana* (Ikl), *Iodes cirrhosa* (Ici), *Iodes seretii* (Ise), *Iodes scandens* (Isc), *Iodes perrieri* (Ipe) and *Iodes liberica* (Ili). The amino acid numbers for each sequence are indicated on the right. In the sequence alignment, identical residues are shown with a black background, and similar residues are shown with a gray background.

* 20 * 40 * 60 * 80 * 100 * 120 *
Ikl : --KVYDWFEERLEIQAIADDITSKYVPPHVNIFYCLGGITLTCFLVQVATGFAMTFYYR------PTVTEAFASVQYIMTEANFGWLIRSVHRWSASMMVLMMILHVFRVYLTGGFKKPRELTWVTGVVLAV : 124
Ise : --KVYDWFEERLEIQAIADDITSKYVPPHVNIFYCLGGITLTCFLVQVATGFAMTFYYRPTITEAPTITEAFASVQYIMTEANFGWLIRSVHRWSASMMVLMMILHVFRVYLTGGFKKPRELTWVTGVVLAV : 130
Ili : --KVYDWFEERLEIQAIADDITSKYVPPHVNIFYCLGGITLTCFLVQVATGFAMTFYYR------PTVTEAFASVQYIMTEANFGWLIRSVHRWSASMMVLMMILHVFRVYLTGGFKKPRELTWVTGVVLAV : 124
Isc : --KVYDWFEERLEIQSIADDITSKYVPPHVNIFYCLGGITLTCFLVQVATGFAMTFYYR------PTVTEAFASVQYIMTEANFGWLIRSVHRWSASMMVLMMILHVFRVYLTGGFKKPRELTWVTGVVLAV : 124
Ici : -MKVYDWFEERLEIQSIADDITSKYVPPHVNIFYCLGGITLTCFLVQVATGFAMTFYYR------PTVTEAFASVQYIMTEANFGWLIRSVHRWSASMMVLMMILHVFRVYLTGGFKKPRELTWVTGVVLAV : 125
Ipe : --KVYDWFEERLEIQAIADDITSKYVPPHVNIFYCLGGITLTCFLVQVATGFAMTFYYR------PTVTEAFASVQYIMTEANFGWLIRSVHRWSSSMMVLMMILHVFRVYLTGGFKKPRELTWVTGVVLGV : 124
Ath : MSKVYDWFEERLEIQAIADDITSKYVPPHVNIFYCLGGITLTCFLVQVATGFAMTFYYR------PTVTEAFASVQYIMTEANFGWLIRSVHRWSASMMVLMMILHVFRVYLTGGFKKPRELTWVTGVVLGV : 126

 140 * 160 * 180 * 200 * 220
Ikl : LTASFGVTGYSLPWDQIGYWAVKIVTGVPEAIPVIGSPLVELLRGSASVGQSTLTRFYSLHTFVLPLLTAVFMLMHFPMIRKQGISGPL : 213
Ise : LTASFGVTGYSLPWDQIGYWAVKIVTGVPEAIPVIGSPLVELLRGSASVGQSTLTRFYSLHTFVLPLLTAVFMLMHFPMIRKQGISGPL : 219
Ili : LTASFGVTGYSLPWDQIGYWAVKIVTGVPEAIPVIGSPLVELLRGSASVGQSTLTRFYSLHTFVLPLLTAVFMLMHFPMIRKQGISGPL : 213
Isc : LTASFGVTGYSLPWDQIGYWAVKIVTGVPEAIPVIGSPLVELLRGSASVGQSTLTRFYSLHTFVLPLLTAVFMLMHFPMIRKQGISGPL : 213
Ici : LTASFGVTGYSLPWDQIGYWAVKIVTGVPEAIPVIGSPLVELLRGSASVGQATLTRFYSLHTFVLPLLTAVFMLMHFPMIRKQGISGPL : 214
Ipe : LTASFGVTGYSLPWDQIGYWAVKIVTGVPEAIPVIGSPLVELLRGSASVGQSTLTRFYSLHTFVLPLLTAVFMLMHFPMIRKQGISGPL : 213
Ath : LTASFGVTGYSLPWDQIGYWAVKIVTGVPDAIPVIGSPLVELLRGSASVGQSTLTRFYSLHTFVLPLLTAVFMLMHFLMIRKQGISGPL : 215

**Figure S24 Multiple sequence alignment of the deduced amino acid sequences of PETB protein.** The origins of the protein sequences used in this alignment are from: *Arabidopsis thaliana* (Ath), *Iodes klaineana* (Ikl), *Iodes cirrhosa* (Ici), *Iodes seretii* (Ise), *Iodes scandens* (Isc), *Iodes perrieri* (Ipe) and *Iodes liberica* (Ili). The amino acid numbers for each sequence are indicated on the right. In the sequence alignment, identical residues are shown with a black background, and similar residues are shown with a gray background.

* 20 * 40 * 60 * 80 * 100 * 120 *
Isc : ---TKKPDLNDPVLRAKLAKGMGHNYYGEPAWPNDLLYIFPVVILGTIACNVGLAVLEPSMIGEPADPFATPLEILPEWYFFPVFQILRTVPNKLLGVLLMVSVPAGLLTVPFLENVNKFQNPFRRPVATTI : 129
Ath : MGVTKKPDLNDPVLRAKLAKGMGHNYYGEPAWPNDLLYIFPVVILGTIACNVGLAVLEPSMIGEPADPFATPLEILPEWYFFPVFQILRTVPNKLLGVLLMVSVPAGLLTVPFLENVNKFQNPFRRPVATTV : 132
Ipe : ---TKKPDLNDPVLRAKLAKGMGHNYYGEPAWPNDLLYIFPVVILGTIACNVGLAVLEPSMIGEPADPFATPLEILPEWYFFPVFQILRTVPNKLLGVLLMVSVPAGLLIVPFLENVNKFQNPFRRPVATTI : 129
Ise : ---TKKPDLNDPVLRAKLAKGMGHNYYGEPAWPNDLLYIFPVVILGTIACNVGLAVLEPSMIGEPADPFATPLEILPEWYFFPVFQILRTVPNKLLGVLLMVSVPAGLLTVPFLENVNKFQNPFRRPVATTI : 129
Ikl : ---TKKPDLNDPVLRAKLAKGMGHNYYGEPAWPNDLLYIFPVVILGTIACNVGLAVLEPSMIGEPADPFATPLEILPEWYFFPVFQILRTVPNKLLGVLLMVSVPAGLLTVPFLENVNKFQNPFRRPVATTI : 129
Ici : MS-TKKPDLNDPVLRAKLAKGMGHNYYGEPAWPNDLLYIFPVVILGTIACNVGLAVLEPSMIGEPADPFATPLEILPEWYFFPVFQILRTVPNKLLGVLLMVSVPAGLLTVPFLENVNKFQNPFRRPVATTI : 131
Ili : -------------------------YYGEPAWPNDLLYIFPVVILGTIACNVGLAVLEPSMIGDPADPFATPLEILPEWYFFPVFQILRTVPNKLLGVLLMVSVPAGLLTVPFLENVNKFQNPFRRPVATTI : 107

 140 * 160
Isc : FLIGTAVALWLGIGATLPIEKSI----- : 152
Ath : FLIGTAAALWLGIGATLPIDKSLTLGLF : 160
Ipe : FLIGTAVALWLGIGATLPIEKSLTLGLF : 157
Ise : FLIGTAVALWLGIGATLPIEKSLTLGLF : 157
Ikl : FLIGTAVALWLGIGATLPIEKSLTLGLF : 157
Ici : FLIGTAVALWLGIGATLPIEKSLTLGLF : 159
Ili : FLIGTAVALWLGIGATLPIEKSLTLGLF : 135

**Figure S25 Multiple sequence alignment of the deduced amino acid sequences of PETD protein.** The origins of the protein sequences used in this alignment are from: *Arabidopsis thaliana* (Ath), *Iodes klaineana* (Ikl), *Iodes cirrhosa* (Ici), *Iodes seretii* (Ise), *Iodes scandens* (Isc), *Iodes perrieri* (Ipe) and *Iodes liberica* (Ili). The amino acid numbers for each sequence are indicated on the right. In the sequence alignment, identical residues are shown with a black background, and similar residues are shown with a gray background.

* 20 * 40 *
Ipe : ----NLKGIVLGLIPITLAGLFVTAYLQY-----------------RRGDQLD- : 32
Ath : MIEVFLFGIVLGLIPITLAGLFVTAYLQY-----------------RRGDQLDF : 37
Ikl : MIEVSLFGIVLGLIPITLAGLFVTAYLQY-----------------RRGDQLD- : 36
Ici : MIEVSLFGIVLGLIPITLAGLFVTAYLQY-----------------RRGDQLDL : 37
Isc : MIEVSLFGIVLGLIPITLAGLFVTAYLQY-----------------RRGDQ--- : 34
Ise : MIEVSLFGIVLGLIPITLAGLFVTAYLQY-----------------RRGDQ--- : 34
Ili : MIEVSLFGIVLGLIPITLAGLFVTAYLQYRLIPITFWLDYSAYLQYRRGDQ--- : 51
**Figure S26 Multiple sequence alignment of the deduced amino acid sequences of PETG protein.** The origins of the protein sequences used in this alignment are from: *Arabidopsis thaliana* (Ath), *Iodes klaineana* (Ikl), *Iodes cirrhosa* (Ici), *Iodes seretii* (Ise), *Iodes scandens* (Isc), *Iodes perrieri* (Ipe) and *Iodes liberica* (Ili). The amino acid numbers for each sequence are indicated on the right. In the sequence alignment, identical residues are shown with a black background, and similar residues are shown with a gray background.

* 20 *
Isc : MPTITSYFGFLLA------------------ : 13
Ath : MPTITSYFGFLLAALTITSVLFIGLSKIRLI : 31
Ici : MPTITSYFGFLLAALTITSALFIGLSKIRLI : 31
Ise : MPTITSYFGFLLAALTITSALFIGLSKIRLI : 31
Ili : MPTITSYFGFLLAALTITSALFIGLSKIRLI : 31
Ikl : MPTITSYFGFLLAALTITSALFIGLSKIRLI : 31
Ipe : MPTLTSYFGFLLAALTITSALFIGLSKIRLI : 31

**Figure S27 Multiple sequence alignment of the deduced amino acid sequences of PETL protein.** The origins of the protein sequences used in this alignment are from: *Arabidopsis thaliana* (Ath), *Iodes klaineana* (Ikl), *Iodes cirrhosa* (Ici), *Iodes seretii* (Ise), *Iodes scandens* (Isc), *Iodes perrieri* (Ipe) and *Iodes liberica* (Ili). The amino acid numbers for each sequence are indicated on the right. In the sequence alignment, identical residues are shown with a black background, and similar residues are shown with a gray background.

* 20 * 40
Ikl : MDIVSLAWAALMVVFTFSLSLVVWGRSGL----------- : 29
Ipe : MDIVSLAWAALMVVFTFSLSLVVWGRSGL----------- : 29
Isc : MDIVSLAWAALMVVFTFSLSLVVWGRSGL----------- : 29
Ise : MDIVSLAWAALMVVFTFSLSLVVWGRSGL----------- : 29
Ili : MDIVSLAWAALMVVFTFSLSLVVWGRSGLNLSLPINGRSG : 40
Ici : MDIVSLAWAALMVVFTFSLSLVVWGRSGL----------- : 29
Ath : MDIVSLAWAALMVVFTFSLSLVVWGRSGL----------- : 29

**Figure S28 Multiple sequence alignment of the deduced amino acid sequences of PETN protein.** The origins of the protein sequences used in this alignment are from: *Arabidopsis thaliana* (Ath), *Iodes klaineana* (Ikl), *Iodes cirrhosa* (Ici), *Iodes seretii* (Ise), *Iodes scandens* (Isc), *Iodes perrieri* (Ipe) and *Iodes liberica* (Ili). The amino acid numbers for each sequence are indicated on the right. In the sequence alignment, identical residues are shown with a black background, and similar residues are shown with a gray background.

* 20 * 40 * 60 * 80 * 100 * 120
Ikl : MIIRSPEPEVKILVDRDHIKTSFEEWARPG--------------------------------------------------------------------------------------------------- : 30
Isc : MIIRSPEPEVKILVDRDHIKTSFEEWARPG--------------------------------------------------------------------------------------------------- : 30
Ici : MIIRSPEPEVKILVDRDHIKTSFEEWARPG--------------------------------------------------------------------------------------------------- : 30
Ipe : MIIRSTEPEVKILVDRDHIKTSFEEWARPG--------------------------------------------------------------------------------------------------- : 30
Ath : MIIRSPEPEVKILVDRDPIKTSFEEWAKPG--------------------------------------------------------------------------------------------------- : 30
Ise : MIIRSPEPEVKILVDRDHIKTSFEEWARPGHFSRTIAKGPDTTTWIWNLHADAHDFDSHTSDLEEISRKVFSAHFGQLSIIFLWLSGMYFHGARFSNYEAWLSDPTHIGPSAQVVWPIVGQEILNGDVG : 129
Ili : --------------------------------------------------------------------------------------------------------------------------------- : -

 * 140 * 160 * 180 * 200 * 220 * 240 * 2
Ikl : ------------------------------------------------------------------------------------------------------------------HFSRTIAKGPDTTTW : 45
Isc : ------------------------------------------------------------------------------------------------------------------HFSRTIAKGPDTTTW : 45
Ici : ------------------------------------------------------------------------------------------------------------------HFSRTIAKGPDTTTW : 45
Ipe : ------------------------------------------------------------------------------------------------------------------HFSRTIAKGPDTTTW : 45
Ath : ------------------------------------------------------------------------------------------------------------------HFSRTIAKGPDTTTW : 45
Ise : GGFRGIQITSGLFQMWRASGITNELQLYCTAIGALVFAALMLFAGWFHYHKAAPKLAWFQDVESMLNHHLAGLLGLGSLAWAGHQIHVSLPINQFLNAGVDPKEIPLPHEFILNRFSQGLAQDP-TTRR : 257
Ili : -------------------------------------------------------------------------------------------------------------------FSQGLAQDP-TTRR : 13

 60 * 280 * 300 * 320 * 340 * 360 * 380
Ikl : IWNLHADAHDFDSHTSDLEE-ISRKVFSAHFGQLSIIFLWLSGMYFHGARFSNYEAWLSDPTHIGPSAQVVW-PIVGQEILNGDVGGGFRG-IQIT-SGLFQMWRASGITNELQLYCTAIGALVFAALM : 170
Isc : IWNLHADAHDFDSHTSDLEE-ISRKVFSAHFGQLSIIFLWLSGMYFHGARFSNYEAWLSDPTHIGPSAQVVW-PIVGQEILNGDVGGGFRG-IQIT-SGLFQMWRASGITNELQLYCTAIGALVFAALM : 170
Ici : IWNLHADAHDFDSHTSDLEE-ISRKVFSAHFGQLSIIFLWLSGMYFHGARFSNYEAWLSDPTHIGPSAQVVW-PIVGQEILNGDVGGGFRG-IQIT-SGLFQMWRASGITSELQLYCTAIGALVFAALM : 170
Ipe : IWNLHADAHDFDSHTSDLEE-ISRKVFSAHFGQLSIIFLWLSGMYFHGARFSNYEAWLSDPTHIGPSAQVVW-PIVGQEILNGDVGGGFRG-IQIT-SGLFQMWRASGITNELQLYCTAIGALVFAALM : 170
Ath : IWNLHADAHDFDSHTSDLEE-ISRKVFSAHFGQLSIIFLWLSGMYFHGARFSNYEAWLSDPTHIGPSAQVVW-PIVGQEILNGDVGGGFRG-IQIT-SGFFQIWRASGITSELQLYCTAIGALVFAALM : 170
Ise : IWFGIATAHDFESHDDITEERLYQNIFASHFGQLAIIFLWTSGNLFHVAWQGNFESWVQDPLHVRPIAHAIWDPHFGQPAVEAFTRGGALGPVNIAYSGVYQWWYTIGLRTNEDLYTGALFLLFLSAIS : 386
Ili : IWFGIATAHDFESHDDITEERLYQNIFASHFGQLAIIFLWTSGNLFHVAWQGNFESWVQDPLHVRPIAHAIWDPHFGQPAVEAFTRGGALGPVNIAYSGVYQWWYTIGLRSNEDLYTGALFLLFLSAIS : 142

* 400 * 420 * 440 * 460 * 480 * 500 *
Ikl : LFAGWFHYH-KAAPKLAWFQDVESMLNHHLAGLLGLGSLAWAGHQVHVSLPINQFLNAGVDPKEIPLPHEFILNRDLLTQLYPVFAEG---ATPFFTLNWSKYAEFLTFRGGLDPVTGGLWLTDIAHHH : 295
Isc : LFAGWFHYH-KAAPKLAWFQDVESMLNHHLAGLLGLGSLAWAGHQVHVSLPINQFLNAGVDPKEIPLPHEFILNRDLLTQLYPVFAEG---ATPFFTLNWSKYAEFLTFRGGLDPVTGGLWLTDIAHHH : 295
Ici : LFAGWFHYH-KAAPKLAWFQDVESMLNHHLAGLLGLGSLAWAGHQVHVSLPINQFLNAGVDPKEIPLPHEFILNRDLLTQLFPVFAEG---ATPFFTLNWSKYAEFLTFRGGLDPVTGGLWLTDIAHHH : 295
Ipe : LFAGWFHYH-KAAPKLAWFQDVESMLNHHLAGLLGLGSLAWAGHQVHVSLPINQFLNAGVDPKEIPLPHEFILNRDLLTQLYPVFAEG---ATPFFTLNWSKYVEFLTFRGGLDPVTGGLWLTDIVHHH : 295
Ath : LFAGWFHYH-KAAPKLAWFQDVESMLNHHLAGLLGLGSLSWAGHQVHVSLPINQFLNAGVDPKEIPLPHEFILNRDLLAQLYPSFAEG---ATPFFTLNWSKYSEFLTFRGGLDPVTGGLWLTDIAHHH : 295
Ise : LIAGWLHLQPKWKPSVSWFKNAESRLNHHLSGLFGVSSLAWTGHLVHVAIPASRGEYIRWNNFLDVLPHPQGLG-PLFTGQWNLYAQNPDSSSHLFGTSQGAGTAILTLLGGFHPQTQSLWLTDIAHHH : 514
Ili : LIAGWLHLQPKWKPSVSWFKNAESRLNHHLSGLFGVSSLAWTGHLVHVAIPASRGEYVRWNNFLDVLPHPQGLG-PLFTGQWNLYAQNPDSSSHLFGTSQGAGTAILTLLGGFHPQTQSLWLTDIAHHH : 270

 520 * 540 * 560 * 580 * 600 * 620 * 640
Ikl : LAIAILFLIAGHMYRTNWGIGHGLKDILEAHKGP--FTGQGHKGLYEILTTSWHAQLSLNLAMLGSLTIIVAHHMYAMPPYPYLATDYGTQLSLFTHHMWIGGFLIVGAAAHAAIFMVRDYDPTTRYND : 422
Isc : LAIAILFLIAGHMYRTNWGIGHGLKDILEAHKGP--FTGQGHKGLYEILTTSWHAQLSLNLAMLGSLTIIVAHHMYAMPPYPYLATDYGTQLSLFTHHMWIGGFLIVGAAAHAAIFMVRDYDPTTRYND : 422
Ici : LAIAILFLIAGHMYRTNWGIGHGLKDILEAHKGP--FTGQGHKGLYEILTTSWHAQLSLNLAMLGSLTIIVAHHMYAMPPYPYLATDYGTQLSLFTHHMWIGGFLIVGAAAHAAIFMVRDYDPTTRYND : 422
Ipe : LAIAILFLIAGHMYRTNWGIGHGLKDILEAHKGP--FTGQGHKGLYEILTTSWHAQLSLNLAMLGSLTIIVAHHMYAMPPYPYLATDYGTQLSLFTHHMWIGGFLIVGAAAHAAIFMVRDYDPTTRYND : 422
Ath : LAIAILFLIAGHMYRTNWGIGHGIKDILEAHKGP--FTGQGHKGLYEILTTSWHAQLSLNLAMLGSLTIIVAHHMYSMPPYPYLATDYATQLSLFTHHMWIGGFLIVGAAAHAAIFMVRDYDPTNRYND : 422
Ise : LAIAFIFLVAGHMYRTNFGIGHSMKDLLDAHIPPGGRLGRGHKGLYDTINNSLHFQLGLALASLGVITSLVAQHMYSLPAYAFIAQDFTTQAALYTHHQYIAGFIMTGAFAHGAIFFIRDYNPEQNEDN : 643
Ili : LAIAFIFLVAGHMYRTNFGIGHSMKDLLDAHIPPGGRLGRGHKGLYDTINNSLHFQLGLALASLGVITSLVAQHMYSLPAYAFIAQDFTTQAALYTHHQYIAGFIMTGAFAHGAIFFIRDYNPEQNEDN : 399

 * 660 * 680 * 700 * 720 * 740 * 760 *
Ikl : LLDRVLRHRDAIISHLNWACIFLGFHSFGLYIHNDTMSALGRPQDMFSDTAIQLQPVFAQWIQNTH--ALAPGATAPGATASTSLTWGGGDLVAVGGKVALLPIPLGTADFLVHHIHAFTIHVTVLILL : 549
Isc : LLDRVLRHRDAIISHLNWACIFLGFHSFGLYIHNDTMSALGRPQDMFSDTAIQLQPVFAQWIQNTH--ALAPGATAPGATTSTSLTWGGGDLVAVGGKVALLPIPLGTADFLVHHIHAFTIHVTVLILL : 549
Ici : LLDRVLRHRDAIISHLNWACIFLGFHSFGLYIHNDTMSALGRPQDMFSDTAIQLQPVFAQWIQNTH--ALAPGATAPGATTSTSLTWGGGDLVAVGGKVALLPIPLGTADFLVHHIHAFTIHVTVLILL : 549
Ipe : LLDRVLRHRDAIISHLNWACIFLGFHSFGLYIHNDTMSALGRPQDIY---------------------------------------------------------------------------------- : 469
Ath : LLDRVLRHRDAIISHLNWVCIFLGFHSFGLYIHNDTMSALGRPQDMFS--------------------------------------------------------------------------------- : 470
Ise : VLARMLDHKEAITSHLSWASLFLGFHTLGLYVHNDVMLAFGTPEKQILIEPIFAQWIQSAHGKTSYGFDVLLSSTNGPAFNAGRSIWLPGWLNAVNENSNSLFLTIGPGDFLVHHAIALGLHTTTLILV : 772
Ili : VLARMLDHKEAITSHLSWASLFLGFHTLGLYVHNDVMLAFGTPEKQILIEPIFAQWIQSAHGKTSYGFDVLLSSTNGPAFNAGRSIWLPGWLNAVNENSNSLFLTIGPGDFLVHHAIALGLHTTTLILV : 528

780 * 800 * 820 * 840 * 860 * 880 * 900
Ikl : KGVLFARSSRLIPDKANLGFRFPCDGPGRGGTCQVSAWDHVFLGLFWMYNSISVVIFHFSWKMQSDVWGNISDQGIVTHITGGNFAQSSITINGWLRDFLWAQASQVIQSYG----SSFSAYGLFFLGA : 674
Isc : KGVLFARSSRLIPDKANLGFRFPCDGPGRGGTCQVSAWDHVFLGLFWMYNSISVVIFHFSWKMQSDVWGNISDQGIVTHITGGNFAQSSITINGWLRDFLWAQASQVIQSYG----SSFSAYGLFFLGA : 674
Ici : KGVLFARSSRLIPDKANLGFRFPCDGPGRGGTCQVSAWDHVFLGLFWMYNSISVVIFHFSWKMQSDVWGNISDQGIVTHITGGNFAQSSITINGWLRDFLWAQASQVIQSYG----SSFSAYGLFFLGA : 674
Ipe : --------------------------------------------------------------------------------------------------------------------------------- : -
Ath : --------------------------------------------------------------------------------------------------------------------------------- : -
Ise : KGALDARGSKLMPDKKDFGYSFPCDGPGRGGTCDISAWDAFYLAVFWMLNTIGWVTFYWHWKHITLWQGNVS-----------QSNESSTYLMGWLRDYLWLNSSQLINGYNPFGMNSLSVWAWMFLFG : 890
Ili : KGALDARGSKLMPDKKDFGYSFPCDGPGRGGTCDISAWDAFYLAVFWMLNTIGWVTFYWHWKHITLWQGNVS-----------QFNESSTYLMGWLRDYLWLNSSQLINGYNPFGMNSLSVWAWMFLFG : 646

 * 920 * 940 * 960 * 980 * 1000 * 1020 *
Ikl : HFVWAFSLMFLFSGRGYWQELIESIVWAHNKLKVAPATQPRALSIVQGRAVGVTHYLLGGIATTWAFFLARIIAVGFSQGLAQDPTTRRIWFGIATAHDFESHDDITEERLYQNIFASHFGQLAIIFLW : 803
Isc : HFVWAFSLMFLFSGRGYWQELIESIVWAHNKLKVAPATQPRALSIVQGRAVGVTHYLLGGIATTWAFFLARIIAVGFSQGLAQDPTTRRIWFGIATAHDFESHDDITEERLYQNIFASHFGQLAIIFLW : 803
Ici : HFVWAFSLMFLFSGRGYWQELIESIVWAHNKLKVAPATQPRALSIVQGRAVGVTHYLLGGIATTWAFFLARIIAVG----------------------------------------------------- : 750
Ipe : ----------------------------------------------------------------------------FSQGLAQDPTTRRIWFGIATAHDFESHDDITEERLYQNIFASHFGQLAIIFLW : 522
Ath : --------------------------------------------------------------------------------------------------------------------------------- : -
Ise : HLVWAIGFMFLISWRGYWQ-------------------------------------------------------------------------------------------------------------- : 909
Ili : HLVWAIGFMFLISWRGYWQG---------------------------------------------------------------PDTTTWIWNLHADAHDFDSHTSDLEE-ISRKVFSAHFGQLSIIFLW : 711

 1040 * 1060 * 1080 * 1100 * 1120 * 1140 * 1160
Ikl : TSGNLFHVAWQGNFESWVQDPLHVRPIAHAIWDPHFGQPAVEAFTRGGALGPVNIAYSGVYQWWYTIGLRTNEDLYTGALFLLFLSAISLIAGWLHLQPKWKPSVSWFKNAESRLNHHLSGLFGVSSLA : 932
Isc : TSGNLFHVAWQGNFESWVQDPLHVRPIAHAIWDPHFGQPAVEAFTRGGALGPVNIAYSGVYQWWYTIGLRTNEDLYTGALFLLFLSAISLIAGWLHLQPKWKPSVSWFKNAESRLNHHLSGLFGVSSLA : 932
Ici : --------------------------------------------------------------------------------------------------------------------------------- : -
Ipe : TSGNLFHVAWQGNFESWVQDPLHVRPIAHAIWDPHFGQPAVEAFTRGGALGPVNIAYSGVYQWWYTIGLRANEDLYTGALFLLFLSAISLIAGWLHLQPKWKPSVSWFKNAESRLNHHLSGLFGVSSLA : 651
Ath : --------------------------------------------------------------------------------------------------------------------------------- : -
Ise : --------------------------------------------------------------------------------------ELIETLAWAHER-TPLANLIRWRDKPVALSIVQARLVGLAHFS : 951
Ili : LSGMYFHGARFSNYEAWLSDPTHIGPSAQVVW-PIVGQEILNGDVGGGFRGIQIT--SGLFQMWRASGITSELQLYCTAIGALVFAALMLFAGWFHYH-KAAPKLAWFQDVESMLNHHLAGLLGLGSLA : 836

* 1180 * 1200 * 1220 * 1240 * 1260 * 1280 *
Ikl : WTGHLVHVAIPASRGESVRWNNFLDVLPHPQGLGPLFTGQWNLYAQNPDSSSHLFGTSQGAGTAILTLLGGFHPQTQSLWLTDIAHHHLAIAFIFLVAGHMYRTNFGIGHSMKDLLDAHIPPGGRLGRG : 1061
Isc : WTGHLVHVAIPASRGEYVRWNNFLDVLPHPQGLGPLFTGQWNLYAQNPDSSSHLFGTSQGAGTAILTLLGGFHPQTQSLWLTDIAHHHLAIAFIFLVAGHMYRTNFGIGHSMKDLLDAHIPPGGRLGRG : 1061
Ici : --------------------------------------------------------------------------------------------------------------------------------- : -
Ipe : WTGHLVHVAIPASRGEYVRWNNFLDVLPHPQGLGPLFTGQWNLYAQNPDSSSHLFGTSQGAGTAILTLLGGFHPQTQSLWLTDIAHHHLAIAFIFLVAGHMYRTNFGIGHSMKDLLDAHIPPGGRLGRG : 780
Ath : --------------------------------------------------------------------------------------------------------------------------------- : -
Ise : VG---------------------------YIFTYAAFLIA--LYPVFAEGATPFFTLNWSKYAEFLTFRGGLDPVTGGLWLTDIAHHHLAIAILFLIAGHMYRTNWGIGHGLKDILEAHKGP--FTGQG : 1049
Ili : WAGHQVHVSLPINQFLNAGVDPKEIPLPHEFILNRDLLTQ--LYPVFAEGATPFFTLNWSKYAEFLTFLGGLDPVTGGLWLTDIAHHHLAIAILFLIAGHMYRTNWGIGHGLKDILEAHKGP--FTGQG : 961

 1300 * 1320 * 1340 * 1360 * 1380 * 1400 * 142
Ikl : HKGLYDTINNSLHFQLGLALASLGVITSLVAQHMYSLPAYAFIAQDFTTQAALYTHHQYIAGFIMTGAFAHGAIFFIRDYNPEQNEDNVLARMLDHKEAITSHLSWASLFLGFHTLGLYVHNDVMLAFG : 1190
Isc : HKGLYDTINNSLHFQLGLALASLGVITSLVAQHMYSLPAYAFIAQDFTTQAALYTHHQYIAGFIMTGAFAHGAIFFIRDYNPEQNEDNVLARMLDHKEAITSHLSWASLFLGFHTLGLYVHNDVMLAFG : 1190
Ici : --------------------------------------------------------------------------------------------------------------------------------- : -
Ipe : HKGLYDTINNSLHFQLGLALASLGVITSLVAQHMYSLPAYAFIAQDFTTQAALYTHHQYIAGFIMTGAFAHGAIFFIRDYNPEQNEDNVLARMLDHKEAITSHLSWASLFLGFHTLGLYVHNDVMLAFG : 909
Ath : --------------------------------------------------------------------------------------------------------------------------------- : -
Ise : HKGLYEILTTSWHAQLSLNLAMLGSLTIIVAHHMYAMPPYPYLATDYGTQLSLFTHHMWIGGFLIVGAAAHAAIFMVRDYDPTTRYNDLLDRVLRHRDAIISHLNWACIFLGFHSFGLYIHNDTMSALG : 1178
Ili : HKGLYEILTTSWHAQLSLNLAMLGSLTIIVAHHMYAMPPYPYLATDYGTQLSLFTHHMWIGGFLIVGAAAHAAIFMVRDYDPTTRYNDLLDRVLRHRDAIISHLNWACIFLGFHSFGLYIHNDTMSALG : 1090

 0 * 1440 * 1460 * 1480 * 1500 * 1520 * 1540
Ikl : TP-----EKQILIEPIFAQWIQSAHGKTSYGFDVLLSSTNGPAFNAGRSI-WLPGWLNAVNENSNSLFLTIGPGDFLVHHAIALGLHTTTLILVKGALDARGSKLMPDKKDFGYSFPCDGPGRGGTCDI : 1313
Isc : TP-----EKQILIEPIFAQWIQSAHGKTSYGFDVLLSSTNGPAFNAGRSI-WLPGWLNAVNENSNSLFLTIGPGDFLVHHAIALGLHTTTLILVKGALDARGSKLMPDKKDFGYSFPCDGPGRGGTCDI : 1313
Ici : --------------------------------------------------------------------------------------------------------------------------------- : -
Ipe : TP-----EKQILIEPIFAQWIQSAHGKTSYGFDVLLSSTNGPAFNAGRSI-WLPGWLNAVNENSNSLFLTIGPGDFLVHHAIALGLHTTTLILVKGALDARGSKLMPDKKDFGYSFPCDGPGRGGTCDI : 1032
Ath : -------DTAIQLQPVFAQWIQNTH--------ALAPGVTAPGETASTSLTWGGGELVAVGGKVALLPIPLGTADFLVHHIHAFTIHVTVLILLKGVLFARSSRLIPDKANLGFRFPCDGPGRGGTCQV : 584
Ise : RPQDMFSDTAIQLQPVFAQWIQNTH--------ALAPGATAPGATASTSLTWGGGDLVAVGGKVALLPIPLGTADFLVHHIHAFTIHVTVLILLKGVLFARSSRLIPDKANLGFRFPCDGPGRGGTCQV : 1299
Ili : RPQDMFSDTAIQLQPVFAQWIQNTH--------ALAPGATAPGATASTSLTWGGGDLVAVGGKVALLPIPLGTADFLVHHIHAFTIHVTVLILLKGVLFARSSRLIPDKANLGFRFPCDGPGRGGTCQV : 1211

* 1560 * 1580 * 1600 * 1620 * 1640 * 1660 * 1
Ikl : SAWDAFYLAVFWMLNTIGWVTFYWHWKHITLWQGNVS-----------QFNESSTYLMGWLRDYLWLNSSQLINGYNPFGMNSLSVWAWMFLFGHLVWAIGFMFLISWRGYWQELIETLAWAHERTPLA : 1431
Isc : SAWDAFYLAVFWMLNTIGWVTFYWHWKHITLWQGNVS-----------QFNESSTYLMGWLRDYLWLNSSQLINGYNPFGMNSLSVWAWMFLFGHLVWAIGFMFLISWRGYWQELIETLAWAHERTPLA : 1431
Ici : --------------------------------------------------------------------------------------------------------------------------------- : -
Ipe : SAWDAFYLAVFWMLNTIGWVTFYWHWKHITLWQGNVS-----------QFNESSTYLMGWLRDYLWLNSSQLINGYNPFGMNSLSVWAWMFLFGHLVWATGFMFLISWRGYWQELIETLAWAHERTPLA : 1150
Ath : SAWDHVFLGLFWMYNAISVVIFHFSWKMQSDVWGSISDQGVVTHITGGNFAQSSITINGWLRDFLWAQASQVIQSYG----SSLSAYGLFFLGAHFVWAFSLMFLFSGRGYWQELIESIVWAHNKLKVA : 709
Ise : SAWDHVFLGLFWMYNSISVVIFHFSWKMQSDVWGNISDQGIVTHITGGNFAQSSITINGWLRDFLWAQASQVIQSYG----SSFSAYGLLFLGAHFVWAFSLMFLFSGRGYWQELIESIVWAHNKLKVA : 1424
Ili : SAWDHVFLGLFWMYNSISVVIFHFSWKMQSDVWGNISDQGIVTHITGGNFAQSSITINGWLRDFLWAQASQVIQSYG----SSFSAYGLFFLGAHFVWAFSLMFLFSGRGYWQELIESIVWAHNKLKVA : 1336

 680 * 1700 * 1720 * 1740 * 1760 * 1780 * 1800
Ikl : NLIRWRDKPVALSIVQARLVGLAHFSVGYIFTYAAFLIA------------------------------------------------------------------------------------------ : 1470
Isc : NLIRWRDKPVALSIVQARLVGLAHFSVGYIFTYAAFLIA------------------------------------------------------------------------------------------ : 1470
Ici : --------------------------------------------------------------------------------------------------------------------------------- : -
Ipe : NLIRWRDKPVALSIVQARLVGLAHFSVGYIFTYAAFLIAMFEDTAIQLQPVFASSSLGIQNTHALAPGATAPGATASTSLTWGGGDLVAVGGKVALLPIPLGTADFLVHHIHAFTIHVTVLILLKGVLF : 1279
Ath : PAT----QPRALSIIQGRAVGVTHYLLGGIATTWAFFLARIIAVG------------------------------------------------------------------------------------ : 750
Ise : PAT----QPRALSIVQGRAVGVTHYLLGGIATTWAFFLARIIAVG------------------------------------------------------------------------------------ : 1465
Ili : PAT----QPRALSIVQGRAVGVTHYLLGGIATTWAFFLARIIAVG------------------------------------------------------------------------------------ : 1377

 * 1820 * 1840 * 1860 * 1880 * 1900 * 1920 *
Ikl : --------------------------------------------------------------------------------------------------------------------------------- : -
Isc : --------------------------------------------------------------------------------------------------------------------------------- : -
Ici : --------------------------------------------------------------------------------------------------------------------------------- : -
Ipe : ARSSRLIPDKANLGFRFPCDGPGRGGTCQVSAWDHVFLGLFWMYNSISVVIFHFSWKMQSDVWGNISDQGIVTHITGGNFAQSSITINGWLRDFLWAQASQVIQSYGSSFSAYGLFFLGAHFVWAFSLM : 1408
Ath : --------------------------------------------------------------------------------------------------------------------------------- : -
Ise : --------------------------------------------------------------------------------------------------------------------------------- : -
Ili : --------------------------------------------------------------------------------------------------------------------------------- : -

1940 * 1960 * 1980 * 2000
Ikl : ------------------------------------------------------------------- : -
Isc : ------------------------------------------------------------------- : -
Ici : ------------------------------------------------------------------- : -
Ipe : FLFSGRGYWQELIESIVWAHNKLKVAPATQPRALSIVQGRAVGVTHYLLGGIATTWAFFLARIIAVG : 1475
Ath : ------------------------------------------------------------------- : -
Ise : ------------------------------------------------------------------- : -
Ili : ------------------------------------------------------------------- : -

**Figure S29 Multiple sequence alignment of the deduced amino acid sequences of PSAA protein.** The origins of the protein sequences used in this alignment are from: *Arabidopsis thaliana* (Ath), *Iodes klaineana* (Ikl), *Iodes cirrhosa* (Ici), *Iodes seretii* (Ise), *Iodes scandens* (Isc), *Iodes perrieri* (Ipe) and *Iodes liberica* (Ili). The amino acid numbers for each sequence are indicated on the right. In the sequence alignment, identical residues are shown with a black background, and similar residues are shown with a gray background.

* 140 * 160 * 180 * 200 * 220 * 240 * 2
Ikl : RTNEDLYTGALFLLFLSAISLIAGWLHLQPKWKPSVSWFKNAESRLNHHLSGLFGVSSLAWTGHLVHVAIPASRGESVRWNNFLDVLPHPQGLGPLFTGQWNLYAQNPDSSSHLFGTSQGAGTAILTLL : 258
Ise : RTNEDLYTGALFLLFLSAISLIAGWLHLQPKWKPSVSWFKNAESRLNHHLSGLFGVSSLAWTGHLVHVAIPASRGEYIRWNNFLDVLPHPQGLGPLFTGQWNLYAQNPDSSSHLFGTSQGAGTAILTLL : 258
Ili : RSNEDLYTGALFLLFLSAISLIAGWLHLQPKWKPSVSWFKNAESRLNHHLSGLFGVSSLAWTGHLVHVAIPASRGEYVRWNNFLDVLPHPQGLGPLFTGQWNLYAQNPDSSSHLFGTSQGAGTAILTLL : 258
Isc : RTNEDLYTGALFLLFLSAISLIAGWLHLQPKWKPSVSWFKNAESRLNHHLSGLFGVSSLAWTGHLVHVAIPASRGEYVRWNNFLDVLPHPQGLGPLFTGQWNLYAQNPDSSSHLFGTSQGAGTAILTLL : 258
Ici : RTNEDLYTGALFLLFLSAISLIAGWLHLQPKWKPSVSWFKNAESRLNHHLSGLFGVSSLAWTGHLVHVAIPASRGEYVRWNNFLDVLPHPQGLGPLFTGQWNLYAQNPDSSSHLFGTSQGAGTAILTLL : 258
Ipe : RANEDLYTGALFLLFLSAISLIAGWLHLQPKWKPSVSWFKNAESRLNHHLSGLFGVSSLAWTGHLVHVAIPASRGEYVRWNNFLDVLPHPQGLGPLFTGQWNLYAQNPDSSSHLFGTSQGAGTAILTLL : 258
Ath : RTNEDLYTGALFLLFLSALSLIGGWLHLQPKWKPRVSWFKNAESRLNHHLSGLFGVSSLAWTGHLVHVAIPASRGEYVRWNNFLNVLPHPQGLGPLFTGQWNLYAQNPDSSSHLFGTSQGSGTAILTLL : 258

 60 * 280 * 300 * 320 * 340 * 360 * 380
Ikl : GGFHPQTQSLWLTDIAHHHLAIAFIFLVAGHMYRTNFGIGHSMKDLLDAHIPPGGRLGRGHKGLYDTINNSLHFQLGLALASLGVITSLVAQHMYSLPAYAFIAQDFTTQAALYTHHQYIAGFIMTGAF : 387
Ise : GGFHPQTQSLWLTDIAHHHLAIAFIFLVAGHMYRTNFGIGHSMKDLLDAHIPPGGRLGRGHKGLYDTINNSLHFQLGLALASLGVITSLVAQHMYSLPAYAFIAQDFTTQAALYTHHQYIAGFIMTGAF : 387
Ili : GGFHPQTQSLWLTDIAHHHLAIAFIFLVAGHMYRTNFGIGHSMKDLLDAHIPPGGRLGRGHKGLYDTINNSLHFQLGLALASLGVITSLVAQHMYSLPAYAFIAQDFTTQAALYTHHQYIAGFIMTGAF : 387
Isc : GGFHPQTQSLWLTDIAHHHLAIAFIFLVAGHMYRTNFGIGHSMKDLLDAHIPPGGRLGRGHKGLYDTINNSLHFQLGLALASLGVITSLVAQHMYSLPAYAFIAQDFTTQAALYTHHQYIAGFIMTGAF : 387
Ici : GGFHPQTQSLWLTDIAHHHLAIAFIFLVAGHMYRTNFGIGHSMKDLLDAHIPPGGRLGRGHKGLYDTINNSLHFQLGLALASLGVITSLVAQHMYSLPAYAFIAQDFTTQAVLYTHHQYIAGFIMTGAF : 387
Ipe : GGFHPQTQSLWLTDIAHHHLAIAFIFLVAGHMYRTNFGIGHSMKDLLDAHIPPGGRLGRGHKGLYDTINNSLHFQLGLALASLGVITSLVAQHMYSLPAYAFIAQDFTTQAALYTHHQYIAGFIMTGAF : 387
Ath : GGFHPQTQSLWLTDMAHHHLAIAILFLIAGHMYRTNFGIGHSIKDLLEAHIPPGGRLGRGHKGLYDTINNSIHFQLGLALASLGVITSLVAQHMYSLPAYAFIAQDFTTQAALYTHHQYIAGFIMTGAF : 387

 * 400 * 420 * 440 * 460 * 480 * 500 *
Ikl : AHGAIFFIRDYNPEQNEDNVLARMLDHKEAITSHLSWASLFLGFHTLGLYVHNDVMLAFGTPEKQILIEPIFAQWIQSAHGKTSYGFDVLLSSTNGPAFNAGRSIWLPGWLNAVNENSNSLFLTIGPGD : 516
Ise : AHGAIFFIRDYNPEQNEDNVLARMLDHKEAITSHLSWASLFLGFHTLGLYVHNDVMLAFGTPEKQILIEPIFAQWIQSAHGKTSYGFDVLLSSTNGPAFNAGRSIWLPGWLNAVNENSNSLFLTIGPGD : 516
Ili : AHGAIFFIRDYNPEQNEDNVLARMLDHKEAITSHLSWASLFLGFHTLGLYVHNDVMLAFGTPEKQILIEPIFAQWIQSAHGKTSYGFDVLLSSTNGPAFNAGRSIWLPGWLNAVNENSNSLFLTIGPGD : 516
Isc : AHGAIFFIRDYNPEQNEDNVLARMLDHKEAITSHLSWASLFLGFHTLGLYVHNDVMLAFGTPEKQILIEPIFAQWIQSAHGKTSYGFDVLLSSTNGPAFNAGRSIWLPGWLNAVNENSNSLFLTIGPGD : 516
Ici : AHGAIFFIRDYNPEQNEDNVLARMLDHKEAITSHLSWASLFLGFHTLGLYVHNDVMLAFGTPEKQILIEPIFAQWIQSAHGKTSYGFDVLLSSTNGPAFNAGRSIWLPGWLNAVNENSNSLFLTIGPGD : 516
Ipe : AHGAIFFIRDYNPEQNEDNVLARMLDHKEAITSHLSWASLFLGFHTLGLYVHNDVMLAFGTPEKQILIEPIFAQWIQSAHGKTSYGFDVLLSSTNGPAFNAGRSIWLPGWLNAVNENSNSLFLTIGPGD : 516
Ath : AHGAIFFIRDYNPEQNEDNVLARMLDHKEAIISHLSWASLFLGFHTLGLYVHNDVMLAFGTPEKQILIEPIFAQWIQSAHGKTSYGFDVLLSSTSGPAFNAGRSIWLPGWLNAINENSNSLFLTIGPGD : 516

520 * 540 * 560 * 580 * 600 * 620 * 640
Ikl : FLVHHAIALGLHTTTLILVKGALDARGSKLMPDKKDFGYSFPCDGPGRGGTCDISAWDAFYLAVFWMLNTIGWVTFYWHWKHITLWQGNVSQFNESSTYLMGWLRDYLWLNSSQLINGYNPFGMNSLSV : 645
Ise : FLVHHAIALGLHTTTLILVKGALDARGSKLMPDKKDFGYSFPCDGPGRGGTCDISAWDAFYLAVFWMLNTIGWVTFYWHWKHITLWQGNVSQSNESSTYLMGWLRDYLWLNSSQLINGYNPFGMNSLSV : 645
Ili : FLVHHAIALGLHTTTLILVKGALDARGSKLMPDKKDFGYSFPCDGPGRGGTCDISAWDAFYLAVFWMLNTIGWVTFYWHWKHITLWQGNVSQFNESSTYLMGWLRDYLWLNSSQLINGYNPFGMNSLSV : 645
Isc : FLVHHAIALGLHTTTLILVKGALDARGSKLMPDKKDFGYSFPCDGPGRGGTCDISAWDAFYLAVFWMLNTIGWVTFYWHWKHITLWQGNVSQFNESSTYLMGWLRDYLWLNSSQLINGYNPFGMNSLSV : 645
Ici : FLVHHAIALGLHTTTLILVKGALDARGSKLMPDKKDFGYSFPCDGPGRGGTCDISAWDAFYLAVFWMLNTIGWVTFYWHWKHITLWQGNVSQFNESSTYLMGWLRDYLWLNSSQLINGYNPFGMNSLSV : 645
Ipe : FLVHHAIALGLHTTTLILVKGALDARGSKLMPDKKDFGYSFPCDGPGRGGTCDISAWDAFYLAVFWMLNTIGWVTFYWHWKHITLWQGNVSQFNESSTYLMGWLRDYLWLNSSQLINGYNPFGMNSLSV : 645
Ath : FLVHHAIALGLHTTTLILVKGALDARGSKLMPDKKDFGYSFPCDGPGRGGTCDISAWDAFYLAVFWMLNTIGWVTFYWHWKHITLWQGNVSQFNESSTYLMGWLRDYLWLNSSQLINGYNPFGMNSLSV : 645

 * 660 * 680 * 700 * 720 * 740 * 760 *
Ikl : WAWMFLFGHLVWAIGFMFLISWRGYWQELIETLAWAHERTPLANLIRWRDKPVALSIVQARLVGLAHFSVGYIFTYAAFLIASTSGKFGFSRTIAKGPDTTTWIWNLHADAHDFDSHTSDLEEISRKVF : 774
Ise : WAWMFLFGHLVWAIGFMFLISWRGYWQELIETLAWAHERTPLANLIRWRDKPVALSIVQARLVGLAHFSVGYIFTYAAFLIASTSGKFG---------------------------------------- : 734
Ili : WAWMFLFGHLVWAIGFMFLISWRGYWQELIETLAWAHERTPLANLIRWRDKPVALSIVQARLVGLAHFSVGYIFTYAAFLIASTSGKFG---------------------------------------- : 734
Isc : WAWMFLFGHLVWAIGFMFLISWRGYWQELIETLAWAHERTPLANLIRWRDKPVALSIVQARLVGLAHFSVGYIFTYAAFLIASTSGKFGFSRTIAKGPDTTTWIWNLHADAHDFDSHTSDLEEISRKVF : 774
Ici : WAWMFLFGHLVWATGFMFLISWRGYWQELIETLAWAHERTPLANLIRWRDKPVALSIVQARLVGLAHFSVGYIFTYAAFLIASTSGKFG---------------------------------------- : 734
Ipe : WAWMFLFGHLVWATGFMFLISWRGYWQELIETLAWAHERTPLANLIRWRDKPVALSIVQARLVGLAHFSVGYIFTYAAFLIASTSGKFGFSRTIAKGPDTTTWIWNLHADAHDFDSHTSDLEEISRKVF : 774
Ath : WAWMFLFGHLVWATGFMFLISWRGYWQELIETLAWAHERTPLANLIRWKDKPVALSIVQARLVGLAHFSVGYIFTYAAFLIASTSGKFG---------------------------------------- : 734

 780 * 800 * 820 * 840 * 860 * 880 * 900
Ikl : SAHFGQLSIIFLWLSGMYFHGARFSNYEAWLSDPTHIGPSAQVVWPIVGQEILNGDVGGGFRGIQITSGLFQMWRASGITNELQLYCTAIGALVFAALMLFAGWFHYHKAAPKLAWFQDVESMLNHHLA : 903
Ise : --------------------------------------------------------------------------------------------------------------------------------- : -
Ili : --------------------------------------------------------------------------------------------------------------------------------- : -
Isc : SAHFGQLSIIFLWLSGMYFHGARFSNYEAWLSDPTHIGPSAQVVWPIVGQEILNGDVGGGFRGIQITSGLFQMWRASGITNELQLYCTAIGALVFAALMLFAGWFHYHKAAPKLAWFQDVESMLNHHLA : 903
Ici : --------------------------------------------------------------------------------------------------------------------------------- : -
Ipe : SAHFGQLSIIFLWLSGMYFHGARFSNYEAWLSDPTHIGPSAQVVWPIVGQEILNGDVGGGFRGIQITSGLFQMWRASGITNELQLYCTAIGALVFAALMLFAGWFHYHKAAPKLAWFQDVESMLNHHLA : 903
Ath : --------------------------------------------------------------------------------------------------------------------------------- : -

* 920 * 940 * 960 * 980 * 1000 * 1020 *
Ikl : GLLGLGSLAWAGHQVHVSLPINQFLNAGVDPKEIPLPHEFILNRDLLTQLYPVFAEGATPFFTLNWSKYAEFLTFRGGLDPVTGGLWLTDIAHHHLAIAILFLIAGHMYRTNWGIGHGLKDILEAHKGP : 1032
Ise : -------------------------------------------------------EGATPFFTLNWSKYAEFLTFRGGLDPVTGGLWLTDIAHHHLAIAILFLIAGHMYRTNWGIGHGLKDILEAHKGP : 808
Ili : -----------GHQVHVSLPINQFLNAGVDPKEIPLPHEFILNRDLLTQLYPVFAEGATPFFTLNWSKYAEFLTFLGGLDPVTGGLWLTDIAHHHLAIAILFLIAGHMYRTNWGIGHGLKDILEAHKGP : 852
Isc : GLLGLGSLAWAGHQVHVSLPINQFLNAGVDPKEIPLPHEFILNRDLLTQLYPVFAEGATPFFTLNWSKYAEFLTFRGGLDPVTGGLWLTDIAHHHLAIAILFLIAGHMYRTNWGIGHGLKDILEAHKGP : 1032
Ici : --------------------------------------------------------------------------------------------------------------------------------- : -
Ipe : GLLGLGSLAWAGHQVHVSLPINQFLNAGVDPKEIPLPHEFILNRDLLTQLYPVFAEGATPFFTLNWSKYVEFLTFRGGLDPVTGGLWLTDIVHHHLAIAILFLIAGHMYRTNWGIGHGLKDILEAHKGP : 1032
Ath : --------------------------------------------------------------------------------------------------------------------------------- : -

 1040 * 1060 * 1080 * 1100 * 1120 * 1140 * 1160
Ikl : FTGQGHKGLYEILTTSWHAQLSLNLAMLGSLTIIVAHHMYAMPPYPYLATDYGTQLSLFTHHMWIGGFLIVGAAAHAAIFMVRDYDPTTRYNDLLDRVLRHRDAIISHLNWACIFLGFHSFGLYIHNDT : 1161
Ise : FTGQGHKGLYEILTTSWHAQLSLNLAMLGSLTIIVAHHMYAMPPYPYLATDYGTQLSLFTHHMWIGGFLIVGAAAHAAIFMVRDYDPTTRYNDLLDRVLRHRDAIISHLNWACIFLGFHSFGLYIHNDT : 937
Ili : FTGQGHKGLYEILTTSWHAQLSLNLAMLGSLTIIVAHHMYAMPPYPYLATDYGTQLSLFTHHMWIGGFLIVGAAAHAAIFMVRDYDPTTRYNDLLDRVLRHRDAIISHLNWACIFLGFHSFGLYIHNDT : 981
Isc : FTGQGHKGLYEILTTSWHAQLSLNLAMLGSLTIIVAHHMYAMPPYPYLATDYGTQLSLFTHHMWIGGFLIVGAAAHAAIFMVRDYDPTTRYNDLLDRVLRHRDAIISHLNWACIFLGFHSFGLYIHNDT : 1161
Ici : --------------------------------------------------------------------------------------------------------------------------------- : -
Ipe : FTGQGHKGLYEILTTSWHAQLSLNLAMLGSLTIIVAHHMYAMPPYPYLATDYGTQLSLFTHHMWIGGFLIVGAAAHAAIFMVRDYDPTTRYNDLLDRVLRHRDAIISHLNWACIFLGFHSFGLYIHNDT : 1161
Ath : --------------------------------------------------------------------------------------------------------------------------------- : -

 * 1180 * 1200 * 1220 * 1240 * 1260 * 1280 *
Ikl : MSALGRPQDMFSDTAIQLQPVFAQWIQNTHALAPGATAPGATASTSLTWGGGDLVAVGGKVALLPIPLGTADFLVHHIHAFTIHVTVLILLKGVLFARSSRLIPDKANLGFRFPCDGPGRGGTCQVSAW : 1290
Ise : MSALGRPQDMFSDTAIQLQPVFAQWIQNTHALAPGATAPGATASTSLTWGGGDLVAVGGKVALLPIPLGTADFLVHHIHAFTIHVTVLILLKGVLFARSSRLIPDKANLGFRFPCDGPGRGGTCQVSAW : 1066
Ili : MSALGRPQDMFSDTAIQLQPVFAQWIQNTHALAPGATAPGATASTSLTWGGGDLVAVGGKVALLPIPLGTADFLVHHIHAFTIHVTVLILLKGVLFARSSRLIPDKANLGFRFPCDGPGRGGTCQVSAW : 1110
Isc : MSALGRPQDMFSDTAIQLQPVFAQWIQNTHALAPGATAPGATTSTSLTWGGGDLVAVGGKVALLPIPLGTADFLVHHIHAFTIHVTVLILLKGVLFARSSRLIPDKANLGFRFPCDGPGRGGTCQVSAW : 1290
Ici : --------------------------------------------------------------------------------------------------------------------------------- : -
Ipe : MSALGRPQDMFSDTAIQLQPVFAQWIQNTHALAPGATAPGATASTSLTWGGGDLVAVGGKVALLPIPLGTADFLVHHIHAFTIHVTVLILLKGVLFARSSRLIPDKANLGFRFPCDGPGRGGTCQVSAW : 1290
Ath : --------------------------------------------------------------------------------------------------------------------------------- : -

1300 * 1320 * 1340 * 1360 * 1380 * 1400 * 142
Ikl : DHVFLGLFWMYNSISVVIFHFSWKMQSDVWGNISDQGIVTHITGGNFAQSSITINGWLRDFLWAQASQVIQSYGSSFSAYGLFFLGAHFVWAFSLMFLFSGRGYWQELIESIVWAHNKLKVAPATQPRA : 1419
Ise : DHVFLGLFWMYNSISVVIFHFSWKMQSDVWGNISDQGIVTHITGGNFAQSSITINGWLRDFLWAQASQVIQSYGSSFSAYGLLFLGAHFVWAFSLMFLFSGRGYWQELIESIVWAHNKLKVAPATQPRA : 1195
Ili : DHVFLGLFWMYNSISVVIFHFSWKMQSDVWGNISDQGIVTHITGGNFAQSSITINGWLRDFLWAQASQVIQSYGSSFSAYGLFFLGAHFVWAFSLMFLFSGRGYWQELIESIVWAHNKLKVAPATQPRA : 1239
Isc : DHVFLGLFWMYNSISVVIFHFSWKMQSDVWGNISDQGIVTHITGGNFAQSSITINGWLRDFLWAQASQVIQSYGSSFSAYGLFFLGAHFVWAFSLMFLFSGRGYWQELIESIVWAHNKLKVAPATQPRA : 1419
Ici : --------------------------------------------------------------------------------------------------------------------------------- : -
Ipe : DHVFLGLFWMYNSISVVIFHFSWKMQSDVWGNISDQGIVTHITGGNFAQSSITINGWLRDFLWAQASQVIQSYGSSFSAYGLFFLGAHFVWAFSLMFLFSGRGYWQELIESIVWAHNKLKVAPATQPRA : 1419
Ath : --------------------------------------------------------------------------------------------------------------------------------- : -

0 * 1440
Ikl : LSIVQGRAVGVTHYLLGGIATTWAFFLA : 1447
Ise : LSIVQGRAVGVTHYLLGGIATTWAFFLA : 1223
Ili : LSIVQGRAVGVTHYLLGGIATTWAFFLA : 1267
Isc : LSIVQGRAVGVTHYLLGGIATTWAFFLA : 1447
Ici : ---------------------------- : -
Ipe : LSIVQGRAVGVTHYLLGGIATTWAFFLA : 1447
Ath : ---------------------------- : -

**Figure S30 Multiple sequence alignment of the deduced amino acid sequences of PSAB protein.** The origins of the protein sequences used in this alignment are from: *Arabidopsis thaliana* (Ath), *Iodes klaineana* (Ikl), *Iodes cirrhosa* (Ici), *Iodes seretii* (Ise), *Iodes scandens* (Isc), *Iodes perrieri* (Ipe) and *Iodes liberica* (Ili). The amino acid numbers for each sequence are indicated on the right. In the sequence alignment, identical residues are shown with a black background, and similar residues are shown with a gray background.

* 20 * 40 * 60 * 80 * 100 * 120 *
Ikl : MSHSVKIYDTCIGCTQCVRACPTDVLEMIPWDGCKAKQIASAPRTEDCVGCKRCESACPTDFLSVRVYLWHETTRSMGLAY--------------------------------------------------- : 81
Ipe : MSHSVKIYDTCIGCTQCVRACPTDVLEMIPWDGCKAKQIASAPRTEDCVGCKRCESACPTDFLSVRVYLWHETTRSMGLAYFDKCIACEVCVRVCPIDLPVVDWKFEIDIRKKRLLNYSIDFGICIFCGNCV : 132
Isc : MSHSVKIYDTCIGCTQCVRACPTDVLEMIPWDGCKAKQIASAPRTEDCVGCKRCESACPTDFLSVRVYLWHETTRSMGLAY--------------------------------------------------- : 81
Ise : MSHSVKIYDTCIGCTQCVRACPTDVLEMIPWDGCKAKQIASAPRTEDCVGCKRCESACPTDFLSVRVYLWHETTRSMGLAY--------------------------------------------------- : 81
Ath : MSHSVKIYDTCIGCTQCVRACPTDVLEMIPWDGCKAKQIASAPRTEDCVGCKRCESACPTDFLSVRVYLWHETTRSMGLAY--------------------------------------------------- : 81
Ici : MSHSVKIYDTCIGCTQCVRACPTDVLEMIPWDGCKAKQIASAPRTEDCVGCKRCESACPTDFLSVRVYLWHETTRSMGLAY--------------------------------------------------- : 81
Ili : -------------------SCRDKILEMIPLFQC----------------------------------LVHSTSRGMGL----------------------------------------------------- : 26

 140
Ikl : ---------- : -
Ipe : EYCPTNCLSM : 142
Isc : ---------- : -
Ise : ---------- : -
Ath : ---------- : -
Ici : ---------- : -
Ili : ---------- : -

**Figure S31 Multiple sequence alignment of the deduced amino acid sequences of PSAC protein.** The origins of the protein sequences used in this alignment are from: *Arabidopsis thaliana* (Ath), *Iodes klaineana* (Ikl), *Iodes cirrhosa* (Ici), *Iodes seretii* (Ise), *Iodes scandens* (Isc), *Iodes perrieri* (Ipe) and *Iodes liberica* (Ili). The amino acid numbers for each sequence are indicated on the right. In the sequence alignment, identical residues are shown with a black background, and similar residues are shown with a gray background.

* 20 *
Ikl : MTTLS-FPSIFVPLVGLVFPAIAMASLFLHVQKNKIV : 36
Ipe : MTTLS-FPSIFVPLVGLVFPAIAMASLFLYVQKNKIV : 36
Isc : MTTLS-FPSIFVPLVGLVFPAIAMASLFLHVQKNKIV : 36
Ise : MTTLS-FPSIFVPLVGLVFPAIAMASLFLHVQKNKIV : 36
Ili : MTTLS-FPSIFVPLVGLVFPAIAMASLFLHVQKNKIV : 36
Ici : MTTLS-FPSIFVPLVGLVFPAIAMASLFLHVQKNKIV : 36
Ath : MTTFNNLPSIFVPLVGLVFPAIAMASLFLHIQKNKIF : 37

**Figure S32 Multiple sequence alignment of the deduced amino acid sequences of PSAI protein.** The origins of the protein sequences used in this alignment are from: *Arabidopsis thaliana* (Ath), *Iodes klaineana* (Ikl), *Iodes cirrhosa* (Ici), *Iodes seretii* (Ise), *Iodes scandens* (Isc), *Iodes perrieri* (Ipe) and *Iodes liberica* (Ili). The amino acid numbers for each sequence are indicated on the right. In the sequence alignment, identical residues are shown with a black background, and similar residues are shown with a gray background.

* 20 * 40
Ise : MRDLKTYLSVAPVLSALWFGSLAGLLIEINRFFPDA-------- : 36
Ili : MRDLKTYLSVAPVLSALWFGSLAGLLIEINRFFPDA-------- : 36
Ipe : MRDLKTYLSVAPVLSALWFGSLAGLLIEINRFFPDA-------- : 36
Ikl : MRDLKTYLSVAPVLSALWFGSLAGLLIEINRFFPDA-------- : 36
Ath : MRDLKTYLSVAPVLSTLWFGSLAGLLIEINRLFPDALTFPFFSF : 44
Ici : MRDLKTYLSVAPVLSALWFGSLAGLLIEINRFFPDALTFPFFSF : 44
Isc : ---------IQPILLAQWKGPLVYLSSESN-------------- : 21

**Figure S33 Multiple sequence alignment of the deduced amino acid sequences of PSAJ protein.** The origins of the protein sequences used in this alignment are from: *Arabidopsis thaliana* (Ath), *Iodes klaineana* (Ikl), *Iodes cirrhosa* (Ici), *Iodes seretii* (Ise), *Iodes scandens* (Isc), *Iodes perrieri* (Ipe) and *Iodes liberica* (Ili). The amino acid numbers for each sequence are indicated on the right. In the sequence alignment, identical residues are shown with a black background, and similar residues are shown with a gray background.

* 20 * 40 * 60 * 80 * 100 * 120 *
Isc : MTAILERRESESLWGRFCNWITSTENRLYIGWFGVLMIPTLLTATSVFIIAFIAAPPVDIDGIREPVSGSLLYGNNIISGAIIPTSAAIGLHFYPIWEAASVDEWLYNGGPYELIVLHFLLGVACYMGREWE : 132
Ath : MTAILERRESESLWGRFCNWITSTENRLYIGWFGVLMIPTLLTATSVFIIAFIAAPPVDIDGIREPVSGSLLYGNNIISGAIIPTSAAIGLHFYPIWEAASVDEWLYNGGPYELIVLHFLLGVACYMGREWE : 132
Ici : MTAILERRESESLWGRFCNWITSTENRLYIGWFGVLMIPTLLTATSVFIIAFIAAPPVDIDGIREPVSGSLLYGNNIISGAIIPTSAAIGLHFYPIWEAASVDEWLYNGGPYELIVLHFLLGVACYMGREWE : 132
Ipe : MTAILERRESESLWGRFCNWITSTENRLYIGWFGVLMIPTLLTATSVFIIAFIAAPPVDIDGIREPVSGSLLYGNNIISGAIIPTSAAIGLHFYPIWEAASVDEWLYNGGPYELIVLHFLLGVACYMGREWE : 132
Ise : MTAILERRESESLWGRFCNWITSTENRLYIGWFGVLMIPTLLTATSVFIIAFIAAPPVDIDGIREPVSGSLLYGNNIISGAIIPTSAAIGLHFYPIWEAASVDEWLYNGGPYELIVLHFLLGVACYMGREWE : 132
Ikl : MTAILERRESESLWGRFCNWITSTENRLYIGWFGVLMIPTLLTATSVFIIAFIAAPPVDIDGIREPVSGSLLYGNNIISGAIIPTSAAIGLHFYPIWEAASVDEWLYNGGPYELIVLHFLLGVACYMGREWE : 132
Ili : MTAILERRESESLWGRFCNWITSTENRLYIGWFGVLMIPTLLTATSVFIIAFIAAPPVDIDGIREPVSGSLLYGNNIISGAIIPTSVAI-LHFYPIWEEASVDEWLYNGGPYELIVLHFLLGVACYMGREWE : 131

 140 * 160 * 180 * 200 * 220 * 240 * 260
Isc : LSFRLGMRPWIAVAYSAPVAAATAVFLIYPIGQGSFSDGMPLGISGTFNFMIVFQAEHNILMHPFHMLGVAGVFGGSLFSAMHGSLVTSSLIRETTENESANEGYRFGQEEETYNIVAAHGYFGRLIFQYAS : 264
Ath : LSFRLGMRPWIAVAYSAPVAAATAVFLIYPIGQGSFSDGMPLGISGTFNFMIVFQAEHNILMHPFHMLGVAGVFGGSLFSAMHGSLVTSSLIRETTENESANEGYRFGQEEETYNIVAAHGYFGRLIFQYAS : 264
Ici : LSFRLGMRPWIAVAYSAPVAAATAVFLIYPIGQGSFSDGMPLGISGTFNFMIVFQAEHNILMHPFHMLGVAGVFGGSLFSAMHGSLVTSSLIRETTENESANEGYRFGQEEETYNIVAAHGYFGRLIFQYAS : 264
Ipe : LSFRLGMRPWIAVAYSAPVAAAAAVFLIYPIGQGSFSDGMPLGISGTFNFMIVFQAEHNILMHPFHMLGVAGVFGGSLFSAMHGSLVTSSLIRETTENESANEGYRFGQEEETYNIVAAHGYFGRLIFQYAS : 264
Ise : LSFRLGMRPWIAVAYSAPVAAAAAVFLIYPIGQGSFSDGMPLGISGTFNFMIVFQAEHNILMHPFHMLGVAGVFGGSLFSAMHGSLVTSSLIRETTENESANEGYRFGQEEETYNIVAAHGYFGRLIFQYAS : 264
Ikl : LSFRLGMRPWIAVAYSAPVAAAAAVFLIYPIGQGSFSDGMPLGISGTFNFMIVFQAEHNILMHPFHMLGVAGVFGGSLFSAMHGSLVTSSLIRETTENESANEGYRFGQEEETYNIVAAHGYFGRLIFQYAS : 264
Ili : LSFRLGMRPWIAVAYSAPVAAAAAVFLIYPIGQGSFSDGMPLGISGTFNFMIVFQAEHNILMHPFHMLGVAGVFGGSLFSAMHGSLVTSSLIRETTENESANEGYRFGQEEETYNIVAAHGYFGRLIFQYAS : 263

 * 280 * 300 * 320 * 340 *
Isc : FNNSRSLHFFLAAWPVVGIWFTSLGISTMAFNLNGFNFNQSVVDSQGRVINTWADIINRANLGMEVMHERNAHNFPLDLAAIEAPSTNG : 353
Ath : FNNSRSLHFFLAAWPVVGIWFTALGISTMAFNLNGFNFNQSVVDSQGRVINTWADIINRANLGMEVMHERNAHNFPLDLAAVEAPSTNG : 353
Ici : FNNSRSLHFFLAAWPVVGIWFTALGISTMAFNLNGFNFNQSVVDSQGRVINTWADIINRANLGMEVMHERNAHNFPLDLAAIEAPSTNG : 353
Ipe : FNNSRSLHFFLAAWPVVGIWFTALGISTMAFNLNGFNFNQSVVDSQGRVINTWADIINRANLGMEVMHERNAHNFPLDLAGIEAPSTNG : 353
Ise : FNNSRSLHFFLAAWPVVGIWFTALGISTMAFNLNGFNFNQSVVDSQGRVINTWADIINRANLGMEVMHERNAHNFPLDLAAIEAPSTNG : 353
Ikl : FNNSRSLHFFLAAWPVVGIWFTALGISTMAFNLNGFNFNQSVVDSQGRVINTWADIINRANLGMEVMHERNAHNFPLDLAAIEAPSTNG : 353
Ili : FNNSRSLHFFLAAWPVVGIWFTALGISTMAFNLNGFNFNQSVVDSQGRVINTWADIINRANLGMEVMHERNAHNFPLDLAAIEAPSTNG : 352

**Figure S34 Multiple sequence alignment of the deduced amino acid sequences of PSBA protein.** The origins of the protein sequences used in this alignment are from: *Arabidopsis thaliana* (Ath), *Iodes klaineana* (Ikl), *Iodes cirrhosa* (Ici), *Iodes seretii* (Ise), *Iodes scandens* (Isc), *Iodes perrieri* (Ipe) and *Iodes liberica* (Ili). The amino acid numbers for each sequence are indicated on the right. In the sequence alignment, identical residues are shown with a black background, and similar residues are shown with a gray background.

* 20 * 40 * 60 * 80 * 100 * 120 *
Ikl : MGLPWYRVHIVVLNDPGRLLSVHIMHTALVAGWAGSMALYELAVFDPSDPVLDPMWRQGMFVIPFMTRLGITNSWGGWSITGGTITNPGIWSYEGVAGAHIVFSGLCFLAAIWHWVYWDLEIFCDERTGK : 130
Ipe : MGLPWYRVHIVVLNDPGRLLSVHIMHTALVAGWAGSMALYELAVFDPSDPVLDPMWRQGMFVIPFMTRLGITNSWGGWSITGGTITNPGIWSYEGVAGAHIVFSGLCFLAAIWHWVYWDLEIFCDERTGK : 130
Isc : MGLPWYRVHIVVLNDPGRLLSVHIMHTALVAGWAGSMALYELAVFDPSDPVLDPMWRQGMFVIPFMTRLGITNSWGGWSITGGTITNSGIWSYEGVAGAHIVFSGLCFLAAIWHWVYWDLEIFCDERTGK : 130
Ici : MGLPWYRVHIVVLNDPGRLLSVHIMHTALVAGWAGSMALYELAVFDPSDPVLDPMWRQGMFVIPFMTRLGITNSWGGWSITGGTITNSGIWSYEGVAGAHIVFSGLCFLAAIWHWVYWDLEIFCDERTGK : 130
Ili : -GLPWYRVHIVVLNDPGRLLSVHIMHTALVAGWAGSMALYELAVFDPSDPVLDPMWRQGMFVIPFMTRLGITNSWGGWSITGGTITNPGIWSYEGVAGAHIVFSGLCFLAAIWHWVYWDLEIFCDERTGK : 129
Ise : MGLPWYRVHIVVLNDPGRLLSVHIMHTALVAGWAGSMALYELAVFDPSDPVLDPMWRQGMFVIPFMTRLGITNSWGGWSITGGTITNPGIWSYEGVAGAHIVFSGLCFLAAIWHWVYWDLEIFCDERTGK : 130
Ath : MGLPWYRVHTVVLNDPGRLLAVHIMHTALVAGWAGSMALYELAVFDPSDPVLDPMWRQGMFVIPFMTRLGITNSWGGWNITGGTITNPGLWSYEGVAGAHIVFSGLCFLAAIWHWVYWDLEIFCDERTGK : 130

 140 * 160 * 180 * 200 * 220 * 240 * 260
Ikl : PSLDLPKIFGIHLFLSGVACFGFGAFHVTGLYGPGIWVSDPYGLTGKVQPVNPAWGVEGFDPFVPGGIASHHIAAGTLGILAGLFHLSVRPPQRLYKGLRMGNIETVLSSSIAAVFFAAFVVAGTMWYGS : 260
Ipe : PSLDLPKIFGIHLFLSGVACFGFGAFHVTGLYGPGIWVSDPYGLTGKVQPVNPAWGVEGFDPFVPGGIASHHIAAGTLGILAGLFHLSVRPPQRLYKGLRMGNIETVLSSSIAAVFFAAFVVAGTMWYGS : 260
Isc : PSLDLPKIFGIHLFLSGVACFGFGAFHVTGLYGPGIWVSDPYGLTGKVQPVNPAWGVEGFDPFVPGGIASHHIAAGTLGILAGLFHLSVRPPQRLYKGLRMGNIETVLSSSIAAVFFAAFVVAGTMWYGS : 260
Ici : PSLDLPKIFGIHLFLSGVACFGFGAFHVTGLYGPGIWVSDPYGLTGKVQSVNPAWGVEGFDPFVPGGIASHHIAAGTLGILAGLFHLSVRPPQRLYKGLRMGNIETVLSSSIAAVFFAAFVVAGTMWYGS : 260
Ili : PSLDLPKIFGIHLFLSGVACFGFGAFHVTGLYGPGIWVSDPYGLTGKVQPVNPAWGVEGFDPFVPGGIASHHIAAGTLGILAGLFHLSVRPPQRLYKGLRMGNIETVLSSSIAAVFFAAFVVAGTMWYGS : 259
Ise : PSLDLPKIFGIHLFLSGVACFGFGAFHVTGLYGPGIWVSDPYGLTGKVQPVNPAWGVEGFDPFVPGGIASHHIAAGTLGILAGLFHLSVRPPQRLYKGLRMGNIETVLSSSIAAVFFAAFVVAGTMWYGS : 260
Ath : PSLDLPKIFGIHLFLSGVACFGFGAFHVTGLYGPGIWVSDPYGLTGKVQPVNPAWGVEGFDPFVPGGIASHHIAAGTLGILAGLFHLSVRPPQRLYKGLRMGNIETVLSSSIAAVFFAAFVVAGTMWYGS : 260

 * 280 * 300 * 320 * 340 * 360 * 380 *
Ikl : ATTPIELFGPTRYQWDQGYFQQEIYRRVSAGLAENQSLSEAWSKIPEKLAFYDYIGNNPAKGGLFRAGSMDNGDGIAVGWLGHPIFRDKEGRELFVRRMPTFFETFPVVLVDGDGIVRADVPFRRAESKY : 390
Ipe : ATTPIELFGPTRYQWDQGYFQQEIYRRVSAGLAENQSLSEAWSKIPEKLAFYDYIGNNPAKGGLFRAGSMDNGDGIAVGWLGHPIFRDKEGRELFVRRMPTFFETFPVVLVDGDGIVRADVPFRRAESKY : 390
Isc : ATTPIELFGPTRYQWDQGYFQQEIYRRVSAGLAENQSLSEAWSKIPEKLAFYDYIGNNPAKGGLFRAGSMDNGDGIAVGWLGHPIFRDKEGRELFVRRMPTFFETFPVVLVDGDGIVRADVPFRRAESKY : 390
Ici : ATTPIELFGPTRYQWDQGYFQQEIYRRVSAGLAENQSLSEAWSKIPEKLAFYDYIGNNPAKGGLFRAGSMDNGDGIAVGWLGHPIFRDKEGRELFVRRMPTFFETFPVVLVDGDGIVRADVPFRRAESKY : 390
Ili : ATTPIELFGPTRYQWDQGYFQQEIYRRVSAGLAENQSLSEAWSKIPEKLAFYDYIGNNPAKGGLFRAGSMDNGDGIAVGWLGHPIFRDKEGRELFVRRMPTFFETFPVVLVDGDGIVRADVPFRRAESKY : 389
Ise : ATTPIELFGPTRYQWDQGYFQQEIYRRVSAGLAENQSLSEAWSKIPEKLAFYDYIGNNPAKGGLFRAGSMDNGDGIAVGWLGHPIFRDKEGRELFVRRMPTFFETFPVVLVDGDGIVRADVPFRRAESKY : 390
Ath : ATTPIELFGPTRYQWDQGYFQQEIYRRVSAGLAENQSLSEAWAKIPEKLAFYDYIGNNPAKGGLFRAGSMDNGDGIAVGWLGHPVFRNKEGRELFVRRMPTFFETFPVVLVDGDGIVRADVPFRRAESKY : 390

400 * 420 * 440 * 460 * 480 * 500
Ikl : SVEQVGVTVEFYGGELNGVSYSDPATVKKYARRAQLGEIFELDRATLKSDGVFRSSPRGWFTFGHASFALLFFFGHIWHGARTLFRDVFAGIDPDLDAQVEFGAFQKLGDPTTRRQGV : 508
Ipe : SVEQVGVTVEFYGGELNGVSYSDPATVKKYARRAQLGEIFELDRATLKSDGVFRSSPRGWFTFGHASFALLFFFGHIWHGARTLFRDVFAGIDPDLDAQVEFGAFQKLGDPTTRRQGV : 508
Isc : SVEQVGVTVEFYGGELNGVSYSDPATVKKYARRAQLGEIFELDRATLKSDGVFRSSPRGWFTFGHASFALLFFFGHIWHGARTLFRDVFAGIDPDLDAQVEFGAFQKIGDPTTRRQGV : 508
Ici : SVEQVGVTVEFYGGELNGVSYSDPATVKKYARRAQLGEIFELDRATLKSDGVFRSSPRGWFTFGHASFALLFFFGHIWHGARTLFRDVFAGIDPDLDAQVEFGAFQKLGDPTTRRQGV : 508
Ili : SVEQVGVTVEFYGGELNGVSYSDPATVKKYARRAQLGEIFELDRATLKSDGVFRSSPRGWFTFGHTSFALLFFFGHIWHGARTLFRDVFAGIDPDLDAQVEFGAFQKLGDPTTRRQGV : 507
Ise : SVEQVGVTVEFYGGELNGVSYSDPATVKKYARRAQLGEIFELDRATLKSDGVFRSSPRGWFTFGHTSFALLFFFGHIWHGARTLFRDVFAGIDPDLDAQVEFG--------------- : 493
Ath : SVEQVGVTVEFYGGELNGVSYSDPATVKKYARRAQLGEIFELDRATLKSDGVFRSSPRGWFTFGHASFALLFFFGHIWHGARTLFRDVFAGIDPDLDAQVEFGAFQKLGDPTTKRQAV : 508

**Figure S35 Multiple sequence alignment of the deduced amino acid sequences of PSBB protein.** The origins of the protein sequences used in this alignment are from: *Arabidopsis thaliana* (Ath), *Iodes klaineana* (Ikl), *Iodes cirrhosa* (Ici), *Iodes seretii* (Ise), *Iodes scandens* (Isc), *Iodes perrieri* (Ipe) and *Iodes liberica* (Ili). The amino acid numbers for each sequence are indicated on the right. In the sequence alignment, identical residues are shown with a black background, and similar residues are shown with a gray background.

* 20 * 40 * 60 * 80 * 100 * 120 *
Isc : MKTLYSLRRFYPVETLFNGTLALAGRDQETTGFAWWAGNARLINLSGKLLGAHVAHAGLIVFWAGAMNLFEVAHFVPEKPMYEQGLILLPHLATLGWGVGPGGEVIDTFPYFVSGVLHLISSAVLGFGGI : 130
Ici : MKTLYSLRRFYPVETLFNGTLALAGRDQETTGFAWWAGNARLINLSGKLLGAHVAHAGLIVFWAGAMNLFEVAHFVPEKPMYEQGLILLPHLATLGWGVGPGGEVIDTFPYFVSGVLHLISSAVLGFGGI : 130
Ikl : MKTLYSLRRFYPVETLFNGTLALAGRDQETTGFAWWAGNARLINLSGKLLGAHVAHAGLIVFWAGAMNLFEVAHFVPEKPMYEQGLILLPHLATLGWGVGPGGEVIDTFPYFVSGVLHLISSAVLGFGGI : 130
Ipe : MKTLYSLRRFYPVETLFNGTLALAGRDQETTGFAWWAGNARLINLSGKLLGAHVAHAGLIVFWAGAMNLFEVAHFVPEKPMYEQGLILLPHLATLGWGVGPGGEVIDTFPYFVSGVLHLISSAVLGFGGI : 130
Ise : MKTLYSLRRFYPVETLFNGTLALAGRDQETTGFAWWAGNARLINLSGKLLGAHVAHAGLIVFWAGAMNLFEVAHFVPEKPMYEQGLILLPHLATLGWGVGPGGEVIDTFPYFVSGVLHLISSAVLGFGGI : 130
Ili : MKTLYSLRRFYPVETLFNGTLALAGRDQETTGFAWWAGNARLINLSGKLLGAHVAHAGLIVFWAGAMNLFEVAHFVPEKPMYEQGLILLPHLATLGWGVGPGGEVIDTFPYFVSGVLHLISSAVLGFGGI : 130
Ath : MKTLYSLRRFYHVETLFNGTLALAGRDQETTGFAWWAGNARLINLSGKLLGAHVAHAGLIVFWAGAMNLFEVAHFVPEKPMYEQGLILLPHLATLGWGVGPGGEVIDTFPYFVSGVLHLISSAVLGFGGI : 130

 140 * 160 * 180 * 200 * 220 * 240 * 260
Isc : YHALLGPETLEESFPFFGYVWKDRNKMTTILGIHLILLGLGAFLLVFKALYFGGVYDTWAPGGGDVRKITNLTLSPSIIFGYLLKSPFGGEGWIVSVDDLEDIIGGHVWLGSICILGGIWHILTKPFAWA : 260
Ici : YHALLGPETLEESFPFFGYVWKDRNKMTTILGIHLILLGLGAFLLVFKALYFGGVYDTWAPGGGDVRKITNLTLSPSIIFGYLLKSPFGGEGWIVSVDDLEDIIGGHVWLGSICILGGIWHILTKPFAWA : 260
Ikl : YHALLGPETLEESFPFFGYVWKDRNKMTTILGIHLILLGLGAFLLVFKALYFGGVYDTWAPGGGDVRKITNLTLSPSIIFGYLLKSPFGGEGWIVSVDDLEDIIGGHVWLGSICILGGIWHILTKPFAWA : 260
Ipe : YHALLGPETLEESFPFFGYVWKDRNKMTTILGIHLILLGLGAFLLVFKALYFGGVYDTWAPGGGDVRKITNLTLSPSIIFGYLLKSPFGGEGWIVSVDDLEDIIGGHVWLGSICILGGIWHILTKPFAWA : 260
Ise : YHALLGPETLEESFPFFGYVWKDRNKMTTILGIHLILLGLGAFLLVFKALYFGGVYDTWAPGGGDVRKITNLTLSPSIIFGYLLKSPFGGEGWIVSVDDLEDIIGGHVWLGSICILGGIWHILTKPFAWA : 260
Ili : YHALLGPETLEESFPFFGYVWKDRNKMTTILGIHLILLGLGAFLLVFKALYFGGVYDTWAPGGGDVRKITNLTLSPSIIFGYLLKSPFGGEGWIVSVDDLEDIIGGHVWLGSICILGGIWHILTKPFAWA : 260
Ath : YHALLGPETLEESFPFFGYVWKDRNKMTTILGIHLILLGVGAFLLVFKALYFGGVYDTWAPGGGDVRKITNLTLSPSVIFGYLLKSPFGGEGWIVSVDDLEDIIGGHVWLGSICIFGGIWHILTKPFAWA : 260

 * 280 * 300 * 320 * 340 * 360 * 380 *
Isc : RRALVWSGEAYLSYSLGALAVFGFIACCFVWFNNTAYPSEFYGPTGPEASQAQAFTFLVRDQRLGANIGSAQGPTGLGKYLMRSPTGEVIFGGETMRFWDLRAPWLEPLRGPNGLDLSRLKKDIQPWQER : 390
Ici : RRALVWSGEAYLSYSLGALAVFGFIACCFVWFNNTAYPSEFYGPTGPEASQAQAFTFLVRDQRLGANIGSAQGPTGLGKYLMRSPTGEVIFGGETMRFWDLRAPWLEPLRGPNGLDLSRLKKDIQPWQER : 390
Ikl : RRALVWSGEAYLSYSLGALSVFGFTACCFVWFNNTAYPSEFYGPTGPEASQAQAFTFLVRDQRLGANVGSAQGPTGLGKYLMRSPTGEVIFGGETMRFWDLRAPWLEPLRGPNGLDLSRLKKDIQPWQER : 390
Ipe : RRALVWSGEAYLSYSLGALSIFGFTACCFVWFNNTAYPSEFYGPTGPEASQAQAFTFLVRDQRLGANVGSAQGPTGLGKYLMRSPTGEVIFGGETMRFWDLRAPWLEPLRGPNGLDLSRLKKDIQPWQER : 390
Ise : RRALVWSGEAYLSYSLGALSVFGFTACCFVWFNNTAYPSEFYGPTGPEASQAQAFTFLVRDQRLGANIGSAQGPTGLGKYLMRSPTGEVIFGGETMRFWDLRAPWLEPLRGPNGLDLSRLKKDIQPWQER : 390
Ili : RRALVWSGEAYLSYSLGALSVFGFTACCFVWFNNTAYPSEFYGPTGPEASQAQAFTFLVR-QCLEANIGFTQVPTSLGKYLMRSPTGEVIFGGETMRFWDLRAPWLEPLRGPNGLDLSRLKKDIQPWQER : 389
Ath : RRALVWSGEAYLSYSLAALSVCGFIACCFVWFNNTAYPSEFYGPTGPEASQAQAFTFLVRDQRLGANVGSAQGPTGLGKYLMRSPTGEVIFGGETMRFWDLRAPWLEPLRGPNGLDLSRLKKDIQPWQER : 390

400 * 420 * 440 * 460 *
Isc : RSAEYMTHAPLGSLNSVGGVATEINAVNYVSPRSWLATSHFVLGFFFFVGHLWHAGRARAAAAGFEKGIDRDFEPVLSMTPL- : 472
Ici : RSAEYMTHAPLGSLNSVGGVATEINAVNYVSPRSWLATSHFVLGFFFFVGHLWHAGRARAAAAGFEKGIDRDFEPVLSMTPLN : 473
Ikl : RSAEYMTHAPLGSLNSVGGVATEINAVNYVSPRSWLATSHFVLGFFFFVGHLWHAGRARAAAAGFEKGIDRDFEPVLSMTPLN : 473
Ipe : RSAEYMTHAPLGSLNSVGGVATEINAVNYVSPRSWLATSHFVLGFFFFVGHLWHAGRARAAAAGFEKGIDRDFEPVLSMTPL- : 472
Ise : RSAEYMTHAPLGSLNSVGGVATEINAVNYVSPRSWLATSHFVLGFFFFVGHLWHAGRARAAAAGFEKGIDRDFEPVLSMTPL- : 472
Ili : RSAEYMTHAPLGSLNSVGGVATEINAVNYVSPRSWLATSHFVLGFFFFVGHLWHAGRARAAAAGFEKGIDRDFEPVLSMTPL- : 471
Ath : RSAEYMTHAPLGSLNSVGGVATEINAVNYVSPRSWLSTSHFVLGFFLFVGHLWHAGRARAAAAGFEKGIDRDFEPVLSMTPLN : 473

**Figure S36 Multiple sequence alignment of the deduced amino acid sequences of PSBC protein.** The origins of the protein sequences used in this alignment are from: *Arabidopsis thaliana* (Ath), *Iodes klaineana* (Ikl), *Iodes cirrhosa* (Ici), *Iodes seretii* (Ise), *Iodes scandens* (Isc), *Iodes perrieri* (Ipe) and *Iodes liberica* (Ili). The amino acid numbers for each sequence are indicated on the right. In the sequence alignment, identical residues are shown with a black background, and similar residues are shown with a gray background.

* 20 * 40 * 60 * 80 * 100 * 120 *
Ipe : MTIALGKFTKDENDLFDIMDDWLRRDRFVFVGWSGLLLFPCAYFALGGWFTGTTFVTSWYTHGLASSY-EGCNFLTAAVSTPANSLAHSLLLLWGPEAQGDFTRWCQLGGLWTFVALHGAFGLIGFMLRQFE : 131
Ise : MTIALGKFTKDENDLFDIMDDWLRRDRFVFVGWSGLLLFPCAYFALGGWFTGTTFVTSWYTHGLASSYLEGCNFLTAAVSTPANSLAHSLLLLWGPEAQGDFTRWCQLGGLWTFVALHGAFGLIGFMLRQFE : 132
Ili : MTIALGKFTKDENDLFDIMDDWLRRDRFVFVGWSGLLLFPCAYFALGGWFTGTTFVTSWYTHGLASSYLEGCNFLTAAVSTPANSLAHSLLLLWGPEAQGDFTRWCQLGGLWTFVALHGAFGLIGFMLRQFE : 132
Isc : MTIALGKFTKDENDLFDIMDDWLRRDRFVFVGWSGLLLFPCAYFALGGWFTGTTFVTSWYTHGLASSYLEGCNFLTAAVSTPANSLAHSLLLLWGPEAQGDFTRWCQLGGLWTFVALHGAFGLIGFMLRQFE : 132
Ikl : MTIALGKFTKDENDLFDIMDDWLRRDRFVFVGWSGLLLFPCAYFALGGWFTGTTFVTSWYTHGLASSYLEGCNFLTAAVSTPANSLAHSLLLLWGPEAQGDFTRWCQLGGLWTFVALHGAFGLIGFMLRQFE : 132
Ici : MTIALGKFTKDENDLFDIMDDWLRRDRFVFVGWSGLLLFPCAYFALGGWFTGTTFVTSWYTHGLASSYLEGCNFLTAAVSTPANSLAHSLLLLWGPEAQGDFTRWCQLGGLWTFVALHGAFGLIGFMLRQFE : 132
Ath : MTIALGKFTKDEKDLFDIMDDWLRRDRFVFVGWSGLLLFPCAYFALGGWFTGTTFVTSWYTHGLASSYLEGCNFLTAAVSTPANSLAHSLLLLWGPEAQGDFTRWCQLGGLWAFVALHGAFALIGFMLRQFE : 132

 140 * 160 * 180 * 200 * 220 * 240 * 260
Ipe : LARSVQLRPYNAIAFSAPIAVFVSVFLIYPLGQSGWFFAPSFGVAAIFRFILFFQGFHNWTLNPFHMMGVAGVLGAALLCAIHGATVENTLFEDGDGANTFRAFNPTQAEETYSMVTANRFWSQIFGVAFSN : 263
Ise : LARSVQLRPYNAIAFSAPIAVFVSVFLIYPLGQSGWFFAPSFGVAAIFRFILFFQGFHNWTLNPFHMMGVAGVLGAALLCAIHGATVENTLFEDGDGANTFRAFNPTQAEETYSMVTANRFWSQIFGVAFSN : 264
Ili : LARSVQLRPYNAIAFSAPIAVFVSVFLIYPLGQSGWFFAPSFGVAAIFRFILFFQGFHNWTLNPFHMMGVAGVLGAALLCAIHGATVENTLFEDGDGANTFRAFNPTQAEETYSMVTANRFWSQIFGVAFSN : 264
Isc : LARSVQLRPYNAIAFSAPIAVFVSVFLIYPLGQSGWFFAPSFGVAAIFRFILFFQGFHNWTLNPFHMMGVAGVLGAALLCAIHGATVENTLFEDGDGANTFRAFNPTQAEETYSMVTANRFWSQIFGVAFSN : 264
Ikl : LARSVQLRPYNAIAFSAPIAVFVSVFLIYPLGQSGWFFAPSFGVAAIFRFILFFQGFHNWTLNPFHMMGVAGVLGAALLCAIHGATVENTLFEDGDGANTFRAFNPTQAEETYSMVTANRFWSQIFGVAFSN : 264
Ici : LARSVQLRPYNAIAFSAPIAVFVSVFLIYPLGQSGWFFAPSFGVAAIFRFILFFQGFHNWTLNPFHMMGVAGVLGAALLCAIHGATVENTLFEDGDGANTFRAFNPTQAEETYSMVTANRFWSQIFGVAFSN : 264
Ath : LARSVQLRPYNAIAFSGPIAVFVSVFLIYPLGQSGWFFAPSFGVAAIFRFILFFQGFHNWTLNPFHMMGVAGVLGAALLCAIHGATVENTLFEDGDGANTFRAFNPTQAEETYSMVTANRFWSQIFGVAFSN : 264

 * 280 * 300 * 320 * 340 *
Ipe : KRWLHFFMLFVPVTGLWMSALGVVGLALNLRAYDFVSQEIRAAEDPEFETFYTKNILLNEGIRAWMAAQDQPHENLIFPEEVLPRGNAL : 352
Ise : KRWLHFFMLFVPVTGLWMSALGVVGLALNLRAYDFVSQEIRAAEDPEFETFYTKNILLNEGIRAWMAAQDQPHENLIFPEEVLPRGNAL : 353
Ili : KRWLHFFMLFVPVTGLWMSALGVVGLALNLRAYDFVSQEIRAAEDPEFETFYTKNILLNEGIRAWMAAQDQPHENLIFPEEVLPRGNAL : 353
Isc : KRWLHFFMLFVPVTGLWMSALGVVGLALNLRAYDFVSQEIRAAEDPEFETFYTKNILLNEGIRAWMAAQDQPHENLIFPEEVLPRGNAL : 353
Ikl : KRWLHFFMLFVPVTGLWMSALGVVGLALNLRAYDFVSQEIRAAEDPEFETFYTKNILLNEGIRAWMAAQDQPHENLIFPEEVLPRGNAL : 353
Ici : KRWLHFFMLFVPVTGLWMSSLGVVGLALNLRAYDFVSQEIRAAEDPEFETFYTKNILLNEGIRAWMAAQDQPHENLIFPEEVLPRGNAL : 353
Ath : KRWLHFFMLFVPVTGLWMSALGVVGLALNLRAYDFVSQEIRAAEDPEFETFYTKNILLNEGIRAWMAAQDQPHENLIFPEEVLPRGNAL : 353

**Figure S37 Multiple sequence alignment of the deduced amino acid sequences of PSBD protein.** The origins of the protein sequences used in this alignment are from: *Arabidopsis thaliana* (Ath), *Iodes klaineana* (Ikl), *Iodes cirrhosa* (Ici), *Iodes seretii* (Ise), *Iodes scandens* (Isc), *Iodes perrieri* (Ipe) and *Iodes liberica* (Ili). The amino acid numbers for each sequence are indicated on the right. In the sequence alignment, identical residues are shown with a black background, and similar residues are shown with a gray background.

* 20 * 40 * 60 * 80
Ikl : MSGSTGERSFADIITSIRYWVIHSITIPSLFIAGWLFVSTGLAYDVFGSPRPNEYFTESRQGIPLITGRFDPLEQLDEFSRSF : 83
Ipe : MSGSTGERSFADIITSIRYWVIHSITIPSLFIAGWLFVSTGLAYDVFGSPRPNEYFTESRQGIPLITGRFDPLEQLDEFSRSF : 83
Isc : MSGSTGERSFADIITSIRYWVIHSITIPSLFIAGWLFVSTGLAYDVFGSPRPNEYFTESRQGIPLITGRFDPLEQLDEFSRSF : 83
Ise : MSGSTGERSFADIITSIRYWVIHSITIPSLFIAGWLFVSTGLAYDVFGSPRPNEYFTESRQGIPLITGRFDPLEQLDEFSRSF : 83
Ili : MSGSTGERSFADIITSIRYWVIHSITIPSLFIAGWLFVSTGLAYDVFGSPRPNEYFTESRQGIPLITGRFDPLEQLDEFSRSF : 83
Ici : MSGSTGERSFADIITSIRYWVIHSITIPSLFIAGWLFVSTGLAYDVFGSPRPNEYFTESRQGIPLITGRFDPLEQLDEFSRSF : 83
Ath : MSGSTGERSFADIITSIRYWVIHSITIPSLFIAGWLFVSTGLAYDVFGSPRPNEYFTESRQGIPLITGRFDPLEQLDEFSRSF : 83

**Figure S38 Multiple sequence alignment of the deduced amino acid sequences of PSBE protein.** The origins of the protein sequences used in this alignment are from: *Arabidopsis thaliana* (Ath), *Iodes klaineana* (Ikl), *Iodes cirrhosa* (Ici), *Iodes seretii* (Ise), *Iodes scandens* (Isc), *Iodes perrieri* (Ipe) and *Iodes liberica* (Ili). The amino acid numbers for each sequence are indicated on the right. In the sequence alignment, identical residues are shown with a black background, and similar residues are shown with a gray background.

* 20 *
Ikl : MTIDRTYPIFTVRWLAVHGLAVPTVSFLGSISAMQFIQR : 39
Ipe : MTIDRTYPIFTVRWLAVHGLAVPTVSFLGSISAMQFIQR : 39
Isc : MTIDRTYPIFTVRWLAVHGLAVPTVSFLGSISAMQFIQR : 39
Ise : MTIDRTYPIFTVRWLAVHGLAVPTVSFLGSISAMQFIQR : 39
Ili : MTIDRTYPIFTVRWLAVHGLAVPTVSFLGSISAMQFIQR : 39
Ici : MTIDRTYPIFTVRWLAVHGLAVPTVSFLGSISAMQFIQR : 39
Ath : MTIDRTYPIFTVRWLAVHGLAVPTVSFLGSISAMQFIQR : 39

**Figure S39 Multiple sequence alignment of the deduced amino acid sequences of PSBF protein.** The origins of the protein sequences used in this alignment are from: *Arabidopsis thaliana* (Ath), *Iodes klaineana* (Ikl), *Iodes cirrhosa* (Ici), *Iodes seretii* (Ise), *Iodes scandens* (Isc), *Iodes perrieri* (Ipe) and *Iodes liberica* (Ili). The amino acid numbers for each sequence are indicated on the right. In the sequence alignment, identical residues are shown with a black background, and similar residues are shown with a gray background.

* 20 * 40 * 60 *
Ikl : MNTIGFMATQTVENSSRSGPRRTTVGSLLKPLNSEYGKVAPGWGTTPLMGVAMSLFAIFLSIILEIFNSSVLLDGISMN : 79
Ici : MNTIGFMATQTVENSSRSGPRRTTVGSLLKPLNSEYGKVAPGWGTTPLMGVAMSLFAIFLSIILEIYNSSVLLDGISMN : 79
Ili : MNTIGFMATQTVENSSRSGPRRTTVGSLLKPLNSEYGKVAPGWGTTPLMGVAMSLFAIFLSIILEIYNSSVLLDGISMN : 79
Isc : MNTIGFMATQTVENSSRSGPRRTTVGSLLKPLNSEYGKVAPGWGTTPLMGVAMSLFAIFLSIILEIYNSSVLLDGISMN : 79
Ise : MNTIGFMATQTVENSSRSGPRRTTVGSLLKPLNSEYGKVAPGWGTTPLMGVAMSLFAIFLSIILEIYNSSVLLDGISMN : 79
Ipe : MNTIGFMATQTVENSSRSGPRRTTVGSLLKPLNSEYGKVAPGWGTTPLMGVAMSLFAIFLSIILEIYNSSVLLDGISMN : 79
Ath : ------MATQTVEDSSRSGPRSTTVGKLLKPLNSEYGKVAPGWGTTPLMGVAMALFAVFLSIILEIYNSSVLLDGISVN : 73

**Figure S40 Multiple sequence alignment of the deduced amino acid sequences of PSBH protein.** The origins of the protein sequences used in this alignment are from: *Arabidopsis thaliana* (Ath), *Iodes klaineana* (Ikl), *Iodes cirrhosa* (Ici), *Iodes seretii* (Ise), *Iodes scandens* (Isc), *Iodes perrieri* (Ipe) and *Iodes liberica* (Ili). The amino acid numbers for each sequence are indicated on the right. In the sequence alignment, identical residues are shown with a black background, and similar residues are shown with a gray background.

* 20 * 40 *
Ise : ------FFFKKP----SYLGDCVMLTLKLFVYTVVIFFVSLFIFGFLSNDPGRNPGREE : 49
Ath : -----------------------MLTLKLFVYTVVIFFVSLFIFGFLSNDPGRNPGREE : 36
Ipe : ---IYSLFFSK-----NHLGDCVMLTLKLFVYTVVIFFVSLFIFGFLSNDPGRNPGREE : 51
Ikl : ------FFFSK-----NHLGDCVMLTLKLFVYTVVIFFVSLFIFGFLSNDPGRNPGREE : 48
Ici : MEYLFSFFFSK-----NHLGDCVMLTLKLFVYTVVIFFVSLFIFGFLSNDPGRNPGREE : 54
Isc : MEYLFSFFFQKFFFSKNHLGDCVMLTLKL------------------------------ : 29
Ili : -EFLYSFLSSP------DYDDC------------------------------------- : 15

**Figure S41 Multiple sequence alignment of the deduced amino acid sequences of PSBI protein.** The origins of the protein sequences used in this alignment are from: *Arabidopsis thaliana* (Ath), *Iodes klaineana* (Ikl), *Iodes cirrhosa* (Ici), *Iodes seretii* (Ise), *Iodes scandens* (Isc), *Iodes perrieri* (Ipe) and *Iodes liberica* (Ili). The amino acid numbers for each sequence are indicated on the right. In the sequence alignment, identical residues are shown with a black background, and similar residues are shown with a gray background.

* 20 * 40 * 60
Ise : MSVMLNIVNLICICLNSALYSSSFFLAKLPEAYAFLNPIVDLMPVIPVLFFLLAFVWQAAVSFR : 64
Ili : MSVMLNIVNLICICLNSALYSSSFFLAKLPEAYAFLNPIVDLMPVIPVLFFLLAFVWQAAVSFR : 64
Ipe : MSVMLNIVNLICICLNSALYSSSFFLAKLPEAYAFLNPIVDLMPVIPVLFFLLAFVWQAAVSFR : 64
Isc : -----NIVNLICICLNSALYSSSFFFAKLPEAYAFLNPIVDLMPVIPVLFFLLAFVWQAAVSFR : 59
Ici : MSVMLNIVNLICICLNSALYSSSFFFAKLPEAYAFLNPIVDLMPVIPVLFFLLAFVWQAAVSFR : 64
Ikl : MSVMLNIVNLICICLNSALYSSSFFLAKLPEAYAFLNPIVDLMPVIPVFFFLLAFVWQAAVSFR : 64
Ath : ---MLNIFNLICIFFNSTLFSSTFLVAKLPEAYAFLNPIVDVMPVIPLFFLLLAFVWQAAVSFR : 61

**Figure S42 Multiple sequence alignment of the deduced amino acid sequences of PSBK protein.** The origins of the protein sequences used in this alignment are from: *Arabidopsis thaliana* (Ath), *Iodes klaineana* (Ikl), *Iodes cirrhosa* (Ici), *Iodes seretii* (Ise), *Iodes scandens* (Isc), *Iodes perrieri* (Ipe) and *Iodes liberica* (Ili). The amino acid numbers for each sequence are indicated on the right. In the sequence alignment, identical residues are shown with a black background, and similar residues are shown with a gray background.

* 20 *
Ikl : TTQSNPNEQNVELNRTSLYWGLLLIFVLAVLFSNYFFN : 38
Isc : TTQSNPNEQNVELNRTSLYWGLLLIFVLAVLFSNY--- : 35
Ici : TTQSNPNEQNVELNRTSLYWGLLLIFVLAVLFSNYFFN : 38
Ipe : TTQSNPNEQNVELNRTSLYWGLLLIFVLAVLFSNYFFN : 38
Ise : TTQSNPNEQNVELNRTSLYWGLLLIFVLAVLFSNYFFN : 38
Ili : TTQSNPNEQNVELNRTSLYWGLLLIFVLAVLFSNYFFN : 38
Ath : MTQSNPNEQSVELNRTSLYWGLLLIFVLAVLFSNYFFN : 38

**Figure S43 Multiple sequence alignment of the deduced amino acid sequences of PSBL protein.** The origins of the protein sequences used in this alignment are from: *Arabidopsis thaliana* (Ath), *Iodes klaineana* (Ikl), *Iodes cirrhosa* (Ici), *Iodes seretii* (Ise), *Iodes scandens* (Isc), *Iodes perrieri* (Ipe) and *Iodes liberica* (Ili). The amino acid numbers for each sequence are indicated on the right. In the sequence alignment, identical residues are shown with a black background, and similar residues are shown with a gray background.

* 20 *
Ikl : MEVNILAFIATALFILVPTAFLLIIYVKTVSKND : 34
Ipe : MEVNILAFIATALFILVPTAFLLIIYVKTVSKND : 34
Isc : MEVNILAFIATALFILVPTAFLLIIYVKTVSKND : 34
Ise : MEVNILAFIATALFILVPTAFLLIIYVKTVSKND : 34
Ili : MEVNILAFIATALFILVPTAFLLIIYVKTVSKND : 34
Ici : MEVNILAFIATALFILVPTAFLLIIYVKTVSKND : 34
Ath : MEVNILAFIATALFILVPTAFLLIIYVKTVSQND : 34

**Figure S44 Multiple sequence alignment of the deduced amino acid sequences of PSBM protein.** The origins of the protein sequences used in this alignment are from: *Arabidopsis thaliana* (Ath), *Iodes klaineana* (Ikl), *Iodes cirrhosa* (Ici), *Iodes seretii* (Ise), *Iodes scandens* (Isc), *Iodes perrieri* (Ipe) and *Iodes liberica* (Ili). The amino acid numbers for each sequence are indicated on the right. In the sequence alignment, identical residues are shown with a black background, and similar residues are shown with a gray background.

* 20 * 40 * 60 *
Ikl : METASLVAIFISGLLVSFTGYALYTAFGQPSQQLRDPFEEHGD---------------------------- : 43
Ipe : METASLVAIFISGLLVSFTGYALYTAFGQPSQQLRDPFEEHGDSMIGSSLYLTFGRPPFETQLPPFREYGD : 71
Isc : METASLVAIFISGLLVSFTGYALYTAFGQPSQQLRDPFEEHGD---------------------------- : 43
Ise : METASLVAIFISGLLVSFTGYALYTAFGQPSQQLRDPFEEHGD---------------------------- : 43
Ili : METASLVAIFISGLLVSFTGYALYTAFGQPSQQLRDPFEEHGD---------------------------- : 43
Ici : METASLVAIFISGLLVSFTGYALYTAFGQPSQQLRDPFEEHGD---------------------------- : 43
Ath : METATLVAIFISGLLVSFTGYALYTAFGQPSQQLRDPFEEHGD---------------------------- : 43

**Figure S45 Multiple sequence alignment of the deduced amino acid sequences of PSBN protein.** The origins of the protein sequences used in this alignment are from: *Arabidopsis thaliana* (Ath), *Iodes klaineana* (Ikl), *Iodes cirrhosa* (Ici), *Iodes seretii* (Ise), *Iodes scandens* (Isc), *Iodes perrieri* (Ipe) and *Iodes liberica* (Ili). The amino acid numbers for each sequence are indicated on the right. In the sequence alignment, identical residues are shown with a black background, and similar residues are shown with a gray background.

* 20 *
Isc : MEALVYTFLLVSTLGIIFFAIFFREPPKVPT---- : 31
Ise : MEALVYTFLLVSTLGIIFFAIFFREPPKVPT---- : 31
Ili : MEALVYTFLLVSTLGIIFFAIFFREPPKVPT---- : 31
Ipe : MEALVYTFLLVSTLGIIFFAIFFREPPKVPT---- : 31
Ici : MEALVYTFLLVSTLGIIFFAIFFREPPKVPTKKMK : 35
Ikl : MEALVYTFLLVSTLGIIFFAIFFREPPKVPTKKMK : 35
Ath : MEALVYTFLLVSTLGIIFFAIFFREPPKISTKK-- : 33

**Figure S46 Multiple sequence alignment of the deduced amino acid sequences of PSBT protein.** The origins of the protein sequences used in this alignment are from: *Arabidopsis thaliana* (Ath), *Iodes klaineana* (Ikl), *Iodes cirrhosa* (Ici), *Iodes seretii* (Ise), *Iodes scandens* (Isc), *Iodes perrieri* (Ipe) and *Iodes liberica* (Ili). The amino acid numbers for each sequence are indicated on the right. In the sequence alignment, identical residues are shown with a black background, and similar residues are shown with a gray background.

* 20 * 40 * 60
Isc : MTLAFQLAVFALIATSSILLISVPVVFASPEGWSSNKNVLFSGTSLWIGLVFLVGILNSLIS : 62
Ici : MTLAFQLAVFALIATSSILLISVPVVFASPEGWSSNKNVLFSGTSLWIGLVFLVGILNSLIS : 62
Ikl : MTLAFQLAVFALIATSSILLISVPVVFASPDGWSSNKNVLFSGTSLWIGLVFLVGILNSLIS : 62
Ipe : MTLAFQLAVFALIATSSILLISVPVVFASPDGWSSNKNVLFSGTSLWIGLVFLVGILNSLIS : 62
Ise : MTLAFQLAVFALIATSSILLISVPVVFASPDGWSSNKNVLFSGTSLWVGLVFLVGILNSLIS : 62
Ili : MTLAFQLAVFALIATSSILLISVPVVFASPDGWSSNKNVLFSGTSLWIGLVFLVGILNSLIS : 62
Ath : MTIAFQLAVFALIITSSILLISVPVVFASPDGWSSNKNVVFSGTSLWIGLVFLVGILNSLIS : 62

**Figure S47 Multiple sequence alignment of the deduced amino acid sequences of PSBZ protein.** The origins of the protein sequences used in this alignment are from: *Arabidopsis thaliana* (Ath), *Iodes klaineana* (Ikl), *Iodes cirrhosa* (Ici), *Iodes seretii* (Ise), *Iodes scandens* (Isc), *Iodes perrieri* (Ipe) and *Iodes liberica* (Ili). The amino acid numbers for each sequence are indicated on the right. In the sequence alignment, identical residues are shown with a black background, and similar residues are shown with a gray background.

* 20 * 40 * 60 * 80 * 100 * 120 *
Ikl : MSPQTETKASVGFKAGVKDYKLTYYTPDYKTKDTDILAAFRVTPQPGVPPEEAGAAVAAESSTGTWTTVWTDGLTSLDRYKGRCYGIEPVAGEENQFIAYVAYPLDLFEEGSVTNMFTSIVGNVFGFKAL : 130
Ili : MSPQTETKASVGFKAGVKDYKLNYYTPDYQTKDTDILAAFRVTPQPGVPPEEAGAAVAAESSTGTWTTVWTDGLTSLDRYKGRCYGIEPVAGEENQFIAYVAYPLDLFEEGSVTNMFTSIVGNVFGFKAL : 130
Isc : MSPQTETKASVGFKAGVKDYKLTYYTPEYQTKATDILAAFRVTPQPGVPPEEAGAAVAAESSTGTWTTVWTDGLTSLDRYKGRCYGIEPVAGEENQYIAYVAYPLDLFEEGSVTNMFTSIVGNVFGFKAL : 130
Ici : MSPQTETKASVGFKAGVKDYKLTYYTPDYQTKDTDILAAFRVTPQPGVPPEEAGAAVAAESSTGTWTTVWTDGLTSLDRYKGRCYEIEPVAGEENQYIAYVAYPLDLFEEGSVTNMLTSIVGNVFGFKAL : 130
Ise : MSPQTETKASVGFKAGVKDYKLTYYTPDYQTKDTDILAAFRVTPQPGVPPEEAGAAVAAESSTGTWTAVWTDGLTSLDRYKGRCYGIEPVAGEENQYIAYVAYPLDLFEEGSVTNMFTSIVGNVFGFKAL : 130
Ipe : MSPQTETKASVGFKAGVKDYKLTYYTPEYQTKDTDILAAFRVTPQPGVPPEEAGAAVAAESSTGTWTTVWTDGLTSLDRYKGRCYGIEPVAGEENQYIAYVAYPLDLFEEGSVTNMFTSIVGNVFGFKAL : 130
Ath : MSPQTETKASVGFKAGVKEYKLTYYTPEYETKDTDILAAFRVTPQPGVPPEEAGAAVAAESSTGTWTTVWTDGLTSLDRYKGRCYHIEPVPGEETQFIAYVAYPLDLFEEGSVTNMFTSIVGNVFGFKAL : 130

 140 * 160 * 180 * 200 * 220 * 240 * 260
Ikl : RALRLEDLRIPPAYTKTFQGPPHGIQVERDKLNKYGRPLLGCTIKPKLGLSAKNYGRAVYECLRGGLDFTKDDENVNSQPFMRWRDRFLFCAEALFKAQAETGEIKGHYLNATAGTCEEMMKRAVFAREL : 260
Ili : RALRLEDLRIPPAYTKTFQGPPHGIQVERDKLNKYGRPLLGCTIKPKLGLSAKNYGRAVYECLRGGLDFTKDDENVNSQPFMRWRDRFLFCAEALYKAQAETGEIKGHYLNATAGTCEEMMKRAVFAREL : 260
Isc : RALRLEDLRIPPAYSKTFQGPPHGIQVERDKLNKYGRPLLGCTIKPKLGLSAKNYGRAVYECLRGGLDFTKDDENVNSQPFMRWRDRFVFCAEAIYKAQAETGEIKGHYLNATAGTCEEMMKRAIFAREL : 260
Ici : RALRLEDLRIPPAYSKTFQGPPHGIQVERDKLNKYGRPLLGCTIKPKLGLSAKNYGRAVYECLRGGLDFTKDDENVNSQPFMRWRDRFVFCAEALYKAQAETGEIKGHYLNATAGTCEEMMKRATFAREL : 260
Ise : RALRLEDLRIPPAYTKTFQGPPHGIQVERDKLNKYGRPLLGCTIKPKLGLSAKNYGRAVYECLRGGLDFTKDDENVNSQPFMRWRDRFLFCTEALYKAQTETGEVKGHYLNATAGTCEEMMKRAIFAREL : 260
Ipe : RALRLEDLRIPPAYTKTFQGPPHGIQVERDKLNKYGRPLLGCTIKPKLGLSAKNYGRAVYECLRGGLDFTKDDENVNSQPFMRWRDRFLFCAEALYKAQAETGEIKGHYLNATAGTCEEMLKRAVFAREL : 260
Ath : AALRLEDLRIPPAYTKTFQGPPHGIQVERDKLNKYGRPLLGCTIKPKLGLSAKNYGRAVYECLRGGLDFTKDDENVNSQPFMRWRDRFLFCAEAIYKSQAETGEIKGHYLNATAGTCEEMIKRAVFAREL : 260

 * 280 * 300 * 320 * 340 * 360 * 380 *
Ikl : GVPIVMHDYLTGGFTANTSLAHYCRDNGLLLHIHRAMHAVIDRQKNHGMHFRVLAKALRLSGGDHIHGGTVVGKLEGEREITLGFVDLLRDDYVEKDRSRGIYFTQDWVSLPGVIPVASGGIHVWHMPAL : 390
Ili : GVPIVMHDYLTGGFTANTSLAHYCRDNGLLLHIHRAMHAVIDRQKNHGMHFRVLAKALRLSGGDHVHGGTVVGKLEGEREITLGFVDLLRDDYVEKDRSRGIYFTQDWVSLPGVIPVASGGIHVWHMPAL : 390
Isc : GVPIVMHDYLTGGFTANTSLAHYCRDNGLLLHIHRAMHAVIDRQKNHGIHFRVLAKALRMSGGDHIHGGTVVGKLEGEREITLGFVDLLRDDYIEKDRSRGIYFTQDWVSLPGVIPVASGGIHVWHMPAL : 390
Ici : GVPIIMHDYLTGGFTANTSLAHYCRDNGLLLHIHRAMHAVIDRQKNHGIHFRVLAKALRMSGGDHIHGGTVVGKLEGEREITLGFVDLLRDDYIEKDRSRGIYFTQDWVSLPGVIPVASGGIHVWHMPAL : 390
Ise : GVPIIMHDYLTGGFTANTSLAHYCRDNGLLLHIHRAMHAVIDRQKNHGMHFRVLAKGLRLSGGDHIHGGTVVGKLEGEREITLGFVDLLRDDYIEKDRSRGIYFTQDWVSLPGVIPVASGGIHVWHMPAL : 390
Ipe : GVPIVMHDYLTGGFTANTSLAHYCRDNGLLLHIHRAMHAVIDRQKNHGMHFRVLAKALRLSGGDHIHGGTVVGKLEGEREITLGFVDLLRDDYVEKDRSRGIYFTQDWVSLPGVIPVASGGIHVWHMPAL : 390
Ath : GVPIVMHDYLTGGFTANTSLSHYCRDNGLLLHIHRAMHAVIDRQKNHGMHFRVLAKALRLSGGDHIHAGTVVGKLEGDRESTLGFVDLLRDDYVEKDRSRGIFFTQDWVSLPGVLPVASGGIHVWHMPAL : 390

400 * 420 * 440 * 460 *
Ikl : TEIFGDDSVLQFGGGTLGHPWGNAPGAVANRVALEACVQARNEGRDLAREGNEIIREASKWSPELAAACEVWKEIKFEFPAMDTI---- : 475
Ili : TEIFGDDSVLQFGGGTLGHPWGNAPGAVANRVALEACVQARNEGRDLAREGNEIIREASKWSPELAAACEVWKEIKFEFPAMDTI---- : 475
Isc : TEIFGDDSVLQFGGGTLGHPWGNAPGAVANRVALEACVQARNEGRDLAREGNEIIREASKWSPELAAACEVWKEIKFEFPAMDTL---- : 475
Ici : TEIFGDDSVLQFGGGTLGHPWGNAPGAVANRVALEACVQARNEGRDLAREGNEIIREASKWSPELAAACEVWKEIKFEFPAMDTL---- : 475
Ise : TEIFGDDSVLQFGGGTLGHPWGNAPGAVANRVALEACVQARNEGRDLAREGNEIIREASKWSPELAAACEVWKEIKFEFPAMDTI---- : 475
Ipe : TEIFGDDSVLQFGGGTLGHPWGNAPGAVANRVALEACVQARNEGRDLAREGNEIIREASKWSPELAAACEVWKEIKFEFPAMDT----- : 474
Ath : TEIFGDDSVLQFGGGTLGHPWGNAPGAVANRVALEACVQARNEGRDLAVEGNEIIREACKWSPELAAACEVWKEITFNFPTIDKLDGQE : 479

**Figure S48 Multiple sequence alignment of the deduced amino acid sequences of RBCL protein.** The origins of the protein sequences used in this alignment are from: *Arabidopsis thaliana* (Ath), *Iodes klaineana* (Ikl), *Iodes cirrhosa* (Ici), *Iodes seretii* (Ise), *Iodes scandens* (Isc), *Iodes perrieri* (Ipe) and *Iodes liberica* (Ili). The amino acid numbers for each sequence are indicated on the right. In the sequence alignment, identical residues are shown with a black background, and similar residues are shown with a gray background.

* 20 * 40 * 60 * 80 * 100 * 120 *
Isc : ---------------------------------------------------------------------------------------------------------------------------------- : -
Ili : MAIHLYKTSTPSTRNGTVDSQVKSNPRNNLIYGQHRCGKGRNARGIITAGHRGGGHKRLYRKIYFRRNEKDIYGRIVTIEYYPNRNAYICLIHYGDGEKRYILHPRGAIIGDTIVSGTEVPIKMGNALPL : 130
Ise : MAIHLYKTSTPSTRNGTVDSQVKSNPRNNLIYGQHPCGKGRNARGIITAGHRGGGHKRLYRKIDFRRNEKDIYGRIVTIEYDPNRNAYICLIHYGDGEKRYILHPRGAIIGDTIVSGTEVPIKMGNALPL : 130
Ici : MAIHLYKTSTPSTRNGTVDSQVKSNPRNNLIYGQHRCGKGRNARGIITAGHRGGGHKRLYRKIDFRRNEKDIYGRIVTIEYDPNRNAYICLIHYGDGEKRYILHPRGAIIGDTIVSGTEVPIKMGNALPL : 130
Ikl : MAIHLYKTSTPSTRNGTVDSQVKSNPRNNLIYGQHRCGKGRNARGIITAGHRGGGHKRLYRKIDFRRNEKDIYGRIVTIEYDPNRNAYICLIHYGDGEKRYILHPRGAIIGDTIVSGTEVPIKMGNALPL : 130
Ipe : MAIHLYKTSTPSTRNGTVDSQVKSNPRNNLIYGQHRCGKGRNARGIITAGHRGGGHKRLYRKIDFRRNEKDIYGRIVTIEYDPNRNAYICLIHYGDGEKRYILHPRGAIIGDTIVSGTEVPIKMGNALPL : 130
Ath : MAIHLYKTSTPSTRNGAVDSQVKSNPRNNLICGQHHCGKGRNARGIITARHRGGGHKRLYRKIDFRRNAKDIYGRIVTIEYDPNRNAYICLIHYGDGEKRYILHPRGAIIGDTIVSGTEVPIKMGNALPL : 130

 140 * 160 * 180 * 200 * 220 * 240 * 260
Isc : ---------------------------------------------------------------------------------------------------------------------------------- : -
Ili : SA-------------------------------------------------------------------------------------------------------------------------------- : 132
Ise : SA-------------------------------------------------------------------------------------------------------------------------------- : 132
Ici : ---------------------------------------------------------------------------------------------------------------------------------- : -
Ikl : SA-------------------------------------------------------------------------------------------------------------------------------- : 132
Ipe : SADISSQIGSCMPLSMMRIGTFVHNIELRPGQGGKLVWAAGTSAKILSEPSMSSSRYCEIKLPSGVKKLIDTKCRATVGMVSNPDHKNKKLRKAGQSRWLGRRPKVRGVAMNPVDHPHGGGEGKSKSSGS : 260
Ath : ---------------------------------------------------------------------------------------------------------------------------------- : -

 * 280 * 300 * 320 * 340 * 360 * 380 *
Isc : ------------TSTDMPLGTAIHNIEITLGKGGQLARAAGAVAKLIAKE---------GKSATLKLPSGEVRLISKNCSATVGQVGNVGVNQKSLGRAGSKRWLGKRPVVRGVVMNPVDHPHGGGEGRA : 109
Ili : ------------TSTDMPLGTAIHNIEITLGKGGQLARAAGAVAKLIAKE---------GKSATLKLPSGEVRLISKNCSATVGQVGNVGVNQKSLGRAGSKRWLGKRPVVRGVVMNPVDHPHGGGEGRA : 241
Ise : ------------TSTDMPLGTAIHNIEITLGKGGQLARAAGAVAKLIAKE---------GKSATLKLPSGEVRLISKNCSATVGQVGNVGVNQKSLGRAGSKRWLGKRPVVRGVVMNPVDHPHGGGEGRA : 241
Ici : ------------TSTDMPLGTAIHNIEITLGKGGQLARAAGAVAKLIAKE---------GKSATLKLPSGEVRLISKNCSATVGQVGNVGVNQKSLGRAGSKRWLGKRPVVRGVVMNPVDHPHGGGEGRA : 239
Ikl : ------------TSTDMPLGTAIHNIEITLGKGGQLARAAGAVAKLIAKE---------GKSATLKLPSGEVRLISKNCSATVGQVGNVGVNQKSLGRAGSKRWLGKRPVVRGVVMNPVDHPHGGGEGRA : 241
Ipe : HGRCSVTPWGKPTSTDMPLGTAIHNIEITLGKGGQLARAAGAVATLIAKKGTEHIKIKRGKLNTLKLPSGEVRLISKNCSATVGQVGNVGVNQKSLGRAGSKRWLGKRPVVRGVVMNPVDHPHGGGEGRA : 390
Ath : --------------TDMPLGTAIHNIEITLGRGGQLARAAGAVAKLIAKE---------GKSATLKLPSGEVRLISKNCSATVGQVGNVGVNQKSLGRAGSKCWLGKRPVVRGVVMNPVDHPHGGGEGRA : 237

400 * 420
Isc : PIGRKKPTTPWGYPALGRRSRKRNKYSDNLILRRRSK : 146
Ili : PIGRKKPTTPWGYPALGRRSRKRNKYSDNLILRRRSK : 278
Ise : PIGRKKPTTPWGYPALGRRSRKRNKYSDNLILRRRSK : 278
Ici : PIGRKKPTTPWGYPALGRRSRKRNKYSDNLILRRRSK : 276
Ikl : PIGRKKPTTPWGYPALGRRSRKRNKYSDNLILRRRSK : 278
Ipe : PIGRKKPTTPWGYPALGRRSRKRNKYSDNLILRRRSK : 427
Ath : PIGRKKPVTPWGYPALGRRTRKRKKYSETLILRRRSK : 274

**Figure S49 Multiple sequence alignment of the deduced amino acid sequences of RPL2 protein.** The origins of the protein sequences used in this alignment are from: *Arabidopsis thaliana* (Ath), *Iodes klaineana* (Ikl), *Iodes cirrhosa* (Ici), *Iodes seretii* (Ise), *Iodes scandens* (Isc), *Iodes perrieri* (Ipe) and *Iodes liberica* (Ili). The amino acid numbers for each sequence are indicated on the right. In the sequence alignment, identical residues are shown with a black background, and similar residues are shown with a gray background.

* 20 * 40 * 60 * 80 * 100 * 120
Ipe : MIQPQTHLNVADNSGARELMCIRIIGASNRRYAHIGDVIVAVIKEALPNMPLERSEIVRAVIVRTCKELKRDNGMIIRYDDNAAVVIDQEGNPKGTRVFGAIARELRQLNFTKIVSLAPEVL : 122
Ici : MIQPQTHLNVADNSGARELMCIRIIGASNRRYAHIGDVIVAVIKEALPNMPLERSEIVRAVIVRTCKELKRDNGMIIRYDDNAAVVIDQEGNPKGTRVFGAIARELRQLNFTKIVSLAPEVL : 122
Ise : MIQPQTHLNVADNSGARELMCIRIIGASNRRYAHIGDVIVAVIKEALPNMPLERSEIVRAVIVRTCKELKRDNGMIIRYDDNAAVVIDQEGNPKGTRVFGAIARELRQLNFTKIVSLAPEVL : 122
Isc : MIQPQTHLNVADNSGARELMCIRIIGASNRRYAHIGDVIVAVIKEALPNMPLERSEIVRAVIVRTCKELKRDNGMIIRYDDNAAVVIDQEGNPRGTRVFGAIARELRQLNFTKIVSLAPEVL : 122
Ikl : MIQPQTHLNVADNSGARELMCIRIIGASNRRYAHIGDVIVAVIKEALPNMPLERSEIVRAVIVRTCKELKRDNGMIIRYDDNAAVVIDQEGNPKGTRVFGAIARELRQLNFTKIVSLAPEVL : 122
Ipe : MIQPQTHLNVADNSGARELMCIRIIGASNRRYAHIGDVIVAVIKEALPNMPLERSEIVRAVIVRTCKELKRDNGMIIRYDDNAAVVIDQEGNPKGTRVFGAIPRELRRLNFTKIVSLAPEVL : 122
Ath : MIQPQTYLNVADNSGARELMCIRIIGASNRRYAHIGDVIVAVIKEAIPNTPLERSEVIRAVIVRTCKELKRNNGTIIRYDDNAAVVIDQEGNPKGTRVFGAIPRELRQLNFTKIVSLAPEVL : 122

**Figure S50 Multiple sequence alignment of the deduced amino acid sequences of RPL14 protein.** The origins of the protein sequences used in this alignment are from: *Arabidopsis thaliana* (Ath), *Iodes klaineana* (Ikl), *Iodes cirrhosa* (Ici), *Iodes seretii* (Ise), *Iodes scandens* (Isc), *Iodes perrieri* (Ipe) and *Iodes liberica* (Ili). The amino acid numbers for each sequence are indicated on the right. In the sequence alignment, identical residues are shown with a black background, and similar residues are shown with a gray background.

* 20 * 40 * 60 * 80 * 100 * 120
Isc : ------------------LFASSFRGAHS---RLTRTITQQKIRALVSAHRDRGRQKINFRRLWITRINAIIRERGVSNSYSRLIHNLYKRQLLLNRKILGQIAISNRNFLYMISKEIIK : 99
Ici : MTRVRRGYIARRRRTKIRLFASSFRGAHS---RLTRTITQQKIRALVSAHRDRGRQKINFRRLWITRINAIIRERGVSNSYSRLIHNLYKRQLLLNRKILGQIAISNRNFLYMISKEIIK : 117
Ikl : --------------TKILLFASSFRGAHS---RLTRTITQQKIRALVSAHRDRGRQKRNFRRLWITRINAIIRERGVSNSYSRLIHNLYKRQLLLNRKILGQIAISNRNFLYMISKEIIK : 103
Ipe : --------------TKILLFASSFRGAHS---RLTRTITQQKIRALVSAHRDRGRQKRNFRRLWITRINAIIRERGVSNSYSRLMHNLYKRQLLLNRKILGQIAISNRNFLYMISKEIIK : 103
Ili : --------------TKILLFASSFRGAHSRLTRLTRTITQQKIRALVSAHRDRGRQKRNFRRLWITRINAIIRERGVSNSYSRLIHNLYKRQLLLNRKILGQIAISNRNFLYMISKEIIK : 106
Ise : --------------TKILLFASSFRGAHS---RLTRTITQQKIRALVSAHRDRGRQKRNFRRLWITRINAIIRERGVSNSYSRLIHN--------------------------------- : 70
Ath : MTRIKRGYIARRRRTKLRLFASSFRGAHS---RLTRTMTQQRIRALVSAHRDRGKRKRDFRRLWITRINAVIHEMGVFYSYNEFIHNLYKKQLLLNRKILAQIALLNRSCLYTISNDIKK : 117

**Figure S51 Multiple sequence alignment of the deduced amino acid sequences of RPL20 protein.** The origins of the protein sequences used in this alignment are from: *Arabidopsis thaliana* (Ath), *Iodes klaineana* (Ikl), *Iodes cirrhosa* (Ici), *Iodes seretii* (Ise), *Iodes scandens* (Isc), *Iodes perrieri* (Ipe) and *Iodes liberica* (Ili). The amino acid numbers for each sequence are indicated on the right. In the sequence alignment, identical residues are shown with a black background, and similar residues are shown with a gray background.

* 20 * 40 * 60 * 80 * 100 * 120 *
Ikl : MLKKIK----TEVSASGKYISLSADKARRVIDQIRGRSYEETLMILELMPYRACYPLLKLVYSAAANASYNMGSTETNLVISKAEVNDGIFVKKFKPRARGR-----SYPIKRPTCHITIAVKDVSLDEYVE : 123
Ath : MIKKRKKKSYTEVYALGQYISMSAHKARRVIDQIRGRSYEEALMILELMPYRGCYPIFKLVYSAAANASHNKGFKETNLVISKAEVNQGNTVKKLKPRARGR-----SYPIKRSTCHITIVLEDISFYQQYE : 127
Ili : MLKKIK----TEVSASGKYISMSADKARRVIDQIRGRSYEETLMILELMPYRACYPLYKLVYSAAANASYNMGSTETNLVISKAEVNDGIFVKKLKPRARGR-----SYPIKRPTCHITIVVKDVSLDEYVE : 123
Ipe : MLKKIK----TEVSASGQYISMSADKARRVIDQIRGRSYEETLMILELMPYRACYPLLKLVYSAAANASYNMGSNETNLVISKAEVNDGIFVKKFKPRARGR-----SYPIKRPTCHITIVVKDVSLDEYVE : 123
Ici : MLKKIK----TEVSASGQYIYMSADKARRVIDQIRGRSYEETLMILELMPYRACYPLLKLVYSAAANASYNMGSNETNLVISKAEVNDGIFVKKFKPRARGR-----SYPIKRPTCHITIVVKDVSLDEYVD : 123
Ise : -LKKRK----TEVSASGQYISMSADKARRVIDQIRGRSYEERLMILELMPYRACYPLLKLVYSAAANATYNMGSNETNLVISKAEVNDGIFVKKFKPRARGRSYPIKSYPIKRPTCHITIVVKDVSLDEYVE : 127
Isc : --------------------------------------------------------------------------------------------------------------ILRPTLNLCLVVK--------- : 13

 140 * 160 *
Ikl : T--------------------AMTYYNMYSSGGVWDKK : 141
Ath : EYLM-YLK----KPGCSNENRNLTCYDTYSSGGLWDKK : 160
Ili : EDYLPWLK----KPRWKKKYTAMTYYNMYSSGGVWDKK : 157
Ipe : EDYLPWLKTMVKKPRWKKKYTAMTYYNIYSSGGVWDKK : 161
Ici : EDYLTWLK----KPRWKKKYTAMTYYNMYSSGGVWDKK : 157
Ise : EDYLPWLK----KPRWKKKYTAMTYYNMYSSGGVWDKK : 161
Isc : -------------------------------------- : -

**Figure S52 Multiple sequence alignment of the deduced amino acid sequences of RPL22 protein.** The origins of the protein sequences used in this alignment are from: *Arabidopsis thaliana* (Ath), *Iodes klaineana* (Ikl), *Iodes cirrhosa* (Ici), *Iodes seretii* (Ise), *Iodes scandens* (Isc), *Iodes perrieri* (Ipe) and *Iodes liberica* (Ili). The amino acid numbers for each sequence are indicated on the right. In the sequence alignment, identical residues are shown with a black background, and similar residues are shown with a gray background.

* 20 * 40 * 60 * 80 *
Isc : ---IRMLVLTDKSIRLLGKNQYTSNVESGSTRIEIKRWVELFFGVKVIAMNSHRLPGKGRRMGPIMGHTMHYRRMIITLQPGYSIPPLRKKR- : 89
Ise : MDGIKYSVFTDKSIRLLGKNQYTSNVESGSTRIEIKRWVELFFGVKVIAMNSHRLPGKGRRMGPIMGHTMHYRRMIITLQPGYSIPPLRKKRT : 93
Ikl : MDGIKYAVFTDKSIRLLGKNQYTSNVESGSTRIEIKRWVELFFGVKVIAMNSHRLPGKGRRMGPIMGHTMHYRRMIITLQPGYSIPPLRKKRT : 93
Ipe : MDGIKYAVFTDKSIRLLGKNQYTSNVESGSTRIEIKRWVELFFGVKVIAMNSHRLPGKGRRMGPIMGHTMHYRRMIITLQPGYSIPPLRKKRT : 93
Ici : MDGIKYAVFTDKSIRLLGKNQYTSNVESGSTRIEIKRWVELFFGVKVIAMNSHRLPGKGRRMGPIMGHTMHYRRMIITLQPGYSIPPLRKKRT : 93
Ili : MDGIKYAVFTDKSIRLLGKNQYTSNVESGSTRIEIKRWVELFFGVKVIAMNSHRLPGKGRRMGPIMGHTMHYRRMIITLQPGYSIPPLRKKRT : 93
Ath : MDGIKYAVFTDKSIRLLGKNQYTFNVESGSTRTEIKHWVELFFGVKVIAMNSHRLPGKVKRMGPILGHTMHYRRMIITLQPGYSIPPLRKKRT : 93

**Figure S53 Multiple sequence alignment of the deduced amino acid sequences of RPL23 protein.** The origins of the protein sequences used in this alignment are from: *Arabidopsis thaliana* (Ath), *Iodes klaineana* (Ikl), *Iodes cirrhosa* (Ici), *Iodes seretii* (Ise), *Iodes scandens* (Isc), *Iodes perrieri* (Ipe) and *Iodes liberica* (Ili). The amino acid numbers for each sequence are indicated on the right. In the sequence alignment, identical residues are shown with a black background, and similar residues are shown with a gray background.

* 20 * 40 *
Ikl : -----------------------GYWAALKAFSLGKSLSTGNSKSFFVRQTK : 29
Ici : MAVPKKRTSISKKRIRKRIWKRKGYWAALKAFSLGKSLSTGNSKSFFVRQTK : 52
Ili : MAVPKKRTSISKKRIRKRIWKRKGYWAALKAFSLGKSLSTGNSKSFFARQTK : 52
Ipe : MAVPKKRTSISKKRIRKRIWKRKGYWATLKAFSLGKSLSTGNSKSFFVRQT- : 51
Isc : MAVPKKRTSISKKRIRKRIWKRKGYWSALKAFSLGKSLSTGNSKSFFVRQT- : 51
Ise : MAVPKKRTSISKKRIRKRIWKRKGYWAALKAFSLGKSLSTGNSKSFFVRQ-- : 50
Ath : MAVPKKRTSISKKRIRKKIWKRKGYWTSLKAFSLGKSLSTGNSKSFFVQQNK : 52

**Figure S54 Multiple sequence alignment of the deduced amino acid sequences of RPL32 protein.** The origins of the protein sequences used in this alignment are from: *Arabidopsis thaliana* (Ath), *Iodes klaineana* (Ikl), *Iodes cirrhosa* (Ici), *Iodes seretii* (Ise), *Iodes scandens* (Isc), *Iodes perrieri* (Ipe) and *Iodes liberica* (Ili). The amino acid numbers for each sequence are indicated on the right. In the sequence alignment, identical residues are shown with a black background, and similar residues are shown with a gray background.

* 20 * 40 * 60
Isc : MAKGKDVRVTVILECTSCVQN--GVNKVSPGIS-------RHNTPNRLELRKFCPHCYKHTIHGEM-- : 57
Ici : MAKGKDVRVTVILECTSCVQN--GVNKVSPGISRYITQKNRHNTPNRLELRKFCPHCYKHTIHGEIKK : 66
Ili : MAKGKDVRVTVILECTSCVQN--GVNKVSPGISRYITQKNRHNTPNRLELRKFYPHCYKHTIHGEIKK : 66
Ipe : MAKGKDARVTVILECTSCVQN--GVNKVSPGISRYITQKNRHNTPNRLELRKFCPHCYKHTIHGEIKK : 66
Ikl : MAKGKDVRVTVILECTSCVQNSVNVNKVSPGISRYITQKNRHNTPNRLELRKFCPHCYKHTIHGEIKK : 68
Ise : MAKGKDVRVTVILECTSCVQN----NKASPGISRYITQKNRHNTHNRLELRKFCPHCYKHTIHGEIKK : 64
Ath : MAKGKDVRVTIILECTSCVRN--DIKKEAAGISRYITQKNRHNTPSRLELRKFCPYCYKHTIHGEIKK : 66

**Figure S55 Multiple sequence alignment of the deduced amino acid sequences of RPL33 protein.** The origins of the protein sequences used in this alignment are from: *Arabidopsis thaliana* (Ath), *Iodes klaineana* (Ikl), *Iodes cirrhosa* (Ici), *Iodes seretii* (Ise), *Iodes scandens* (Isc), *Iodes perrieri* (Ipe) and *Iodes liberica* (Ili). The amino acid numbers for each sequence are indicated on the right. In the sequence alignment, identical residues are shown with a black background, and similar residues are shown with a gray background.

* 20 * 40 * 60 * 80 * 100 * 120 *
Ikl : MVREKVTVSTRTLQWRCVESRADSKRLYYGRFILSPLMKGQADTIGIAMR----------RALLGEIEGTCITRAKSEKIPHEYSTIRGIQESVHEILMNLKEIVLRS-----NLYGTCDASICVKGPRYVT : 117
Ise : MVREKVTVSTRTLQWRCVESRADSKRLYYGRFILSPLMKGQADTIGIAMRRALLLGIAMRRALLGEIEGTCITRAKSEKIPHEYSTIRGIQESVHEILMNLKEIVLRS-----NLYGTCGASICVKGPRYVT : 127
Ipe : MVREKVKVSTRTLQWRCVESRADSKRLYYGRFILSPLMKGQADTIGIAMR----------RALLGEIEGTCITRAKSEKIPHEYSTIRGIQESVHEILMNLKEIVLRS-----NLYGTCDASICVKGPRYVT : 117
Isc : MVREKVTVSTRTLQWRCVESRADSKRLYYGRFILSPLMKGQADTIGIAMR----------RALLGEIEGTCITRAKSEKIPHEYSTIRGIQESVHEIRMNLKEIVLRSNLYKSNLYGTCDASICVKGPRYVT : 122
Ici : MVREKVTVSTRTLQWRCVESRADSKRLYYGRFILSPLMKGQADTIGIAMR----------RALLGEIEGTCITRAKSEKIPHEYSTIRGIQESVHEILMNLKEIVLRS-----NLYGTCDASICVKGPRYVT : 117
Ili : MVREKVTVSTRTLQWRCVESRADSKRLYYGRFILSPLMKGQADTIGIAMR----------RALLGEIEGTCITRAKSEKIPHEYSTIRGIQS-VHEILMNLKEIVLRS-----NLYGTCDASICVKGPRYVT : 116
Ath : MVREKVKVSTRTLQWKCVESKRDSKRLYYGRFILSPLMKGQADTIGIAMR----------RALLGEIEGTCITRAKSENIPHDYSNIAGIQESVHEILMNLNEIVLRS-----NLYGTRNALICVQGPGYIT : 117

 140 * 160 * 180 * 200 * 220 * 240 * 260
Ikl : AQDIILPPYIEIVDNRQHIASLTEPIELRIGLQIERNRGYLIKIPNNFQDGSYPIDAVFMPVRNANHSIHSYGTGNEKQEILFLEIWTNGSLTPKEALHEASRNLIDFFITFLHTEEENLHLEDNQHTVPLS : 249
Ise : AQDIILPPYIEIVDNRQHIASLTEPIELRIGLQIERNRGYLIKTPNNFQDGSYPIDAVFMPVRNANHSIHSYGTGNEKQEILFLEIWTNGSLTPKEALHEASRNLIDFFITFLHTEEENLHLEDNQHTVPLS : 259
Ipe : AQDIILPPYIEIVDNRQHIASLTEPIELRIGLQIERNRGYLIKTPNNFQDGSYPIDAVFMPVRNANHSIHSYGTGNEKQEILFLEIWTNGSLTPKEALHEASRNLIDFFITFLHTEEENLHLEDNQHTVPLS : 249
Isc : AQDIILPPYIEIVDNRQHI-----------------------------------------------HSIHSYGTGNEKQEILFLEIWTNGSLTPKEALHEASRNLIDFFITFLHTEEENLHLEDNQHMVPLS : 207
Ici : AQDIILPPYIEIVDNRQHIANLTEPIELRIGLQIERNRGYLIKTRNNFQDGSYPIDAVFMPVRNANHSIHSYGTGNEKQEILFLEIWTNGSLTPKEALHEASRNLIDFFITFLHTEEENLHLEDNQHMVPLS : 249
Ili : AQDIILPP---------------------------------------------------------------------------------------------------------------------------- : 124
Ath : ARDIILPPAVEIIDNTQHIATLTEPIDLCIELKIERNRGYSLKMSNNFEDRSYPIDAVFMPVENANHSIHSYGNGNEKQEILFLEIWTNGSLTPKEALHQASRNLINLFIPFLHVEEETFYLENNQHQVTLP : 249

 * 280 * 300 * 320 * 340 *
Ikl : PFIFHD------------------------------------------------------------------------------------- : 255
Ise : PFIFHDKLAKLRKNK-KKT-TLKSIFIDQLELPSRIYNCLKGSNI-----------------EHLRLEDVKQILGILEKHFEIDLPKKKF- : 330
Ipe : PFIFHDKLAKLRKNK-KKT-ALKSIFIDQLELPSRIYNCLKGSNIYTLLDLL--SQEDLMKIEHLRLEDVKQILGILEKHFAIDLPKKSLN : 336
Isc : PFIFHDKLSKLRKNK-KKTAALKSIFIDQSELPSRIYNCLKGSNIYTLLDLLNNSQEDLMKIEHLHLEDVKQILGILEKHFAIDLPKKRF- : 296
Ici : PFIFHDKLSKLRKNK-KKT-ALKSIFIDQSELPSRIYNCLKGSNIYTLLDLLNNSQEDLMKIEHLRLEDVKRILGILEKHFAIDLPKKSFN : 338
Ili : ------------------------------------------------------------------------------------------- : -
Ath : FFPFHNRLVNLRKKKKTKELAFQYIFIDQLELPPRIYNCLKKSNIHTLLDLLNNSQEDLIKIEHFHVEDVKKILDILEKK----------- : 329

**Figure S56 Multiple sequence alignment of the deduced amino acid sequences of RPOA protein.** The origins of the protein sequences used in this alignment are from: *Arabidopsis thaliana* (Ath), *Iodes klaineana* (Ikl), *Iodes cirrhosa* (Ici), *Iodes seretii* (Ise), *Iodes scandens* (Isc), *Iodes perrieri* (Ipe) and *Iodes liberica* (Ili). The amino acid numbers for each sequence are indicated on the right. In the sequence alignment, identical residues are shown with a black background, and similar residues are shown with a gray background.

* 20 * 40 * 60 * 80 * 100 * 120
Ikl : MFQDENEQMSTIPSFNQIQFEGFCRFIDQGLTEELYKFPKIEDTDQEIEFQLFVETYQLVEPLIKERDAVYESLTYSSELYVSAGLIWKN-SRDMQEQTIFIGNIPLMNSLGTSIVNGIYRIVINQILQ : 128
Ili : MFQDENEQMSTIPSFNQIQFEGFCRFIDQGLTEELYKFPKIEDTDQEIEFQLFVETYQLVEPLIKERDAVYESLTYSSELYVSAGLIWKN-SRDMQEQTIFIGNIPLMNSLGTSIVNGIYRIVINQILQ : 128
Ipe : MFQDENEQMSTIPSFNQIQFEGFCRFIDQGLTEELYKFPKIEDTDQEIEFQLFVETYQLVEPLIKERDAVYESLTYSSELYVSAGLIWKN-SRDMQEQTIFIGNIPLMNSLGTSIVNGIYRIVINQILQ : 128
Ici : MFQDENEQMSTIPSFNQIQFEGFCRFIDQGLTEELYKFPKIEDTDQEIEFQLFVETYQLVEPLIKERDAVYESLTYSSELYVSAGLIWKN-SRDMQEQTIFIGNIPLMNSLGTSIVNGIYRIVINQILQ : 128
Ise : MFQ---EQMSTIPSFNQIQFEGFCRFIDQGLTEELYKFPKIEDTDQEIEFQLFVETYQLVEPLIKERDAVYESLTYSSELYVSAGLILKNNSRDMQEQTIFIGNIPLMNSLGTSIVNGIYRIVINQILQ : 126
Isc : -----------------------------------------------------------------------------------------------------LNNIPLMNSLGTSIVNGIYRIVINQILQ : 28
Ath : MLGDEKEGTSAIPGFNQIQFEGFYRFIDQGLIEELAKFPKIEDIDHEIEFQLFVETYQLVEPLIKERDAVYESLTYSSELYVSAGLIWKT-SRNMQEQRIFIGNIPLMNSLGTSIVNGIYRIVINQILQ : 128

 * 140 * 160 * 180 * 200 * 220 * 240 * 2
Ikl : SPGIYYRSELDHNGISVYTGTIISDWGGRSELEIDRKARIWARVSRKQKISILVLSSAMGSNLREILENVCYPEIFLSFLNDKERKKIGLKENAILEFYQQFACVGGDPVFSESLCKELQKKFFQQRCE : 257
Ili : SPGIYYRSELDHNGISVYTGTIISDWGGRSELEIDRKARIWARVSRKQKISILVLSSAMGSNLREILENVCYPEIFLSFLNDKERKKIGLKENAILEFYQQFACVGGDPVFSESLCKELQKKFFQQRCE : 257
Ipe : SPGIYYRSELDHNGISVYTGTIISDWGGRSELEIDRKARIWARVSRKQKISILVLSSAMGSNLREILENVCYPEIFLSFLNDKERKKMGLKENAILEFYQQFACVGGDPVFSESLCKELQKKFFQQRCE : 257
Ici : SPGIYYRSELDHNGISVYTGTIISDWGGRSELEIDRKARIWARVSRKQKISILVLSSAMGSNLREILENVCYPEIFLSFLNDKERKKIGLKENAILEFYQQFACVGGDPVFSESLCKELQKKFFQQRCE : 257
Ise : SPGIYYRSELDHNGISVYTGTIISDWGGRSELEIDRKARIWARVSRKQKISILVLSSAMGSNLREILENVCYPEIFLSFLNDKERKKIGLKENAILEFYQQFACVGGDPVFSESLCKELQKKFFQQRCE : 255
Isc : SPGIYYRSELDHNGISVYTGTIISDWGGRSELEIDRKARIWARVSRKQKISILVLSSAMGSNLREILENVCYPEIFLSFLNDKERKKIGLKENAILEFYQQFACVGGDPVFSESLCKELQKKFFQQRCE : 157
Ath : SPGIYYQSELDHNGISVYTGTIISDWGGRLELEIDKKARIWARVSRKQKISILVLSSAMGLNLREILENVCYPEIFLSFLTDKEKKKIGSKENAILEFYQQFSCVGGDPIFSESLCKELQKKFFHQRCE : 257

 60 * 280 * 300 * 320 * 340 * 360 * 380
Ikl : LGRIGRRNINQRLNLDIPQNNTFLLPRDILAAADHLIGMKFGMGTLDDMNHLKNKRIRSVADLLQDQFGLALVRLENVVRGTICGAIRHKLIPTPQNLVTSTPLTTTYESFFGLHPLSQVLDRTNPLTQ : 386
Ili : LGRIGRRNINQRLNLDIPQNNTFLLPRDILAAADHLIGMKFGMGTLDDMNHLKNKRIRSVADLLQDQFGLALVRLENVVRGTICGAIRHKLIPTPQNLVTSTPLTTTYESFFGLHPLSQVLDRTNPLTQ : 386
Ipe : LGRIGRRNINQRLNLDIPQNNTFLLPRDILAAADHLIGMKFGMGTLDDMNHLKNKRIRSVADLLQDQFGLALVRLENVVRGTICGAIRHKLIPTPQNLVTSTPLTTTYESFFGLHPLSQVLDRTNPLTQ : 386
Ici : LGKIGRRNINQRLNLDIPQNNTFLLPRDILAAADHLIGMKFGMGTLDDMNHLKNKRIRSVADLLQDQFGLALVRLENVVRGTICGAIRHKLIPTPQNLVTSTPLTTTYESFFGLHPLSQVLDRTNPLTQ : 386
Ise : LGRIGRRNINQRLNLDIPQNNTFLLPRDILAAADHLIGMKFGMGTLDDMNHLKNKRIRSVADLLQDQFGLALVRLENVVRGTICGAIRHKLIPTPQNLVTSTPLTTTYESFFGLHPLSQVLDRTNPLTQ : 384
Isc : LGKIGRRNINQRLNLDIPQNNTFLLPRDILAAADHLIGMKFGMGTLDDINHLKNKRIRSVADLLQDQFGLALVRLENVVRGTICGAIRHKLIPTPQNLVTSTPLTTTYESFFGLHPLSQVLDRTNPLTQ : 286
Ath : LGRIGRRNINWRLNLNIPQNNIFLLPRDVLAAADHLIGMKFGMGTLDDMNHLKNKRIRSVADLLQDQLGLALARLENVVKGTISGAIRHKLIPTPQNLVTSTPLTTTYESFFGLHPLSQVLDRTNPLTQ : 386

* 400 * 420 * 440 * 460 * 480 * 500 *
Ikl : IVHGRKLSYLGPGGLTGRTASFRIRDIHPSHYGRICPIDTSEGINVGLIGSLAIHARISHWGFLETPFFEIYERS--RGVRILYLSPGIDEYYMVAGGNSLALN-----QGIQEEQVVPTRYRQEFLTI : 508
Ili : IVHGRKLSYLGPGGLTGRTASFRIRDIHPSHYGRICPIDTSEGINVGLIGSLAIHARISHWGFLETPFFEIYERS--TGVRILYLSPGIDEYYMVAGGNSLALNSLALNQGIQEEQVVPTRYRQEFLTI : 513
Ipe : IVHGRKLSYLGPGGLTGRTASFRIRDIHPSHYGRICPIDTSEGINVGLIGSLAIHARISHWGFLETPFYEIYERS--TGVRILYLSPGIDEYYMVAGGSSLALN-----QGIQEEQVVPTRYRQEFLTI : 508
Ici : IVHGRKLSYLGPGGLTGRTASFRIRDIHPSHYGRICPIDTSEGINVGLIGSLAIHARIGHWGFLETPFYEIYERS--TGVRILYLSPGIDEYYMVAGGNSLALN-----QGIQEEQVVPTRYRQEFLTI : 508
Ise : IVHGRKLSYLGPGGLTGRTASFRIRDIHPSHYGRICPIDTSEGINVGLIGSLAIHARISHWGFLETPFYEIYERS--TGVRILYLSPGIDEYYMVAGGNSLALN-----QGIQEEQVVPTRYRQEFLTI : 506
Isc : IVHGRKLSYLGPGGLTGRTASFRIRDIHPSHYGRICPIDTSEGINVGLIGSLAIHARIGHWGFLETPFYEIYERS--TGVRIRYLSPGIDEYYMVAGGNSLALN-----QGIQEEQVVPTRYRQEFLTI : 408
Ath : IVHGRKLSYLGPGGLTGRTANFRIRDIHPSHYGRICPIDTSEGINVGLIGSLSIHARIGDWGSLESPFYELFEKSKKARIRMLFLSPSQDEYYMIAAGNSLALN-----RGIQEEQAVPARYRQEFLTI : 510

 520 * 540 * 560 * 580 * 600 * 620 * 640
Ikl : AWEQVHLRSIFPFQFFSIGASLIPFIEHNDANRALMSSNMQRQAVPLSRSEKCIVGTGLERQAALDSGALAIAEREGKIIYTDTGKILLSGNGDTLSIPLVTYQRSNKNTCMHQKPQVPRGKCIKKGQI : 637
Ili : AWEQVHLRSIFPFQYFSIGASLIPFIEHNDANRALMSSNMQRQAVPLSRSEKCIVGTGLERQAALDSGALAIAEREGKIIYTDTGKILLSGNGDTLSIPLVTYQRSNKNTCMHQKPQVPRGKCIKKGQI : 642
Ipe : AWEQVHLRSIFPFQYFSIGASLIPFIEHNDANRALMSSNMQRQAVPLSRSEKCIVGTGLERQAALDSGALAIAEREGKIIYTDTGKILLSGNGDTLSIPLVTYQRSNKNTCMHQRPQVPRGKCIKKGQI : 637
Ici : AWEQVHLRSIFPFQYFSIGASLIPFIEHNDANRALMSSNMQRQAVPLSRSEKCIVGTGLERQAALDSGALAIAEREGKIIYTDTGKILLSGNGDTLSIPLVTYQRSNKNTCMHQKPQVPRGKCIKKGQI : 637
Ise : AWERVHLRSIFPFQYFSIGASLIPFIEHNDANRALMSSNMQRQAVPLSRSEKCIVGTGLERQAALDSGALAIADREGKIIYTDTGKILLSGNGDTLSIPLVTYQRSNKNTCMHQKPQVPRGKCIKKGQI : 635
Isc : AWEQVHLRSIFPFQYFSIGASLIPFIEHNDANRALMSSNMQRQAVPLSRSEKCIVGTGLERQAALDSGALAIAEREGKIIYTDTGKILLSGNGDTLSIPLVTYQRSNKNTCMHQKPQVPRGKCIKRGQI : 537
Ath : AWEEVHLRSIFPFQYFSIGASLIPFIEHNDANRALMSSNMQRQAVPLSRSEKCIVGTGLERQVALDSGVPAIAEHEGKILYTDTEKIVFSGNGDTLSIPLIMYQRSNKNTCMHQKPQVRRGKCIKKGQI : 639

 * 660 * 680 * 700 * 720 * 740 * 760 *
Ikl : LADGAATVGGELALGKNVLVAYMPWEGYNFEDAVLISERLVYEDIYTSFHIRKYEIQIHVTSQGPERVTNEIPHLEARLLRNLDKNGIVMLGSWVETGDILVGKLTPQIVKESSYAPEDRLLRAILGIQ : 766
Ili : LADGAATVGGELALGKNVLVAYMPWEGYNFEDAVLISERLVYEDIYTSFHIRKYEIQIHVTSQGPERVTNEIPHLEARLLRNLDKNGIVMLGSWVETGDILVGKLTPQIVKESSYAPEDRLLRAILGIQ : 771
Ipe : LADGAATVGGELALGKNVLVAYMPWEGYNFEDAVLISERLVYEDIYTSFHIRKYEIKIHVTSQGPERVTNEIPHLEARLLRNLDKNGIVMLGSWVETGDILVGKLTPQIVKESSYAPEDRLLRAILGIQ : 766
Ici : LADGAATVGGELALGKNVLVAYMPWEGYNFEDAVLISERLVYEDIYTSFHIRKYEIKIHVTSQGPERVTNEIPHLEARLLRNLDKNGIVMLGSWVETGDILVGKLTPQIVKESSYAPEDRLLRAILGIQ : 766
Ise : LADGAATVGGELALGKNVLVAYMPWEGYNFEDAVLISERLVYEDIYTSFHIRKYEIKIHVTSQGPERVTNEIPHLEARLLRNLDKNGIVMLGSWVETGDILVGKLTPQIVKESSYAPEDRLLRAILGIQ : 764
Isc : LADGAATVGGELALGKNVLVAYMPWEGYNFEDAVLISERLVYEDIYTSFHIRKYEIKIHVTSQGPERVTKEIPHLEARLLRNLDKNGIVMLGSWVETGDILVGKLTPQIVKESSYAPEDRLLRAILGIQ : 666
Ath : LADGAATVGGELALGKNILVAYMPWEGYNFEDAVLISECLVYGDIYTSFHIRKYEIQTHVTTQGPERITKEIPHLEGRLLRNLDKNGIVMLGSWVETGDILVGKLTPQVAKESSYAPEDRLLRAILGIQ : 768

780 * 800 * 820 * 840 * 860 * 880 * 900
Ikl : VSTSKETCLKLPIGGRGRVIDVRWIQKKGGSAYNPEMIRVYISQKREIKVGDKVAGRHGNKGIISKILSRQDMPYLQDGRPVDMVFN----PLGVPSRMNVGQIFECSLGLAGGLLDRHYRIVPFDERY : 891
Ili : VSTSKETCLKLPIGGRGRVIDVRWIQKKGGSAYNPEMIRVYISQKREIKVGDKVAGRH-NKGIISKILSRQDMPYLQDGRPVDMVFN----PLGVPSRMNVGQIFECSLGLAGGLLDRHYRIVPFDERY : 895
Ipe : VSTSKETCLKLPIGGRGRVIDVRWIQKKGGSAYNPEMIRVYISQKREIKVGDKVAGRHGNKGIISKILSRQDMPYLQDGRPVDMVFN----PLGVPSRMNVGQIFECSLGLAGGLLDRHYRIVPFDERY : 891
Ici : VSTSKETCLKLPIGGRGRVIDVRWIQKKGGSAYNPEMIRVYISQKREIKVGDKVAGRHGNKGIISKILSRQDMPYLQDGRPVDMVFN----PLGVPSRMNVGQIFECSLGLAGGLLDRHYRIVPFDERY : 891
Ise : VSTSKETCLKLPIGGRGRVIDVRWIQKKGGSAYNPEIIRVYISQKREIKVGDKIAGRHGNKGIISKILSRQDMPYLQDGRPVDMVFN----PLGVPSRMNVGQIFECSLGLAGGLLDRHYRIVPFDERY : 889
Isc : VSTSKETCLKLPIGGRGRVIDVRWIQKKGGSAYNPEMIRVYISQKREIKVGDKVAGRHGNKGIISKILSRQDMPYLQDGRPVDMVFNPLGVPLGVPSRMNVGQIFECSLGLAGGLLDRHYRIVPFDERY : 795
Ath : VSTSKETCLKLPIGGRGRVIDVRWVQKKGGSSYNPEIIRVYISQKREIKVGDKVAGRHGNKGIISKILPRQDMPYLQDGRPVDMVFN----PLGVPSRMNVGQIFECSLGLAGSLLDRHYRIAPFDERY : 893

 * 920 * 940 * 960 * 980 * 1000 * 1020 *
Ikl : EQEASRKLVFSELYEASKQTANPWVFEPEYPGKSRILDGRTGYPFEQPVIIGKPYILKLIHQVDDKIHGRSSGHYALVTQQPLRGKAKQGGQRVGEMEVWALEGFGVAHILQEMLTYKSDHIRARQEAL : 1020
Ili : EQEASRKLVFSELYEASKQTANPWVFEPEYPGKSRILDGRTGYPFEQP--------------------------------------------------------------------------------- : 943
Ipe : EQEASRKLVFAELYEASKQTANPWVFEPEYPGKSRILDGRTGYPFEQPVIIGKPYILKLIHQVDDKIHGRSSGHYALVTQQPLRGKAKQGGQRVGEMEVWALEGFGVAHILQEMLTYKSDHIRARQEAL : 1020
Ici : EQEASRKLVFSELYEASKQTANPWVFEPEYPGKSRILDGRTGYPFEQPVIIGKPYILKLIHQVDDKIHGRSSGHYALVTQQPLRGKAKQGGQRVGEMEVWALEGFGVAHILQEMLTYKSDHIRARQEAL : 1020
Ise : EQEASRKLVFSELSEASKQTANPWVFEPEYPGKSRILDGRTGYPFEQPVIIGKPYILKLIHQVDDKIHGRSSGHYALVTQQPLRGKAKQGGQRVGEMEVWALEGFGVAHILQEMLTYKSDHIRARQEAL : 1018
Isc : EQEASRKLVFSELYEASKQTANPWVFEPEYPGKSRILDGRTGYPFEQPVIIGKPYILKLIHQVDDKIHGRSSGHYALVTQQPLRGKAKQGGQRVGEMEVWALEGFGVAHILQEMLTYKSDHIRARQEAL : 924
Ath : EQEASRKLVFSELYEASKQTANPWVFEPEYPGKSRIFDGRTGDPFEQPVIIGKPYILKLIHQVDDKIHGRSSGHYALVTQQPLRGRSKQGGQRVGEMEVWALEGFGVAHILQEMLTYKSDHIRARQEVL : 1022

 1040 * 1060 * 1080
Ikl : GTTIIGGTIPNPEDAPESFRLLVRELRSLALELNHFLVSEKNFQINRKEA : 1070
Ili : -------------------------------------------------- : -
Ipe : GTTIIGGTIPNPEDAPESFRLLVRELRSLALELNHFLVSEKNFQINRKEA : 1070
Ici : GTTIIGGTIPNPEDAPESFRLLVRELRSLALELNHFLVSEKNFQINRKEA : 1070
Ise : GTTIIGGTIPNPEDAPESFRLLVRELRSLALELNHFLVSEKNFQINRKEA : 1068
Isc : GTTIIGGTIPNPEDAPESFRLLVRELRSLALELNHFLVSEKNFQINRKEA : 974
Ath : GTTIIGGTIPKPEDAPESFRLLVRELRSLALELNHFLVSEKNFQINRKEV : 1072

**Figure S57 Multiple sequence alignment of the deduced amino acid sequences of RPOB protein.** The origins of the protein sequences used in this alignment are from: *Arabidopsis thaliana* (Ath), *Iodes klaineana* (Ikl), *Iodes cirrhosa* (Ici), *Iodes seretii* (Ise), *Iodes scandens* (Isc), *Iodes perrieri* (Ipe) and *Iodes liberica* (Ili). The amino acid numbers for each sequence are indicated on the right. In the sequence alignment, identical residues are shown with a black background, and similar residues are shown with a gray background.

* 20 * 40 * 60 * 80 * 100 * 120 *
Ipe : MIDRYKHQQLRIGSVSPEQISAWANKILPNGEIVGEVTKPYTFHYKTNKPEKDGLFCERIFGPIKSGICACGNYRVIGDEKEDPKFCEQCGVEFTDSRIRRYQMGYIKLACPVDSRIRRYQMG-YIK--- : 126
Ili : MIDRYKHQQLRIGSVSPEQISAWANKILPNGEIVGEVTKPYTFHYKTNKPEKDGLFCERIFGPIKSGICACGNYRVIGDEKEDPKFCEQCGVEFTDSRIRRYQMGYIKLACPVTHVWYLKRLPSYIANLL : 130
Ikl : MIDRYKHQHLRIGSVSPEQISAWANKILPNGEIVGEVTKPYTFHYKTNKPEKDGLFCERIFGPIKSGICACGNYRVIGDEKEDPKFCEQCGVEFTDSRIRRYQMGYIKLACPVTHVWYLKRLPSYIANLL : 130
Ici : MIDRYKHQQLRIGSVSPQQISAWANKILPNGEIVGEVTKPYTFHYKTNKPEKDGLFCERIFGPIKSGICACGNYRVIRDEKEDPKFCEQCGVEFTDSRIRRYQMGYIKLACPVTHVWYLKRLPSYIANLL : 130
Ise : MIDRYKHQQLRIGSVSPEQISAWANKILPNGEIVGEVTKPYTFHYKTNKPEKDGLFCERIFGPIKSGICACGNYRVIGDEKEDPKFCEQCGVEFTDSRIRRYQMGYIKLACPVTHVWYLKRLPSYIANLL : 130
Isc : MIDRYKHQQLRIGSVSPQQISAWANKILPNGEIVGEVTK-------------------------------------------------------------------IKLACPVTHVWYLKRLPSYIANLL : 63
Ath : MIDRYKHQQLRIGLVSPQQISAWATKIIPNGEIVGEVTKPYTFHYKTNKPEKDGLFCERIFGPIKSGICACGNYRVIGDEKEDPKFCEQCGVEFVDSRIRRYQMGYIKLTCPVTHVWYLKRLPSYIANLL : 130

 140 * 160 * 180 * 200 * 220 * 240 * 260
Ipe : ----------LACPVFSFARPITKKPTFLRLRGSFEYEIQSWKYSIPLFFTTQGFDTFRNREISTGAGAVREQLADLDLRIIVDNSLVEWKELGEDGPTGNEWEDRKVGRRKDFLVRRMELAKNFIRTNI : 246
Ili : DKPLKELEGLVYCDVFSFARPITKKPTFLRLRGSFEYEIQSWKYSIPLFFTTQGFDTFRNREISTGAGAVREQLADLDLRIIVDNSLVEWKELGEDGPTGNEWEDRKVGRRKDFLVRRMELAKHFIRTNI : 260
Ikl : DKPLKELEGLVYCDVFSFARPITKKPTFLRLRGSFEYEIQSWKYSIPLFFTTQGFDTFRNREISTGAGAVREQLADLDLRIIVDNSLVEWKELGEDGPTGNEWEDRKVGRRKDFLVRRMELAKHFIRTNI : 260
Ici : DKPLKELEGLVYCDVFSFARPIAKKPTFLRLRGSFEYEIQSWKYSIPLFFTTQGFDTFRNREISTGAGAVREQLADLDLRIIVDNSLVEWKELGEDGPTGNEWEDRKVGRRKDFLVRRMELAKHFIRTNI : 260
Ise : DKPLKELEGLVYCDVFSFARPITKKPTFLRLRGSFEYEIQSWKYSIPLFFTIQGFDTFRNREISTGAGAVREQLADLDLRIIVDNSLVEWKELGEDGPTGNEWEDRKVGRRKDFLVRRMELAKHFIRTNI : 260
Isc : DKPLKELEGLVYCDVFSFARPIAKKPTFLRLRGSFEYEIQSWKYSIPLFFTTQDFDTFCNREISTGAGAVREQLADLDLRIIVDNSLVEWKELGEDGPTGNEWEDRKVGRRKDFLVRRMELAKHFIRTNI : 193
Ath : DKPLKELEGLVYCD-FSFARPITKKPTFLRLRGSFEYEIQSWKYSIPLFFTTQGFDIFRNREISTGAGAIREQLADLDLRIIIENSLVEWKQLGEEGPTGNEWEDRKIVRRKDFLVRRMELAKHFIRTNI : 259

 * 280 * 300 * 320 * 340 * 360 * 380 *
Ipe : EPEWMVLCLLPVLPPELRPIIQIDGGKLMSSDINELYRRVIYRNNTLTDLLTTSRSTPGELVMCQEKLVQEAVDTLLDNGIRGQPMRDGHNKVYKSFSDVIEGKEGRFRETLLGKRVDYSGRSVIVVGPS : 376
Ili : EPEWMVLCLLPVLPPELRPIIQIDGGKLMSSDINELYRRVIYRNNTLTDLLTTSRSTPGELVMCQEKLVQEAVDTLLDNGIRGQPMRDGHNKVYKSFSDVIEGKEGRFRETLLGKRVDYSGRSVIVVGPS : 390
Ikl : EPEWMVLCLLPVLPPELRPIIQIDGGKLMSSDINELYRRVIYRNNTLTDLLTTSRSTPGELVMCQEKLVQEAVDTLLDNGIRGQPMRDGHNKVYKSFSDVIEGKEGRFRETLLGKRVDYSGRSVIVVGPS : 390
Ici : EPEWMVLCLLPVLPPELRPIIQIDGGKLMSSDINELYRRVIYRNNTLTDLLTTSRSTPGELVMCQEKLVQEAVDTLLDNGIRGQPMRDGHNKVYKSFSDVIEGKEGRFRETLLGKRVDYSGRSVIVVGPS : 390
Ise : EPEWMVLCLLPVLPPELRPIIQIDGGKLMSSDINELYRRVIYRNNTLTDLLTTSRSTPGELVMCQEKLVQEAVDTLLDNGIRGQPMRDGHNKVYKSFSDVIEGKEGRFRETLLGKRVDYSGRSVIVVGPS : 390
Isc : EPEWMVLCLLPVLPPELRPIIQIDGGKLMSSDINELYRRVIYRNNTLTDLLTTSRSTPGELVMCQEKLVQEAVDTLLDNGIRGQPMRDGHNKVYKSFSDVIEGKEGRFRETLLGKRVDYSGRSVIVVGPS : 323
Ath : EPEWMVLCLLPVLPPELRPIIQIEGGKLMSSDINELYRRVIYRNNTLTDLLTTSRSTPGELVMCQEKLVQEAVDTLLDNGIRGQPMRDGHNKVYKSFSDVIEGKEGRFRETLLGKRVDYSGRSVIVVGPS : 389

400 * 420 * 440 * 460 * 480 * 500 * 520
Ipe : LSLHRCGLPREVAIELFQTFLIRGLIRQHLASNIGVAKSKIREKE-----PIVWEILQKVMRGHPLLLNRAPTLHRLGIQAFHPVLVEGRAICLHPLVCKGFNADFDGDQMAVHVPLSLEAQAEARLLMF : 501
Ili : LSLHRCGLPREIAIELFQAFLIRGLIRQHLASNIGVAKSQIREKEPIVKEPIVWEILQEVMRGHPLLLNRAPTLHRLGIQAFHPVLVEGRAICLHPLVCKGFNADFDGDQMAVHVPLSLEAQAEARLLMF : 520
Ikl : LSLHRCGLPREIAIELFQAFLIRGLIRQHLASNIGVAKSKIREKE-----PIVWEILQEVMRGHPLLLNRAPTLHRLGIQAFHPVLVEGRAICLHPLVCKGFNADFDGDQMAVHVPLSLEAQAEARLLMF : 515
Ici : LSLHQCGLPREIAIELFQTFLIRGLIRQHLASNIGVAKSKIREKE-----PIVWEILQEVMRGHPLLLNRAPTLHRLGIQAFHPVLVEGRAICLHPLVCKGFNADFDGDQMAVHVPLSLEAQAEARLLMF : 515
Ise : LSLHRCGLPREIAIELFQTFVIRGLIRQHLASNIGVAKSKIREKE-----PIVWEILQEVMRGHPLLLNRAPTLHRLGIQAFHPVLVEGRAICLHPLVCKGFNADFDGDQMAVHVPLSLEAQAEARLLMF : 515
Isc : LSLHQCGLPREIAIELFQTFLIRGLIRQYLASNIGVAKSKIREKE-----PIVWEILQEVMRGHPLLLNRAPTLHRLGIQAFHPVLVEGRAICLHPLVCKGFNADFDGDQMAVHVPLSLEAQAEARLLMF : 448
Ath : LSLHRCGLPREIAIELFQTFVIRGLIRQHLASNIGVAKSQIREKK-----PIVWEILQEVMQGHPVLLNRAPTLHRLGIQSFQPILVEGRTICLHPLVCKGFNADFDGDQMAVHVPLSLEAQAEARLLMF : 514

 * 540 * 560 * 580 * 600 * 620 * 640 *
Ipe : SHMNLLSPAIGDPISIPTQDMLIGLYILTSGNPRGICVNRYNPCNQRNYQNERREKNNYKYTKKKEPFFCNSYDAIGAYRQKRINLDSPLWLRWRLDQRVIASREVPLEVHYESLGTYYEIYGHYLIVRS : 631
Ili : SHMNLLSPAIGDPISIPTQDMLIGLYILTSGNRRGICVNRYNPCNQRNYQNERREKNNYKYTKKKEPFFCNSYDAIGAYRQKRINLDSPLWLRWRLDQRVIASREAPLEVHYESLGTYYEIYGHYLIVRS : 650
Ikl : SHMNLLSPAIGDPISIPTQDMLIGLYILTSGNRRGICVNRYNPCNQRNYQ--RREKNNYKYTKKKEPFFCNSYDAIGAYRQKRINLDSPLWLRWRLDQRVIASREAPLEVHYESLGTYYEIYGHYLIVRS : 643
Ici : SHMNLLSPAIGDPISIPTQDMLIGLYILTSRNRRGICVNRYNPCNQRNYQNERREKNNYKYTKKKEPFFCNSYDAIGAYRQKRINLDSPLWLRWRLDQRVIASREAPLEVHYESLGTYYEIYGHYLIVRN : 645
Ise : SHMNLLSPAIGDPISIPTQDMLIGLYILTSWNRRGICVNRYNPCNQRNYQNERREKNNYKYTKKKEPFFCNSYDAIGASRQKRINLDSPLWLRWRLDQRVIASREAPLEVHYESLGTYYEIYGHYLIVRS : 645
Isc : SHMNLLSPAIGDPISIPTQDMLIGLYILTSGNRRGICVNRYNPCNQRNYQNERREKKEVGYRKER----------------------------------------------------------------- : 513
Ath : SHMNLLSPAIGDPISVPTQDMLIGLYVLTSGTRRGICANRYNPCNRKNYQNERIYETNYKYTK--EPFFCNSYDAIGAYRQKKINLDSPLWLRWQLDQRVIASREVPIEVHYESFGNYHEIYAHYLIVRS : 642

 660 * 680
Ipe : IKKEILYIYIRTTVGHISLYREIEEAIQGFCQASFYDT : 669
Ili : IKKEILYIYIRTTVGHISLYREIEEAIQGFCQASFYDT : 688
Ikl : IKKEILYIYIRTT------YREIEEAIQGFCQASFYDT : 675
Ici : IKKEILYIYIRTTVGHISLYREIEEAIQGFCQASFYDT : 683
Ise : IKKE---------------------------------- : 649
Isc : -------------------------------------- : -
Ath : VKKENFCIYIRTTVGHISFYREIEEAIQGFSQACSYDT : 680

**Figure S58 Multiple sequence alignment of the deduced amino acid sequences of RPOC1 protein.** The origins of the protein sequences used in this alignment are from: *Arabidopsis thaliana* (Ath), *Iodes klaineana* (Ikl), *Iodes cirrhosa* (Ici), *Iodes seretii* (Ise), *Iodes scandens* (Isc), *Iodes perrieri* (Ipe) and *Iodes liberica* (Ili). The amino acid numbers for each sequence are indicated on the right. In the sequence alignment, identical residues are shown with a black background, and similar residues are shown with a gray background.

* 20 * 40 * 60 * 80 * 100 * 120
Ise : MEVLMAERANLVFHNKMVDGTAMKGLISRLIDHFGMAYTSHILDQVKTLGFQQATLTSISLGIDDLLTIPSKGWLVQDSEQQSLILEKHHHYGNVHAVEKLRQSIEIWYATSEYLRQEMNPNFSMTDPF : 129
Ili : MEVLMAERANLVFYNKMVDGTAMKRLISRLIDHFGMAYTSHILDQVKTLGFQQATLTSISLGIDDLLTIPSKGWLVQDSEQQSLILEKHHHYGNVHAVEKLRQSIEIWYATSEYLRQEMNPNFSMTDPF : 129
Ipe : MEVLMAERANLVFHNKMVDGTAMKRLISRLIDHFGMAYTSHILDQVKTLGFQQATLTSISLGIDDLLTIPSKGWLVQDSEQQSLILEKHHHYGHVHAVEKLRQSIEIWYATSEYLRQEMNPNFSMTDPF : 129
Ikl : MEVLMAERANLVFYNKMVDGTAMKRLISRLIDHFGMAYTSHILDQVKTLGFQQATLTSISLGIDDLLTIPSKGWLVQDSEQQSLILEKHHHYGNVHAVEKLRQSIEIWYATSEYLRQEMNPNFSMTDPF : 129
Ici : MEVLMAERANLVFHNKMVDGTAMKRLISRLIDHFGMAYTSHILDQVKTLGFQQATLTSISLGIDDLLTIPSKGWLVQDSEQQSLILEKHHHYGNVHAVEKLRQSIEIWYATSEYLRQEMNPNFSMTDPF : 129
Isc : --------------------------------------------------------------------------------------------------------------------------------- : -
Ath : ----MAERANLVFHNKVIDGTAIKRLISRLIDHFGMAYTSHILDQVKTLGFQQATATSISLGIDDLLTIPSKGWLVQDAEQQSWILEKHHHYGNVHAVEKLRQSIEIWYATSEYLRQEMNPNFRMTDPF : 125

 * 140 * 160 * 180 * 200 * 220 * 240 * 2
Ise : NPVHIMSFSGARGNASQVHQLVGMRGLMSDPQGQMIDLPIQSNLREGLSLTEYIISCYGARKGVVDTAVRTSDAGYLTRRLVEITRRLVEVVQHKKVRRTDCGTARSISVSPRN--GMMPERIFIQTLI : 256
Ili : NPVHIMSFSGARGNASQVHQLVGMRGLMSDPQGQMIDLPIQSNLREGLSLTEYIISCYGARKGVVDTAVRTSDAGYLTR-------RLVEVVQHIVVRRTDCGTARSISVSPRN--GMVPERIFIQTLI : 249
Ipe : NPVHIMSFSGARGNASQVHQLVGMRGLMSDPQGQMIDLPIQSNLREGLSLTEYIISCYGARKGVVDTAVRTSDAGYLTR-------RLVEVVQHIVVRRTDCGTARSISVSPRN--GMMPERIFIQTLI : 249
Ikl : NPVHIMSFSGARGNASQVHQLVGMRGLMSDPQGQMIDLPIQSNLREGLSLTEYIISCYGARKGVVDTAVRTSDAGYLTR-------RLVEVVQHIVVRRTDCGTARSISVSPRN--GMMPERIFIQTLI : 249
Ici : NPVHIMSFSGARGNASQVHQLVGMRGLMSDPQGQMIDLPIQSNLREGLSLTEYIISCYGARKGVVDTAVRTSDAGYLTR-------RLVEVVQHIVVRRTDCGTARSISVSPRN--GMMPERIFIQTLI : 249
Isc : ---------------SQVHQLVGMRGLMSDPQGQMIDLPIQSNLREGLSLTEYIISCYGARKGVVDTAVRTSDAGYLTR-------RLVEVVQHIVVRRTDCGTARSFSVSPRN--GMMSERIFIQTLI : 105
Ath : NPVHMMSFSGARGNASQVHQLVGMRGLMSDPQGQMIDLPIQSNLREGLSLTEYIISCYGARKGVVDTAVRTSDAGYLTR-------RLVEVVQHIVVRRTDCGTIRGISVSPRNKNRMMSERIFIQTLI : 247

 60 * 280 * 300 * 320 * 340 * 360 * 380
Ise : GRVLADDIYMGPRCIATRNQDIGIKLVNRFITFRAQPISIRTPFTCRSTSWICRLCYGRSPAHGDLVELGEAVGIIAGQSIGEPGTQLTLRTFHTGGVFTGGTAEHVRAPSNGK---IKFNENLVHPTR : 382
Ili : GRVLADDIYMGSRCIATRNQDIGIKLVNRFITFRAQPISIRTPFTCRSTSWICRLCYGRSPAHGDLVELGEAVGIIAGQSIGEPGTQLTLRTFHTGGVFTGGTAEHVRAPSNGNRKFNKFNENLVHPTR : 378
Ipe : GRVLADDIYMGPRCIATRNQDIGIKLVNRFITFRAQPISIRTPFTCRSTSWICRLCYGRSPAHGDLVELGEAVGIIAGQSIGEPGTQLTLRTFHTGGVFTGGTAEHVRAPSNGK---IKFNENLVHPTR : 375
Ikl : GRVLADDIYMGPRCIATRNQDIGIKLVNRFITFRAQPISIRTPFTCRSTSWICRLCYGRSPAHGDLVELGEAVGIIAGQSIGEPGTQLTLRTFHTGGVFTGGTAEHVRAPSNGK---IKFNENLVHPTR : 375
Ici : GRVLADDIYMGPRCIATRNQDIGIKLVNRFITFRAQPISIRTPFTCRSTSWICRLCYGRSPAHGDLVELGEAVGIIAGQSIGEPGTQLTLRTFHTGGVFTGGTAEHVRAPSNGK---IKFNENLVHPTR : 375
Isc : GRVLADDIYMGPRCIATRNQDIGIKLVNRFITLRAQPISIRTPFTCRSTSWICRLCYGRSPAHGDLVELGEAVGIIAGQSIGEPGTQLTLRTFHTGGVFTGGTAEHVRAPSNGK---IKFNENLVHPTR : 231
Ath : GRVLADDIYIGSRCVAFRNQDLGIGLVNRLITFGTQSISIRTPFTCRSTSWICRLCYGRSPTHGDLVELGEAVGIIAGQSIGEPGTQLTLRTFHTGGVFTGGTAEHVRAPYNGK---IKFNEDLVHPTR : 373

* 400 * 420 * 440 * 460 * 480 * 500 *
Ise : IRHGHPAFLCSIDLYVTIESEDIIHNVYIPPKSFLLVQNDQYVESEQVIAEIRARTFTFNFKEKVLKHIYSDSEG----------------------EMHWSTDVYHTPEFTYGNVHLLPKPSHLWILL : 489
Ili : TRHGHPAFLCSIDLYVTIESEDIIHNVYIPPKSFLLVQNDQYVESEQVIAEIRARAFTFNFKEKVLKHIYSDSEG----------------------EMHWSTDVYHTPEFTYGNVDLLPKPSHLWILL : 485
Ipe : TRHGHPAFLCSIDLYVTIESEDIIHNVYIPPKSFLLVQNDQYVESEQVIAEIRARTFTFNFKEKVLKHIYSDSEG----------------------EMHWSTDVYHTPEFTYGNVHLLPKPSHLWILL : 482
Ikl : TRHGHPAFLCSIDLYVTIESEDIIHNVYIPPKSFLLVQNDQYVESEQVIAEIRARAFTFNFKEKVLKHIYSDSEG----------------------EMHWSTDVYHTPEFTYGNVHLLPKPSHLWILL : 482
Ici : TRHGHPAFLCSIDLYVTIESEDIIHNVYIPSKSFLLVQNDQYVESEQVIAEIRARTFIFNFKEKVRKHIYSDSEG----------------------EMHWSTDVYHTPEFTYGNVHLLPKPSHLWILL : 482
Isc : TRHGHPAFLCSIDLYVTIESEDIIHNVYIPPKSFLLVQNDQYVESEQVIAEIRARAFIFNFKEKVRKHIYSDSEGEMHGTPNNIHKVRKHIYSDSEGEMHWSTDVYHTPEFIYGNVHLLPKPSHLWILL : 360
Ath : TRHGHPAFLCYIDLSVIIESEDIIHSVTIPPKSFLLVQNDQYVESEQVIAEIREGTYTFHFKERVRKYIYSDSEG----------------------EMHWSTDVSHAPEFTYSNVHLLPKTSHLWILS : 480

 520 * 540 * 560 * 580 * 600 * 620 * 640
Ise : GVPCRSSLVSPLLHRDQDQMNAHSLSVKQGSISSPSVTNDQARHKFFSSNFSGKKEDRITTYSELNRILCTSRSNLIYSDILHENSDLLAKRRRNRFIIPLQSIHERENELMPRSGISIEIPINGTFSR : 618
Ili : GVPCRSSLVSPLLHKDQDQMNTHSLSVKQGSISSPSVTNDQARHKFFSSNVSGKKEDRITNYSELNRILCTSRSNLIYSDILHENSDLLAKRRRNRFIIPLQSIHERENELMPRSGISIEIPRNG---- : 610
Ipe : GVPCRSSLVSPLLHKDQDQMNAHSLSVKQGSISSPSVTNDQARHKFFSSNFSGKKEDRITNYSELNRILCTSRSNLIYSDILHENSDLLAKRRRNRFIIPLQSIHERENELMPRSGISIEIPINGTFSR : 611
Ikl : GVPCRSSLVSPLLHKDQDQINAHSLSVKQGSISSPSVTNDQARHKFFSSNVSGKKEDRSTNYSELNRILCTSRSNLIYSDILHENSDLLAKRRRNRFIIPLQSIHERENELMPRSGISIEIPRNGTFSR : 611
Ici : GVSCRSSLVSPLLHKDQDQMNAHSLSVKQGSISSPSVTNDQARHKFFSSNFSGKKEDRIPNYSELNRILCTSRSNLIYSYTLHENSDLLAKRRRNRFIIPLQSIRERENELMPRSGISIEIPINGTFSR : 611
Isc : GVPCIS--------------------------------------------------------------------------------------------------------------------------- : 366
Ath : GGSCGSSLIRFSIHKDQDQMNIPFLSAERKSISSLSVNNDQVSQKFFSSDFADPKKLGIYDYSELNGNLGTSHYNLIYSAIFHENSDLLAKRRRNRFLIPFQSIQEQEKEFIPQSGISVEIPINGIFRR : 609

 * 660 * 680 * 700 * 720 * 740 * 760 *
Ise : NSILAYFDEPRYRRTSSGITKYGTLEVHSIVKKEDLVEYQGVKEFRPKYQNQTKVDRFFFIPEEVHILPESSSIMVRNNSIIGVDTQITLNTRSRVGGLVRVERKKKKNGTSILEIFIFPEKQIRYPGE : 747
Ili : ---------PRYRRTSSGITKYGTLEVHSIVKKEDLVEYQGVKEFRPKYQNQTKVDQFFFIPEEVHILPESSSIMVRNNSIIGVDTQITLNTRSRVGGLVRVERKKKR-----IELKIFSG-DIHFPGE : 724
Ipe : NSILAYFDEPRYRRTSSGITKYGTLEVHSIVKKEDLVEYQGVKEFRPKYQNQTKVDLFFFIPEEVHILSESSSIMVRNNSIIGVDTQITLNTRSRVGGLVRVERKKKR-----IELKIFSG-------- : 727
Ikl : NGILAYFDEPRYRRTSSGITKYGTLEVHSIVKKEDLVEYQGVKEFRPKYQNQTKVDRFFFIPEEVHILPESSSIMVRNNSIIGVDTQITLNTRSRVGGLVRVERKKKR-----IELKIFSG-------- : 727
Ici : NSILAYFDEPRYRRTSSGITKYGTLEVHSIVKKEDLVEYQGVKEFRPKYQNQTKVDRFFFIPEEVHILPESSSIMVRNNSIIGVDTQITLNTRSRVGGLVRVERKKKR-----IELKIFSG-------- : 727
Isc : ----------------------GTLEVHSIVKKEDLVEYQGVKEFRPKYQNQTKVDRFFFIPEEVHILSESSSIMVRNNSIIGVDTQITLNRRSRVGGLVRVERKKKR-----IELKIFSG-------- : 460
Ath : NSIFAFFDDPRYRRKSSGILKYGTLKADSIIQKEDMIEYRGVQKIKTKYE--MKVDRFFFIPEEVHILPESSAIMVQNYSIIGVDTRLTLNIRSQVGGLIRVEKKKKR-----IELKIFSG-------- : 723

780 * 800 * 820 * 840 * 860 * 880 * 900
Ise : KKKKEWNFNSGDIHFPGETDKISRHSGVLIPPGMGKANSKESKKFQNWIYVQRITPSKKKYFVLVRPVVAYEITDGINLSTLFPPDLLQERDNVKLRVVNYILYGNGKPIRGISDTSIQLVRTCLVLNW : 876
Ili : TDK--------DIHFPGETDKISRHSGVLIPPGMGKANSKESKKFKNWIYVQRITPSKKKYFVLVRPVVAYEITDGINLSTLFPPDLLQERDNVKLRVVNYILYGNGKPIRGISDTSIQLVRTCLVLNW : 845
Ipe : -----------DIHFPGETDKISRHSGVLIPPGMGKANSKESKKLKNWIYVQRITPSKKKYFVLVRPVVAYEITDGINLLTLFPPDLLQERDNVKLRVVNYILYGNGKPIRGISDTSIQLVRTCLVLNW : 845
Ikl : -----------DIHFPGETDKISRHSGVLIPPGMGKANSKESKKFKNWIYVQRITPSKKKYFVLVRPVVAYEITDGINLSTLFPPDLLQERDNVKLRVVNYILYGNGKPIRGISDTSIQLVRTCLVLNW : 845
Ici : -----------DIHFPGETDKISRHSGVLIPPGMGKANSKESKKFKNWIYVQRITPSKKKYFVLVRPVVAYEITDGINLSTLFPPDLLQEGDNVQLRIVNYILYGNGKPIRGISDTSIQLVRTCLVLNW : 845
Isc : -----------DIHFPGETDKISRHSGVLIPPGMGKANSKESKKFKNWIYVQRITPRKKKYFLLVRPVVAYEITDGINLSTLFPPDLLQEGDNVQLRIVNYILYGNGKPIRGISDTSIQLVRTCLVLNW : 578
Ath : -----------DIHFPDKTDKISRHSGILIPPGRGKKNSKESKKFKNWIYVQRITPTKKKFFVLVRPVATYEIADSINLATLFPQDLFREKDNIQLRVFNYILYGNGKPTRGISDTSIQLVRTCLVLNW : 841

 * 920 * 940 * 960 * 980 * 1000 * 1020 *
Ise : DQDTKNSSNEEARASFVEIKANGLIRDFLRVDLVKSPISYSRKRKDPSSSGLISDNGSDRANTNPFSYISKTG--IRQYFNQNKGTIHTLVNRNKQCQSLIILSSSNCFRMGPFKDVK--YHNGIKESI : 1001
Ili : DQDTKNSSNDEARASFVEIKINGLIRDFLRVDLVKSPISYSRKRKDPSSSGLISDNGSDRANTNPFSYIS-QG--IRQYFNQNQGTIHTLVNRNKECQS-LILSSFNCFRMGPFDNVK--YHNGIKELI : 968
Ipe : DQDTKNSSNEGARASLVEIKTNGLIQDFLRIDVVKSPISYSRKRKDPSSSGLISDNGSDRANTNPFSYISKTG--IRQYFNQNQGTIHTLVNRNKECQSLIILSSSNCFRMGPFNDVK--YHNGIKESI : 970
Ikl : DQDTKNSSNEEARASFVEIKINGLIRDFLRVDLVKSPISYSRKRKDPSSSGLISDNGSDRANTNPFSYISKTG--IRQYFNQNQGTIHTLVNRNKECQSLIILSSSNCFRMGPFNDVKYHYHNGIKESI : 972
Ici : DQDTKNSSSEEARASFVEIKTNGLIRDFLRVDLVKSPISYSRKRKDPSSLGLISDNGLDRANTNPFSYISKTG--IRQYFNQNQGTIHTLLNRNKECQSLIILTSSNCFRMGPFNDVK--YHNGIKESI : 970
Isc : DQDTKNSSSEEARASFVEIKTNGLIRDFLRVDLVKSPISYSRKRKDPSSLGLISDNGLDRANTNPFFYISKTG--IRQYFNQNQGTIHTLVNRNKECQS---------------------EYQGVKEFR : 684
Ath : D---KNSSLEEVRAFFVEVSTKGLIQDFIRIGLVKSHISYIRKRNNSPDSGLISAD-----HMNPFYSISPKSGILQQSLRQNHGTIRMFLNRNKESQSLLILSSSNCFRMGPFNHVK--HHNVINQSI : 960

 1040 * 1060 * 1080 * 1100 * 1120 * 1140 * 1160
Ise : KKDPLTPIRNSLGPLGTALPIANFYSFYHLITHNQVLATNYLQLNNLKQTFQVLNYYLMDENGKIYNPDLFNNIILNPFNLNWYFLHH----NYCEETSTIISLGQFICENVCIAKNGPHLKSGQVLIV : 1126
Ili : KKDPPMPIRNLLGPLGTALPIANFYSFYHLITHNQVLATNYLQLNNLKQTFQVD------ENGKT-NPDLFNNIILNPFNLNWYFLHH----NYCEETSTIISLGQFICENVCIAKNGPHLKSGQVLIV : 1086
Ipe : KKYPPMPIRNSLGPLGTALPIANFYSFYHLITYNQVLATNYLQLNNLKQTFQVLNYYLMDENGKIYNPDLF---ILNPFNLNWYFLHH----NYCEETSTIISLGQFICENVCIAKNGPHLKSGQVLIV : 1092
Ikl : KKDPPMPIRNLLGPLGTALPIANFYSFYHLITHNQVLATNYLQLNNLKQTFQVLN--------------------------YYLMLHH----NYCEETSTIISLGQFICENVCIAKNGPHLKSGQVLIV : 1071
Ici : KKDPLMPIRNSLGPLGTALPIANFYSFYHLITHNQVLVTNYLQLNNLKQTFQVLNYYLMDENGKIYNPDLFNNIILNPFNLNWYFLHH----NYCEETSTIISLGQFICENVCIAKNGPHLKSGQALIV : 1095
Isc : PKY-----------------------------QNQTKVD------------------------------------------------------------------------LIVAKNGPHLKSGQVLIV : 712
Ath : KKNTLITIKNSSGPLGTATPISNFYSFLPLLTYNQISLIKYFQLDNLKYIFQKINSYLIDENGIILNLDPYSNVVLNPFKLNWYFLHQNYHHNYCEETSTIISLGQFFCENVCIAKKEPHLKSGQVLIV : 1089

* 1180 * 1200 * 1220 * 1240 * 1260 * 1280 *
Ise : QVNSVVIRSARPYLATPGATVHGHYGKILYEGDTLVTFIYEKSRSGDITQGLPKVEQVLEVRSVDSISMNLENRIEGWNECIPRILGIPWGFLIGAELTIAQSRISLVNKIQKVYRSQGVQIHNRHIEI : 1255
Ili : QVNSVVIRSARPYLATPGATVHGHYGKILYEGDTLVTFIYEKSGSGDITQGLPKVEQVLEL-SVDSISMNLENRIEGWNECIPRILGIKN-----------------------FYDTS----------- : 1180
Ipe : QINSVVIRSARPYLATPGATVHGHYGKILYEGDTLVTFLYEKSRSGDITQGLPKVEQVLEVRSVDSISMNLENRIEGWNECIPRILGIPWGFLIGAELTIAQSRISLVNKIQKVYRSQGVQIHNRHIEI : 1221
Ikl : QVNSVVIRSARPYLATPGATVHGHYGKILYEGDTLVTFIYEKSRSGDITQGLPKVEQILEVRSVDSISMNLENRIEGWNECIPRILGIPWGFLIGAELTIAQSRISLVNKIQKVYRSQGVQIHNRHIEI : 1200
Ici : QVNSVVIRSARSYLATPGASVHGHYGKILYEGDTLVTFIYEKSRSGDITQGLPKVEQVLEVRSVDSISMNLENRIEGWNECIPRILGIPWGFLIGAELTIAQSRISLVNKIQKVYRSQGVQIHNRHIEI : 1224
Isc : QVNSVVIRSARSYLATPGATVHGHYGKILYEGDTLVTFIYEKSRSGDITQGLPKVEQVLEVRSVDSISMNLENRIEGWNECIPRILGIPWGFLIGAELTIAQSRISLVNKIQKVYRSQGVQIHNRHIEI : 841
Ath : QRDSAVIRSAKPYLATPGAKVHGHYSEILYEGDTLVTFIYEKSRSGDITQGLPKVEQVLEVRSIDSISLNLEKRIKGWNKCITRILGIPWGFLIGAELTIVQSRISLVNKIQKVYRSQGVQIHNRHIEI : 1218

 1300 * 1320 * 1340 * 1360 * 1380 * 1400 * 142
Ise : IVRQITSKVLVSEDGMSNVFLPGELIGLLRAERMGRALEEAICYRAILLGITKASLNTQSFISEASFQETARVLAKAALRGRIDWLKGLKENVVLGGMIPVGTGF-KGLVHTSRHYNNIPFEIKKK--N : 1381
Ili : ---------------------------------------------------------EQSFIG---FNDS----------------------------------------------------------- : 1190
Ipe : IVRQITSKVLVSEDGMSNVFLPGELIGLLRAERMGRALEEAICYRAILLGITKASLNTQSFISEASFQETARVLAKAALRGRIDWLKGLKENVVLGGMIPVGTGF-KGLVHTSRHYNNLPFEIKKK--N : 1347
Ikl : IVRQITSKVLVSEDGISNVFLPGELIGLLRAERMGRALEEAICYRAILLGITKASLNTQSFISEASFQETARVLAKAAVRGRIDWLKGLKENVVLGGMIPVGTGF-KGLVHTSRHYNNIPFEIKKK--N : 1326
Ici : IVRQITSKVLVSEDGMSNVFLPGELIGLLRAERMGRALEEAICYRAILLRITRASLNTQSFIFEASFQETARVLAKVALQGCIYWLKGLKENVVLRGMIPVGTGF-KGLVHTSRHYNNILFDFKKK--N : 1350
Isc : IVRQITSKVLVSEDGMSNVFLPGELIGLLRAERMGRALEEAICYRAILLGITRASLNTQSFISEASFQETTRVLAKAALRGRIDWLKGLKENVVLGGMIPVGTGF-KGLVHTSRHYNNIPFEIKKK--N : 967
Ath : IVRQITSKVLVSEEGMSNVFLPGELIGLLRAERTGRALEEAICYRAVLLGITRASLNTQSFISEASFQETARVLAKAALRGRIDWLKGLKENVVLGGVIPAGTGFNKGLVHCSRQHTNIILEKKTKNLA : 1347

 0 * 1440 *
Ise : --------------------------------------- : -
Ili : --------------------------------------- : -
Ipe : LFEGEIGDILFHHRKLFDSCLSKNFYDTSEQSFIGFNDS : 1386
Ikl : LFEGEIGDILFHHRKLFDSCLSKNFYDTSEQSFIGFNDS : 1365
Ici : LFEGEIGDILFHHRKLFDSCLSKNFYDTSEQSFIRFNDS : 1389
Isc : LFEGEIGDILFHHRKLFDSCLSKNFYDTSEQSFIGFNDS : 1006
Ath : LFEGDMRDILFYHREFCDSSISKSDFSRI---------- : 1376

**Figure S59 Multiple sequence alignment of the deduced amino acid sequences of RPOC2 protein.** The origins of the protein sequences used in this alignment are from: *Arabidopsis thaliana* (Ath), *Iodes klaineana* (Ikl), *Iodes cirrhosa* (Ici), *Iodes seretii* (Ise), *Iodes scandens* (Isc), *Iodes perrieri* (Ipe) and *Iodes liberica* (Ili). The amino acid numbers for each sequence are indicated on the right. In the sequence alignment, identical residues are shown with a black background, and similar residues are shown with a gray background.

* 20 * 40 * 60 * 80 * 100 * 120 *
Ikl : MTRRYWNINLEEMMEAGVHFGHGTRKWNPKMEPYISAKRKGIHITNLTRTARFLSEACDLVFDAASRGKQFLIVGTKNKAADSVAWAAIRARCHYVNKKWLGGMLTNWSTTETRLSKFRDLRTEQKT----- : 127
Ise : MTRRYWNINLEEMMEAGVLFGHGTRKWNPKMEPYISAKRKGIHITNLTRTARFLSEACDLVFDAASRGKQFLIVGTKNKAADSVAWAAIRARCHYVNKKWLGGMLTNWSTTETRLSKFRDLRTEQKKGLENG : 132
Isc : MTRRYWNINLEEMMEAGVHFGHGTRKWNPKMAPYISAKRKGIHITNLTRTARFLSEGCDLVFDAASRGKQFLIVGTKNKAADSVAWAAIRARCHYVNKKWLGGMLTNWSTTETRLSKFRDLRTEQKT----- : 127
Ipe : MTRRYWNINLEEMMEAGVHFGHGTRKWNPKMEPYISAKRKGIHITNLTRTARFLSEACDLVFDAASRGKQFLIVGTKKKAADSVAWAAIRARCHYVNKKWLGGMLTNWSTTETRLSKFRDLRTEQKT----- : 127
Ili : MTRRYWNINLEEMMEAGVHFGHGTRKWNPKMEPYISAKRKGIHITNLTRTARFLSEACDLDFGVASKGK--LIVGTKKKAADSVAWAAIRARCHYVNKKWLGGMLTNWSTTETRLSKFRDLRTEQKT----- : 125
Ici : MTRRYWNINLEEMMEAGVHFGHGTRKWNPKMAPYISAKRKGIHITNLTRTARFLSEACDLVFDAASRGKQFLIVGTKNKAADSVAWAAIRARCHYVNKKWLGGMLTNWSTTETRLSKFRDLRTEQKT----- : 127
Ath : MTKRYWNIDLEEMMRAGVHFGHGTRKWNPRMAPYISAKRKGIHIINLTRTARFLSEACDLVFDAASRGKQFLIVGTKNKAADLVSRAAIRARCHYVNKKWLGGMLTNWSTTEKRLHKFRDLRTEQKT----- : 127

 140 * 160 * 180 * 200 * 220 * 240
Ikl : ---GRLNRLPKRDAAMLKRQLSRLQTYLGGIKYMTGLPDIVIIVDQHEEYTALRECITLGIPTICLIDTNCDPDLADISIPANDDAISSIRLILNKLVFAISEG-------- : 228
Ise : TKKGRLNRLPKRDAAMLKRQLSRLQTYLGGIKYMTGLPDIVIIVDQHEEYTALRECITLGIPTICLIDTNCDPDLADISIPANDDAISSIRLILNKLVFAISEGR------- : 237
Isc : ---GRLNRLPKRDAAMLKRQLSRLQTYLGGIKYMTGLPDIVIIVDQHEEYTALRECITLGIPTICLI--------------------------------------------- : 191
Ipe : ---GRLNRLPKRDAAMLKRQLSRLQTYLGGIKYMTGLPDIVIIVDQHEEYMALRECITLGIPTICLIDTNCDPDLADISIPANDDAISSIRLILNKLVFAISEGRSSYIRNP : 236
Ili : ---GRLNRLPKRDAAMLKRQLSRLQTYLGGIKYMTGLPDIVIIVDQHEEYTALRECITLGIPTICLIDTNCDPDLADISIPANDDAISSIRLILNKLVFAISEGRSSYIRNP : 234
Ici : ---GRLNRLPKRDAAILKRQLSRLQTYLGGIKYMTGLPDIVIIVDQHEEYTALRECITLGIPTICLIDTNCDPDLADISIPANDDAISSIRLILNKLVFAISEGRSSYIRNP : 236
Ath : ---EGFNRLPKRDAAVLKRQLSRLETYLGGIKYMTGLPDIVIILDQQEEYTALRECITLGIPTISLIDTNCNPDLADISIPANDDAIASIRFILNKLVFAICEGRSSYIQNS : 236

**Figure S60 Multiple sequence alignment of the deduced amino acid sequences of RPS2 protein.** The origins of the protein sequences used in this alignment are from: *Arabidopsis thaliana* (Ath), *Iodes klaineana* (Ikl), *Iodes cirrhosa* (Ici), *Iodes seretii* (Ise), *Iodes scandens* (Isc), *Iodes perrieri* (Ipe) and *Iodes liberica* (Ili). The amino acid numbers for each sequence are indicated on the right. In the sequence alignment, identical residues are shown with a black background, and similar residues are shown with a gray background.

* 20 * 40 * 60 * 80 * 100 * 120 *
Isc : --------------------------------------------------------------------------------------------------NGQKELNCVNRKLNISVARISKPYRNP------- : 27
Ici : MGQKINPLGFRLGTTQDHHSLWFAQPKNYSEGLQEDQKIRDYIKDYVQKNMRISSGVEGIARIEIQKRIDLIQVIIFMGFPTKLLIESRPLGIEELQKNLQKELNCVNRKLNISVARISKPYRNP------- : 125
Ikl : MGQKINPLGFRLGTTQDHHSLWFAQPKNYSEGLQEDQKIRDYIKDYVQKNMRISSGVEGIARIEIQKRIDLIQVIIFMGFPTKLLIESRPLGIEELQKNLQKELNCVNRKLNISVARISKPYGNP------- : 125
Ise : MGQKINPLGFRLGTTQDHHSLWFAQPKNYSEGIKEDQKIRDYIKDYVQKNMRISSGVEGIARIEIQKRIDLIQVIIFMGFPTKLLESRPLG-IEELQKNLQKELNCVNRKLNISVARISKPYGNP------- : 124
Ipe : MGQKINPLGFRLGTTQDHHSLWFAQPKNYSEGLQEDQKIRDSIKDYVQKNMRISSGVEGIARIEIQKRIDLIQVIIFMGFPTKLLIESRPLGIEELQKNLQKELNCVNRKLNISVARISKPYGNP------- : 125
Ili : --------------------------------------------------------------------------------------------------MGQK-RNPLGFRLGTTQDHHSLWFAQP------- : 26
Ath : --------------------------------------------------------------------------------------------------MGQK-INPLGFRLGTTQSHHSLWFAQPKKYSEGL : 33

 140 * 160 * 180 * 200 * 220 * 240 * 260
Isc : -------------------------------------------------------------------------------------------TILS---AGQLKNRVSFRKAMKKAIELTEQADTKGIQIQIA : 65
Ici : -------------------------------------------------------------------------------------------TILSEFIAGQLKNRVSFRKAMKKAIELTEQADTKGIQIQIA : 166
Ikl : -------------------------------------------------------------------------------------------TILSEFIAGQLKNRVSFRKAMKKAIELTEQADTKGIQIQIA : 166
Ise : -------------------------------------------------------------------------------------------TILSEFIAGQLKNRVSFRKAMKKAIELTEQADTKGIKIQIA : 165
Ipe : -------------------------------------------------------------------------------------------TILSEFIAGQLKNRVSFRKAMKKAIELTEQADTKGIQIQIA : 166
Ili : ------------------------------------------------------------------------------------------------------------------------------------ : -
Ath : EEDKKIRDCIKNYVQKNIRISSGMEGIARIEIQKRIDLIQIIIYMGFPKLLIEDKPRRVEELQMNVQKELNCVNRKLNIAITRISNPYGDPNILAEFIAGQLKNRVSFRKAMKKAIELTEQANTKGIQVQIA : 165

 * 280 * 300 *
Isc : GRIDGK----------------------------------------------- : 71
Ici : GRIDGKEIARVEWIREGRVPLQTIRAKIDYCFYTVRTIYGVLGIKIWIFIDEE : 219
Ikl : GRIDGKEIARVEWIREGRVPLQTIRAKIDYSFYTVRTIYGVLGIKIWIFIDEE : 219
Ise : GRIDGKEIARVEWIREGRVPLQTIRAKIDYCFYTVRTIYGVLGIKIWIFIDEE : 218
Ipe : GRIDGKEIARVEWIREGRVPLQTIRAKIDYCCYTVRTIYGVLGIKIWIFIDKE : 219
Ili : ----------------------------------------------------- : -
Ath : GRIDGKEIARVEWIREGRVPLQTIEAKIDYCSYTVRTIYGVLGIKIWIFVDEE : 218

**Figure S61 Multiple sequence alignment of the deduced amino acid sequences of RPS3 protein.** The origins of the protein sequences used in this alignment are from: *Arabidopsis thaliana* (Ath), *Iodes klaineana* (Ikl), *Iodes cirrhosa* (Ici), *Iodes seretii* (Ise), *Iodes scandens* (Isc), *Iodes perrieri* (Ipe) and *Iodes liberica* (Ili). The amino acid numbers for each sequence are indicated on the right. In the sequence alignment, identical residues are shown with a black background, and similar residues are shown with a gray background.

* 20 * 40 * 60 * 80 * 100 * 120 *
Isc : ------------------------------------------------------------------------------TGQVLLQLLEMRLDNILFRLGMASTIPGARQLVNHRHILVNGGIVDIPSYRCKP : 54
Ici : MSRYRGPRFKKIRRLGDLPGLTNKRSRAGSDLRNQSRSGKKSQYRIRLEEKQKLRFHYGLTERQLLKYVRIARKAKGSTGQVLLQLLEMRLDNILFRLGMASTIPGARQLVNHRHILVNGRIVDIPSYRCKP : 132
Ise : MSRYRGPRFKKIRRLGALPGLTNKRSRVVSDLKNQSRSGKKSQYRIRLEEKQKLRFHYGLTERQLLKYVRIARKAKGSTGQVLLQLLEMRLDNILFRLGMASTIPGARQLVNHRHILVNGRIVDIPSYRCKP : 132
Ipe : MSRYRGPRFKRIRRLGALPGLTNKRSRVGSDLRNQSRSGKKSQYRIRLEEKQKLRFHYGLTERQLLKYVRIARKAKGSTGEVLLQLLEMRLDNILFRLGMASTIPGARQLVNHRHILINGRIVDIPSYRCKP : 132
Ikl : MSRYRGPRFKRIRRLGALPGLTNKRSRAGSDLRNQSRSGKKSQYRIRLEEKQKLRFHYGLTERQLLKYVRIARKAKGSTGQVLLQLLEMRLDNILFRLGMASTIPGARQLVNHRHILVNGRIVDIPSYRCKP : 132
Ili : MSRYRGPRFKRIRRLGALPGLTNKRSRAGSDLRNQSRSGKKSQYRIRLEEKQKLRFHYGLTERQLLKYVRIARKAKGSTGQVLLQLLEMRLDNILFRLGMASTIPGARQLVNHRHILVNGRIVDIP------ : 126
Ath : MSRYRGPRFKKIRRLGALPGLTSKRPKAGSDLRNQSRSVKKSQYRIRLEEKQKLRFHYGLTEHQLLKYVRIAGKAKGSTGQVLLQLLEMRLDNILFRLGMALTIPQARQLVNHGHILVNGRIVDIPSYRCKP : 132

 140 * 160 * 180 * 200
Isc : RDIITARDEQKSRVLIQNSLDSYL--------------------------------------------- : 78
Ici : RDIITARDEQKSRVLIQNSLDSYLYEELPKHLTLHPFQYKGLVNQIIDSKWVGLKINELLVVEYYSRQT : 201
Ise : RDIVTVKDEQKSRVLIQNSLESYLHEELPKHLTLHPFQYKGLVNQIIDSKWVGLKINELLVVEYYSR-- : 199
Ipe : RDIITVKDEQKSRVLIQNSLDSYLHEELPKHLTLHPFQYKGLVNQIIDSKWVGLKINELLVVEYYSRQT : 201
Ikl : RDIITVKDEQKSRVLIQNSLDSYLHEELPKHLTLHPFQYKGLVNQIIDSKWVGLKINELLVVEYYSRQT : 201
Ili : --------------------------------------------------------------------- : -
Ath : RDIITVKDEQNSRTLVQNLLDSSAPEELPNHLTLHTFQYEGLVNQIIDRKCVGLKINELLVVEYYSRQT : 201

**Figure S62 Multiple sequence alignment of the deduced amino acid sequences of RPS4 protein.** The origins of the protein sequences used in this alignment are from: *Arabidopsis thaliana* (Ath), *Iodes klaineana* (Ikl), *Iodes cirrhosa* (Ici), *Iodes seretii* (Ise), *Iodes scandens* (Isc), *Iodes perrieri* (Ipe) and *Iodes liberica* (Ili). The amino acid numbers for each sequence are indicated on the right. In the sequence alignment, identical residues are shown with a black background, and similar residues are shown with a gray background.

* 20 * 40 * 60 * 80 * 100 * 120 *
Ili : MSRRGTAEKKTTKSDPIYRNRLVNMLVNRILKHGKKSLAYQIIYRAVKKIQQKTETNPLSVLRQAIRGGTPDIAVKARRVGGSTHQVPIEIGSTQGKALAIRWLLAASRKRPGRNMAFKLSSELVDAAKGSG : 132
Ath : MSRRGTAEEKTAKSDPIYRNRLVNMLVNRILKHGKKSLAYQIIYRALKKIQQKTETNPLSVLRQAIRGVTPDIAVKARRVGGSTHQVPIEIGSTQGKALAIRWLLGASRKRPGRNMAFKLSSELVDAAKGSG : 132
Ise : MSRRGTAEKKTTKSDPIYRNRLVNMLVNRILKHGKKSLAYQIIYRAVKKIQQKTETNPLSVLRQAIRGGTPDIAVKARRVGGSTHQVPIEIGSTQGKALAIRWLLAASRKRPGRNMAFKLSSELVDAAKGSG : 132
Ici : MSRRGTAEKKTTKSDPIYRNRLVNMLVNRILKHGKKSLAYQIIYRAVKKIQQKTETNPLSVLRQAIRGGTPDIAVKARRVGGSTHQVPIEIGSTQGKALAIRWLLAASRKRPGRNMAFKLSSELVDAAKGSG : 132
Isc : MSRRGTAEKKTTKSDPIYRNRLVNMLVNRILKHGKKSLAYQIIYRAVKKIQQKTETNPLSVLRQAIRGGTPDIAVKARRVGGSTHQVPIEIGSTQGKALAIRWLLAASRKRPGRNMAFKLSSELVDAAKGSG : 132
Ikl : MSRRGTAEKKTTKSDPIYRNRLVNMLVNRILKHGKKSLAYQIIYRAVKKIQQKTETNPLSVLRQAIRGGTPDIAVKARRVGGSTHQVPIEIGSTQGKALAIRWLLAASRKRPGRNMAFKLSSELVDAAKGSG : 132
Ipe : MSRRGTAEKKTTKSDPIYRNRLVNMLVNRILKHGKKSLAYQIIYRAVKKIQQKTETNPLSVLRQAIRGGTPDIAVKARRVGGSTHQVPIEIGSTQGKALAIRWLLAASRKRPGRNMSTERRGLRISPIDYRQ : 132

 140 *
Ili : DAIRKKEETHRMAEANRAFAHFR : 155
Ath : DAIRKKEETHRMAEANRAFAHFR : 155
Ise : DAIRKKEETHRMAEANRAFAHFR : 155
Ici : DAIRKKEETHRMAEANRAFAHFR : 155
Isc : DAIRKKEETHRMAEANRAFAHFR : 155
Ikl : DAIRKKEETHRMAEANRAFAHFR : 155
Ipe : IAIRWLLAASRKRPG-RNM---- : 150

**Figure S63 Multiple sequence alignment of the deduced amino acid sequences of RPS7 protein.** The origins of the protein sequences used in this alignment are from: *Arabidopsis thaliana* (Ath), *Iodes klaineana* (Ikl), *Iodes cirrhosa* (Ici), *Iodes seretii* (Ise), *Iodes scandens* (Isc), *Iodes perrieri* (Ipe) and *Iodes liberica* (Ili). The amino acid numbers for each sequence are indicated on the right. In the sequence alignment, identical residues are shown with a black background, and similar residues are shown with a gray background.

* 20 * 40 * 60 * 80 * 100 * 120 *
Ikl : MAKAITRIGSRRNGRIGSRKNARRIPKGVIHVQASFNNTIVTVTDVRGRVVSWSSAGTCGFRGTRRGTPFAAQTTAVNAIRTVVDQGMQRAEVMIKGPGLGRDAALRAIRRSGILLTFIRDVTPMPHNGC-- : 130
Isc : MAKAITRIGSRRNGRIGSRKNARRIPKGVIH-------------------------------------------------RTVVDQGMQRAEVMIKGPGLGRDAALRAIRRSGILLTFIRDVTPMPHNGC-- : 81
Ili : MAKAITRIGSRRNGRIGSRKNARRIPKGVIHVQASFNNTIVTVTDVRGRVVSWSSAGTCGFRGTRRGTPFAAQTTAVNAIRTVVDQGMQRAEVMIKGPGLGRDAALRAIRRSGILLTFIRDVTPMPHNGC-- : 130
Ipe : MTKAITRIGSRRNGRIGSRKNARRIPKGVIHVQASFNNTIVTVTDVRGRVVSWSSAGTCGFRGTRRGTPFAAQTTAVNAIRTVVDQGMQRAEVMIKGPGLGRDAALRAIRRSGILLTFIRDVTPMPHNGC-- : 130
Ici : MAKAITRIGSRRNGRIGSRKNARRIPKGVIHVQASFNNTIVTVTDVRGRVVSWSSAGTCGFRGTRRGTPFAAQTTAVNAIRTVVDQGMQRAEVMIKGPGLGRDAALRAIRRSGILLTFIRDVTPMPHNGCRP : 132
Ise : MAKAITRIGSRRNGRIGSRKNVRRIPKGVIHVQASFNNTIVTVTDVRGRVISWSSAGTCGFRGTRRGTPFAAQTTAVNAIRTVVDQGMQRAEVMIKGPGLGRDAALRAIRRSGILLTFIRDVTPMPHNGCRP : 132
Ath : MAKPILRIGSRKNTRSGSRKNVRRIPKGVIHVQASFNNTIVTVTDVRGRVISWSSAGTCGFRGTRRGTPFAAQTAAGNAIRAVVDQGMQRAEVRIKGPGLGRDAALRAIRRSGILLSFVRDVTPMPHNGCRP : 132


Ikl : ------ : -
Isc : ------ : -
Ili : ------ : -
Ipe : ------ : -
Ici : PKKRRV : 138
Ise : PKKRRV : 138
Ath : PKKRRV : 138

**Figure S64 Multiple sequence alignment of the deduced amino acid sequences of RPS11 protein.** The origins of the protein sequences used in this alignment are from: *Arabidopsis thaliana* (Ath), *Iodes klaineana* (Ikl), *Iodes cirrhosa* (Ici), *Iodes seretii* (Ise), *Iodes scandens* (Isc), *Iodes perrieri* (Ipe) and *Iodes liberica* (Ili). The amino acid numbers for each sequence are indicated on the right. In the sequence alignment, identical residues are shown with a black background, and similar residues are shown with a gray background.

* 20 * 40 * 60 * 80 * 100 * 120 *
Ise : MPTIKQLIRNTRQPIRNVTKSPALRGCPQRRGTCTRVYVTITPKKPNSALRKVARVRLTSGFEITAYIPGIGHNSQEHSVVLVRGGRVKDLPGVRYHIVRGTLDAVGVKDRQQGRSSAL------------- : 119
Ici : MPTIKQLIRNTRQPIRNVTKSPALRGCPQRRGTCTRVYVTITPKKPNSALRKVARVRLTSGFEITAYIPGIGHNSQEHSVVLVRGGRVKDLPGVRYHIVRGTLDAVGVKDRQQGRSSAL------------- : 119
Ipe : MPTIKQLIRNTRQPIRNVTKSPALRGCPQRRGTCTRVYVTITPKKPNSALRKVARVRLTSGFEITAYIPGIGHNSQEHSVVLVRGGRVKDLPGVRYHIVRGTLDAVGVKDRQQGRSSAL------------- : 119
Ath : MPTIKQLIRNTRQPIRNVTKSPALRGCPQRRGTCTRVY-TITPKKPNSALRKVARVRLTSGFEITAYIPGIGHNLQEHSVVLVRGGRVKDLPGVRYHIVRGTLDAVGVKDRQQGRSKYGV----------KK : 121
Ikl : ---------------------------------------TITPKKPNSALRKVARVRLTSGFEITAYIPGIGHNSQEHSVVLVRGGRVKDLPGVRYHIVRGTLDAVGVKDRQQGRSSALMPTIKQLIRNTRQ : 93
Isc : ---------------------------------------TITPKKPNSALRKVARVRLTSGFEITAYIPGIGHNSQEHSVVLVRGGRVKDLPGVRYHIVRGTLDAVGVKDRQQGRSSAL---------NTRQ : 84
Ili : ---------------------------------------TITPKKPNSALRKVARVRLTSGFEITAYIPGIGHNSQEHSVVLVRGGRVKDLPGVRYHIVRGTLDAVGVKDRQQGRSSALMPTIKQLIRNTRQ : 93

 140 *
Ise : -------------------------- : -
Ici : -------------------------- : -
Ipe : -------------------------- : -
Ath : PK------------------------ : 123
Ikl : PIRNVTKSPALRGCPQRRGTCTRVYV : 119
Isc : PIRNVTKSPALRGCPQRRGTCTRVYV : 110
Ili : PIRNVTK------------------- : 100

**Figure S65 Multiple sequence alignment of the deduced amino acid sequences of RPS12 protein.** The origins of the protein sequences used in this alignment are from: *Arabidopsis thaliana* (Ath), *Iodes klaineana* (Ikl), *Iodes cirrhosa* (Ici), *Iodes seretii* (Ise), *Iodes scandens* (Isc), *Iodes perrieri* (Ipe) and *Iodes liberica* (Ili). The amino acid numbers for each sequence are indicated on the right. In the sequence alignment, identical residues are shown with a black background, and similar residues are shown with a gray background.

* 20 * 40 * 60 * 80 * 100
Ikl : MARKSLIQREKKRQKLEQKYQLIRRSLKKEIRKVTLLSDKWEIYGKLQSPPRNSAPTRLHRRCFSTGRPRANYRYFGLSGHILREMVHACLLPGATRSSW : 100
Ili : MARKSLIQREKKRQKLEQKYQLIRRSLKKEIRKVTLLSDKWEIYGKLQSPPRNSAPTRLHRRCFSTGRPRANYRYFGLSGHILREMVHACLLPGATRSSW : 100
Ise : MARKSLIQREKKRQKLEQKYQLIRRSLKKEISKVTLLSDKWEIYGKLQSPPRNSAPTRLHRRCFSTGRPRANYRYFGLSGHILREMVHACFLPGATRSSW : 100
Ipe : MARKSLIQREKKRQKLEQKYQLIRRSLKKEISKVTLLSDKWEIYGKLQSLPRNSAPTRLHRRCFSTGRPRANYRYFGLSGHILREMVHACLLPGATRSSW : 100
Isc : MARKSLIQREKKRQKLEQKYQLIRRSLKKEMSKVTLLSDKWEIYGKLQSLPRNSAPTRLHRRCFSTGRPRANYRYFGLSGHILREMVHACLLPGATRSSW : 100
Ici : MARKSLIQREKKRQKLEQKYQLIRRSLKKEISKVTLLSDKWEIYGKLQSLPRNSAPTRLHRRCFSTGRPRGNYRYFGLSGHILREMVHACLLPGATRSSW : 100
Ath : MAKKSLIYREKKRQKLEKKYHLIRRSSKKEISKIPSLSEKWKIHGKLQSPPRNSAPTRLHRRCFSTGRPRANYRDFGLSGHILREMVQACLLPGATRSSW : 100

**Figure S66 Multiple sequence alignment of the deduced amino acid sequences of RPS14 protein.** The origins of the protein sequences used in this alignment are from: *Arabidopsis thaliana* (Ath), *Iodes klaineana* (Ikl), *Iodes cirrhosa* (Ici), *Iodes seretii* (Ise), *Iodes scandens* (Isc), *Iodes perrieri* (Ipe) and *Iodes liberica* (Ili). The amino acid numbers for each sequence are indicated on the right. In the sequence alignment, identical residues are shown with a black background, and similar residues are shown with a gray background.

* 20 * 40 * 60 * 80
Ise : -------------------------------------------DYSSQRGLRKILGKRQRLLTYLSKKNRIRSKELINQLDIRETKNR : 45
Ath : MIKNIVISFEEQKEESRGSVEFQVFSFTNKIRRLTSHLELHRKDYLSQRGLRKILGKRQRLLAYLSKKNRVRYKELINQLNIRELKTR : 88
Ili : ----------------------QVFSSTTKIRRLTFHLELHKKDYSSQRGLRKILGKRQRLLAYLSKKNRIRSKELINQLDIRETKNR : 66
Ipe : -MVKKSLISVSSQEENRGSVEFQVFSFTTKIRRLTFHLELHKKDYSSQRGLRKILGKRQRLLAYLSKKNRIRSKELINQLDIRKTKNR : 87
Isc : ----------SSQEENRGSVEFQVFSFTTKIRRLTFHLELHKKDYS------------------------------------------ : 36
Ikl : -MVKKSLISVSSQEENRGSVEFQVFSFTTKIRRLTFHLELHKKDYSSQRGLRKILGKRQRLLAYLSKKNRIRSKELINQLDIRETKN- : 86
Ici : -MVKKSLISVSSQEENRGSVEFQVFSFTTKIRRLTFHLELHKKDYSSQRGLRKILGKRQRLLAYLSKKNRIRSKELINQLDIRETKNR : 87

**Figure S67 Multiple sequence alignment of the deduced amino acid sequences of RPS15 protein.** The origins of the protein sequences used in this alignment are from: *Arabidopsis thaliana* (Ath), *Iodes klaineana* (Ikl), *Iodes cirrhosa* (Ici), *Iodes seretii* (Ise), *Iodes scandens* (Isc), *Iodes perrieri* (Ipe) and *Iodes liberica* (Ili). The amino acid numbers for each sequence are indicated on the right. In the sequence alignment, identical residues are shown with a black background, and similar residues are shown with a gray background.

* 20 * 40 * 60 * 80 *
Ipe : -----------------AVYRIVAIDVRSRREGRDLRKVGFYDPIKNQTYLNVPAILYFLERGAQPTGTIQDILKKAEVFKKFQPNQTKAN : 74
Ili : -----------------AVYRIVAIDVRSRREGRDLRKVGFYDPIKNQTYLNVPAILYFLERGAQPTGTIQDILKKAEVFKKFHPNQTKAN : 74
Isc : -----------------AVYRIVAIDVRSRREGRDLRKVGFYDPIKNQTYLNVTAILF--------------------------------- : 41
Ici : ---MVKLRLKRCGRKQRAVYRIVAIDVRSRREGRDLRKVGFYDPIKNQTYLNVPAILYFLERGAQPTGTIQDILKKAEVFKKFHPNQTKAN : 88
Ikl : MVKLRLKRCGRKQRATRAVYRIVAIDVRSRREGRDLRKVGFYDPIKNQTYLNVPAILYFLERGAQPTGTIQDILKKAEVFKKFHPNQTKAN : 91
Ise : MVKLRLKRCGRKQRATRAVYRIVAIDVRSRREGRDLRKVGFYDPIKNQ------------------------------------------- : 48
Ath : MVKLRLKRCGRKQ---RAVYRILAIDVRYRREGRDLSKVGFYDPITNQTFLNLSAILDFLKKGAQPTRTAHDISKKAGIFTE--------- : 79

**Figure S68 Multiple sequence alignment of the deduced amino acid sequences of RPS16 protein.** The origins of the protein sequences used in this alignment are from: *Arabidopsis thaliana* (Ath), *Iodes klaineana* (Ikl), *Iodes cirrhosa* (Ici), *Iodes seretii* (Ise), *Iodes scandens* (Isc), *Iodes perrieri* (Ipe) and *Iodes liberica* (Ili). The amino acid numbers for each sequence are indicated on the right. In the sequence alignment, identical residues are shown with a black background, and similar residues are shown with a gray background.

* 20 * 40 * 60 * 80 * 100
Isc : --------------------------------------------------------------------ARILSSLPFLNNEKQFE---RSESTIITPGLRNR-- : 31
Ici : MDKSKRPFLKSKRFFRRRLPPIESGDRIDYRNMSLISRFISEQGKILSRRVNRLTLKQQRLITIAIKQARILSSLPFLNNEKQFEKFERSESTIITPGLRNRKK : 104
Ili : ---------------------IESGDRIDYRNMSLISRFISEQGKILSRRVNRLTLKQQRLITIAIKQARILSSLPFLNNEKQFE---RSESTIITPGLRNR-- : 78
Ikl : ---------------------IESGDRIDYRNMSLISRFISEQGKILSRRVNRLTLKQQRLITIAIKQARILSSLPFLNNEKQFE---RSESTIITPGLRNRKK : 80
Ipe : ---------------------IQSGDRIDYRNMSLISRFISEQGKILSRRVNRLTLKQQRLITIAIKQARILSSLPFLNNEKQFE---RSESTIITPGLRNRKK : 80
Ise : ---------------------IESGDRIDYRNMSLISRFISEQGKILSRRVNRLTLKQQRLITIAIKQARILSSLPFLNNEKQFE---RSESTTIT-------- : 72
Ath : MNKSKRLFTKSKRSFRRRLPPIQSGDRIDYRNMSLISRFISEQGKILSRRVNRVTLKQQRLITIAIKQARILSLLPFLNNQKQFE---RSESTPRTTSLRTRKK : 101

**Figure S69 Multiple sequence alignment of the deduced amino acid sequences of RPS18 protein.** The origins of the protein sequences used in this alignment are from: *Arabidopsis thaliana* (Ath), *Iodes klaineana* (Ikl), *Iodes cirrhosa* (Ici), *Iodes seretii* (Ise), *Iodes scandens* (Isc), *Iodes perrieri* (Ipe) and *Iodes liberica* (Ili). The amino acid numbers for each sequence are indicated on the right. In the sequence alignment, identical residues are shown with a black background, and similar residues are shown with a gray background.

* 20 * 40 * 60 * 80 *
Isc : VTRSLKKNPFVSNHLLRKMDKLNTKAEKEIIITWSRASTIIPTMIGHTIAIHNGKGHLPIYITDRMVGHKLGE---FAPTLNFR-GHAKSDN---- : 88
Ise : VTRSLKKNPFVSNHLLRKMDKLNTKAEKEIIITWSRASTIIPTMIGHTIAIHNGKGHLPIYITDRMVGHKLGE---FAPTLNFR-GHAKSDNRSRR : 92
Ipe : VTRSLKKNPFVSNHLLRKMDKLNTKAEKEIIITWSRASTIIPTMIGHTIAIHNGKGHLPIYITDRMVGHKLGE---FAPTLNFR-GHAKNDNRSRR : 92
Ici : VTRSLKKNPFVANHLLRKMDKLNTKAEKEIIITWSRASTVIPTMIGHTIAIHNGKGHLPIYITDRMVGHKLGE---LAPTLNFR-GHAKSDNRSRR : 92
Ath : MTRSLKKNPFVAKHLLRKIEKLNTKAEKEIIITWSRASTIIPTMIGHTIAIHNGREHLPVYIIDLMVGHKLGE---FSPTINFR-GHAKNDNRSRR : 92
Ikl : VTRSLKKNPFVSNHLLRKMDKLNTKAEKEIIITWSRASTIIPTMIGHTIAIHNGKGHLPIYITDRMVGHKLGE---VAPTFHFR-GHAKSDNRSRR : 92
Ili : ------------NHLLRKMDKLNTKAEKEIIITWSRASTIIPTMIGHTIAIHNGKGHLPIYIIDRMVGHKLGEGTQIGRISTFHFGHAKSNNRSHR : 84

**Figure S70 Multiple sequence alignment of the deduced amino acid sequences of RPS19 protein.** The origins of the protein sequences used in this alignment are from: *Arabidopsis thaliana* (Ath), *Iodes klaineana* (Ikl), *Iodes cirrhosa* (Ici), *Iodes seretii* (Ise), *Iodes scandens* (Isc), *Iodes perrieri* (Ipe) and *Iodes liberica* (Ili). The amino acid numbers for each sequence are indicated on the right. In the sequence alignment, identical residues are shown with a black background, and similar residues are shown with a gray background.

* 20 * 40 * 60 * 80 * 100 * 120
Ise : -MIFKSFLLGNLVSLCMKIINSVVVVGLYYGFLTTFSIGPSYLFLLQAQVMEEG---TEKKVSATTGFITGQLMMFISIYYAPLHLALGRPHTITVLALPYLLFHFFWNNHKHFSDYRSTTRNSMRNLS : 125
Ili : ---------------------------------------PSYLFLLQAQVMEEG---TEKKVSATTGFITGQLMMFISIYYAPLHLALGRPHTITVLALPYLLFHFFWNNHKHFFDYRSTTRNSMRNLS : 87
Ikl : -MILKSFLLGNLVSLCMKIINSVVVVGLYYGFLTTFSIGPSYLFLLQAQVMEEG---TEKKVSATTGFITGQLMMFISIYYAPLHLALGRPHTITVLALPYLLFHFFWNNHKHFFDYRSTTRNSMRNLS : 125
Isc : -MILKSFLLGNLVSLCMKIINSVVVVGLYYGFLTTFSIGPSYLFLLQAQVMEEG---TEKKVSATTGFITGQLMMFISIYYAPLHLALGRPHTITVLALPYLLFHFFWNNHKHFFDYRSTTRNSMRNLS : 125
Ipe : -MILKSFLLGNLVSLCMKIINSVVVVGLYYGFLTTFSIGPSYLFLLQAQVMEEG---TEKKVSATTGFITGQLMMFISIYYAPLHLALGRPHTITVLALPYLLFHFFWNNHKHFFDYRSTTRNSMRNLS : 125
Ici : -MILKSFLLGNLVSLCMKIINSVVVVGLYYGFLTTFSIGPSYLFLLQAQVMEEG---TEKKVSATTGFITGQLMMFISIYYAPLHLALGRPHTITVLALPYLLFHFFWNNHKHFFDYRSTTRNSMRNLS : 125
Ath : MMVFQSFILGNLVSLCMKIINSVVVVGLYYGFLTTFSIGPSYLFLLRARVMDEGEEGTEKKVSATTGFIAGQLMMFISIYYAPLHLALGRPHTITVLALPYLLFHFFWNNHKHFFDYGSTTRNEMRNLR : 129

 * 140 * 160 * 180 * 200 * 220 * 240 * 2
Ise : IQCVFLNNLIFQLFNHFILPSSMLARLVNIYMFRCNNKMLFVTSSFVGWLIGHILFMKWVGLVLVWIRQNHSIRSNKYIRSNKYIRSNKYLVSELRNSMARIFSILLFITCVYYLGRIPSPILTKKLKE : 254
Ili : IQCVFLNNLIFQLFNHFILPSSMLARLVNIYMFRCNNKMLFVTSSFVGWLIGHILFMKWVGLVLVWIRQNHSIRSNKYIRSNKYIRSNKYLVSELRNSMARIFSILLFITCVYYLGRIPSPILTKKLKE : 216
Ikl : IQCVFLNNLIFQLFNHFILPSSMLARLVNIYMFRCNNKMLFVTSSFVGWLIGHILFMKWVGLVLVWIRQNHSIRSNKYIRSNKYIRSNKYLVSELRNSMARIFSILLFITCVYYLGRIPSPILTKKLKE : 254
Isc : IQCVFLNNLIFQLFNHFILPSSMLARLVNIYMFRCNNKMLFVTSSFVGWLIGHILFMKWVGLVLVWIRQNHSIRSNKYIRSNKYIRSNKYLVSELRNSMARIFSILLFITCVYYLGRIPSPILTKKLKE : 254
Ipe : IQCVFLNNLIFQLFNHFILPSSMLARLVNIYMFRCNNKMLFVTSSFVGWLIGHILFMKWVGLVLVWIRQNHSIRSNKYIRSNKYIRSNKYLVSELRNSMARIFSILLFITCVYYLGRIPSPILTKKLKE : 254
Ici : IQCVFLNNLIFQLFNHFILPSSMLARLVNIYMFRCNNKMLFVTSSFVGWLIGHILFMKWVGLVLVWIRQNHSIRSNKYIRSNKYIRSNKYLVSELRNSMARIFSILLFITCVYYLGRIPSPILTKKLKE : 254
Ath : IQCVFLNNLIFQLFNHFILPSSMLARLVNIYMFRCNNKMLFVTSSFVGWLIGHILFMKWVGLVLVWIQQNNSIRSNVVIRSNKY----KFLVSELRNSMARIFSILLFITCVYYLGRIPSPIFTKKLKG : 254

 60 * 280 * 300 * 320 * 340 * 360 * 380
Ise : TSKTEERVEIEEETDVEIERASEMKGTKQEQEGSTEEDPSPYLFSEEKGDPDKIDETEEIRVNGKEKTKDEFHFQFTETCYNNSPVYEVSYLDRNKDWNQENWKFQLLEDE------------------ : 365
Ili : TSKTEERVEIEEETDVEIERASEMKGTKQEQEGSTEEDPSPYLFSEEKGDPDKIDETEEIRVNGKEKTKDEFHFHFTETCYNNSPVYEVSYLDRNKDWNQENWKFQLLEDE------------------ : 327
Ikl : TSKTEERVEIEEETDVEIERASEMKGTKQEQEGSTEEDPSPYLFSEEKGDPDKIDETEEIRVNGKEKTKDEFHFHFTETCYNNSPVYEVSYLDRNKDWNQENWKFQLLEDE------------------ : 365
Isc : TSKTEERVEIEEETDVEIERDSEMKGTKQEQEGSTEEDPSPSLFSEEKGDPDKIDETEEIRVNGKEKTKDEFNFHFTETCYNNSPVYEVSYLDRNKDWNQENCKFQLLEDE------------------ : 365
Ipe : TSKTEERVEIEEETDVEIERASEMKGTKQEQEGSTEEDPSPYLFSEEKGDPDKIDETEEIRVNGKEKT------------------YEVSYLDRNKDWNQENWKFQLLEDE------------------ : 347
Ici : TSKTEERVEIEEETDVEIERASEMKGTKQEQEGSTEEDPSPSLFSEEKGDPDKIDETEEIRVNGKEKTKDEFN--------NNSPVYEVSYLDRNKDWN-----LQLLEDKVIN--------------- : 355
Ath : T--------------------SETGGTKQDQEVSTEEAPFPSLFSEEGEDLDKIDEMEEIRVNGKDKINKDDEFHVR-TYYNYKTVSENLYGNK-ENSNLEFFKIKKKEDHFLWFEKPFVTLVFDYKRW : 361

* 400 * 420 * 440 * 460 * 480 * 500 *
Ise : --------------------------------------------------------------------------------------------------------------------------------- : -
Ili : --------------------------------------------------------------------------------------------------------------------------------- : -
Ikl : --------------------------------------------------------------------------------------------------------------------------------- : -
Isc : --------------------------------------------------------------------------------------------------------------------------------- : -
Ipe : --------------------------------------------------------------------------------------------------------------------------------- : -
Ici : --------------------------------------------------------------------------------------------------------------------------------- : -
Ath : NRPNRYIKNDKIENIVRNEMSQYFFYTCQSDGKERISFTYPPNLSTFFEMIQKRIPSFTKEKKTFDQVSTYWSLIHEEKRENLKKEFLNRIEALDKEWSVENILEKTTRFCYNEAKKEYLPKIYDPFLH : 490

 520 * 540 * 560 * 580 * 600 * 620 * 640
Ise : --------------------------------------------------------------------------------------------------------------------------------- : -
Ili : --------------------------------------------------------------------------------------------------------------------------------- : -
Ikl : --------------------------------------------------------------------------------------------------------------------------------- : -
Isc : --------------------------------------------------------------------------------------------------------------------------------- : -
Ipe : --------------------------------------------------------------------------------------------------------------------------------- : -
Ici : --------------------------------------------------------------------------------------------------------------------------------- : -
Ath : GISRGRIKKLPPFQIITETYRKNNLGGSWINKIHGLLLKINYKKFEQTIEKFNRKSLSIEKKLSFFSEPQQEEKINSEEEIKTFKFLFDIVRTDSNDQTLIKNFMDFPEINKKVPRWSYKLISELEELE : 619

 * 660 * 680 * 700 * 720 * 740 * 760 *
Ise : --------------------------------------------------------------------------------------------------------------------------------- : -
Ili : --------------------------------------------------------------------------------------------------------------------------------- : -
Ikl : --------------------------------------------------------------------------------------------------------------------------------- : -
Isc : --------------------------------------------------------------------------------------------------------------------------------- : -
Ipe : --------------------------------------------------------------------------------------------------------------------------------- : -
Ici : --------------------------------------------------------------------------------------------------------------------------------- : -
Ath : GENEENVPMEPGIRSRKAKRVVVFTDKEPHGEIYTNLKDNQNSDQNDEMALIRYSQQSDFRREIIKGSMRSQRRKTVIWEFFQAKVHSPLFFDRIDKLFFFSFDIWGLKKKIIKNFIWKKKIDKKEEEQ : 748

780 * 800 * 820 * 840 * 860 * 880 * 900
Ise : --------------------------------------------------------------------------------------------------------------------------------- : -
Ili : --------------------------------------------------------------------------------------------------------------------------------- : -
Ikl : --------------------------------------------------------------------------------------------------------------------------------- : -
Isc : --------------------------------------------------------------------------------------------------------------------------------- : -
Ipe : --------------------------------------------------------------------------------------------------------------------------------- : -
Ici : --------------------------------------------------------------------------------------------------------------------------------- : -
Ath : SKREETRRIEIAETWDSFLFAQIIRGSLLVTQSILRKYIILPLLIIIKNSVRMLLFQFPEWSQDLKDWKREMHVKCTYNGVQLSETEFPRNWLTDGIQIKILFPFYLKPWHKSKFQASQKARLKKTKDK : 877

 * 920 * 940 * 960 * 980 * 1000 * 1020 *
Ise : --------------------------------------------------------------------------------------------------------------------------------- : -
Ili : --------------------------------------------------------------------------------------------------------------------------------- : -
Ikl : --------------------------------------------------------------------------------------------------------------------------------- : -
Isc : --------------------------------------------------------------------------------------------------------------------------------- : -
Ipe : --------------------------------------------------------------------------------------------------------------------------------- : -
Ici : --------------------------------------------------------------------------------------------------------------------------------- : -
Ath : GEKNDFCFLTVWGMETELPFGSAQRKPSFFEPISKELKKRIKKLKKKSFVVLKIFKERAPIFLKVAKETKNWILKNFIFIKGISKRNLIPLFGPREIYELNEPKKDSIISNQMIHELSVQNKSLEWTNS : 1006


 1040 * 1060 * 1080 * 1100 * 1120 * 1140 * 1160
Ise : --------------------------------------------------------------------------------------------------------------------------------- : -
Ili : --------------------------------------------------------------------------------------------------------------------------------- : -
Ikl : --------------------------------------------------------------------------------------------------------------------------------- : -
Isc : --------------------------------------------------------------------------------------------------------------------------------- : -
Ipe : --------------------------------------------------------------------------------------------------------------------------------- : -
Ici : --------------------------------------------------------------------------------------------------------------------------------- : -
Ath : SLSEKKIKNLIDRKKTIRNQIEEISKEKQNLTNSCTKLRYDSKIIESSKKIWQTFKRKNTRLIRKSIFFFKFCIEQMSIAIFLGIINIPRITTQLFFESTKKILDKYIYKNEENGEKKKNTLYFISTIK : 1135

* 1180 * 1200 * 1220 * 1240 * 1260 * 1280 *
Ise : --------------------------------------------------------------------------------------------------------------------------------- : -
Ili : --------------------------------------------------------------------------------------------------------------------------------- : -
Ikl : --------------------------------------------------------------------------------------------------------------------------------- : -
Isc : --------------------------------------------------------------------------------------------------------------------------------- : -
Ipe : --------------------------------------------------------------------------------------------------------------------------------- : -
Ici : --------------------------------------------------------------------------------------------------------------------------------- : -
Ath : NLISNKKKMSYDLCSLSQAYVFYKLSQIKVSNFCKLKAVLEYNICITSFFVKNKIKVFFQEHGIFHYELKNKTFLNSEVNQWKNWLRSQYQYNLPQISWARLVTQNWKNKINKDSLVLNPSLTKEDSYE : 1264

 1300 * 1320 * 1340 * 1360 * 1380 * 1400 * 142
Ise : --------------------------------------------------------------------------------------------------------------------------------- : -
Ili : --------------------------------------------------------------------------------------------------------------------------------- : -
Ikl : --------------------------------------------------------------------------------------------------------------------------------- : -
Isc : --------------------------------------------------------------------------------------------------------------------------------- : -
Ipe : --------------------------------------------------------------------------------------------------------------------------------- : -
Ici : --------------------------------------------------------------------------------------------------------------------------------- : -
Ath : KKKFDNYKKQKFFEADSLLNPKHNVKKDSIYNLFCYKSIHSTEKNFDMSIGIALDNCLVSSFLEKYNIRGMGEIRHRKYLDWRILNFWFTKKVTIEPWVDTKSKKKYINTKVQNYQKIDKITQTDLANK : 1393

 0 * 1440 * 1460 * 1480 * 1500 * 1520 * 1540
Ise : --------------------------------------------------------------------------------------------------------------------------------- : -
Ili : --------------------------------------------------------------------------------------------------------------------------------- : -
Ikl : --------------------------------------------------------------------------------------------------------------------------------- : -
Isc : --------------------------------------------------------------------------------------------------------------------------------- : -
Ipe : --------------------------------------------------------------------------------------------------------------------------------- : -
Ici : --------------------------------------------------------------------------------------------------------------------------------- : -
Ath : KRNFFDWMGMNEEILNQRITNFEFFFFPEFFLFSSTYKMKPWVIPIKLLLLNFNENINVNKKIIRKKKGFIPSNEKESLRFYNLNKEEKESAGQVELESDKETKRNPEAARLNQEKNIEENFAESTIKK : 1522

* 1560 * 1580 * 1600 * 1620 * 1640 * 1660 * 1
Ise : --------------------------------------------------------------------------------------------------------------------------------- : -
Ili : --------------------------------------------------------------------------------------------------------------------------------- : -
Ikl : --------------------------------------------------------------------------------------------------------------------------------- : -
Isc : --------------------------------------------------------------------------------------------------------------------------------- : -
Ipe : --------------------------------------------------------------------------------------------------------------------------------- : -
Ici : --------------------------------------------------------------------------------------------------------------------------------- : -
Ath : RKNKKQYKSNTEAELDLFLTRYSRFQLRWNCFFNQKILNNVKVYCLLVRLNNPNEIAVSSIERGEMSLDILMIEKNFTFAKLMKKGILIIEPVRLSVQNDGQLIIYRTIGISLVHKNKHKISKRYKKKS : 1651

 680 * 1700 * 1720 * 1740 * 1760 * 1780 * 1800
Ise : --------------------------------------------------------------------------------------------------------------------------------- : -
Ili : --------------------------------------------------------------------------------------------------------------------------------- : -
Ikl : --------------------------------------------------------------------------------------------------------------------------------- : -
Isc : --------------------------------------------------------------------------------------------------------------------------------- : -
Ipe : --------------------------------------------------------------------------------------------------------------------------------- : -
Ici : --------------------------------------------------------------------------------------------------------------------------------- : -
Ath : YINKKFFEKSITKYQNKTVNKKKNNYDFFVPEKILSPKRRREFRILICFNLKKKNARDTNSRFDKNIQNLTTVLHKKKDLDLDKDKNNLINLKSFLWPNFKLEDLACMNRYWFNTTNGNHFSMIRIRMY : 1780

 *
Ise : ------ : -
Ili : ------ : -
Ikl : ------ : -
Isc : ------ : -
Ipe : ------ : -
Ici : ------ : -
Ath : TRFPIP : 1786

**Figure S71 Multiple sequence alignment of the deduced amino acid sequences of YCF1 protein.** The origins of the protein sequences used in this alignment are from: *Arabidopsis thaliana* (Ath), *Iodes klaineana* (Ikl), *Iodes cirrhosa* (Ici), *Iodes seretii* (Ise), *Iodes scandens* (Isc), *Iodes perrieri* (Ipe) and *Iodes liberica* (Ili). The amino acid numbers for each sequence are indicated on the right. In the sequence alignment, identical residues are shown with a black background, and similar residues are shown with a gray background.

* 20 * 40 * 60 * 80 * 100 * 120
Ikl : MKGHQFKSWIFELREILREIKNSHYFLDSWTQFNSVGSFIHIFFHQERFIKLFDPRIWSILLSRNSQGSTSNRYFTIKGVILFVVAVLIYRINNRNMVERKNLYLIGLLPIPMNSIGPRNDTLEESVGS : 129
Ili : MKGHQFKSWIFELREILREIKNSHYFLDSWTQFNSVGSFIHIFFHQERFIKLFDPRIWSILLSRNSQGSTSNRYFTIKGVILFVVAVLIYRINNRNMVERKNLYLIGLLPIPMNSIGPRNDTLEESVGS : 129
Isc : --------------------------------------------------------------------------------------------------------------------------------- : -
Ici : MKGHQFKSWIFELREILREIKNSHYFLDSWTQFNSVGSFIHIFFHQERFIKLFDPRIWSILLSRNSQGSTSNRYFTIKGVILFVVAVLIYRINNRNMVERKNLYLIGLLPIPMNSIGPRNDTLEESVGS : 129
Ipe : MKGHQFKSWIFELREILREIKNSHYFLDSWTQFNSVGSFIHIFFHQERFIKLFDPRIWSILLSRNSQGSTSNRYFTIKGVILFVVAVLIYRINNRNMVERKNLYLIGLLPIPMNSIGPRNDTLEESVGS : 129
Ise : MKGHQFKSWIFELREILREIKNSHYFLDSWTQFNSVGSFIHIFFHQERFIKLFDPRIWSILLSRNSQGSTSNRYFTIKGVILFVVAVLIYRINNRNMVERKNLYLIGLLPIPMNSIGPRNDTLEESVGS : 129
Ath : MKGHQFKSWIFELREIVREIKNAHYFLDSWTQFNSVGSFIHIFFHQERFRKLLDPRIFSILLLRNSQGSTSNRYFTIKGVVLFVVAALLYRINNRNMVESKNLYLKGLLPIPMNSIGPRNDTSEESFGS : 129

 * 140 * 160 * 180 * 200 * 220 * 240 * 2
Ikl : SNINRLIVSLLYLPKGKKISESCFLNPKESTWVLPITN------------------------------------------------------------------------------------------- : 167
Ili : SNINRLIVSLLYLPKGKKISESCFLNPKESTWVLPITN------------------------------------------------------------------------------------------- : 167
Isc : --------------------------------------------------------------------------------------------------------------------------------- : -
Ici : SNINRLIVSLLYLPKGKKISESCFLNPKESTWVLPITN------------------------------------------------------------------------------------------- : 167
Ipe : SNINRLIVSLLYLPKGKKISESCFLNPKESTWVLPLTN------------------------------------------------------------------------------------------- : 167
Ise : SNINRLIVSLLYLPKGKKISESCFLNPKESTWVLPITN------------------------------------------------------------------------------------------- : 167
Ath : CNINRLIVSLLYLTKGKKISESCFRDPKESTWVLPITQKCIMPESNWSSRWWRNWIGKKRGFCCKISNETVAGIDISFKEKDIKYLEFLFVYYMDDPIRKGHDWELFDRLSPSKRRNIINLNSGQLFEI : 258

 60 * 280 * 300 * 320 * 340 * 360 * 380
Ikl : --------------------------------------------------------------------------------------------------------RFFSKVRNVSSNIQYDFTRSSFVQV : 192
Ili : --------------------------------------------------------------------------------------------------------RFFSKVRNVSSNIQYDFTRSSFVQV : 192
Isc : --------------------------------------------------------------------------------------------------------------------------------- : -
Ici : --------------------------------------------------------------------------------------------------------RFFSKVRNVSSNIQYDFTRSSFVQV : 192
Ipe : --------------------------------------------------------------------------------------------------------RFFSKVRNVSSNIQYDFTRSSFVQV : 192
Ise : --------------------------------------------------------------------------------------------------------RFFSKVRNVSSNIQYDFTRSSFVQV : 192
Ath : LVKDWICYLMFAFREKIPIEVEGFCKQQGAGSTIQSNDIEHVSHLFSRNKWAISLQNCAQFHMWQFHQDLFVSWGKNPHESDFFRKISRENWIWLDNVWLVNKDRFFSKVRNVSSNIQYDSTRSSFVQV : 387

* 400 * 420 * 440 * 460 * 480 * 500 *
Ikl : TDSSQLKGSSDQSRDHLDCISNEDS--AYHTLINQREIQQLKERSILWDPSFLQTERTEIESDRFPKCLSGYSSMSRLFTEREKQMINHLLPEEIEEVLGNPTRSVRSFFSDRWSELHLGSNPTERSTR : 319
Ili : TDSSQLKGSSNQSRDHLDCISNEDS--AYHTLINQREIQQLKERSILWDPSFLQTERTEIESDRFPKCLSGYSSMSRLFTEREKQMINHLLPEEIEEVLGNPTRSVRSFFSDRWSELHLGSNPTERSTR : 319
Isc : --------------------------------------------------------------------------------------------------------------------------------- : -
Ici : TDSSQLKGSSDQSRDHLDCISNEDS--EYHTLINQREIQQLKERSILWDPSFLQTEQTEIESDRFPKCLSGYSSMSRLFTEREKQMINHLLPEEIEEVLGNPTRSVRSFFSDRWSELHLGSNPTERSTR : 319
Ipe : TDSSQLKGSSDQSRDHLDCISNEDS--AYHTLINQREIQQLKERSILWDPSFLQTERTEIESDRFPKCLSGYSSMSRLFTEREKQMINHLLPEEIEEVLGNPTRSVRSFFSDRWSELHLGSNPTERSTR : 319
Ise : TDSSQLKGSSDQSRDHLDCISNEDS--AYHTLINQREIQQLKERSILWDPSFLQTERTEIESDRFPKCLSGYSSMSRLFTEREKQMINHLLPEEIEEVLGNPTRSVRSFFSDRWSELHLGSNPTERSTR : 319
Ath : TDSSQLNGSSDQFIDPFDSISNEDSEYHYHTLINQREIQQLKERSILLDPSFIQTEGREIESDRFPKYLSGYSSMPRLFTEREKRMNNHLLPEESEEFLGNPTRAIRSFFSDRWSELHLGSNPTERSTR : 516

 520 * 540 * 560 * 580 * 600 * 620 * 640
Ikl : DQKLLKKEQDLSFVPSRRSENKEMVNIFKIITYLQNTVSIHPISSDPGCDMVPKDEPDMDSSNKISFLNKNPFFDLFHLFHDWNRGGYTLHHDFESEERFQEMADLFTLSITEPDLVYHKRFAFSIDSY : 448
Ili : DQKLLKKEQDLSFVPSRRSENKEMVNIFKIITYLQNTVSIHPISSDPGCDMVPKDEPDMDSSNKISFLNKNPFFDLFHLFHDWNRGGYTLHHDFESEERFQEMADLFTLSITEPDLVYHKRFAFSIDSY : 448
Isc : --------------------------------------------------------------------------------------------------------------------------------- : -
Ici : DQKLLKKEQDLSFVPSRRSENKEMVNIFKIITYLQNTVSIHPISSDPGCDMVPKDEPDMDSSNKISFLNKNPFFDLFHLFHDWNRGGYTLHHDFESEERFQEMADLFTLSITEPDLVYHKRFAFSIDSY : 448
Ipe : DQKLLKKEQDLSFVPSRRSENKEMVNIFKIITYLQNTVSIHPISSDPGCDMVPKDEPDMDSSNKISFLNKNPFFDLFHLFHDWNRGGYTLHHDFESEERFQEMADLFTLSITEPDLVYHKRFAFSIDSY : 448
Ise : DQKLLKKEQDLSFVPSRRSENKEMVNIFKIITYLQNTVSIHPISSDPGCDMVPKDEPDMDSSNKISFLNKNPFFDLFHLFHDWNRGGYTLHHDFESEERFQEMADLFTLSITEPDLVYHKRFAFSIDSY : 448
Ath : DQKLLKKEQDVSFVPSRRSENKEIVNIFKIITYLQNTVSIHPISSDLGCDMVPKDELDMDSSNKISFLNKNPFFDLFHLFHERKRGGYTLRH--ESEERFQEMADLFTLSITEPDLVYHKGFAFSIDSY : 643

 * 660 * 680 * 700 * 720 * 740 * 760 *
Ikl : GLDQKQFLNEVFNSRDESKKKSLLVLPPIFYEENESFYRRIRKKRVRISCGNDLEDPKPKIVVFASNNIMEAVNQYRLIRNLIQIQYST--YGYIRNILNRFFLMNKSDRNFEYGIQRDQIGKDTLNHR : 575
Ili : GFDQKQFLNEVFNSRDESKKKSLLVLPPIFYEENESFYRRIRKKWVRISCGNDLEDPKPKIVVFASNNIMEAVNQYRLIRNLIQIQYST--YGYIRNILNRFFLMNKSDRNFEYGIQRDQIGKDTLNHR : 575
Isc : --------------------------------------------------------------------------------------------------------------------------------- : -
Ici : GLDQKQFLNEVFNSRDESKKKSLLVLPPIFYEENESFYRRIRKKWVRISCGNDLEDPKPKIVVFASNNIMEAVNQYRLIRNLIQIQYST--YGYIRNILNRFFLMNKSDRNFEYGIQRDQIGKDTLNHR : 575
Ipe : GLDQKQFLNEVFNSRDESKKKSLLVLPPIFYEENESFYRRIRKKWVRISCGNDLEDPKPKIVVFASNNIMEAVNQYRLIRNLIQIQYST--YGYIRNILNRFFLMNKSDRNFEYGIQRDQIGKDTLNHR : 575
Ise : GLDQKQFLNEVFNSRDESKKKSLLVLPPIFYEEND--YRRIRKKWVRISCGNDLEDPKPKIVVFASNNIMEAVNQYRLIRNLIQIQYST--YGYIRNILNRFFLMNKSDRNFEYGIQRDQIGKDTLNHR : 573
Ath : GLDQRQFLKEVFNFRDESKKKSLLVLPPIFYEENESFYRRLRKIWVRISCGNYLEDQKR--VVFASNNIMEAVNQYRLIRNMIQIQFQYSPYGYIRNVLNRFFLMKRPDRNFEYGIQRDLIGNDTLNHR : 770

780 * 800 * 820 * 840 * 860 * 880 * 900
Ikl : TIMKYTINRHLSNLKKSQKKGFDPLILISRTERSMNRDPDAYRSKWSNGSKNFQEHLEHFVSEQKSRFQVVFDRLRINQYSIDWSEVIDKKDLSKPLRFFLS----------------------KSLPF : 682
Ili : TIMKYTINRHLSNLKKSQKKGFDPLILISRTERSMNRDPDAYRSKWSNGSKNFQEHLEHFVSEQKSRFQVVFDRLRINQYSIDWSEVIDKKDLSKPLRFFLS----------------------KSLPF : 682
Isc : --------------------------------------------------------------------------------------------------------------------------------- : -
Ici : TIMKYTINRHLSNLKKSQKKGFDPLIFIER---SMNRDPDAYRYKWSNGSK---NFQEHFVSEQKSRFQVVFDRLRINQYSIDWSEVIDKKDLSKPLRFFLS----------------------KSLPF : 676
Ipe : TIMKYTINRHLSNLKKSQKKGFDPLIFISRTKRSMNRDPDAYRYKWSNGSK---NFQEHFVSEQKSRFQVVFDRLRINQYSIDWSEVIDKKDLSKPLRFFLS----------------------KSLPF : 679
Ise : TIMKYTINRHLSNLKKSQKKGFDPLIFISRTERSMNRDPDAYRYKWSNGSKNFQEHLEHFVSEQKSRFQVVFDRLRINQYSIDWSEVIDKKDLSKPLRFFLS----------------------KSLPF : 680
Ath : TIMKDTINQHLSNLKKSQKKWFDPLIFLSQTERSINRDPNAYRYKWSNGSKNFQEHLEHFVSERKSRFQVVFDQLCINQYSIDWSEVIDKKDLSKSLRFFLSKLLRFFLSKLLLFLSKLLLFLSNSLPF : 899

 * 920 * 940 * 960 * 980 * 1000 * 1020 *
Ikl : FFVSFGNIPIHRSEIYIYELKGPNDQLCNQLLESIGLQIVHLKKLKPFLLDDHDTSQKSKFLINGGTISPFLFNKIPKWMIGSFHTRNNRRKSFDNTDS-YFSMIFHDQDNWLNPVKPFHRSSLISSFY : 810
Ili : FFVSFGNIPIHRSEIYIYELKGPNDQLCNQLLESIGLQIVHLKKLKPFLLDDHDTSQKSKFLINGGTISPFLFNKIPKWMIGSFHTRNNRRKSFDNTDS-YFSMIFHDQDNWLNPVKPFHRSSLISSFY : 810
Isc : ------------------------------------------------------TSQKSKFLINGGTISPFLN-KIPKWMIDSFHTRNNRRKSFDNTDS-YFSMIFHDQDNWLNPVKPFHRS------- : 66
Ici : FFVSFGNIPIHRSEIYIYELKGPNDQLCNQLLESIGLQIVHLKKLKPFLLDDHDTSQKSKFLINGGTISPFLFNKIPKWMIDSFHTRNNRRKSFDNTDS-YFSMIFHDQDNWLNPVKPFHRSSLISSFY : 804
Ipe : FFVSFGNIPIHRSEIYIYELKGPNDQLCNQLLESIGLQIVHLKKLKPFLLDDHDTSQKSKFLINGGTISPFLFNKIPKWMIDSFHTRNNRRKSFDNTDS-YFSMIFHDQDNWLNPVKPFHRSSLISSFY : 807
Ise : FFVSFGNIPIHRSEIYIYELKGPNDQLCNQLLESIGFQIVHLKKLKPFLLDDHDTSQKSKFLINGGTISPFLFNKIPKWMIDSFHTRNNRRKSFDNTDS-YFSMIFHDQDNWLNPVKPFHRSSLISSFY : 808
Ath : FFVSFENIPIHRSEIHIYELKGPNDQLCNQLLESIGLQIVHLKKLKPFLLDDHNTSQKSKFLINGGTISPFLFNKIPKWMIDSFHTRKNRRKSFDNTDSAYFSIVSHDQDNWLNPVKPFQRSSLISSFS : 1028

 1040 * 1060 * 1080 * 1100 * 1120 * 1140 * 1160
Ikl : KANRLRFLNNPHHFCFYCNKRFPFYVEKARINN--YDFTYGQFLNILFIRNKIVSLCVGKKKHAFGGRDTIS--SIESQVSNIFIPNGFPQSGNETYNLYKSFHFPSRSDPFVRRAIYSIADISGTPLT : 935
Ili : KANRLRFLNNPHHFCFYCNKRFPFYVEKARINN--YDFTYGQFLNILFIRNKIVSLCVGKKKHAFGGRDTIS--SIESQVSNIFIPNGFPQSGNETYNLYKSFHFPSRSDPFVRRAIYSIADISGTPLT : 935
Isc : -------------FCFYCNKRFPFYVEKARINN--YDFTYGQFLNILFIRNKIVSLCVGKKKHAFGGRDTIS--SIESQVSNIFIPNGFPQSGNETYNLYKSFHFPSRSDPFVRRAIYSIADISGTPLT : 178
Ici : KANRLRFLNNPHHFCFYCNKRFPFYVEKARINN--YDFTYGQFLNILFIRNKIVSLCVGKKKHAFGGRDTIS--SIESQVSNIFIPNGFPQSGNETYNLYKSFHFPSRSDPFVRRAIYSIADISGTPLT : 929
Ipe : KANRLRFLNNPHHFCFYCNKRFPFYVEKARINN--YDFTYGQFLNILFIRNKIVSLCVGKKKHAFGGRDTIS--SIESQVSNIFIPNGFPQSGNETYNLYKSFHFPSRSDPFVRRAIYSIADISGTPLT : 932
Ise : KANRLRFLNNPHHFCFYCNKRFPFYVEKARINN--YDFTYGQFLNILFIRNKIVSLCVGKKKHAFGGRDTIS--SIESQVSNIFIPNGFPQSGNETYNLYKSFHFPSRSDPFVRRAIYSIADISGTPLT : 933
Ath : KANRLRFLNNPHHFCFYCNKRFPFYVEKARLNNSDFTFTYGQFLTILFIHNKTFSSCGGKKKHAFLERDTISPSSIESQVSNIFISNDFPQSGDERYNLYKSFHFPIRSDPLVRRAIYSIADISGTPLI : 1157

* 1180 * 1200 * 1220 * 1240 * 1260 * 1280 *
Ikl : KGQIVNFERTYCQPLSDMNLSDSEGKSLHQYLNFNSNMGLIHTPCSEKYLPSEK------RKKRNLCLKKCVEKGQMYRTFQRDSAFSTLSKWNRFQTYMPWFLTSTGYKYLNLIFLDTFSDLLPILSS : 1058
Ili : KGQIVNFERTYCQPLSDMNLSDSEGKSLHQYLNFNSNMGLIHTPCSEHPKRGKNGIFVRNALRKGRCIEPFVEKGQMYRTFQRDSAFSTLSKWNRFQTYMPWFLTSTGYKYLNLIFLDTFSDLLPILSS : 1064
Isc : KGQIVNFERTYCQPLSDMNLSDSEGKSLHQYLNFNSNMGLIHTPCSEKYLPSEKG------KKRNLCLKKCVEKGRMYRTFQRDSAFSTLSKWNRFQTYMPWFLTSTGYKYLNLIFLDTFSDLLPILSS : 301
Ici : KGQIVNFERTYCQPLSDMNLSDSEGKSLHQYLNFNSNMGLIHTPCSEKYLPSEKG------KKRNLCLKKCVEKGQMYRTFQRDSAFSTLSKWNRFQTYMPWFLTSTGYKYLNLIFLDTFSDLLPILSS : 1052
Ipe : KGQIVNFERTYCQPLSDMNLSDSEGKSLHQYLNFNSNMGLIHTPCSEKYLPSEKR------KKRNLCLKKCVEKGQMYRTFQRDSAFSTLSKWNRFQTYMPWFLTSTGYKYLNLIFLDTFSDLLPILSS : 1055
Ise : KGQIVNFERTYCQPLSDMNLSDSEGKSLHQYLNFNSNMGLIHTPCSEKYLPSEKR------KKRNLCLKKCVEKGQMYRTFQRDSAFSTLSKWNRFQTYMPWFLTSTGYKYLNLIFLDTFSDLLPILSS : 1056
Ath : EGQRVNFERTYCQTLSDMNLSDSEEKSLHQYLNFNSNMGLIHTPCSEKYLQRKKR---------SLCLKKCVDKGQMDRTFQRDSAFSTLSKWNLFQTYMPWFFTSTGYKYLNLIFLDTFSDLLRILSS : 1277

 1300 * 1320 * 1340 * 1360 * 1380 * 1400 * 142
Ikl : SQKFVSIFHDIMHGSDISWRILQKKWCLPQWNLISEISSKCLHNLLLSEEMIHRNNESPLISTHLRSPNVREFLYSILFLLLVAGYLVRTHLLFVSRASSELQTDFEKVKSLMIPSSMIELRKLLDRYP : 1187
Ili : SQKFVSIFHDIMHGSDISWRILQKKWCLPQWNLISEISSKCLHNLLLSEEMIHRNNESPLISTHLRSPNVREFLYSILFLLLVAGYLVRTHLLFVSRASSELQTDFEKVKSLMIPSSMIELRKLLDRYP : 1193
Isc : SQKFVSIFHDIMHGSDISWRILQKKWCLPQWNLISEISSKCLHNLLLSEEMIHRNNESPLISTHLRSPNVREFLYSILFLLLVAGYLVRTHLLFVSRASSELQTDFEKVKSLMIPSSMIELRKLLDRYP : 430
Ici : SQKFVSIFHDIMHGSDISWRILQKKWCLPQWNLISEISSKCLHNLLLSEEMIHRNNESPLISTHLRSPNVREFLYSILFLLLVAGYLVRTHLLFVSRASSELQTDFEKVKSLMIPSSMIELRKLLDRYP : 1181
Ipe : SQKFVSIFHDIMHGSDISWRILQKKWCLPQWNLISEISSKCLHNLLLSEEMIHRNNESPLISTHLRSPNVREFLYSILFLLLVAGYLIRTHLLFVSRASSELQTDFEKVKSLMIPSSMIELRKLLDRYP : 1184
Ise : SQKFVSIFHDIMHGSDISWRILQKKWCLPQWNLISEISSKCLHNLLLSEEMIHRNNESPLISTHLRSPNVREFLYSILFLLLVAGYLVRTHLLFVSRASSELQTDFEKVKSLMIPSSMIELRKLLDRYP : 1185
Ath : SQKFVSIFHDIMHGLDISWRILQKKLCLPQRNLISEISSKSLHNLLLSEEMIHRNNESSLISTHLRSPNVREVLYSILFLLLVAGYIVRTHLLFVSRAYSELQTEFEKIKSLMIPSYMIELRKLLDRYP : 1406

 0 * 1440 * 1460 * 1480 * 1500 * 1520 * 1540
Ikl : TSEPNSFWLKNLFLVALEQLGDSIEEIRGSASGGNMPLGGGPAYGVKSIRSKKKYLNIN---LIDLISLIPNPINRITFSRNTRHLSHTSKEIYSLIRKGKNVNGDWIDDKIESWVANSDSIDDEEREY : 1313
Ili : TSEPNSFWLKNLFLVALEQLGDSIEEIRGSASGGNMPLGGGPAYGVKSIRSKKKYLNIN---LIDLISLIPNPINRITFSRNTRHLSHTSKEIYSLIRKGKNVNGDWIDDKIESWVANSDSIDDEEREY : 1319
Isc : TSEPNSFWLKNLFLVALEQLGDSIEEIRSSASGGNMPLGGGPAYGVKSIRSKKKYLNIN---LIDLISLIPNPINRITFSRNTRHLSHTSKEIYSLIRKGKNVNGDWIDDKIESWVANSDSIDDEEREY : 556
Ici : TSEPNSFWLKNLFLVALEQLGDSIEEIRSSASGGNMPLGGGPAYGVKSIRSKKKYLNIN---LIDLISLIPNPINRITFSRNTRHLSHTSKEIYSLIRKGKNVNGDWIDDKIESWVANSDSIDDEEREY : 1307
Ipe : TSEPNSFWLKNLFLVALEQLGDSIEEIRGSASGGNMPLGGGPAYGVKSIRSKKKYLNIN---LIDLISLIPNPINRITFSRNTRHLSHTSKEIYSLIRKGKNVNGDWIDDKIESWVANSDSIDDEEREY : 1310
Ise : TSEPNSFWLKNLFLVALEQLGDSIEEIRGSASGGNMPLGGGPAYGVKSIRSKKKYLNIN---LIDLISLIPNPINRITFSRNTRHLSHTSQEIYSLIRKGKNVNGDWIDDKIESWVANSDSIDDEEREY : 1311
Ath : TSELNSFWLKNLFLVALEQLGDCLEEIRG--SGGNMLWGGDPAYGVKSIRSKKKDLKINFIDIIDLISIIPNPINRITFSRNTRHLSHTSKEIYSLIRKRKNVSGDWIDDKIESWVANSDSIDDKEREF : 1533

* 1560 * 1580 * 1600 * 1620 * 1640 * 1660 * 1
Ikl : LVQFSTLTTEKGIDQILLSLTHSDHLSKNDSGYQMIEQPGAIYLGYLVDIHKKYLMNYEFNTSCLAERRIFLAHYQTITYSQTSCGANSFHFPSHGKPFSLRLALSPSRGILVIGSIGTGRSYLVKYLV : 1442
Ili : LVQFSTLTTEKGIDQILLSLTHSDHLSKNDSGYQMIEQPGAIYLGYLVDIHKKYLMNYEFNTSCLAERRIFLAHYQTITYSQTSCGANSFHFPSHGKPFSLRLTLSPSRGILVIGSIGTGRSYLVKYLV : 1448
Isc : LVQFSTLTTEKGIDQILLSLTHSDHLSKNDSGYQMIEQPGAIYLGYLVDIHKKYLMNYEFNTSCLAERRIFLAHYQTITYSQTSCGANSFHFPSHGKPFSLRLALSPSRGILVIGSIGTGRSYLVKYLV : 685
Ici : LVQFSTLTTEKGIDQILLSLTHSDHLSKNDSGYQMIEQPGAIYLGYLVDIHKKYLMNYEFNTSCLAERRIFLAHYQTITYSQTSCGANSFHFPSHGKPFSLRLALSPSRGILVIGSIGTGRSYLVKYLV : 1436
Ipe : LVQFSTLTTEKGIDQILLSLTHSDHLSKNDSGYQMIEQPGAIYLGYLVDIHKKYLMNYEFNTSCLAERRIFLAHYQTITYSQTSCGANSFHFPSHGKPFSLRLALSPSRGILVIGSIGTGRSYLVKYLV : 1439
Ise : LVQFSTLTTEKGIDQILLSLTHSDHLSKNDSGYQMIEQPGAIYLGYLVDIHKKYLMNYEFNTSCLAERRIFLAHYQTITYSQTSCGANSFHFPSHGKPFSLRLALSPSRGILVIGSIGTGRSYLVKYLV : 1440
Ath : LVQFSTLRAEKRIDQILLSLTHSDHLSKNDSGYQMIEQPGTIYLRYLVDIHKKYLMNYEFNTSCLAERRIFLAHYQTITYSQTSCGANSFHFPSHGKPFSLRLALSPSRSILVIGSIGTGRSYLVKYLA : 1662

 680 * 1700 * 1720 * 1740 * 1760 * 1780 * 1800
Ikl : TNSYVPFITVFLN-----KPKGFLIDYIDIDDSDDIDDS-----------DDIDASDDIDASDDIDASDDIDRDLDTELGLLTMMNAVTMDMMPEPEIDRFYITLQFELAKAMSPCIIWIPNIHDLDVN : 1555
Ili : TNSYVPFIMVFLN-----KPKGFLMDYIDIDDSDDIDDSDIDDSDDIDDSDDIDASDDIDASDDIDASDDIDRDLDTELGLLTMMNAVTMDMMPEPEIDRFYITLQFELAKAMSPCIIWIPNIHDLDVN : 1572
Isc : TNSYVPFITVFLN-----KPKGFLIDYIDIDDSDDIDDSDIDDSDDIDDSD------------DIDASDDIDRDLDTELGLLTMMNAVTMDMMPEPEIDRFYITLQFELAKAMSPCIIWIPNIHDLDVN : 797
Ici : TNSYVPFITVFLN-----KPKGFLIDYIDIDDSDDIDDSDIDDSDDIDDSDDIDA------SDDIDASDDIDRDLDTELGLLTMMNAVTMDMMPEPEIDRFYITLQFELAKAMSPCIIWIPNIHDLDVN : 1554
Ipe : TNSYVPFITVFLN-----KPKGFLIDYIDIDDSDDIDDSDIDDSDDIDDSDDIDASDDIDASDDIDASDDIDRDLDTELGLLTMMNAVTMDMMPEPEIDRFYITLQFELAKAMSPCIIWIPNIHDLDVN : 1563
Ise : TNSYVPFITVFLN-----KPKGFLIDYIDIDDSDDIDDSD-----DIDASD------------DIDASDDIDRDLDTELGLLTMMNAVTMDMMPEPEIDRFYITLQFELAKAMSPCIIWIPNIHDLDVN : 1547
Ath : TNSYVPFITVFLNKFLDNKPKGFFIDDIDIDDSD-----------------------------DIDASNDIDRELDTELELLTMMNALTMDMML--EIDRFYITLQFELAKAMSPCIIWIPNIHDLDVN : 1760

 * 1820 * 1840 * 1860 * 1880 * 1900 * 1920 *
Ikl : ESNYLSLSLLVNYLSRDCERCSTRNILVIASTHIPQKVDPALIAPN---------KLNTCIKIRRLLIPQQRKHFFTISYTRGFHLEKKMFHTNGFGSITMGSNARDLVALTNEALSISITQKKSIIDT : 1675
Ili : ESNYLSLSLLVNYLSRDCERCSTRNILVIASTHIPQKVDPALIAPN---------KLNTCIKIRRLLIPQQRKHFFTISYTRGFHLEKKMFHTNGFGSITMGSNARDLVALTNEALSISITQKKSIIDT : 1692
Isc : ESNYLSLSLLVNYLSRDCERCSTRNILVIASTHIPQKVILFLLLVAGYLVRTHLLFLNTCIKIRRLLIPQQRKHFFTISYTRGFHFLGNMSRSN----------------------------------- : 891
Ici : ESNYLSLSLLVNYLSRDCERCSTRNILVIASTHIPQKVDPALIAPN---------KLNTCIKIRRLLIPQQRKHFFTISYTRGFHLEKKMFHTNGFGSITMGSNARDLVALTNEALSISITQKKSIIDT : 1674
Ipe : ESNYLSLSLLVNYLSKDCERCSTRNILVIASTHIPQKVDPALIAPN---------KLNTCIKIRRLLIPQQRKHFFTISYTRGFHLEKKMFHTNGFGSITMGSNARDLVALTNEALSISITQKKSIIDT : 1683
Ise : ESNYLSLSLLVNYLSRDCERCSTRNILVIASTHIPQKVDPALIAPN---------KLNTCIKIRRLLIPQQRKHFFTISYTRGFHLEKKMFHTNGFGSITMGSNARDLVALTNEALSISITQKKSIIDT : 1667
Ath : ESSYLALGLLVNSLSRDCERCSTRNILVIASTHIPQKVDPALIAPN---------KLNTCIKIRRLLIPQQRKHFFTLSYTRGFHLEKKMFHTNGFESITMGSSARDLVALTNEALSISITQKKSIIDT : 1880

1940 * 1960 * 1980 * 2000 * 2020 * 2040 * 2060
Ikl : NTIRSALHRQTWDLRSQVRSVQDHGILFYQIGRAVAQNVLLSNCPIDPISIYMKKKSCNEGDSYLYKWYFELGTSMKKLTILLYLLSCSAGSVAQDLWSLPGPDEQNGITSYGLVENDSDLVHGLLEVE : 1804
Ili : NTIRSALHRQTWDLRSQVRSVQDHGILFYQIGRAVAQNVLLSNCPIDPISIYMKKKSCNEGDSYLYKWYFELGTSMKKLTILLYLLSCSAGSVAQDLWSLPGPDEQNGITSYGLVENDSDLVHGLLEVE : 1821
Isc : ------------------------------------------------------------------------------------------------------------------------------EVE : 894
Ici : NTIRSALHRQTWDLRSQVRSVQDHGILFYQIGRAVAQNVLLSNCPIDPISIYMKKKSCNEGDSYLYKWYFELGTSMKKLTILLYLLSCSAGSVAQDLWSLPGPDEQNGITSYGLVENDSDLVHGLLEVE : 1803
Ipe : NTIRSALHRQTWDLRSQVRSVQDHGILFYQIGRAVAQNVLLSNCPIDPISIYMKKKSCNEGDSYLYKWYFELGTSMKKLTILLYLLSCSAGSVAQDLWSLPGPDEQNGITSYGLVENDSDLVHGLLEVE : 1812
Ise : NTIRSALHRQTWDLRSQVRSVQDHGILFYQIGRAVAQNVLLSNCPIDPISIYMKKKSCNEGDSYLYKWYFELGTSMKKLTILLYLLSCSAGSVAQDLWSLPGPDEQNGITSYGLVENDSDLVHGLLEVE : 1796
Ath : NTIRSALHRQTWDLRSQVRSVQDHGILFYQIGRAVAQNVLISNCPIDPISIYMKKKSCNEGDSYLYKWYFELGTSMKKFTILLYLLSCSAGSVAQDLWSLPVPDEKNRITSYGFVENDSDLVHGLLEVQ : 2009

 * 2080 * 2100 * 2120 * 2140 * 2160 * 2180 *
Ikl : GALVGSSRTEKDCSQFDNDRVTLLLRPEPRNPLNMMQNGFFSILDQRFLYEKNESEFEEGE-------VDLEEDLFNHIVWAPRIWRPWGFLFDCIERPNELGFPYWSRSFRGKRIIYDEEDELQEIYD : 1926
Ili : GALVGSSRTEKDCSQFDNDRVTLLLRPEPRNPLNMMQNGFFSILDQRFLYEKNESEFEEGE-------VDLEEDLFNHIVWAPRIWRPWGFLFDCIERPNELGFPYWSRSFRGKRIIYDEEDELQEIYD : 1943
Isc : GALVGSSRTEKDCSQFDNDRVTLLLRPEPRNPLNMMQNGFFSILDQRFLYEKNESEFEEGE-------VDLEEDLFNHIVWAPRIWRPWGFLFDCIERPNELGFPYWSRSFRGKRIIYDE--------- : 1007
Ici : GALVGSSRTEKDCSQFDNDRVTLLLRPEPRNPLNMMQNGFFSILDQRFLYEKNESEFEEGE-------VDLEEDLFNHIVWAPRIWRPWGFLFDCIERPNELGFPYWSRSFRGKRIIYDEEDELQEIYD : 1925
Ipe : GALVGSSRTEKDCSQFDNDRVTLLLRPEPRNPLNMMQNGFFSILDQRFLYEKNESEFEEGE-------VDLEEDLFNHIVWAPRIWRPWGFLFDCIERPNELGFPYWSRSFRGKRIIYDEEDELQEIYD : 1934
Ise : GALVGSSRTEKDCSQFDNDRVTLLLRPEPRNPLNMMQNGFFSILDQRFLYEKNESEFEEGE-------VDLEEDLFNHIVWAPRIWRPWGFLFDCIERPNELGFPYWSRSFRGKRIIYDEEDELQEIYD : 1918
Ath : GALVGSSRTEKDCSQFDNDRVTLLFRSEPRDPLYMMQDGSCSIVDQRFLYEKYESEFEEGEGEGVLDPQQIEEDLFNHIVWAPRIWRPRGFLFDCIERPNELGFPYSAGSFRGKRIIYDEKYELQE--- : 2135

 2200 * 2220 * 2240 * 2260 * 2280 * 2300 * 2320
Ikl : EEDELQENDSEFLQSGTMQYQTRDRSSKEQGLFGISQFIWDPGDPLFFLFKDQPFGSVFSHRELFADEEMSKGLLTLTSQTDPPKSIYKRWFSKNMQEKHFELLINRQRWLRTNSSLSNESFRSNTLSE : 2055
Ili : EEDELQENDSEFLQSGTMQYQTRDRSSKEQGLFGISQFIWDPGDPLFFLFKDQPFGSVFSHRELFADEEMSKGLLTLTSQTDPPKSIYKRWFSKNMQEKHFELLINRQRWLRTNSSLSNESFRSNTLSE : 2072
Isc : --------------------------------------------------------------------------------------------------------------------------------- : -
Ici : EEDELQENDSEFLQSGTMQYQTRDRSSKEQGLFGISQFIWDPGDPLFFLFKDQPFGSVFSHRELFADEEMSKGLLTLTSQTDPPKSIYKRWFSKNMQEKHFELLINRQRWLRTNSSLSNESFRSNTLSE : 2054
Ipe : EEDELQENDSEFLQSGTMQYQTRDRSSKEQGLFGISQFIWDPGDPLFFLFKDQPFGSVFSHRELFADEEMSKGLLTLTSQTDPPKSIYKRWFSKNMQEKHFELLINRQRWLRTNSSLSNESFRSNTLSE : 2063
Ise : EEDELQENDSEFLQSGTMQYQTRDRSSQEQGLFGISQFIWDPGDPLFFLFKDQPFGSVFSHRELFADEEMSKGLLTLTSQTDPPKSIYKRWFSKNMQEKHFELLINRQRWLRTNSSLSNESFRSNTLSE : 2047
Ath : -------NDSEFLQSGTMQYQRRDRSSKEQGFFRISQFIWDPADPLFFLFKDQPFVSVFSHREFFADEEMSKG--LLTSQTDPPTSIYKRWFIKNTQEKHFELLIQRQRWLRTNSSLSNGFFRSNTRSE : 2255

* 2340 * 2360
Ikl : SYQYLSNLFLSNGTLLDQMTKTLLRKRWLFPDEMKIGFM : 2094
Ili : SYQYLSNLFLSNGTLLDQMTKTLLRKRWLFPDEMKIGFM : 2111
Isc : -----------------------LRKRWLFPDEMKIGFM : 1023
Ici : SYQYLSNLFLSNGTLLDQMTKTLLRKRWLFPDEMKIGFM : 2093
Ipe : SYQYLSNLFLSNGTLLDQMTKTLLRKRWLFPDEMKIGFM : 2102
Ise : SYQYLSNLFLSNGTLLDQMTKTLLRKRWLFPDEMKIGFM : 2086
Ath : SYQYLSNLFISNGTLLDRMTKTLLKKRWLFSDEMKIGFM : 2294

**Figure S72 Multiple sequence alignment of the deduced amino acid sequences of YCF2 protein.** The origins of the protein sequences used in this alignment are from: *Arabidopsis thaliana* (Ath), *Iodes klaineana* (Ikl), *Iodes cirrhosa* (Ici), *Iodes seretii* (Ise), *Iodes scandens* (Isc), *Iodes perrieri* (Ipe) and *Iodes liberica* (Ili). The amino acid numbers for each sequence are indicated on the right. In the sequence alignment, identical residues are shown with a black background, and similar residues are shown with a gray background.

* 20 * 40 * 60 * 80 * 100 * 120 *
Ipe : MPRSRINANFIDKTFSIVANILLRIIPTTSGEKEAFTYYRDGAIMSAQSEGNYAEALQNYYEAMRLEIDPYDRSYILYNIGLIHTSNGEHTKALEYYFRALERNPFLPQAFNNMAVICHYRGEQAIRQGDSE : 132
Ise : MPRSRINENFIDKTFSIVANILLRIIPTTSGEKEAFTYYRDGAI-------------------------PYDRSYILYNIGLIHTSNGEHTKALEYYFRALERNPFLPQAFNNMAVICHYRGEQAIRQGDSE : 107
Ikl : MPRSRINANFIDKTFSIVANILLRIIPTTSGEKEAFTYYRDGMSAQ--SEGNYAEALQNYYEAMRLEIDPYDRSYILYNIGLIHTSNGEHTKALEYYFRALERNPFLPQAFNNMAVICHYRGEQAIRQGDSE : 130
Isc : MSRSRINANFIDKTFSIVANILLRIMPTTAGEKEAFTYYRDGAIMSAQSEGNYAEALQNYYEAMRLEIDPYDRSYILYNIGLIHTSNGEHTKALEYYFRALERNPFLPQAFNNMAVICHY-------QGDSE : 125
Ici : MPRSRINANFIDKTFSIVANILLRIIPTTSGEKEAFTYYRDGGMSAQ-SEGNYAEALQNYYEAMRLEIDPYDRSYILYNIGLIHTSNGEHTKALEYYFRALERNPFLPQAFNNMAVICHYRGEQAIRQGDSE : 131
Ath : MPRSRINGNFIDKTFTIVADILLRVIPTTSGEKEAFTYYRDGMSAQ--SEGNYAEALQNYYEAMRLEIDPYDRSYILYNIGLIHTSNGEHTKALEYYFRALERNPFLPQAFNNMAVICHYRGEQAIQQGDSE : 130
Ili : MPRSRINANFIDKTFSIVANILLRIIPTTSGEKEAFTYYRDGAIMSAQSEGNYAEALQNYYEAMRLETDPYDRNYILFNIGLIHTSN-ERTYELEYYFRALERNPFLP---HLIIWICHYRGEQAIRQGDSE : 128

 140 * 160 *
Ipe : IAEAWFDQAAEYWKQAIALTPGNYIEAHNWLKITRRFE : 170
Ise : IAEAWFDQAAEYWKQAIALTPGNYIEAHNWLKITRRFE : 145
Ikl : IAEAWFDQAAEYWKQAIALTPGNYIEAHNWLKITRRFE : 168
Isc : IAEAWFDQAAEYWKQAIALTPGNYIEAHNWLKITRRFE : 163
Ici : IAEAWFDQAAEYWKQAIALTPGNYIEAHNWLKITRRFE : 169
Ath : MAEAWFAQAAEYWKQAITLTPGNYIEAQNWLTITRRFE : 168
Ili : IAEAWFDQAAEYWKQAIALTPGNYIEAHNWL------- : 159

**Figure S73 Multiple sequence alignment of the deduced amino acid sequences of YCF3 protein.** The origins of the protein sequences used in this alignment are from: *Arabidopsis thaliana* (Ath), *Iodes klaineana* (Ikl), *Iodes cirrhosa* (Ici), *Iodes seretii* (Ise), *Iodes scandens* (Isc), *Iodes perrieri* (Ipe) and *Iodes liberica* (Ili). The amino acid numbers for each sequence are indicated on the right. In the sequence alignment, identical residues are shown with a black background, and similar residues are shown with a gray background.

* 20 * 40 * 60 * 80 * 100 * 120 *
Ikl : MSWRSEHIWIELIRGSRKTSNFCWAFILLFGSLGFLLVGTSSYLGRNLISFFPCQQIIFFPQGLVMSFYGIAGLFISSYLWCTISWNVGSGYDRFDRKKGIVCIFRWGFPGKNRRIFLRFFIKDIQSIRIEV : 132
Ipe : MSWRSEHIWIELIRGSRKTSNFCWAFILLFGSLGFLLVGTSSYLGRNLISFFPCQQIIFFPQGLVMSFYGIAGLFISSYLWCTISWNVGSGYDRFDRKKGIVCIFRWGFPGKNRRIFLRFFIKDIQSIRIEV : 132
Ili : MSWRSEHLWIELIRGSRKTSNFCWAFILLFGSLGFLLVGTSSYLGRNLISFFPCQQIIFFPQGLVMSFYGIAGLFISSYLWCTISWNVGSGYDRFDRKKGIVCIFRWGFPGKNRRIFLRFFIKDIQSIRIEV : 132
Isc : MSWRSEHIWIELIRGSRKTSNFCWAFILLFGSLGFLLVGTSSYLGRNLISFFPCPQIIFFPQGLVMSFYGIAGLFISSYLWCTISWNVGSGYDRFDRKKGIVCIFRWGFPGKNRRIFLRFFIKDIQSIRIEV : 132
Ise : MSWRSEHIWIELIRGSRKTSNFCWAFILLFGSLGFLLVGTSSYLGRNLISFFPCQQIIFFPQGLVMSFYGIAGLFISSYLWCTISWNVGSGYDRFDRKKGIVCIFRWGFPGKNRRIFLRFFIKDIQSIRIEV : 132
Ici : MSWRSEHIWIELIKGSRKTSNFCWAFILLFGSLGFLLVGTSSYLGRNLISFFPCQQIIFFPQGLVMSFYGIAGLFISSYLWCTISWNVGSGYDRFDRKKGIVCIFRWGFPGKNRRIFLRFFIKDIQSIRIEV : 132
Ath : MSWRSESIWIEFITGSRKTSNFCWAFILFLGSLGFLLVGTSSYLGRNVISLFPSQQIIFFPQGIVMSFYGIAGLFISCYLWCTILWNVGSGYDLFDRKEGIVRIFRWGFPGKSRRIFLRFFMKDIQSIRIEV : 132

 140 * 160 * 180
Ikl : KDGISARRVLYMDIRGQGAVPLTRTDENLTPREIEQKAAELAYFLRIPIEVF : 184
Ipe : KAGISARRVLYMAIRGQGAVPLTRTDENLTPREIEQKAAELAYFLRIPIEVF : 184
Ili : KDGISARRVLYMDIRGQGAVPLTRTDENFTPREIEQKAAELAYFLRIPIEVF : 184
Isc : KDGISARRVLYMAIRGQGAVPLTRTDENFTPREIEQKAAELAYFLRIPIEVF : 184
Ise : KDGISARRVLYMAIKGQGAVPLSRTDENFTPREIEQKAAELAYFLRIPIEVF : 184
Ici : KDGISARRVLYMAIRGQGAVPLTRTDENFTPREIEQKAAELAYFLRIPIEVF : 184
Ath : KEGVSARRVLYMEIRGQGAIPLIRTDENFTTREIEQKAAELAYFLRVPIEVF : 184

**Figure S74 Multiple sequence alignment of the deduced amino acid sequences of YCF4 protein.** The origins of the protein sequences used in this alignment are from: *Arabidopsis thaliana* (Ath), *Iodes klaineana* (Ikl), *Iodes cirrhosa* (Ici), *Iodes seretii* (Ise), *Iodes scandens* (Isc), *Iodes perrieri* (Ipe) and *Iodes liberica* (Ili). The amino acid numbers for each sequence are indicated on the right. In the sequence alignment, identical residues are shown with a black background, and similar residues are shown with a gray background.

* 20 * 40 * 60 * 80 * 100 * 120 *
Previous study: --------------QHDFLYPLIFQESTYILAHNHG-----------------DNKSSLLIVKRLITRMYQQN---------NQNLFFGRNKNLYSKIISEGFAVIVEIPFSLR--------------TLR : 77
Our study: MEEFKKKLRIDRFQQHDFLYPLIFQESTYILAHNHGL-----NRSILLENRGYDNKSSLLIVKRLITRMYQQNHLIPFANDSNQNQFFGRNKNLYSKIISEGFAVIVEIPFSLRLTSFLEGKDIIQSHTLR : 126
*A. thaliana*: MDKFQGYLEFDGARQQSFLYPLFFREYIYVLAYDHGLNRLNRNRYIFLENADYDKKYSSLITKRLILRMYEQNRLIIPTKDVNQNSFLGHTSLFYYQMISVLFAVIVEIPFSLRLGSSFQGKQLKKSYNLQ : 131

 140 * 160 * 180 * 200 * 220 * 240 * 260
Previous study: SIHSIFPFLEDNFLHLNYALDIRIPHPIHLEILVQTIRYWVKDASSLHLLRFFLHEY--------------------HRFFFFLYNSYVCEYESVFVFLCNQSSHLRSTSSGTFIERIYFYVKIECVVEV- : 187
Our study: SIHSIFPFLEDNFLHLNYALDIRIPHPIHLEILVQTIRYWVKDASSLHLLRFFLHEYGDLNSPITSKKVSYSFSKRNHRFFFFLYNSYVCEYESVFVFLCNQSSHLRSTSSGTFIERIYFYVKIECVVEVF : 257
*A. thaliana*: SIHSIFPFLEDKLGHFNYVLDVLIPYPIHLEILVQTLRYRVKDASSLHFFRFCLYEYCNWKNFYIKKK-----SILNPRFFLFLYNSHVCEYESIFFFLRKRSSHLRSTSYEVLFERIVFYGKIHHFFKVF : 257

 * 280 * 300 * 320 * 340 * 360 * 380 *
Previous study: -KVSQANLWLFKDPFMYYVRYQGKSILASKGT-LLMNKWKYYLVNFCQSYFDLWSNPGRIHINRLSKHSLDFMGYLSSVQLNSSIIRSQMLENSFLVDNAIKKFDTILPIITLIGSLAKAKFCNTLGHPIS : 316
Our study: SKVSQANLWLFKDPFMYYVRYQGKSILASKGTFLLMNKWKYYLVNFCQSYFDLWSNPGRIHINRLSKHSLDFMGYLSSVQLNSSIIRSQMLENSFLVDNAIKKFDTILPIITLIGSLAKAKFCNTLGHPIS : 388
*A. thaliana*: VNNFPAILGLLKDPFIHYVRYHGRCILATKDTPLLMNKWKYYFVNLWQCYFSVWFQSQKVNINQLSKDNLEFLGYLSSLRLNPLVVRSQMLENSFLIDNVRIKLDSKIPISSIIGSLAKDKFCNVLGHPIS : 388

 400 * 420 * 440 * 460 * 480 * 500
Previous study: KPVRAHLSDSDIIDRFGRICRNLFHYYSGSSTKKRLYRIKYILRLSCARTLARKHKSTVRAF-----PEFLEKFLTAEGEVLSLTFPIVSS-LRGLYKRRIWYLDIICIN------ : 420
Our study: KPVRAHLSDSDIIDRFGRICRNLFHYYSGSSTKKRLYRIKYILRLSCARTLARKHKSTVRAFLK---PEFLEKFLTAEGEVLSLTFPIVSSILRGLYKRRIWYLDIICINDLANYE : 501
*A. thaliana*: KATWTDSSDSDILNRFVRICRNISHYYSGSSKKKNLYRIKYILRLCCVKTLARKHKSTVRTFLKRLGSGLLEEFLTGEDQVLSLIFPRSYYASKRLYRVRIWYLDILYLNDLVNHE : 504

**Figure S75 The alignment of the MATK protein.** The origin of the three sequences is from *Iodes cirrhosa* in our study and in previous study by Stull, and *Arabidopsis thaliana*. The regions that are significantly improved are shown in red square.

* 20 * 40 * 60 * 80 * 100 * 120 *
Our study: MEEFKKYLRIDKSQQHDFLYPLIFQESTYILAHDHGL-----NRSILLENGGYGKKFSLLIVKRLITRMYQQNHLIPFANDSNQNLFFGRNKNLYSQIISEGFAVIVEIPFSLRLTSFLEGKDIIQSHTLR : 126
Previous study: --------------QHDFLYPLIFQESTYILAHDHG-----------------GKKFSLLIVKRLITRMYQQN---------NQNLFFGRNKNLYSQIISEGFAVIVEIPFSLR--------------TLR : 77
*A. thaliana*: MDKFQGYLEFDGARQQSFLYPLFFREYIYVLAYDHGLNRLNRNRYIFLENADYDKKYSSLITKRLILRMYEQNRLIIPTKDVNQNSFLGHTSLFYYQMISVLFAVIVEIPFSLRLGSSFQGKQLKKSYNLQ : 131

 140 * 160 * 180 * 200 * 220 * 240 * 260
Our study: SIHSIFPFLEDNFLHLNYALDILIPHPIHLEILVQTIRYWVKDASSLHLLRFFLHEYGNLTSTITSKKVSYSFSKGNHRFFFFLYNSYICEYESVFVFLCNQSSHLRSTSSGAFIERIYFYLKMECVVEVF : 257
Previous study: SIHSIFPFLEDNFLHLNYALDILIPHPIHLEILVQTIRYWVKDASSLHLLRFFLHEY--------------------HRFFFFLYNSYICEYESVFVFLCNQSSHLRSTSSGAFIERIYFYLKMECVVEV- : 187
*A. thaliana*: SIHSIFPFLEDKLGHFNYVLDVLIPYPIHLEILVQTLRYRVKDASSLHFFRFCLYEYCNWKNFYIKKK-----SILNPRFFLFLYNSHVCEYESIFFFLRKRSSHLRSTSYEVLFERIVFYGKIHHFFKVF : 257

 * 280 * 300 * 320 * 340 * 360 * 380 *
Our study: SKVSQANLWLFKDPFMYYVRYQGKSILASKGTFLLMKKWKYYLVNFCQSYFDLWSNPGRIYVNQLCKHSLDFMGYLSSVQLNSSIIRSQMLENSFLVDNAIKKFDTILPIITLIGSLAKAKFCNTLGHPIS : 388
Previous study: -KVSQANLWLFKDPFMYYVRYQGKSILASKGT-LLMKKWKYYLVNFCQSYFDLWSNPGRIYVNQLCKHSLDFMGYLSSVQLNSSIIRSQMLENSFLVDNAIKKFDTILPIITLIGSLAKAKFCNTLGHPIS : 316
*A. thaliana*: VNNFPAILGLLKDPFIHYVRYHGRCILATKDTPLLMNKWKYYFVNLWQCYFSVWFQSQKVNINQLSKDNLEFLGYLSSLRLNPLVVRSQMLENSFLIDNVRIKLDSKIPISSIIGSLAKDKFCNVLGHPIS : 388

 400 * 420 * 440 * 460 * 480 * 500
Our study: KPVRAHLSDSDIIDRFGRICRNLFHYYSGSSKKKRLYRIKYILRLSCARTLARKHKSTVRAFLK---PEFFEKFLTAEGEVLSLTFPIVSSILRGLYRRRIWYLDIICINDLANYE : 501
Previous study: KPVRAHLSDSDIIDRFGRICRNLFHYYSGSSKKKRLYRIKYILRXXXXXXXXXXXKSTVRAF-----PEFFEKFLTAEGEVLSLTFPIVSS-LRGLYRRRIWYLDIICIN------ : 420
*A. thaliana*: KATWTDSSDSDILNRFVRICRNISHYYSGSSKKKNLYRIKYILRLCCVKTLARKHKSTVRTFLKRLGSGLLEEFLTGEDQVLSLIFPRSYYASKRLYRVRIWYLDILYLNDLVNHE : 504

**Figure S76 The alignment of the MATK protein.** The origin of the three sequences is from *Iodes klaineana* in our study and in previous study by Stull, and *Arabidopsis thaliana*. The regions that are significantly improved are shown in red square.

* 20 * 40 * 60 * 80 * 100 * 120 *
Our study: MEEFKKYLRIDKSQQHDFLYPLIFQESTYILAHDHGL-----NRSILLENGGYGKKSSLLIVKRLITRMYQQNHLIPFANDSNQNLFFGRNKNLYSQIISEGFAVIVEIPFSL------------------ : 108
Previous study: --------------QHDFLYPLIFQESTYILAHDHG-----------------GKKSSLLIVKRLITRMYQQN---------NQNLFFGRNKNLYSQIISEGFAVIVEIPFSLRT--------------LR : 77
*A. thaliana*: MDKFQGYLEFDGARQQSFLYPLFFREYIYVLAYDHGLNRLNRNRYIFLENADYDKKYSSLITKRLILRMYEQNRLIIPTKDVNQNSFLGHTSLFYYQMISVLFAVIVEIPFSLRLGSSFQGKQLKKSYNLQ : 131

 140 * 160 * 180 * 200 * 220 * 240 * 260
Our study: ----------------------------------------------------------------------------------------------------------------------------------- : -
Previous study: SIHSIFPFLEDNFLHLNYALDILIPHPIHLEXXXXXXXXXXXXXXXXXXXXXXXXXXXXXXXXXXXXXXXXXXXXXXXXX---------------XXXXHLRSTSSGAFIERIYFYLKMECVVEVKVS--Q : 191
*A. thaliana*: SIHSIFPFLEDKLGHFNYVLDVLIPYPIHLEILVQTLRYRVKDASSLHFFRFCLYEYCNWKNFYIKKKSILNPRFFLFLYNSHVCEYESIFFFLRKRSSHLRSTSYEVLFERIVFYGKIHHFFKVFVNNFP : 262

 * 280 * 300 * 320 * 340 * 360 * 380 *
Our study: ----------MYYVRYQGKSILASKGTFLLMKKWKYYLVNFCQSYFDLWSNPGRIYVNRLCKHSLDFMGYLSSVQLNSSIIRSQMLENSFLVDNAIKKFDTILPIITLIGSLAKAKFCNTLGHPISKPVRA : 229
Previous study: ANLWLFKDPFMYYVRYQGKSILASKGT-LLMKKWKYYLVNFCQSYFDLWSNPGRIYVNRLCKHSLDFMGYLSSVQLNSSIIRSQMLENSFLVDNAIKKFDTILPIITLIGSLAKAKFCNTLGHPISKPVRA : 321
*A. thaliana*: AILGLLKDPFIHYVRYHGRCILATKDTPLLMNKWKYYFVNLWQCYFSVWFQSQKVNINQLSKDNLEFLGYLSSLRLNPLVVRSQMLENSFLIDNVRIKLDSKIPISSIIGSLAKDKFCNVLGHPISKATWT : 393

 400 * 420 * 440 * 460 * 480 * 500
Our study: HLSDSDIIDRFGRICRNLFHYYSGSSKKKRLYRIKYILRLSCARTLARKHKSTVRAFLK---PEFFEKFLTAEGEVLSLTFPIVSSILRGLYRRRIWYLDIICINDLANYE : 337
Previous study: HLSDSDIIDRFGRICRNLFHYYSGSSKKKRLYRIKYILRLSCARTLARKHKSTVRAF-----PEFFEKFLTAEGEVLSLTFPIVSS-LRGLYRRRIWYLDIICIN------ : 420
*A. thaliana*: DSSDSDILNRFVRICRNISHYYSGSSKKKNLYRIKYILRLCCVKTLARKHKSTVRTFLKRLGSGLLEEFLTGEDQVLSLIFPRSYYASKRLYRVRIWYLDILYLNDLVNHE : 504

**Figure S77 The alignment of the MATK protein.** The origin of the three sequences is from *Iodes liberica* in our study and in previous study by Stull, and *Arabidopsis thaliana*. The regions that are significantly improved are shown in red square.

* 20 * 40 * 60 * 80 * 100 * 120 *
Our study: MEEFKKYLRIDKSQQHDFLYPLIFQESTYILAHDHGL-----NRSILLENRGYEKKSSLLIVKRLITRMYQQNHLIPFANDSNQNLFFGRNKNLYLQIISEGFAVIVEIPFSLRLTSFLEGKDIIQSHTLR : 126
Previous study: --------------QHDFLYPLIFQESTYILAHDHG-----------------EKKSSLLIVKRLITRMYQQN---------NQNLFFGRNKNLYLQIISEGFAVIVEIPFSLR--------------TLR : 77
*A. thaliana*: MDKFQGYLEFDGARQQSFLYPLFFREYIYVLAYDHGLNRLNRNRYIFLENADYDKKYSSLITKRLILRMYEQNRLIIPTKDVNQNSFLGHTSLFYYQMISVLFAVIVEIPFSLRLGSSFQGKQLKKSYNLQ : 131

 140 * 160 * 180 * 200 * 220 * 240 * 260
Our study: SIHSIFPFLEDNFLHLNYALDILIPYPIHLEILVQTIRYWVKDASSLHLLRFFLHEYGNLASTITSKKVSYSFSKGRFFFFLYNSYVCEYESVFVFLCNQSSHLRSTSSGAFIERIYFYLKMECVVEVFSK : 257
Previous study: SIHSIFPFLEDNFLHLNYALDILIPYPIHLEILVQTIRYWVKDASSLHLLRFFLHEYX------------------RFFFFLYNSYVCEYESVFVFLCNQSSHLRSTSSGAFIERIYFYLKMECVVEV--K : 188
*A. thaliana*: SIHSIFPFLEDKLGHFNYVLDVLIPYPIHLEILVQTLRYRVKDASSLHFFRFCLYEYCNWKNFYIKKK---SILNPRFFLFLYNSHVCEYESIFFFLRKRSSHLRSTSYEVLFERIVFYGKIHHFFKVFVN : 259

 * 280 * 300 * 320 * 340 * 360 * 380 *
Our study: VSQANLWLFKDPFMYYVRYQGKSILASKGTFLLMKKWKYYLVNFCQSYFDLWSNPGRIYINRLCKHSLDFMGYLSSVQLNSSIIRSQMLENSFLVDNAIKKFDTILPIITLIGSLAKAKFCNTLGHPISKP : 388
Previous study: VSQANLWLFKDPFMYYVRYQGKSILASKGT-LLMKKWKYYLVNFCQSYFDLWSNPGRIYINRLCKHSLDFMGYLSSVQLNSSIIRSQMLENSFLVDNAIKKFDTILPIITLIGSLAKAKFCNTLGHPISKP : 318
*A. thaliana*: NFPAILGLLKDPFIHYVRYHGRCILATKDTPLLMNKWKYYFVNLWQCYFSVWFQSQKVNINQLSKDNLEFLGYLSSLRLNPLVVRSQMLENSFLIDNVRIKLDSKIPISSIIGSLAKDKFCNVLGHPISKA : 390

 400 * 420 * 440 * 460 * 480 * 500
Our study: VRAHLSDSDIIDRFGRLCRNLFHYYSGSSKKKRLYRIKYILRLSCARTLARKHKSTVRDFLK---PEFLEKFLTAEGEVLSLNFPIVSSILRGLYIRRIWYLDIICINDLANYE : 499
Previous study: VRAHLSDSDIIDRFGRLCRNLFHYYSGSSKKKRLYRIKYILRLSCARTLARKHKSTVRDF-----PEFLEKFLTAEGEVLSLNFPIVSS-LRGLYIRRIWYLDIICIN------ : 420
*A. thaliana*: TWTDSSDSDILNRFVRICRNISHYYSGSSKKKNLYRIKYILRLCCVKTLARKHKSTVRTFLKRLGSGLLEEFLTGEDQVLSLIFPRSYYASKRLYRVRIWYLDILYLNDLVNHE : 504

**Figure S78 The alignment of the MATK protein.** The origin of the three sequences is from *Iodes perrieri* in our study and in previous study by Stull, and *Arabidopsis thaliana*. The regions that are significantly improved are shown in red square.

* 20 * 40 * 60 * 80 * 100 * 120 *
Our study: --------------------------------------------------------------------------------------------------------------------MEEFKKYLRIDRSQQ : 15
Previous study: --------------QHDFLYPLIFQESTYILAHDRGDN-----------------KSSLLIVKRLITRMYQQN---------NQNLFFGRNQNXXXXXXXXXXXXXXXXXXXXXXXXXXXXXXXXXXXXXX : 91
*A. thaliana*: MDKFQGYLEFDGARQQSFLYPLFFREYIYVLAYDHGLNRLNRNRYIFLENADYDKKYSSLITKRLILRMYEQNRLIIPTKDVNQNSFLGHTSLFYYQMISVLFAVIVEIPFSLRLGSSFQGKQLKKSYNLQ : 131

 140 * 160 * 180 * 200 * 220 * 240 * 260
Our study: -------------------------HDFLYPLIFQESTYEYGNLNSPITSKKVSYSFSKRN-----------HRFFFFLYNSYVCEYESVFVFLCNQSSLLK----------------------------- : 81
Previous study: -------------------------XXXXXXXXXXXXXXXXXXXXXXXXXXXXXXXXXXXXXFFLHE----YHRFFFFLYNSYVCEYESVFVFLCNQSSHLRSTSSGAFIERIYFYVKIECVVEVXXXXXX : 193
*A. thaliana*: SIHSIFPFLEDKLGHFNYVLDVLIPYPIHLEILVQTLRYRVKDASSLHFFRFCLYEYCNWKNFYIKKKSILNPRFFLFLYNSHVCEYESIFFFLRKRSSHLRSTSYEVLFERIVFYGKIHHFFKVFVNNFP : 262

 * 280 * 300 * 320 * 340 * 360 * 380 *
Our study: -------DPFMYYVRYQGKSILASKGTFLLMNKWKYYLVNFCQSYFHLWSNPGRIYINRLCKHSLDFMGYLSSVQLNSSIIRSQMLENSFLVDNAIKKFDTILPIITLIGSLAKAKFCNTLGHPISKPVRA : 205
Previous study: XXX--XXXPFMYYVRYQGKSILASKGT-LLMNKWKYYLVNFCQSYFHLWSNPGRIYINRLCKHSLDFMGYLSSVQLNSSIIRSQMLENSFLVDNAIKKFDTILPIITLIGSLAKAKFCNTLGHPISKPVRA : 321
*A. thaliana*: AILGLLKDPFIHYVRYHGRCILATKDTPLLMNKWKYYFVNLWQCYFSVWFQSQKVNINQLSKDNLEFLGYLSSLRLNPLVVRSQMLENSFLIDNVRIKLDSKIPISSIIGSLAKDKFCNVLGHPISKATWT : 393

 400 * 420 * 440 * 460 * 480 * 500
Our study: HLSDSDIIDRFGRICRNLFHYYSGSSTKKRLYRIKYILRLSCARTLARKHKSTVRAFLK---PEFLEKFLTAEGEVLSLTFPIVSSILRGLYKRRIWYLDIIFINDPANYE : 313
Previous study: HLSDSDIIDRFGRICRNLFHYYSGSSTKKRLYRIKYILRLSCARTLARKHKSTVRAF-----PEFLEKFLTAEGEVLSLTFPIVSS-LRGLYKR----------------- : 409
*A. thaliana*: DSSDSDILNRFVRICRNISHYYSGSSKKKNLYRIKYILRLCCVKTLARKHKSTVRTFLKRLGSGLLEEFLTGEDQVLSLIFPRSYYASKRLYRVRIWYLDILYLNDLVNHE : 504

**Figure S79 The alignment of the MATK protein.** The origin of the three sequences is from *Iodes scandens* in our study and in previous study by Stull, and *Arabidopsis thaliana*. The regions that are significantly improved are shown in red square.

* 20 * 40 * 60 * 80 * 100 * 120 *
Our study: MEEFKKYLRIDKSQQHDFLYPLIFQESTYILAHDHGL-----NRSILLENRGYDKK-------------SSQNHLIPFANDSNQNQFFGRNKNLYSQIISEGFAVIVEIPFSLRLTSFLEGKDIIQSHTLR : 113
Previous study: --------------QHDFLYPLIFQESTYILAHDHGD-----KKSSLLIVKXXXXX-------------XXX--------QNNQNQFFGRNKNLYSQIISEGFAVIVEIPFSLR--------------TLR : 77
*A. thaliana*: MDKFQGYLEFDGARQQSFLYPLFFREYIYVLAYDHGLNRLNRNRYIFLENADYDKKYSSLITKRLILRMYEQNRLIIPTKDVNQNSFLGHTSLFYYQMISVLFAVIVEIPFSLRLGSSFQGKQLKKSYNLQ : 131

 140 * 160 * 180 * 200 * 220 * 240 * 260
Our study: SIHSIFPFLEDNFLHLNYALDILIPHPIHLEILVQTIRYWVKDASSLHLLRFFLHEYGNLTSTISKG----NQRFFFFLYNSYVCQYESVFVFLCNQSSHLRSTSYGAFIERISFYLKMECVVEV------ : 234
Previous study: SIHSIFPFLEDNFLHLNYALDILIPHPIHLEILVQTIRYWVKDASSLHLLRFFLHEY---------------QRFFFFLYNSYVCQYESVFVFLCNQSSHLRSTSYGAFIERISFYLKMECVVEVKV--FQ : 191
*A. thaliana*: SIHSIFPFLEDKLGHFNYVLDVLIPYPIHLEILVQTLRYRVKDASSLHFFRFCLYEYCNWKNFYIKKKSILNPRFFLFLYNSHVCEYESIFFFLRKRSSHLRSTSYEVLFERIVFYGKIHHFFKVFVNNFP : 262

 * 280 * 300 * 320 * 340 * 360 * 380 *
Our study: --------------------------------------------------------FSRLCKHSLDFMGYLSSVQLNSSIIRSQMLENSFLVDNAITKFDTILPIITLIGSLAKAKFCNTLGHPISKPVRA : 309
Previous study: ANLWLFKDPFMYYVRYQGKSILASKGT-LLMKKWKYYLVNFCQSYFDLWSNPGRIYXXRLCKHSLDFMGYLSSVQLNSSIIRSQMLENSFLVDNAITKFDTILPIITLIGSLAKAKFCNTLGHPISKPVRA : 321
*A. thaliana*: AILGLLKDPFIHYVRYHGRCILATKDTPLLMNKWKYYFVNLWQCYFSVWFQSQKVNINQLSKDNLEFLGYLSSLRLNPLVVRSQMLENSFLIDNVRIKLDSKIPISSIIGSLAKDKFCNVLGHPISKATWT : 393

 400 * 420 * 440 * 460 * 480 * 500
Our study: HLSDSDIIDRFGRICRNLFHYYSGSSKKKRLYRIKYILRLSCARTLARKHKSTLRAFLK---PEFLEKFLTAEGEVLSLTFPIVSSILRGLYRRRIWYLDIICINDLANYE : 417
Previous study: HLSDSDIIDRFGRICRNLFHYYSGSSKKKRLYRIKYILRLSCARTLARKHKSTLRAF-----PEFLEKFLTAEGEVLSLTFPIVSS-LRGLYRRRIWYLDIICIN------ : 420
*A. thaliana*: DSSDSDILNRFVRICRNISHYYSGSSKKKNLYRIKYILRLCCVKTLARKHKSTVRTFLKRLGSGLLEEFLTGEDQVLSLIFPRSYYASKRLYRVRIWYLDILYLNDLVNHE : 504

**Figure S80 The alignment of the MATK protein.** The origin of the three sequences is from *Iodes seretii* in our study and in previous study by Stull, and *Arabidopsis thaliana*. The regions that are significantly improved are shown in red square.


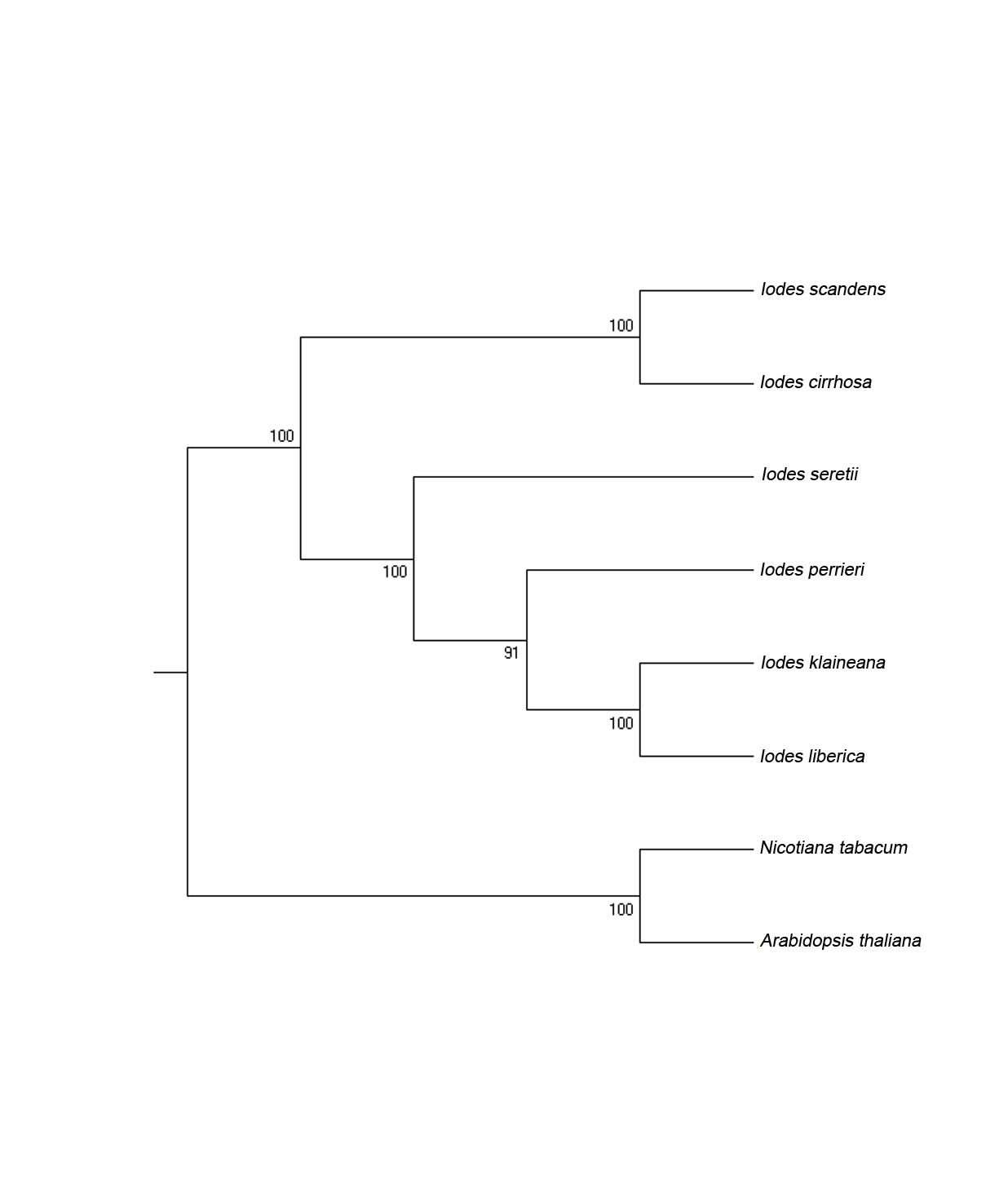


**Figure S81 Molecular phylogenetic analysis of 74 shared proteins sequences from 8 species.** Numbers above each node represent the bootstrap support values. The percentage of replicate trees in which the associated taxa clustered together in the bootstrap test (1000 replicates) is shown next to the corresponding branch. *Arabidopsis thaliana* and *Nicotiana tabacum* are as outgroups.

* 20 * 40 * 60 * 80
Ici : ATGCAAATAAATAGGGGGGAAGAAAACTCTATGGAAAGATGGTGGTTTAATTCGGTCTTGTTTAAGAAGGAGTTAAAACACAGGTGTGG : 89
Isc : ------------CGCTCTTCCGATCTGATCGTGGAAAGATGGTGGTTTAATTCGGTCTTCTTTAAGAAGGAGTTAAAACACAGGTGTGG : 77
Ikl : ---------------GGGGAAGAAAACTCTATGGAAAGATGGTGGTTTAATTCGGTCTTGTTTAAGAAGGAGTTAAAACACAGGTGTGG : 74
Ipe : ---------------GGGGAAGAAAACTCTATGGAAAGATGGTGGTTTAATTCGGTCTTGTTTAAGAAGGAGTTAAAACACAGGTGTGG : 74
Ili : ---------------GGGGAAGAAAACTCTATGGAAAGATGGTGGTTTAATTCGGTCTTGTTTAAGAAGGAGTTAAAACACAGGTGTGG : 74
Ise : ---------------GGGGAAGAAAACTCTATGGAAAGATGGTGGTTTAATTCGGTCTTGTTTAAGAAGGAGTTAAAACACAGGTGTGG : 74
Ath : ------------------------------ATGGAAAAATCGTGGTTCAATTTTATGTTTTCTAAGGGAGAATTGGAATACAGAGGTGA : 59

 * 100 * 120 * 140 * 160 * 180
Ici : GCCAAGTAAATCAATGGATAGTCTTGGTCCTACTGAAAATACCAGTCAAAATGAAGATCCTAATATAAATGATACGGCTAAAAACATTC : 178
Isc : GCCAAGTAAATCAATGGATAGTCTTGGTCCTACTGAAAATACCAGTCAAAATGAAGATCCTAATATAAATGATACGGCTAAAAACATTC : 166
Ikl : GCCAAGTAAATCAATGGATAGTCTTGGTCCTACTGAAAATACCAGTCAAAATGAAGATCCTAATATAAATGATACGGCTAAAAACATTC : 163
Ipe : ACCAAGTAAATCAATGGATAGTCTTGGTCCTACTGAAAATACCAGTCAAAATGAAGATCCTAATATAAATGATACGGCTAAAAACATTC : 163
Ili : GCCAAGTAAATCAATGGATAGTCTTGGTCCTACTGAAAATACCAGTCAAAATGAAGATCCTAATATAAATGATACGGCTAAAAACATTC : 163
Ise : GCCAAGTAAATCAATGGATAGTCTTGGTCCTTCTGAAAATACCAGTCAAAATGAAGATCCTAATATAAATGATACGGCTAAAAACATTC : 163
Ath : GCTAAGTAAAGCAATGGATAGTTTTGCTCCTGGTGAAAAGACTACTATAAGTCAAGACCGTTTTATATATGATATGGATAAAAACTTTT : 148

 180 * 200 * 220 * 240 * 260
Ici : ATAGTTGGAGTGATCGT------------------------GGTAATAGCAATCTTGTAG---ACCTCAAAGACATTCGTAATTTAATC : 240
Isc : ATAGTTGGAGTCATCGT------------------------GGTAATAGCAATCTTGTAG---ACCTCAAAGACATTCGTAATTTAATC : 228
Ikl : ATAGTTGGAGTGATCGT------------------------GGTAATAGCAATCTTGTAG---ACCTCAAAGACATTCGTCATTTAATC : 225
Ipe : ATAGTTGGAGTGATCGT------------------------GGTAATAGCAATCTTGTAG---ACCTCAAAGACATTCGTAATTTAATC : 225
Ili : ATAGTTGGAGTGATCGT------------------------GGTAATAGCAATCTTGTAG---ACCTCAAAGACATTCGTAATTTAATC : 225
Ise : ATAGTTGGAGTGATCGT------------------------GGTAATAGCAATCTTGTAG---ACCTCCAAGACATTCGTAATTTAATC : 225
Ath : ATGGTTGGGATGAGCGTTCTAGTTATTCTTCTAGTTATTCCAATAATGTTGATCTTTTAGTTAGCTCCAAGGACATTCGCAATTTCATA : 237

* 280 * 300 * 320 * 340 *
Ici : TCTGATGACACTTTTTTAGTTAGGGATAGTAATGAAAATAGTTATTCCATATATTTTGATATTGAAAATCAGATTTTTGAGATTGACAA : 329
Isc : TCGGATGACACTTTTTTAGTTAGGGATAGTAATGAAAATAGTTATTCCATATATTTTGATATTGAAAATCAGATTTTTGAGATTGACAA : 317
Ikl : TCTGATGACACTTTTTTAGTTAGGGATAGTAATGAAAATAGTTATTCCATATATTTTGATATTGAAAATCAGATTTTTGAGATTGACAA : 314
Ipe : TCTGATGACACTTTTTTAGTTAGGGATAGTAATGAAAATAGTTATTCCATATATTTTGATATTGAAAATCAGATTTTTGAGATTGACAA : 314
Ili : TCTGATGACACTTTTTTAGTTAGGGATAGTAATGAAAATAGTTATTCCATATATTTTGATATTGAAAAT------TTTGAGATTGACAA : 308
Ise : TCTGATGACACTTTTTTAGTTAGGGATAGTAATGAAAATAGTTATTCCATATATTTTGATATTGAAAATCAGATTTTTGAGATTGACAA : 314
Ath : TCGGATGACACCTTTTTTGTTAGGGATAGTAATAAGAATAGTTATTCTATATTTTTTGATAAAAAAAAAAAAATTTTTGAGATTGACAA : 326

 360 * 380 * 400 * 420 * 440
Ici : TGATCATTCTTTTCTGAGTGAAGTAGAAAGTTCTTTTTATAATTATCGAAATTCTAGTTATCTGAATAATAGATCTAAGAGTG---ACG : 415
Isc : TGATCATTCTTTTCTGAGTGAAGTAGAAAGTTCTTTTTATAATTATCGAAATTCTAGTTATCTGAATAATAGATCTAAGAGTG---ACG : 403
Ikl : TGATCATTCTTTTCTGAGTGAAG---------------------------------------------------------------ACG : 340
Ipe : TGATCATTCTTTTCTGAGTGAAGTAGAAAGTTCTTTTTATAATTATCGAAATTCTAGTTATCTGAATAATAGATCTAAGAGTG---ACG : 400
Ili : TGATCATTCTTTTCTGAGTGAAGTAGAAAGTTCTTTTTATAATTATCGAAATTCTAGTTATCTGAATAATAGATCTAAGAGTG---ACG : 394
Ise : TGATCATTCTTTTCTGAGTGAAGTAGAAAGTTCTTTTTCTAATTATCGAAATTCTAGTTATCTGAATAATAGATCTAAGAGTG---ACG : 400
Ath : TGATTTT---------AGTGACCTAGAAAAATTTTTTTATAGTTATTGTAGTTCTAGTTATCTAAATAATAGATCTAAAGGTGACAACG : 406

 * 460 * 480 * 500 * 520 *
Ici : ATCTACACTATGATCGTTACATGTATAATACTCAATCTAGTTGGAATAATCACATTAATAGTTGCATTGACAGTTATCTTCAGTCTCAA : 504
Isc : ATCTACACTATGATCGTTACATGTATGATACTCAATCTAGTTGGAATAATCACATTAATAGTTGCATTGACAGTTATCTTCAGTCTCAA : 492
Ikl : ATCTACACTATGATCGTTACATGTATGATACTCAATCTAGCTGGAATAATCACATTAATAGTTGCATTGACAGTTATCTTCAGTCTCAA : 429
Ipe : ATCTACACTATGATCGTTACATGTATGATACTCAATCTAGTTGGAATAATCACATTAATAGTTGCATTGACAGTTATCTTCAGTCTCAA : 489
Ili : ATCTACACTATGATCGTTACATGTATGATACTCAATCTAGTTGGAATAATCACATTAATAGTTGCATTGACAGTTATCTTCAGTCTCAA : 483
Ise : ATCTACACTATGATCGTTACATGTATGATACTCAATCTAGTTGGAATAATCACATTAATAGTTGCATTGACAGTTATCT--AGATTTAA : 487
Ath : ATCTGCACTATGATCCTTACATTAAGGATACTAAATATAATTGTACTAATCACATTAATAGTTGCATTGATTCTTATTTTCGTTCTTAC : 495

540 * 560 * 580 * 600 * 620
Ici : ATCTCTATTGATACTTACATTGAAAGTGGCAGTGACAACTCCAGTGACAGTTACATTTATAGTTCCATTTGTGGTGAAAGTAGAAAGAG : 593
Isc : ATCTCTATTGATACTT------------------------------------------------------------------------- : 508
Ikl : ATCTCTATTGATACTTACATTGAAAGTGGCAGTGACAATTCCAGCGACAGTTACATTTATAGTTACATTTGTGGTGAAAGTGGAAAGAG : 518
Ipe : ATCTCTATTGATACTTACATTGAAAGTGGCAGTGACAATTCCAGCGACAGTTACATTTATAGTTACATTTGTGGTGAAAGTGGAAAGAG : 578
Ili : ATCTCTATTGATACTTACATTGAAAGTGGCAGTGACAATTCCAGCGACAGTTACATTTATAGTTACATTTGTGGTGAAAGTGGAAAGAG : 572
Ise : CTCTA-AGAGAAAGTT------------------------------------------------------------------------- : 502
Ath : ATCTGTATTGATAATAACTTTTTAATCGATAGTAATAATTTTAATGAAAGTTACATTTATAATTTCATTTGTAGTGAAAGCGGAAAGAT : 584

 * 640 * 660 * 680 * 700 *
Ici : TAGTGAAAGCGGGAGTTCTAGTATACGAACCAGCACAAGTGGTAGTGATTTAACTCTAAGAGAAAGTTCGAATGATCTCGATGGAGAAA : 682
Isc : ----------------------------------------------------------------------------------------- : -
Ikl : TAGTGAAAGTGGGAGTTCTAGTATACGAACCAGCACAAGTGGTAGTGATTTAACTCTAAGAGAAAGTTCTAATGATCTCGA-------- : 599
Ipe : TAGTGAAAGCGGGAGTTCTAGTATACGAACCAGCACAAGTGGTAGTGATTTAACTCTAAGAGAAAGTTCTAATGATCTCGA-------- : 659
Ili : TAGTGAAAGTGGGAGTTCGAGTATACGAACCAGCACAAGTGGTAGTGATTTAACTCTAAGAGAAAGTTCTAATGATCTCGA-------- : 653
Ise : --------------------------------------------------------------------CTAATGATCTCGA-------- : 515
Ath : TCGTGAAAGTAAAAATTACAAGATAAGAACTAATAGGAATCGTAGTAATTTAAT----------------------------------- : 638

 720 * 740 * 760 * 780 * 800
Ici : GTTCGAATGATTTCGATGGAGAAAGTTCGAATGATCTCGATGGGACTCAAAAATACAGGCATTTGTGGATTCAATGCGAAAATTGTTAT : 771
Isc : ----------------------------------------------------------------------------------------- : -
Ikl : ----------------TGGAGAAAGTTCTAATGATCTCGATGGGACTCAAAAATACAGGCATTTGTGGATTCAATGCGAAAATTGTTAT : 672
Ipe : ----------------TGGAGAAAGTTCTAATGATCTCGATGGGACTCAAAAATACAGGCATTTGTGGATTCAATGCGAAAATTGTTAT : 732
Ili : ----------------TGGAGAAAGTTCTAATGATCTCGATGGGACTCAAAAATACAGACATTTGTGGATTCAATGCGAAAATTGTTAT : 726
Ise : ----------------TGGAGAAAGTTCTAATGATCTCGATGGGACTCAAAAATACAGGCATTTGTGGATT------------------ : 570
Ath : ----------------------AAGTTCTAAGGATTTCGATATAACTCAAAACTACAATCAATTGTGGATTCAATGCGACAATTGTTAT : 705

* 820 * 840 * 860 * 880 *
Ici : GCATTAAATTATAAAAAAAATTTTAAATCCAAAATGAATATTTGTGAACAATGTGGATATCATTTGAAAATTAGTAGTTCAGATAGAAT : 860
Isc : ----------------------------------------------------------------------------------------- : -
Ikl : GCATTAAATTATAAAAAAATTTTTAAATCAAAAATGAATATTTGTGAACAATGTGGATATCATTTGAAAATTAGTAGTTCAGATAGAAT : 761
Ipe : GCATTAAATTATAAAAAAAATTTGAAATCAAAAATGAATATTTGTGAACAATGTGGATATCATTTGAAAATTAGCAGTTCAGATAGAAT : 821
Ili : GCATTAAATTATAAAA------------------------TTTGTGAACAATGTGGATATCATTTGAAAATTAGTAGTTCAGATAGAAT : 791
Ise : ----------------------------------------------------------------------------------------- : -
Ath : GGATTAATGTATAAGAAAGTC---------AAAATGAATGTTTGTGAACAATGTGGACATTATTTGAAAATGAGTAGTTCAGAAAGAAT : 785

 900 * 920 * 940 * 960 * 98
Ici : TGAACTTTCGATCGACCCAGGCACTTGGGATCCTATGGACGAAGACATGGTCTCTCTGGATCCCATTGAATTTCATTCGGAGGAGGAGT : 949
Isc : ----------------------------------------------------------------------------------------- : -
Ikl : TGAACTTTCGATCGACCCGGGCACTTGGGATCCTATGGACGAAGACATGGTCTCTCTGGATCCCATTGAATTTCATTCGGAGGAGGAGT : 850
Ipe : TGAACTTTCGATCGACCCGGGCACTTGGGATCCTATGGACGAAGACATGGTCTCTCTGGATCCCATTGAATTTCATTCGGAGGAGGAGT : 910
Ili : TGAACTTTCGATCGACCCGGGCACTTGGGATCCTATGGACGAAGACATGGTCTCTCTGGATCCCATTGAATTTCATTCGGAGGAGGAGT : 880
Ise : ----------------------------------------------------------------ATTGAATTTCATTCGGAGGAGGAGT : 595
Ath : CGAGCTTTCGATTGATCCGGGTACTTGGAATCCTATGGATGAAGACATGGTCTCTGCGGATCCCATTAAATTTCATTCGAAGGAGGAAC : 874

 980 * 1000 * 1020 * 1040 * 1060
Ici : CTTATAAAGACCGTATTGATTCTTATCAAAGAAAGACAGGATTAACCGAGGCTGTTCAAACAGGTATAGGCCAATTAAATGGTATTCCC : 1038
Isc : CTTATAAAGACCGCATTGATTCTTATCAAAGAAAGACAGGATTAACCGAGGCTGTTCAAACAGGCATAGGCCAATTAAATGGTATTCCC : 597
Ikl : CTTATAAAGACCGTATTGATTCTTATCAAAGAAAGACAGGATTAACCGAGGCTGTTCAAACAGGCATAGGCCAATTAAATGGTATTCCC : 939
Ipe : CTTATAAAGACCGTATTGATTCTTATCAAAGAAAGACAGGATTAACCGAAGCTGTTCAAACAGGCATAGGCCAATTAAATGGTATTCCC : 999
Ili : CTTATAAAGACCGTATTGATTCTTATCAAAGAAAGACAGGATTAACCGAGGCTGTTCAAACAGGCATAGGCCAATTAAATGGTATTCCC : 969
Ise : CTTATAAAGACCGTATTGATTCTTATCAAAGAAAGACAGGATTAACCGAGGCTGTTCAAACAGGCATAGGCCAATTAAATGGTATTCCC : 684
Ath : CTTATAAAAACCGTATTGACTCTGCGCAAAAAACTACAGGATTGACTGACGCTGTTCAAACAGGTACAGGTCAACTAAACGGTATTCCG : 963

* 1080 * 1100 * 1120 * 1140 * 180
Ici : ATAGCAATTGGGGTTATGGATTTTCAGTTTATGGGTGGGAGTATGGGATCTGTAGTTGGGGAGAAAATCACCCGTTTGATTGAATACGC : 1127
Isc : ATAGCAATTGGGGTTATGGATTTTCAGTTTATGGGTGGGAGTATGGGATCTGTAGTTGGGGAGAAAATCACCCGTTTGATTGAATACGC : 686
Ikl : ATAGCAATTGGGGTTATGGATTTTCAGTTTATGGGTGGGAGTATGGGATCTGTAGTTGGGGAGAAAATCACCCGTTTGATTGAATACGC : 1028
Ipe : ATAGCAATTGGGGTTATGGATTTTCAGTTTATGGGTGGTAGTATGGGATCTGTAGTTGGGGAGAAAATCACCCGTTTGATTGAATACGC : 1088
Ili : ATAGCAATTGGGGTTATGGATTTTCAGTTTATGGGTGGGAGTATGGGATCTGTAGTTGGGGAGAAAATCACCCGTTTGATTGAATACGC : 1058
Ise : ATAGCAATTGGGGTTATGGATTTTCAGTTTATGGGTGGGAGTATGGGATCTGTAGTTGGGGAGAAAATCACCCGTTTGATTGAATACGC : 773
Ath : GTAGCCCTTGGGGTTATGGATTTTCGGTTTATGGGGGGTAGTATGGGATCCGTAGTAGGCGAAAAAATAACTCGTTTGATCGAGTATGC : 1052

1160 * 1180 * 1200 * 1220 * 1240
Ici : TACCAATAAATGTCTACCTCTTGTCATAGTATGTGCTTCGGGGGGGGCACGCATGCAAGAAGGAAGTTTGAGTTTGATGCAAATGGCTA : 1216
Isc : TACCAATAAATGTCTACCTCTTGTCATAGTATGTGCTTCGGGGGGGGCACGCATGCAAGAAGGAAGTTTGAGTTTGATGCAAATGGCTA : 775
Ikl : TACCAATAAATGTCTACCTCTTGTCATAGTATGTGCTTCGGGGGGGGCACGCATGCAAGAAGGAAGTTTGAGTTTGATGCAAATGGCTA : 1117
Ipe : TACCAATAAATGTCTACCTCTTGTCATAGTATGTGCTTCGGGGGGGGCACGCATGCAAGAAGGAAGTTTGAGTTTGATGCAAATGGCTA : 1177
Ili : TACCAATAAATGTCTACCTCTTGTCATAGTATGTGCTTCGGGGGGGGCACGCATGCAAGAAGGAAGTTTGAGTTTGATGCAAATGGCTA : 1147
Ise : TACCAATAAATGTCTACCTCTTGTCATAGTATGTGCTTCGGGGGGGGCACGCATGCAAGAAGGAAGTTTGAGTTTGATGCAAATGGCTA : 862
Ath : TACCAATCAATGTTTACCTCTTATTTTAGTGTGTTCTTCCGGAGGAGCACGAATGCAAGAAGGAAGTTTAAGTTTGATGCAAATGGCTA : 1141

 * 1260 * 1280 * 1300 * 1320 *
Ici : AAATATCGTCTGCTTTATATGATTATCAATCAAATAAAAAGTTATTCTATGTATCAATCCTTACATCTCCTACGACGGGTGGGGTGACA : 1305
Isc : AAATATCGTCTGCTTTATATGATTATCAATCAAATAAAAAGTTATTCTATGTATCAATCCTTACATCTCCTACGACGGGTGGGGTGACA : 864
Ikl : AAATATCGTCTGCTTTATATGATTATCAATCAAATAAAAAGTTATTCTATGTATCAATCCTTACATCTCCTACGACGGGTGGGGTGACA : 1206
Ipe : AAATATCGTCTGCTTTATATGATTATCAATCAAATAAAAAGTTATTCTATGTATCAATCCTTACATCTCCTACGACGGGTGGGGTAACA : 1266
Ili : AAATATCGTCTGCTTTATATGATTATCAATCAAATAAAAAGTTATTCTATGTATCAATCCTTACATCTCCTACGACGGGTGGGGTGACA : 1236
Ise : AAATATCGTCTGCTTTATATGATTATCAATCAAATAAAAAGTTATTCTATGTATCAATCCTTACATCTCCTACGACGGGTGGGGTGACA : 951
Ath : AAATTTCTTCGGTTTTATGTGATTATCAATCAAGTAAAAAGTTATTCTATATATCAATTCTTACATCTCCTACTACCGGTGGAGTGACA : 1230

1340 * 1360 * 1380 * 1400 * 1420
Ici : GCGAGTTTTGGTATGTTGGGGGATATCATTATTGCCGAACCCAATGCCTACATTGCATTTGCGGGTAAAAGAGTAATTGAACAAACATT : 1394
Isc : GCGAGTTTTGGTATGTTGGGGGATATCATTATTGCCGAACCGAATACTCTACTT---TTTGCGGGTAAAAGAGTAATTGAACAAACATT : 950
Ikl : GCGAGTTTTGGTATGTTGGGGGATATCATTATTGCCGAACCCAATGCCTACATTGCATTTGCGGGTAAAAGAGTAATTGAACAAACATT : 1295
Ipe : GCGAGTTTTGGTATGTTGGGGGATATCATTATTGCCGAACCCAATGCCTACATTGCATTTGCGGGTAAAAGAGTAATTGAACAAACATT : 1355
Ili : GCGAGTTTTGGTATGTTGGGGGATATCATTATTGCCGAACCCAATGCCTACATTGCATTTGCGGGTAAAAGAGTAATTGAACAAACATT : 1325
Ise : GCGAGTTTTGGTATGTTGGGGGATATCATTATTGCCGAACCCAATGCCTACATTGCATTTGCGGGTAAAAGAGTAATTGAACAAACATT : 1040
Ath : GCTAGTTTTGGTATGTTGGGGGATATCATTATTGCCGAACCCTATGCCTATATTGCATTTGCGGGTAAAAGAGTAATTGAACAAACATT : 1319

 * 1440 * 1460 * 1480 * 1500 *
Ici : GAATAAAACAGTACCTGAAGGTTCACAGGAGGCTGAATATTTATTTCAGAAAGGCTTATTCGATCTAATCGTACCACGTAATCCTTTAA : 1483
Isc : GAATAAAACAGTACCTGAAGGTTCACAGGAGGCTGAATATTTATTCCAGAAAGGCTTATTCGATCTAATCGTACCACGTAATCCTTTAA : 1039
Ikl : GAATAAAACAGTACCTGAAGGTTCACAGGAGGCTGAATATTTATTCCAGAAGGGCTTATTCGATCTAATCGTACCACGTAATCCTTTAA : 1384
Ipe : GAATAAAACAGTACCTGAAGGTTCACAGGAGGCTGAATATTTATTCCAGAAGGGCTTATTCGATCTAATCGTACCACGTAATCCTTTAA : 1444
Ili : GAATAAAACAGTACCTGAAGGTTCACAGGAGGCTGAATATTTATTCCAGAAGGGCTTATTCGATCTAATCGTACCACGTAATCCTTTAA : 1414
Ise : GAATAAAACAGTACCTGAAGGTTCACAGGAGGCTGAATATTTATTCCAGAAGGGCTTATTCGATCTAATCGTACCACGTAATCCTTTAA : 1129
Ath : GAAAAAAGCCGTGCCTGAAGGTTCACAAGCGGCTGAATCTTTATTACGTAAGGGCTTATTGGATGCAATTGTACCACGTAATCTTTTAA : 1408

 1520 * 1540 * 1560 *
Ici : AAAACGTTTTGAGGGAGTTATTTCAACTCCACGCTTTCTTTCCTTTGAATAAAAATTAA : 1542
Isc : AAAACGTTTTGAAGGAGTTATTTCAACTCCACGCTTTCTTTCCTTTGAATAAAAAT--- : 1095
Ikl : AAAACGTTTTGAAAGAGTTATTTCAACTCCACGCTTTCTTTCCTTTGAATAAAAAT--- : 1440
Ipe : AAAACGTTTTGAAAGAGTTATTTCAACTCCACGCTTTCTTTCCTTTGAATAAAAAT--- : 1500
Ili : AAAACGTTTTGAAAGAGTTATTTCAACTCCACGCTTTCTTTCCT--------------- : 1458
Ise : AAAACGTTTTGAAAGAG------------------------------------------ : 1146
Ath : AAGGTGTTCTGAGCGAGTTATTTCAGCTCCATGCTTTTTTTCCTTTGAACACAAATTAA : 1467

**Figure S82 Multiple sequence alignment of the deduced nucleotide sequences of *acc*D gene.** The origins of the nucleotide sequences used in this alignment are from: *Arabidopsis thaliana* (Ath), *Iodes klaineana* (Ikl), *Iodes cirrhosa* (Ici), *Iodes seretii* (Ise), *Iodes scandens* (Isc), *Iodes perrieri* (Ipe) and *Iodes liberica* (Ili). The nucleotide numbers for each sequence are indicated on the right. In the sequence alignment, identical nucleotides are shown with a black background, and similar residues are shown with a gray background.

* 20 * 40 * 60 * 80
Ici : ATGGTAACCATTCGAGCCGACGAAATTAGTAATATTATCCGCGAACGGATTGAACAATATAATAGAGAGGTAAAGATTGTAAATACTGG : 89
Isc : ---------------------------------------------------------------------GTAAAGATTGTAAATACTGG : 20
Ipe : ATGGTAACCATTCGAGCCGACGAAATTAGTAATATTATCCGCGAACGGATTGAACAATATAATAGAGAGGTAAAGATTGTAAATACTGG : 89
Ise : ATGGTAACCATTCGAGCCGACGAAATTAGTAATATTATCCGCGAACGGATTGAACAATATAATAGAGAGGTAAAGATTGTAAATACTGG : 89
Ikl : ATGGTAACCATTCGAGCCGACGAAATTAGTAATATTATCCGCGAACGGATTGAACAATATAATTGAGAGGTAAAGATTGTAAATACTGG : 89
Ili : ATGGTAACCATTCGAGCCGACGAAATTAGTAATATTATCCGCGAACGGATTGAACAATATAATAGAGAGGTAAAGATTGTAAATACTGG : 89
Ath : ATGGTAACCATTAGAGCCGACGAAATTAGTAATATTATCCGTGAACGTATTGAGCAATATAATAGAGAAGTAACGATTGTAAATACCGG : 89

 * 100 * 120 * 140 * 160 * 180
Ici : TACCGTACTTCAAGTGGGCGATGGCATTGCTCGTATTCATGGTCTTGATGAAGTAATGGCGGGTGAATTAGTAGAATTTGAAGAGGGTA : 178
Isc : TACCGTACTTCAAGTGGGCGATGGCATTGCTCGTATTCATGGTCTTGATGAAGTAATGGCGGGTGAATTAGTAGAATTTGAAGAGGGTA : 109
Ipe : TACCGTACTTCAAGTGGGCGATGGCATTGCTCGTATTCATGGTCTTGATGAAGTAATGGCGGGTGAATTAGTAGAATTTGAAGAGGGTA : 178
Ise : TACCGTACTTCAAGTGGGCGATGGCATTGCTCGTATTCATGGTCTTGATGAAGTAATGGCGGGTGAATTAGTAGAATTTGAAGAGGGTA : 178
Ikl : TACCGTACTTCAAGTGGGCGATGGCATTGCTCGTATTCATGGTCTTGATGAAGTAATGGCGGGTGAATTAGTAGAATTTGAAGAGGGTA : 178
Ili : TACCGTACTTCAAGTGGGCGATGGCATTGCTCGTATTCATGGTCTTGATGAAGTAATGGCGGGTGAATTAGTAGAATTTGAAGAGGGTA : 178
Ath : TACCGTACTTCAAGTGGGCGATGGCATCGCTCGTATTTATGGTCTTGATGAAGTAATGGCAGGTGAATTAGTAGAATTTGAGGAGGGTA : 178

 180 * 200 * 220 * 240 * 260
Ici : CAATAGGCATTGCTCTAAATTTGGAATCAAATAATGTTGGTGTTGTATTAATGGGTGATGGTTTGATGATACAAGAAGGAAGTTCTGTG : 267
Isc : CAATAGGCATTGCTCTGAATTTGGAATCAAATAATGTTGGTGTTGTATTAATGGGTGATGGTTTGATGATACAAGAAGGAAGTTCTGTG : 198
Ipe : CAATAGGCATTGCTCTGAATTTGGAATCAAATAATGTTGGTGTTGTATTAATGGGTGATGGTTTGATGATACAAGAAGGAAGTTCTGTG : 267
Ise : CAATAGGCATTGCTCTGAATTTGGAATCAAATAATGTTGGTGTTGTATTAATGGGTGATGGTTTGATGATACAAGAAGGAAGTTCTGTG : 267
Ikl : CAATAGGCATTGCTCTGAATTTGGAATCAAATAATGTTGGTGTTGTATTAATGGGTGATGGTTTGATGATACAAGAAGGAAGTTCTGTG : 267
Ili : CAATAGGCATTGCTCTGAATTTGGAATCAAATAATGTTGGTGTTGTATTAATGGGTGATGGTTTGATGATACAAGAAGGAAGTTCTGTG : 267
Ath : CTATAGGTATTGCCCTTAATTTAGAATCCAATAATGTTGGTGTTGTATTAATGGGTGACGGTTTGATGATCCAAGAAGGAAGTTCAGTC : 267

* 280 * 300 * 320 * 340 *
Ici : AAAGCAACAGGAAGAATTGCTCAGATACCGGTGAGTGAAGCCTATTTGGGTCGTGTTCTAAATGCCTTGGCTAAACCTATTGATGGTAG : 356
Isc : AAAGCAACAGGAAGAATTGCTCAGATACCGGTGAGTGAAGCCTGTTTGGGTCGTGTTCTAAATGCCTTGGCTAAACCTATTGATGGTAG : 287
Ipe : AAAGCAACAGGAAGAATTGCTCAGATACCGGTGAGTGAAGCCTATTTGGGTCGTGTTCTAAATGCCTTGGCTAAACCTATTGATGGTAG : 356
Ise : AAAGCAACAGGAAGAATTGCTCAGATACCGGTGAGTGAAGCCTATTTGGGTCGTGTTCTAAATGCCTTGGCTAAACCTATTGATGGTAG : 356
Ikl : AAAGCAACAGGAAGAATTGCTCAGATACCGGTGAGTGAAGCCTATTTGGGCCGTGTTCTAAATGCCTTGGCTAAACCTATTGATGGTAG : 356
Ili : AAAGCAACAGGAAGAATTGCTCAGATACCGGTGAGTGAAGCCTATTTGGGCCGTGTTCTAAATGCCTTGGCTAAACCTATTGATGGTAG : 356
Ath : AAAGCTACGGGAAAAATTGCTCAGATACCGGTGAGTGAGGCTTATTTGGGGCGTGTTATAAACGCCTTGGCTAACCCTATTGATGGTCG : 356

 360 * 380 * 400 * 420 * 440
Ici : AGGTGAAATTTCAGCTTCTGAGTCTCGGTTAATAGAATCTCCCGCTCCGGGTATTATTTCGCGGCGTTCCGTATATGAGCCCCTTCAAA : 445
Isc : AGGTGAAATTTCAGCTTCTGAGTCTCGGTTAATAGAATCTCCCGCTCCGGGTATTATTTCGCGGCGTTCCGTATATGAGCCCCTTCAAA : 376
Ipe : AGGTGAAATTTCAGCTTCTGAGTCTAGGTTAATAGAATCTCCCGCTCCGGGTATTATTTCGCGGCGTTCCGTATATGAGCCTCTTCAAA : 445
Ise : AGGTGAAATTTCAGCTTCTGAGTCTAGGTTAATAGAATCTCCCGCTCCGGGTATTATTTCGCGGCGTTCCGTATATGAGCCTCTTCAAA : 445
Ikl : AGGTGAAATTTCAGCTTCTGAGTCTAGGTTAATAGAATCTCCCGCTCCGGGTATTATTTCGCGGCGTTCCGTATATGAGCCTCTTCAAA : 445
Ili : AGGTTAAATTTCAGCTTCTGAGTCTAGGTTAATAGAATCTCCCGCTCCGGGTATTATTTTGCGGCGTTCCGTATATGAGCCTCTTCAAA : 445
Ath : AGGTAAGATTTCCGCTTCTGAATCTCGGTTAATTGAATCTCCTGCCCCAGGTATTATTTCGAGACGTTCTGTATATGAGCCTCTTCAAA : 445

 * 460 * 480 * 500 * 520 *
Ici : CAGGGCTTATTGCTATTGATTCGATGATACCTATAGGACGTGGTCAACGAGAATTAATTATTGGGGACAGACAGACTGGTAAAACAGCA : 534
Isc : CAGGGCTTATTGCTATTGATTCGATGATACCTATAGGACGTGGTCAACGAGAATTAATTATTGGGGACAGACAGACTGGTAAAACAGCA : 465
Ipe : CAGGGCTTATTGCTATTGATTCGATGATACCTATAGGACGTGGTCAACGAGAATTAATTATTGGGGATAGACAGACTGGTAAAACAGCA : 534
Ise : CAGGGCTTATTGCTATTGATTCGATGATACCTATAGGACGTGGTCAACGAGAATTAATTATTGGGGACAGACAGACTGGTAAAACAGCA : 534
Ikl : CAGGGCTTATTGCTATTGATTCGATGATACCTATAGGACGTGGTCAGCGAGAATTAATTATTGGGGACAGACAGACTGGTAAAACAGCA : 534
Ili : CAGGGCTTATTGCTATTGATTCGATGATACCTATAGGACGTGGTCAGCGAGAATTAATTATTGGGGACAGACAGACTGGTAAAACAGCA : 534
Ath : CAGGACTTATTGCTATTGATTCCATGATCCCTATAGGACGCGGCCAGCGAGAATTAATTATTGGTGACAGACAGACCGGTAAAACAGCA : 534

540 * 560 * 580 * 600 * 620
Ici : GTAGCCACAGATACGATTCTCAATCAACAAGGTCAAAATGTAATATGTGTTTATGTGGCTATTGGTCAAAAAGCATCTTCTGTGGCTCA : 623
Isc : GTAGCCACAGATACGATTCTCAATCAACAAGGTCAAAATGTAATATGTGTTTATGTGGCTATTGGTCAAAAAGCATCTTCTGTGGCTCA : 554
Ipe : GTAGCCACAGATACAATTCTCAATCAACAAGGTCAAAATGTAATATGTGTTTATGTGGCTATTGGTCAAAAAGCATCTTCTGTGGCTCA : 623
Ise : GTAGCCACAGATACGATTCTCAATCAACAAGGTCAAAATGTAATATGTGTTTATGTGGCTATTGGTCAAAAAGCATCTTCTGTGGCTCA : 623
Ikl : GTAGCCACAGATACGATTCTCAATCAACAAGGTCAAAATGTAATATGTGTTTATGTGGCTATTGGTCAAAAAGCATCTTCTGTGGCTCA : 623
Ili : GTAGCCACAGATACGATTCTCAATCAACAAGGTCAAAATGTAATATGTGTTTATGTGGCTATTGGTCAAAAAGCATCTTCTGTGGCTCA : 623
Ath : GTAGCCACAGATACAATTCTCAATCAACAAGGCCAAAATGTAATATGTGTTTATGTAGCTATTGGTCAAAAAGCTTCTTCCGTGGCTCA : 623

 * 640 * 660 * 680 * 700 *
Ici : GGTAGTGACCACGTTACAGGAAAGGGGAGCGATGGAATACACTATTGTGGTAGCCGAAACAGCGGATTCCCCTGCTACATTACAATATC : 712
Isc : GGTAGTGACCACGTTACAGGAAAGGGGAGCTATGGAATACACTATTGTGGTAGCCGAAACAGCGGATTCCCCTGCTACATTACAATATC : 643
Ipe : GGTAGTGACCACGTTACAGGAAAGGGGAGCGATGGAATACACTATTGTGGTAGCCGAAACAGCGGATTCCCCTGCTACATTACAATATC : 712
Ise : GGTAGTGACCACGTTACAGGAAAGGGGAGCGATGGAATACACTATTGTGGTAGCCGAAACAGCGGATTCCCCTGCTACATTACAATATC : 712
Ikl : GGTAGTGACCACGTTACAGGAAAGGGGAGCGATGGAATACACTATTGTGGTAGCCGAAACAGCGGATTCCCCTGCTACATTACAATATC : 712
Ili : GGTAGTGACCACGTTACAGGAAAGGGGAGCGATGGAATACACTATTGTGGTAGCCGAAACAGCGGATTCCCCTGCTACATTACAATATC : 712
Ath : GGTAGTGACCAGTTTACAGGAACGAGGGGCAATGGAATACACTATTGTGGTAGCTGAAACGGCCGATTCCCCAGCTACGTTACAATACC : 712

 720 * 740 * 760 * 780 * 800
Ici : TTGCTCCTTATACAGGAGCAGCCCTGGCTGAATATTTTATGTACCGTGAACGACACACTTCAATCATTTATGATGATCCCTCCAAACAA : 801
Isc : TTGCTCCTTATACAGGAGCAGCCCTGGCTGAATATTTTATGTACCGTGAACGACACACTTCAATCATTTATGATGATCCCTCCAAACAA : 732
Ipe : TTGCTCCTTATACAGGAGCAGCCCTGGCTGAATATTTTATGTACCGTGAACGACACACTTCAATCATTTATGATGATCTCTCCAAACAA : 801
Ise : TTGCTCCTTATACAGGAGCAGCCCTGGCTGAATCTTTTATGTACCGTGAACGACACACTTCAATCATTTATGATGATCTCTCCAAACAA : 801
Ikl : TTGCTCCTTATACAGGAGCAGCCCTGGCTGAATATTTTATGTACCGTGAACGACACACTTCAATCATTTATGATGATCTCTCCAAACAA : 801
Ili : TTGCTCCTTATACAGGAGCAGCCCTGGCTGAATATTTTATGTACCGTGAACGACACACTTCAATCATTTATGATGATCTCTCCAAACAA : 801
Ath : TCGCGCCTTATACAGGAGCAGCCTTGGCTGAATATTTTATGTACCGTGAACAACACACTTTAATAATTTATGATGATCTTTCCAAACAA : 801

* 820 * 840 * 860 * 880 *
Ici : GCCCAAGCTTATCGCCAAATGTCTCTTCTACTACGAAGACCTCCCGGTCGTGAAGCTTACCCAGGGGATGTTTTTTATTTGCACTCACG : 890
Isc : GCACAAGCTTATCGCCAAATGTCTCTTCTACTACGAAGACCTCCTGGTCGTGAAGCTTACCCGGGGGATGTTTTTTATTTGCACTCACG : 821
Ipe : GCACAAGCTTATCGCCAAATGTCTCTTCTACTACGAAGACCTCCTGGTCGTGAAGCTTACCCAGGGGATGTTTTTTATTTGCACTCACG : 890
Ise : GCACAAGCTTATCGCCAAATGTCTCTTCTACTACGAAGACCTCCTGGTCATGAAGCTTACCCAGGGGATGTTTTTTATTTGCACTCACG : 890
Ikl : GCACAAGCTTATCGCCAAATGTCTCTTCTACTACGAAGACCTCCTGGTCGTGAAGCTTACCCAGGGGATGTTTTTTATTTGCACTCACG : 890
Ili : GCACAAGCTTATCGCCAAATGTCTCTTCTACTACGAAGACCTCCTGGTCGTGAAGCTTACCCAGGGGATGTTTTTTATTTGCACTCACG : 890
Ath : GCACAAGCTTATCGACAAATGTCTCTTCTATTACGAAGACCGCCGGGTCGTGAAGCTTATCCAGGAGATGTTTTTTATTTACATTCACG : 890

 900 * 920 * 940 * 960 * 98
Ici : CCTTTTGGAAAGAGCCGCTAAATCAAGTTCTAGTTTAGGTGAAGGAAGTATGACTGCTTTACCAATAGTTGAAACTCAATCGGGAGATG : 979
Isc : CCTTTTGGAAAGAGCCGCTAAATCAAGCTCTAGTTTAGGTGAAGGAAGTATGACTGCTTTACCAATAGTTGAAACTCAATCGGGAGATG : 910
Ipe : CCTTTTGGAAAGAGCCGCTAAATCAAGTTCTAGTTTAGGTGAAGGAAGTATGACTGCTTTACCAATAGTTGAAACTCAATCGGGAGATG : 979
Ise : CCTTTTGGAAAGAGCCGCTAAATCAAGTTCTAGTTTAGGTGAAGGAAGTATGACTGCTTTACCAATAGTTGAAACTCAATCGGGAGATG : 979
Ikl : CCTTTTGGAAAGAGCCGCTAAATCAAGTTCTAGTTTAGGTGAAGGAAGTATGACTGCTTTACCAATAGTTGAAACTCAATCGGGAGATG : 979
Ili : CCTTTTGGAAAGAGCCGCTAAATCAAGTTCTAGTTTAGGTGAAGGAAGTATGACTGCTTTACCAATAGTTGAAACTCAATCGGGAGATG : 979
Ath : TCTTTTAGAAAGAGCCGCTAAATTAAGCTCTCAATTAGGTGAAGGGAGTATGACTGCCTTACCAATCGTCGAGACCCAGTCAGGAGATG : 979

 980 * 1000 * 1020 * 1040 * 1060
Ici : TTTCGGCTTATATTCCTACTAATGTAATTTCTATTACTGATGGCCAAATATTCTTATCCGCCGATCTATTCAATGCTGGAATCAGACCA : 1068
Isc : TTTCGGCTTATATTCCTACTAATGTAATTTCTATTACTGATGGCCAAATATTCTTATCCGCCGATCTATTCAATGCTGGAATCAGACCA : 999
Ipe : TTTCGGCTTATATTCCTACTAATGTAATTTCTATTACTGATGGCCAAATATTTTTATCCGCCGATCTATTCAATGCTGGAATCAGACCA : 1068
Ise : TTTCGGCTTATATTCCTACTAATGTAATTTCTATTACTGATGGCCAAATATTTTTATCCGCCGATCTATTCAATGCTGGAATCAGACCA : 1068
Ikl : TTTCGGCTTATATTCCTACTAATGTAATTTCTATTACTGATGGCCAAATATTTTTATCCGCCGATCTATTCAATGCTGGAAGCAGACCA : 1068
Ili : TTTCGGCTTATATTCCTACTAATGTAATTTCTATTACTGATGGCCAAATATTTTTATCCGCCGATCTATTCAATGCTGGAATCAGACCA : 1068
Ath : TTTCAGCTTATATTCCTACTAATGTAATTTCCATTACAGATGGACAAATATTCTTATCCGCCGATCTTTTTAATGCTGGAATCAGACCT : 1068

* 1080 * 1100 * 1120 * 1140 * 1160
Ici : GCTATTAATGTGGGTATCTCAGTTTCGAGAGTTGGATCTGCGGCTCAAATTAAAGCCATGAAACAAGTAGCCGGCAAATTAAAATTGGA : 1157
Isc : GCTATTAATGTGGGTATCTCAGTTTCCAGAGTTGGATCTGCGGCTCAAATTAAAGCCATGAAACAAGTAGCCGGCAAATTAAAATTGGA : 1088
Ipe : GCTATTAATGTGGGTATCTCAGTTTCCAGAGTTGGATCTGCGGCTCAAATTAAAGCCATGAAACAAGTAGCCGGCAAATTAAAATTGGA : 1157
Ise : GCTATTAAT------------------AGAGTTGGATCTGCAGCTCAAATTAAAGCCATGAAACAAGTAGCCGGCAAATTAAAATTGGA : 1139
Ikl : GCCATTAATGTGGGTATCTCAGTTTCCAGAGTTGGATCTGCGGCTCAAATTAAAGCCATGAAACAAGTAGCCGGCAAATTAAAATTGGA : 1157
Ili : GCCATTAATGTGGGTATCTCAGTTTCCAGAGTTGGATCTGCGGCTCAAATTAAAGCCATGAAACAAGTAGCCGGCAAATTCAAATTGGA : 1157
Ath : GCTATTAATGTAGGGATTTCTGTCTCGAGAGTAGGATCCGCCGCTCAAATTAAAGCTATGAAACAGGTAGCTGGAAAATTAAAATTGGA : 1157

 1160 * 1180 * 1200 * 1220 * 1240
Ici : ACTGGCGCAATTCGCAGAATTAGAAGCT----TTTGCA--------CAATTTGCTTCGGATCTCGATAAAACTACTCAGAATCAATTGG : 1234
Isc : ACTGGCGCAATTCGCAGAATTAGAAGCT----TTTGCA--------CAATTTGCTTCGGATCTCGATAAAGCTACTCAGAATCAATTGG : 1165
Ipe : ACTGGCGCAATTCGCAGAATTAGAAGCT----TTTGCA--------CAATTTGCTTCGGATCTCGATAAAGCTACTCAGAATCAATTGG : 1234
Ise : ACTGGCGCAATTCGCAGAATTAGAAGCT----TTTGCA--------CAATTTGCTTCGGATCTCGATAAAGCTACTCAGAATCAATTGG : 1216
Ikl : ACTGGCGCAATTCGCAGAATTAGAAGCT----TTTGCA--------CAATTTGCTTCGGATCTCGATAAAGCTACTCAGAATCAATTGG : 1234
Ili : ACTGGCCTAATTCATAGAATTAGAAGGCACAATTTGCTTCGGATCTCAATTTGCTTCGGATCTCGATAAAGCTACTCAGAATCAATTGG : 1246
Ath : ATTGGCTCAATTCGCTGAATTAGAAGCC----TTTTCC--------CAATTTTCTTCTGATCTCGATAAAGCTACTCAGAATCAATTGG : 1234

 * 1260 * 1280 * 1300 * 1320 *
Ici : CAAGAGGTCAACGATTACGCGAATTACTCAAACAATCCCAATCCGCACCTCTCGCGGTAGAAGAACAGATAATGACTATTTATACCGGA : 1323
Isc : CAAGAGGTCAACGATTACGTGAATTACTCAAACAATCCCAATCCGCACCTCTCGCGGTAGAAGAACAGATAATGACTATTTATACCGGA : 1254
Ipe : CAAGAGGTCAACGATTACGTGAATTACTCAAACAATCCCAATCCGCACCTCTCGCGGTAGAAGAACAGATAATGACTATTTATACCGGA : 1323
Ise : CAAGAGGTCAACGATTACGTGAATTACTCAAACAATCCCAATCCGCACCTCTCGCGGTAGAAGAACAGATAATGACTATTTATACCGGA : 1305
Ikl : CAAGAGGTCAACGATTACGTGAATTACTCAAACAATCCCAATCCGCACCTCTCGCGGTAGAAGAACAGATAATGACTATTTATACCGGA : 1323
Ili : CAAGAGGTCAACGATTACGTGAATTACTCAAACAATCCCAATCCGCACCTCTCGCGGTAGAAGAACAGATAATTCCTATTTATACCGGG : 1335
Ath : CAAGAGGTCAACGATTGCGTGAGTTACTGAAACAATCCCAATCAGCCCCCCTCACAGTGGAAGAACAGATAATGACCATTTATACCGGA : 1323

1340 * 1360 * 1380 * 1400 * 1420
Ici : ACAAATGGTTATCTTGATTCATTAGAAATTGGACAGGTAAGAAAATTTCTTGTTGAGTTACGTACTTACTTAAAAAGTAATAAACCCGA : 1412
Isc : ACAAATGGTTATCTTGATTCATTAGAAATCGGACAGGTAAGAAAATTTCTTGTTGAGTTACGTACTTACTTAAAAAGTAATAAACCCGA : 1343
Ipe : ACAAATGGTTATCTTGATTCATTAGATATTGGACAGGTAAGAAAATTTCTTGTTGAGTTACGTACTTACTTAAAAAGTAATAAACCCGA : 1412
Ise : ACAAATGGTTATCTTGATTCATTAGAAATTGGACAGGTAAGAAAATTTCTTGTTGAGTTACGTACTTACTTAAAAAGTAATAAACCCGA : 1394
Ikl : ACAAATGGTTATCTGGATTCATTAGAAATTGGACAGGTAAGAAAATTTCTTGTTGAGTTACGTACTTACTTA----------------- : 1395
Ili : ACAAATGGTTATCTGGATTCATTAGAAATTGGACAGGTAAGAAAATTTCTTGTTGAGTTACGTACTTACTTAAAAAGTAATAAACCCGA : 1424
Ath : ACAAATGGTTATCTGGATGGATTAGAAATTGGACAAGTAAGAAAATTTCTCGTTCAATTACGCACTTACTTAAAAACGAATAAACCTCA : 1412

 * 1440 * 1460 * 1480 * 1500 *
Ici : ATTCAAAAAAATCATATCTTCTACCAAGACATTCACCGAAGAAGCAGAAGCCATTTTGAAAGAAGCTATTCAGGAACAAATGGAACGCT : 1501
Isc : ATTCAAAAAAATCATATCTTCTACCAAGACATTCACCGAAGAAGCAGAAACCATTTTGAAAGAAGCTATTCAGGAACAAATGGAACGCT : 1432
Ipe : ATTCAAAAAAATCATATCTTCTACCAAGACATTCACCGAAGAAGCAGAAGCCATTTTGAAAGAAGCTATTCAGGAACAAATGGAACGCT : 1501
Ise : ATTCAAAAAAATCATATCTTCTACCAAGACATTCACCGAAGAAGCAGAAGCCATTTTGAAAGAAGCTATTCAGGAACAAATGGAACGCT : 1483
Ikl : ----------------------------------------------------------------------------------------- : -
Ili : ATTCAAAAAAATAATATCTTCTACCAAGACATTCACC---------------------------------------------------- : 1461
Ath : GTTTCAAGAAATCATAGCCTCTACCAAGACATTAACCGCTGAAGCAGAAAGCTTTTTGAAAGAAGGTATTCAAGAGCAACTAGAACGTT : 1501

1520 *
Ici : TTAGACTTCAGGAACAACTATAA : 1524
Isc : TTAGACTTCAGGAACAACTA--- : 1452
Ipe : TTAGACTTCAGGAACAACTC--- : 1521
Ise : TTAGACTTCAGGAACAA------ : 1500
Ikl : ----------------------- : -
Ili : ----------------------- : -
Ath : TCCTACTTCAGGAGAAAGTATAA : 1524
**Figure S83 Multiple sequence alignment of the deduced nucleotide sequences of *atp*A gene.** The origins of the nucleotide sequences used in this alignment are from: *Arabidopsis thaliana* (Ath), *Iodes klaineana* (Ikl), *Iodes cirrhosa* (Ici), *Iodes seretii* (Ise), *Iodes scandens* (Isc), *Iodes perrieri* (Ipe) and *Iodes liberica* (Ili). The nucleotide numbers for each sequence are indicated on the right. In the sequence alignment, identical nucleotides are shown with a black background, and similar residues are shown with a gray background.

* 20 * 40 * 60 * 80
Ikl : ATGGGAACAAATCCTACTACTTCTGGTTCTGGGATTTCCA---------AAAAAAACCTTGGACGTATTGCACAAATCATTGGTCCGGT : 80
Ili : ATGGGAACAAATCCTACTACTTCTGGTTCTGGGATTTCCACGCTTGAAAAAAAAAACCTTGGACGTATTGCACAAATCATTGGTCCGGT : 89
Ise : ATGGGAACAAATCCTACTACTTCTGGTTCTGGGATTTCCACGCTTGACAAAAAAAACCTTGGACGTATTGCACAAATCATTGGTCCGGT : 89
Ici : ATGGGAACAAATCCTACTACTTCTGGTTCTGGGATTTCCACGCTTGACAAAAAAAACCTTGGACGTATTGCACAAATCATTGGTCCGGT : 89
Isc : ATGGGAACAAATCCTACTACTTCTGGTTCTGGGATTTCCACGCTTGACAAAAAAAACCTTGGACGTATTGCACAAATCATTGGTCCGGT : 89
Ipe : ------------------------------------------CTTGACAAAAAAAACCTTGGACGTATTGCACAAATCATTGGTCCGGT : 47
Ath : ATGAGAACAAATCCTACTACTTCAAATCCAGAGGTTTCGATACGTGAAAAAAAAAACCTGGGACGTATCGCCCAAATCATTGGTCCGGT : 89

 * 100 * 120 * 140 * 160 * 180
Ikl : ACTCGACGTAGCTTTTGCACCAGGCAAGATGCCTAATATTTATAACGCTCTGGTAGTTAAGGGTCGAGATACTGCTGGTCAACCAATTA : 169
Ili : ACTCGACGTAACTTTTGCACCAGTCAAGATGCCTAATATTTATAACGCTCTGGTAGTTAAGGGTCGAGATACTGCTGGTCAACCAATTA : 178
Ise : ACTCGATGTAGCTTTTGCACCAGGCAAGATGCCTAATATTTATAACGCTCTGGTAGTTAAGGGTCGAGATACTGCTGGTCAACCAATTA : 178
Ici : ACTCGATGTAGCTTTTGCACGGGGCAAGATGCCTAATATTTATAACGCTCTGGTAGTTAAGGGTCGAGATACTGCTGGTCAACCAATTA : 178
Isc : ACTCGATGTAGCTTTTGCGCCGGGCAAGATGCCTAATATTTATAACGCTCTGGTAGTTAAGGGTCGAGATACTGCTGGTCAACCAATTA : 178
Ipe : ACTCGATGTAGCTTTTGCACCAGGCAAGATGCCTAATATTTATAACGCTCTGGTAGTTAAGGGTCGAGATACTGCTGGTCAACCAATTA : 136
Ath : ACTGGATGTAGCCTTCCCCCCGGGCAAAATGCCTAATATTTACAATGCTCTGGTGGTTAAGGGTCGAGATACTCTTGGTCAAGAAATTA : 178

 180 * 200 * 220 * 240 * 260
Ikl : ATGTGACTTGTGAGGTACAGCAATTATTAGGAAACAATCGCGTTAGAGCTGTGGCTATGAGTGCTACAGATGGTCTAACGAGAGGAATG : 258
Ili : ATGTGACTTGTGAGGTACAGCAATTATTAGGAAACAATCGCGTTAGAGCTGTGGCTATGAGTGCTACAGATGGTCTAACGAGAGGAATG : 267
Ise : ATGTGACTTGTGAGGTACAGCAATTATTAGGAAACAATCGAGTTAGAGCTGTGGCTATGAGTGCTACAGATGGTCTAACAAGAGGAATG : 267
Ici : ATGTGACTTGTGAGGTACAGCAATTATTAGGAAACAATCAAGTTAGAGCTGTGGCTATGAGTGCTACAGATGGTCTAACGAGAGGAATG : 267
Isc : ATGTGACTTGTGAGGTACAGCAATTATTAGGAAACAATCGAGTTAGAGCTGTGGCTATGAGTGCTACAGATGGTCTAACGAGAGGAATG : 267
Ipe : ATGTGACTTGTGAGGTACAGCAATTATTAGGAAACAATCGAGTTAGAGCTGTGGCTATGAGTGCTACAGAGGGTCTAACGAGAGGAATG : 225
Ath : ATGTGACTTGTGAAGTACAGCAATTATTAGGAAATAATCGAGTTAGAGCTGTAGCTATGAGTGCGACAGAGGGTTTAAAGAGAGGAATG : 267

* 280 * 300 * 320 * 340 *
Ikl : GAAGTGATTGACACGGGAGCTCCTCTAAGTGTTCCGGTCGGTGGGGTGACTCTTGGACGAATTTTCAATGTGCTCGGAGAACCTGTTGA : 347
Ili : GAAGTGATTGACACGGGAGCTCCTCTAAGTGTTCCGGTCGGTGGGGTGACTCTTGGACGAATTTTCAATGTGCTCGGAGAACCTGTTGA : 356
Ise : GAAGTGATTGACACGGGGGCTCCTCTAAGTGTTCCGGTCGGTGGGGTGACTCTTGGACGAATTTTCAATGTGCTCGGAGAGCCTGTTGA : 356
Ici : GAAGTGATTGACACGGGAGCTCCTCTAAGTGTTCCGGTCGGTGGGGCGACTCTTGGACGAATTTTCAATGTGCTTGGAGAGCCTGTTGA : 356
Isc : GAAGTGATTGACACAGGAGCTCCTCTAAGTGTTCCGGTCGGCGGGGCGACTCTTGGACGAATTTTCAATGTGCTTGGAGAGCCTGTTGA : 356
Ipe : GAAGTGATTGACACGGGAGCTCCTCTAAGTGTTCCGGTCGGTGGGGTGACTCTTGGACGAATTTTCAATGTGCTCGGAGAGCCTGTTGA : 314
Ath : GACGTGGTTGATATGGGAAATCCTCTAAGTGTTCCAGTCGGCGGAGCGACTCTAGGACGAATTTTCAACGTGCTTGGGGAACCCGTTGA : 356

 360 * 380 * 400 * 420 * 440
Ikl : TAATTTAGGTCCTGTAGATACTCGTACAACATCTCCTATTCATAGATCAGCGCCCGCCTTTATACAGTTAGATACAAAATTATCTATTT : 436
Ili : TAATTTAGGTCCTGTAGATACTCGTACAACATCTCCTATTCATAGATCAGCGCCCGCCTTTATACAGTTAGATACAAAATTATCTATTT : 445
Ise : TAATTTAGGTCCTGTAGATACTCGTACAACATCTCCTATTCATAGATCTGCGCCCGCCTTTATACAGTTAGATACAAAATTATCTATTT : 445
Ici : TAATTTAGGTCCTGTAGATACTCGTACAACATCTCCTATTCATAGATCTGCGCCCGCCTTTATACAGTTAGATACAAAATTATCTATTT : 445
Isc : TAATTTAGGTCCTGTAGATACTCGTACAACATCTCCTATTCATAGATCTGCGCCCGCCTTTATACAGTTAGATACAAAATTATCTATTT : 445
Ipe : TAATTTAGGTCCTGTAGATACTCGTACAACATCTCCTATTCATAGATCTGCGCCCACCTTTATACAGTTAGATACAAAATTATCTATTT : 403
Ath : TAATTTAGGTCCTGTCGATACTCGCACAACATCTCCTATCCATAAATCCGCGCCTGCTTTTATAGAATTAGATACAAAATTATCGATTT : 445

 * 460 * 480 * 500 * 520 *
Ikl : TTGAAACAGGAATTAAGGTAGTAGATCTTTTAGCCCCCTATCGACGTGGAGGAAAAATCGGACTATTCGGGGGGGCTGGAGTGGGTAAA : 525
Ili : TTGAAACAGGAATTAAGGTAGTAGATCTTTTAGCCCCCTATCGACGTGGAGGAAAAATCGGACTATTCGGGGGGGCTGGAGTGGGTAAA : 534
Ise : TTGAAACAGGAATTAAGGTAGTAGATCTTTTAGCCCCCTATCGACGTGGAGGAAAAATCGGACTATTCGGGGGGGCTGGAGTGGGTAAA : 534
Ici : TTGAAACAGGAATTAAGGTAGTAGATCTTTTAGCCCCCTATCGACGTGGAGGAAAAATCGGACTATTCGGGGGGGCTGGGGTGGGTAAA : 534
Isc : TTGAAACAGGAATTAAGGTAGTAGATCTTTTAGCCCCCTATCGACGTGGAGGAAAAATCGGACTATTCGGGGGGGCTGGAGTGGGTAAA : 534
Ipe : TTGAAACAGGAATTAAGGTAGTAGATCTTTTAGCCCCCTATCGACGTGGAGGAAAAATCGGACTATTCGGGGGGGCTGGAGTGGGTAAA : 492
Ath : TTGAAACAGGAATTAAAGTAGTAGATCTTTTGGCCCCTTATCGTCGTGGGGGAAAAATTGGACTATTCGGTGGGGCTGGCGTGGGTAAA : 534

540 * 560 * 580 * 600 * 620
Ikl : ACAGTACTCATTATGGAATTGATTAACAATATTGCCAAAGCTCACGGGGGTGTATCCGTATTTGGCGGAGTGGGTGAACGTACTCGTGA : 614
Ili : ACAGTACTCATTATGGAATTGATTAACAATATTGCCAAAGCTCACGGGGGTGTATCCGTATTTGGCGGAGTGGGTGAACGTACTCGTGA : 623
Ise : ACAGTACTCATTATGGAATTGATTAACAATATTGCCAAAGCTCACGGGGGTGTATCCGTATTTGGCGGAGTGGGTGAACGTACTCGTGA : 623
Ici : ACAGTACTCATTATGGAATTGATTAATAATATTGCCAAAGCTCACGGGGGTGTATCCGTATTTGGAGGAGTGGGTGAACGTACTCGTGA : 623
Isc : ACAGTACTCATTATGGAATTGATTAACAATATTGCCAAAGCTCACGGGGGTGTATCCGTATTTGGAGGAGTGGGTGAACGTACTCGTGA : 623
Ipe : ACAGTACTCATTATGGAATTGATTAACAATATTGCCAAAGCTCACGGGGGTGTATCCGTATTTGGCGGAGTGGGTGAACGTACTCGTGA : 581
Ath : ACAGTACTAATTATGGAATTGATCAACAACATTGCTAAAGCTCATGGTGGTGTATCCGTATTTGGTGGAGTAGGCGAACGGACTCGTGA : 623

 * 640 * 660 * 680 * 700 *
Ikl : AGGAAATGATCTTTACATGGAAATGAAAGAATCTGGAGTGATTAATGAACAAAATATTGCAGAATCAAAAGTGGCTCTAGTTTACGGTC : 703
Ili : AGGAAATGATCTTTACATGGAAATGAAAGAATCTGGAGTGATTAATGAACAAAATATTGCAGAATCAAAAGTAGCTCTAGTTTACGGTC : 712
Ise : AGGAAATGATCTTTACATGGAAATGAAAGAATCTGGAGTGATTAATGAACAAAATATTGCAGAATCAAAAGTGGCTCTAGTTTACGGTC : 712
Ici : AGGAAATGATCTTTACATGGAAATGAAAGAATCTGGAGTGATTAATGAACAAAATATTGCAGAATCAAAAGTGGCTCTAGTTTACGGTC : 712
Isc : AGGAAATGATCTTTACATGGAAATGAAAGAATCTGGAGTGATTAATGAACAAAATATTGCAGAATCAAAAGTGGCTCTAGTTTACGGTC : 712
Ipe : AGGAAATGATCTTTACATGGAAATGAAAGAATCTGGAGTGATTAATGAACAAAATATTGCAGAATCAAAAGTGGCTCTAGTTTACGGTC : 670
Ath : AGGAAATGATCTTTACATGGAAATGAAAGAATCTGGAGTCATTAATGAACAAAATCTTGCGGAATCCAAAGTGGCCCTAGTTTATGGTC : 712

 720 * 740 * 760 * 780 * 800
Ikl : AGATGAATGAACCGCCAGGAGCTCGTATGAGAGTTGGTTTGACTGCCCTAACTATGGCGGAATATTTCCGCGATGTTAATGAACAAGAC : 792
Ili : AGATGAATGAACCGCCAGGAGCTCGTATGAGAGTTGGTTTGACTGCCCTAACTATGGCGGAATATTTCCGCGATGTTAATGAACAAGAC : 801
Ise : AGATGAATGAACCGCCAGGAGCTCGTATGAGAGTTGGTTTGACTGCCCTAACTATGGCGGAATATTTCCGCGATGTTAATGAACAAGAC : 801
Ici : AGATGAATGAACCGCCAGGAGCTCGTATGAGAGTTGGTTTGACTGCCCTAACTATGGCGGAATATTTCCGCGATGTTAATGAACAAGAC : 801
Isc : AGATGAATGAACCGCCAGGAGCTCGTATGAGAGTTGGTTTGACTGCCCTAACTATGGCGGAATATTTCCGCGATGTTAATGAACAAGAC : 801
Ipe : AAATGAATGAACCGCCAGGAGCTCGTATGAGAGTTGGTTTGACTGCCCTAACTATGGCGGAATATTTCCGCGATGTTAATGAGCAAGAC : 759
Ath : AGATGAATGAACCGCCAGGAGCTCGTATGAGAGTTGGTCTGACTGCCTTAACTATGGCAGAATATTTCCGAGATGTTAATGAGCAAGAC : 801

* 820 * 840 * 860 * 880 *
Ikl : GTACTTTTATTTATCGACAATATTTTCCGTTTCGTTCAAGCAGGATCCGAAGTATCTGCCTTATTGGGTAGAATGCCTTCCGCCGTAGG : 881
Ili : GTACTTTTATTTATCGACAATATTTTCCGTTTCGTTCAAGCAGGATCCGAAGTATCTGCCTTATTGGGTAGAATGCCTTCCGCCGTAGG : 890
Ise : GTACTTTTTTTTATCGACTCGATTTTCCGTTTCGTTCAAGCAGGATCCGAAGTATCTGCCCTATTGGGTAGAATGCCTTCCGCCGTAGG : 890
Ici : GTACTTTTATTTATCGACAATATTTTCCGTTTCGTCCAAGCAGGATCCGAAGTATCTGCCTTATTGGGTAGAATGCCTTCCGCCGTAGG : 890
Isc : GTACTTTTATTTATCGACAATATTTTCCGTTTCGTCCAAGCAGGATCCGAAGTATCTGCCTTATTGGGTAGAATGCCTTCCGCCGTAGG : 890
Ipe : GTACTTTTATTTATCGACAATATTTTCCGTTTCGTTCAAGCAGGATCCGAAGTATCTGCCTTATTGGGTAGAATGCCTTCCGCCGTAGG : 848
Ath : GTACTTCTATTTATCGACAATATCTTCCGTTTCGTACAAGCAGGATCCGAGGTATCCGCCTTATTGGGTAGAATGCCTTCTGCTGTGGG : 890

 900 * 920 * 940 * 960 * 98
Ikl : TTATCAACCCACCCTGAGTACCGAAATGGGTTCTTTACAAGAAAGAATTACTTCTACCAAAGACGGGTCCATAACTTCTATTCAAGCAG : 970
Ili : TTATCAACCCACCCTGAGTACCGAAATGGGTTCTTTACAAGAAAGAATTACTTCTACCAAAGAAGGGTCCATAACTTCTATTCAAGCAG : 979
Ise : TTATCAACCCACCCTGAGTACCGAAATGGGTTCTTTACAAGAAAGAATTACTTCTACCAAAGAAGGGTCCATAACTTCTATTCAAGCAG : 979
Ici : TTATCAACCCACCCTGAGTACCGAAATGGGTTCTTTACAAGAAAGAATTACTTCTACCAAAGAAGGGTCCATAACTTCTATTCAAGCAG : 979
Isc : TTATCAACCCACCCTGAGTACCGAAATGGGTTCTTTACAAGAAAGAATTACTTCTACCAAAGAAGGGTCCATAACTTCTATTCAAGCAG : 979
Ipe : TTATCAACCCACCCTGAGTACCGAAATGGGTTCTTTACAAGAAAGAATTACTTCTACCAAAGAAGGGTCCATAACTTCTATTCAAGCAG : 937
Ath : TTATCAACCCACCCTTAGTACCGAAATGGGTACTTTACAAGAAAGAATTACTTCTACGAAAAAGGGGTCCATAACCTCTATTCAAGCAG : 979

 980 * 1000 * 1020 * 1040 * 1060
Ikl : TTTATGTACCTGCAGACGATTTGACCGACCCTGCTCCTGCTACGACATTTGCACATTTAGATGCGACTACCGTACTATCAAGAGGATTA : 1059
Ili : TTTATGTACCTGCAGACGATTTGACCGACCCTGCTCCTGCTACGACATTTGCACATTTAGATGCGACTACCGTACTATCAAGAGGATTA : 1068
Ise : TTTATGTACCTGCAGACGATTTGACCGACCCTGCTCCTGCTACGACATTTGCACATTTAGATGCGACTACCGTACTATCAAGAGGATTA : 1068
Ici : TTTATGTACCTGCAGACGATTTGACTGACCCTGCTCCTGCTACGACATTTGCACATTTAGATGCGACTACCGTACTATCAAGAGGATTA : 1068
Isc : TTTATGTACCTGCAGACGATTTGACTGACCCTGCTCCTGCTACGACATTTGCACATTTAGATGCGACTACCGTACTATCAAGAGGATTA : 1068
Ipe : TTTATGTACCTGCAGACGATTTGACCGACCCTGCTCCTGCTACGACATTTGCACATTTAGATGCGACTACCGTACTATCAAGAGGATTA : 1026
Ath : TTTATGTACCTGCAGATGATTTGACTGACCCCGCACCTGCCACCACATTTGCACATTTAGATGCGACTACCGTACTATCAAGAGGATTA : 1068

* 1080 * 1100 * 1120 * 1140 * 1160
Ikl : GCTGCCAAAGGTATCTATCCAGCAGTAGACCCTTTAGATTCAACGTCAACTATGCTTCAACCTAGGATCGTTGGTGAGGAACATTATGA : 1148
Ili : GCTGCCAAAGGTATCTATCCAGCAGTAGACCCTTTAGATTCAACGTCAACTATGCTTCAACCTAGGATCGTTGGTGAGGAACATTATGA : 1157
Ise : GCTGCCAAAGGTATCTATCCAGCAGTAGACCCTTTAGATTCAACGTCAACTATGCTTCAACCTAGGATCGTTGGCGAGGAACATTATGA : 1157
Ici : GCTGCCAAAGGTATCTATCCAGCAGTAGACCCTTTAGATTCAACGTCAACTATGCTTCAACCTAGGATCGTTGGTGAGGAACATTATGA : 1157
Isc : GCTGCCAAAGGTATCTATCCAGCAGTAGACCCTTTAGATTCAACGTCAACTATGCTTCAACCTAGGATCGTTGGTGAGGAACATTATGA : 1157
Ipe : GCTGCCAAAGGTATCTATCCAGCAGTAGATCCTTTAGATTCAACGTCAACTATGCTTCAACCTAGGATCGTTGGTGAGGAACATTATGA : 1115
Ath : GCTGCCAAAGGTATCTATCCAGCGGTAGATCCTTTAGATTCAACGTCAACTATGCTACAACCTCGAATCGTTGGCGAGGAACATTATGA : 1157
 1160 * 1180 * 1200 * 1220 * 1240
Ikl : AACTGCGCAAAGAGTTAAGCAAACTTTACAACGTTACAAAGAACTTCAGGACATTATAGCTATCCTTGGGTTGGACGAATTATCCGAAG : 1237
Ili : AACTGCGCAAAGAGTTAAGCAAACTTTACAACGTTACAAAGAACTTCAGGAAATTATAGCTATCCTTGGGTTGGACGAATTATCCGAAG : 1246
Ise : AACTGCGCAAAGAGTTAAGCAAACTTTACAACGTTACAAAGAACTTCAGGACATTATAGCTATCCTTGGGTTGGACGAATTATCCGAAG : 1246
Ici : AACTGCGCAAAGAGTTAAGCAAACTTTACAACGTTACAAAGAACTTCAGGACATTATAGCTATCCTTGGGTTGGACGAATTATCCGAAG : 1246
Isc : AACCGCGCAAAGAGTTAAGCAAACTTTACAACGTTACAAAGAACTTCAGGACATTATAGCTATCCTTGGGTTGGACGAATTATCCGAAG : 1246
Ipe : AATGGCGCAAAGAGTTAAGCAAACTTTACAACGTTACAAAGAACTTCAGGACATTATAGCTATCCTTGGGTTGGACGAATTATCCGAAG : 1204
Ath : AACTGCGCAACAAGTAAAACAAACTTTACAACGTTACAAGGAGCTTCAGGACATTATAGCTATCCTGGGGTTGGATGAATTATCCGAAG : 1246

 * 1260 * 1280 * 1300 * 1320 *
Ikl : AGGATCGTTTAACTGTAGCAAGAGCGCGAAAAATAGAACGTTTCTTATCACAACCCTTTTTTGTAGCAGAAGTCTTTACTGGTTCTCCA : 1326
Ili : AGGATCGTTTAACTGTAGCAAGAGCGCGAAAAATAGAACGTTTCTTATCACAACCCTTTTTTGTAGCAGAAGTATTTACTGGTTCTCCA : 1335
Ise : AGGATCGTTTAACCGTAGCAAGAGCGCGAAAAATAGAACGTTTCTTATCACAACCCTTTTTTGTAGCAGAAGTCTTTACTGGTTCTCCA : 1335
Ici : AGGATCGTTTAACCGTAGCAAGAGCGCGAAAAATAGAACGTTTCTTATCACAACCCTTTTTTGTAGCAGAAGTATTTACTGGTTCTCCG : 1335
Isc : AGGATCGTTTAACCGTAGCAAGAGCGCGAAAAATAGAACGTTTCTTATCACAACCCTTTTTTGTAGCAGAAGTATTTACTGGTTCTCCA : 1335
Ipe : AGGATCGTTTAACCGTAGCAAGAGCGCGAAAAATAGAACGTTTCTTATCACAACCCTTTTTTGTAGCAGAAGTATTTACTGGTTCTCCA : 1293
Ath : AGGATCGCTTAACCGTAGCAAGAGCACGAAAAATTGAGCGTTTCTTATCACAACCCTTTTTCGTAGCAGAAGTATTTACAGGTTCTCCG : 1335

1340 * 1360 * 1380 * 1400 * 1420
Ikl : GGGAAATATGTTGGTCTAACAGAAACAATTAGAGGGTTTCAATTAATCCTTTCCGGAGAATTAGACGGTCTTCCTGAACAGGCCTTTTA : 1415
Ili : GGGAAATATGTTGGTCTAACAGAAACAATTAGAGGGTTTCAATTAATCCTTTCCGGAGAATTAGACGGTCTTCCTGAACAGGCCTTTTA : 1424
Ise : GGGAAATATGTTGGTCTAACAGAAACAATTAGAGGGTTTCAATTAATCCTTTCCGGAGAATTAGACGGTCTTCCTGAACAGGCCTTTTA : 1424
Ici : GGGAAATATGTTGGTCTAACAGAAACAATTAGAGGGTTTCAATTAATCCTTTCCGGAGAATTAGACGGTCTTCCTGAACAGGCCTTTTA : 1424
Isc : GGGAAATATGTTGGTCTAACAGAAACAATTAGAGGGTTTCAATTAATCCTTTCCGGAGAATTAGACGGTCTTCCTGAACAGGCCTTTTA : 1424
Ipe : GGGAAATATGTTGGTCTAACAGAAACAATTAGAGGGTTTCAATTAATCCTTTCCGGAGAATTAGACGGTCTTCCTGAACAGGCCTTTTA : 1382
Ath : GGAAAATATGTTGGTCTAGCGGAAACAATTAGAGGGTTTAATTTGATCCTTTCCGGAGAATTTGATTCTCTTCCCGAACAGGCCTTTTA : 1424

 * 1440 * 1460 * 1480 *
Ikl : TTTGGTAGGTAACATCGATGAAGCTACCGCGAAGGCTATGAACTTAGAAATGGAGAGCAATTTGAAGAAA--- : 1485
Ili : TTTGGTAGGTAACATCGATGAAGCTACCGCGAAGGCTATGAACTTAGAAATGGAGAGCAATTTGAAGAAA--- : 1494
Ise : TTTGGTAGGTAACATCGATGAAGCTACCGCGAAGGCTATGAACTTAGAAATGGAGAGCAATTTGAAGAAA--- : 1494
Ici : TTTGGTAGGTAACATCGATGAAGCTACCGCGAAGGCTATGAACTTAGAAATGGAGAGCAATTTGAAGAAATGA : 1497
Isc : TTTGGTAGGTAACATCGATGAAGCTACCGCGAAGGCTATGAACTTAGAAATGGAGAGCAATTTGAAGAAA--- : 1494
Ipe : TTTGGTAGGTAACATCGATGAAGCTACCGCGAAGGCTATCAACTTAGAAATGGAGAGCAATTTGAAGAAA--- : 1452
Ath : CTTAGTGGGTAACATCGATGAAGCTACTGCGAAGGCTACGAACTTAGAAATGGAGAGTAAATTGAAGAAATGA : 1497

**Figure S84 Multiple sequence alignment of the deduced nucleotide sequences of *atp*B gene.** The origins of the nucleotide sequences used in this alignment are from: *Arabidopsis thaliana* (Ath), *Iodes klaineana* (Ikl), *Iodes cirrhosa* (Ici), *Iodes seretii* (Ise), *Iodes scandens* (Isc), *Iodes perrieri* (Ipe) and *Iodes liberica* (Ili). The nucleotide numbers for each sequence are indicated on the right. In the sequence alignment, identical nucleotides are shown with a black background, and similar residues are shown with a gray background.

* 20 * 40 * 60 * 80 *
Ikl : ATGACCTTAAATCTTTGTGTACTGACCCCTAACCGAATTGTTTGGGATTCAGAAGTGAACGAAATCATTTTATCTACTAATAATGGTCAA : 90
Ili : ATGACCTTAAATCTTTGTGTACTGACCCCTAACCGAATTGTTTGGGATTCAGAAGTGAACGAAATCATTTTATCTACTAATAATGGTCAA : 90
Ise : ATGACCTTAAATCTTTGTGTACTGACCCCTAACCGAATTGTTTGGGATTCAGAAGTGAACGAAATCATTTTATCTACTAATAATGGTCAA : 90
Ipe : ATGACCTTAAATCTTTGTGTACTGACCCCTAACCGAATTGTTTGGGATTCAGAAGTGAACGAAATCATTTTATCTACTAATAATGGTCAA : 90
Ici : ATGACCTTAAATCTTTGTGTACTGACCCCTAACCGAATTGTTTGGGATTCAGAAGTGAAAGAAATCATTTTATCTACTAATAATGGTCAA : 90
Isc : ATGACCTTAAATCTTTGTGTACTGACCCCTAACCGAATTGTTTGGGATTCAGAAGTGAAAGAAATCATTTTATCTACTAATAATGGTCAA : 90
Ath : ATGACCTTAAATCTTTGTGTACTGACTCCGAATCGAATTGTTTGGGATTCAGAAGTAAAAGAAATCATTTTATCTACTAATAGTGGACAA : 90

 100 * 120 * 140 * 160 * 180
Ikl : ATTGGCGTATTACCAAATCATGCCCCTATTGCCACAGCTGTAGATATAGGTATTTTGAAAAAACGCCTTAACGGCCAATGGTTAGCAATG : 180
Ili : ATTGGCGTATTACCAAATCATGCCCCTATTGCCACAGCTGTAGATATAGGTATTTTGAAAAAACGCCTTAACGGCCAATGGTTAGCAATG : 180
Ise : ATTGGCGTATTACCAAATCATGCCCCTATTGCCACAGCTGTAGATATAGGTATTTTGAAAATACGCCTTAAGGGCCAATGGTTAGCAATG : 180
Ipe : ATTGGCGTATTACCAAATCATGCCCCTATTGCCACAGCTGTAGATATAGGTATTTTCAAAATACGCCTTAACGACCAATGGTTAGCAATG : 180
Ici : ATTGGCGTATTACCAAATCATGCCCCTATTGCCACAGCTGTAGATATAGGTATTTTGAAAATACGCCTTAACGACCAATGGTTAGCAATG : 180
Isc : ATTGGCGTATTACCAAATCATGCCCCTATTGCCACAGCTGTAGATATAGGTATTTTGAAAATACGCCTTAACGACCAATGGTTAGCAATG : 180
Ath : ATTGGTGTATTAGCAAACCACGCGCCGATTGCCACAGCTGTTGATATAGGTATTTTGAAAATACGCCTTGCTAACCAATGGTTAACGATG : 180

 * 200 * 220 * 240 * 260 *
Ikl : GCCTTGATGGGTGGTTTTGCTAGAATAGGCAAAAATGAGATCACTATTTTAGTAAATGATGCGGAGAAGAGTAGTGACATTGATCCACAA : 270
Ili : GCCTTGATGGGTGGTTTTGCTAGAATAGGCAAAAATGAGATCACTATTTTAGTAAATGATGCGGAGAAGAGTAGTGACATTGATCCACAA : 270
Ise : GCCTTGATGGGTGGTTTTGCTAGAATAGGCAAAAATGAGATCACTATTTTAGTAAATGATGCAGAGAAGAGTAGTGACATTGATCCACAA : 270
Ipe : GCCTTGATGGGTGGTTTTGCTAGAATAGGCAAAAATGAGATCACTATTTTAGTAAATGATGCAGAGAAGAGTAGTAACATTGATCCACAA : 270
Ici : GCCTTGATGGGCGGTTTTGCTAGAATAGGCAAAAATGAGATCACTATTTTAGTAAATGATGCAGAGAAGAGTAGTGACATTGATCCACAA : 270
Isc : GCCTTGATGGGTGGTTTTGCTAGAATAGGCAAAAATGAGATCACTATTTTAGTAAATGATGCAGAGAAGAGTAGTGACATTGATCCACAA : 270
Ath : GCTCTGATGGGCGGTTTTGCTAGAATAGGCAATAATGAAATCACTATTTTAGTAAATGATGCAGAGAAGAATAGTGACATTGATCCACAA : 270

280 * 300 * 320 * 340 * 360
Ikl : GAAGCTCAGAAAACTCTTGAAATAGCGGAAGCTAACTTGAGAAAGGCTGAGGGAAAGAGACAAACAATTGAGGCAAATTTAGCCCTCCGG : 360
Ili : GAAGCTCAGAAAACTCTTGAAATAGCGGAAGCTAACTTGAGAAAGGCTGAGGGAAAGAGACAAACAATTGAGGCAAATTTAGCCCTCCGG : 360
Ise : GAAGCTCAGAAAACTCTTGAAATAGCGGAAGCTAACTTGAGAAAGGCTGAGGGAAAGAGACAAACAATTGAGGCAAATTTAGCCCTCCGG : 360
Ipe : GAAGCTCAGAAAACTCTTGAAATAGCGGAAGCTAACTTGAGAAAGGCTGAGGGAAAGAGACAAACAATTGAGGCAAATTTAGCCCTCCGG : 360
Ici : GAAGCTCAGAAAACTCTTGAAATAGCGGAAGCTAACTTGAGAAAGGCTGAGGGAAAGAGACAAACAATTGAGGCAAATTTATCCCTCCGG : 360
Isc : GAAGCTCAGAAAACTCTTGAAATAGCGGAAGCTAACTTGAGAAAGGCTGAGGGAAAGAGACAAACAATTGAGGCAAATTTAGCCCTCCGT : 360
Ath : GAAGCTCAGCAAACTCTTGAAATAGCAGAAGCTAACTTGAGAAAAGCTGAAGGTAAGAGACAGACAATTGAGGCTAATCTAGCTCTCAGA : 360
 * 380 * 400
Ikl : CGAGCTAGGACACGAGTAGAGGCTATCAATATGATTTCG--- : 399
Ili : CGAGCTAGGACACGAGTAGAAGCTATCAATATGATTTCG--- : 399
Ise : CGAGCTAGGACACGAGTAGAGGCTATCAATATGATTTCG--- : 399
Ipe : CGAGCTAGGACACGAGTAGAGGCTATCAATATGATTTCG--- : 399
Ici : CGAGCTAGGACACGAGTAGAGGCTATCAATATGATTTCGTAA : 402
Isc : CGAGCGAGGACACGAGTAGAGGCTATC--------------- : 387
Ath : CGAGCTCGGACACGAGTCGAGGCTCTCAATACGATTTGA--- : 399

**Figure S85 Multiple sequence alignment of the deduced nucleotide sequences of *atp*E gene.** The origins of the nucleotide sequences used in this alignment are from: *Arabidopsis thaliana* (Ath), *Iodes klaineana* (Ikl), *Iodes cirrhosa* (Ici), *Iodes seretii* (Ise), *Iodes scandens* (Isc), *Iodes perrieri* (Ipe) and *Iodes liberica* (Ili). The nucleotide numbers for each sequence are indicated on the right. In the sequence alignment, identical nucleotides are shown with a black background, and similar residues are shown with a gray background.

* 20 * 40 * 60 * 80 *
Ikl : ATGAAAACTGTAACCGACTCTTTCGTTTCTTTGGGCTACTGGCCATCCGCCGGGAGTTTCGGGTTTAATACCGATATTTTAGCAACAAAT : 90
Ili : ATGAAAACTGTAACCGACTCTTTCGTTTCTTTGGGCTACTGGCCATCCGCCGGGAGTTTCGGGTTTAATACCGATATTTTAGCAACAAAT : 90
Ipe : ATGAAAACTGTAACCGACTCTTTCGTTTCTTTGGGCTACTGGCCATCCGCCGGGAGTTTCGGGTTTAATACCGATATTTTAGCAACAAAT : 90
Ise : ATGAAAACTGTAACCGACTCTTTCGTTTCTTTGGGCTCCTGGCCATCCGCCGGGAGTTTCGGGTTTAATACCGATATTTTAGCAACAAAT : 90
Ici : ATGAAAACTGTAACCGACTCTTTCGTTTCTTTGGGCTACTGGCCATCCGCCGGGAGTTTCGGGTTTAATACCGATATTTTAGCAACAAAT : 90
Ath : ATGAAAAATTTAACCGATTCTTTCGTTTACTTGGGTCACTGGCCATCCGCCGGGAGTTTCGGATTTAATACCGATATTTTAGCAACAAAT : 90
Isc : ATGAAAACTGTAACCGACTCTTTCCTTTCTTTGGGCTACTGGCCATCCGCCGGGAGTTTCGGGTTTAATACCGATATTTTAGCAACAAAT : 90

 100 * 120 * 140 * 160 * 180
Ikl : CCAATAAATCTAAGTGTAGTGCTTGGTGTATTGATCTTTTTTGGAAAGGGAGTGT-GTGCGAGTTGTTTA-----CGAAAACAGAGGATC : 174
Ili : CCAATAAATCTAAGTGTAGTGCTTGGTGTATTGATCTTTTTTGGAAAGGGAGTGT-GTGCGAGTTGTTTA-----CGAAAACAGAGGATC : 174
Ipe : CCAATAAATCTAAGTGTAGTGCTTGGTGTATTGATCTTTTTTGGAAAGGGAGTGT-GTGCGAGTTGTTTA-----CGAAAACAGAGGATC : 174
Ise : CCAATAAATCTAAGTGTAGTGCTTGGTGTATTGATCTTTTTTGGAAAGGGAGTGT-GTGCGAGTTGTTTA-----CGAAAACAGAGGATC : 174
Ici : CCAATAAATCTAAGTGTAGTACTTGGTGTATTGATCTTTTTTGGAAAGGGAGTGTTAAGTGATTTATTAGATAATCGAAAACAGAGGATC : 180
Ath : CCAATAAATCTAAGTGTAGTTTTCGGTGTATTGATCTTTTTTGGAAAGGGAGTGTTAAATGATTTATTAGATAACCGAAAGCAGAGGATA : 180
Isc : CCAATAAATCTAAGTGTAGTACTTGGTGTATTGATCTTTTTTGGAAAGGGAGTGT-GTGCGAGTTGTTTA-------------------- : 159

 * 200 * 220 * 240 * 260 *
Ikl : TTGAATACTATTCAAAATTCAGCAGAACTGCGTGGGAGGGCCATTGAACAGCTGGAAAAGGCTCGAGCTCGCTTACGGAAAGTAGAAATG : 264
Ili : TTGAATACTATTCAAAATTCAGCAGAACTGCGTGGGAGGGCCATTGAACAGCTGGAAAAGGCTCGAACTCGCTTACGGAAAGTGGAAATG : 264
Ipe : TTGAATACTATTCAAAATTCAGCAGAACTCCGTGGGAGGGCCATTGAACAGCTGGAAAAGGCTCGAGCTCGCTTACGGAAAGTAGAAATG : 264
Ise : TTGAATACTATTCAAAATTCAGCAGAACTGCGTGGGAGGGCCATTGAACAGCTGGAAAAGGCTCGAGCTCGCTTACGGAAAGTAGAAATG : 264
Ici : TTGAATACTATTCAAAATTCAGCAGAACTGCGTGGGAGGGCCATTGAACAGCTGGAAAAGGCTCGAGCTCGCTTACGGAAAGTAGAAATG : 270
Ath : TTAAATACTATTCGAAATTCAGAAGAACTGCGTGAAGGAGCTATTCAACAATTAGAAAATGCCCGGGCGCGCTTGCGTAACGTAGAAACG : 270
Isc : ------------CAAAATTCAGCAGAACTGCGCGGGAGGGCCATTGAACAGCTGGAAAAGGCTCGAGCTCGCTTA------GCAGAACTG : 231

280 * 300 * 320 * 340 * 360
Ikl : GAAGCGGATCAGTTTCGAGTGAATGGATACTCTGAGATAGAACGAGAAAAACGGAATTTGATTAATTCGACTTATAAGGCTTTGGAACAA : 354
Ili : GAAGCGGATCAGTTTCGAGTGAATGGATACTCTGAGATAGAACGAGAAAAACGGAATTTGATTAATTCGACTTATAAGGCTTTGGAACAA : 354
Ipe : GAAGCGGATCAGTTTCGAGTGAATGGATACTCTGAGATAGAACGAGAAAAACAGAATTTGATTAATTCGACTTATAAGGCTTTGGAACAA : 354
Ise : GAAGCGGATCAGTTTCGAGTGAATGGATACTCTGAGATAGAACGAGAAAAAGGGAATTTGATTAATTCGACTTATAAGGCTTTGGAACAA : 354
Ici : GAAGCGGATCAGTTTCGAGTGAATGGATACTCTGAGATAGAACGAGAAAAACGTAATTTGATTAATTCGACTTATAAGGCTTTGGAACAA : 360
Ath : GAAGCGGATAAGTTTCGCGTGAATGGATACTCTGAAATCGAACGAGAAAAATTGAATTTGATTAATTCAACTTATAAAACTTTGAAACAA : 360
Isc : ---------------CGCGGGAGGG----CCATTGAACAGCTGGAAAAGGCTCGAGCTCGCTTACTTCAATTACTTATG----------- : 291

 * 380 * 400 * 420 * 440 *
Ikl : TTCGAAAACTACAAAAACGAAACTATTCGTTTTGAACAACAAAGGGCGATTAATCAAGTCCGACAACGGGTTTTCCAACAAGCCTTACAA : 444
Ili : TTCGAAAACTACAAAAACGAAACTATTCGTTTTGAACAACAAAGGGCGATTAATCAAGTCCGACAACGGGTTTTCCAACAAGCCTTACAA : 444
Ipe : TTCGAAAACTACAAAAACGAAACTATTCGTTTTGAACAACAAAGGGCGATTAATCAAGTCCGACAACGGGTTTTCCAACAAGCCTTACAA : 444
Ise : TTCGAAAACTACAAAAACGAAACTATTCGTTTTGAACAACAAAGGGCAATTAATCAAGTCCGACAACGGGTTTTCCAACAAGCCTTACAA : 444
Ici : TTCGAAAACTACAAAAACGAAACTATTCGTTTTGAACAACAAAGGGCGATTAATCAAGTCCGACAACGGGTTTTCCAACAAGCCTTACAA : 450
Ath : TTAGAAAATTACAAAAATGAAACCATTCTTTTTGAGCAACAAAGAACAATTAATCAAGTCCGCGAACGGGTTTTCCAACAAGCTTTACAA : 450
Isc : ------------------------------------------------------------------------------------------ : -

 460 * 480 * 500 * 520 * 540
Ikl : GGAGCTCTAGGAACTCTGAATAGTTGTTTGAACAACGAGTTACATTTACGTACTATCAGTGCCAATATTGGCATGTTGGGGTCGATGAAA : 534
Ili : GGAGCTCTAGGAACTCTGAATAGTTGTTTGAACAACGAGTTACATTTACGTACTATCAGTGCCAATATTGGCATGTTGGGGTCGATGAAA : 534
Ipe : GGAGCTCTAGGAACTCTGAATAGTTGTTTGAACAACGAGTTACATTTACGTACTATCAGTGCCAATATTGGCATGTTGGGGTCGATGAAA : 534
Ise : GGAGCTCTAGGAACTCTGAATAGTTGTTTGAACAACGAGTTACATTTACGTACTATCAGTGCCAATATTGGCATGTTGGGGTCTATGAAA : 534
Ici : GCAGCTCTAGGAACTCTGAATAGTTGTTTGAACAACGAGTTACATTTACGTACTATCAGTGCCAATATTGGCATGTTGAGGTCGATGAAA : 540
Ath : GGAGCTATAGGAACCCTAAATAGTTGTTTGAGTAATGAGTTACATTTACGTACTATTAATGCAAATATTGGGATGTTTGGTACGATGAAA : 540
Isc : ------------------------------------------------------------------------------------------ : -

*
Ikl : GAAATAACTGAT--- : 546
Ili : GAAATAACTGAT--- : 546
Ipe : GAAATAACTGAT--- : 546
Ise : GAAATAACTGAT--- : 546
Ici : GAAATAACTGATTAG : 555
Ath : GAAATAACTGATTAA : 555
Isc : --------------- : -

**Figure S86 Multiple sequence alignment of the deduced nucleotide sequences of *atp*F gene.** The origins of the nucleotide sequences used in this alignment are from: *Arabidopsis thaliana* (Ath), *Iodes klaineana* (Ikl), *Iodes cirrhosa* (Ici), *Iodes seretii* (Ise), *Iodes scandens* (Isc), *Iodes perrieri* (Ipe) and *Iodes liberica* (Ili). The nucleotide numbers for each sequence are indicated on the right. In the sequence alignment, identical nucleotides are shown with a black background, and similar residues are shown with a gray background.

* 20 * 40 * 60 * 80 *
Isc : ATGAATCCACTGATTTCTGCTGCTTCCGTTATTGCTGCTGGATTGGCCGTAGGGCTTGCTTCTATTGGACCTGGAGTTGGTCAAGGCACT : 90
Ise : ATGAATCCACTGATTTCTGCTGCTTCCGTTATTGCTGCTGGATTGGCCGTAGGGCTTGCTTCTATTGGACCTGGAGTTGGTCAAGGCACT : 90
Ipe : ATGAATCCACTGATTTCTGCTGCTTCCGTTATTGCTGCTGGATTGGCCGTAGGGCTTGCTTCTATTGGACCTGGAGTTGGTCAAGGCACT : 90
Ici : ATGAATCCACTGATTTCTGCTGCTTCCGTTATTGCTGCTGGATTGGCCGTAGGGCTTGCTTCTATTGGACCTGGAGTTGGTCAAGGCACT : 90
Ikl : ATGAATCCACTGATTTCTGCTGCTTCCGTTATTGCTGCTGGATTGGCCGTAGGGCTTGCTTCTATTGGACCTGGAGTTGGTCAAGGCACT : 90
Ili : ATGAATCCACTGATTTCTGCTGCTTCCGTTATTGCTGCTGGATTGGCCGTAGGGCTTGCTTCTATTGGACCTGGAGTTGGTCAAGGCACT : 90
Ath : ATGAATCCACTGGTTTCTGCTGCTTCGGTTATTGCTGCTGGGTTGGCTGTTGGGCTTGCTTCTATTGGACCTGGAGTCGGTCAAGGTACA : 90

 100 * 120 * 140 * 160 * 180
Isc : GCTGCGGGTCAAGCTGTAGAAGGTATCGCGAGACAGCCCGAGGCTGAAGGAAAAATCCGGGGCACTTTATTGCTTAGTCTAGCTTTTATG : 180
Ise : GCTGCGGGTCAAGCTGTAGAAGGTATCGCGAGACAGCCCGAGGCTGAAGGAAAAATCCGGGGCACTTTATTGCTTAGTCTAGCTTTTATG : 180
Ipe : GCTGCGGGTCAAGCTGTAGAAGGTATCGCGAGACAGCCCGAGGCTGAAGGAAAAATCCGGGGCACTTTATTGCTTAGTCTAGCTTTTATG : 180
Ici : GCTGCGGGTCAAGCTGTAGAAGGTATCGCGAGACAGCCCGAGGCTGAAGGAAAAATCCGGGGCACTTTATTGCTTAGTCTAGCTTTTATG : 180
Ikl : GCTGCGGGTCAAGCTGTAGAAGGTATCGCGAGACAACCTGAGGCTGAAGGAAAAATCCGGGGCACTTTATTGCTTAGTCTAGCTTTTATG : 180
Ili : GCTGCGGGTCAAGCTGTAGAAGGTATCGCGAGACAACCTGAGGCTGAAGGAAAAATCCGGGGCACTTTATTGCTTAGTCTAGCTTTTATG : 180
Ath : GCTGCGGGTCAAGCTGTCGAAGGTATCGCGAGACAACCTGAGGCAGAAGGAAAAATACGAGGTACTTTATTGCTTAGTTTGGCTTTTATG : 180

* 200 * 220 * 240
Isc : GAAGCTTTAACAATTTATGGACTGGTTGTAGCATTAGCACTTTTATTTGCGAATCCTTTTGTT--- : 243
Ise : GAAGCTTTAACAATTTATGGACTGGTTGTAGCATTAGCACTTTTATTTGCGAATCCTTTTGTT--- : 243
Ipe : GAAGCTTTAACAATTTATGGACTGGTTGTAGCATTAGCACTTTTATTTGCGAATCCTTTTGTT--- : 243
Ici : GAAGCTTTAACAATTTATGGACTGGTTGTAGCATTAGCACTTTTATTTGCGAATCCTTTTGTTTAA : 246
Ikl : GAAGCTTTAACAATTTATGGACTGGTTGTA------------------------------------ : 210
Ili : GAAGCTTTAACAATTTATGGACTGGTTGTAGCATTAGCACTTTTATTTGCGAATCCTTTTGTT--- : 243
Ath : GAAGCTTTAACAATTTATGGCCTGGTTGTAGCATTAGCGCTTTTATTTGCGAATCCTTTTGTTTAA : 246

**Figure S87 Multiple sequence alignment of the deduced nucleotide sequences of *atp*H gene.** The origins of the nucleotide sequences used in this alignment are from: *Arabidopsis thaliana* (Ath), *Iodes klaineana* (Ikl), *Iodes cirrhosa* (Ici), *Iodes seretii* (Ise), *Iodes scandens* (Isc), *Iodes perrieri* (Ipe) and *Iodes liberica* (Ili). The nucleotide numbers for each sequence are indicated on the right. In the sequence alignment, identical nucleotides are shown with a black background, and similar residues are shown with a gray background.

* 20 * 40 * 60 * 80 *
Ikl : ATGAATGTTCTATCATGTTCCATCAACACCCTAA------AGGGGTTATACGATATATCCGGCGTGGAAGTCGGCCAACATTTGTATTGG : 84
Ili : ATGAATGTTCTATCATGTTCCATCAACACCCTAA------AGGGGTTATACGATATATCCGGCGTGGAAGTCGGCCAACATTTGTATTGG : 84
Ise : ATGAATGTTCTATCATGTTCCATCAACACCCTAA------AGGGGTTATACGATATATCCGTCGTGGAAGTCGGCCAACATTTGTATTGG : 84
Ipe : ATGAATGTTCTATCATGTTCCATCAACACCCTAA------AGGGGTTATACGATATATCCGGCGTGGAAGTCGGCCAACATTTGTATTGG : 84
Ici : ATGAATGTTCTATCATGTTCCATCAACACCCTAA------AGGGGTTATACGATATATCCGGCGTGGAAGTCGGCCAACATTTGTATTGG : 84
Isc : ATGAATGTTCTATCATGTTCCATCAACACCCTAA------AGGGGTTATACGATATATCCGGCGTGGAAGTCGGCCAACATTTGGATTGG : 84
Ath : ATGAATGTTTTATCATGTTCCATCAACACACTAATAAAAGAAGGGTTATATGAGATATCTGGTGTAGAAGTAGGCCAACATTTCTATTGG : 90

 100 * 120 * 140 * 160 * 180
Ikl : GAAATAGGGGGTTTCAAAATCCACGGCCAGGTACTTATTACTTCTTGGGTTGTAATTGCTATCTTATTAGGTTCAGCTACTATAGCTGTC : 174
Ili : GAAATAGGGGGTTTCAAAATCCACGGCCAGGTACTTATTACTTCTTGGGTTGTAATTGCTATCTTATTAGGTTCAGCTACTATAGCTGTC : 174
Ise : GAAATAGGGGGTTTCAAAATCCACGGCCAGGTACTTATTACTTCTTGGGTTGTAATTGCTATCTTATTAGGTTCAGCTACTATAGCTGTC : 174
Ipe : GAAATAGGGGGTTTCAAAATCCACGGCCAGGTACTTATTACTTCTTGGGTTGTAATTGCTATCTTATTAGGTTCAGCTACTATAGCTGTC : 174
Ici : CAAATAGGGGGTTTCAAAATCCACGGCCAGGTACTTATTACTTCTTGGGTTGTAATTGCTATCTTATTAGGTTCAGCTACTATAGCTGTC : 174
Isc : CAAATAGGGGGTTTCAAAATCCACGGCCAGGTACTTATTACTTCTTGGGTTGTAATTGCTATCTTATTAGGTTCAGCTACTATAGCTGCC : 174
Ath : CAAATAGGGGGTTTCCAGGTCCATGCCCAAGTTCTTATTACTTCTTGGGTTGTAATTGCTATCTTATTAGGTTCCGCAGTTCTAGCGATT : 180

 * 200 * 220 * 240 * 260 *
Ikl : CGGAACCCACAAACCATTCCGACCAGAGGTCAGAATTTCTTCGAATATGTCCTTGAATTCATTCGAGATGTGAGTAAAACTCAAATTGGA : 264
Ili : CGTAACCCACAAACCATTCCGACCAGAGGTCAGAATTTCTTCGAATATGTCCTTGAATTCATTCGAGATGTGAGTAAAACTCAAATTGGA : 264
Ise : CGAAACCCACAAACCATTCCGACCAGAGGTCAGAATTTCTTCGAATATGTCCTTGAATTCATTCGAGATGTGAGTAAAACTCAAATTGGA : 264
Ipe : CGGAACCCACAAACCATTCCGACCAGAGGTCAGAATTTCTTCGAATATGTCCTTGAATTCATTCGAGATGTGAGTAAAACTCAAATTGGA : 264
Ici : CGGAACCCACAAACCATTCCGACCAGAGCTCAGAATTTCTTCGAATATGTCCTTGAATTCATTCGAGATGTGAGTAAAACTCAAATTGGA : 264
Isc : CGGAATCCACAAACCATTCCGACCAGAGCTCAGAATTTCTTCGAATATGTCCTTGAATTCATTCGAGATGTGAGTAAAACTCAAATTGGA : 264
Ath : CGCAATCCACAAACAATTCCAACTGACGGCCAAAATTTCTTTGAATTTGTCCTTGAATTCATTCGAGACGTGAGTAAAACCCAGATTGGA : 270

280 * 300 * 320 * 340 * 360
Ikl : GAAGAATATGGTCCTTGGGTTTCTTTTATTGGAACTATGTTTCTATTTATTTTTGTTTCGAATTGGTCAGGGGCTCTTTTACCCTGGAAA : 354
Ili : GAAGAATATGGTCCTTGGGTTTCTTTTATTGGAACTATGTTTCTATTTATTTTTGTTTCGAATTGGTCAGGGGCTCTTTTACCCTGGAAA : 354
Ise : GAAGAATATGGTCCTTGGGTTTCTTTTATTGGAACTATGTTTCTATTTATTTTTGTTTCTAATTGGTCAGGGGCTCTTTTACCCTGGAAA : 354
Ipe : GAAGAATATGGTCCTTGGGTTTCGTTTATTGGAACTATGTTTCTATTTATTTTTGTTTCTAATTGGTCAGGGGCTCTTTTACCCTGGAAA : 354
Ici : GAAGAATATGGTCCTTGGGTTTCTTTTATTGGAACTATGTTTCTATTTATTTTTGTTTCTAATTGGTCGGGGGCTCTTTTACCCTGGAAA : 354
Isc : GAAGAATATGGTCCTTGGGTTTCTTTTATTGGAACTATGTTTCTATTTATTTTTGTTTCTAATTGGTCAGGGGCTCTTTTACCCTGGAAA : 354
Ath : GAAGAATATGGTCCATGGGTTCCCTTTATTGGAACCCTGTTTTTATTTATTTTTGTTTCTAACTGGTCAGGAGCCCTTTTACCGTGGAAA : 360

 * 380 * 400 * 420 * 440 *
Ikl : ATCATCCAATTACCTCATGGGGAGTTAGCCGCACCTACGAATGATATAAATACTACTGTTGCTTTGGCTTTACTCACATCAGTGGCATAT : 444
Ili : ATCATCCAATTACCTCATGGGGAGTTAGCCGCACCTACGAATGATATAAATACTACTGTTGCTTTGGCTTTACTCACATCAGTGGCATAT : 444
Ise : ATCATCCAATTACCTCATGGGGAGTTAGCCGCACCTACGAATGATATAAATACTACTGTTGCTTTGGCTTTACTCACATCAGTGGCATAT : 444
Ipe : ATCATCCAATTACCTCATGGGGAGTTAGCCGCACCTACGAATGATATAAATACTACTGTTGCTTTGGCTTTACTCACATCAGTGGCATAT : 444
Ici : ATCATCCAATTACCTCATGGGGAGTTAGCCGCACCTACGAATGATATAAATACTACTGTTGCTTTGGCTTTACTCACGTCAGTGGCATAT : 444
Isc : ATCATCCAATTACCTCATGGGGAGTTAGCCGCACCTACGAATGATATAAATACTACTGTTGCTTTGTCTTTACTCACGTCAGTGGCATAT : 444
Ath : ATTATCCAGTTACCTCAAGGGGAGTTAGCAGCACCAACGAATGATATAAATACGACGGTTGCTTTAGCTTTACTCACATCAGTAGCCTAT : 450

 460 * 480 * 500 * 520 * 540
Ikl : TTCTATGCGGGTCTTAGCAAAAAAGGATTAGGTTATTTCGGGAAATATATTCAACCAACTCCAATACTTTTACCCATTAACATCTTAGAA : 534
Ili : TTCTATGCGGGTCTTAGCAAAAAAGGATTAGGTTATTTCGGGAAATATATTCAACCAACTCCAATACTTTTACCCATTAACATCTTATAA : 534
Ise : TTCTATGCGGGTCTTAGCAAAAAAGGATTAGGTTATTTCGGGAAATATATTCAACCAACTCCAATACTTTTACCCATTAACATCTTAGAA : 534
Ipe : TTCTATGCGGGTCTTAGCAAAAAAGGATTAGGTTATTTCGGGAAATATATTCAACCAACTCCAATACTTTTACCCATTAACATCTTAGAA : 534
Ici : TTCTATGCGGGTCTTAGCAAAAAAGGATTAGGTTATTTCGGTAAATATATTCAACCAACTCCAATACTTTTACCCATTAACATCTTAGAA : 534
Isc : TTCTATGCGGGCCTTAGCAAAAAAGGATTAGGTTATTTCGGTAAATATATTCAACCAACTCCAATACTTTTACCCATTAACATCTTAGAA : 534
Ath : TTTTATGCGGGGCTTAGCAAAAAAGGATTAGGGTATTTCAGTAAATACATTCAACCAACCCCAATTCTTTTACCCATTAACATCTTAGAA : 540

* 560 * 580 * 600 * 620 *
Ikl : GATTTCACAAAGCCCTTATCGCTGAGTTTTCGACTTTTCGGGAATATATTGGCTGATGAATTAGTAGTTGTTGTTCTTGTTTCTTTAGTA : 624
Ili : GATTTCACAAAGCCCTTATCGCTTAGTTTTCGACTTTTCGGGAATATATTGGCTGATGAATTAGTAGTTGTTGTTCTTGTTTCTTTAGTA : 624
Ise : GATTTCACAAAGCCCTTATCGCTTAGTTTTCGACTTTTCGGGAATATATTGGCTGATGAATTAGTAGTTCTTGTTCTTGTTTCTTTAGTA : 624
Ipe : GATTTCACAAAGCCCTTATCGCTTAGTTTTCGACTTTTCGGGAATATATTGGCTGATGAATTAGTAGTTGTTGTTCTTGTTTCTTTAGTA : 624
Ici : GATTTCACAAAGCCCTTATCGCTTAGTTTTCGGCTTTTCGGGAATATATTGGCTGATGAATTAGTAGTTGTTGTTCTTGTTTCTTTAGTA : 624
Isc : GATTTCACAAAGCCCTTATCGCTTAGTTTTCGACTTTTCGGGAATATATTGGCTGATGAATTAGTAGTTGTTGTTCTTGTTTCTTTAGTA : 624
Ath : GATTTTACAAAACCCCTATCACTTAGTTTTCGACTTTTCGGAAATATATTAGCCGATGAATTAGTCGTTGTTGTTCTTGTTTCTTTAGTA : 630

 640 * 660 * 680 * 700 * 720
Ikl : CCTTTAGTAGTTCCTATACCTGTGATGTTCCTTGGATTATTTACAAGTGGTATCCAAGCTCTTATTTTTGCAACTTTAGCCGCAGCTTAT : 714
Ili : CCTTTAGTAGTTCCTATACCTGTGATGTTCCTTGGATTATTTACAAGTGGTATCCAAGCTCTTATTTTTGCAACTTTAGCCGCAGCTTAT : 714
Ise : CCTTTAGTAGTTCCTATACCTGTGATGTTCCTTGGATTATTTACAAGTGGTATCCAAGCTCTTATTTTTGCAACTTTAGCCGCAGCTTAT : 714
Ipe : CCTTTAGTAGTTCCTATACCTGTGATGTTCCTTGGATTATTTACAAGTGGTATCCAAGCTCTTATTTTTGCAACTTTAGCCGCAGCTTAT : 714
Ici : CCTTTAGTAGTTCCTATACCTGTGATGTTCCTTGGATTATTTACAAGTGGTATCCAAGCTCTTATTTTTGCAACTTTAGCCGCAGCTTAT : 714
Isc : CCTTTAGTAGTTCCTATACCTGTGATGTTCCTTGGATTATTTACAAGTGGTATCCAAGCTCTTATTTTTGCAACTTTAGCCGCAGCTTAT : 714
Ath : CCTTTAGTGGTTCCTATACCTGTTATGTTCCTTGGATTATTTACAAGCGGGATTCAAGCTCTCATTTTTGCCACTTTAGCTGCGGCTTAT : 720

 * 740 *
Ikl : ATAGGCGAATCCATGGAGGGCCATCAT--- : 741
Ili : ATAGGCGAATCCATGGAGGGCCATCAT--- : 741
Ise : ATAGGCGAATCCATGGAGGGCCATCAT--- : 741
Ipe : ATAGGCGAATCCATGGAGGGCCATCAT--- : 741
Ici : ATAGGTGAATCCATGGAGGGCCATCATTAA : 744
Isc : ATAGGTGAATCCATGGAGGGCCATCAT--- : 741
Ath : ATAGGTGAGTCTATGGAAGGTCATCATTAA : 750

**Figure S88 Multiple sequence alignment of the deduced nucleotide sequences of *atp*I gene.** The origins of the nucleotide sequences used in this alignment are from: *Arabidopsis thaliana* (Ath), *Iodes klaineana* (Ikl), *Iodes cirrhosa* (Ici), *Iodes seretii* (Ise), *Iodes scandens* (Isc), *Iodes perrieri* (Ipe) and *Iodes liberica* (Ili). The nucleotide numbers for each sequence are indicated on the right. In the sequence alignment, identical nucleotides are shown with a black background, and similar residues are shown with a gray background.

* 20 * 40 * 60 * 80 *
Isc : ---------ATGATATTTTTCACTTTAGAGCATATATTAACCTTTTCGATCGTTTCAGTTGTAATTACAATTCATTTTATAACCTTATTC : 81
Ise : ------------------------------------------------------------------------------------TTATTC : 6
Ici : ATGATATTTTTAACTTTAGAGCATATATTAACTCATATTTCCTTTTCGATCGTTTCAATTGTAATTACAATTCATTTTATAACCTTATTC : 90
Ikl : ATGATATTTTTAACTTTAGAGCATATATTAACTCATATTTCCTTTTCGATCGTTTCAATTGTAATTACAATTCATTTTATAACCTTATTC : 90
Ipe : ATGATATTTTTAACTTTAGAGCATATATTAACTCATATTTCCTTTTCCATCGTTTCAATTGTCATTACAATTCATTTTATAACCTTATTC : 90
Ili : ATGATATTTTTAACTTTAGAGCATATATTAACTCATATTTCCTTTTCGATCGTTTCAATTGTAATTACAATTCATTTTATAACCTTATTC : 90
Ath : ATGATTTTTTCAATTTTAGAGCATATATTAACTCATATATCTTTTTCGGTCGTTTCAATTGTGCTGACAATTTATTTTTTAACTTTATTA : 90

 100 * 120 * 140 * 160 * 180
Isc : GTC------GATGAAATTGTAAGATTATATGATTCGTCAGAAAAGGGCATGATAGCTGTTTTTTCTTGTATAACAGGATTATTAGTCACT : 165
Ise : GTC------GATGAAATTGTAAGATTATATGATTCGTCAGAAAAGGGCATGATAGCTGTTTTTTCTTGTATAACAGGATTATTAGTCACT : 90
Ici : GTC------GATGAAATTGTAAGATTATATGATTCGTCAGAAAAGGGCATGATAGCAGTTTTTTCTTGTATAACAGGATTATTAGTCACT : 174
Ikl : GTC------GATGAAATTGTAAGATTATATGATTCGTCAGAAAAGGGCATGATAGCTGTTTTTTCTTGTATAACAGGATTATTAGTCACT : 174
Ipe : GTC------GATGAAATTGTAAGATTATATGATTCGTCAGAAAAGGGCATGATAGCTGTTTTTTCTTGTATAACAGGATTATTAGTCACT : 174
Ili : GTC------GATGAAATTGTAAGATTATATGATTCGTCAGAAAAGGGCATGATAGCTGTTTTTTCTTGTATAACAGGATTATTAGTCACT : 174
Ath : GTTAATTTAGATGAAATCATAGGATTTTTTGATTCATCAGATAAAGGAATCATAATTACGTTTTTTGGTATAACAGGATTATTATTAACT : 180

 * 200 * 220 * 240 * 260 *
Isc : CGTTGCATTTATTCAGGGCATTTTCCACTAAGTGATTTATATGAATCATTAATCTTCCTTTCATGGAGTTTCTCAATTATTCATCTAATT : 255
Ise : CGTTGGATTTATTCAGGGCATTTTCCACTAAGTGATTTATATGAATCATTAATCTTCCTTTCATGGAGTTTTTCAATTATTCATCTAATT : 180
Ici : CGTTGGATTTATTCAGGGCATTTTCCACTAAGTGACTTATATGAATCATTAATCTTCCTTTCATGGAGTTTCTCAATTATTCATCTAATT : 264
Ikl : CGTTGGATTTATTCAGGGCATTTTCCACTAAGTGATTTATATGAATCATTAATCTTCCTTTCATGGAGTTTCTCAATTATTCATCTAATT : 264
Ipe : CGTTGGATTTATTCAGGGCATTTTCCACTAAGTGATTTATATGAATCATTAATCTTCCTTTCATGGAGTTTCTCAATTATTCATCTAATT : 264
Ili : CGTTGGATTTATTCAGGGCATTTTCCACTAAGTGATTTATATGAATCATTAATCTTCCTTTCATGGAGTTTCTCAATTATTCATCTAATT : 264
Ath : CGTTGGATTTATTCAGGACATTTTCCATTAAGTAATTTATATGAATCATTAATTTTTCTTTCGTGGGCTTTTTCAATTATTCATATGGTT : 270

280 * 300 * 320 * 340 * 360
Isc : ---------------------------------------------CCTTATTTCAAAAAAAATAAAAAAGAT---TTAAGGACAATAACT : 297
Ise : CCTTATAGATCGGAAGAGCACACGTCTTCAATTATTCATCTAATTCCTTATTTCAAAAAAAATAAAAAAAAT---TTAAGGACAATAACT : 267
Ici : ---------------------------------------------CCTTATTTCAAAAAAAATAAAACAAAT---TTAAGGCCAATAACT : 306
Ikl : ---------------------------------------------CCTTATTTCAAAAAAAATAAAACAAAT---TTAAGGACAATAACT : 306
Ipe : ---------------------------------------------CCTTATTTAAAAAAAAATAAAACAAAT---TTAAGGACAATAACT : 306
Ili : ---------------------------------------------CCTTATTTCAAAAAAAATAAAAAAAAT---TTAAGGACAATAACT : 306
Ath : ---------------------------------------------TCCTATTTTAATAAAAAACAACAAAATAAGTTAAACACAATAACT : 315

 * 380 * 400 * 420 * 440 *
Isc : GCGCCAAGTGCTATTTTTAGCCAAGGCTTTACTACTTCAGGTCTTTTAACTGAAATAAATCAATCCACAATATTAGTACCCGCTCTTCAA : 387
Ise : GCGCCAAGTGCTATTTTTAGCCAAGGCTTTACTACTTCAGGTCTTTTAACTGAAATACATCAATCCACAATATTAGTACCCGCTCTTCAA : 357
Ici : GTGCCAAGTGCTATTTTTAGCCAAGGCTTTACTACTTCAGGTCTTTTAACTGAAATACATCAATCCACAATATTAGTACCCGCTCTTCAA : 396
Ikl : GCGCCAAGTGCTATTTTTAGCCAAGGCTTTACTACTTCAGGTCTTTTAACTGAAATACATCAATCCACAATATTAGTACCCGCTCTTCAA : 396
Ipe : GCGCCAAGTGCTATTTTTAGCCAAGGCTTTACTACTTCAGGTCTTTTAACTGAAATACATCAATCCACAATATTAGTACCCGCTCTTCAA : 396
Ili : GCGCCAAGTGCTATTTTTAGCCAAGGCTTTACTACTTCAGGTCTTTTAACTGAAATACATCAATCCACAATATTAGTACCCGCTCTTCAA : 396
Ath : GCGCCAAGTGTTATTTTTATTCAGGGTTTTGCTACTTCAGGTCTTTTAAACAAAATGCCTCAGTCTGCAATATTAGTACCAGCTCTCCAG : 405

 460 * 480 * 500 * 520 * 540
Isc : TCCGAATGGTTAATAATGCACGTAAGTATGATGATATTAGGCTATGCTGCTCTTTTATGTGGATCATTATTGTCAGTAGCACTTCTAGTC : 477
Ise : TCCGAATGGTTAATAATGCACGTAAGTATGATGATATTAGGCTATGCTGCTCTTTTATGTGGATCATTATTATCAGTAGCACTTCTAGTC : 447
Ici : TCCGAATGGTTAATAATGCACGTAAGTATGATGATATTAGGCTATGCTTCTCTTTTATGTGGATCATTATTGTCAGTAGCACTTCTAGTC : 486
Ikl : TCCGAATGGTTAATAATGCACGTAAGTATGATGATATTAGGCTATGCTGCTCTTTTATGTGGATCATTATTATCAGTAGCACTTCTAGTC : 486
Ipe : TCCGAATGGTTAATAATGCACGTAAGTATGATGATATTAGGCTATGCTGCTCTTTTATGTGGATCATTATTATCAGTAGCACTTCTAGTC : 486
Ili : TCCGAATGGTTAATAATGCACGTAAGTATGATGATATTAGGCTATGCTGCTCTTTTATGTGGATCATTATTATCAGTAGCACTTCTAGTC : 486
Ath : TCCCAGTGGTTAATGATGCACGTAAGTATGATGATATTAGGCTATGGCGCTCTGTTATGCGGATCATTATTATCAATAGCTCTTCTAGTG : 495

* 560 * 580 * 600 * 620 *
Isc : ATTACATTTCGAAAAAATAGAAAAAATTATTCTAAAAGTTATAA------------TTTATTAAATAAGCCGTTTTTCTTTGGCGAAATC : 555
Ise : ATTACATTTCGAAAAAATAGAAAGAATTTTTCTAAAAGTCATAA------------TTTATTAAATAAGCCGTTTTTCTTTGGCGAAATC : 525
Ici : ATTACATTTCGAAAAAATAGAAAAAATTATTCAAAAAGTCATAA------------TTTATTAAATAAGCCGTTTTTCTTTGGCGAAATC : 564
Ikl : ATTACATTTCGAAAAAATAGAAAGATTTTTTCGAAAAGTCATAA------------TTTATTAAATAAGCCGTTTTTCTTTGACGAAATC : 564
Ipe : ATTACATTTCGAAAAAATATTTCTATTTTTTCGAAAAGTCATAA------------TTTATTAAATAAGCCGTTTTTCTTTGGCGAAATC : 564
Ili : ATTACATTTCGAAAAAATCTTTCTATTTTTTCGAAAAGTCATAA------------TTTATTAAATAAGCCGTTTTTCTTTGACGAAATC : 564
Ath : ATTACATTTCGCAAAGTCGGACCTACTTTTTGGAAAAAGAATATAAAAAAAAATTTTTTATTAAATGAATTATTTTCTTTTGATGTACTT : 585

 640 * 660 * 680 * 700 * 720
Isc : CAAT------ACATGAATAAAAAAAGAAATGTTTTACGA-AATACTTCTCTTTTT--------TCTG-------CTAAAAATTATTACCA : 623
Ise : CAATACCAATACATGAATAAAAAAAGAAATGTTTTACAA-AATACTTCTCTTTTT--------TCTT-------CTAAAAATTATTACCA : 599
Ici : CAAT------ACATGAATAAAAAAAGAAATGTTTTACGA-AATACTTCTCTTTTT--------TCTTTTT-CTGCTAAAAATTATTACCA : 638
Ikl : CAAT------ACATGAATAAAAAAAGAAATGTTTTACGA-AATACTTCTCTTTTT--------TCTT-------CTAAAAATTATTACCA : 632
Ipe : CAAT------ACATGAATAAAAAAAGAAATGTTTTACGA-AATACTTCTCTTTTT--------TCTT-------CTAAAAATTATTACCA : 632
Ili : CAAT------ACATGAATAAAAAAAGAAATGTTTTACGA-AATACTTCTCTTTTTAATACTTCTCTTTTTTCTTCTAAAAATTATTACCA : 647
Ath : TACT------ACATAAATGAAAGAAATTCTATTTTACTACAACAAAACATTAATT----TTAGTTTTT------CTAGAAATTATTATAG : 659

 * 740 * 760 * 780 * 800 *
Isc : GTCCCAATTGATTCAGCAACTGGATTATTGGAGTTATCGTGTTATTAGTCTAGGATTTATCTTTTTAACCATAGGTATACTTTCAGGAGC : 713
Ise : GT---------------------------GGAGTTATCGTGTTATTAGTCTAGGATTTATCTTTTTAACCATCGGTATACTTTCGGGAGC : 662
Ici : GTCCCAATTGATTCAGCAACTGGATTATTGGAGTTATCGTGTTATTAGTCTAGGATTTATCTTTTTAACCATAGGTATACTTTCGGGAGC : 728
Ikl : GTACCAATTGATTCAGCAACTGGATTATTGGAGTTATCGTGTTATTAGTCTAGGATTTATCTTTTTAACCATAGGTATACTTTCGGGAGC : 722
Ipe : GTACCAATTGATTCAGCAACTGGATTATTGGAGTTATCGTGTTATTAGTCTAGGATTTTTCTTTTTAACCATAGGTATACTTTCAGGAGC : 722
Ili : GTACCAATTGATTCAGCAACTGGATTATTGGAGTTATCGTGTTATTAGTCTAGGATTTATCTTTTTAACCATAGGTATACTTTCGGGAGC : 737
Ath : GTATCAACTGATTCAACAATTAGATTTTTGGAGTTTTCGTATTATTAGTCTTGGATTTATCTTTTTAACCGTCGGCATTCTTTCAGGAGC : 749

820 * 840 * 860 * 880 * 900
Isc : AGTATGGGCTAATGAAGCATGGGGATCATATTGGAATTGGGATCCAAAAGAAACTTGGGCATTTATTACTTGGATCATATTTGCGATTTA : 803
Ise : AGTATGGGCTAATGAAGCATGGGGATCATATTGGAATTGGGATCCAAAAGAAACTTGGGCATTTATTACTTGGATCATATTTGCGATTTA : 752
Ici : AGTATGGGCTAATGAAGCATGGGGATCATATTGGAATTGGGATCCAAAAGAAACTTGGGCATTTATTACTTGGATCATATTTGCGATTTA : 818
Ikl : AGTATGGGCTAATGAAGCATGGGGATCATATTGGAATTGGGATCCAAAAGAAACTTGGGCATTTATTACTTGGATCATATTTGCGATTTA : 812
Ipe : AGTATGGGCTAATGAAGCATGGGGATCATATTGGAATTGGGATCCAAAAGAAACTTGGGCATTTATTACTTGGATCATATTTGCGATTTA : 812
Ili : AGTATGGGCTAATGAAGCATGGGGATCATATTGGAATTGGGATCCAAAAGAAACTTGGGCATTTATTACTTGGATCATATTTGCGATTTA : 827
Ath : TGTCTGGGCTAATGAGACATGGGGTTCATATTGGAACTGGGACCCAAAAGAAACTTGGGCATTTATTACTTGGACTATATTCGCAATTTA : 839

 * 920 * 940 * 960 * 980 *
Isc : TTTACATACTCGAATAAATAAAAATTTGCAAGGTGTAAATTCCGCAATTGTGCCATCTATAGGCTTTCTTATCATTTGGATATGTTATTT : 893
Ise : TTTACATACTCGAATAAATAAAAATTTGCAAGGTGTAAATTCCGCAATTGTTCCATCTATAGGCTTTCTTATCATTTGGATATGTTATTT : 842
Ici : TTTACATACTCGAATAAATAAAAATTTGCAAGGTGTAAATTCCGCAATTGTGCCATCTATAGGCTTTCTTATCATTTGGATATGTTATTT : 908
Ikl : TTTACATACTCGAATAAATAAAAATTTACAAGGTGAAAATTCCGCAATTGTGCCATCTATAGGCTTTCTTATCATTTGGATATGTTATTT : 902
Ipe : TTTACATACTCGAATAAATAAAAATTTGCAAGGTGTAAATTCCGCAATTGTGCCATCTATAGGCTTTCTTATCATTTGGATATGTTATTT : 902
Ili : TTTACATACTCGAATAAATAAAAATTTGCAAGGTGTAAATTCCGCAATTGTGCCATCTATAGGCTTTCTTATCATT-------------- : 903
Ath : TTTACATATTAAAACAAATAGGAATGTTAGGGGTATAAATTCTGCAATTGTGGCTTTGATCGGTTTTATTTTAATTTGGATATGCTATTT : 929

 1000 * 1020 * 1040 *
Isc : TGGAGTCAATCTATTAGGAATAGGATTACATAGTTATGGTTCATTTACATTAATGTCTAAT--- : 954
Ise : TGGAGTCAATCTATTAGGAATAGGGTTACATAGTTATGGTTCATTTACATTAACGTCTAAT--- : 903
Ici : TGGAGTCAATCTAGTAGGAATAGGATTACATAGTTATGGTTCATTTACATTAATGTCTAATTGA : 972
Ikl : TGGAGTCAATCTATTAGGAATAGGATTACATAGTTATGGTTCATTTACATTAACGTCTAAT--- : 963
Ipe : TGGAGTCAATCTATTAGGAATAGGATTACATAGTTATGGTTCATTTACATTAACGTCTAAT--- : 963
Ili : ---------------------------------------------------------------- : -
Ath : TGGCGTCAATCTTTTAGGAATAGGTTTACATAGTTATGGTTCATTTACATCGAATTAA------ : 987

**Figure S89 Multiple sequence alignment of the deduced nucleotide sequences of *ccs*A gene.** The origins of the nucleotide sequences used in this alignment are from: *Arabidopsis thaliana* (Ath), *Iodes klaineana* (Ikl), *Iodes cirrhosa* (Ici), *Iodes seretii* (Ise), *Iodes scandens* (Isc), *Iodes perrieri* (Ipe) and *Iodes liberica* (Ili). The nucleotide numbers for each sequence are indicated on the right. In the sequence alignment, identical nucleotides are shown with a black background, and similar residues are shown with a gray background.

* 20 * 40 * 60 * 80 *
Ici : ATGGCAAAAAAGAAAGCATTCACTCCTCTTTTATATCTTGCATCTATAGTATTTTTGCCCTGGTGTATTTCTCTCCCATTTAATAAAAGT : 90
Isc : ------------------------------------------------------------------------------------------ : -
Ikl : ATGGCAAAAAAGAAAGCATTCACTCCTCTTTTATATCTTGCATCTATAGTATTTTTGCCCTGGTGTATTTCTCTCCCATTTAATAAAAGT : 90
Ipe : ATGGAAAAAAAGAAAGCATTCATTCCTCTTTTATATCTTGCATCTATAGTATTTTTGCCCTGGTGTATTTCTCTCCCATTTAATAAAAGT : 90
Ili : ------------------------------------------------------------------------------------------ : -
Ise : ------------------------------------------------------------------------------------------ : -
Ath : ATGGCAAAAAAGAAAGCATTCATTCCTTTTTTTTATTTTCTATCTATAGTCTTTTTGCCCTGGTTGATCTCTCTCTGCTGTAATAAAAGT : 90

 100 * 120 * 140 * 160 * 180
Ici : TTTGAATCTTGGGTTACTAATTGGTGGGATACTAGGCAATCCGAAATTCTTTTGAATGATATTCAAGAAAAGAGTATTCTAGAAAAATTC : 180
Isc : ------------------------------------------------------------------------------------------ : -
Ikl : TTTGAATCTTGGGTTACTAATT------ATACTAGGCAATCCGAAATTCTTTTGAATGATATTCAAGAAAAGAGTATTCTAGAAAAATTC : 174
Ipe : TTTGAATCTTGGGTTCCTAATT------ATACTAGGCAATCCGAAATTCTTTTGAATGATATTCAAGAAAAGAGTATTCTAGAAAAATTC : 174
Ili : ------TCTTGGGTTACTAATT------ATACTAGGCAATCCGAAATTCTTTTGAATGATATTCAAGAAAAGAGTATTCTAGAAAAATTC : 78
Ise : ------------------------------------------------------------------------------------------ : -
Ath : TTGAAAACTTGGATTACTAATTGGTGGAATACTAGACAATGCGAAACTTTTTTGAATGATATTCAAGAAAAAAGTTTTCTAGAAAAATTC : 180

 * 200 * 220 * 240 * 260 *
Ici : ATAGAATTAGAGGAACTCCTCCTCTTGGAGGAAATAATCAAGGAATACTCGGAGACAGATCGACAAAAGTTTCGTATCGGAATCCACAAA : 270
Isc : ---------------------------------------------------------------------------------ATCCACAAA : 9
Ikl : ATAGAATTAGAGGAACTCCTCCTCTTGGACGAAATAATCAAGGAATACTCGGAGACAGATCGACAAAAGTTTCGTATCGGAATCCACAAA : 264
Ipe : ATAGAATTAGAGGAACTCCTCCTCTTGGACGAAATAATCAAGGAATACTCGGAGACAGATCGACAAAAGTTTCGTATCGGAATCCACAAA : 264
Ili : ATAGAATTAGAGGAACTCCTCCTCTTGGACGAAATAATCAAGGAATACTCGGAGACAGATCGACAAAAGTTTCGTATCGGAATCCACAAA : 168
Ise : ------------------------------------------------------------------------------------------ : -
Ath : ATACAATTAGAGGAACTATTCCAGCTCGATGAAATGATAAAGGAATACCCAGAAACCAATTTACAACAATTTCGTCTAGGAATCCACAAA : 270

280 * 300 * 320 * 340 * 360
Ici : GAAATGATCCAATTAATCAAGATATACAATGAGGATCATATTAATACGATTTTGCACTTCTCAACAAATATAATCTGCTTCGTTATTCTA : 360
Isc : GAAATGATCCAATTAATCAAGATATACAATGAGGATCATATTAATACGATTTTGCACTTCTCAACAAATATAATCTGCTTCGTTATTCTA : 99
Ikl : GAAATGATCCAATTAATCAAGATATACAATGAGGATCATATTAATACGATTTTGCACTTCTCAACAAATATAATCTGCTTCATTATTCTA : 354
Ipe : GAAATGATCCAATTAATCAAGATATACAATGAGGATCATATTAATACGATTTTGCACTTCTCAACAAATATAATCTGCTTCATTATTCTA : 354
Ili : GAAATGATCCAATTAATCAAGATATACAATGAGGATCATATTAATACGATTTTGCACTTCTCAACAAATATAATCTGCTTCATTATTCTA : 258
Ise : ------------------------------------------------------------------------------------------ : -
Ath : GAAACGATCCAATTCATCAAGATACACAATGAGTATAATATCCATACAATCTTGCACTTCTCGACAAATCTAATATCTTTCGTTATTCTA : 360

 * 380 * 400 * 420 * 440 *
Ici : AGCGGTTATTCTATTTTGGGTAATGACGAACTTGTTCTTCTTAACTCTTGGGTTCAGGAATTTCTATATAACTTAAGCGACACAATAAAA : 450
Isc : AGCGGTTATTCTATTTTGGGTAATGACGAACTTGTTCTTCTTAACTCTTGGGTTCAGGAATTTCTATATAACTTAAGCGACACAATAAAA : 189
Ikl : AGTGCTTATTCTATTTTGGGTAATGACGAACTTGTTCTTCTTAACTCTTGGGTTCAGGAATTTCTATATAACTTAAGCGACACAATAAAA : 444
Ipe : AGTGCTTATTCTATTTTGGGTAATGACGAACTTGTTCTTCTTAACTCTTGGGTTCAGGAATTTCTATATAACTTAAGCGACACAATAAAA : 444
Ili : AGTGCTTATTCTATTTTGGGTAATGACGAACTTGTTCTTCTTAACTCTTGGGTTCAGGAATTTCTATATAACTTAAGCGACACAATAAAA : 348
Ise : ------------------GGTAATGAGGAACTTGTTCTTCTTAACTCTTGGGTTCAGGAATTTCTATATAACTTAAGCGACACAATAAAA : 72
Ath : AGTGGTTATTCCTTTTGGGGTAAGGAAAAGCTTTTTATTCTGAATTCTTGGGTTCAAGAATTCCTATATAATTTAAGTGATACAATTAAA : 450

 460 * 480 * 500 * 520 * 540
Ici : GCTTTTTCTATTCTTTTATTAACTGATTTATGTATTGGATTCCATTCACCCCATGGTTGGGAACTAATGATTGGCTCTGTCTATAAAGAT : 540
Isc : GCTTTTTCTATTCTTTTATTAACTGATTTATGTATTGGATTCCATTCACCC--------------------------------------- : 240
Ikl : GCTTTTTCTATTCTTTTATTAACTGATTTATGTATCGGATTCCATTCACCCCATGGTTGGGAACTAATGATTGGCTCTGTCTATAAAGAT : 534
Ipe : GCTTTTTCTATTCTTTTATTAACTGATTTATGTATCGGATTCCATTCACCCCATGGTTGGGAACTAATGATTGGCTCTGTTTATAAAGAT : 534
Ili : GCTTTTTCTATTCTTTTATTAACTGATTTTTTTATCGGATTCCATTCACCCCATGGTTGGGAACTAATGATTGGCTCTGTCTATAAAGAT : 438
Ise : GCTTTTTCTATTCTTTTATTAACTGATTTATGTATCGGATTCCATTCACCCCATGGTTGGGAACTAATGATTGGCTCTGTCTATAAAGAT : 162
Ath : GCTTTTTCGATTCTTTTATTAACTGATTTATGTATCGGATTCCATTCGCCTCACGGTTGGGAACTAATGATTGGTTATATTTACAAAGAT : 540

* 560 * 580 * 600 * 620 *
Ici : TTTGGATTTGTTCATAATGATCAAATTATATCTGGTCTTGTTTCCACTTTTCCAGTCATTCTAGATACAATTTTAAAATATTGGATTTTC : 630
Isc : ------------------------------------------------------------------------------------------ : -
Ikl : TTTGGATTTGTTCATAATGATCAAATTATATCTGGTCTTGTTTCTACTTTTCCAGTCATTCTCGATACAATTTTAAAATATTGGATTTTC : 624
Ipe : TTTGGATTTGTTCATAATGATCAAATTATATCTGGTCTTGTTTCTACTTTTCCAGTCATTCTAGATACAATTTTAAAATATTGGATTTTC : 624
Ili : TTTGGATTTGTTCATAATGATCAAATTATATCTGGTCTTGTTTCTACTTTTCCAGTTATTCTAGATACAATTTTAAAATATTGGATTTTC : 528
Ise : TTTGGATTTGTTCATAATGATCAAATTATATCTGGTCTTGTTTCTACTTTTCCAGTCATTCTAGATACAATTTTAAAATATTGGATTTTC : 252
Ath : TTTGGGTTTGCTCATTATGAGCAAATTTTATCTGGTCTAGTTTCTACCTTTCCAGTAATTCTTGATACAATTTTTAAATATTGGATTTTT : 630

 640 * 660 * 680 *
Ici : CGCTATTTAAATCGTGTATCCCCGTCACTTGTAGTAATTTATCATTCAATGAATGAATGA : 690
Isc : ------------------------------------------------------------ : -
Ikl : CGCTATTTAAATCGTGTATCCCCGTCA--------------------------------- : 651
Ipe : CGCTATTTAAATCGTGTATCTCCGTCACTTGTAGTAATTTATCATTCAATG--------- : 675
Ili : CGCTATTTAAATCGTGTATCCCCGTCACTTGTAGTAATTTATCATTCAATGAATGAA--- : 585
Ise : CGCTATTTAAATCGTGTATCCCCGTCACTTGTAGTAATT--------------------- : 291
Ath : CGTTATTTAAATCGTGTATCTCCGTCACTTGTAGTGATTTATCATGCAATAAACGACTAA : 690

**Figure S90 Multiple sequence alignment of the deduced nucleotide sequences of *cem*A gene.** The origins of the nucleotide sequences used in this alignment are from: *Arabidopsis thaliana* (Ath), *Iodes klaineana* (Ikl), *Iodes cirrhosa* (Ici), *Iodes seretii* (Ise), *Iodes scandens* (Isc), *Iodes perrieri* (Ipe) and *Iodes liberica* (Ili). The nucleotide numbers for each sequence are indicated on the right. In the sequence alignment, identical nucleotides are shown with a black background, and similar residues are shown with a gray background.

* 20 * 40 * 60 * 80 *
Ili : ATGCCTGTTGGTGTTCCAAAAGTACCTTTCCGCAGTCCTGGAGAAGAAGATGCATCTTGGGTTGACATA------CGACTTTATCGAGAA : 84
Ise : ATGCCTGTTGGTGTTCCAAAAGTACCTTTCCGCAGTCCTGGAGAAGAAGATGCATCTTGGGTTGACATA---AACCGACTTTATCGAGAA : 87
Ikl : ATGCCTGTTGGTGTTCCAAAAGTACCTTTCCGCAGTCCTGGAGAAGAAGATGCATCTTGGGTTGACATA---AACCGACTTTATCGAGAA : 87
Ici : ATGCCTGTTGGTGTTCCAAAAGTACCTTTCCGCAGTCCTGGAGAAGAAGATGCATCTTGGGTTGACATA---AACCGACTTTATCGAGAA : 87
Ipe : ATGCCTGTTGGTGTTCCAAAAGTACCTTTCCGCAGTCCTGGAGAAGAAGATGCATCTTGGGTTGACATA---AACCGACTTTATCGAGAA : 87
Isc : ATGCCTGTTGGTGTTCCAAAAGTACCTTTCCGCAGTCCTGGAGAAGAAGATGCATCTTGGGTTGACATA---AACCGACTTTATCGAGAA : 87
Ath : ATGCCTATTGGCGTTCCAAAAGTACCTTTTCGAAGTCCTGGAGAAGGAGATACATCTTGGGTTGACATATACAACCGACTTTATCGAGAA : 90

 100 * 120 * 140 * 160 * 180
Ili : AGATTACTTTTTTTAGGACAAGCGGTTGATAGCGAG---------------ATCTCGAATCAACTTATTGGTCTTATGGTATATCTCAGT : 159
Ise : AGATTGCTTTTTTTAGGACAAGCGGTTGATAGCGAGGCGGTTGATAGCGAGATCTCGAATCAACTTATTGGTCTTATGGTATATCTCAGT : 177
Ikl : AGATTACTTTTTTTAGGACAAGCGGTTGATAGCGAG---------------ATCTCGAATCAACTTATTGGTCTTATGGTATATCTCAGT : 162
Ici : AGATTACTTTTTTTAGGACAAGCGGTTGATAGCGAG---------------ATCTCGAATCAACTTATTGGTCTTATGGTATATCTCAGT : 162
Ipe : AGATTACTTTTTTTAGGACAAGCGGTTGATAGCGAG---------------ATCTCGAATCAACTTATTGGTCTTATGGTATATCTCAGT : 162
Isc : AGATTACTTTTTTTAGGACAAGCGGTTGATAGCGAG---------------ATCTCGAATCAACTTATTGGTCTTATGGTATATCTCAGT : 162
Ath : AGATTATTTTTTTTAGGCCAAGAGGTTGATACCGAA---------------ATCTCGAATCAACTTATTAGTCTTATGATATATCTCAGT : 165

 * 200 * 220 * 240 * 260 *
Ili : ATCGAGGATGATACCAAAGATCTGCATTTGTTTATAAACTCTCCTGGTGGATGGGTAATACCTGGAGTAGGTATTTATGATACTATGCAA : 249
Ise : ATCGAGGATGATACCAAAGATCTGCATTTGTTTATAAACTCTCCTGGTGGATGGGTAATACCTGGAGTAGGTATTTATGATACTATGCAA : 267
Ikl : ATCGAGGATGATACCAAAGATCTGCATTTGTTTATAAACTCTCCTGGTGGATGGGTAATACCTGGAGTAGGTATTTATGATACTATGCAA : 252
Ici : ATCGAGGATGATACCAAAGATCTGCATTTGTTTATAAACTCTCCTGGTGGATGGGTAATACCTGGAGTAGGTATTTATGATACTATGCAA : 252
Ipe : ATCGAGGATGATACCAAAGATCTGCATTTGTTTATAAACTCTCCTGGTGGATGGGTAATACCTGGAGTAGGTATTTATGATACTATGCAA : 252
Isc : ATCGAGGATGATACCAAAGATCTGCATTTGTTTATAAACTCTCCTGGTGGATGGGTAATACCTGGAGTAGGTATTTATGATACTATGCAA : 252
Ath : ATAGAAAAGGATACCAAAGATCTTTATTTGTTTATAAACTCTCCTGGTGGATGGGTAATATCTGGAATGGCTATTTATGATACTATGCAA : 255

280 * 300 * 320 * 340 * 360
Ili : TTTGTGCGACCAGATGTACATACAATATGCATGGGATTAGCCGCTTCAATGGGATCTTTTATCTTGGTTGGAGGAGAAATTACCAAACGT : 339
Ise : TTTGTGCGACCAGATGTACATACAATATGCATGGGATTAGCCGCTTCAATGGGATCTTTTATCTTGGTTGGAGGAGAAATTACCAAACGT : 357
Ikl : TTTGTGCGACCAGATGTACATACAATATGCATGGGATTAGCCGCTTCAATGGGATCTTTTATCTTGGTTGGAGGAGAAATTACCAAACGT : 342
Ici : TTTGTGCGACCAGATGTACATACAATATGTATGGGATTAGCCGCCTCAATGGGATCTTTTATCTTGGTTGGAGGAGAAATTACCAAACGT : 342
Ipe : TTTGTGCGACCAGATGTACATACAATATGCATGGGATTAGCCGCTTCAATGGGATCTTTTATCTTGGTTGGAGGAGAAATTACCAAACGT : 342
Isc : TTTGTGCGACCAGATGTACATACAATATGCATGGGATTAGCCGCTTCAATGGGATCCTTTATCTTGGTTGGAGGAGAAATTACCAAACGT : 342
Ath : TTTGTGCGACCCGATGTACAGACAATATGCATGGGATTGGCCGCTTCAATAGCATCCTTTATCCTAGTCGGAGGAGCAATTACCAAACGT : 345

 * 380 * 400 * 420 * 440 *
Ili : CTAGCATTCCCTCACGCTAGGGTAATGATCCATCAACCTGCTAGTTCTTTTTACGAGTCACAAACGGGAGAATTTATCCTGGAAGCGGAA : 429
Ise : CTAGCATTCCCTCACGCTAGGGTAATGATCCATCAACCTGCTAGTTCTTTTTAGGAGTCACAAACGGGAGAATTTATCCTGGAAGCGGAA : 447
Ikl : CTAGCATTCCCTCACGCTAGGGTAATGATCCATCAACCTGCTAGTTCTTTTTACGAGTCACAAACGGGAGAATTTATCCTGGAAGCGGAA : 432
Ici : CTAGCATTCCCTCACGCTAGGGTAATGATCCATCAACCTGCTAGTTCTTTTTACGAGTCACAAACGGGAGAATTTATCCTGGAAGCGGAA : 432
Ipe : CTAGCATTCCCTCACGCTAGGGTAATGATCCATCAACCTGCTAGTTCTTTTTACGAGTCACAAACGGGAGAATTTATCCTGGAAGCGGAA : 432
Isc : CTAGCATTCCCTCACGCTAGGGTAATGATCCATCAACCCGCTAGTTCTTTTTACGAGTCACAAACGGGAGAATTTATCCTGGAAGCGGAA : 432
Ath : ATAGCATTCCCTCACGCTAGGGTAATGATCCATCAACCCGCTAGTTCGTTTTATGAGGCACAAACGGGAGAATTTATCTTGGAAGCGGAA : 435

 460 * 480 * 500 * 520 * 540
Ili : GAATTGCTGAAACTGCGTGAAACCCTAACAAGGGTTTATGTCCAAAGAACGGGCAAACCCTTATGGGTTGTATCCGAAGACATGGAAAGA : 519
Ise : GAATTGCTGAAACTGCGCGAAACCCTAACAAGGGTTTATGTCCAAAGAACGGGCAAACCCTTATGGCTTGTATCCGAAGACATGGAAAGA : 537
Ikl : GAATTGCTGAAACTGCGTGAAACCCTAACAAGGGTTTATGTCCAAAGAACGGGCAAACCCTTATGGGTTGTATCCGAAGACATGGAAAGA : 522
Ici : GAATTGCTGAAACTGCGTGAAACCCTAACAAGGGTTTATGTCCAAAGAACGGGCAAACCCTTATGGCTTGTATCCGAAGACATGGAAAGA : 522
Ipe : GAATTGCTTAAACTGCGTGAAACCCTAACAAGGGTTTATGTCCAACGAACGGGCAAACCGTTATGGCTTGTATCCGAAGACATGGAAAGA : 522
Isc : GAATTGCTGAAACTGCGTGAAACCCTAACAAGGGTTTATGTCCAAAGAACGGGCAAACCCTTATGGCTTGTATCCGAAGACATGGAAAGA : 522
Ath : GAATTACTTAAACTTCGCGAAACCATCACAAGGGTTTATGTACAAAGAACGGGCAAACCTATATGGGTTATATCCGAAGACATGGAACGG : 525

* 560 * 580 * 600
Ili : GATGTTTTTATGTCAGCAACAGAAGCACAAGCGTATGGAATTGTTGATCTTGTAGCGGTTGAA--- : 582
Ise : GATGTTTTTATGTCAGCAACAGAAGCACAAGCGTATGGAATTGTTGATCTTGTAGCGGTTGAA--- : 600
Ikl : GATGTTTTTATGTCAGCAACAGAAGCACAAGCGTATGGAATTGTTGATCTTGTAGCGGTTGAA--- : 585
Ici : GATGTTTTTATGTCAGCAACAGAAGCACAAGCGTATGGAATTGTTGATCTTGTAGCGGTTGAATGA : 588
Ipe : GATGTTTTTATGTCATCAACAGAAGCACAAGCGTATGGAATTGTTGATCTTGTAGCGGTTGAA--- : 585
Isc : GATGTTTTTATGTCAGCAACAGAAGCACAAGCGTATGGAATTGTTGATCTTGTAGCG--------- : 579
Ath : GATGTTTTTATGTCAGCAACAGAAGCCCAAGCTCATGGAATTGTTGATCTTGTAGCGGTTCAATAA : 591

**Figure S91 Multiple sequence alignment of the deduced nucleotide sequences of *clp*P gene.** The origins of the nucleotide sequences used in this alignment are from: *Arabidopsis thaliana* (Ath), *Iodes klaineana* (Ikl), *Iodes cirrhosa* (Ici), *Iodes seretii* (Ise), *Iodes scandens* (Isc), *Iodes perrieri* (Ipe) and *Iodes liberica* (Ili). The nucleotide numbers for each sequence are indicated on the right. In the sequence alignment, identical nucleotides are shown with a black background, and similar residues are shown with a gray background.

* 20 * 40 * 60 * 80
Ikl : ATGGAGGAATTCAAAAAATATTTACGGATAGATAAATCTCAACAACACGACTTCTTATATCCACTTATCTTTCAGGAGTCCACTTATAT : 89
Ise : ATGGAGGAATTCAAAAAATATTTACGGATAGATAAATCTCAACAACACGACTTCTTATATCCACTTATCTTTCAGGAGTCCACTTATAT : 89
Ipe : ATGGAGGAATTCAAAAAATATTTACGGATAGATAAATCTCAACAACATGACTTCTTATATCCACTTATCTTTCAGGAGTCCACTTATAT : 89
Ici : ATGGAGGAATTCAAAAAAAAATTACGGATAGATAGATTTCAACAACACGACTTCTTATATCCACTTATCTTTCAGGAGTCCACTTATAT : 89
Ath : ATGGATAAATTTCAAGGATATTTAGAGTTCGATGGGGCTCGGCAACAGAGTTTTCTATATCCACTTTTTTTTCGGGAGTATATTTATGT : 89
Ili : ATGGAGGAATTCAAAAAATATTTACGGATAGATAAATCTCAACAACACGACTTCTTATATCCACTTATCTTTCAGGAGTCCACTTATAT : 89
Isc : ----------------------------------------------------------------------------------------- : -

 * 100 * 120 * 140 * 160 * 180
Ikl : ACTTGCTCATGATCATGGTTTAAATAGATC---------------TATTTTGCTAGAAAATGGAGGTTATGGAAAAAAATTCAGCTTAC : 163
Ise : ACTTGCTCATGATCATGGTTTAAATAGATC---------------TATTTTGTTAGAAAATAGAGGTTATGACAAAAAATCCAGC---- : 159
Ipe : ACTTGCTCATGATCATGGTTTAAATAGATC---------------TATTTTGCTAGAAAATAGAGGTTATGAGAAAAAATCCAGCTTAC : 163
Ici : ACTTGCTCATAATCATGGTTTAAATAGATC---------------TATTTTGCTAGAAAATAGAGGTTATGACAATAAATCCAGCTTAC : 163
Ath : ACTTGCTTATGATCATGGTTTAAATAGATTAAATAGAAATCGCTATATTTTCTTGGAAAATGCGGATTATGACAAAAAATATAGTTCAC : 178
Ili : ACTTGCTCATGATCATGGTTTAAATAGATC---------------TATTTTGCTAGAAAATGGAGGTTATGGCAAAAAATCCAGCTTAC : 163
Isc : ----------------------------------------------------------------------------------------- : -

 180 * 200 * 220 * 240 * 260
Ikl : TAATTGTAAAACGTTTAATTACTCGAATGTATCAACAGAATCATTTGATTCCCTTTGCTAATGATTCTAACCAAAATCTATTTTTTGGA : 252
Ise : -----------------------------------CAGAATCATTTGATTCCCTTTGCTAATGATTCTAACCAAAATCAATTTTTTGGA : 213
Ipe : TAATTGTGAAACGTTTAATTACTCGAATGTATCAACAGAATCATTTGATTCCCTTTGCTAATGATTCTAATCAAAATCTATTTTTTGGA : 252
Ici : TAATTGTGAAACGTTTAATTACTCGAATGTATCAACAGAATCATTTGATTCCTTTTGCTAATGATTCTAACCAAAATCAATTTTTTGGA : 252
Ath : TAATTACGAAACGCTTAATTTTGCGAATGTATGAACAGAATCGTTTGATTATTCCCACTAAGGATGTGAACCAAAATTCCTTTTTGGGG : 267
Ili : TAATTGTAAAACGTTTAATTACTCGAATGTATCAACAGAATCATTTGATTCCCTTTGCTAATGATTCTAACCAAAATCTATTTTTTGGA : 252
Isc : ----------------------------------------------------------------------------------------- : -

* 280 * 300 * 320 * 340 *
Ikl : CGCAACAAGAATTTGTATTCGCAAATTATATCAGAAGGATTTGCAGTCATTGTGGAAATTCCATTTTCTCTACGATTAACGTCTTTCCT : 341
Ise : CGCAACAAGAATTTGTATTCGCAAATTATATCAGAAGGATTTGCAGTCATTGTGGAAATTCCATTTTCTCTACGATTAACGTCTTTCCT : 302
Ipe : CGCAACAAGAATTTGTATTTGCAAATTATATCAGAAGGATTTGCAGTCATTGTGGAAATTCCATTTTCTCTACGATTAACGTCTTTCCT : 341
Ici : CGCAACAAGAATTTGTATTCGAAAATTATATCAGAAGGATTTGCAGTCATTGTGGAAATTCCATTTTCTCTACGATTAACATCTTTCCT : 341
Ath : CATACCAGTCTTTTCTATTATCAAATGATATCTGTTTTATTTGCAGTGATTGTCGAAATTCCATTTTCCCTAAGATTAGGATCCTCTTT : 356
Ili : CGCAACAAGAATTTGTATTCGCAAATTATATCAGAAGGATTTGCAGTCATTGTGGAAATTCCATTTTCTCTAATA-------------- : 327
Isc : ----------------------------------------------------------------------------------------- : -

 360 * 380 * 400 * 420 * 440
Ikl : AGAAGGGAAAGATATAATACAATCTCATACTTTACGATCAATTCATTCAATATTTCCTTTTTTAGAGGACAACTTTTTACATTTAAATT : 430
Ise : AGAAGGGAAAGATATAATACAATCTCATACTTTACGATCAATTCATTCAATATTTCCTTTTTTAGAGGACAACTTTTTACATTTAAATT : 391
Ipe : AGAAGGGAAAGATATAATACAATCTCATACTTTACGATCAATTCATTCAATATTTCCTTTTTTAGAGGACAACTTTTTACATTTAAATT : 430
Ici : AGAAGGGAAAGATATAATACAATCTCATACTTTACGATCAATTCATTCAATATTTCCTTTTTTAGAGGACAACTTTTTACATTTAAATT : 430
Ath : TCAAGGAAAACAATTAAAAAAATCTTATAATTTACAATCAATTCATTCAATATTTCCCTTTTTAGAAGACAAATTAGGACATTTTAATT : 445
Ili : ---------------AATAGATTTTCGCAACTTACA--TACCTTAT-CAGAATTT----TTTAAAGTGACAAA-----ACCCTCAAGTT : 389
Isc : --------------------------------ATGGAGGAATTCAAAAAATATTT-----ACGGATAGATAGATCTCAACAACACGACT : 52

 * 460 * 480 * 500 * 520 *
Ikl : ATGCGTTAGATATACTAATACCCCACCCTATTCATCTGGAAATCTTGGTTCAAACTATTCGCTACTGGGTCAAAGATGCCTCTTCTTTG : 519
Ise : ATGCGTTAGATATACTAATACCCCACCCTATTCATCTGGAAATCTTGGTTCAAACTATTCGCTACTGGGTCAAAGATGCCTCTTCTTTG : 480
Ipe : ATGCGTTAGATATACTAATACCCTACCCTATTCATCTGGAAATCTTGGTTCAAACTATTCGCTACTGGGTCAAAGATGCCTCTTCTTTG : 519
Ici : ATGCGTTAGATATACGAATACCCCACCCTATTCATCTGGAAATCTTGGTTCAAACTATTCGCTACTGGGTAAAAGATGCCTCCTCCTTG : 519
Ath : ATGTGTTAGATGTACTAATACCTTACCCCATCCATCTAGAAATCTTGGTTCAAACCCTACGTTACCGGGTAAAAGATGCCTCTTCTTTG : 534
Ili : GTGCCATATACACCTTAACAATTCAGT-TGTCC-TCTCAACA-CTAGGATATGACA---------TGTATCAAAGAAATCTATTCCTTC : 466
Isc : ---TCTTATATCCACTTATCTTTCAGG-AGTCCACTTA-----TTTGGATCGAGCCA--------AGTATCACAACTTTTTTTACCCAC : 124

540 * 560 * 580 * 600 * 620
Ikl : CATT--TATTACGATTTTTTCTCCATGAATATGGTAATTTGACTAGTACTATTACTTCAAAGAAAGTCAGTTATTCTTTTTCAAAAGGA : 606
Ise : CATT--TATTACGATTTTTTCTCCATGAATATGGTAATTTGACTAGTACTATT---------------------------TCAAAAGGA : 540
Ipe : CATT--TATTACGATTTTTTCTCCATGAATATGGTAATTTGGCTAGTACTATTACTTCAAAGAAAGTCAGTTATTCTTTTTCAAAAGGA : 606
Ici : CATT--TATTACGATTCTTTCTCCATGAATATGGTGATTTGAATAGTCCTATTACTTCAAAGAAAGTCAGTTATTCTTTTTCAAAAAGA : 606
Ath : CATT--TTTTTCGGTTCTGTTTATACGAGTATTGTAATTGGAAGAATTTTTATATTAAAAAAAAA---------------TCAATTTTG : 606
Ili : CTTT--TGCTA-------ATTTACACC----TTGCAA--------ATTTTTAT------------------------------------ : 498
Isc : CTTTGATTTTTATGGAACAGACATATGAATATGGTAATTTGAATAGTCCTATTACTTCAAAGAAAGTCAGTTATTCTTTTTCAAAAAGA : 213

 * 640 * 660 * 680 * 700 *
Ikl : AATCACAGATTCTTCTTCTTTTTATATAATTCTTATATATGTGAATATGAATCCGTCTTTGTCTTTCTCTGTAATCAATCTTCTCATTT : 695
Ise : AATCAGAGATTCTTCTTCTTTTTATATAATTCTTATGTATGTCAATATGAATCCGTCTTTGTCTTTCTCTGTAATCAATCTTCTCATTT : 629
Ipe : A------GATTCTTCTTCTTTTTATATAATTCTTATGTATGTGAATATGAATCCGTCTTTGTCTTTCTCTGTAATCAATCTTCTCATTT : 689
Ici : AATCACAGATTCTTCTTCTTTTTATATAATTCTTATGTATGTGAATATGAATCCGTCTTTGTCTTTCTCTGTAATCAATCTTCTCATTT : 695
Ath : AATCCAAGATTTTTCTTGTTCTTATATAATTCTCATGTATGTGAATACGAATCCATCTTTTTTTTTCTACGCAAGCGGTCTTCGCATTT : 695
Ili : ---------------------TTATTCGAGTATTCTAGGAGGAATTACG----CTTACTTGTTTTTTAGTTCAAGTAGCTACGGGGTTC : 562
Isc : AATCACAGATTCTTCTTCTTTTTATATAATTCTTATGTATGTGAATATGAATCCGTCTTTGTCTTTCTCTGTAATCAATCTTCTCTTTT : 302

 720 * 740 * 760 * 780 * 800
Ikl : ACGATCAACATCCTCCGGGGCCTTTATTGAACGAATATATTTCTATCTAAAAATGGAATGTGTTGTAGAAGTCTTTTCTAAGGTTTCTC : 784
Ise : ACGATCAACATCCTACGGGGCCTTTATTGAACGAATATCTTTCTATCTAAAAATGGAATGTGTTGTAGAAGTCTTTTCT---------- : 708
Ipe : ACGATCAACATCCTCCGGGGCCTTTATTGAACGAATATATTTCTATCTAAAAATGGAATGTGTTGTAGAAGTCTTTTCTAAGGTTTCTC : 778
Ici : ACGATCAACATCCTCCGGGACCTTTATTGAACGAATATATTTCTATGTAAAAATAGAATGTGTTGTAGAAGTTTTTTCTAAGGTTTCTC : 784
Ath : ACGATCGACATCTTATGAAGTCCTTTTTGAGCGAATTGTATTCTATGGAAAAATACACCATTTTTTCAAAGTTTTTGTTAATAATTTTC : 784
Ili : GCTAT-GACATGTATTATGTTAGGTATCAAGGAAAATCAATTTTGGCTTCAAAAGGGACGTTTCTTT---------------------- : 628
Isc : ATTCT-----TTTTTCTAAAAAATTCCTATATGAAAGATCCTTTCATGTATTATGTTAGGTATCAAGGAAA------------------ : 368

* 820 * 840 * 860 * 880 *
Ikl : AGGCCAACCTATGGTTGTTCAAAGATCCTTTCATGTATTATGTTAGGTATCAAGGAAAATCTATTTTGGCTTCAAAAGGGACGTTTCTT : 873
Ise : ----------------------------------------------------------------------------------------- : -
Ipe : AGGCCAACCTATGGTTGTTCAAAGATCCTTTCATGTATTATGTTAGGTATCAAGGAAAATCTATTTTGGCTTCAAAAGGGACGTTTCTT : 867
Ici : AGGCCAACCTATGGTTGTTCAAAGATCCTTTCATGTATTATGTTAGGTATCAAGGAAAATCTATTTTGGCTTCAAAAGGGACGTTTCTT : 873
Ath : CGGCAATCCTAGGGTTGCTCAAGGATCCTTTCATACATTATGTTAGATATCACGGAAGATGCATTCTGGCAACAAAGGATACGCCGCTT : 873
Ili : ----------------------------------------------------------------------------------------- : -
Isc : ----------------------------------------------------------ATCGATTTTGGCTTCAAAAGGGACGTTTCTT : 399

 900 * 920 * 940 * 960 * 98
Ikl : TTGATGAAAAAATGGAAATATTACCTTGTAAATTTTTGTCAATCTTATTTTGATCTGTGGTCTAATCCGGGAAGGATCTATGTAAACCA : 962
Ise : ---------------------------------------------------------------------------------------CG : 710
Ipe : TTGATGAAAAAATGGAAATATTACCTTGTAAATTTTTGTCAATCTTATTTTGATCTGTGGTCTAATCCGGGAAGGATCTATATAAACCG : 956
Ici : TTGATGAATAAATGGAAATATTACCTTGTAAATTTTTGTCAATCTTATTTTGATCTGTGGTCTAATCCGGGAAGGATCCATATAAACCG : 962
Ath : CTGATGAATAAATGGAAATATTATTTTGTTAATTTATGGCAATGTTATTTTTCGGTATGGTTTCAATCGCAAAAGGTCAATATAAATCA : 962
Ili : -TGATGAAAAAATGGAAATATTACCTTGTAAATTTTTGTCAATCTTATTTTGATCTGTGGTCTAATCCGGGAAGGATCTATGTAAACCG : 716
Isc : TTGATGAATAAATGGAAATATTACCTTGTAAATTTTTGTCAATCTTATTTTCATCTGTGGTCTAATCCGGGAAGGATCTATATAAACCG : 488

 980 * 1000 * 1020 * 1040 * 1060
Ikl : ATTATGCAAACATTCCCTTGACTTTATGGGTTATCTTTCAAGTGTGCAACTAAACTCTTCAATCATACGGAGTCAAATGCTAGAAAATT : 1051
Ise : ATTATGCAAACATTCCCTTGACTTTATGGGTTATCTTTCAAGTGTGCAACTAAACTCTTCAATCATACGGAGTCAAATGCTAGAAAATT : 799
Ipe : ATTATGCAAACATTCCCTTGACTTTATGGGTTATCTTTCAAGTGTGCAACTAAACTCTTCAATCATACGGAGTCAAATGCTAGAAAATT : 1045
Ici : ATTATCCAAACATTCCCTTGACTTTATGGGTTATCTTTCAAGTGTGCAACTAAACTCTTCAATCATACGGAGTCAAATGCTAGAAAATT : 1051
Ath : ATTATCTAAAGATAATTTAGAGTTTCTGGGTTATCTGTCAAGTTTGCGATTAAACCCTTTAGTGGTACGTAGTCAAATGCTAGAAAACT : 1051
Ili : ATTATGCAAACATTCCCTTGACTTTATGGGTTACCTTTCAAGTGTGCAACTAAACTCTTCAATCATACGGAGTCAAATGCTAGAAAATT : 805
Isc : ATTATGCAAGCATTCCCTTGACTTTATGGGTTATCTTTCAAGTGTGCAACTAAACTCTTCAATCATACGGAGTCAAATGCTAGAAAATT : 577

* 1080 * 1100 * 1120 * 1140 * 1160
Ikl : CATTTCTAGTCGATAATGCTATTAAGAAGTTCGATACTATTCTTCCAATTATTACTCTGATTGGATCATTGGCTAAAGCGAAATTTTGT : 1140
Ise : CATTTCTAGTCGATAATGCTATTACGAAGTTTGATACTATTCTTCCAATTATTACTCTGATTGGATCATTGGCTAAAGCGAAATTTTGT : 888
Ipe : CATTTCTAGTCGATAATGCTATTAAGAAGTTCGATACTATTCTTCCAATTATTACTCTGATTGGATCATTGGCTAAAGCGAAATTTTGT : 1134
Ici : CATTTCTAGTCGATAATGCTATTAAGAAGTTCGATACTATTCTTCCAATTATTACTCTGATTGGATCATTGGCTAAAGCGAAATTTTGT : 1140
Ath : CATTTCTAATAGATAATGTTAGAATCAAATTGGATAGCAAAATTCCAATTTCTTCTATTATTGGATCGTTGGCTAAAGATAAATTTTGT : 1140
Ili : CATTTCTAGTCGATAATGCTATTAAGAAGTTCGATACTATTCTTCCAATTATTACTCTGATTGGATCATTGGCTAAAGCGAAATTTTGT : 894
Isc : CATTTCTAGTCGATAATGCTATTAAGAAGTTTGATACTATTCTTCCAATTATTACTCTGATTGGATCATTGGCTAAAGCGAAATTTTGT : 666

 1160 * 1180 * 1200 * 1220 * 1240
Ikl : AACACATTAGGGCATCCCATTAGTAAGCCGGTCCGGGCCCATTTATCTGATTCTGATATTATTGACCGATTTGGGCGTATATGCAGAAA : 1229
Ise : AACACATTAGGGCATCCCATTAGTAAGCCGGTCCGGGCCCATTTATCTGATTCTGATATTATTGACCGATTTGGGCGTATATGCAGAAA : 977
Ipe : AACACATTAGGGCATCCCATTAGTAAGCCGGTCCGGGCCCATTTATCAGATTCTGATATTATTGACCGATTTGGGCGTCTATGCAGAAA : 1223
Ici : AACACATTAGGGCATCCCATTAGTAAGCCGGTCCGGGCCCATTTATCTGATTCTGATATTATTGACCGATTTGGGCGTATATGCAGAAA : 1229
Ath : AATGTATTAGGGCATCCCATTAGTAAAGCAACCTGGACGGATTCATCAGATTCTGATATTCTCAACCGATTTGTGCGGATATGCAGAAA : 1229
Ili : AACACATTAGGGCATCCCATTAGTAAGCCGGTCCGGGCCCATTTATCTGATTCTGATATTATTGACCGATTTGGGCGTATATGCAGAAA : 983
Isc : AACACATTAGGGCATCCCATTAGTAAGCCGGTCCGGGCTCATTTATCTGATTCTGATATTATTGACCGATTTGGGCGTATATGCAGAAA : 755

 * 1260 * 1280 * 1300 * 1320 *
Ikl : TCTTTTTCATTATTATAGTGGATCCTCAAAAAAAAAGAGATTGTATCGAATAAAGTATATACTTCGACTTTCTTGTGCTAGAACTTTGG : 1318
Ise : TCTTTTTCATTATTATAGTGGATCCTCAAAAAAAAAGAGATTGTATCGAATAAAGTATATACTTCGACTTTCGTGTGCCAGAACTTTGG : 1066
Ipe : TCTTTTTCATTATTATAGTGGATCCTCAAAAAAAAAGAGATTGTATCGAATAAAGTATATACTTCGACTTTCGTGTGCCAGAACTTTGG : 1312
Ici : TCTTTTTCATTATTATAGTGGATCCTCAACAAAAAAGAGATTGTATCGAATAAAGTATATACTTCGACTTTCGTGTGCCAGAACTTTGG : 1318
Ath : TATTTCTCATTATTACAGCGGATCTTCAAAAAAAAAGAATTTGTATCGAATAAAATATATACTTCGTCTTTGTTGTGTTAAAACTTTGG : 1318
Ili : TCTTTTTCATTATTATAGTGGATCCTCAAAAAAAAAGAGATTGTATCGAATAAAGTATATACTTCGACTTTCGTGTGCCCGAACTTTGG : 1072
Isc : TCTTTTTCATTATTATAGTGGATCCTCAACAAAAAAGAGATTGTATCGAATAAAGTATATACTTCGACTTTCGTGTGCCAGAACTTTGG : 844

1340 * 1360 * 1380 * 1400 * 1420
Ikl : CTCGGAAACACAAAAGTACTGTACGTGCTTTTTTGAAA---------CCGGAATTTTTTGAAAAGTTCCTTACGGCGGAAGGGGAAGTT : 1398
Ise : CTCGAAAACACAAAAGTACTCTACGTGCTTTTTTGAAA---------CCGGAATTTTTGGAAAAGTTCCTTACGGCGGAAGGGGAAGTT : 1146
Ipe : CTCGAAAACACAAAAGTACTGTACGTGATTTTTTGAAA---------CCGGAATTTTTGGAAAAGTTCCTTACGGCGGAAGGGGAAGTT : 1392
Ici : CTCGAAAACACAAAAGTACTGTACGTGCTTTTTTGAAA---------CCGGAATTTTTGGAAAAGTTCCTTACGGCGGAAGGGGAAGTT : 1398
Ath : CTCGTAAACACAAAAGTACTGTACGCACTTTTTTAAAAAGGTTGGGCTCTGGTTTATTGGAAGAATTCCTTACGGGGGAAGACCAAGTT : 1407
Ili : CTCGAAAACACAAAAGTACTGTACGTGCTTTTTTGAAA---------CCGGAATTTTTTGAAAAGTTCCTTACGGCGGAAGGGGAAGTT : 1152
Isc : CTCGAAAACACAAAAGTACTGTACGTGCTTTTTTGAAA---------CCGGAATTTTTGGAAAAGTTCCTTACGGCGGAAGGGGAAGTT : 924

 * 1440 * 1460 * 1480 * 1500 *
Ikl : CTGTCTTTGACCTTTCCAATAGTTTCTTCTATTTTGCGGGGGTTATATAGAAGGCGAATTTGGTATTTGGATATTATTTGTATTAATGA : 1487
Ise : CTGTCTTTGACCTTTCCAATAGTTTCTTCTATTTTGCGGGGTTTATATAGAAGGCGAATTTGGTATTTGGATATTATTTGTATTAATGA : 1235
Ipe : CTGTCTTTGAACTTTCCAATAGTTTCTTCTATTTTGCGGGGGTTATATATAAGGCGAATTTGGTATTTGGATATTATTTGTATTAATGA : 1481
Ici : CTGTCTTTGACCTTTCCAATAGTTTCTTCTATTTTGCGGGGGTTATATAAAAGGCGGATTTGGTATTTGGATATTATTTGTATTAATGA : 1487
Ath : CTTTCTTTAATCTTCCCAAGAAGTTATTATGCTTCTAAAAGATTATATCGAGTGCGAATTTGGTATTTGGATATTCTTTATCTTAATGA : 1496
Ili : CTGTCTTTGACCTTTCCAATAGTTTCTTCTATTTTGCGGGGGTTATATAGAAGGCGAATTTGGTATTTGGATATTATTTGTATTAATGA : 1241
Isc : CTGTCTTTGACCTTTCCAATAGTTTCTTCTATTTTGCGGGGGTTATATAAAAGGCGGATTTGGTATTTGGATATTATTTTTATTAATGA : 1013

 1520 * 1540 * 1560 * 1580 * 1600
Ikl : TCTGGCCAATTATGAA------------------------------------------------------------------------- : 1503
Ise : TCTGGCCAATTATGAA------------------------------------------------------------------------- : 1251
Ipe : TCTGGCCAATTATGAA------------------------------------------------------------------------- : 1497
Ici : TCTGGCCAATTATGAATGA---------------------------------------------------------------------- : 1506
Ath : TTTGGTCAATCATGAATAA---------------------------------------------------------------------- : 1515
Ili : TCTGGCCAATTATGAACCTGGCAGAATGCATGTCAAGGCCAGGCCTAGGCTAACCATTGCTTTTGTTGGTAATACGATCGGAAGGAGCT : 1330
Isc : TCCGGCCAATTATGAAAATACGATCGGAAGGAGCTAATTCAAACCCCTCCGGTAAAATAAGAACA--GCTCCGACATTCAAAGCCCCTT : 1100

* 1620 * 1640 * 1660 * 1680
Ikl : -------------------------------------------------------------------------------------- : -
Ise : -------------------------------------------------------------------------------------- : -
Ipe : -------------------------------------------------------------------------------------- : -
Ici : -------------------------------------------------------------------------------------- : -
Ath : -------------------------------------------------------------------------------------- : -
Ili : AATTCAAACCCCTCCGGTAAAATAAGAACAGCCCCGACATTCAAAGCCCCTTTTTTACCATTAGCAAGAACTTGTTTCAGTTGCAT : 1416
Isc : TTTTACCATTAGCAAGAACTTGTTTCAGTTGCATTTCTATTCCACCTCTTAGAAAGAAAAGAGATTGTATCGAATAAAGTATATA- : 1185

**Figure S92 Multiple sequence alignment of the deduced nucleotide sequences of *mat*K gene.** The origins of the nucleotide sequences used in this alignment are from: *Arabidopsis thaliana* (Ath), *Iodes klaineana* (Ikl), *Iodes cirrhosa* (Ici), *Iodes seretii* (Ise), *Iodes scandens* (Isc), *Iodes perrieri* (Ipe) and *Iodes liberica* (Ili). The nucleotide numbers for each sequence are indicated on the right. In the sequence alignment, identical nucleotides are shown with a black background, and similar residues are shown with a gray background.

* 20 * 40 * 60 * 80
Ili : ATGAGAATTGATACAACAGAGGTACAAGCTATAAAGTCCTTTTCCAGATTGGAATTAGAATCCTTAAAAGAGGTCTATGGAATCATAGG : 89
Ipe : ATGATAATTGATACAACAGAGGTACAAGCTATAAAGTCTTTTTCCAGATTGGAATTAGAATCCTTAAAAGAGGTCTATGGAATCATAGG : 89
Ici : ATGATAATTGATACAACAGAGGTACAAGCTATAAAGTCTTTTTCCAGATTGGAATTAGAATCCTTAAAAGAGGTCTATGGAATCATAGG : 89
Ikl : ATGAGAATTGATACAACAGAGGTACAAGCTATAAAGTCCTTTTCCAGATTGGAATTAGAATCCTTAAAAGAGGTCTATGGAATCATAGG : 89
Ise : ATGATAATTGATACAACAGAGGTACAAGCTATAAAGTCTTTTTCCAGATTGGAATTCGAATCCTTAAAAGAGGTCTATGGAATCATAGG : 89
Ath : ATGATAATTTATGCAACAGCAGTCCAAACTATAAATTCTTTTGTTAAATTGGA------ATCTTTAAAAGAGGTCTATGGACTCATATG : 83
Isc : ATGATAATTGATACAACAGAGGTACAAGCTATAAAGTCTTTTTCCAGATTGGAATTAGAATCCTTAAAAGAGGTCTATGGAATCATAGG : 89

 * 100 * 120 * 140 * 160 * 180
Ili : GGGGCTTGTGCCTATTTTAACTCTTGTCTTAGGAATCACAATAGGTGTACTAGTAATTGTTTGGTTAGAAAGACAAATATCTGCGGGGA : 178
Ipe : GGGGCTTGTGCCTATTTTAACTCTTGTCTTAGGAATCACAATAGGTGTACTAGTAATTGTTTGGTTAGAAAGACAAATATCTGCGGGGA : 178
Ici : GGGGCTTGTCCCTATTTTAACTCTTGTCTTAGGAATTACAATAGGTGTACTAGTAATTGTTTGGTTAGAAAGAGAAATATCTGCGGGGA : 178
Ikl : GGGGCTTGTGCCTATTTTAACTCTTGTCTTAGGAATCACAATAGGTGTACTAGTAATTGTTTGGTTAGAAAGAGAAATATCTGCAGGGA : 178
Ise : GGGGCTTGTGCCTATTTTAACTCTTGTCTTAGGAATCACAATAGGTGTACTAGTAATTGTTTGGTTAGAAAGAGAAATATCTGCAGGGA : 178
Ath : GATATTTGTCCCTATATTTTCTCTTGTATTGGGAATCATAACAGGTGTACTAGTAATTGTGTGGTTAGAAAGAGAAATATCTGCAGGGA : 172
Isc : GGGGCTTGTGCCTATTTTAACTCTTGTCTTAGGAATTACAATAGGTGTACTAGTAATTGTTTGGTTAGAAAGAGAAA------------ : 166

 80 * 200 * 220 * 240 * 260
Ili : TACAACAACGTATTGGACCTGAATACGCCGGTCCTTTGGGAATTCTTCAAGTTCTAGCAGATGGTACAAAATTACTTCTCAAAGAGAAT : 267
Ipe : TACAACAACGTATTGGACCTGAATACGCCGGTCCTTTGGGAATTCTTCAAGTTCTAGCGGATGGTACAAAATTACTTCTCAAAGAGAAT : 267
Ici : TACAACAACGTATTGGACCTGAATACGCCGGTCCTTTGGGAATTCTTCAAGTTCTAGCAGATGGTACAAAATTACTTTTCAAAGAGAAT : 267
Ikl : TACAACAACGTATTGGACCTGAATACGCCGGTCCTTTGGGAATTCTTCAAGTTCTAGCAGATGGTACAAAATTACTTCTCAAAGAGAAT : 267
Ise : TACAACAACGTATTGGACCTGAATACGCCGGTCCTTTGGGAATTCTTCAAGTTCTAGCAGATGGTACAAAATTACTTCTCAAAGAGAAT : 267
Ath : TACAACAACGTATTGGACCTGAATACGCCGGCCCGTTGGGAATTCTTCAAGCTCTAGCCGATGGGACAAAACTACTTTTCAAAGAAAAT : 261
Isc : ------------------------------------------------------------------CAAAATTACTTTTCAAAGAGAAT : 189

* 280 * 300 * 320 * 340 *
Ili : CTTCTGCCATCTAGAGGAAATATCCCTTTATTCAGTATCGGACCATCCATAGCAGTCATATCAATTTTACTAAGTTATTTAGTAATTCC : 356
Ipe : CTTCTGCCATCTAGAGGAAATATCCCTTTATTCAGTATCGGACCATCCATAGCAGTCATATCAATTTTACTAAGTTATTTAGTAATTCC : 356
Ici : CTTCTGCCATCTAGAGGAAATATCCCTTTATTCAGTATCGGACCATCCATAGCAGTCATATCAATTTTACTAAGTTATTTAGTAATTCC : 356
Ikl : CTTCTGCCATCTAGAGGAAATATCCCTTTATTCAGTATCGGACCATCCATAGCAGTCATATCAATTTTACTAAGTTATTTAGTAATTCC : 356
Ise : CTTCTGCCATCTAGAGGAAATATCCCTTTATTCAGTATCGGACCATCCATAGCAGTCATATCAATTTTACTAAGTTATTTAGTAATTCC : 356
Ath : CTTCGTCCATCTAGAGGAAATACTCCTTTATTTAGTATTGGACCATCTATAGCAGTTATCTCTATTTTACTAAGTTATTCAGTAATTCC : 350
Isc : CTTCTGCCATCTAGAGGAAATATCCCTTTATTCAGTATCGGACCATCCATAGCAGTCATATCAATTTTACTAAGTTATTTAGTAATTCC : 278

 360 * 380 * 400 * 420 * 440
Ili : TTTTGGCTATCACCTTGTTCTAGCCGATCTCAGTATTGGTCTTTTTTTATGGATCGCCATTTCAAGTATTGCTCCTGTTGGACTTCTTA : 445
Ipe : TTTTGGCTATCACCTTGTTCTAGCCGATCTCAGTATTGGTCTTTTTTTATGGATCGCCATTTCAAGTATTGCTCCTGTTGGACTTCTTA : 445
Ici : TTTTGGCTATCACCTTGTTCTAGCCAATCTCAGTATTGGTCTTTTTTTATGGATCGCCATTTCAAGTATTGCTCCTGTTGGACTTCTTA : 445
Ikl : TTTTGGCTATCACCTTGTTCTAGCCGATCTCAGTATTGGTCTTTTTTTATGGATCGCCATTTCAAGTATTGCTCCTGTTGGACTTCTTA : 445
Ise : TTTTGGCTATCACCTTGTTCTAGCCGATCTCAGTATTGGTCTTTTTTTATGGATCTCCATTTCAAGTATTGCTCCTGTTGGACTTCTTA : 445
Ath : CTTTAGCAATCACCTTGTTTTAGCGGATCTCAATATCGGTATTTTTTTATGGATTGCCATCTCAAGTATTGCTCCTATTGGACTTCTTA : 439
Isc : TTTTGGCTATCACCTTGTTCTAGCCGATCTCAGTATTGGTCTTTTTTTATGGATCGCCATTTCAAGTATTGCTCCTGTTGGACTTCTTA : 367

 * 460 * 480 * 500 * 520 *
Ili : TGTCAGGATATGGATCAAATAATAAATATTCCTTTTTAGGTGGTCTCCGAGCTGCTGCTCAATCAATTAGTTATGAACTACCATTAACT : 534
Ipe : TGTCAGGATATGGATCAAATAATAAATATTCCTTTTTAGGTGGTCTCCGAGCTGCTGCTCAATCAATTAGTTATGAACTACCATTAACT : 534
Ici : TGTCAGGATATGGATCAAATAATAAATATTCCTTTTTAGGTGGTCTCCGAGCTGCTGCTCAATCAATTAGTTATGAACTACCATTAACT : 534
Ikl : TGTCAGGATATGGATCAAATAATAAATATTCCTTTTTAGGTGGTCTCCGAGCTGCTGCTCAATCAATTAGTTATGAACTACCATTAACT : 534
Ise : TGTCAGGATATGGATCAAATAATAAATATTCCTTTTTAGGTGGTCTCCGAGCTGCTGCTCAATCAATTAGTTATGAACTACCATTAACT : 534
Ath : TGTCAGGATATGGATCAAATAATAAATATTCTTTTTTAGGTGGTCTGCGAGCTGCTGCCCAATCGATTAGTTATGAAATACCATTAACT : 528
Isc : TGTCAGGATATGGATCAAATAATAAATATTCCTTTTTAGGTGGTCTCCGAGCTGCTGCTCAATCAATTAGTTATGAACTACCATTAACT : 456

540 * 560 * 580 * 600 * 620
Ili : TTATGTGTATTATCAATATCTCTA------------------AC--------------------------------------------- : 560
Ipe : TTATGTGTATTATCAATATCTCTA------------------ACAGTTGATATAGTTGAAGCACAGTCAAAATTTGGTTGTTGGGGATG : 605
Ici : TTATGTGTATTATCAATATCTCTACTATCTAACAGTTCAAGTACAGTTGATATAGTTGAAGCACAGTCAAAATTTGGTTTTTGGGGATG : 623
Ikl : TTATGTGTATTATCAATATCTCTATTATCTAACAGTTCAAGTACAGTTGATATAGTTGAAGCACAGTCAAAATTTGGTTTTTGGGGATG : 623
Ise : TTATGTGTATTATCAATATCTCTATTATCTAACAGTTCAAGTACAGTTGATATAGTTGAAGCACAGTCAAAATTTGGTTTTTGGGGATG : 623
Ath : CTATGTGTTTTATCAATATCTCTACTATCTAACAGTTTAAGTACAGTTGATATAGTTGAGGCACAATCAAAATATGGTTTTTGGGGATG : 617
Isc : TTATGTGTATTATCAATATCTCTATTATCTAACAGTTCAAGTACAGTTGATATAGTTGAAGCACAGTCAAAATTTGGTTTTTGGGGATG : 545

 * 640 * 660 * 680 * 700 *
Ili : ----------------------------------------------------------------------------------------- : -
Ipe : GAATTTGTGGCGTCAACCTATAGGGTTTATCGTTTTTCTAATTTCTTCCCTAGCCGAGTGTGAGAGATTACCTTTTGATTTACCAGAAG : 694
Ici : GAATTTGTGGCGTCAACCTATAGGGTTTATCGTTTTTCTAATTTCTTCCCTAGCAGAGTGTGAGAGATTACCTTTTGATTTACCAGAAG : 712
Ikl : GAATTTGTGGCGTCAACCTATAGGGTTTATCGTTTTTCTAATTTCTTCCCTAGCCGAGTGTGAGAGATTACCTTTTGATTTACCAGAAG : 712
Ise : GAATTTGTGGCGTCAACCTATAGGGTTTATCGTTTTTCTAATTTCTTCCCTAGCCGAGTGTGAGAGATTACCTTTTGATTTACCAGAAG : 712
Ath : GAATTTGTGGCGTCAACCTATAGGTTTTATCATTTTTCTAATTTCTTCCCTAGCAGAATGCGAGAGGTTACCGTTTGATTTACCAGAAG : 706
Isc : GAATTTGTGGCGTCAACCTATAGGGTTTATCGTTTTTCTAATTTCTTCCCTAGCAGAGTGTGAGAGATTACCTTTTGATTTACCAGAAG : 634

 720 * 740 * 760 * 780 * 800
Ili : -------------------------------CGAATATTCAGGTATCAAATTTGGTTTATTTTACATTGCTTCATATTTGAATCTACTT : 618
Ipe : CAGAAGAAGAATTAGTAGCAGGTTATCAAACCGAATATTCAGGTATCAAATTTGGTTTATTTTACATTGCTTCATATTTGAATCTACTT : 783
Ici : CAGAAGAAGAATTAGTAGCAGGTTATCAAACCGAATATTCAGGTATCAAATTTGGTTTATTTTACATTGCTTCATATTTGAATCTACTT : 801
Ikl : CAGAAGAAGAATTAGTAGCAGGTTATCAAACCGAATATTCAGGTATCAAATTTGGTTTATTTTACATTGCTTCATATTTGAATCTACTT : 801
Ise : CAGAAGAAGAATTAGTAGCAGGTTATCAAACCGAATATTCAGGTATCAAATTTGGTTTATTTTACATTGCTTCATATTTGAATCTACTT : 801
Ath : CGGAAGAAGAATTAATAGCAGGTTATCAAACTGAATATTCAGGTATAAAATTTGGTTTATTTTACGTTGCTTCTTATCTAAATCTATTA : 795
Isc : CAGAAGAAGAATTAGTAGCAGGTTATCAAACCGAATATTCAGGTATCAAATTTGGTTTCTCTTGTTTGAGACAAGTCATAACAAGATGG : 723

* 820 * 840 * 860 * 880 *
Ili : GTTTCTTCATTATTTGTAACAGTTCTTTACTTAGGA---GGTTGGAATCTTTCTATTCCGTACATATTTGTTCCCGGTCTTTTGGACAT : 704
Ipe : GTTTCTTCATTATTTGTAACAGTTCTTTACTTGGGA---GGTTGGAATCTTTCTATTCCGTACATATTTGTTCCCGGTCTTTTGGACAT : 869
Ici : GTTTCTTCATTATTTGTAACAGTTCTTTACTTGGGA---GGTTGGAATCTTTCTATTCCGTACATATTTGTTCCCGGGCTTTTGGACAT : 887
Ikl : GTTTCTTCATTATTTGTAACAGTTCTTTACTTAGGA---GGTTGGAATCTTTCTATTCCGTACATATTTGTTCCCGGTCTT-------- : 879
Ise : GTTTCTTCA------------------------GCA---GGTAGA-------------------------------------------- : 819
Ath : ATTTCCTCATTATTTGTAACAGTTCTATACTTAGGC---GGTTGGAATATTTCTATTCCGTATATATCTATTCTGGAGCTATT------ : 875
Isc : ACTTTACCAAGGCTCAGAATGGACCAACTATTAAATCTTGGATGGAA-ATTTCTTTTACCTATTTCTCTAGGTAAT------------- : 798

 900 * 920 * 940 * 960 * 98
Ili : AAATAAGGCAGGTAGAGTCTTTGGAATAATAATTGGTATTTTTATTACATTAGCTAAAACTTATTTGTTCTTGTGCATTTCTATCACAA : 793
Ipe : AAATAAGGCGGGTAGAGTCTTTGGAATAATAATTGGTATTTTTATTACATTAGCTAAAACTTATTTGTTCTTGTGCATTTCTATCACAA : 958
Ici : AAATAAGGCAGGTAGAGTCTTTGGAATAATAATTGGTATTTTTATTACATTAGCTAAAACTTATTTGTTCTTGTGCATTTCTATCACAA : 976
Ikl : ----------------GTCTTTGGAATAATACTTGGTATTTTTATTACATTAGCTAAAACTTATTTGTTCTTGTGCATTTCTATAACAA : 952
Ise : ----------------GTCTTTGGAATAATAATTGATATTTTTATTACATTAGCTAAAACTTATTTGTTCTTGTGCATTTCTATCACAA : 892
Ath : ---TCAAAGGGATCAAATTTTTGGAACAACAATTGGTATCTTTATTACATTAGCTAAAACTTATTTGTTTTTGTTCGTTTCTATCGCAA : 961
Isc : ----------------------------------------------------------------------------------------- : -

 0 * 1000 * 1020 * 1040 * 1060
Ili : CAAGATGGACTTTACCAAGGCTCAGAATGGACCAACTATTAAATCTTGGATGGAAATTTCTTTTACCTATTTCTCTAGGTAAT------ : 876
Ipe : CAAGATGGACTTTACCAAGGCTCAGAATGGACCAACTATTAAACCTTGGATGGAAATTTCTTTTACCTATTTCTCTAGGTAAT------ : 1041
Ici : CAAGATGGACTTTACCAAGGCTCAGAATGGACCAACTATTAAATCTTGGATGGAAATTTCTTTTACCTATTTCTCTAGGTAATCTATTA : 1065
Ikl : CAAGATGGACTTTACCAAGGCTCAGAATGGACCAACTATTAAATCTTGGATGGAAATTTCTTTTACCTATTTCTCTAGGTAAT------ : 1035
Ise : CAAGATGGACTTTACCAAGGCTCAGAATGGACCAACTATTAAATCTTGGATGGAAATTT------------------------------ : 951
Ath : CAAGATGGACTTTACCTAGGCTAAGAATGGATCAACTATTAAATCTTGGATGGAAATTTCTTTTACCTATTTCCCTTGGTAATCTATTA : 1050
Isc : ----------------------------------------------------------------------------------------- : -

* 1080 * 1100
Ili : --------------------------------- : -
Ipe : --------------------------------- : -
Ici : TTAACAACGTCTTCACAACTTTTTTCACTGTAA : 1098
Ikl : --------------------------------- : -
Ise : --------------------------------- : -
Ath : TTAACCACTTCTTTCCAACTCTTTTCACTCTAA : 1083
Isc : --------------------------------- : -

**Figure S93 Multiple sequence alignment of the deduced nucleotide sequences of *ndh*A gene.** The origins of the nucleotide sequences used in this alignment are from: *Arabidopsis thaliana* (Ath), *Iodes klaineana* (Ikl), *Iodes cirrhosa* (Ici), *Iodes seretii* (Ise), *Iodes scandens* (Isc), *Iodes perrieri* (Ipe) and *Iodes liberica* (Ili). The nucleotide numbers for each sequence are indicated on the right. In the sequence alignment, identical nucleotides are shown with a black background, and similar residues are shown with a gray background.

* 20 * 40 * 60 * 80
Ath : ATGATCTGGCATGTACAGAATGAAAATTTCATTCTCGATTCTACGAGAATTTTTATGAAAGCCTTTCATTTGCTTCTCTTCGATGGAAG : 89
Ikl : ATGATCTGGCATGTACAGAATGAAAACGTCATTCTCGATTCTACGAGAATTTTTATGAAAGCCTTTCATTTGCTTCTCTTCGATGGAAG : 89
Ili : ATGATCTGGCATGTACAGAATGAAAACGTCATTCTCGATTCTACGAGAATTTTTATGAAAGCCTTTCATTTGCTTCTCTTCGATGGAAG : 89
Ipe : ATGATCTGGCATGTACAGAATGAAAACGTCATTCTCGATTCTACGAGAATTTTTATGAAAGCCTTTCATTTGCTTCTCTTCGATGGAAG : 89
Ise : ATGATCTGGCATGTACAGAATGAAAACGTCATTCTCGATTCTACGAGAATTTTTATGAAAGCCTTTCATTTGCTTCTCTTCGATGGAAG : 89
Ici : ATGATCTGGCATGTACAGAATGAAAACGTCATTCTCGATTCTACGAGAATTTTTATGAAAGCCTTTCATTTGCTTCTCTTCGATGGAAG : 89
Isc : ATGATCTGGCATGTACAGAATGAAAACGTCATTCTCGATTCTACGAGAATTTTTATGAAAGCCTTTCATTTGCTTCTCCTCGATGGAAG : 89

 * 100 * 120 * 140 * 160 * 180
Ath : TTTTATTTTCC------------------------------CAGAATGTATCCTAATTTTTGGCCTAATTCTTCTTCTGATGATCGATT : 148
Ikl : TTTTATTCTCC------------------------------CAGAATGTATCCTAATTTTTGGCCTAATTCTTCTTCTGATGATCGATT : 148
Ili : TTTTATTCTCC------------------------------CAGAATGTATCCTAATTTTTGGCCTAATTCTTCTTCTGATGATCGATT : 148
Ipe : TTTTATTCTCC------------------------------CAGAATGTATCCTAATTTTTGGCCTAATTCTTCTTCTGATGATCGATT : 148
Ise : TTTTATTCTCC------------------------------CAGAATGTATCCTAATTTTTGGCCTAATTCTTCTTCTGATGATCGATT : 148
Ici : TTTTATTCTCC------------------------------CAGAATGTATCCTAATTTTTGGCCTAATTCTTCTTCTGATGATCGATT : 148
Isc : TTTTATTCTCCTCTTTCTTGTTGAAGTCCCTATTATTCTCCCAGAATGTATCCTAATTTTTGGCCTAATTCTTCTTCTGATGATCGATT : 178

 80 * 200 * 220 * 240 * 260
Ath : CAACCTCTGATCAAAA---AGATATACCTTGGTTATATTTCATCTCTTCAACAAGTTTCGTAATGAGCATAACGGCCCTATTGTTCCGA : 234
Ikl : CAACCTCTGATCAAAA---AGACATACCTTGGTTCTATTTCATCTCTTCAACAAGTTTAGTAATGAGCATAACGGCCCTATTGTTCCGA : 234
Ili : CAACCTCTGATCAAAA---AGACATACCTTGGTTCTATTTCATCTCTTCAACAAGTTTAGTAATGAGCATAACGGCCCTATTGTTCCGA : 234
Ipe : CAACCTCTGATCAAAA---AGACATACCTTGGTTCTATTTCATCTCTTCAACAAGTTTAGTAATGAGCATAACTGCCCTATTGTTCCGA : 234
Ise : CAACCTCTGATCAAAA---AGACATACCTTGGTTCTATTTCATCTCTTCAACAAGTTTAGTAATGAGCATAACGGCCCTATTGTTCCGA : 234
Ici : CAACCTCTGATCAAAA---AGACATACCTTGGTTCTATTTCATCTCTTCAACAAGTTTAGTAATGAGCATAACGGCCCTATTGTTCCGA : 234
Isc : CAACCTCTGATAAAAAGAAAGACATACCTTGGTTCTATTTCATCTCTTCAACAAGTTTAGTAATGAGCATAACGGCCCTATTGTTCCGA : 267

* 280 * 300 * 320 * 340 *
Ath : TGGAGAGAAGAACCTATGATTAGCTTTTCAGGAAATTTCCAAACGAACAATTTCAACGAAATCTTTCAATTTCTTATTTTACTATGTTC : 323
Ikl : TGGAGAGAAGAACCTATGATTAGCTTTTCGGGAAATTTCCAAACGAACAATTTCAACGAAATCTTTCAATTTCTTATTTTACTATGTTC : 323
Ili : TGGAGAGAAGAACCTATGATTAGCTTTTCGGGAAATTTCCAAACGAACAATTTCAACGAAATCTTTCAATTTCTTATTTTACTATGTTC : 323
Ipe : TGGAGAGAAGAACCTATGATTAGCTTTTCGGGAAATTTCCAAACGAACAATTTCAACGAAATCTTTCAATTTCTTATTTTACTATGTTC : 323
Ise : TGGAGAGAAGAACCTATGATTAGCTTTTCGGGAAATTTCCAAACGAACAATTTCAACGAAATCTTTCAATTTCTTATTTTACTATGTTC : 323
Ici : TGGAGAGAAGAACCTATGATTAGCTTTTCGGGAAATTTCCAAACGAACAATTTCAACGAAATCTTTCAATTTCTTATTTTACTATGTTC : 323
Isc : TGGAGAGAAGAACCTATGATTAGCTTTTCGGGAAATTTCCAAACGAACAATTTCAACGAAATCTTTCAATTTCTTATTTTACTATGTTC : 356

 360 * 380 * 400 * 420 * 440
Ath : AACTCTCTGTATTCCTCTATCCGTAGAGTACATTGAATGTACAGAAATGGCTATAACAGAGTTTCTGTTATTCATATTAACAGCTACTC : 412
Ikl : AACTCTATGTATTCCTCTATCCGTAGAGTACATTGAATGTACAGAAATGGCTATAACAGAGTTTCTCTTATTCGTATTAACAGCTACTC : 412
Ili : AACTCTATGTATTCCTCTATCCGTAGAGTACATTGAATGTACAGAAATGGCTATAACAGAGTTTCTCTTATTCGTATTAACAGCTACTC : 412
Ipe : AACTCTATGTATTCCTCTATCCGTAGAGTACATTGAATGTACAGAAATGGCTATAACAGAGTTTCTCTTATTCGTATTAACAGCTACTC : 412
Ise : AACTCTATGTATTCCTCTATCCGTAGAGTACATTGAATGTACAGAAATGGCTATAACAGAGTTTCTCTTATTCGTATTAACAGCTACTC : 412
Ici : AACTCTATGTATTCCTCTATCCGTAGAGTACATTGAATGTACAGAAATGGCTATAACAGAGTTTCTCTTATTCGTATTAACAGCTACTC : 412
Isc : AACTCTATGTATTCCTCTATCCGTAGAGTACATTGAATGTACAGAAATGGCTATAACAGAGTTTCTCTTATTCGTATTAACAGCTACTC : 445

* 460 * 480 * 500 * 520 *
Ath : TAGGAGGAATGTTTTTATGTGGTGCTAACGATTTAATAACTATCTTTGTAGCTCCAGAATGTTTCAGTTTATGCTCCTACCTATTATCT : 501
Ikl : TAGGAGGAATGTTTTTATGCGGTGCTAATGATTTAATAACTATCTTTGTAGCTCCAGAATGTTTCAGTTTATGCTCCTACCTATTATCT : 501
Ili : TAGGAGGAATGTTTTTATGCGGTGCTAATGATTTAATAACTATCTTTGTAGCTCCAGAATGTTTCAGTTTATGCTCCTACCTATTATCT : 501
Ipe : TAGGAGGAATGTTTTTATGCGGTGCTAATGATTTAATAACTATCTTTGTAGCTCCAGAATGTTTCAGTTTATGCTCCTACCTATTATCT : 501
Ise : TAGGAGGAATGTTTTTATGCGGTGCTAATGATTTAATAACTATCTTTGTAGCTCCAGAATGTTTCAGTTTATGCTCCTACCTATTATCT : 501
Ici : TAGGAGGAATGTTTTTATGCGGTGCTAATGATTTAATAACTATCTTTGTAGCTCCAGAATGTTTCAGTTTATGCTCCTACCTATTATCT : 501
Isc : TAGGAGGAATGTTTTTATGCGGTGCTAATGATTTAATAACTATCTTTGTAGCTCCAGAATGTTTCAGTTTATGCTCCTACCTATTATCT : 534

540 * 560 * 580 * 600 * 620
Ath : GGATATACCAAGAAAGATATACGATCTAATGAGGCTACTATGAAATATTTACTCATGGGTGGGGCAAGCTCTTCTATTCTGGTTCATGG : 590
Ikl : GGATATACCAAGAAAGATGTACGGTCTAATGAGGCTACTATGAAATATTTACTCATGGGCGGGGCAAGCTCTTCTATTCTGGTTCATGG : 590
Ili : GGATATACCAAGAAAGATGTACGGTCTAATGAGGCTACTATGAAATATTTACTCATGGGCGGGGCAAGCTCTTCTATTCTGGTTCATGG : 590
Ipe : GGATATACCAAGAAAGATGTACGGTCTAATGAGGCTACTATGAAATATTTACTCATGGGCGGGGCAAGCTCTTCTATTCTGGTTCATGG : 590
Ise : GGATATACCAAGAAAGATGTACGGTCTAATGAGGCTACTATGAAATATTTACTCATGGGCGGGGCAAGCTCTTCTATTCTGGTTCATGG : 590
Ici : GGATATACCAAGAAAGATGTACGGTCTAATGAGGCTACTATGAAATATTTACTCATGGGCGGGGCAAGCTCTTCTATTCTGGTTCATGG : 590
Isc : GGATATACCAAGAAAGATGTACGGTCTAATGAGGCTACTATGAAATATTTACTCATGGGCGGGGCAAGCTCTTCTATTCTGGTTCATGG : 623

 * 640 * 660 * 680 * 700 *
Ath : TTTCTCTTGGCTATATGGTTCATCCGGGGGAGAGATTGAGCTTCAAGAAATAGTGAATGGTCTTATCAATACACAAATGTATAACTCCC : 679
Ikl : TTTCTCTTGGCTATATGGTTCATCCGGGGGAGAGATCGAGCTTCAAGAAATAGTGAATGGTCTTATCAATACACAAATGTATAACTCCC : 679
Ili : TTTCTCTTGGCTATATGGTTCATCCGGGGGAGAGATCGAGCTTCAAGAAATAGTGAATGGTCTTATCAATACACAAATGTATAACTCCC : 679
Ipe : TTTCTCTTGGCTATATGGTTCATCCGGGGGAGAGATCGAGCTTCAAGAAATAGTGAATGGTCTTATCAATACACAAATGTATAACTCCC : 679
Ise : TTTCTCTTGGCTATATGGTTCATCCGGGGGAGAGATCGAGCTTCAAGAAATAGTGAATGGTCTTATCAATACACAAATGTATAACTCCC : 679
Ici : TTTCTCTTGGCTATATGGTTCATCCGGGGGAGAGATCGAGCTTCAAGAAATAGTGAATGGTCTTATCAATACACAAATGTATAACTCCC : 679
Isc : TTTCTCTTGGCTATATGGTTCATCCGGGGGAGAGATCGAGCTTCAAGAAATAGTGAATGGTCTTATCAATACACAAATGTATAACTCCC : 712

 720 * 740 * 760 * 780 * 800
Ath : CAGGAATTTCAATTGCGCTTATATTCATCACTGTAGGAATTGGGTTCAAGCTTTCCCTAGCCCCTTCTCATCAATGGACTCCTGACGTA : 768
Ikl : CAGGAATTTCAATTGCGCTCATATTCATCACTGTAGGAATTGGGTTCAAGCTTTCCCTAGCCCCTTCTCATCAATGGACTCCTGACGTA : 768
Ili : CAGGAATTTCAATTGCGCTCATATTCATCACTGTAGGAATTGGGTTCAAGCTTTCCCTAGCCCCTTCTCATCAATGGACTCCTGACGTA : 768
Ipe : CAGGAATTTCAATTGCGCTCATATTCATCACTGTAGGAATTGGGTTCAAGCTTTCCCTAGCCCCTTCTCATCAATGGACTCCTGACGTA : 768
Ise : CAGGAATTTCAATTGCGCTCATATTCATCACTGTAGGAATTGGGTTCAAGCTTTCCCTAGCCCCTTCTCATCAATGGACTCCTGACGTA : 768
Ici : CAGGAATTTCAATTGCGCTCATATTCATCACTGTAGGAATTGGGTTCAAGCTTTCCCTAGCCCCTTCTCATCAATGGACTCCTGACGTA : 768
Isc : CAGGAATTTCAATTGCGCTCATATTCATCACTGTAGGAATTGGGTTCAAGCTTTCCCTAGCCCCTTCTCATCAATGGACTCCTGACGTA : 801

* 820 * 840 * 860 * 880 *
Ath : TACGAAGGA-------------------------------------------------------------------------------- : 777
Ikl : TACGAAGGA-------------------------------------------------------------------------------- : 777
Ili : TACGAAGGA-------------------------------------------------------------------------------- : 777
Ipe : TACGAAGGA-------------------------------------------------------------------------------- : 777
Ise : TACGAAGGA-------------------------------------------------------------------------------- : 777
Ici : TACGAAGGA-------------------------------------------------------------------------------- : 777
Isc : TACGAAGGAGTGCGGTTCGTTCGAAAAATTCCTACCTCTCTATCTATCTCTGAGATGTTTGGATTTTTCAAAACTCCATGGACATGCAG : 890

900 * 920 * 940 * 960 * 98
Ath : ----------------------TCTCCCACTCCAGTCGTTGCTTTTCTTTCTGTTACTTCGAAAGTAGCTGCTTCAGCTTCAGCCACTC : 844
Ikl : ----------------------TCTCCCACTCCAGTCGTTGCTTTTCTTTCTGTTACTTCGAAAGTAGCTGCTTCAGCTTCAGCCACTC : 844
Ili : ----------------------TCTCCCACTCCAGTCGTTGCTTTTCTTTCTGTTACTTCGAAAGTAGCTGCTTCAGCTTCAGCCACTC : 844
Ipe : ----------------------TCTCCCACTCCAGTCGTTGCTTTTCTTTCTGTTACTTCGAAAGTAGCTGCTTCAGCTTCAGCCACTC : 844
Ise : ----------------------TCTCCCACTCCAGTCGTTGCTTTTCTTTCTGTTACTTCGAAAGTAGCTGCTTCAGCTTCAGCCACTC : 844
Ici : ----------------------TCTCCCACTCCAGTCGTTGCTTTTCTTTCTGTTACTTCGAAAGTAGCTGCTTCAGCTTCAGCCACTC : 844
Isc : AAGAGAAATGCTATCCCCACTCTCTCCCACTCCAGTCGTTGCTTTTCTTTCTGTTACTTCGAAAGTAGCTGCTTCAGCTTCAGCCACTC : 979

 0 * 1000 * 1020 * 1040 * 1060
Ath : GAATTTTCGATATTCCTTTTTATTTCTCATCAAATGAATGGCATCTTCTTCTGGAAATCCTAGCTATTCTTAGCATGATATTCGGGAAT : 933
Ikl : GAATTTTCGATATTCCTTTTTATTTCTCATCAAACGAATGGCATCTTCTTCTGGAAATCCTAGGTATTCTTAGCATGATATTGGGAAAT : 933
Ili : GAATTTTCGATATTCCTTTTTATTTCTCATCAAACGAATGGCATCTTCTTCTGGAAATCCTAGGTATTCTTAGCATGATATTGGGAAAT : 933
Ipe : GAATTTTCGATATTCCTTTTTATTTCTCATCAAACGAATGGCATCTTCTTCTGGAAATCCTAGGTATTCTTAGCATGATATTGGGAAAT : 933
Ise : GAATTTTCGATATTCCTTTTTATTTCTCATCAAACGAATGGCATCTTCTTCTGGAAATCCTAGGTATTCTTAGCATGATATTGGGAAAT : 933
Ici : GAATTTTCGATATTCCTTTTTATTTCTCATCAAACGAATGGCATCTTCTTCTGGAAATCCTAGGTATTCTTAGCATGATATTGGGAAAT : 933
Isc : GAATTTTCGATATTCCTTTTTATTTCTCATCAAACGAATGGCATCTTCTTCTGGAAATCCTAGGTATTCTTAGCATGATATTGGGAAAT : 1068

* 1080 * 1100 * 1120 * 1140 * 1160
Ath : CTCATTGCTATTACTCAAACAAGCATGAAACGTATGCTTGCATATTCGTCCATAGGTCAAATCGGATATGTAATTATTGGAATAATTGT : 1022
Ikl : CTCATTGCTATTACTCAAACAAGCATGAAACGTATGCTTGCATATTCGTCCATAGGTCAAATCGGATATGTAATTATTGGAATAATTGT : 1022
Ili : CTCATTGCTATTACTCAAACAAGCATGAAACGTATGCTTGCATATTCGTCCATAGGTCAAATCGGATATGTAATTATTGGAATAATTGT : 1022
Ipe : CTCATTGCTATTACTCAAACAAGCATGAAACGTATGCTTGCATATTCGTCCATAGGTCAAATCGGATATGTAATTATTGGAATAATTGT : 1022
Ise : CTCATTGCTATTACTCAAACAAGCATGAAACGTATGCTTGCATATTCGTCCATAGGTCAAATCGGATATGTAATTATTGGAATAATTGT : 1022
Ici : CTCATTGCTATTACTCAAACAAGCATGAAACGTATGCTTGCATATTCGTCCATAGGTCAAATCGGATATGTAATTATTGGAATAATTGT : 1022
Isc : CTCATTGCTATTACTCAAACAAGCATGAAACGTATGCTTGCATATTCGTCCATAGGTCAAATCGGATATGTAATTATTGGAATAATTGT : 1157

 1160 * 1180 * 1200 * 1220 * 1240
Ath : TGGAGACTCAAATGGTGGATATGCGAGCATGATAACTTATATGCTGTTCTATATCGCCATGAATCTAGGAACTTTTGCTTGCATTATAT : 1111
Ikl : TGGAGACTCAAATGATGGATATGCAAGCATGATAACTTATATGCTGTTCTATATCGCCATGAATCTAGGAACTTTTGCTTGCATTGTAT : 1111
Ili : TGGAGACTCAAATGATGGATATGCAAGCATGATAACTTATATGCTGTTCTATATCGCCATGAATCTAGGAACTTTTGCTTGCATTGTAT : 1111
Ipe : AGGAGACTCAAATGATGGATATGCAAGCATGATAACTTATATGCTGTTCTATATCGCCATGAATCTAGGAACTTTTGCTTGCATTGTAT : 1111
Ise : TGGAGACTCAAATGATGGATATGCAAGCATGATAACTTATATGCTGTTCTATATCGCCATGAATCTAGGAACTTTTGCTTGCATTGTAT : 1111
Ici : TGGAGACTCAAATGATGGATATGCAAGCATGATAACTTATATGCTGTTCTATATCGCCATGAATCTAGGAACTTTTGCTTGCATTGTAT : 1111
Isc : TGGAGACTCAAATGATGGATATGCAAGCATGATAACTTATATGCTGTTCTATATCGCCATGAATCTAGGAACTTTTGCTTGCATTGTAT : 1246

 * 1260 * 1280 * 1300 * 1320 *
Ath : TATTTGGTCTACGTACCGGAACTGATAACATTCGAGATTATGCAGGATTATACACAAAAGATCCTTTTTTGGCTCTCTCTTTAGCTCTA : 1200
Ikl : TATTTGGTCTACGTACCGGAACTGATAACATTCGAGATTATGCAGGATTATACACGAAAGATCCTTTTTTGGCTCTCTCTTTAACCCTA : 1200
Ili : TATTTGGTCTACGTACCGGAACTGATAACATTCGAGATTATGCAGGATTATACACGAAAGATCCTTTTTTGGCTCTCTCTTTAACCCTA : 1200
Ipe : TATTTGGTCTACGTACCGGAACTGATAACATTCGAGATTATGCAGGATTATACACGAAAGATCCTTTTTTGGCTCTCTCTTTAACCCTA : 1200
Ise : TATTTGGTCTACGTACCGGAACTGATAACATTCGAGATTATGCAGGATTATACACGAAAGATCCTTTTTTGGCTCTCTCTTTAACCCTA : 1200
Ici : TATTTGGTCTACGTACCGGAACTGATAACATTCGAGATTATGCAGGATTATACACGAAAGATCCTTTTTTGGCTCTCTCTTTAACCCTA : 1200
Isc : TATTTGGTCTACGTACCGGAACTGATAACATTCGAGATTATGCAGGATTATACACGAAAGATCCTTTTTTGGCTCTCTCTTTAACCCTA : 1335

1340 * 1360 * 1380 * 1400 * 1420
Ath : TGTCTCTTATCCCTAGGAGGTCTTCCTCCACTAGCAGGTTTTTTTGGAAAACTCCATTTATTCTGGTGTGGATGGCAGGCAGGCCTATA : 1289
Ikl : TGTCTCTTATCCCTAGGAGGTCTTCCTCCACTAGCAGGGTTTTTCGGAAAACTCTATTTATTCTGGTGTGGATGGCAGGCAGGCCTATA : 1289
Ili : TGTCTCTTATCCCTAGGAGGTCTTCCTCCACTAGCAGGGTTTTTCGGAAAACTCTATTTATTCTGGTGTGGATGGCAGGCAGGCCTATA : 1289
Ipe : TGTCTCTTATCCCTAGGAGGTCTTCCTCCACTAGCAGGTTTTTTCGGAAAACTCTATTTATTCTGGTGTGGATGGCAGGCAGGCCTATA : 1289
Ise : TGTCTCTTATCCCTAGGAGGTCTTCCTCCACTAGCAGGTTTTTTCGGAAAACTCTATTTATTCTGGTGTGGATGGCAGGCAGGCCTATA : 1289
Ici : TGTCTCTTATCCCTAGGAGGTCTTCCTCCACTAGCAGGTTTTTTCGGAAAACTCTATTTATTCTGGTGTGGATGGCAGGCAGGCCTATA : 1289
Isc : TGTCTCTTATCCCTAGGAGGTCTTCCTCCACTAGCAGGTTTTTTCGGAAAACTCTATTTATTCTGGTGTGGATGGCAGGCAGGCCTATA : 1424

 * 1440 * 1460 * 1480 * 1500 *
Ath : TTTCTTGGTTTCAATAGGACTCCTTACGAGCGT-TCTTTC-TATCTACTATTAT-CTAAAAATAATCAAGTTATTAATGACTGGACGAA : 1375
Ikl : TTTCTTGGTTTTAATAGGACTCCTTACAAGCGT-TGTTTC-TATCTACTATTAT-CTAAAAATAATCAAGTTATTAATGACTGGACGAA : 1375
Ili : TTTCTTGGTTTTAATAGGACTCCTTACAAGCGT-TGTTTC-TATCTACTATTAT-CTAAAAATAATCAAGTTATTAATGACTGGACGAA : 1375
Ipe : TTTCTTGGTTTTAATAGGACTCCTTACAAGCGT-TGTTTC-TATCTACTATTAT-CTAAAAATAATCAAGTTATTAATGACTGGACGAA : 1375
Ise : TTTCTTGGTTTTAATAGGACTCCTTACAAGCGT-TGTTTC-TATCTACTATTAT-CTAAAAATAATCAAGTTATTAATGACTGGACGAA : 1375
Ici : TTTCTTGGTTTTAATAGGACTCCTTACAAGCGT-TGTTTC-TATCTACTATTAT-CTAAAAATAATCAAGTTATTAATGACTGGACGAA : 1375
Isc : TTTCTTGGTTAATTTTGAATTGAGTATGATTGTATGTGTGATAGCATCTACTATACCAGGAATATCAATGAACCCGATTATTGCAATTG : 1513

 1520 * 1540 * 1560 * 1580 * 1600
Ath : ACCAAGAAATAACCCCTCACATGCGAAATTATAGAATATCCCCTTTAAGATCAAACAATTCCATCGAATTGAGTATGATTGTATGTGTG : 1464
Ikl : ACCAAGAAATAACCCCTCACGTGCGAAATTATAGAAGATCTCCTTTAAGATCAAACAATTCCATCGAATTGAGTATGATTGTATGTGTG : 1464
Ili : ACCAAGAAATAACCCCTCACGTGCGAAATTATAGAAGATCTCCTTTAAGATCAAACAATTCCATCGAATTGAGTATGATTGTATGTGTG : 1464
Ipe : ACCAAGAAATAACCCCTCACGTGCGAAATTATAGAAGATCTCCTTTAAGATCAAACAATTCCATCGAATTGAGTATGATTGTATGTGTG : 1464
Ise : ACCAAGAAATAACCCCTCACGTGCGAAATTATAGAAGATCTCCTTTAAGATCAAACAATTCCATCGAATTGAGTATGATTGTATGTGTG : 1464
Ici : ACCAAGAAATAACCCCTCACGTGCGAAATTATAGAAGATCTCCTTTAAGATCAAACAATTCCATCGAATTGAGTATGATTGTATGTGTG : 1464
Isc : CTCAGGATACCCTTTTT------------------------------------------------------------------------ : 1530

* 1620 * 1640 * 1660 * 1680 *
Ikl : ATAGCATCTACTATACCAGGAATATCAATGAACCCGATTATTGCAATTGCTCAGGATACCCTTTTT----------------------- : 1530
Ili : ATAGCATCTACTATACCAGGAATATCAATGAACCCGATTATTGCAATTGCTCAGGATACCCTTTTT----------------------- : 1530
Ipe : ATAGCATCTACTATACCAGGAATATCAATGAACCCGATTATTGCAATTGCTCAGGATACCCTTTTT----------------------- : 1530
Ise : ATAGCATCTACTATACCAGGAATATCAATGAACCCGATTATTGCAATTGCTCAGGATACCCTTTTTGTATTATTTGATCTGTTCTATAT : 1553
Ici : ATAGCATCTACTATACCAGGAATATCAATGAACCCGATTATTGCAATTGCTCAGGATACCCTTTTTTAG-------------------- : 1533
Isc : ----------------------------------------------------------------------------------------- : -

 1700 * 1720 *
Ath : ------------------------------------------- : -
Ikl : ------------------------------------------- : -
Ili : ------------------------------------------- : -
Ipe : ------------------------------------------- : -
Ise : CGCCATGAATCTAGGAACTTTTGCTTGCATTGTATTATTTGGT : 1596
Ici : ------------------------------------------- : -
Isc : ------------------------------------------- : -
**Figure S94 Multiple sequence alignment of the deduced nucleotide sequences of *ndh*B gene.** The origins of the nucleotide sequences used in this alignment are from: *Arabidopsis thaliana* (Ath), *Iodes klaineana* (Ikl), *Iodes cirrhosa* (Ici), *Iodes seretii* (Ise), *Iodes scandens* (Isc), *Iodes perrieri* (Ipe) and *Iodes liberica* (Ili). The nucleotide numbers for each sequence are indicated on the right. In the sequence alignment, identical nucleotides are shown with a black background, and similar residues are shown with a gray background.

* 20 * 40 * 60 * 80 *
Ikl : ATGTTTCTGCTTTACGAATATGATATTTTTTGGGTATTTCTGATTATATCAAGTCTTATTCCTTTTTTGGCACTTTTCATTTCTAGAGTT : 90
Ipe : ATGTTTCTGCTTTACGAATATGATATTTTTTGGGTATTTCTGATTATATCAAGTCTTATTCCTTTTTTGGCACTTTTCATTTCGAGAGTT : 90
Ici : ATGTTTCTGCTTTACGAATATGATATTTTTTGGGTATTTCTAATTATATCAAGTCTTATTCCTGTTTTGGCACTTTTCATTTCTAGAGTT : 90
Ise : ATGTTTCTGCTTTACGAATATGATATTTTTTGGGTATTTCTAATTATATCAAGTCTTATTCCTTTTTTGGCACTTTTCATTTCTAGAGTT : 90
Ili : ------------------------------------------------------------------------------------------ : -
Ath : ATGTTTCTGCTTTACGAATATGATATTTTCTGGGCATTTTTACTAATATCAAGTGCTATTCCTGTTTTGGCATTTCTAATTTCCGGAGTT : 90
Isc : CTCTATGTTGTTTACGAA-ATCTGGTTCTTTTGGCTTTATTCGCGATATT---TCTATCTATTATTTTGGAGATTTATAATTCTTATATT : 86

 100 * 120 * 140 * 160 * 180
Ikl : TTAGCCCCAATTAACAAAAGCGCAGAGAAGCTTTCTAGT-TATGAATCGGGTATAGAACCGATGGGCG---ACGCTTGGTTACAATTTCG : 176
Ipe : TTAGCCCCAATTAACAAAGGCCCAGAGAAGCTTTCGAGT-TATGAATCGGGTATAGAACCGATGGGCG---ACGCTTGGTTACAATTTCG : 176
Ici : TTAGCCCCAATTAACAAAGGCCCAGAGAAGCTTTCTAGT-TATGAATCGGGTATAGAACCGATGGGCG---ACGCTTGGTTACAACTTCG : 176
Ise : TTAGCCCCAATTAACAAAGGCCCAGAGAAGTTTTCTAGT-TATGAATCGGGTATAGAACCGATGGGCG---ACGCTTGGTTACAATTTCG : 176
Ili : ------------------------------------------------------------------------------------------ : -
Ath : TTATCTCCAATTAGGAAGGGGCCGGAGAAACTTTCTAGT-TATGAATCAGGTATAGAACCGATCGGGG---ATGCTTGGTTACAATTTAG : 176
Isc : TCTTATGTACCCATAGGTGCTATATTGGATTTATATAGTATATGGATACAGAATGTATTGGGTTTACGTATATCCTTATTCTATATTTTA : 176

 * 200 * 220 * 240 * 260 *
Ikl : AAT---CCGTTATTATATGTTTGCT--CTAGTTTTTGTTGTTTTTGATGTT--GAAACAGTTTTTCTTTATCCATGGGCAATGAGTTT-- : 257
Ipe : AAT---CCGTTATTATATGTTTGCT--CTAGTTTTTGTTGTTTTTGATGTT--GAAACAGTTTTTCTTTATCCATGGGCAATGAGTTT-- : 257
Ici : AAT---TCGTTATTATATGTTTGCT--CTAGTTTTTGTTGTTTTTGATGTT--GAAACAGTTTTTCTTTATCCAGGGGCAATGAGTTT-- : 257
Ise : AAT---CCGTTATTATATGTTTGCT--CTAGTTTTTGTTGTTTTTGATGTT--GAAACCGTTTTTCTTTATCCATGGGCAATGAGTTT-- : 257
Ili : ----------------CTGTTTGCT--CTAGTTTTTGTTGTTTTTGATGTT--GAAACAGTTTTTCTTTATCCATGGGCAATGAGTTT-- : 68
Ath : AAT---CCGTTATTATATGTTTGCT--CTAGTTTTTGTTGTTTTTGATGTT--GAAACTGTTTTTCTGTATCCGTGGGCAATGAGTTT-- : 257
Isc : TGTATATCCTTATTCTATATTTTATTACTACTATTTTTGGTTTTGGATCTTCCAAATCATTTTCTGGATATTCCTCAATGTCCCGTCTAT : 266

280 * 300 * 320 * 340 * 360
Ikl : CGATGTATTAGGTGTATCTGTATTTATAGAAGCTTTGATTTTCGTAC--TTAT----------CTTAATTGT----TGGTTTAGTTTATG : 331
Ipe : CGATGTATTAGGTGTATCTGTATTTATAGAAGCTTTGATTTTCGTAC--TTAT----------CTTAATTGT----TGGTTTAGTTTATG : 331
Ici : CGATGTATTAGGTGTATCTGTATTTATAGAAGCTTTGATTTTCGTAC--TTAT----------CTTAATTGT----TGGTTTAGTTTATG : 331
Ise : CGATGTATTAGGTGTATCTGTATTTATAGAAGCTTTGATTTTCGTAC--TTAT----------CTTAATTGT----TGGTTTAGTTTATG : 331
Ili : CGATGTATTAGGTGTATCCGTATTTATAGAAGCTTTGATTTTCGTAC--TTAT----------CTTAATTGT----TGGTTTAGTTTATG : 142
Ath : CGATGTACTGGGGGTATCTGCTTTTATAGAAGCTTTCATTTTCGTGC--TTAT----------CCTAATTCT----TGGTTTAGTTTATG : 331
Isc : TAGTATCTTGCCAGTATCGGTAT--AAATGATCTTTCCCTCGCGTTCGGCTATAGCGAGAGCCCCTGAATCTAGAGCGGCTTGGCGT-TC : 353

 * 380 * 400 * 420 * 440 *
Ikl : CATGGCGAAAAGGGGCATTGGAATGGTCT------------------------------------------------------------- : 360
Ipe : CATGGCGAAAAGGGGCATTGGAATGGTCT------------------------------------------------------------- : 360
Ici : CATGGCGAAAAGGGGCATTGGAATGGTCTTAG---------------------------------------------------------- : 363
Ise : CATGGCGAAAAGGGGCATTGGAATGGTCT------------------------------------------------------------- : 360
Ili : CATGGCGAAAAGGGGCATTGGAATGGTCT------------------------------------------------------------- : 171
Ath : CATGGCGAAAAGGAGCATTGGAATGGTCTTAG---------------------------------------------------------- : 363
Isc : CCTGCTAAAAAGGATGGTGATCCTAATACCAAGGGTACTACTACTGATCAACCGGCTGAACAAAGTTCTAGCGCCATGCCAAATGTGTCC : 443
 460 * 480 * 500 * 520 * 540
Ikl : ------------------------------------------------------------------------------------------ : -
Ipe : ------------------------------------------------------------------------------------------ : -
Ici : ------------------------------------------------------------------------------------------ : -
Ise : ------------------------------------------------------------------------------------------ : -
Ili : ------------------------------------------------------------------------------------------ : -
Ath : ------------------------------------------------------------------------------------------ : -
Isc : GAAGAAGAAAAGCCCCAGAGGGAAAGGTCCTTCCCGGCCGGTTGTGGGCGAGGAGGGATTCGAACCCTATATGTTTATATATATGTATTT : 533

* 560 * 580 * 600 * 620 *
Ikl : ------------------------------------------------------------------------------------------ : -
Ipe : ------------------------------------------------------------------------------------------ : -
Ici : ------------------------------------------------------------------------------------------ : -
Ise : ------------------------------------------------------------------------------------------ : -
Ili : ------------------------------------------------------------------------------------------ : -
Ath : ------------------------------------------------------------------------------------------ : -
Isc : TGCTTAACCTGGTTCAGTAATATTTCTGACGCTTTATTTCTATCCTAGTTGATCCTGATTCGACATTAGAAGTATATTGATTTTTGTATT : 623

 640 * 660 * 680 * 700 * 720
Ikl : ------------------------------------------------------------------------------------------ : -
Ipe : ------------------------------------------------------------------------------------------ : -
Ici : ------------------------------------------------------------------------------------------ : -
Ise : ------------------------------------------------------------------------------------------ : -
Ili : ------------------------------------------------------------------------------------------ : -
Ath : ------------------------------------------------------------------------------------------ : -
Isc : CATCTATCCCGGGGATATAATCGGAGGACATGTATGGTTAGGTTCCATTTGTATACTTGGTGGAATCTGGCATATCTTAACCAAACCATT : 713

 * 740 * 760 * 780 * 800 *
Ikl : ------------------------------------------------------------------------------------------ : -
Ipe : ------------------------------------------------------------------------------------------ : -
Ici : ------------------------------------------------------------------------------------------ : -
Ise : ------------------------------------------------------------------------------------------ : -
Ili : ------------------------------------------------------------------------------------------ : -
Ath : ------------------------------------------------------------------------------------------ : -
Isc : TGCCTGGGCTCGACGGGCACTTGTATGGTCTAACTTACTTATACCAGATCTTTATTTTGTTTTGCCCCTTATCGTAGTATTCATATTACA : 803

820 * 840 *
Ikl : ------------------------------------------- : -
Ipe : ------------------------------------------- : -
Ici : ------------------------------------------- : -
Ise : ------------------------------------------- : -
Ili : ------------------------------------------- : -
Ath : ------------------------------------------- : -
Isc : AATCTTACTAGAACTGCTCGTTTTCTATCGGAAGGCTGTGATT : 846

**Figure S95 Multiple sequence alignment of the deduced nucleotide sequences of *ndh*C gene.** The origins of the nucleotide sequences used in this alignment are from: *Arabidopsis thaliana* (Ath), *Iodes klaineana* (Ikl), *Iodes cirrhosa* (Ici), *Iodes seretii* (Ise), *Iodes scandens* (Isc), *Iodes perrieri* (Ipe) and *Iodes liberica* (Ili). The nucleotide numbers for each sequence are indicated on the right. In the sequence alignment, identical nucleotides are shown with a black background, and similar residues are shown with a gray background.

* 20 * 40 * 60 * 80
Ikl : ACGAATTATTTTCCTTGGTTAACAATAATTGTCGTTTTGCCAATATCTGCAGGTTCCTTAATTTTCTTTCTTCCCCATAGAGGAAATAG : 89
Ili : ----------------------------------------------------------------------------------------- : -
Ise : ---------------------------------------CCAATATCTGCAGGTTCCTTAATTTTCTTTCTTCCCCATAGGGGAAATAG : 50
Ipe : ACGAATTATTTTCCTTGGTTAACAATAATTGTCGTTTTGCCAATATCTGCAGGTTCCTTAATTTTCTTTCTTCCCCATAAGGGAAATAG : 89
Ici : ACGAATTATTTTCCTTGGTTAACAATAATTGTCGTTTTGCCAATATCTGCAGGTTCCTTAATTTTCTTTCTTCCCCATAGAGGAAATAG : 89
Isc : ACGAATTATTTCCCTTGGTTAACAATAATTGTCGTTTTGCCAATATCTGCAGGTTCCTTAATTTTCTTTCTTCCCCATAGAGGAAATAG : 89
Ath : ACGAATGATTTTCCTTGGTTAACAATAATTGTTGTTTTTCCAATATCTGCTGGTTCATTAATGTTATTTCTCCCGCATAGGGGAAATAA : 89

 * 100 * 120 * 140 * 160 * 180
Ikl : GACAATTCAATGGTATACTATATGCATATGTATTTTAGAACTCCTTTTAACGACCTATACATTCTGTTATCATTTTCAACCAGATGATC : 178
Ili : ----------------------------------------------------------------------------------------- : -
Ise : GACAATTAAGTGGTATACTATATGCATATGTATTTTAGAACTCCTTTTAACGACCTATACATTCTGTTATCATTTTCAACCAGATGATC : 139
Ipe : GACAATCAGGTGGTATACTATATGTATATGTATTTTAGAACTCCTTTTAACGACCTATACGTTCTGTTATCATTTTCAACCAGATGATC : 178
Ici : GACAATTCAGTGGTATACTATATGCATATGTATTTTGGAACTCCTTTTAACGACCTATACATTCTGTTATCATTTTCAACCGGGTGATC : 178
Isc : GACAATTCTGTGGTATACTATATGCATATGTATTTTAGAACTCCTTTTAACGACCTATACATTCTGTTATCATTTTCAACCAGGTGATC : 178
Ath : AGTCAATAAATGGTATACTATATGCATTTGCATCTTAGAACTTCTTCTAACGACCTACGCTTTTTGTTATAATTTTAAAATGGACGATC : 178

 80 * 200 * 220 * 240 * 260
Ikl : CATTAATACAACTAGTAGAGGATTATAAATGGATCAATTTTTTTGATTTCCGTTGGAGATTAGGAATAGACGGACTTTCTATAGGACCC : 267
Ili : ----------------------------------------------------------------------------------------- : -
Ise : CATTAATACAACTAGTAGAGGATTATAAATGGATCAATTTTTTTGATTTCCATTGGAGATTAGGAATAGACGGACTTTCTATAGGACCC : 228
Ipe : CATTAATACAACTAGTAGAGGATTATAAATGGATCAATTTTTTTGATTTCCATTGGAGATTAGGAATAGACGGACTTTCTATAGGACCC : 267
Ici : CATTAATACAACTAGTAGAGGATTATAAATGGATCAATTTTTTTGATTTCCATTGGAGATTAGGAATAGACGGACTTTCTATAGGACCC : 267
Isc : CATTAATACAACTAGTAGAGGATTATAAATGGATCAATTTTTTTTATTTCCATTGGAGATTAGGAATAGACGGACTTTCTATAGGACCC : 267
Ath : CATTAATTCAACTGTCCGAAGATTATAAATGGATCGATTTTTTTGATTTTTACTGGAGAATGGGAATAGATGGACTTTCTATAGGAACT : 267

* 280 * 300 * 320 * 340 *
Ikl : ATTCTACTGACGGGATTCATTACCACTCTAGCCACTTTAGTGGCTCCGCCCGTTACTCGAGATTCGCGATTATTCCATTTCCTGATGTT : 356
Ili : ----------------------------------------------------------------------------------------- : -
Ise : ATTCTACTGACGGGATTCATTACCACTCTAGCCACTTTAGTGGCTCGGCCAGTTACTCGAGATTCGCGATTATTCCATTTCCTGATGTT : 317
Ipe : ATTCTACTGACGGGGTTCATTACCACTCTAGCCACTTTAGTGGCTCGGCCAGTTACTCGAGATTCGCGATTATTCCATTTCCTGATGTT : 356
Ici : ATTCTACTGACGGGATTCATTACCACTCTAGCCACTTTAGTGGCTCGGCCAGTTACTCGAGATTCACGATTATTCCATTTCCTGATGTT : 356
Isc : ATTCTACTGACGGGATTCATTACCACTCTAGCCACTTTAGTGGCTCGGCCAGTTACTCGAGATTCACGATTATTCCATTTTCTGATGTT : 356
Ath : ATTTTACTGACGGGATTTATTACTACTTTAGCCACTTTAGCGGCTTTTCCAGTTACTCGGGATTCCCGATTTTTTCATTTCCTGATGTT : 356

 360 * 380 * 400 * 420 * 440
Ikl : AGCAATGTACAGCGGTCAAATAGGATCATTTTCTTCTCAGAACCTTTTACTTTTTTTCATCATGTGGGAATTAGAATTAATTCCTGTTT : 445
Ili : ----------------------------------------------------------------------------------------- : -
Ise : AGCAATGTACAGCGGTCAAATAGGATCATTTTCTTCTCAGAACCTTTTACTTTTTTTCATCATGTGGGAATTAGAATTAATTCCTGTTT : 406
Ipe : AGCAATGTACAGCGGTCAAATAGGATCATTTTCTTCTCAGAACCTTTTATTTTTTTTCATCATGTGGGAATTAGAATTAATTCCTGTTT : 445
Ici : AGCAATGTACAGCGGTCAAATAGGATCATTTTCTTCTCAGAACCTTTTACTTTTTTTTATCATGTGGGAATTAGAATTAATTCCTGTTT : 445
Isc : AGCAATGTACAGCGGTCAAATAGGATCATTTTCTTCTCAGAACCTTTTACTTTTTTTTATCATGTGGGAATTAGAATTAATTCCTGTTT : 445
Ath : AGCAATGTACAGCGGTCAAATAGGATCATTTTCTTCTCGGGATCTTCTACTTTTTTTCATCATGTGGGAATTAGAATTAATTCCCGTTT : 445

 * 460 * 480 * 500 * 520 *
Ikl : ATATCCTTCTATCCATGTGGGGAGGAAAGAAACGTCTTTACTCAGCTACAAAATTTATTTTGTACACGGCGGGGGGTTCCGTTTTTCTC : 534
Ili : ----------------------------------------------------------------------------------------- : -
Ise : ATATCCTTCTATCCATGTGGGGAGGAAAGAAGCGTCTTTACTCAGCTACAAAATTTATTTTGTACACGGCGGGGGGTTCCGTTTTTCTC : 495
Ipe : ATATCCTTCTATCCATGTGGGGAGGAAAGAAACGTCTTTACTCAGCTACAAAATTTATTTTGTACACGGCGGGGGGTTCCATTTTTCTC : 534
Ici : ATATCCTTCTATCCACGTGGGGAGGAAAGAAACGTCTTTACTCAGCTACAAAATTTATTTTGTACACGGCGGGGGGTTCCGTTTTTCTC : 534
Isc : ATATCCTTCTATCCATGTGGGGAGGAAAGAAACGTCTTTACTCAGCTACAAAATTTATTTTGTACACGGCGGGGGGTTCCGTTTTTCTC : 534
Ath : ATCTACTTTTATCCATGTGGGGTGGAAAGAAACGTCTGTATTCGGCTACAAAATTTATTTTGTACACGGCAGGAAGTTCTATTTTTTTA : 534

540 * 560 * 580 * 600 * 620
Ikl : TTAATGTCAGTTTTGGGTATCCATTTATATGGTTCTAATGAACAACTAACATTTCATTTTGAAACATTAGCCACCCGGTCGTATCCTGT : 623
Ili : ------------------------------GGTTCTAATGAACAACTAACATTTCATTTTGAAACATTAGCCACCCGGTCGTATCCTGT : 59
Ise : TTAATGTCAGTTTTGGGTATCCATTTATATGGTTCTAATGAAC---CAACATTTAATTTTGAAACATTAGCCACCCGGTCGTATCCTGT : 581
Ipe : TTAATGTCAGTTTTGGGTATCCATTTATATGGTTCTAATGAAC---CAACATTTCATTTTGAAACATTAGCCACCCGGTCGTATCCTGT : 620
Ici : TTAATGTCAGTTTTGGGTATCCATTTATATGGTTCTAATGAAC---CAACATTTCATTTTGAAATATTAGCCACCCGGTCGTATCCTGT : 620
Isc : TTAATGTCAGTTTTGGGTATACATTTATATGGTTCTAATGAAC---CAACATTTCATTTTGAAACATTAACCACCCGGTCGTATCCTGT : 620
Ath : TTAATAGGAGTTTTAGGTATAAGTTTATATGGTTCGAACGAAC---CAACATTAAATTTAGAACTCTTAGCTAATAAATCCTATCCTGT : 620

 * 640 * 660 * 680 * 700 *
Ikl : GGCCTTGGAAATACTATTCTATATTGGATTTTTTATTGCTTTTGCTGTCAAATTGCCAATTATACCCCTACATACATGGTTACCAGATA : 712
Ili : GGCCTTGGAAATACTATTCTATATTGGATTTTTTATTGCTTTTGCTGTCAAATTGCCAATTATACCCCTACATACATGGTTACCAGATA : 148
Ise : AGCCTTGGAAATACTATTCTATATTGGATTTTTTATTGCTTTTGCTGTCAAATTGCCAATTATACCCCTACATACATGGTTACCAGATA : 670
Ipe : GGCCTTGGAAATACTATTCTATATTGGATTTTTTATTGCTTTTGCTGTCAAATTGCCAATTATACCCCTACATACATGGTTACCAGATA : 709
Ici : GGCCTTGGAAATACTATTCTATATTGGATTTTTTATTGCTTTTGCTGTCAAATTGCCGATTATACCCCTACATACATGGTTACCAGATA : 709
Isc : GGCCCTGGAAATACTATTCTATATTGGATTTTTTATTGCTTTTGCTGTCAAATTGCCGATTATACCCCTACATACATGGTTACCAGATA : 709
Ath : TACACTTGAAATACTATTTTATATTGGATTTCTTATTGCTTTTGCCGTCAAATCACCGATTATACCTTTACATACTTGGTTACCTGACA : 709

 720 * 740 * 760 * 780 * 800
Ikl : CCCACGGAGAAGCCCATTACAGTACTTGTATGCTTCTAGCCGGAATC------TTATTAAAAATGGGAGCATATGGATTGATTCGAATC : 795
Ili : CCCACGGAGAAGCCCATTACAGTACTTGTATGCTTCTAGCCGGAATC------TTATTAAAAATGGGAGCATATGGATTGATTCGAATC : 231
Ise : CCCACGGAGAAGCCCATTACAGTACTTGTATGCTTCTAGCCGGAATC------TTATTAAAAATGGGAGCATATGGATTGATTCGAATC : 753
Ipe : CCCACGGAGAAGCCCATTACAGTACTTGTATGCTTCTAGCCGGAATC------TTATTAAAAATGGGAGCATATGGATTGATTCGAATC : 792
Ici : CCCATGGAGAAGCCCATTACAGTACTTGTATGCTTCTAGCCGGAATC------TTATTAAAAATGGGAGCATATGGATTGATTCGAATC : 792
Isc : CCCATGGAGAAGCCCATTACAGTACTTGTATGCTTCTAGCCGGAATCGGAATCTTATTAAAAATGGGAGCATATGGATTGATTCGAATC : 798
Ath : CCCACGGTGAGGCACATTACAGTACCTGTATGCTTCTCGCTGGAATC------TTATTAAAAATGGGAGCCTATGGGTTGGTTCGAATC : 792

* 820 * 840 * 860 * 880 *
Ikl : AATATGGAATTATTACCGCATGCTCATTCCATATTTTCACCTTGGTTGATGACAGTAGGCGCAATACAAATAATCTATGCAGCTTCAAC : 884
Ili : AATATGGAATTATTACCGCATGCTCATTCCATATTTTCGCCTTGGTTGATGACAGTAGGCGCAATACAAATAATCTATGCAGCTTCAAC : 320
Ise : AATATGGAATTATTACCGCATGCTCATTCCATATTTTCGCCTTGGTTGATGACAGTAGGCGCAATACAAATAATCTATGCAGCTTCAAC : 842
Ipe : AATATGGAATTATTACCGCATGCTCATTCTATATTTTCGCCTTGGTTGATGACAGTAGGTGCAATACAAATAATCTATGCAGCTTCAAC : 881
Ici : AATATGGAATTATTACCGCACGCTCATTCCATATTTTCGCCTTGGTTGATGACAGTAGGCGCAATACAAATAATCTATGCAGCTTCAAC : 881
Isc : AATATGGGGTTGATGA----------------ACTTTAACGAAAGATCATGA-----GGCGCAATACAAATAATCTATGCAGCTTCAAC : 866
Ath : AATATGGAATTATTACCCCACGCTCATTCTATGTTTTCTCCTTGGTTGTTGGTAGTCGGTACAATCCAAATAATTTATGCAGCTTCAAC : 881

900 * 920 * 940 * 960 * 98
Ikl : ATCTCCTGGACAGCGGAATTTTAAAAAAAGGCTAGCCTATTCCTCTGTATCTCATATGGGTTTCATAATTATAGGAATTGGTTCTATAA : 973
Ili : ATCTCCTGGACAGCGGAATTTAAAAAAAAGGCTAGCCTATTCCTCTGTATCTCATATGGGTTTCATAATTATAGGAATTGGTTCTATAA : 409
Ise : ATCTCCTGGACAGCGGAATTTAAAAAAAAGGCTAGCCTATTCCTCTGTATCTCATATGGGTTTTATAATTATAGGAATTGGTTCTATAA : 931
Ipe : ATCTCCTGGACAGCGGAATTTAAAAAAAAGGCTAGCCTATTCCTCTGTATCTCATATGGGTTTCATAATTATAGGAATTGGTTCTATAA : 970
Ici : ATCTCCTGGACAGCGGAATTTTAAAAAAAGACTAGCCTATTCCTCTGTATCTCATATGGGTTTTATAATTATAGGAATTGGTTCGATAA : 970
Isc : ATCTCCTGGACAGCGGAATTTAAAAAAAAGACTAGCCTATTCCTCTGTATCTCATATGGGTTTTATAATTATAGGAATTGCTTCTATAA : 955
Ath : ATCTCCCGGTCAACGTAATTTAAAAAAGAGAATAGCCTATTCTTCTGTATCTCATATGGGTTTTATAATTATAGGTATTAGTTCTATAA : 970

0 * 1000 * 1020 * 1040 * 1060
Ikl : CCGATATGGGGCTCAACGGGGCCCTTTTACAAATAATCTCTCATGGATTTATTGGTGCTGCACTTTTTTTCTTGGCGGGAACGAGTTAT : 1062
Ili : CCGATATGGGGCTCAACGGGGCCCTTTTACAAATAATCTCTCATGGATTTATTGGTGCTGCACTTTTTTTCTTGGCGGGAACGAGTTAT : 498
Ise : CCGATATGGGGCTCAACGGGGCCCTTTTACAAATAATCTCTCATGGATTTATTGGTGCTGCACTTTTTTTCTTGGCGGGAACGAGTTAT : 1020
Ipe : CCGATATGGGGCTCAACGGAGCCCTTTTACAAATAATCTCTCATGGATTTATTGGTGCTGCACTTTTTTTCTTGGCGGGAACGAGTTAT : 1059
Ici : CCGATATGGGACTCAACGGGGCCCTTTTACAAATAATCTCTCATGGATTTATTGGTGCTGCACTTTTTTTCTTGGCGGGAACGAGTTAT : 1059
Isc : CCGATATGGGGCTCAACGGGGCCCTTTTACAAATAATCTCTCATGGATTTATTGGTGCTGCACTCTTTTTCTTGGCGGGAACGAATTAT : 1044
Ath : CGGATCCTGGACTTAATGGAGCTATTTTACAAATAATTTCTCATGGATTTATTGGCGCTGCACTTTTTTTCTTGGCAGGAACGAGTTAT : 1059

* 1080 * 1100 * 1120 * 1140 * 1160
Ikl : GATAGAATACGTCTTGTTTATCTTGATGAAATGGGTGGAATAGGTATTCCAATGCCAAAAATATTCACAATGTTTAGTAGCTTTTCAAT : 1151
Ili : GATAGAATACGTCTTGTTTATCTTGATGAAATGGGTGGAATAGGTATTCCAATGCCAAAAATATTCACAATGTTTAGTAGCTTTTCAAT : 587
Ise : GATAGAATACGTCTTGTTTATCTTGATGAAATGGGTGGAATAGGTATTCCAATGCCAAAAATATTCACAATGTTTAGTAGCTTTGCAAT : 1109
Ipe : GATAGAATACGTCTTGTTTATCTTGACGAAATGGGTGGAATAGGTATTCCAATGCCAAAAATATTCACAATGTTTAGTAGCTTTTCAAT : 1148
Ici : GATAGAATACGTCTTGTTTATCTTGATGAAATGGGTGGAATAGGTATTCCAATGCCAAAAATATTCACAATGTTTAGTAGCTTTTCAAT : 1148
Isc : GATAGAATACGTCTTGTTTATCTTGATGAAATGGGTGGAATAGGT-------------------------------------------- : 1089
Ath : GATAGAATTAGGCTTGTTTATCTTGATGAAATGGGTGGAATGGCTATCTCCATTCCAAAAATATTTACAATGTTCACTATTTTATCGAT : 1148

 1160 * 1180 * 1200 * 1220 * 1240
Ikl : GGCTTCCCTTGCATTACCAGGTATGAGTGGGTTTGTTGCAGAATTGATAGTCTTTTTTGGAATAATTACCAGTCAAAAATGTCTTTTAA : 1240
Ili : GGCTTCCCTTGCATTACCAGGTATGAGTGGTTTTGTTGCAGAATTGATAGTCTTTTTTGGAATAATTACCAGTCAAAAATGTCTTTTAA : 676
Ise : GGCTTCCCTTGCATTACCAGGTATGAGTGGTTTTGTTGCAGAATTGATAGTCTTTTTTGGAATAATTACCTGTCAAAAATGTCTTTTAA : 1198
Ipe : GGCTTCCCTTGCATTACCAGGTATGAGTGGTTTTGTTGCAGAATTGATAGTCTTTTTTGGAATAATTACCAGTCAAAAATGTCTTTTAA : 1237
Ici : GGCTTCCCTTGCATTACCAGGTATGAGTGGTTTTGTTGCAGAATTGATAGTCTTTTTTGGAATAATTACCAGTCAAAAATGTCTTTTAA : 1237
Isc : ----------------------------------------------------------------------------------------- : -
Ath : GGCTTCCCTTGCATTGCCGGGCATGAGTGGTTTTATTGCAGAATTCATTGTTTTTTTTGGAATAATTACCAGCCAAAAATATTTCTTAA : 1237

* 1260 * 1280 * 1300 * 1320 *
Ikl : TGCCAAAAATACTAATTACTTTAGTAATGGCAATTGGAATGATATTAACTCCTATTTATTCATTATCTATGTTACGACAAATATTCTAT : 1329
Ili : TGCCAAAAATACTAATTACTTTAGTAATGGCAATTGGAATGATATTAACTCCTATTTATTCATTATCTATGTTACGACAAATATTCTAT : 765
Ise : TGCCAAAAATAATAATTACTTTAGTAATGGCAATTGGAATGATATTAACTCCTATTTATTCATTATCTATGTTACGACAAATATTCTAT : 1287
Ipe : TGCCAAAAATACTAATTACTTTAGTAATGGCAATTGGAATGATATTAACTCCTATTTATTCATTATCTATGTTACGACAAATATTCTAT : 1326
Ici : TGACAAAAATACTAATTACTTTAGTAATGGCAATTGGAATGATATTAACTCCTATTTATTCATTATCTATGTTACGACAAATATTCTAT : 1326
Isc : ----------------------------------------------------------------------------------------- : -
Ath : TTTCCAAAATTTTCATTATTTTTGTAATGGCAATTGGAATGATATTAACTCCTATATATTTATTATCTATGTTACGCCAAATGTTCTAT : 1326

1340 * 1360 * 1380 * 1400 * 1420
Ikl : GGATACAAACTATTTAATACCCCAAACTCTTATTTTTTTGATTCTGGACCACGAGAGCTATTTGTTTCGATCTCTATATTTTTACCTGT : 1418
Ili : GGATACAAACTATTTAATACCCCAAACTCTTATTTTTTTGATTCTGGACCACGAGAGCTATTTGTTTCGATCTCTATATTTTTACCTGT : 854
Ise : GGATACAAACTATTTAATACTCCAAACTCTTATTTTTTTGATTCTGGACCACGAGAGCTATTTGTTTCGATCTCTATATTTTTACCTGT : 1376
Ipe : GGATACAAACTATTTAATACCCCAAACTCTTATTTTTTTGATTCTGGACCACGAGAGCTATTTGTTTCGATCTCTATATTTTTACCTGT : 1415
Ici : GGATACAAACTATTTAATACCCCAAACTCTTATTTTTTTGATTCTGGACCACGAGAGCTATTTGTTTCGATCTCTATATTTTTACCTGT : 1415
Isc : ----------------------------------------------------------------------------------------- : -
Ath : GGATACAAGTTAATTAATATCAAAAACTTTTCTTTTTTTGATTCTGGACCCCGAGAGTTATTTCTTTCAATCTCTATTCTTTTACCCAT : 1415

* 1440 * 1460 * 1480 * 1500 *
Ikl : AATAGGTATTGGTATATACCCCGATTTCGTTCTTTCACTATCAGTTGACAAGGTCGAAGTTATTCTATCCAATTATTTTTATAGA---- : 1503
Ili : AATAGGTATTGGTATATACCCCGATTTCGTTCTTTCACTATCAGTTGACAAGGTCGAAGTTATTCTATCCAATTATTTTTATAGA---- : 939
Ise : AATAGGTATTGGTATATACCCCGATTTCGTTCTTTCACTATCAGTTGACAAGGTCGAAGTTATTCTATCCAATTAT------------- : 1452
Ipe : AATAGGTATTGGTATATACCCTGATTTCGTTCTTTCACTATCAGTTGACAAGGTCGAAGTTATTCTATCCAATTTTTTTTATAGAACTA : 1504
Ici : AATAGGTATTGGTATATACCCCGATTTCGTTCTTTCACTATCAGTTGACAAGGTCGAACTTATTCTATCCAATTATTTTTATAGATAG- : 1503
Isc : ----------------------------------------------------------------------------------------- : -
Ath : AATTGGTATTGGGATTTATCCTGATTTTGTGCTCTCATTAGCAAGTGACAAGGTCGAATCCATTTTATCTAATTATTTTTATGGATAG- : 1503

 1520 * 1540 * 1560 * 1580 * 1600
Ikl : ----------------------------------------------------------------------------------------- : -
Ili : ----------------------------------------------------------------------------------------- : -
Ise : ----------------------------------------------------------------------------------------- : -
Ipe : GTTCTAATTTGATACAAGTTTATATTTTTTGGGAATTGGTTGGAATGTGTTCCTATCTATTAATAGGGTTTTGGTTCACACGACCTGCT : 1593
Ici : ----------------------------------------------------------------------------------------- : -
Isc : ----------------------------------------------------------------------------------------- : -
Ath : ----------------------------------------------------------------------------------------- : -

* 1620 * 1640 * 1660 * 1680 *
Ikl : ----------------------------------------------------------------------------------------- : -
Ili : ----------------------------------------------------------------------------------------- : -
Ise : ----------------------------------------------------------------------------------------- : -
Ipe : GCAGCCAATGCTTGTCAAAAAGCGTTTGTAACTAATCGTGTAGGCGATTTCGGTCTATTATTAGGAATTTTGTCTTTTTATTGGATAAC : 1682
Ici : ----------------------------------------------------------------------------------------- : -
Isc : ----------------------------------------------------------------------------------------- : -
Ath : ----------------------------------------------------------------------------------------- : -

1700 * 1720 * 1740 * 1760 * 1780
Ikl : ----------------------------------------------------------------------------------------- : -
[truncated: 509,231 more chars]
